# Decoding skeletal diversity and complexity: A biomimetic and photochemical remodeling strategy

**DOI:** 10.1126/sciadv.aeh0883

**Published:** 2026-06-24

**Authors:** Quan Xu, Yuan Cai, Qiong Wu, Zhenghui Huang, Ze-Nan Yang, Chang-Wei Shao, Hong-Xia Dai, Wang-Yang Du, Xiang-Yang Zhang, Yue-Wei Guo, Lubin Jiang, Xu-Wen Li

**Affiliations:** ^1^ State Key Laboratory of Chemical Biology, Shanghai Institute of Materia Medica, Chinese Academy of Sciences, Shanghai 201203, China.; ^2^ Shandong Laboratory of Yantai Drug Discovery, Bohai rim Advanced Research Institute for Drug Discovery, Yantai 264117, China.; ^3^ School of Pharmaceutical Science and Technology, Faculty of Medicine, Tianjin University, Tianjin 300072, China.; ^4^ University of Chinese Academy of Sciences, Beijing 100049, China.

## Abstract

Constructing three-dimensional polycyclic scaffolds from a single precursor is a long-standing challenge in organic synthesis. Inspired by our proposed biosynthetic pathway of marine natural product ocellatusone C (**1**), we achieved its concise biomimetic synthesis from tridachiahydropyrone (**2**) through an acid-triggered cascade featuring a Dieckmann-type cyclization. This transformation further established a platform for skeletal diversification, yielding a series of [3.3.1] bicyclic analogs with broad functional tolerance. Guided by density functional theory (DFT) calculations, we uncovered a light-induced skeletal reorganization that efficiently generates elusive polycyclic architectures. Mechanistic investigations integrating DFT calculations and quasi-classical dynamics simulations revealed that selectivity in a critical vinylcyclopropane-cyclopentene rearrangement arises from post-spin crossing dynamic effects. Cross-coupling further expanded these scaffolds into a focused library of complex molecules. The bioassays indicated that these architecturally diverse systems have substantial antimalarial bioactivity, highlighting their potential as drug leads.

## Teaser

A unified biomimetic and photochemical strategy builds diverse 3D polycyclic frameworks and delivers potent antimalarial leads.

## INTRODUCTION

The three-dimensional (3D) architecture of organic molecules dictates their biological function and material properties (*1*, *2*). Polycyclic natural products, with their sp^3^-rich and conformationally constrained frameworks, epitomize this structural complexity and often exhibit potent activities (*3*–*7*). However, constructing these congested carbocycles remains one of the most persistent challenges in synthesis, requiring precise stereocontrol, management of strain, and difficult cyclizations (*7*, *8*). This challenge is exacerbated when the target is not a single molecule but a family of skeletally diverse isomers (*9*, *10*), where small topological changes can transform biological activity (*11*–*13*). Strategies that directly interconvert these frameworks are rare, forcing chemists into inefficient, target-specific synthetic routes that limit systematic structure-activity relationship (SAR) exploration (*9*, *10*). Nature, by contrast, assembles complex architectures with unparalleled efficiency through biosynthetic cascades. Biomimetic synthesis captures this logic, using inherent reactivity to streamline construction of natural products (*14*–*18*). While traditionally focused on replicating one specific pathway, its potential for divergent skeletal diversification remains underexplored. Such an approach could provide access to families of shape-diverse scaffolds from a common precursor, directly addressing the demand for unconventional, lead-like architectures in drug discovery (*19*, *20*).

**Fig. 1.**
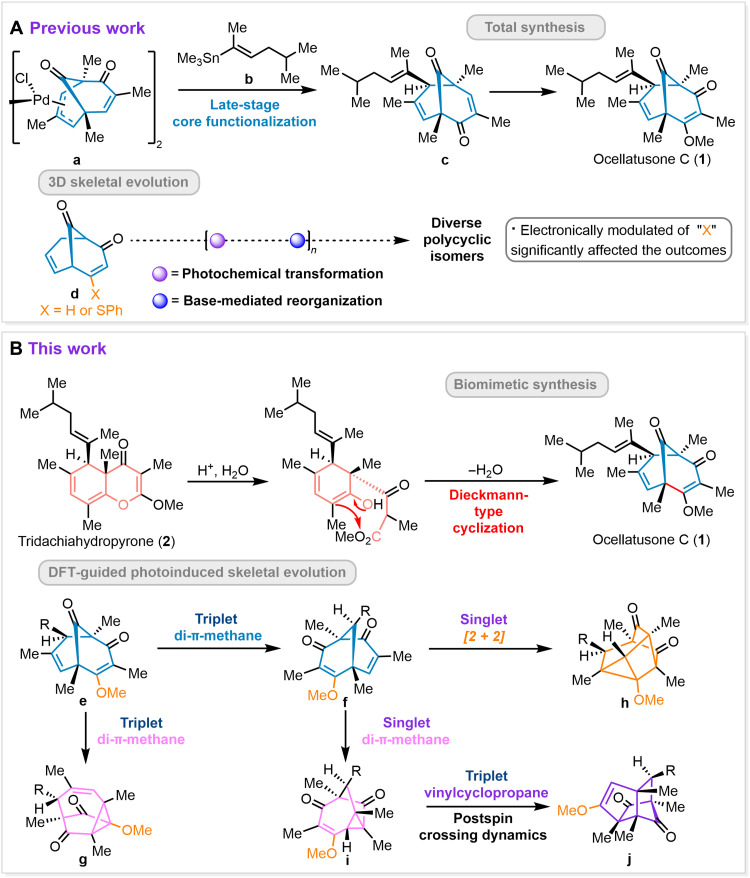
Total synthesis of ocellatusone C (1) and late-stage skeletal evolution. (**A**) Total synthesis and 3D skeletal evolution of ocellatusone C by Maimone and co-workers. (**B**) This work reports the biomimetic synthesis of ocellatusone C and its photoinduced skeletal evolution.

The ocean continues to yield structurally distinct marine natural products (MNPs) that inspire new strategies for chemical synthesis and drug discovery. Our research group has long pursued bioactive MNPs and their biomimetic synthesis as a route to drug leads (*21*, *22*). This ongoing effort led to the discovery of ocellatusone C (**1**) in 2020, a polyketide featuring a previously unreported bridged bicyclo[3.3.1]nonane core from the photosynthetic mollusk *Placobranchus ocellatus* (*23*). Its unusual architecture is hypothesized to arise from skeletal rearrangement of the γ-pyrone precursor tridachiahydropyrone (**2**), presenting a compelling opportunity for synthetic validation (*23*). Subsequently, Sanchez and Maimone (*24*) reported a nice total synthesis of **1** by leveraging dynamic anion chemistry for late-stage functionalization of a preconstructed core (Fig. 1A). In contrast, we pursued a complementary biomimetic strategy that starts from the γ-pyrone **2** and uses a key Dieckmann-type cyclization to orchestrate late-stage skeletal reorganization. This approach efficiently constructing the target framework and provides direct experimental support for its biosynthetic origin (Fig. 1B).

The inherent photolability of the ocellatusone C framework (*25*) motivated us to explore its potential for photocatalytic skeletal diversification. Building on Maimone’s demonstration that homoconjugated bicyclic cores can undergo light- and base-mediated rearrangements with substituent-dependent outcomes (Fig. 1A) (*25*), we investigated the effect of a methoxy group on the photochemical evolution of the core. Continuous irradiation of methoxy-functionalized analogs yielded a family of uncommon polycyclic scaffolds, expanding structural diversity far beyond the natural product (Fig. 1B). Our transformation proceeded through an unusual vinylcyclopropane-cyclopentene (VCP-CP) rearrangement, unveiling by density functional theory (DFT) calculations and quasi-classical dynamics simulations. Computations revealed that selectivity was dictated not by classical transition–state energetics but by post-spin crossing dynamic effects (*26*). To our knowledge, this represents the first example where these spin-state dynamics, rather than conventional orbital symmetry rules or kinetic control, govern the outcome of a complex VCP-CP rearrangement within a natural product–derived polycyclic system. These findings underscore how biomimetic synthesis, coupled with photochemical diversification, can unlock unconventional reactivity and rapidly expand access to complex 3D architectures.

## RESULTS

### Biomimetic synthesis and mechanistic insights of ocellatusone C from tridachiahydropyrone

**Fig. 2.**
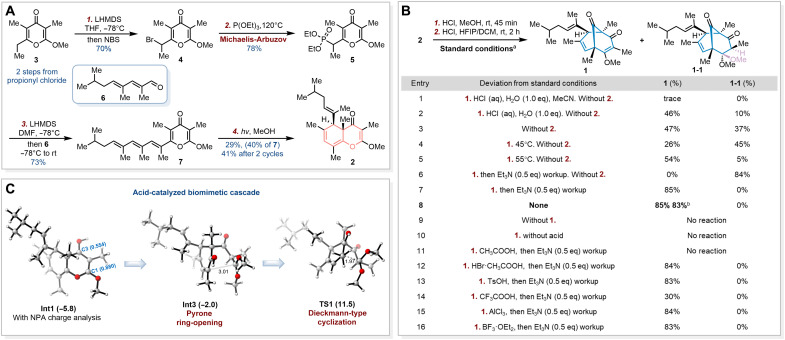
Biomimetic synthesis and mechanistic study of ocellatusone C (1). (**A**) Total synthesis of biosynthetic precursor tridachiahydropyrone (**2**). DMF, *N*,*N*′-dimethylformamide. (**B**) Optimization of the reaction conditions for the acid-catalyzed biomimetic rearrangement of ocellatusone C (**1**). MeCN, acetonitrile. (**C**) Optimized geometries for key intermediates and transition states in acid-cascaded biomimetic pathway [bond lengths in angstroms and relative Gibbs free energies (Δ*G*) in kilocalories per mole]. The values in blue parentheses represent the natural population analysis (NPA) charges for the relevant atoms, while the values in black parentheses represent the relative Gibbs free energies. ^a^Standard conditions: 1. **2** (5 mg, 1.0 eq.), HCl·methanol (MeOH) (10 mol %) as an acid catalyst, methanol (0.1 M), room temperature (rt), 45 min. 2. HCl·ethyl acetate (EtOAc) (5 mol %), HFIP/DCM (4:1, 0.1 M), room temperature, 2 hours (h). ^b^Isolated yields.

Our synthesis began with the preparation of the key biosynthetic precursor, tridachiahydropyrone (**2**), a γ-pyrone polypropionate accessible through a streamlined approach from commercially available propanoyl chloride (Fig. 2A). Following a reported two-step procedure, intermediate **3** was prepared efficiently on gram scale (*27*, *28*). Initial attempts at direct α-bromination of **3** with lithium hexamethyldisilazide (LHMDS) and *N*-bromosuccinimide (NBS) led mainly to dimerization (see the Supplementary Materials, part 1), with only trace formation of the desired monobromide **4**. This problem was solved by reversing the addition sequence: Slow addition of the preformed lithium enolate of **3** to a solution of NBS in tetrahydrofuran (THF) at −78°C effectively suppressed dimerization and afforded bromide **4** in 70% yield. Subsequent transformation proceeded smoothly: A Michaelis-Arbuzov reaction provided phosphonate ester **5** in 78% yield, Horner-Wadsworth-Emmons olefination with aldehyde **6** furnished the conjugated polyene **7** in 73% yield, and photochemical 6π-conrotatory electrocyclization delivered tridachiahydropyrone (**2**) in 29% yield with 40% recovery of the starting material (*29*); after two recycling cycles, **2** was obtained in 41% isolated yield.

With tridachiahydropyrone (**2**) in hand, we next pursued its biomimetic conversion to ocellatusone C (**1**) guided by our biosynthetic hypothesis (Fig. 2B). We envisioned that acid-promoted ring opening of the γ-pyrone in **2** would generate a key β-keto ester intermediate poised for intramolecular Dieckmann-type cyclization to form the [3.3.1] bicyclic framework of **1** (fig. S7) (*23*). Initial efforts using HCl as a catalyst with 0.1 equivalent of water in acetonitrile (entry 1), as well as in dichloromethane (DCM), THF, or dioxane (entries S1 to S3; table S1), afforded only trace amounts of **1** alongside substantial decomposition of **2**. Switching to methanol as the solvent delivered **1** in 46% yield (entry 2), suggesting that methanol acts as a more effective nucleophile than water in facilitating pyrone opening. This hypothesis was confirmed under rigorously anhydrous conditions, which further improved reaction efficiency (entry 3). The structural elucidation of a methanol adduct **1-1** confirmed its direct involvement in the cascade.

Efforts on the conversion of **1-1** to the final natural product **1** revealed that simple heating was ineffective (entries 4 and 5), whereas quenching the reaction with triethylamine cleanly afforded **1-1** in 84% yield (entry 6). Notably, **1-1** slowly evolved to **1** in CDCl_3_ (fig. S2), indicating that mild acidity facilitates methanol elimination. Inspired by the distinct properties of hexafluoroisopropanol (HFIP), a strong hydrogen bond donor with mild acidity (*30*–*33*), we treated the reaction mixture containing **1-1** with 5 mol % HCl in HFIP/DCM (4:1), enabling complete conversion to **1** within 2 hours (entry 7). A streamlined one-pot procedure omitting the triethylamine quench also proved highly effective, delivering **1** in 83% isolated yield (entry 8). Control experiments underscored the essential roles of both acid and methanol in this transformation (entries 9 and 10), and broader screening showed that several Brønsted and Lewis acids efficiently mediated the rearrangement (entries 11 to 16). Together, these results support the hypothesis that tridachiahydropyrone (**2**) is a plausible biosynthetic precursor of ocellatusone C (**1**) and uncover a previously unreported acid-catalyzed cascade that rapidly constructs the complex [3.3.1]-bridged framework. This transformation provides an alternative strategic entry point for the synthesis of architecturally demanding natural products.

To elucidate the mechanism of the biomimetic skeletal rearrangement from **2** to **1**, we first performed deuterium-labeling experiments using CD_3_OD in place of methanol as the solvent, which led to near-complete deuterium incorporation into the methoxy groups of both intermediate **1-1** and final product **1** (fig. S1). This result indicates a rapid and reversible nucleophilic addition-elimination at the α,β-unsaturated ketone under acidic conditions. DFT calculations (Fig. 2C and fig. S8) further delineated the catalytic cycle. Protonation of the carbonyl oxygen in **2** generates a key cationic intermediate Int1 stabilized by resonance delocalization of the positive charge. Nucleophilic attack by methanol triggers the pyrone ring opening and formation of the acetal oxocarbenium ion intermediate Int3, which undergoes an intramolecular Dieckmann-type cyclization via transition state TS1 with a modest energy barrier of 13.5 kcal/mol to form the bicyclic intermediate Int4. Subsequent deprotonation affords the methanol adduct **1-1**, which lastly eliminates methanol to deliver the thermodynamically stable natural product ocellatusone C (**1**). Collectively, the experimental and computational results reveal a previously undescribed acid-promoted cascade featuring pyrone ring-opening and Dieckmann-type cyclization as the key steps governing skeletal reorganization.

### Substrate scope and structural diversification

**Fig. 3.**
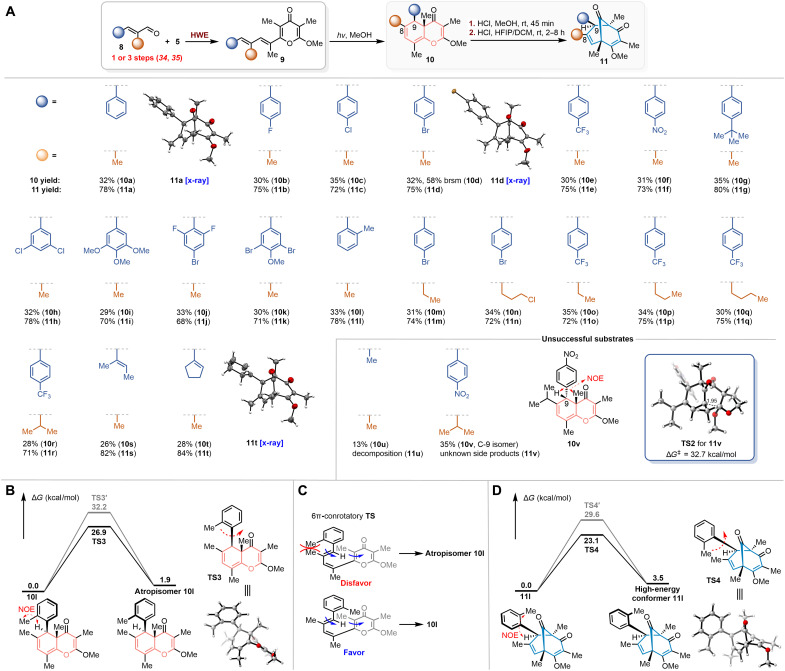
Substrate scope of the biomimetic transformation. (**A**) Synthesis of ocellatusone C analogs via acid-catalyzed biomimetic rearrangement. HWE, Horner-Wadsworth-Emmons. (**B**) Free energy profile of the conversion of **10l** to atropisomer **10l**. (**C**) Proposed mechanism for the selective photochemical formation of **10l**. (**D**) Free energy profile of the conversion of **11l** to high energy conformer **11l**. Bond lengths in angstoms and relative Gibbs free energies (Δ*G*) in kilocalories per mole. Ortep diagram of compounds **11a**, **11d** and **11t** with 50% probability thermal ellipsoids.

We next evaluate the generality of our acid-catalyzed biomimetic rearrangement for constructing the [3.3.1] bicyclic framework and for diversifying the molecular architecture of ocellatusone C (Fig. 3A). Introduction of aryl substituents at the C-9 side chain was achieved by coupling aldehyde **8**, synthesized via a modified literature approach to incorporate the arene motif (*34*, *35*), with phosphonate ester **5** to provide polyene precursor **9**, which underwent photochemical 6π-conrotatory electrocyclization to deliver a series of tridachiahydropyrone analogs **10a** to **10l**. All aryl-modified substrates **10a** to **10l** rearranged smoothly to the corresponding [3.3.1] bicycles **11a** to **11l** in good yields (68 to 80%), demonstrating broad functional group tolerance, including halogenated (**11b** to **11d)**, electron-withdrawing (**11e** and **11f**), electron-donating (**11g** and **11l**), and multisubstituted arenes (**11h** to **11k**). Modifications at the C-8 position were also well accommodated, as replacing the methyl group with ethyl (**11m** and **11o**), propyl (**11p**), butyl (**11q**), isopropyl (**11r**), and even 1-chloropropyl (**11n**) substituents all delivered the desired products in 71 to 75% isolated yield. Extension of the C-9 chain similarly gave **11s** and **11t**, further attesting to the flexibility of the cascade. In contrast, truncation of the C-9 side chain to a methyl group (**10u**) substantially reduced photocyclization efficiency (13%) and abolished downstream rearrangement, highlighting the importance of π-conjugation for productive reactivity. A more notable case arose with precursor **10v**, incorporating a *p*-nitrophenyl group at C-9 and an isopropyl unit at C-8. This substrate formed as a C-9 epimer via photoinduced *E/Z* isomerization (fig. S9) and proved resistant to rearrangement under standard conditions. DFT calculations revealed a prohibitive barrier (32.7 kcal/mol) for the Dieckmann cyclization step, emphasizing the essential role of stereochemistry alignment at C-9 for successful biomimetic transformation.

Structural characterization of the [3.3.1] bicyclic derivatives (series **11**) revealed anomalous nuclear magnetic resonance (NMR) behavior associated with the aromatic rings: pronounced signal broadening in ^1^H NMR spectra and extensive line broadening or loss of signals in ^13^C NMR spectra (fig. S15). Single-crystal x-ray structures of **11a** and **11d** unambiguously confirmed the molecular frameworks, ruling out misassignment. We attributed the NMR anomalies to restricted, but not fully frozen, rotation of the aryl ring, resulting in dynamic broadening on the NMR timescale (*36*). To test this hypothesis, we introduced *ortho*-methyl substitution on the arene ring, anticipating that steric interaction would increase the torsional barrier and potentially stabilize atropisomers. The corresponding photocyclization product **10l** was obtained cleanly as a single atropisomer. Nuclear Overhauser effect (NOE) correlations established that the C-9 hydrogen and the *ortho*-methyl group of benzene ring occupy the same face of the bicyclic framework (Fig. 3B). DFT calculations revealed a rotational barrier of 26.9 kcal/mol for the aryl axis in **10l**, consistent with configurational stability. We rationalize the stereochemical outcome of the 6π-electrocyclization through a transition state model in which the *ortho*-methyl group aligns *syn* to the C-9 hydrogen to alleviate steric repulsion (Fig. 3C and fig. S12). Notably, the acid-catalyzed rearrangement of **10l** to **11l** proceeded with complete retention of the aryl conformation. NOE experiments again confirmed *syn* disposition of the hydrogen at C-9 and *ortho*-methyl group (Fig. 3D). DFT analysis indicated that the rotational barrier in **11l** remains high (23.1 kcal/mol), effectively locking the arene conformation. As expected, **11l** exhibited sharp, well-resolved NMR signals, confirming that steric engineering successfully suppressed the dynamic behavior and validated our initial hypothesis. Collectively, beyond demonstrating the broad applicability of the acid-catalyzed biomimetic rearrangement for diversifying the ocellatusone C scaffold, we uncovered and rationally controlled conformational dynamics within the [3.3.1] framework. These findings not only expand the toolbox for skeletal reorganization in natural product synthesis but also open avenues toward the design and synthesis of atropisomeric scaffolds with potential applications in chiral catalysis and functional materials.

### DFT-guided photoinduced skeleton evolution

**Fig. 4.**
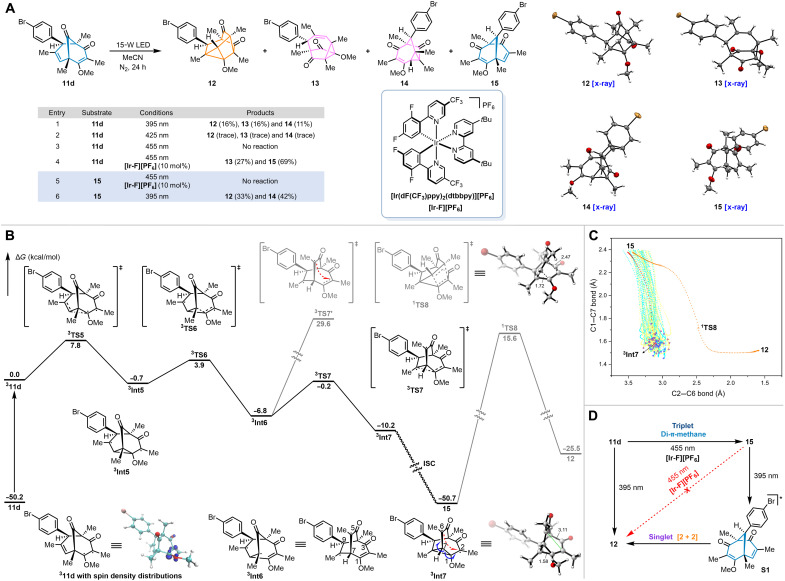
DFT-guided photoinduced skeleton evolution. (**A**) Screening of reaction conditions for the photoinduced skeletal diversification and evolution. Ortep diagram of compounds **12** to **15** with 20% probability thermal ellipsoids. (**B**) Calculated energy profile of the photoinduced triplet state reaction pathway of **11d** at the (U)B3LYP-D3BJ/Def2-TZVP/SMD(MeCN)//(U)B3LYP-D3BJ/Def2-SVP/SMD(MeCN) level of theory. Bond lengths in angstroms and relative Gibbs free energies (Δ*G*) in kilocalories per mole. ISC, intersystem crossing. (**C**) The study of dynamic behaviors of ^3^Int7. Calculated at the (U)B3LYP-D3BJ/def2-SVP/SMD(MeCN) level of theory. All the paths and trajectories are projected to the plane with respect to the C2─C6 and C1─C7 distances in angstroms. Spontaneous relaxation path for ^3^Int7 structure on the open- or closed-shell singlet state and the intrinsic reaction coordinate (IRC) path for ^1^TS8 are shown by red and orange curves, respectively. The overlays of the Born-Oppenheimer molecular dynamics (BOMD) simulation trajectories with open-shell singlet (OSS) sampling and triplet (T) sampling are shown by green and yellow curves, respectively. The red and orange triangles show the positions of ^3^Int7 (3.11, 1.58) and ^1^TS8 (2.47, 1.72), respectively. The blue and pink circles show the positions of initial geometries for all OSS sampling and T sampling trajectories, respectively. (**D**) Proposed mechanism for the photoinduced skeletal evolution of **11d** to **12** and **15**.

The [3.3.1] bicyclic framework, noted for its distinctive photosensitivity and responsiveness to peripheral electronic effects (*25*), offers a versatile platform for structural diversification. Building on our developed acid-catalyzed biomimetic rearrangement strategy, we turned to compound **11d**, a side-chain analog of the natural product ocellatusone C featuring an aromatic substitution. Upon irradiation at 395 nm, a wavelength previously established to promote [3.3.1] skeletal rearrangements (*25*), **11d** underwent an unanticipated photoreaction (Fig. 4A, entry 1). Instead of the expected products (*25*), three architectures emerged: a tetracyclo[3.3.1.0^2,8^.0^3,7^]nonane (**12**) and two tricyclo[4.2.1.0^2,8^]nonane derivatives (**13** and **14**), isolated in 16, 16, and 11% yields, respectively. The formation of the tetracyclic framework in **12** is particularly notable, as it represents a previously inaccessible transformation from the [3.3.1] scaffold. Its cage-like topology not only expands the repertoire of photoinduced skeletal reorganization but also motivated further mechanistic analysis guided by DFT calculations.

To elucidate the pathway leading to **12** (fig. S16), we mapped the triplet potential energy surface of **11d** by DFT (Fig. 4B). The excited triplet readily undergoes a di-π-methane (DPM) rearrangement via transition state ^3^TS5 with a modest barrier of 7.8 kcal/mol, affording intermediate ^3^Int5. Subsequent ring opening of the strained tricyclic system yields the more stable α,β-unsaturated ^3^Int6, followed by radical attack of C-8 on C-1 to form key intermediate ^3^Int7, which was expected to recombine into **12**. However, spontaneous relaxation simulations revealed a strong thermodynamic preference for tension release, leading instead to compound **15**. To further exclude the possibility of product control via conformation, a behavior recently termed the “post-spin crossing dynamics effect” (*26*), we performed Born-Oppenheimer molecular dynamics (BOMD) simulations (Fig. 4C) (*26*, *37*, *38*). All trajectories, including 30 initiated from the triplet state and 30 from the open-shell singlet (OSS) state, consistently converged to **15**, with no instances of **12** formation. These results indicated that **12** could not arise from the triplet pathway and suggested a singlet-state origin. In addition, there should be some undetected or overlooked photoreaction products, such as the triplet-derived compound **15**.

We next enforced triplet reactivity experimentally by using **11d** (*39*). Initial wavelength screening revealed that **11d** was photoinactive at 455 nm (entry 3), but with [Ir(dF(CF_3_)ppy)_2_(dtbbpy)][PF_6_] as the triplet sensitizer (*40*), **11d** underwent efficient transformation, affording the predicted triplet product **15** in 69% yield, alongside product **13** (27% yield). A mechanistic scheme illustrating the proposed pathway for the formation of **13** under triplet excitation is provided in fig. S19. Notably, **15** proved photostable under triplet-sensitized conditions (entry 5). DFT analysis revealed that the T_1_ state of **15** corresponds to intermediate ^3^Int7, which undergoes spontaneous relaxation exclusively back to **15**, effectively shutting down further triplet reactivity (fig. S20).

Direct irradiation at 395 nm demonstrated that **15** undergoes photoconversion to afford **12** and **14** in 33 and 42% yields, respectively (entry 6). These results support a bifurcated reaction mechanism: Triplet excitation drives **11d** to **15** via a triplet-sensitized DPM rearrangement, while subsequent single-state photochemistry of **15** diverges, producing **12** through an intramolecular [2 + 2] cyclization (Fig. 4D) and **14** through a distinct singlet-state DPM rearrangement (fig. S25).

Together, these studies demonstrate how integrating computation with controlled photochemical conditions enables programmable skeletal evolution of the [3.3.1] framework. The ability to access otherwise unattainable cage-like architectures highlights the broader potential of mechanism-driven design in synthetic photochemistry.

### Post-spin crossing dynamics in the photoinduced VCP-CP rearrangement

**Fig. 5.**
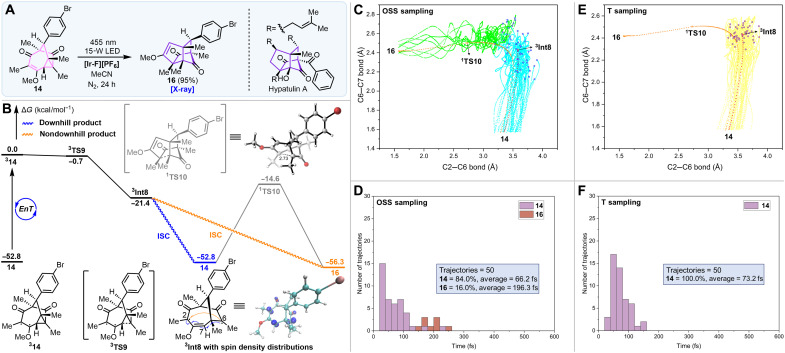
Post-spin crossing dynamics in the photoinduced VCP-CP rearrangement of 14 to 16. (**A**) Photoinduced VCP-CP rearrangement of **14** to **16**. (**B**) Calculated energy profile of the photoinduced VCP-CP rearrangement of **14** to **16**. Bond lengths in angstroms and relative Gibbs free energies (Δ*G*) in kilocalories per mole. The study of dynamic behaviors of ^3^Int8 based on OSS sampling (**C**) and T sampling (**E**). All the paths and trajectories are projected to the plane with respect to the C2─C6 and C6─C7 distances in angstroms. Spontaneous relaxation path for ^3^Int8 structure on the open- or closed-shell singlet state and the IRC path for ^1^TS10 are shown by red and orange curves, respectively. The overlays of the BOMD simulation trajectories with OSS sampling (C) are shown by green (leading to **16**) and cyan (leading to **14**) curves. The overlays of the BOMD simulation trajectories with T sampling (E) are shown by yellow curves. The red and orange triangles show the positions of ^3^Int8 (3.58, 2.43) and ^1^TS10 (2.73, 2.50), respectively. The blue and pink circles show the positions of initial geometries for all OSS sampling (C) and T sampling (E) trajectories, respectively. Product formation timing histograms of OSS sampling (**D**) and T sampling (**F**). Trajectories were sorted into 20-fs wide bins, with purple bins corresponding to trajectories forming **14** and reddish brown bins corresponding to trajectories forming **16**.

Building on the skeletal plasticity of the [3.3.1] framework, we next examined compound **14**, which retains an α,β-unsaturated carbonyl motif with high photoreactivity. Irradiation of **14** led to a high-yielding (95%) VCP-CP rearrangement toward **16** (Fig. 5A), whose tricyclic octahydro-1,5-methanopentalene core is identical to that of the natural product hypatulin A (*41*). Given the scarcity of documented photomediated VCP-CP rearrangements (*42*), we used DFT calculations to gain further mechanistic insight.

DFT calculations revealed that photoexcitation of **14** undergoes ring opening of the strained tricyclic system via transition state ^3^TS9 to form the triplet diradical intermediate ^3^Int8 (Fig. 5B). Spontaneous relaxation of ^3^Int8 results in recombination of the C-6 and C-7 radicals, regenerating the starting material **14**. A direct pathway from **14** to **16** via an OSS transition state ^1^TS10 was ruled out because of a high energy barrier (38.2 kcal/mol), indicating that the relaxation outcome of ^3^Int8 might be conformationally controlled. BOMD simulations were then performed by sampling 50 trajectories from the OSS (Fig. 5C) and 50 from triplet states (Fig. 5E) of ^3^Int8. Notably, eight trajectories (16%) originating from the OSS state led to **16**, whereas all triplet trajectories reverted to **14**. We attribute this divergence to the more dispersed conformational distribution in OSS sampling compared to T sampling (see blue and pink circles in Fig. 5, C and E, respectively). Moreover, the timescales for reversion to **14** were considerably shorter (66.2 fs for OSS sampling and 73.2 fs for T sampling; Fig. 5, D and F) than the formation of **16** (196.3 fs; Fig. 5D). These results indicate a mechanism that photoexcited **14** forms diradical intermediate ^3^Int8, whose relaxation not only predominantly regenerates the starting material but also leads to minor quantities of **16**. Recycling of **14** through repeated excitation-relaxation cycles accounts for the high overall yield of **16** (fig. S26). Moreover, BOMD simulations revealed a general conformational guideline governing product selectivity: Among the sampled initial geometries of ^3^Int8 (blue circles in Fig. 5C), those with a shorter C2─C6 distance and a longer C6─C7 distance preferentially lead to **16**, whereas the opposite conformational preference favors reversion to **14**. This indicates that the geometric conformation of the photogenerated diradical intermediate plays a crucial role in determining the product outcome. Thus, the VCP-CP rearrangement described here is governed by post-spin crossing dynamics, diverging from conventional thermodynamic or kinetic control paradigms (*26*) and underscoring the essential role of ultrafast dynamics in steering photochemical skeletal reorganization.

### Diversification of polycyclic architectures and antimalarial activity evaluation

**Fig. 6.**
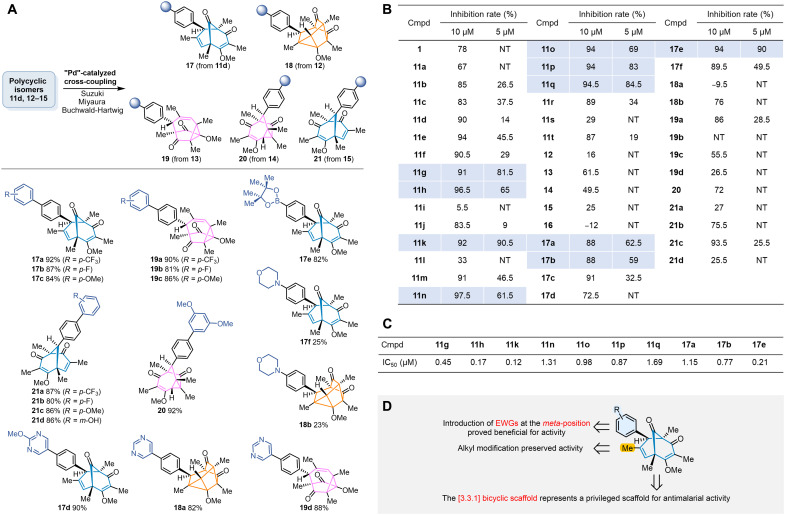
Diversification of polycyclic architectures and antimalarial activity evaluation. (**A**) Diversification of polycyclic architectures via Pd-catalyzed cross-coupling. See the Supplementary Materials for detailed procedures. (**B**) Antimalarial activity assay of polycyclic compounds (Cmpd). NT indicates “not tested.” (**C**) Antimalarial IC_50_ values of polycyclic compounds. (**D**) SAR analysis of polycyclic compounds for antimalarial activity. EWGs, electron-withdrawing groups.

To explore the potential of these architecturally distinct scaffolds for late-stage diversification, we evaluated the compatibility of representative compounds (**11d** and **12** to **15**) with cross-coupling reactions (Fig. 6A and see the Supplementary Materials, part 6). Suzuki coupling proved broadly effective, delivering biphenyl-functionalized derivatives (**17a** to **17c**, **19a** to **19c**, **20**, and **21a** to **21d**) and phenylpyrimidine analogs (**17d**, **18a**, and **19d**) in excellent yields (80 to 92%). Miyaura borylation of **11d** provided the corresponding boronate ester **17e** in 82% yield, and Buchwald-Hartwig amination afforded C─N coupling products **17f** and **18b** in moderate yields. Capitalizing on the efficient diversification enabled by these coupling reactions, we next evaluated the antimalarial activity of the resulting 3D polycyclic systems (**11** to **21**).

Biological screening revealed that the ocellatusone C analogs **11g**, **11h**, **11k**, **11n**, **11o**, **11p**, **11q**, **17a**, **17b**, and **17e** exhibited substantial antimalarial activity with median inhibitory concentration (IC_50_) values ranging from 0.12 to 1.69 μM (Fig. 6, B and C). SAR analysis indicated that the [3.3.1] bicyclic scaffold of the natural product ocellatusone C has superior antimalarial potential comparing to other polycyclic frameworks. Among the ocellatusone C analogs with a phenyl ring at C-9, the introduction of electron-withdrawing groups at the *meta*-position was beneficial for activity and vice versa (compounds **11k** and **11h** versus **11i**). In addition, modifying the methyl group at the C-8 position of the ocellatusone C scaffold to other alkyl groups did not result in substantial changes in antimalarial activity (Fig. 6D). It should be noted that all analogs were evaluated as racemic mixtures and future studies with single enantiomers may reveal enhanced antimalarial activity. Collectively, this study offers valuable insights for developing alternative molecular scaffolds with antimalarial lead potential, and the most bioactive compounds **11h**, **11k**, and **17e**, with IC_50_ values of 0.17, 0.12, and 0.21 respectively, are worth for further medicinal investigation.

## DISCUSSION

This work establishes a unified strategy for the synthesis and diversification of complex natural product derived polycyclic scaffolds. A biomimetic synthesis of ocellatusone C (**1**) not only validates its proposed biosynthetic origin but also furnishes a versatile platform for skeletal diversification. Harnessing the intrinsic photoreactivity of the [3.3.1] framework enabled controlled skeletal evolution, transforming a single natural product–like core into an array of structurally diverse and synthetically challenging cages and fused ring systems.

A key mechanistic advance of this study is the elucidation of a photochemical VCP-CP rearrangement governed by post-spin crossing dynamics, in which product selectivity is dictated by nonstatistical relaxation of a common diradical intermediate rather than by conventional kinetic or thermodynamic control. This rare example underscores the broader role of ultrafast dynamics in shaping photochemical outcomes.

Last, the demonstrated compatibility of these scaffolds with late-stage cross-coupling provides a direct avenue for exploring SARs. Together with the biological activity data demonstrating the substantial antimalarial potency of several compounds with [3.3.1] bicyclic scaffold (**11h**, **11k**, and **17e**), these results highlight the potential of this approach to expand molecular shape space for drug discovery and functional materials. By integrating biosynthetic logic, mechanistic insight, and photochemical design, this work offers a paradigm for not merely emulating nature but extending it by opening access to underexplored regions of polycyclic chemical space.

## MATERIALS AND METHODS

### General experimental procedures

All reagents and solvents were purchased from common commercial suppliers and were used without further purification. Photochemical 6π-conrotatory electrocyclization reactions were conducted using a 250-W long-arc mercury lamp. Photoinduced skeletal rearrangement reactions were performed in a parallel light reactor (RLH-18, ROGER Technology Limited) equipped with 15-W light-emitting diode (LED) lamps emitting at 395 nm (violet), 425 nm (deep blue), and 455 nm (azure). All irradiations were carried out in borosilicate glass vessels. Unless otherwise specified, reactions were conducted under a nitrogen atmosphere. ^1^H NMR spectra were recorded at 400, 500, and 600 MHz, and ^13^C NMR were recorded at 100, 125, and 150 MHz on a Bruker NMR spectrometer instrument (Avance III 400, Avance III 500 and Avance III 600, Bruker Biospin AG, Uster, Switzerland). Chemical shifts are reported with the residual CHCl_3_ [δ_H_ = 7.26 parts per million (ppm); δ_C_ = 77.16 ppm] as the internal standard for ^1^H and ^13^C NMR spectra. All coupling constants (*J* values) are reported in hertz. NMR abbreviations are as follows: s, singlet; d, doublet; t, triplet; q, quartet; m, multiplet; dd, doublet of doublets; dt, doublet of triplets; dq, doublet of quartets; td, triplet of doublets; hept, heptet; and br, broad. High-resolution electrospray ionization mass spectrometry spectra were recorded on Waters VION IMS QTOF and Agilent G6250 Q-TOF (Agilent, Santa Clara, CA, USA). X-ray analyses were carried out on a Bruker APEX II CCD diffractometer with Cu Kα radiation (*l* = 1.54178 Å). Reversed-phase high-performance liquid chromatography was performed on an Agilent 1260 series liquid chromatography equipped with a 1260 DAD WR G7115A detector at 210 and 254 nm. An Agilent semipreparative XDB-C18 column (5 μm, 9.4 mm by 250 mm) was used for the purification. Thin-layer chromatography was performed on precoated silica gel plates (HSGF254, Sinopharm Chemical Reagent Co. Ltd., Shanghai, China). Column chromatography was performed using a 200- to 300-mesh silica gel (Sinopharm Chemical Reagent Co. Ltd., Shanghai, China). All solvents used for column chromatography were of analytical grade (Shanghai Chemical Reagents Co. Ltd.). For the details of synthetic process, please see the Supplementary Materials.

### Computational detail

All DFT calculations were performed using Gaussian 16 software (*43*). Optimizations of intermediates and transition states were carried out at the B3LYP (*44*, *45*)–D3BJ (*46*, *47*) level of theory with the Def2-SVP basis set, including SMD (*48*) solvation model, with unrestricted DFT used for open-shell species. Attempts to find OSS species were performed for specific structures by first performing a single-point energy calculation on a triplet surface and reading that wave function into the singlet geometry optimization as the initial guess. We subsequently confirmed that all OSS species have stable wave functions. The vibrational frequencies were also computed at the same level to confirm that the transition states have only one imaginary frequency and the intermediates have no imaginary frequency. The Gibbs free energies of the optimized structures were obtained from the thermal correction to Gibbs free energy at the SMD-B3LYP-D3BJ/Def2-SVP level and single-point energy at the SMD-B3LYP-D3BJ/Def2-TZVP level, with unrestricted DFT used for open-shell species. Molecular graphics have been produced with CYLview (*49*). Intrinsic reaction coordinate (IRC) calculations were performed to ensure that the saddle points found were true transition states connecting the reactants and the products. The geometries of ^3^Int7 and ^3^Int8 with their stable wave functions on OSS states (using the “irc = downhill” and “guess = mix” keywords in Gaussian), which provided minimal energy paths connecting to the neighboring local minima. Spin population analyses were performed using Multiwfn (*50*) and visualized with visual molecular dynamics (VMD) (*51*).

Photoinitiated π → π* and n → π* excitation calculations. Photoinitiated excitation calculations were performed using Gaussian 16 software with the time-dependent DFT (TD-DFT), and the calculation began with the optimization of the single-crystal structure of compound **11d**, **15**, and **14**. The vertical excitation (S_0_ → S_1_) was calculated by TD-DFT using td (singlets) with B3LYP-D3BJ/Def2-TZVP with SMD solvation model of acetonitrile at singlet state, and that for S_0_ → T_1_ was calculated using td (triplets) with the same level of theory at singlet state. The minima for T_1_ was optimized with UB3LYP-D3BJ/Def2-SVP in SMD (acetonitrile) at triplet state. The electron-hole analysis was used to judge the excitation type of S_1_ and T_1_ by Multiwfn, and the results were visualized by VMD.

Quasi-classical BOMD simulations were conducted in a manner similar to Zhu and Zheng (*26*), at the UB3LYP-D3BJ/Def2-SVP level of theory with SMD solvation model of acetonitrile (using the “bomd” keyword in Gaussian). Initial conditions were generated for both ^3^Int7 and ^3^Int8 via normal mode sampling at 298 K on their respective OSS and triplet state, creating distinct OSS sampling and T sampling ensembles for each species. The simulations were conducted as follows: For ^3^Int7, 30 trajectories were propagated from the OSS sampling ensemble, and 30 were propagated from the T sampling ensemble; and for ^3^Int8, 50 trajectories were propagated from the OSS sampling ensemble, and 50 were propagated from the T sampling ensemble. All trajectories were propagated until the products were fully formed, the termination conditions are as follows: For product **12**, C2─C6 was less than 3.0 bohr (~1.58 Å); for product **15**, C1─C7 was more than 4.5 bohr (~2.37 Å); for product **14**, C6─C7 was less than 3.0 bohr (~1.58 Å); and for product **16**, C2─C6 was less than 3.0 bohr (~1.58 Å). For trajectories initiated from any T sampling ensemble, the wave function was first recalculated on the OSS state (using the “guess = mix, stable = opt” options in Gaussian) before propagation.

### In vitro growth inhibition assay for IC_50_ determination

A 3-day SYBR Green I growth inhibition assay was performed as described previously (*52*). Briefly, highly synchronized ring-stage parasites (1% parasitemia and 2% hematocrit) were prepared using 5% sorbitol and seeded into 96-well plates. Parasites were cultured at 37°C in a humidified atmosphere of 5% CO_2_, 5% O_2_, and 90% N_2_ for 72 hours.

In the initial compound screening, 42 test compounds were evaluated at a single concentration of 10 μM. Those exhibiting over 80% inhibition of parasite growth were advanced to secondary screening at 5 μM. Among these, compounds demonstrated sustained inhibition greater than 50% at 5 μM were subsequently selected for IC_50_ determination.

For IC_50_ determination, the selected compounds underwent a twofold serial dilution across 10 wells, starting from 10 μM. Dimethyl sulfoxide (DMSO) [0.5% (v/v) final concentration in all wells] served as a negative control.

Parasites were lysates after 72 hours of culture by adding 100 μl of lysis buffer [saponin (0.12 mg/ml), 0.12% Triton X-100, 30 mM tris-HCl, and 7.5 mM EDTA] containing 5× SYBR Green I (Invitrogen, 10,000× stock). Plates were incubated in the dark for 2 hours, and fluorescence intensity was measured using a microplate reader at 485/535 nm (excitation/emission) to quantify parasite DNA. Growth inhibition rates were calculated after normalization to DMSO control, and IC_50_ values were generated by nonlinear regression in GraphPad Prism using a log(inhibitor) versus response model. All experiments included two biological replicates, each with two technical replicates.

## Supplementary Materials

### This PDF file includes:

Supplementary Text Figs. S1 to S27 Tables S1 to S5 References
