# Supplementary material for: Decoding skeletal diversity and complexity: A biomimetic and photochemical remodeling strategy
Source: Sci Adv. 2026 Jun 24;12(26):eaeh0883. doi: 10.1126/sciadv.aeh0883 (PMC13292994; doi:10.1126/sciadv.aeh0883)
Supplement: Supplementary file 1 — Supplementary Text Figs. S1 to S27 Tables S1 to S5 References [file sciadv.aeh0883_sm.pdf]

Science Advances
AAAS

# Supplementary Materials for

## Decoding skeletal diversity and complexity: A biomimetic and photochemical remodeling strategy

Quan Xu *et al.*

Corresponding author: Xu-Wen Li, xwli@simm.ac.cn

*Sci. Adv.* **12**, eaeh0883 (2026)
DOI: 10.1126/sciadv.aeh0883

**This PDF file includes:**

Supplementary Text
Figs. S1 to S27
Tables S1 to S5
References

## Part 1. Synthesis of Tridachiahydropyrone (2)

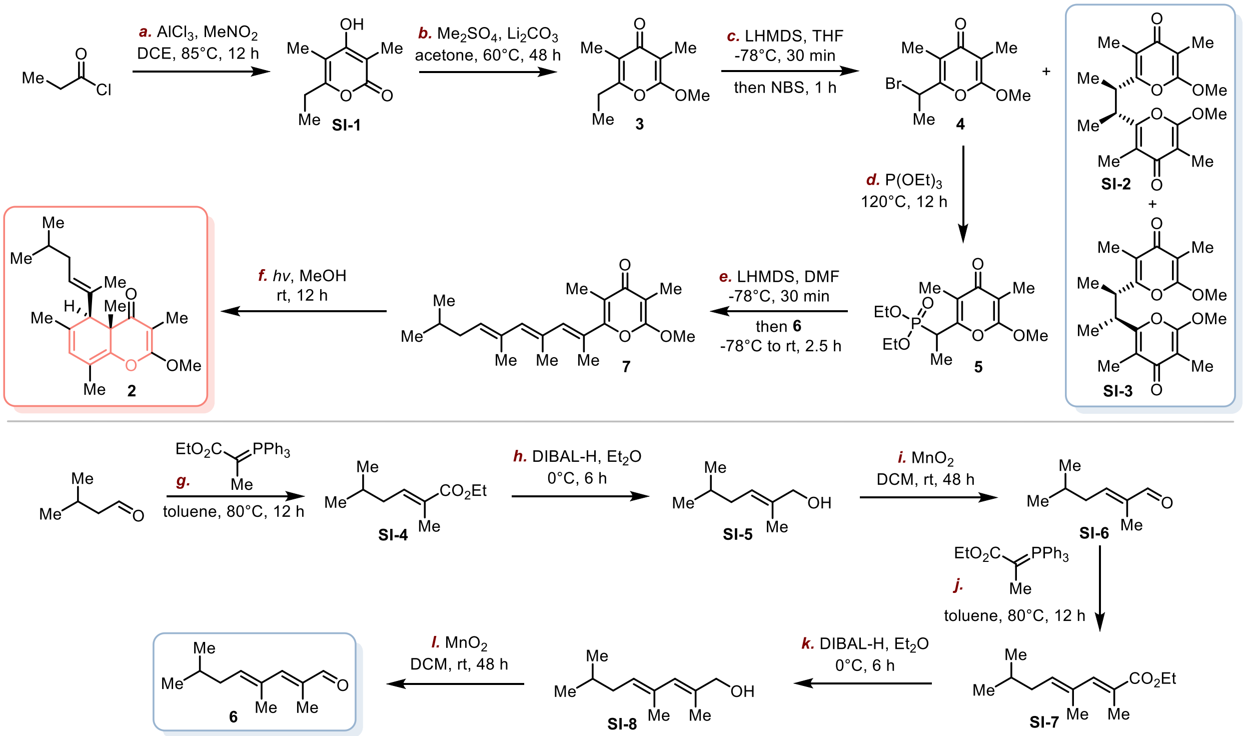

***Step A*:** A 500 mL round-bottom flask equipped with a magnetic stir bar and a reflux condenser was added $AlCl_3$ (14.41 g, 108.1 mmol, 1.0 eq.) and anhydrous 1,2-DCE (150 mL). To this mixture were successively added $MeNO_2$ (5.83 mL, 108.1 mmol, 1.0 eq.) and propionyl chloride (9.44 mL, 108.1 mmol, 1.0 eq.). The reaction vessel was evacuated and back-filled with $N_2$ (three cycles), and the mixture was heated to 85 °C in an oil bath with stirring for 12 hours. After cooling to room temperature, the reaction mixture was carefully transferred into an Erlenmeyer flask containing a 1:1 (v/v) mixture of a saturated aqueous solution of Rochelle's salt and DCM (150 mL total). The resulting mixture was stirred vigorously for 30 min. The layers were separated, and the aqueous phase was extracted with DCM (3 × 100 mL). The combined organic extracts were dried over anhydrous $Na_2SO_4$, filtered, and concentrated under reduced pressure. The crude product was purified by flash chromatography (1% to 4% MeOH in DCM) to yield pyrone **SI-1** (4.54 g, 75% yield) as a colorless solid *(27)*.

6-Ethyl-4-hydroxy-3,5-dimethyl-2*H*-pyran-2-one (**SI-1**) *(27)*. **$^1$H NMR** (400 MHz, $CDCl_3$) $\delta_H$ 2.52 (q, $J$ = 7.6 Hz, 2H), 2.00 (s, 3H), 1.96 (s, 3H), 1.17 (t, $J$ = 7.5 Hz, 3H); **$^{13}$C NMR** (101 MHz, $CDCl_3$) $\delta_C$ 167.2, 166.2, 160.0, 107.1, 98.5, 24.3, 11.8, 9.8, 8.8.

***Step B*:** A 100 mL round-bottom flask equipped with a magnetic stir bar and a reflux condenser was added pyrone **SI-1** (2.85 g, 16.95 mmol, 1.0 eq.), $Li_2CO_3$ (3.76 g, 50.84 mmol, 3.0 eq.), and dimethyl sulfate (3.21 mL, 33.89 mmol, 2.0 eq.) in acetone (30 mL). The reaction mixture was heated to 60 °C in an oil bath with stirring for 48 hours. Subsequently, the reaction was quenched by the addition of a 25% ammonium hydroxide solution (25 mL) and stirred with heating for an additional 2 hours. After cooling to room temperature, the mixture was diluted with $Et_2O$ (30 mL) and filtered through a pad of Celite. The filtrate was concentrated under reduced pressure. The

crude residue was purified by flash chromatography (20% EtOAc in PE) to afford the methylated product **3** (1.85 g, 60% yield) as a white solid *(28)*.

2-Ethyl-6-methoxy-3,5-dimethyl-4*H*-pyran-4-one (**3**) *(28)*. **$^{1}$H NMR** (600 MHz, $CDCl_3$) $\delta_H$ 3.95 (s, 3H), 2.61 (q, $J$ = 7.6 Hz, 2H), 1.93 (s, 3H), 1.83 (s, 3H), 1.22 (t, $J$ = 7.6 Hz, 3H); **$^{13}$C NMR** (151 MHz, $CDCl_3$) $\delta_C$ 181.2, 162.3, 159.3, 117.7, 99.4, 55.3, 24.3, 11.4, 9.8, 7.0.

***Step C*:** A 100 mL two-neck round-bottom flask equipped with a magnetic stir bar was added with compound **3** (1.0 g, 5.5 mmol, 1.0 eq.) in anhydrous THF (20 mL), and the mixture was purged with $N_2$. At −78 °C, LHMDS (5.5 mL of a 1 M solution in THF, 5.5 mmol, 1.0 eq.) was added dropwise over 10 minutes. Stirring was continued at the same temperature for 30 minutes, during which the solution color changed from reddish-brown to bright yellow, indicating the formation of the lithium enolate of **3**. Subsequent product distribution (**4**, **SI-2**, and **SI-3**) depended on the following conditions: under **Condition A**, a solution of NBS (1.47 g, 8.2 mmol, 1.5 eq.) in anhydrous THF (10 mL) was added dropwise to the enolate over 10 minutes at −78 °C, and the mixture was stirred for another hour; under **Condition B**, the enolate solution was added dropwise over 30 minutes into a $N_2$-purged solution of NBS (1.47 g, 8.2 mmol, 1.5 eq.) in anhydrous THF (10 mL) at –78 °C, followed by stirring for an additional hour. The reaction was quenched with saturated aqueous $NH_4Cl$ (20 mL), and the crude mixture was extracted with EtOAc (2 × 20 mL). The combined organic layers were dried over anhydrous $Na_2SO_4$, filtered, and concentrated under reduced pressure to give a brown oil, which was purified by flash chromatography (Condition A: 40% EtOAc in PE; Condition B: 20% EtOAc in PE) to afford the products: for Condition A, **SI-2** and **SI-3** (0.6 g in total, 60% combined yield); for Condition B, the bromide **4** (1.0 g, 70% yield) as a brown solid.
**Note:** Compounds **SI-2** and **SI-3** are the meso and racemic diastereomers, respectively, and are indistinguishable by NMR spectroscopy.

2-(1-Bromoethyl)-6-methoxy-3,5-dimethyl-4*H*-pyran-4-one (**4**). HRESIMS *m/z* 261.0120 $[M + H]^+$ (calcd. for $C_{10}H_{14}BrO_3$, 261.0121); **$^{1}$H NMR** (500 MHz, $CDCl_3$) $\delta_H$ 5.19 (q, $J$ = 6.9 Hz, 1H), 4.03 (s, 3H), 1.97 (overlap, 6H), 1.84 (s, 3H); **$^{13}$C NMR** (126 MHz, $CDCl_3$) $\delta_C$ 180.5, 162.0, 154.8, 118.5, 100.2, 55.7, 40.4, 22.7, 9.7, 7.1.

***Step D*:** A 50 mL round-bottom flask equipped with a magnetic stir bar and a reflux condenser was added compound **4** (1.0 g, 3.8 mmol, 1.0 eq.) and triethyl phosphite (10 mL), the reaction vessel was evacuated and back-filled with $N_2$ (three cycles), and the mixture was heated to 120 °C in an oil bath with stirring for 12 hours. After cooling to room temperature, the volatiles were removed under reduced pressure. The resulting crude residue was purified by flash chromatography (10% MeOH in DCM) to afford the phosphonate ester **5** (0.95 g, 78% yield) as a brown oil.

Diethyl (1-(6-methoxy-3,5-dimethyl-4-oxo-4*H*-pyran-2-yl)ethyl)phosphonate (**5**) *(28)*. **$^{1}$H NMR** (600 MHz, $CDCl_3$) $\delta_H$ 4.17 – 4.02 (m, 4H), 4.00 (s, 3H), 3.46 – 3.38 (m, 1H), 1.97 (d, $J$ = 3.3 Hz, 3H), 1.84 (s, 3H), 1.55 (dd, $J$ = 18.0, 7.2 Hz, 3H), 1.32 (t, $J$ = 7.1 Hz, 3H), 1.25 (t, $J$ = 7.1 Hz, 3H); **$^{13}$C NMR** (151 MHz, $CDCl_3$) $\delta_C$ 180.6, 162.3, 153.6 (d, $J$ = 11.4 Hz), 120.1 (d, $J$ = 8.8 Hz), 99.5, 62.9 (d, $J$ = 7.1 Hz), 62.5 (d, $J$ = 7.0 Hz), 55.7, 34.6 (d, $J$ = 140.8 Hz), 16.6 (d, $J$ = 5.8 Hz), 12.2 (d, $J$ = 4.9 Hz), 10.3, 7.0.

***Step E***: A 50 mL round-bottom flask equipped with a magnetic stir bar was added with compound **5** (0.6 g, 1.9 mmol, 1.0 eq.) in anhydrous DMF (15 mL), and the mixture was purged with $N_2$. After cooling to −78 °C, LHMDS (2.3 mL of a 1 M solution in THF, 2.3 mmol, 1.2 eq.) was added dropwise over 10 minutes. The resulting mixture was stirred at this temperature for 30 minutes, followed by the dropwise addition of aldehyde **6** (0.63 g, 3.8 mmol, 2.0 eq.) at −78 °C. The reaction mixture was then allowed to warm gradually to room temperature and stirred for an additional 2.5 hours. The reaction was quenched with saturated aqueous $NH_4Cl$ (20 mL) and extracted with EtOAc (2 × 20 mL). The combined organic layers were dried over anhydrous $Na_2SO_4$, filtered, and concentrated under reduced pressure to afford a brown oil. Purification by flash chromatography (20% EtOAc in PE) yielded the conjugated polyene **6** (0.45 g, 73% yield) as a yellow oil *(28)*.

***Step F***: A solution of compound **6** (0.4 g, 1.2 mmol, 1.0 eq.) in anhydrous MeOH (100 mL) in a 250 mL round-bottom flask was subjected to three $N_2$ purge cycles (evacuation/back-filling). The reaction mixture was then irradiated using a 250 W mercury lamp at room temperature for 12 hours. After completion, the solvent was removed under reduced pressure, and the crude residue was purified by flash chromatography (4% EtOAc in PE) to afford tridachiahydropyrone **2** (0.116 g, 29% yield, 48% brsm yield) as a white solid *(29, 34)*.

Tridachiahydropyrone (**2**) *(34)*. **$^1$H NMR** (400 MHz, $CDCl_3$) $\delta_H$ 5.51 (t, $J$ = 7.5 Hz, 1H), 5.44 (s, 1H), 3.96 (s, 3H), 3.91 (s, 1H), 1.89 (t, $J$ = 7.0 Hz, 2H), 1.75 (s, 3H), 1.66 (m, 1H), 1.64 (s, 3H), 1.63 (s, 3H), 1.52 (s, 3H), 1.16 (s, 3H), 0.89 (s, 3H), 0.87 (s, 3H); **$^{13}$C NMR** (151 MHz, $CDCl_3$) $\delta_C$ 196.0, 166.2, 145.4, 134.6, 133.5, 130.5, 121.4, 115.9, 88.0, 55.3, 53.6, 46.7, 37.4, 28.9, 22.8, 22.6, 21.9, 21.4, 14.8, 13.9, 7.6.

***Step G***: A 250 mL round-bottom flask equipped with a magnetic stir bar was added with isovaleraldehyde (5.0 g, 58.1 mmol, 1.0 eq.), (carbethoxyethylidene)triphenylphosphorane (21.0 g, 58.1 mmol, 1.0 eq.) in toluene (80 mL). The reaction mixture was heated to 80 °C in an oil bath and stirred for 12 hours. After completion, the solvent was removed under reduced pressure. The residue was treated with $Et_2O$ (30 mL), and the resulting precipitate of triphenylphosphine oxide was removed by filtration. The filtrate was concentrated under reduced pressure, and the crude product was purified by flash chromatography (3% EtOAc in PE) to afford ester **SI-4** (9.4 g, 95% yield) as a colorless oil *(34)*.

***Step H***: A 250 mL round-bottom flask equipped with a magnetic stir bar was added with compound **SI-4** (9.0 g, 52.9 mmol, 1.0 eq.) in $Et_2O$ (30 mL), and the mixture was purged with $N_2$. After cooling to 0 °C, DIBAL-H (105.8 mL of a 1 M solution in hexane, 105.8 mmol, 2.0 eq.) was added dropwise over 10 minutes. The reaction mixture was stirred at 0 °C for 6 hours and then carefully quenched by the addition of MeOH. After completion, the reaction was quenched by the cautious addition of MeOH. A saturated aqueous solution of Rochelle's salt was added, and the mixture was stirred vigorously for several hours until two clear layers separated. The layers were separated, and the aqueous phase was extracted with EtOAc (2 × 50 mL). The combined organic extracts were dried over anhydrous $Na_2SO_4$, filtered, and concentrated under reduced pressure. Purification of the crude product by flash chromatography (10% EtOAc in PE) afforded alcohol **SI-5** (6.4 g, 95% yield) as a colorless oil *(34)*.

***Step I***: A 250 mL round-bottom flask equipped with a magnetic stir bar was added with compound **SI-5** (6.0 g, 46.8 mmol, 1.0 eq.), $MnO_2$ (40.7 g, 468 mmol, 10.0 eq.) in DCM (120 mL). The reaction mixture was stirred for 48 hours at room temperature. After completion, the reaction mixture was then filtered through Celite, and the filtrate concentrated under reduced pressure, and the crude product was purified by flash chromatography (3% EtOAc in PE) to afford aldehyde **SI-6** (4.4 g, 75% yield) as a colorless oil *(34)*.

(*E*)-2,5-dimethylhex-2-enal (**SI-6**) *(34, 53)*. **$^{1}$H NMR** (600 MHz, $CDCl_3$) $\delta_H$ 9.40 (s, 1H), 6.51 (td, *J* = 7.5, 1.4 Hz, 1H), 2.27 – 2.19 (m, 2H), 1.82 (dt, *J* = 13.4, 6.7 Hz, 1H), 1.73 (s, 3H), 0.95 (d, *J* = 6.7 Hz, 6H). **$^{13}$C NMR** (151 MHz, $CDCl_3$) $\delta_C$ 195.5, 154.0, 140.1, 38.1, 28.4, 22.6, 9.4.

***Step J***: A 250 mL round-bottom flask equipped with a magnetic stir bar was added with **SI-6** (4.4 g, 34.9 mmol, 1.0 eq.), (carbethoxyethylidene)triphenylphosphorane (12.6 g, 34.9 mmol, 1.0 eq.) in toluene (50 mL). The reaction mixture was heated to 80 °C in an oil bath and stirred for 12 hours. After completion, the solvent was removed under reduced pressure. The residue was treated with $Et_2O$ (20 mL), and the resulting precipitate of triphenylphosphine oxide was removed by filtration. The filtrate was concentrated under reduced pressure, and the crude product was purified by flash chromatography (5% EtOAc in PE) to afford ester **SI-7** (6.7 g, 92% yield) as a colorless oil.

***Step K***: A 250 mL round-bottom flask equipped with a magnetic stir bar was added with compound **SI-7** (6.0 g, 28.5 mmol, 1.0 eq.) in $Et_2O$ (20 mL), and the mixture was purged with $N_2$. After cooling to 0 °C, DIBAL-H (57.0 mL of a 1 M solution in hexane, 57.0 mmol, 2.0 eq.) was added dropwise over 10 minutes. The reaction mixture was stirred at 0 °C for 6 hours and then carefully quenched by the addition of MeOH. After completion, the reaction was quenched by the cautious addition of MeOH. A saturated aqueous solution of Rochelle's salt was added, and the mixture was stirred vigorously for several hours until two clear layers separated. The layers were separated, and the aqueous phase was extracted with EtOAc (2 × 50 mL). The combined organic extracts were dried over anhydrous $Na_2SO_4$, filtered, and concentrated under reduced pressure. Purification of the crude product by flash chromatography (10% EtOAc in PE) afforded alcohol **SI-8** (4.6 g, 95% yield) as a colorless oil.

***Step I***: A 250 mL round-bottom flask equipped with a magnetic stir bar was added with compound **SI-8** (4.6 g, 27.1 mmol, 1.0 eq.), $MnO_2$ (23.6 g, 271 mmol, 10.0 eq.) in DCM (80 mL). The reaction mixture was stirred for 24 hours at room temperature. After completion, the reaction mixture was then filtered through Celite, and the filtrate concentrated under reduced pressure, and the crude product was purified by flash chromatography (5% EtOAc in PE) to afford aldehyde **6** (3.6 g, 80% yield) as a yellow oil.

(2*E*,4*E*)-2,4,7-trimethylocta-2,4-dienal (**6**). HRESIMS *m/z* 167.1424 $[M + H]^+$ (calcd. for $C_{11}H_{19}O$, 167.1430); **$^{1}$H NMR** (600 MHz, $CDCl_3$) $\delta_H$ 9.37 (s, 1H), 6.73 (s, 1H), 5.90 (t, *J* = 7.6 Hz, 1H), 2.08 (t, *J* = 7.2 Hz, 3H), 1.94 (overlap, 6H), 1.72 (hept, *J* = 6.7 Hz, 1H), 0.93 (d, *J* = 6.7 Hz, 6H); **$^{13}$C NMR** (151 MHz, $CDCl_3$) $\delta_C$ 196.3, 155.4, 140.5, 135.3, 133.7, 37.9, 28.8, 22.6, 16.2, 10.8.

## Part 2. Biomimetic Synthesis of Ocellatusone C (1)

**Table S1.** Optimization of the reaction conditions for biomimetic synthesis of ocellatusone C (**1**).

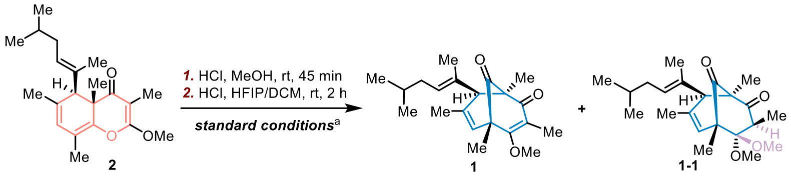


| entry | deviation from standard conditions | 1 (%)[b] | 1-1 (%)[b] |
|---|---|---|---|
| 1 | *1.* HCl (aq), $H_2O$ (1.0 eq), MeCN. Without **2.** | trace | 0% |
| 2 | *1.* HCl (aq), $H_2O$ (1.0 eq). Without **2.** | 46% | 10% |
| S1 | *1.* HCl (aq), $H_2O$ (1.0 eq), DCM. Without **2.** | trace | 0% |
| S2 | *1.* HCl (aq), $H_2O$ (1.0 eq), THF. Without **2.** | trace | 0% |
| S3 | *1.* HCl (aq), $H_2O$ (1.0 eq), dioxane. Without **2.** | trace | 0% |
| 3 | Without **2.** | 47% | 37% |
| 4 | *1.* 45°C. Without **2.** | 26% | 45% |
| 5 | *1.* 55°C. Without **2.** | 54% | 5% |
| 6 | *1.* then $Et_3N$ (0.5 eq) workup. Without **2.** | 0% | 84% |
| 7 | *1.* then $Et_3N$ (0.5 eq) workup | 85% | 0% |
| **8** | **none** | **85% 83%**[c] | 0% |
| 9 | Without *1.* | no reaction | |
| 10 | *1.* without acid | no reaction | |
| 11 | *1.* $CH_3COOH$, then $Et_3N$ (0.5 eq) workup | no reaction | |
| 12 | *1.* HBr·$CH_3COOH$, then $Et_3N$ (0.5 eq) workup | 84% | 0% |
| 13 | *1.* TsOH, then $Et_3N$ (0.5 eq) workup | 83% | 0% |
| 14 | *1.* $CF_3COOH$, then $Et_3N$ (0.5 eq) workup | 30% | 0% |
| 15 | *1.* $AlCl_3$, then $Et_3N$ (0.5 eq) workup | 84% | 0% |
| 16 | *1.* $BF_3$·$OEt_2$, then $Et_3N$ (0.5 eq) workup | 83% | 0% |

[a]Standard conditions: 1. **2** (5 mg, 1.0 eq.), HCl·MeOH (10 mol%) as an acid catalyst, MeOH (0.1 M), room temperature, 45 min. 2. HCl·EtOAc (5 mol%), HFIP/DCM (4:1, 0.1 M), room temperature, 2 h. [b]All yields are determined by $^1$H NMR (600 MHz, $CDCl_3$) with internal standard $CH_2Br_2$. [c]Isolated yields.

## General procedure:

<u>*Standard conditions:*</u>

***Step 1:*** To a solution of compound **2** (5 mg, 0.015 mmol, 1.0 eq.) and acid catalyst 4 M HCl·MeOH (0.38 µL, 10 mol%) in dry MeOH (151 µL, 0.1 M) at room temperature. The reaction was carried out at room temperature for 45 minutes. After completion, the solvent was removed under reduced pressure to give a crude mixture.

***Step 2:*** The above crude mixture was dissolved in HFIP/DCM (151 µL, 0.1 M, 4:1 v/v), followed by the addition of 4 M HCl·EtOAc (0.19 µL, 5 mol%). The reaction was allowed to proceed at room temperature for 2 hours. Upon completion, the solvent was removed under reduced pressure to afford the crude product. For isolated yield of ocellatusone C (**1**): the crude residue was purified by flash chromatography (3% EtOAc in PE) to afford ocellatusone C (**1**) (4.2 mg, 83% yield) as a colorless oil.

<u>*Deviation from standard conditions:*</u>

Entries 1-2 and S1-S3: ***Step 1:*** Using 12 M HCl (aq.) (10 mol%) instead 4 M HCl·MeOH, additionally, $H_2O$ (0.27 µL, 1.0 eq) was added. For entry 1, using MeCN solvent instead of MeOH; For entry S1, using DCM solvent instead of MeOH; For entry S2, using THF solvent instead of MeOH; For entry S3, using dioxane solvent instead of MeOH. Without performing ***Step 2***.

Entry 3: Without performing ***Step 2***.

Entries 4 and 5: ***Step 1:*** The reaction was performed at 45 °C for entry 4 and at 55 °C for entry 5, instead of at room temperature. Without performing ***Step 2***.

Entries 6 and 7: ***Step 1:*** After the reaction was complete, it was quenched with $Et_3N$ (0.77 mg, 0.5 eq.) followed by removal of the solvent under reduced pressure to afford a residue. The residue was were dissolved in water (1 mL) and EtOAc (1 mL), the layers were separated, and the aqueous phase was extracted with EtOAc (2 × 1 mL). The combined organic extracts were dried over anhydrous $Na_2SO_4$, filtered, and concentrated under reduced pressure to give a crude mixture. For entry 6: Without performing ***Step 2***.

Entry 9: Without performing ***Step 1***.

Entry 10: ***Step 1:*** without using acid catalyst.

Entries 11-16: ***Step 1:*** Using other acid catalyst (10 mol%) instead 4 M HCl·MeOH. For entry 11, using $CH_3COOH$ instead of HCl·MeOH; For entry 12, using HBr·$CH_3COOH$ instead of HCl·MeOH; For entry 13, using TsOH instead of HCl·MeOH; For entry 14, using $CF_3COOH$ instead of HCl·MeOH; For entry 15, using $AlCl_3$ instead of HCl·MeOH; For entry 11, using $BF_3$·$OEt_2$ instead of HCl·MeOH. After the reaction was complete, it was quenched with $Et_3N$ (0.77 mg, 0.5 eq.) followed by removal of the solvent under reduced pressure to afford a residue. The residue was were dissolved in water (1 mL) and EtOAc (1 mL), the layers were separated, and the aqueous phase was extracted with EtOAc (2 × 1 mL). The combined organic extracts were dried over anhydrous $Na_2SO_4$, filtered, and concentrated under reduced pressure to give a crude mixture.

Ocellatusone C (**1**): $^{1}$**H NMR** (600 MHz, $CDCl_3$) $\delta_H$ 5.65 (s, 1H), 5.36 (brs, 1H), 3.91 (s, 3H), 3.15 (s, 1H), 1.91 (m, 2H), 1.84 (s, 3H), 1.64 (hept, $J$ = 6.6 Hz, 1H), 1.49 (s, 3H), 1.32 (s, 3H), 1.25 (brs, 3H), 1.14 (s, 3H), 0.89 (d, $J$ = 6.7 Hz, 3H), 0.88 (d, $J$ = 6.3 Hz, 3H); $^{13}$**C NMR** (151 MHz, $CDCl_3$) $\delta_C$ 208.7, 202.5, 179.4, 135.5, 132.7, 131.6, 117.2, 65.4, 61.8, 60.2, 53.3, 37.4, 28.9, 22.6, 22.5, 21.5, 15.7, 15.7, 12.0, 10.4.

Intermediate **1-1**: $^{1}$**H NMR** (600 MHz, $CDCl_3$) $\delta_H$ 5.55 (s, 1H), 5.20 (s, 1H), 3.70 (q, $J$ = 6.7 Hz, 1H), 3.48 (s, 3H), 3.25 (s, 3H), 2.93 (s, 1H), 1.93 – 1.85 (m, 2H), 1.65 – 1.60 (m, 1H), 1.59 (s, 3H), 1.37 (s, 3H), 1.27 (s, 3H), 1.21 (d, $J$ = 6.8 Hz, 3H), 0.88 (t, $J$ = 6.7 Hz, 6H); $^{13}$**C NMR** (151 MHz, $CDCl_3$) $\delta_C$ 131.8, 103.2, 58.2, 53.0, 52.7, 49.1, 37.3, 28.9, 22.6, 22.5, 21.7, 15.8, 14.8, 9.8.

## Part 3. Mechanism Study of Biomimetic Synthesis

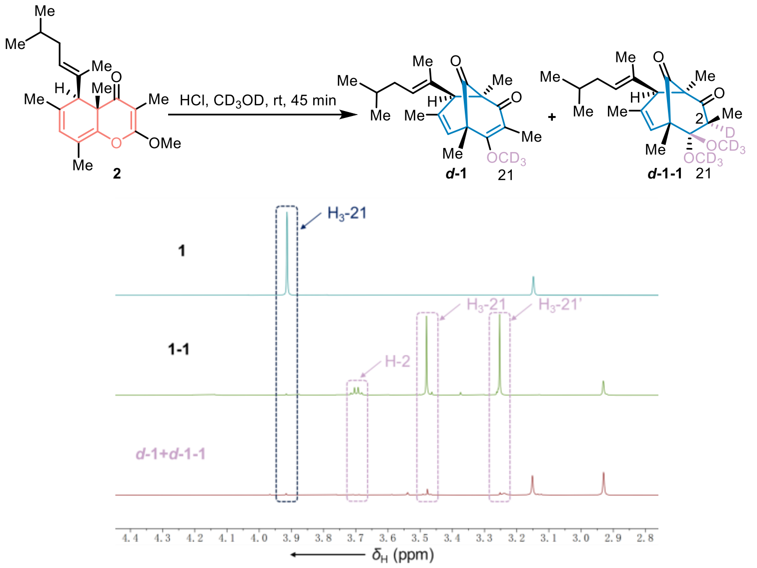


**Fig. S1.** Deuterium experiment of biomimetic synthesis of ocellatusone C (**1**).

To a solution of compound **2** (5 mg, 0.015 mmol, 1.0 eq.) and acid catalyst 4 M HCl·EtOAc (0.38 µL, 10 mol%) in $CD_3OD$ (151 µL, 0.1 M) at room temperature. The reaction was carried out at room temperature for 45 minutes. After completion, the solvent was removed under reduced pressure to give a crude mixture. The mixture was directly subjected to $^1H$ NMR analysis.

## Part 4. Synthesis of Analogues via Biomimetic Route

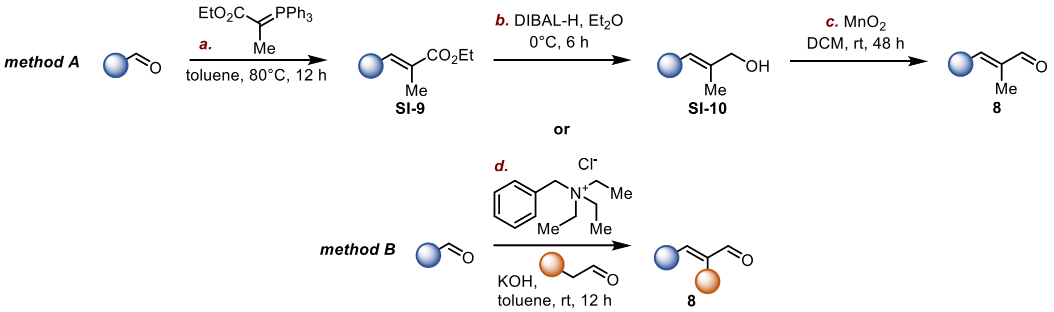


*Method A* (34)

***Step A:*** A 100 mL round-bottom flask equipped with a magnetic stir bar was added with various aldehydes (10.0 mmol, 1.0 eq.), (carbethoxyethylidene)triphenylphosphorane (10.0 mmol, 1.0 eq.) in toluene (20 mL). The reaction mixture was heated to 80 °C in an oil bath and stirred for 12 hours. After completion, the solvent was removed under reduced pressure. The residue was treated with $Et_2O$ (10 mL), and the resulting precipitate of triphenylphosphine oxide was removed by filtration. The filtrate was concentrated under reduced pressure, and the crude product was purified by flash chromatography (PE/EtOAc) to afford corresponding ester **SI-9**. The obtained ester **SI-9** was carried forward to the next step.

***Step B***: A 250 mL round-bottom flask equipped with a magnetic stir bar was added with compound **SI-9** (1.0 eq.) in $Et_2O$ (15 mL), and the mixture was purged with $N_2$. After cooling to 0 °C, DIBAL-H (a 1 M solution in hexane, 2.0 eq.) was added dropwise over 10 minutes. The reaction mixture was stirred at 0 °C for 6 hours and then carefully quenched by the addition of MeOH. After completion, the reaction was quenched by the cautious addition of MeOH. A saturated aqueous solution of Rochelle's salt was added, and the mixture was stirred vigorously for several hours until two clear layers separated. The layers were separated, and the aqueous phase was extracted with EtOAc (2 × 30 mL). The combined organic extracts were dried over anhydrous $Na_2SO_4$, filtered, and concentrated under reduced pressure. Purification of the crude product by flash chromatography (PE/EtOAc) afforded corresponding alcohol **SI-10**. The obtained alcohol **SI-10** was carried forward to the next step.

***Step I***: A 100 mL round-bottom flask equipped with a magnetic stir bar was added with compound **SI-10** (1.0 eq.), $MnO_2$ (10.0 eq.) in DCM (20 mL). The reaction mixture was stirred for 48 hours at room temperature. After completion, the reaction mixture was then filtered through Celite, and the filtrate concentrated under reduced pressure, and the crude product was purified by flash chromatography (PE/EtOAc) to afford corresponding aldehyde **8**.

*Method B (35)*
***Step D:*** A 100 mL round-bottom flask equipped with a magnetic stir bar was added with aryl aldehyde (10.0 mmol, 1.0 eq.), benzyl triethylammonium chloride (1.0 mmol, 0.1 eq.), KOH (1.5 mmol, 0.15 eq.) and aliphatic aldehyde (12.0 mmol, 1.2 eq.) in 15 mL of toluene. The reaction mixture was stirred at room temperature for 12 hours and then it was extracted with EtOAc (2 × 30 mL) and $H_2O$ (50 mL). The combined organic layers were dried over $Na_2SO_4$, filtered and concentrated under reduced pressure. The crude was purified by column chromatography (PE/EtOAc) to afford the corresponding aldehyde **8**.

(*E*)-3-(4-fluorophenyl)-2-methylacrylaldehyde (**8b**) *(54)*: synthesized via Method B, aldehyde **8b** (1.2 g, 75% yield) was obtained as a white solid. **$^1$H NMR** (600 MHz, $CDCl_3$) $\delta_H$ 9.56 (s, 1H), 7.54 – 7.50 (m, 2H), 7.22 (s, 1H), 7.13 (t, *J* = 8.6 Hz, 2H), 2.05 (d, *J* = 1.5 Hz, 3H); **$^{13}$C NMR** (151 MHz, $CDCl_3$) $\delta_C$ 195.5, 163.3 (d, *J* = 251.8 Hz), 148.6, 138.1 (d, *J* = 1.7 Hz), 132.1 (d, *J* = 8.6 Hz), 131.5 (d, *J* = 3.4 Hz), 116.0 (d, *J* = 21.8 Hz), 11.0.

(*E*)-3-(4-chlorophenyl)-2-methylacrylaldehyde (**8c**) *(54)*: synthesized via Method B, aldehyde **8c** (1.4 g, 78% yield) was obtained as a white solid. **$^1$H NMR** (600 MHz, $CDCl_3$) $\delta_H$ 9.57 (s, 1H), 7.47 – 7.39 (m, 4H), 7.20 (s, 1H), 2.05 (d, *J* = 1.6 Hz, 3H); **$^{13}$C NMR** (151 MHz, $CDCl_3$) $\delta_C$ 195.3, 148.2, 138.8, 135.7, 133.7, 131.3, 129.1, 11.0.

(*E*)-3-(4-bromophenyl)-2-methylacrylaldehyde (**8d**) *(55)*: synthesized via Method B, aldehyde **8d** (1.8 g, 80% yield) was obtained as a yellow solid. **$^1$H NMR** (600 MHz, $CDCl_3$) $\delta_H$ 9.58 (s, 1H), 7.57 (d, *J* = 8.5 Hz, 2H), 7.39 (d, *J* = 8.5 Hz, 2H), 7.19 (s, 1H), 2.05 (d, *J* = 1.5 Hz, 3H).

(*E*)-2-methyl-3-(4-(trifluoromethyl)phenyl)acrylaldehyde (**8e**) *(55)*: synthesized via Method B, aldehyde **8e** (1.8 g, 85% yield) was obtained as a yellow oil. **$^1$H NMR** (600 MHz, $CDCl_3$) $\delta_H$ 9.62 (s, 1H), 7.70 (d, *J* = 8.2 Hz, 2H), 7.61 (d, *J* = 8.1 Hz, 2H), 7.30 (s, 1H), 2.07 (d, *J* = 1.2 Hz, 3H).

($E$)-2-methyl-3-(4-nitrophenyl)acrylaldehyde (**8f**) *(56)*: synthesized via Method B, aldehyde **8f** (1.6 g, 85% yield) was obtained as a yellow solid. **$^1$H NMR** (600 MHz, $CDCl_3$) $\delta_H$ 9.65 (s, 1H), 8.31 (d, $J$ = 8.8 Hz, 2H), 7.66 (d, $J$ = 8.8 Hz, 2H), 7.32 (s, 1H), 2.09 (d, $J$ = 1.5 Hz, 3H); **$^{13}$C NMR** (151 MHz, $CDCl_3$) $\delta_C$ 194.8, 146.2, 141.5, 141.4, 130.6, 124.0, 11.2.

($E$)-3-(4-(tert-butyl)phenyl)-2-methylacrylaldehyde (**8g**): synthesized via Method B, aldehyde **8g** (1.4 g, 70% overall yield over three steps) was obtained as a white solid. HRESIMS *m/z* 203.1431 $[M + H]^+$ (calcd. for $C_{14}H_{19}O$, 203.1430); **$^1$H NMR** (600 MHz, $CDCl_3$) $\delta_H$ 9.58 (s, 1H), 7.53 – 7.46 (m, 4H), 7.24 (s, 1H), 2.10 (d, $J$ = 1.4 Hz, 3H), 1.35 (s, 9H); **$^{13}$C NMR** (151 MHz, $CDCl_3$) $\delta_C$ 195.8, 153.3, 150.0, 137.8, 132.6, 130.2, 125.9, 35.0, 31.3, 11.1.

($E$)-3-(3,5-dichlorophenyl)-2-methylacrylaldehyde (**8h**): synthesized via Method B, aldehyde **8h** (1.7 g, 78% yield) was obtained as a white solid. HRESIMS *m/z* 215.0025 $[M + H]^+$ (calcd. for $C_{10}H_9Cl_2O$, 215.0025); **$^1$H NMR** (600 MHz, $CDCl_3$) $\delta_H$ 9.58 (s, 1H), 7.40 – 7.34 (overlap, 3H), 7.13 (s, 1H), 2.05 (d, $J$ = 1.5 Hz, 3H); **$^{13}$C NMR** (151 MHz, $CDCl_3$) $\delta_C$ 194.9, 146.1, 140.7, 138.0, 135.5, 129.3, 128.0, 11.1.

($E$)-2-methyl-3-(3,4,5-trimethoxyphenyl)acrylaldehyde (**8i**) *(57)*: synthesized via Method A, aldehyde **8i** (1.7 g, 70% overall yield over three steps) was obtained as a white solid. **$^1$H NMR** (600 MHz, $CDCl_3$) $\delta_H$ 9.51 (s, 1H), 7.15 (s, 1H), 6.74 (s, 2H), 3.86 (overlap, 9H), 2.06 (d, $J$ = 1.6 Hz, 3H); **$^{13}$C NMR** (151 MHz, $CDCl_3$) $\delta_C$ 195.3, 153.2, 150.0, 139.4, 137.5, 130.6, 107.6, 60.9, 56.2, 11.0.

($E$)-3-(4-bromo-2,6-difluorophenyl)-2-methylacrylaldehyde (**8j**): synthesized via Method A, aldehyde **8j** (1.9 g, 72% overall yield over three steps) was obtained as a yellow solid. HRESIMS *m/z* 260.9712 $[M + H]^+$ (calcd. for $C_{10}H_8BrF_2O$, 260.9721); **$^1$H NMR** (600 MHz, $CDCl_3$) $\delta_H$ 9.66 (s, 1H), 7.21 – 7.17 (m, 2H), 7.07 (s, 1H), 1.80 (d, $J$ = 1.4 Hz, 3H); **$^{13}$C NMR** (151 MHz, $CDCl_3$) $\delta_C$ 194.2, 159.7 (dd, $J$ = 255.5, 8.2 Hz), 144.4, 134.8, 123.4 (t, $J$ = 12.5 Hz), 117.1 – 114.8 (m), 112.1 (t, $J$ = 19.3 Hz), 11.9; **$^{19}$F NMR** (565 MHz, $CDCl_3$) $\delta_F$ -106.87 (d, $J$ = 8.0 Hz).

($E$)-3-(3,5-dibromo-4-methoxyphenyl)-2-methylacrylaldehyde (**8k**): synthesized via Method B, aldehyde **8k** (2.6 g, 78% yield) was obtained as a yellow solid. HRESIMS *m/z* 332.3120 $[M + H]^+$ (calcd. for $C_{11}H_{11}Br_2O_2$, 332.3120); **$^1$H NMR** (600 MHz, $CDCl_3$) $\delta_H$ 9.55 (s, 1H), 7.66 (s, 2H), 7.08 (s, 1H), 3.93 (s, 3H), 2.06 (s, 3H); **$^{13}$C NMR** (151 MHz, $CDCl_3$) $\delta_C$ 194.9, 155.0, 145.8, 139.8, 134.0, 133.8, 118.7, 60.9, 11.0.

($E$)-2-methyl-3-(*o*-tolyl)acrylaldehyde (**8l**) *(54)*: synthesized via Method B, aldehyde **8l** (1.2 g, 74% yield) was obtained as a white solid. **$^1$H NMR** (600 MHz, $CDCl_3$) $\delta_H$ 9.65 (s, 1H), 7.44 (s, 1H), 7.34 (d, $J$ = 7.6 Hz, 1H), 7.27 (overlap, 3H), 2.35 (s, 3H), 1.94 (d, $J$ = 1.4 Hz, 3H); **$^{13}$C NMR** (151 MHz, $CDCl_3$) $\delta_C$ 195.6, 148.5, 139.4, 137.1, 134.0, 130.5, 129.3, 128.9, 125.8, 20.0, 10.9.

($E$)-2-(4-bromobenzylidene)butanal (**8m**) *(58)*: synthesized via Method B, aldehyde **8m** (1.9 g, 80% yield) was obtained as a yellow oil. **$^1$H NMR** (600 MHz, $CDCl_3$) $\delta_H$ 9.54 (s, 1H), 7.58 (d, $J$ = 8.5 Hz, 2H), 7.36 (d, $J$ = 8.5 Hz, 2H), 7.13 (s, 1H), 2.53 (q, $J$ = 7.5 Hz, 2H), 1.13 (t, $J$ = 7.5 Hz, 3H); **$^{13}$C NMR** (151 MHz, CDCl3) $\delta_C$ 195.4, 148.1, 145.1, 133.9, 132.2, 131.2, 124.0, 18.2, 12.9.

($E$)-2-(4-bromobenzylidene)-5-chloropentanal (**8n**): synthesized via Method B, aldehyde **8n** (2.2 g, 78% yield) was obtained as a yellow solid. HRESIMS *m/z* 286.9826 $[M + H]^+$ (calcd. for $C_{12}H_{13}BrClO$, 286.9832); **$^1$H NMR** (600 MHz, $CDCl_3$) $\delta_H$ 9.55 (s, 1H), 7.59 (d, $J$ = 8.5 Hz, 2H), 7.40 (d, $J$ = 8.5 Hz, 2H), 7.21 (s, 1H), 3.59 (t, $J$ = 6.3 Hz, 2H), 2.71 – 2.65 (m, 2H), 1.95 (dt, $J$ = 14.3, 6.3 Hz, 2H); **$^{13}$C NMR** (151 MHz, $CDCl_3$) $\delta_C$ 195.3, 149.3, 142.1, 133.5, 132.3, 131.3, 124.5, 45.2, 30.9, 22.6.

($E$)-2-(4-(trifluoromethyl)benzylidene)butanal (**8o**): synthesized via Method B, aldehyde **8o** (1.9 g, 82% yield) was obtained as a yellow oil. HRESIMS *m/z* 229.0846 $[M + H]^+$ (calcd. for $C_{12}H_{12}F_3O$, 229.0834); **$^1$H NMR** (600 MHz, $CDCl_3$) $\delta_H$ 9.59 (s, 1H), 7.70 (d, $J$ = 8.1 Hz, 2H), 7.59 (d, $J$ = 8.1 Hz, 2H), 7.24 (s, 1H), 2.53 (q, $J$ = 7.5 Hz, 2H), 1.14 (t, $J$ = 7.6 Hz, 3H); **$^{13}$C NMR** (151 MHz, $CDCl_3$) $\delta_C$ 195.2, 147.3, 146.4, 138.5, 131.2 (d, $J$ = 33.0 Hz), 129.7, 125.9 (q, $J$ = 3.8 Hz), 124.0 (d, $J$ = 272.1 Hz), 18.3, 13.1; **$^{19}$F NMR** (565 MHz, $CDCl_3$) $\delta_F$ -62.9.

($E$)-2-(4-(trifluoromethyl)benzylidene)pentanal (**8p**): synthesized via Method B, aldehyde **8p** (1.9 g, 79% yield) was obtained as a yellow oil. HRESIMS *m/z* 243.0985 $[M + H]^+$ (calcd. for $C_{13}H_{14}F_3O$, 243.0991); **$^1$H NMR** (600 MHz, $CDCl_3$) $\delta_H$ 9.58 (s, 1H), 7.70 (d, $J$ = 8.1 Hz, 2H), 7.57 (d, $J$ = 8.1 Hz, 2H), 7.25 (s, 1H), 2.51 – 2.45 (m, 2H), 1.52 (dq, $J$ = 15.1, 7.5 Hz, 2H), 0.96 (t, $J$ = 7.4 Hz, 3H); **$^{13}$C NMR** (151 MHz, $CDCl_3$) $\delta_C$ 195.4, 147.7, 145.1, 138.6, 131.2 (q, $J$ = 32.7 Hz), 129.7, 125.9 (q, $J$ = 3.8 Hz), 124.0 (d, $J$ = 272.4 Hz), 26.9, 21.9, 14.4. **$^{19}$F NMR** (565 MHz, $CDCl_3$) $\delta_F$ -62.9.

($E$)-2-(4-(trifluoromethyl)benzylidene)hexanal (**8q**): synthesized via Method B, aldehyde **8q** (2.2 g, 85% yield) was obtained as a yellow oil. HRESIMS *m/z* 257.1147 $[M + H]^+$ (calcd. for $C_{14}H_{16}F_3O$, 257.1147); **$^1$H NMR** (600 MHz, $CDCl_3$) $\delta_H$ 9.58 (s, 1H), 7.70 (d, $J$ = 8.2 Hz, 2H), 7.58 (d, $J$ = 8.1 Hz, 2H), 7.24 (s, 1H), 2.53 – 2.47 (m, 2H), 1.50 – 1.42 (m, 2H), 1.38 (dq, $J$ = 14.6, 7.3 Hz, 2H), 0.91 (t, $J$ = 7.3 Hz, 3H); **$^{13}$C NMR** (151 MHz, $CDCl_3$) $\delta_C$ 195.3, 147.5, 145.3, 138.6 (d, $J$ = 1.5 Hz), 131.1 (q, $J$ = 32.8 Hz), 129.7, 127.1 – 120.8 (m), 125.8 (q, $J$ = 3.6 Hz), 30.6, 24.7, 23.1, 13.9; **$^{19}$F NMR** (565 MHz, $CDCl_3$) $\delta_F$ -62.9.

($E$)-3-methyl-2-(4-(trifluoromethyl)benzylidene)butanal (**8r**): synthesized via Method B, aldehyde **8r** (1.8 g, 75% yield) was obtained as a yellow oil. HRESIMS *m/z* 243.0991 $[M + H]^+$ (calcd. for $C_{13}H_{14}F_3O$, 243.0991); **$^1$H NMR** (600 MHz, $CDCl_3$) $\delta_H$ 9.56 (d, $J$ = 2.1 Hz, 1H), 7.68 (d, $J$ = 8.1 Hz, 2H), 7.46 (d, $J$ = 8.0 Hz, 2H), 7.23 (s, 1H), 3.05 (pd, $J$ = 7.0, 2.2 Hz, 1H), 1.26 (d, $J$ = 7.1 Hz, 6H); **$^{13}$C NMR** (151 MHz, $CDCl_3$) $\delta_C$ 195.3, 149.7, 147.4, 138.9, 130.9 (q, $J$ = 32.7 Hz), 129.2, 125.7 (q, $J$ = 3.8 Hz), 124.0 (q, $J$ = 272.2 Hz), 27.2, 20.5; **$^{19}$F NMR** (565 MHz, $CDCl_3$) $\delta_F$ -62.8.

(2$E$,4$E$)-2,4-dimethylhexa-2,4-dienal (**8s**) *(59)*: synthesized via Method A, aldehyde **8s** (0.76 g, 61% overall yield over three steps) was obtained as a colorless oil. **$^1$H NMR** (600 MHz, $CDCl_3$) $\delta_H$ 9.35 (s, 1H), 6.71 (s, 1H), 5.97 (q, $J$ = 7.1 Hz, 1H), 1.95 (s, 3H), 1.93 (s, 3H), 1.79 (d, $J$ = 7.2 Hz, 3H); **$^{13}$C NMR** (151 MHz, $CDCl_3$) $\delta_C$ 196.3, 155.2, 135.7, 135.1, 134.2, 15.7, 14.5, 10.7.

($E$)-3-(cyclopent-1-en-1-yl)-2-methylacrylaldehyde (**8t**): synthesized via Method A, aldehyde **8t** (0.95 g, 70% overall yield over three steps) was obtained as a colorless oil. HRESIMS *m/z* 137.0967 $[M + H]^+$ (calcd. for $C_9H_{13}O$, 137.0960); **$^1$H NMR** (600 MHz, $CDCl_3$) $\delta_H$ 9.40 (s, 1H),

6.92 (s, 1H), 6.24 (s, 1H), 2.75 – 2.68 (m, 2H), 2.46 (s, 2H), 1.99 (p, $J$ = 7.6 Hz, 2H), 1.92 (s, 3H); **$^{13}$C NMR** (151 MHz, $CDCl_3$) $\delta_C$ 196.0, 146.6, 142.5, 141.6, 136.3, 34.2, 32.9, 24.1, 10.4.

($E$)-3-methyl-2-(4-nitrobenzylidene)butanal (**8v**) *(60)*: synthesized via Method B, aldehyde **8v** (1.9 g, 85% yield) was obtained as a yellow oil. **$^1$H NMR** (600 MHz, $CDCl_3$) $\delta_H$ 9.58 (d, $J$ = 2.1 Hz, 1H), 8.28 (d, $J$ = 8.7 Hz, 1H), 7.51 (d, $J$ = 8.7 Hz, 2H), 7.24 (s, 1H), 3.02 (heptd, $J$ = 7.0, 2.2 Hz, 1H), 1.27 (d, $J$ = 7.0 Hz, 6H).

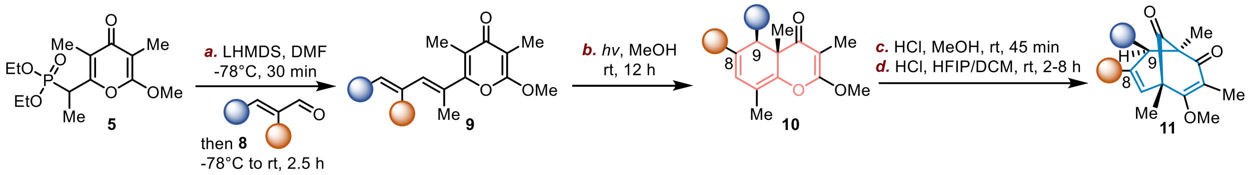


***Step A***: A 50 mL round-bottom flask equipped with a magnetic stir bar was added with compound **5** (0.2 g, 0.63 mmol, 1.0 eq.) in anhydrous DMF (10 mL), and the mixture was purged with $N_2$. After cooling to −78 °C, LHMDS (1.3 mL of a 1 M solution in THF, 1.3 mmol, 2.0 eq.) was added dropwise over 10 minutes. The resulting mixture was stirred at this temperature for 30 minutes, followed by the dropwise addition of corresponding aldehyde **8** (1.3 mmol, 2.0 eq.) at −78 °C. The reaction mixture was then allowed to warm gradually to room temperature and stirred for an additional 2.5 hours. The reaction was quenched with saturated aqueous $NH_4Cl$ (15 mL) and extracted with EtOAc (2 × 25 mL). The combined organic layers were dried over anhydrous $Na_2SO_4$, filtered, and concentrated under reduced pressure to afford a brown oil. Purification by flash chromatography (PE/EtOAc) yielded the corresponding conjugated polyene **9**. The conjugated polyene **9** was not sufficiently stable and was immediately carried forward to the next step.

***Step B***: A solution of compound **9** (1.0 eq.) in anhydrous MeOH (60 mL) in a 250 mL round-bottom flask was subjected to three $N_2$ purge cycles (evacuation/back-filling). The reaction mixture was then irradiated using a 250 W mercury lamp at room temperature for 12 hours. After completion, the solvent was removed under reduced pressure, and the crude residue was purified by flash chromatography (PE/EtOAc) to afford tridachiahydropyrone analogues **10**.

Tridachiahydropyrone analogue **10a**: synthesized from 2-methoxy-3,5-dimethyl-6-((2$E$,4$E$)-4-methyl-5-phenylpenta-2,4-dien-2-yl)-4$H$-pyran-4-one (**9a**). **9a** was synthesized via Step A, affording 0.146 g (75% yield) as a colorless oil. **10a** was synthesized via Step B, affording 0.047 g (32% yield) as a white solid. HRESIMS $m/z$ 311.1642 $[M + H]^+$ (calcd. for $C_{20}H_{23}O_3$, 311.1642); **$^1$H NMR** (400 MHz, $CDCl_3$) $\delta_H$ 7.51 (d, $J$ = 7.2 Hz, 1H), 7.29 (m, 1H), 7.24 – 7.14 (overlap, 3H), 5.58 – 5.52 (m, 1H), 4.46 (s, 1H), 3.97 (s, 3H), 1.83 (s, 3H), 1.65 (s, 3H), 1.49 (t, $J$ = 1.4 Hz, 3H), 0.93 (s, 3H); **$^{13}$C NMR** (101 MHz, $CDCl_3$) $\delta_C$ 195.9, 165.3, 144.4, 140.5, 135.0, 132.4, 129.6, 127.9, 127.5, 126.4, 121.6, 114.7, 87.9, 55.1, 50.7, 47.2, 23.6, 22.4, 14.5, 7.2.

Tridachiahydropyrone analogue **10b**: synthesized from 2-((2$E$,4$E$)-5-(4-fluorophenyl)-4-methylpenta-2,4-dien-2-yl)-6-methoxy-3,5-dimethyl-4$H$-pyran-4-one (**9b**). **9b** was synthesized via Step A, affording 0.142 g (69% yield) as a colorless oil. **10b** was synthesized via Step B, affording 0.043 g (30% yield) as a white solid. HRESIMS $m/z$ 329.1551 $[M + H]^+$ (calcd. for $C_{20}H_{22}FO_3$, 329.1548); **$^1$H NMR** (600 MHz, $CDCl_3$) $\delta_H$ 7.51 (t, $J$ = 6.4 Hz, 1H), 7.11 (s, 1H), 7.00 – 6.91 (m, 2H), 5.55 (s, 1H), 4.44 (s, 1H), 3.97 (s, 3H), 1.83 (s, 3H), 1.64 (s, 3H), 1.48 (s, 3H),

0.94 (s, 3H); **$^{13}$C NMR** (151 MHz, $CDCl_3$) $\delta_C$ 195.9, 165.3, 161.7 (d, $J$ = 244.6 Hz), 144.5, 136.2 (d, $J$ = 3.4 Hz), 134.6, 134.1 (d, $J$ = 7.6 Hz), 131.2 (d, $J$ = 7.9 Hz), 122.1, 114.6, 114.5, 88.1, 55.2, 50.3, 47.3, 23.7, 22.4, 14.6, 7.3; **$^{19}$F NMR** (565 MHz, $CDCl_3$) $\delta_F$ -116.7 (dt, $J$ = 9.6, 5.3 Hz).

Tridachiahydropyrone analogue **10c**: synthesized from 2-((2*E*,4*E*)-5-(4-chlorophenyl)-4-methylpenta-2,4-dien-2-yl)-6-methoxy-3,5-dimethyl-4*H*-pyran-4-one (**9c**). **9c** was synthesized via Step A, affording 0.156 g (72% yield) as a yellow oil. **10c** was synthesized via Step B, affording 0.055 g (35% yield) as a white solid. HRESIMS *m/z* 345.1264 $[M + H]^+$ (calcd. for $C_{20}H_{22}ClO_3$, 345.1252); **$^1$H NMR** (600 MHz, $CDCl_3$) $\delta_H$ 7.49 (d, $J$ = 7.9 Hz, 1H), 7.26 (overlap, 1H), 7.23 (d, $J$ = 8.4 Hz, 1H), 7.09 (d, $J$ = 7.2 Hz, 1H), 5.56 (s, 1H), 4.43 (s, 1H), 3.97 (s, 3H), 1.82 (s, 3H), 1.64 (s, 3H), 1.47 (s, 3H), 0.94 (s, 3H); **$^{13}$C NMR** (151 MHz, $CDCl_3$) $\delta_C$ 195.8, 165.3, 144.4, 139.1, 134.2, 134.0, 132.3, 131.2, 128.1, 127.8, 122.3, 114.6, 88.1, 55.2, 50.5, 47.2, 23.7, 22.4, 14.6, 7.3.

Tridachiahydropyrone analogue **10d**: synthesized from 2-((2*E*,4*E*)-5-(4-bromophenyl)-4-methylpenta-2,4-dien-2-yl)-6-methoxy-3,5-dimethyl-4*H*-pyran-4-one (**9d**). **9d** was synthesized via Step A, affording 0.191 g (78% yield) as a yellow oil. **10d** was synthesized via Step B, affording 0.061 g (32% yield, 58% brsm yield) as a white solid. HRESIMS *m/z* 389.0747 $[M + H]^+$ (calcd. for $C_{20}H_{22}BrO_3$, 389.0747); **$^1$H NMR** (600 MHz, $CDCl_3$) $\delta_H$ 7.44 – 7.40 (m, 2H), 7.37 (d, $J$ = 8.3 Hz, 1H), 7.03 (d, $J$ = 7.9 Hz, 1H), 5.55 (s, 1H), 4.41 (s, 1H), 3.96 (s, 3H), 1.82 (s, 3H), 1.63 (s, 3H), 1.46 (s, 3H), 0.94 (s, 3H); **$^{13}$C NMR** (151 MHz, $CDCl_3$) $\delta_C$ 195.7, 165.3, 144.4, 139.6, 134.4, 134.0, 131.6, 131.1, 130.7, 122.4, 120.5, 114.6, 88.0, 55.2, 50.6, 47.1, 23.7, 22.4, 14.6, 7.3.

Tridachiahydropyrone analogue **10e**: synthesized from 2-methoxy-3,5-dimethyl-6-((2*E*,4*E*)-4-methyl-5-(4-(trifluoromethyl)phenyl)penta-2,4-dien-2-yl)-4*H*-pyran-4-one (**9e**). **9e** was synthesized via Step A, affording 0.155 g (65% yield) as a yellow oil. **10e** was synthesized via Step B, affording 0.046 g (30% yield) as a yellow oil. HRESIMS *m/z* 379.1512 $[M + H]^+$ (calcd. for $C_{21}H_{22}F_3O_3$, 379.1516); **$^1$H NMR** (600 MHz, $CDCl_3$) $\delta_H$ 7.69 (d, $J$ = 7.5 Hz, 1H), 7.55 (d, $J$ = 7.8 Hz, 1H), 7.51 (d, $J$ = 8.0 Hz, 1H), 7.28 (d, $J$ = 7.7 Hz, 1H), 5.60 (s, 1H), 4.51 (s, 1H), 3.98 (s, 3H), 1.84 (s, 3H), 1.64 (s, 3H), 1.46 (s, 3H), 0.96 (s, 3H); **$^{13}$C NMR** (151 MHz, $CDCl_3$) $\delta_C$ 195.6, 165.3, 144.7, 144.4, 133.7, 133.1, 130.3, 128.9, 128.7, 125.4, 124.7 (d, $J$ = 82.9 Hz), 122.7, 114.6, 88.1, 55.3, 51.1, 47.2, 23.7, 22.4, 14.6, 7.3; **$^{19}$F NMR** (565 MHz, $CDCl_3$) $\delta_F$ -62.6.

Tridachiahydropyrone analogue **10f**: synthesized from 2-methoxy-3,5-dimethyl-6-((2*E*,4*E*)-4-methyl-5-(4-nitrophenyl)penta-2,4-dien-2-yl)-4*H*-pyran-4-one (**9f**). **9f** was synthesized via Step A, affording 0.150 g (67% yield) as a yellow oil. **10f** was synthesized via Step B, affording 0.046 g (31% yield) as a yellow solid. HRESIMS *m/z* 356.1494 $[M + H]^+$ (calcd. for $C_{20}H_{22}NO_5$, 356.1493); **$^1$H NMR** (600 MHz, $CDCl_3$) $\delta_H$ 8.18 – 8.10 (m, 2H), 7.76 (d, $J$ = 8.4 Hz, 1H), 7.33 (d, $J$ = 8.1 Hz, 1H), 5.63 (t, $J$ = 1.9 Hz, 1H), 4.53 (s, 1H), 3.98 (s, 3H), 1.84 (s, 3H), 1.63 (s, 3H), 1.45 (s, 3H), 0.99 (s, 3H); **$^{13}$C NMR** (151 MHz, $CDCl_3$) $\delta_C$ 195.2, 165.2, 148.5, 146.7, 144.3, 133.9, 132.6, 130.8, 123.4, 123.2, 122.6, 114.6, 88.1, 55.3, 51.4, 47.2, 23.5, 22.3, 14.5, 7.2.

Tridachiahydropyrone analogue **10g**: synthesized from 2-((2*E*,4*E*)-5-(4-(tert-butyl)phenyl)-4-methylpenta-2,4-dien-2-yl)-6-methoxy-3,5-dimethyl-4*H*-pyran-4-one (**9g**). **9g** was synthesized via Step A, affording 0.161 g (70% yield) as a colorless oil. **10g** was synthesized via Step B, affording 0.056 g (35% yield) as a white solid. HRESIMS *m/z* 367.2257 $[M + H]^+$ (calcd. for $C_{24}H_{31}O_3$, 367.2268); **$^1$H NMR** (600 MHz, $CDCl_3$) $\delta_H$ 7.42 (d, $J$ = 7.9 Hz, 1H), 7.26 (t, $J$ = 6.9 Hz,

2H), 7.07 (d, $J$ = 8.3 Hz, 1H), 5.53 (s, 1H), 4.41 (s, 1H), 3.96 (s, 3H), 1.82 (s, 3H), 1.65 (s, 3H), 1.49 (s, 3H), 1.30 (s, 9H), 0.94 (s, 3H); **$^{13}$C NMR** (151 MHz, $CDCl_3$) $\delta_C$ 196.1, 165.3, 149.1, 144.5, 137.2, 135.4, 131.9, 129.2, 124.5, 124.5, 121.4, 114.7, 88.0, 55.0, 50.3, 47.4, 34.4, 31.4, 23.5, 22.4, 14.5, 7.2.

Tridachiahydropyrone analogue **10h**: synthesized from 2-((2*E*,4*E*)-5-(3,5-dichlorophenyl)-4-methylpenta-2,4-dien-2-yl)-6-methoxy-3,5-dimethyl-4*H*-pyran-4-one (**9h**). **9h** was synthesized via Step A, affording 0.191 g (80% yield) as a yellow oil. **10h** was synthesized via Step B, affording 0.061 g (32% yield) as a white solid. HRESIMS *m/z* 379.0852 $[M + H]^+$ (calcd. for $C_{20}H_{21}Cl_2O_3$, 379.0862); **$^1$H NMR** (600 MHz, $CDCl_3$) $\delta_H$ 7.48 (s, 1H), 7.22 (t, $J$ = 1.9 Hz, 1H), 7.03 (s, 1H), 5.58 (s, 1H), 4.43 (s, 1H), 3.98 (s, 3H), 1.83 (s, 3H), 1.65 (s, 3H), 1.49 (s, 3H), 0.97 (s, 3H); **$^{13}$C NMR** (151 MHz, $CDCl_3$) $\delta_C$ 195.3, 165.4, 144.3, 144.2, 134.5, 134.2, 133.1, 130.9, 128.3, 126.9, 122.9, 114.7, 88.0, 55.3, 50.7, 47.2, 23.9, 22.4, 14.6, 7.3.

Tridachiahydropyrone analogue **10i**: synthesized from 2-methoxy-3,5-dimethyl-6-((2*E*,4*E*)-4-methyl-5-(3,4,5-trimethoxyphenyl)penta-2,4-dien-2-yl)-4*H*-pyran-4-one (**9i**). **9i** was synthesized via Step A, affording 0.151 g (60% yield) as a yellow oil. **10i** was synthesized via Step B, affording 0.044 g (29% yield) as a white solid. HRESIMS *m/z* 401.1960 $[M + H]^+$ (calcd. for $C_{23}H_{29}O_6$, 401.1959); **$^1$H NMR** (600 MHz, $CDCl_3$) $\delta_H$ 6.80 (s, 1H), 6.36 (s, 1H), 5.54 (t, $J$ = 1.6 Hz, 1H), 4.45 (s, 1H), 3.98 (s, 3H), 3.89 (s, 3H), 3.83 (s, 3H), 3.80 (s, 3H), 1.83 (s, 3H), 1.66 (s, 3H), 1.54 (s, 3H), 0.96 (s, 3H); **$^{13}$C NMR** (151 MHz, $CDCl_3$) $\delta_C$ 196.1, 165.6, 152.9, 152.5, 144.4, 136.7, 136.3, 135.0, 121.6, 114.7, 109.7, 106.6, 88.1, 61.0, 56.4, 56.3, 55.3, 50.8, 47.5, 23.9, 22.5, 14.7, 7.5.

Tridachiahydropyrone analogue **10j**: synthesized from 2-((2*E*,4*E*)-5-(4-bromo-2,6-difluorophenyl)-4-methylpenta-2,4-dien-2-yl)-6-methoxy-3,5-dimethyl-4*H*-pyran-4-one (**9j**). **9j** was synthesized via Step A, affording 0.166 g (62% yield) as a yellow oil. **10j** was synthesized via Step B, affording 0.055 g (33% yield) as a yellow oil. HRESIMS *m/z* 425.05494 $[M + H]^+$ (calcd. for $C_{20}H_{20}BrF_2O_3$, 425.05584); **$^1$H NMR** (600 MHz, $CDCl_3$) $\delta_H$ 7.12 (dt, $J$ = 8.8, 1.7 Hz, 1H), 7.03 (dt, $J$ = 9.9, 1.9 Hz, 1H), 5.62 (dd, $J$ = 3.0, 1.6 Hz, 1H), 4.60 (s, 1H), 3.98 (s, 3H), 1.83 (s, 3H), 1.61 (s, 3H), 1.52 (s, 3H), 1.23 (d, $J$ = 5.5 Hz, 3H); **$^{13}$C NMR** (151 MHz, $CDCl_3$) $\delta_C$ 194.1, 164.9, 145.2, 131.5, 122.7, 120.3, 115.5, 115.3 (d, $J$ = 3.4 Hz), 115.2 (d, $J$ = 3.8 Hz), 114.5, 87.8, 55.1, 47.6, 41.6, 21.6 (d, $J$ = 2.4 Hz), 20.8 (d, $J$ = 10.6 Hz), 14.4, 7.0; **$^{19}$F NMR** (565 MHz, $CDCl_3$) $\delta_F$ -104.5 – -104.7 (m), -106.8 (d, $J$ = 8.0 Hz).

Tridachiahydropyrone analogue **10k**: synthesized from 2-((2*E*,4*E*)-5-(3,5-dibromo-4-methoxyphenyl)-4-methylpenta-2,4-dien-2-yl)-6-methoxy-3,5-dimethyl-4*H*-pyran-4-one (**9k**). **9k** was synthesized via Step A, affording 0.235 g (75% yield) as a yellow oil. **10k** was synthesized via Step B, affording 0.070 g (30% yield) as a white solid. HRESIMS *m/z* 496.9965 $[M + H]^+$ (calcd. for $C_{21}H_{23}Br_2O_4$, 496.9958); **$^1$H NMR** (600 MHz, $CDCl_3$) $\delta_H$ 7.73 (s, 1H), 7.26 (s, 1H), 5.57 (s, 1H), 4.37 (s, 1H), 3.98 (s, 3H), 3.88 (s, 3H), 1.82 (s, 3H), 1.65 (s, 3H), 1.49 (s, 3H), 0.98 (s, 3H); **$^{13}$C NMR** (151 MHz, $CDCl_3$) $\delta_C$ 195.4, 165.4, 152.7, 144.3, 139.5, 136.5, 133.8, 133.2, 122.9, 117.4, 117.3, 114.6, 88.1, 60.8, 55.3, 50.1, 47.2, 23.9, 22.4, 14.6, 7.3.

Tridachiahydropyrone analogue **10l**: synthesized from 2-methoxy-3,5-dimethyl-6-((2*E*,4*E*)-4-methyl-5-(o-tolyl)penta-2,4-dien-2-yl)-4*H*-pyran-4-one (**9l**). **9l** was synthesized via Step A,

affording 0.143 g (70% yield) as a colorless oil. **10l** was synthesized via Step B, affording 0.047 g (33% yield) as a white solid. HRESIMS *m/z* 325.1795 $[M + H]^+$ (calcd. for $C_{21}H_{25}O_3$, 325.1798); $^{1}$**H NMR** (600 MHz, $CDCl_3$) $\delta_H$ 7.21 – 7.18 (m, 1H), 7.15 – 7.13 (m, 1H), 7.12 – 7.08 (m, 2H), 5.53 (s, 1H), 4.85 (s, 1H), 3.98 (s, 3H), 2.49 (s, 3H), 1.85 (s, 3H), 1.66 (s, 3H), 1.48 (s, 3H), 0.86 (s, 3H); $^{13}$**C NMR** (151 MHz, $CDCl_3$) $\delta_C$ 196.0, 166.5, 144.7, 139.5, 137.7, 136.8, 130.3, 129.4, 126.5, 125.9, 120.8, 115.8, 88.2, 55.3, 47.8, 44.4, 22.9, 22.2, 20.6, 14.9, 7.5.

Tridachiahydropyrone analogue **10m**: synthesized from 2-((*E*)-4-((*E*)-4-bromobenzylidene)hex-2-en-2-yl)-6-methoxy-3,5-dimethyl-4*H*-pyran-4-one (**9m**). **9m** was synthesized via Step A, affording 0.180 g (71% yield) as a yellow oil. **10m** was synthesized via Step B, affording 0.056 g (31% yield) as a yellow oil. HRESIMS *m/z* 403.0909 $[M + H]^+$ (calcd. for $C_{21}H_{24}BrO_3$, 403.0903); $^{1}$**H NMR** (600 MHz, $CDCl_3$) $\delta_H$ 7.41 (s, 2H), 7.37 (d, $J$ = 8.5 Hz, 1H), 7.04 (d, $J$ = 8.3 Hz, 1H), 5.55 (s, 1H), 4.51 (s, 1H), 3.97 (s, 3H), 1.85 (s, 3H), 1.83 – 1.79 (m, 1H), 1.72 (dq, $J$ = 15.1, 7.0 Hz, 1H), 1.64 (s, 3H), 0.92 (s, 3H), 0.88 (t, $J$ = 7.4 Hz, 3H); $^{13}$**C NMR** (151 MHz, $CDCl_3$) $\delta_C$ 195.7, 165.5, 144.5, 140.0, 139.7, 134.3, 131.6, 131.1, 130.8, 120.5, 120.1, 114.9, 88.1, 55.3, 49.0, 47.2, 28.2, 23.8, 14.8, 12.2, 7.3.

Tridachiahydropyrone analogue **10n**: synthesized from 2-((*E*)-4-((*E*)-4-bromobenzylidene)-7-chlorohept-2-en-2-yl)-6-methoxy-3,5-dimethyl-4*H*-pyran-4-one (**9n**). **9n** was synthesized via Step A, affording 0.219 g (77% yield) as a yellow oil. **10n** was synthesized via Step B, affording 0.074 g (34% yield) as a white solid. HRESIMS *m/z* 451.0656 $[M + H]^+$ (calcd. for $C_{22}H_{25}BrClO_3$, 451.0670); $^{1}$**H NMR** (600 MHz, $CDCl_3$) $\delta_H$ 7.39 (overlap, 3H), 7.06 (d, $J$ = 7.5 Hz, 1H), 5.61 (s, 1H), 4.47 (s, 1H), 3.97 (s, 3H), 3.45 – 3.34 (m, 2H), 2.02 (dt, $J$ = 15.4, 8.0 Hz, 1H), 1.84 (overlap, 4H), 1.82 – 1.67 (m, 2H), 1.64 (s, 3H), 0.91 (s, 3H); $^{13}$**C NMR** (151 MHz, $CDCl_3$) $\delta_C$ 195.5, 165.6, 145.1, 139.2, 136.4, 134.1, 131.5, 131.2, 131.0, 122.4, 120.7, 114.8, 88.2, 55.3, 48.3, 47.2, 44.4, 32.2, 30.3, 23.7, 14.7, 7.4.

Tridachiahydropyrone analogue **10o**: synthesized from 2-methoxy-3,5-dimethyl-6-((*E*)-4-((*E*)-4-(trifluoromethyl)benzylidene)hex-2-en-2-yl)-4*H*-pyran-4-one (**9o**). **9o** was synthesized via Step A, affording 0.168 g (68% yield) as a yellow oil. **10o** was synthesized via Step B, affording 0.059 g (35% yield) as a yellow oil. HRESIMS *m/z* 393.1648 $[M + H]^+$ (calcd. for $C_{22}H_{24}F_3O_3$, 393.1672); $^{1}$**H NMR** (600 MHz, $CDCl_3$) $\delta_H$ 7.66 (d, $J$ = 8.0 Hz, 1H), 7.54 (d, $J$ = 8.1 Hz, 1H), 7.50 (d, $J$ = 8.3 Hz, 1H), 7.29 (d, $J$ = 8.2 Hz, 1H), 5.58 (d, $J$ = 1.7 Hz, 1H), 4.60 (s, 1H), 3.98 (s, 3H), 1.86 (s, 3H), 1.81 (dt, $J$ = 15.0, 7.6 Hz, 1H), 1.72 – 1.67 (m, 1H), 1.64 (s, 3H), 0.93 (s, 3H), 0.88 (t, $J$ = 7.4 Hz, 3H); $^{13}$**C NMR** (151 MHz, $CDCl_3$) $\delta_C$195.6, 165.5, 144.8, 144.5, 139.5, 132.9, 130.2, 128.8 (d, $J$ = 32.2 Hz), 127.8 (d, $J$ = 194.0 Hz), 124.5 (d, $J$ = 272.0 Hz), 124.7 (d, $J$ = 68.6 Hz), 120.5, 114.9, 88.1, 55.3, 49.5, 47.2, 28.2, 23.8, 14.8, 12.1, 7.3; $^{19}$**F NMR** (565 MHz, $CDCl_3$) $\delta_F$ -62.3.

Tridachiahydropyrone analogue **10p**: synthesized from 2-methoxy-3,5-dimethyl-6-((*E*)-4-((*E*)-4-(trifluoromethyl)benzylidene)hept-2-en-2-yl)-4*H*-pyran-4-one (**9p**). **9p** was synthesized via Step A, affording 0.166 g (65% yield) as a yellow oil. **10p** was synthesized via Step B, affording 0.056 g (34% yield) as a yellow oil. HRESIMS *m/z* 407.1813 $[M + H]^+$ (calcd. for $C_{23}H_{26}F_3O_3$, 407.1828); $^{1}$**H NMR** (600 MHz, $CDCl_3$) $\delta_H$ 7.65 (d, $J$ = 7.7 Hz, 1H), 7.54 (d, $J$ = 7.7 Hz, 1H), 7.50 (d, $J$ = 7.9 Hz, 1H), 7.30 (d, $J$ = 7.8 Hz, 1H), 5.58 (s, 1H), 4.59 (s, 1H), 3.97 (s, 3H), 1.85 (s, 3H), 1.76 – 1.68 (m, 2H), 1.64 (s, 3H), 1.39 – 1.30 (m, 1H), 1.24 (td, $J$ = 15.0, 7.6 Hz, 1H), 0.91 (s, 3H), 0.74 (t, $J$ = 7.3 Hz, 3H); $^{13}$**C NMR** (151 MHz, $CDCl_3$) $\delta_C$ 195.6, 165.5, 144.8, 144.5, 137.9, 132.8, 130.3,

128.8 (d, $J$ = 32.1 Hz), 125.4, 124.7 (d, $J$ = 69.6 Hz), 123.6, 121.5, 114.9, 88.1, 55.3, 49.2, 47.2, 37.4, 23.8, 20.7, 14.8, 13.8, 7.3; **$^{19}$F NMR** (565 MHz, $CDCl_3$) $\delta_F$ -62.3.

Tridachiahydropyrone analogue **10q**: synthesized from 2-methoxy-3,5-dimethyl-6-((*E*)-4-((*E*)-4-(trifluoromethyl)benzylidene)oct-2-en-2-yl)-4*H*-pyran-4-one (**9q**). **9q** was synthesized via Step A, affording 0.164 g (62% yield) as a yellow oil. **10q** was synthesized via Step B, affording 0.049 g (30% yield) as a yellow oil. HRESIMS *m/z* 421.1983 $[M + H]^+$ (calcd. for $C_{24}H_{28}F_3O_3$, 421.1985); **$^1$H NMR** (600 MHz, $CDCl_3$) $\delta_H$ 7.66 (d, $J$ = 7.7 Hz, 1H), 7.55 (d, $J$ = 8.0 Hz, 1H), 7.50 (d, $J$ = 8.0 Hz, 1H), 7.30 (d, $J$ = 7.8 Hz, 1H), 5.58 (d, $J$ = 1.5 Hz, 1H), 4.60 (s, 1H), 3.98 (s, 3H), 1.85 (s, 3H), 1.78 (ddd, $J$ = 16.0, 9.4, 6.6 Hz, 1H), 1.70 (ddd, $J$ = 15.0, 9.2, 5.0 Hz, 1H), 1.65 (s, 3H), 1.35 – 1.24 (m, 1H), 1.24 – 1.13 (m, 2H), 1.13 – 1.04 (m, 1H), 0.92 (s, 3H), 0.77 (t, $J$ = 7.3 Hz, 3H); **$^{13}$C NMR** (151 MHz, $CDCl_3$) $\delta_C$ 195.6, 165.5, 144.8, 144.5, 138.2, 132.8, 130.3, 128.8 (q, $J$ = 32.2 Hz), 125.4, 124.7 (d, $J$ = 72.1 Hz), 123.6, 121.4, 114.9, 88.1, 55.3, 49.3, 47.2, 35.0, 29.7, 23.8, 22.4, 14.8, 14.0, 7.3; **$^{19}$F NMR** (565 MHz, $CDCl_3$) $\delta_F$ -62.3.

Tridachiahydropyrone analogue **10r**: synthesized from 2-methoxy-3,5-dimethyl-6-((*E*)-5-methyl-4-((*E*)-4-(trifluoromethyl)benzylidene)hex-2-en-2-yl)-4*H*-pyran-4-one (**9r**). **9r** was synthesized via Step A, affording 0.163 g (64% yield) as a yellow oil. **10r** was synthesized via Step B, affording 0.046 g (28% yield) as a yellow oil. HRESIMS *m/z* 407.1830 $[M + H]^+$ (calcd. for $C_{23}H_{26}F_3O_3$, 407.1828); **$^1$H NMR** (600 MHz, $CDCl_3$) $\delta_H$ 7.61 (d, $J$ = 7.6 Hz, 1H), 7.54 (d, $J$ = 8.0 Hz, 1H), 7.50 (d, $J$ = 8.2 Hz, 1H), 7.30 (d, $J$ = 8.0 Hz, 1H), 5.59 (s, 1H), 4.71 (s, 1H), 3.98 (s, 3H), 1.88 (overlap, 4H), 1.65 (s, 3H), 0.94 (d, $J$ = 6.8 Hz, 3H), 0.89 (d, $J$ = 6.8 Hz, 3H), 0.88 (s, 3H); **$^{13}$C NMR** (151 MHz, $CDCl_3$) $\delta_C$ 195.4, 165.8, 145.2, 144.6, 132.5, 129.9, 129.0, 128.8, 125.0, 124.7, 123.6, 118.4, 115.3, 88.2, 55.3, 48.5, 47.4, 31.6, 23.7, 22.7, 20.8, 15.0, 7.4; **$^{19}$F NMR** (565 MHz, $CDCl_3$) $\delta_F$ -62.3.

Tridachiahydropyrone analogue **10s**: synthesized from 2-((2*E*,4*E*,6*E*)-4,6-dimethylocta-2,4,6-trien-2-yl)-6-methoxy-3,5-dimethyl-4*H*-pyran-4-one (**9s**). **9s** was synthesized via Step A, affording 0.136 g (75% yield) as a colorless oil. **10s** was synthesized via Step B, affording 0.035 g (26% yield) as a white solid. HRESIMS *m/z* 289.1795 $[M + H]^+$ (calcd. for $C_{18}H_{25}O_3$, 289.1798); **$^1$H NMR** (600 MHz, $CDCl_3$) $\delta_H$ 5.58 (q, $J$ = 6.8 Hz, 1H), 5.44 (s, 1H), 3.96 (s, 3H), 3.91 (s, 1H), 1.75 (s, 3H), 1.63 (s, 3H), 1.61 (s, 3H), 1.59 (d, $J$ = 6.2 Hz, 3H), 1.53 (s, 3H), 1.16 (s, 3H); **$^{13}$C NMR** (151 MHz, $CDCl_3$) $\delta_C$ 196.0, 166.0, 145.4, 134.6, 134.1, 125.2, 121.4, 115.7, 88.0, 55.2, 53.4, 46.8, 21.9, 21.5, 14.8, 13.8, 13.5, 7.5.

Tridachiahydropyrone analogue **10t**: synthesized from 2-((2*E*,4*E*)-5-(cyclopent-1-en-1-yl)-4-methylpenta-2,4-dien-2-yl)-6-methoxy-3,5-dimethyl-4*H*-pyran-4-one (**9t**). **9t** was synthesized via Step A, affording 0.151 g (80% yield) as a colorless oil. **10t** was synthesized via Step B, affording 0.042 g (28% yield) as a white solid. HRESIMS *m/z* 301.1795 $[M + H]^+$ (calcd. for $C_{19}H_{25}O_3$, 301.1798); **$^1$H NMR** (600 MHz, $CDCl_3$) $\delta_H$ 5.57 (s, 1H), 5.43 (s, 1H), 3.95 (s, 3H), 3.94 (s, 1H), 2.38 – 2.24 (m, 4H), 1.84 (qd, $J$ = 7.7, 2.4 Hz, 2H), 1.75 (s, 3H), 1.64 (s, 3H), 1.63 (s, 3H), 1.19 (s, 3H); **$^{13}$C NMR** (151 MHz, $CDCl_3$) $\delta_C$ 195.6, 165.7, 145.3, 143.0, 134.4, 129.8, 121.3, 115.2, 88.1, 55.2, 47.1, 46.5, 35.0, 32.3, 24.1, 21.7, 20.7, 14.5, 7.3.

Tridachiahydropyrone analogue **10u**: synthesized from 2-methoxy-3,5-dimethyl-6-((2*E*,4*E*)-4-methylhexa-2,4-dien-2-yl)-4*H*-pyran-4-one (**9u**). **9u** was synthesized via Step A, affording 0.081

g (52% yield) as a colorless oil. **10u** was synthesized via Step B, affording 0.011 g (13% yield) as a colorless oil. HRESIMS *m/z* 249.1489 $[M + H]^+$ (calcd. for $C_{15}H_{21}O_3$, 249.1485); **$^1$H NMR** (600 MHz, $CDCl_3$) $\delta_H$ 5.41 (s, 1H), 3.95 (s, 3H), 2.95 (d, $J$ = 7.2 Hz, 1H), 1.75 (overlap, 6H), 1.65 (s, 3H), 1.43 (d, $J$ = 7.3 Hz, 3H), 1.17 (s, 3H); **$^{13}$C NMR** (151 MHz, $CDCl_3$) $\delta_C$ 196.2, 164.6, 145.4, 135.9, 121.3, 113.9, 87.9, 55.0, 46.0, 39.2, 21.1, 17.6, 14.5, 14.2, 7.0.

Tridachiahydropyrone analogue **10v**: synthesized from 2-methoxy-3,5-dimethyl-6-((*E*)-5-methyl-4-((*E*)-4-nitrobenzylidene)hex-2-en-2-yl)-4H-pyran-4-one (**9v**). **9v** was synthesized via Step A, affording 0.152 g (63% yield) as a yellow oil. **10v** was synthesized via Step B, affording 0.053 g (35% yield) as a yellow oil. HRESIMS *m/z* 384.1810 $[M + H]^+$ (calcd. for $C_{22}H_{26}NO_5$, 384.1805); **$^1$H NMR** (600 MHz, $CDCl_3$) $\delta_H$ 7.99 (d, $J$ = 8.8 Hz, 2H), 7.48 (d, $J$ = 8.8 Hz, 2H), 5.73 (s, 1H), 3.94 (s, 3H), 3.79 (s, 1H), 2.08 (p, $J$ = 6.8 Hz, 1H), 1.91 (s, 3H), 1.49 (s, 3H), 1.40 (s, 3H), 0.96 (overlap, 6H); **$^{13}$C NMR** (151 MHz, $CDCl_3$) $\delta_C$ 191.6, 164.8, 147.0, 146.3, 145.2, 144.1, 131.0, 122.9, 120.5, 113.5, 89.6, 55.0, 51.2, 48.8, 33.2, 25.3, 21.7, 21.3, 13.8, 6.4.

***Steps C and D:*** To a solution of compound **10** (0.045 mmol, 1.0 eq.) and acid catalyst 4 M HCl·MeOH (1.13 μL, 10 mol%) in dry MeOH (450 μL, 0.1 M) at room temperature. The reaction was carried out at room temperature for 45 minutes. After completion, the solvent was removed under reduced pressure to give a crude mixture. The above crude mixture was dissolved in HFIP/DCM (450 μL, 0.1 M, 4:1 v/v), followed by the addition of 4 M HCl·EtOAc (0.56 μL, 5 mol%). The reaction was allowed to proceed at room temperature for 2–8 hours and was monitored by thin-layer chromatography (TLC). Upon completion, the solvent was removed under reduced pressure to afford the crude product. The crude residue was purified by flash chromatography (PE/EtOAc) to afford ocellatusone C analogues **11**.

Ocellatusone C analogue **11a**: synthesized from **10a** via a two-step sequence (steps C and D) to give 10.9 mg of a white solid with an overall yield of 78%. HRESIMS *m/z* 311.1644 $[M + H]^+$ (calcd. for $C_{20}H_{23}O_3$, 311.1642); **$^1$H NMR** (600 MHz, $CDCl_3$) $\delta_H$ 7.30 – 7.27 (m, 2H), 7.26 – 7.23 (m, 1H), 6.95 (s, 2H), 5.74 (d, $J$ = 1.5 Hz, 1H), 3.96 (s, 3H), 3.75 (s, 1H), 1.90 (s, 3H), 1.43 (d, $J$ = 1.4 Hz, 3H), 1.42 (s, 3H), 0.86 (s, 3H); **$^{13}$C NMR** (151 MHz, $CDCl_3$) $\delta_C$ 207.2, 202.4, 178.8, 137.1, 136.2, 133.0, 128.6, 127.7, 117.2, 61.8, 61.0, 60.9, 53.6, 21.8, 16.1, 15.7, 10.5.

Ocellatusone C analogue **11b**: synthesized from **10b** via a two-step sequence (steps C and D) to give 11.1 mg of a white solid with an overall yield of 75%. HRESIMS *m/z* 329.1549 $[M + H]^+$ (calcd. for $C_{20}H_{22}FO_3$, 329.1548); **$^1$H NMR** (600 MHz, $CDCl_3$) $\delta_H$ 6.97 (overlap, 4H), 5.73 (d, $J$ = 1.5 Hz, 1H), 3.96 (s, 3H), 3.74 (s, 1H), 1.90 (s, 3H), 1.43 (d, $J$ = 0.8 Hz, 3H), 1.41 (s, 3H), 0.85 (s, 3H); **$^{13}$C NMR** (151 MHz, $CDCl_3$) $\delta_C$ 207.0, 202.0, 178.4, 162.2 (d, $J$ = 246.3 Hz), 135.9, 133.2, 132.6 (d, $J$ = 3.3 Hz), 117.0, 115.4 (d, $J$ = 21.3 Hz), 61.7, 60.7, 60.0, 53.5, 21.5, 16.0, 15.5, 10.4; **$^{19}$F NMR** (565 MHz, $CDCl_3$) $\delta_F$ -114.9 (q, $J$ = 7.2 Hz).

Ocellatusone C analogue **11c**: synthesized from **10c** via a two-step sequence (steps C and D) to give 11.2 mg of a white solid with an overall yield of 72%. HRESIMS *m/z* 345.1252 $[M + H]^+$ (calcd. for $C_{20}H_{22}ClO_3$, 345.1252); **$^1$H NMR** (600 MHz, $CDCl_3$) $\delta_H$ 7.27 (s, 2H), 6.90 (s, 2H), 5.76 (d, $J$ = 1.4 Hz, 1H), 3.97 (s, 3H), 3.75 (s, 1H), 1.92 (s, 3H), 1.44 (d, $J$ = 1.4 Hz, 3H), 1.42 (s, 3H), 0.87 (s, 3H); **$^{13}$C NMR** (151 MHz, $CDCl_3$) $\delta_C$ 207.0, 202.0, 178.5, 135.8, 135.6, 133.6, 133.5, 128.8, 117.1, 61.9, 60.7, 60.3, 53.6, 21.7, 16.1, 15.7, 10.5.

Ocellatusone C analogue **11d**: synthesized from **10d** via a two-step sequence (steps C and D) to give 13.1 mg of a white solid with an overall yield of 75%. HRESIMS *m/z* 389.0749 $[M + H]^+$ (calcd. for $C_{20}H_{22}BrO_3$, 389.0747); $^1$**H NMR** (600 MHz, $CDCl_3$) $\delta_H$ 7.41 (d, $J$ = 8.9 Hz, 2H), 6.83 (s, 2H), 5.74 (d, $J$ = 1.5 Hz, 1H), 3.96 (s, 3H), 3.72 (s, 1H), 1.90 (s, 3H), 1.42 (s, 3H), 1.41 (s, 3H), 0.85 (s, 3H); $^{13}$**C NMR** (151 MHz, $CDCl_3$) $\delta_C$ 206.9, 202.0, 178.5, 136.2, 135.7, 133.5, 131.8, 121.8, 117.1, 61.9, 60.6, 60.3, 53.6, 21.7, 16.1, 15.7, 10.5.

Ocellatusone C analogue **11e**: synthesized from **10e** via a two-step sequence (steps C and D) to give 12.8 mg of a white solid with an overall yield of 75%. HRESIMS *m/z* 379.1520 $[M + H]^+$ (calcd. for $C_{21}H_{22}F_3O_3$, 379.1516); $^1$**H NMR** (600 MHz, $CDCl_3$) $\delta_H$ 7.55 (d, $J$ = 8.1 Hz, 2H), 7.08 (s, 2H), 5.79 (s, 1H), 3.97 (s, 3H), 3.83 (s, 1H), 1.92 (s, 3H), 1.43 (overlap, 6H), 0.85 (s, 3H); $^{13}$**C NMR** (151 MHz, $CDCl_3$) $\delta_C$ 206.8, 201.8, 178.5, 141.3, 135.4, 133.9, 132.6, 130.1 (d, $J$ = 32.6 Hz), 125.6, 125.1, 123.3, 117.1, 61.9, 60.5, 60.5, 53.6, 21.7, 16.1, 15.7, 10.6; $^{19}$**F NMR** (565 MHz, $CDCl_3$) $\delta_F$ -62.6.

Ocellatusone C analogue **11f**: synthesized from **10f** via a two-step sequence (steps C and D) to give 11.7 mg of a white solid with an overall yield of 73%. HRESIMS *m/z* 356.1494 $[M + H]^+$ (calcd. for $C_{20}H_{22}NO_5$, 356.1493); $^1$**H NMR** (600 MHz, $CDCl_3$) $\delta_H$ 8.16 (d, $J$ = 9.1 Hz, 2H), 7.14 (s, 2H), 5.82 (d, $J$ = 1.5 Hz, 1H), 3.99 (s, 3H), 3.89 (s, 1H), 1.93 (s, 3H), 1.44 (overlap, 6H), 0.85 (s, 3H); $^{13}$**C NMR** (151 MHz, $CDCl_3$) $\delta_C$ 206.5, 201.3, 178.3, 147.7, 144.9, 135.0, 134.4, 123.9, 117.0, 62.0, 60.4, 60.2, 53.6, 21.7, 16.1, 15.7, 10.6.

Ocellatusone C analogue **11g**: synthesized from **10g** via a two-step sequence (steps C and D) to give 13.2 mg of a white solid with an overall yield of 80%. HRESIMS *m/z* 367.2226 $[M + H]^+$ (calcd. for $C_{24}H_{31}O_3$, 367.2268); $^1$**H NMR** (600 MHz, $CDCl_3$) $\delta_H$ 7.27 (d, $J$ = 9.1 Hz, 2H), 6.86 (s, 2H), 5.72 (d, $J$ = 1.5 Hz, 1H), 3.95 (s, 3H), 3.72 (s, 1H), 1.90 (s, 3H), 1.43 (d, $J$ = 1.4 Hz, 3H), 1.42 (s, 3H), 1.28 (s, 9H), 0.86 (s, 3H); $^{13}$**C NMR** (151 MHz, $CDCl_3$) $\delta_C$ 207.4, 202.5, 178.8, 150.5, 136.4, 133.8, 132.8, 125.4, 117.3, 61.8, 61.1, 60.6, 53.6, 34.6, 31.5, 21.8, 16.2, 15.7, 10.5.

Ocellatusone C analogue **11h**: synthesized from **10h** via a two-step sequence (steps C and D) to give 13.3 mg of a white solid with an overall yield of 78%. HRESIMS *m/z* 379.0858 $[M + H]^+$ (calcd. for $C_{20}H_{21}Cl_2O_3$, 379.0862); $^1$**H NMR** (600 MHz, $CDCl_3$) $\delta_H$ 7.28 – 7.25 (m, 1H), 6.84 (s, 2H), 5.78 (d, $J$ = 1.4 Hz, 1H), 3.97 (s, 3H), 3.70 (s, 1H), 1.90 (s, 3H), 1.44 (d, $J$ = 1.4 Hz, 3H), 1.42 (s, 3H), 0.89 (s, 3H); $^{13}$**C NMR** (151 MHz, $CDCl_3$) $\delta_C$ 206.4, 201.5, 178.3, 140.8, 135.0, 134.3, 128.2, 117.0, 61.9, 60.3, 60.1, 53.6, 21.7, 16.1, 15.7, 10.5.

Ocellatusone C analogue **11i**: synthesized from **10i** via a two-step sequence (steps C and D) to give 12.6 mg of a white solid with an overall yield of 70%. HRESIMS *m/z* 401.1958 $[M + H]^+$ (calcd. for $C_{23}H_{29}O_6$, 401.1959); $^1$**H NMR** (600 MHz, $CDCl_3$) $\delta_H$ 6.01 (s, 2H), 5.73 (d, $J$ = 1.5 Hz, 1H), 3.97 (s, 3H), 3.83 (overlap, 9H), 3.65 (s, 3H), 1.91 (s, 3H), 1.48 (d, $J$ = 1.4 Hz, 3H), 1.40 (s, 3H), 0.90 (s, 3H); $^{13}$**C NMR** (151 MHz, $CDCl_3$) $\delta_C$ 207.3, 202.3, 178.6, 137.4, 136.4, 133.0, 132.6, 117.1, 61.9, 61.3, 61.1, 61.0, 56.1, 53.6, 21.8, 16.1, 15.9, 10.5.

Ocellatusone C analogue **11j**: synthesized from **10j** via a two-step sequence (steps C and D) to give 13.0 mg of a colorless oil with an overall yield of 68%. HRESIMS *m/z* 425.05516 $[M + H]^+$

(calcd. for $C_{20}H_{20}BrF_2O_3$, 425.05584); **$^1$H NMR** (600 MHz, $CDCl_3$) $\delta_H$ 7.12 (dt, $J$ = 8.6, 1.8 Hz, 1H), 6.97 (dt, $J$ = 10.0, 1.9 Hz, 1H), 5.72 (s, 1H), 4.25 (s, 1H), 3.96 (s, 3H), 1.89 (s, 3H), 1.49 (d, $J$ = 0.8 Hz, 3H), 1.38 (s, 3H), 1.00 (s, 3H); **$^{13}$C NMR** (151 MHz, $CDCl_3$) $\delta_C$ 206.2, 201.5, 179.1, 133.9, 132.0, 116.9, 116.3 (d, $J$ = 25.8 Hz), 115.5 (d, $J$ = 27.5 Hz), 61.9, 59.1, 53.3, 49.1 (d, $J$ = 3.8 Hz), 21.5, 15.2, 15.1, 10.5; **$^{19}$F NMR** (565 MHz, $CDCl_3$) $\delta_F$ -105.6 (dd, $J$ = 10.7, 4.1 Hz), -110.0 – -111.2 (m).

Ocellatusone C analogue **11k**: synthesized from **10k** via a two-step sequence (steps C and D) to give 15.9 mg of a white solid with an overall yield of 71%. HRESIMS $m/z$ 496.9956 $[M + H]^+$ (calcd. for $C_{21}H_{23}Br_2O_4$, 496.9958); **$^1$H NMR** (600 MHz, $CDCl_3$) $\delta_H$ 7.06 (s, 2H), 5.77 (d, $J$ = 1.5 Hz, 1H), 3.97 (s, 3H), 3.87 (s, 3H), 3.65 (s, 1H), 1.90 (s, 3H), 1.45 (s, 3H), 1.41 (s, 3H), 0.90 (s, 3H); **$^{13}$C NMR** (151 MHz, $CDCl_3$) $\delta_C$ 206.5, 201.5, 178.3, 153.7, 135.9, 135.1, 134.2, 117.0, 61.9, 60.8, 60.5, 59.4, 53.6, 21.7, 16.2, 15.7, 10.5.

Ocellatusone C analogue **11l**: synthesized from **10l** via a two-step sequence (steps C and D) to give 11.4 mg of a white solid with an overall yield of 78%. HRESIMS $m/z$ 325.1793 $[M + H]^+$ (calcd. for $C_{21}H_{25}O_3$, 325.1798); **$^1$H NMR** (600 MHz, $CDCl_3$) $\delta_H$ 7.17 (d, $J$ = 7.3 Hz, 1H), 7.12 (td, $J$ = 7.3, 1.5 Hz, 1H), 7.09 (td, $J$ = 7.5, 1.6 Hz, 1H), 6.62 (dd, $J$ = 7.7, 1.5 Hz, 1H), 5.70 (d, $J$ = 1.6 Hz, 1H), 4.19 (s, 1H), 3.96 (s, 3H), 2.42 (s, 3H), 1.91 (s, 3H), 1.44 (s, 3H), 1.39 (d, $J$ = 0.8 Hz, 3H), 0.86 (s, 3H); **$^{13}$C NMR** (151 MHz, $CDCl_3$) $\delta_C$ 207.6, 202.5, 179.2, 137.9, 137.1, 135.9, 132.4, 130.7, 127.3, 126.7, 126.6, 117.2, 61.9, 61.2, 55.2, 53.3, 21.6, 20.5, 15.9, 14.7, 10.5.

Ocellatusone C analogue **11m**: synthesized from **10m** via a two-step sequence (steps C and D) to give 13.4 mg of a colorless oil with an overall yield of 74%. HRESIMS $m/z$ 403.0919 $[M + H]^+$ (calcd. for $C_{21}H_{24}BrO_3$, 403.0903); **$^1$H NMR** (600 MHz, $CDCl_3$) $\delta_H$ 7.40 (d, $J$ = 9.0 Hz, 2H), 6.82 (s, 2H), 5.73 (s, 1H), 3.96 (s, 3H), 3.80 (s, 1H), 1.90 (s, 3H), 1.69 (q, $J$ = 7.1 Hz, 2H), 1.42 (s, 3H), 0.92 – 0.82 (overlap, 6H); **$^{13}$C NMR** (151 MHz, $CDCl_3$) $\delta_C$ 207.1, 202.0, 178.5, 140.9, 136.4, 131.8, 131.7, 121.7, 117.1, 61.9, 60.7, 59.1, 53.6, 27.5, 16.1, 15.8, 11.8, 10.5.

Ocellatusone C analogue **11n**: synthesized from **10n** via a two-step sequence (steps C and D) to give 14.6 mg of a colorless oil with an overall yield of 72%. HRESIMS $m/z$ 451.0665 $[M + H]^+$ (calcd. for $C_{22}H_{25}BrClO_3$, 451.0670); **$^1$H NMR** (600 MHz, $CDCl_3$) $\delta_H$ 7.41 (d, $J$ = 8.8 Hz, 2H), 6.82 (s, 2H), 5.83 (s, 1H), 3.97 (s, 3H), 3.78 (s, 1H), 3.40 (dt, $J$ = 11.3, 5.8 Hz, 1H), 3.28 (ddd, $J$ = 10.9, 8.0, 5.6 Hz, 1H), 1.89 (overlap, 4H), 1.81 – 1.63 (overlap, 3H), 1.42 (s, 3H), 0.86 (s, 3H); **$^{13}$C NMR** (151 MHz, $CDCl_3$) $\delta_C$ 206.6, 201.9, 178.4, 137.7, 135.8, 134.4, 131.8, 122.0, 117.0, 62.0, 60.8, 58.6, 53.8, 43.9, 31.4, 30.0, 16.0, 15.6, 10.5.

Ocellatusone C analogue **11o**: synthesized from **10o** via a two-step sequence (steps C and D) to give 12.7 mg of a colorless oil with an overall yield of 72%. HRESIMS $m/z$ 393.1655 $[M + H]^+$ (calcd. for $C_{22}H_{24}F_3O_3$, 393.1672); **$^1$H NMR** (600 MHz, $CDCl_3$) $\delta_H$ 7.53 (d, $J$ = 8.0 Hz, 2H), 7.07 (s, 2H), 5.77 (s, 1H), 3.97 (s, 3H), 3.90 (s, 1H), 1.91 (s, 3H), 1.68 (q, $J$ = 7.3 Hz, 2H), 1.44 (s, 3H), 0.90 – 0.82 (overlap, 6H); **$^{13}$C NMR** (151 MHz, $CDCl_3$) $\delta_C$ 207.0, 201.8, 178.5, 141.5, 140.6, 132.1, 130.0 (d, $J$ = 32.4 Hz), 125.5, 124.2 (d, $J$ = 272.1 Hz), 117.1, 62.0, 60.6, 59.3, 53.6, 27.5, 16.1, 15.8, 11.8, 10.6; **$^{19}$F NMR** (565 MHz, $CDCl_3$) $\delta_F$ -62.6.

Ocellatusone C analogue **11p**: synthesized from **10p** via a two-step sequence (steps C and D) to give 13.7 mg of a colorless oil with an overall yield of 75%. HRESIMS *m/z* 407.1849 $[M + H]^+$ (calcd. for $C_{23}H_{26}F_3O_3$, 407.1828); **$^1$H NMR** (600 MHz, $CDCl_3$) $\delta_H$ 7.54 (d, $J$ = 8.2 Hz, 2H), 7.07 (s, 2H), 5.79 (s, 1H), 3.97 (s, 3H), 3.90 (s, 1H), 1.90 (s, 3H), 1.63 (t, $J$ = 8.3 Hz, 3H), 1.44 (s, 3H), 1.35 (td, $J$ = 13.6, 7.3 Hz, 1H), 1.29 – 1.17 (m, 1H), 0.86 (s, 3H), 0.73 (t, $J$ = 7.3 Hz, 3H); **$^{13}$C NMR** (151 MHz, $CDCl_3$) $\delta_C$ 206.9, 201.7, 178.7, 141.4, 139.0, 133.5, 130.0 (d, $J$ = 32.4 Hz), 125.5, 124.2 (d, $J$ = 272.3 Hz), 117.1, 62.0, 60.7, 58.9, 53.8, 36.5, 20.4, 16.1, 15.7, 13.4, 10.5; **$^{19}$F NMR** (565 MHz, $CDCl_3$) $\delta_F$ -62.6.

Ocellatusone C analogue **11q**: synthesized from **10q** via a two-step sequence (steps C and D) to give 14.2 mg of a colorless oil with an overall yield of 75%. HRESIMS *m/z* 421.1981 $[M + H]^+$ (calcd. for $C_{24}H_{28}F_3O_3$, 421.1985); **$^1$H NMR** (600 MHz, $CDCl_3$) $\delta_H$ 7.54 (d, $J$ = 8.0 Hz, 2H), 7.07 (s, 2H), 5.78 (s, 1H), 3.97 (s, 3H), 3.90 (s, 1H), 1.90 (s, 3H), 1.71 – 1.59 (m, 2H), 1.44 (s, 3H), 1.34 – 1.24 (m, 1H), 1.23 – 1.04 (overlap, 3H), 0.85 (s, 3H), 0.79 (t, $J$ = 7.3 Hz, 3H); **$^{13}$C NMR** (151 MHz, $CDCl_3$) $\delta_C$ 206.9, 201.7, 178.6, 141.4, 139.3, 133.2, 130.0 (d, $J$ = 32.2 Hz), 125.5, 124.2 (d, $J$ = 272.2 Hz), 117.1, 62.0, 60.7, 59.0, 53.7, 34.2, 29.4, 22.1, 16.1, 15.7, 13.9, 10.5; **$^{19}$F NMR** (565 MHz, $CDCl_3$) $\delta_F$ -62.6.

Ocellatusone C analogue **11r**: synthesized from **10r** via a two-step sequence (steps C and D) to give 13.0 mg of a colorless oil with an overall yield of 71%. HRESIMS *m/z* 407.1829 $[M + H]^+$ (calcd. for $C_{23}H_{26}F_3O_3$, 407.1829); **$^1$H NMR** (600 MHz, $CDCl_3$) $\delta_H$ 7.53 (d, $J$ = 8.1 Hz, 2H), 7.07 (s, 2H), 5.80 (s, 1H), 4.01 (s, 1H), 3.97 (s, 3H), 1.91 (s, 3H), 1.78 (p, $J$ = 6.8 Hz, 1H), 1.45 (s, 3H), 0.87 (s, 3H), 0.84 (t, $J$ = 7.1 Hz, 6H); **$^{13}$C NMR** (151 MHz, $CDCl_3$) $\delta_C$ 207.0, 201.7, 178.3, 145.0, 141.6, 131.5, 130.0 (q, $J$ = 32.6 Hz), 125.5 (d, $J$ = 4.1 Hz), 124.2 (d, $J$ = 272.0 Hz), 117.0, 62.1, 61.0, 58.6, 53.7, 32.2, 22.3, 21.2, 16.1, 15.7, 10.5. **$^{19}$F NMR** (565 MHz, $CDCl_3$) $\delta_F$ -62.6.

Ocellatusone C analogue **11s**: synthesized from **10s** via a two-step sequence (steps C and D) to give 10.6 mg of a white solid with an overall yield of 82%. HRESIMS *m/z* 289.1796 $[M + H]^+$ (calcd. for $C_{18}H_{25}O_3$, 289.1798); **$^1$H NMR** (600 MHz, $CDCl_3$) $\delta_H$ 5.64 (s, 1H), 5.40 (s, 1H), 3.91 (s, 3H), 3.14 (s, 1H), 1.84 (s, 3H), 1.61 (d, $J$ = 6.6 Hz, 3H), 1.48 (s, 3H), 1.32 (s, 3H), 1.26 (s, 3H), 1.11 (s, 3H); **$^{13}$C NMR** (151 MHz, $CDCl_3$) $\delta_C$ 208.6, 202.4, 179.4, 135.5, 132.7, 132.0, 127.2, 117.2, 65.0, 61.8, 60.2, 53.3, 21.5, 15.7, 15.6, 13.7, 11.9, 10.4.

Ocellatusone C analogue **11t**: synthesized from **10t** via a two-step sequence (steps C and D) to give 11.4 mg of a white solid with an overall yield of 84%. HRESIMS *m/z* 301.1807 $[M + H]^+$ (calcd. for $C_{19}H_{25}O_3$, 301.1798); **$^1$H NMR** (600 MHz, $CDCl_3$) $\delta_H$ 5.60 (d, $J$ = 1.5 Hz, 1H), 5.56 (s, 1H), 3.91 (s, 3H), 3.43 (s, 1H), 2.33 – 2.24 (m, 2H), 2.01 – 1.92 (m, 1H), 1.84 (overlap, 5H), 1.79 – 1.71 (m, 1H), 1.50 (d, $J$ = 1.0 Hz, 3H), 1.30 (s, 3H), 1.14 (s, 3H); **$^{13}$C NMR** (151 MHz, $CDCl_3$) $\delta_C$ 208.3, 202.3, 179.2, 140.6, 135.5, 132.3, 132.0, 117.4, 61.8, 60.1, 57.1, 53.3, 32.2, 31.9, 23.9, 21.5, 15.7, 15.5, 10.4.

## Part 5. Synthesis of Compounds 12–16 by Photoinduced Skeletal Evolution

**Table S2.** Screening of reaction conditions for the photoinduced skeletal diversification and evolution.

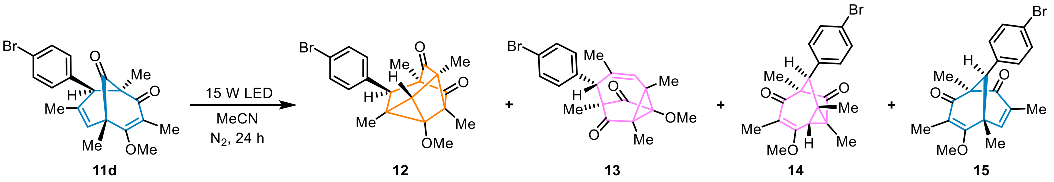


| entry | substrate | conditions | products |
|---|---|---|---|
| 1 | **11d** | 395 nm | **12** (16%), **13** (16%) and **14** (11%) |
| 2 | **11d** | 425 nm | **12** (trace), **13** (trace) and **14** (trace) |
| 3 | **11d** | 455 nm | no reaction |
| 4 | **11d** | 455 nm **[Ir-F][PF$_6$]** (10 mol%) | **13** (27%) and **15** (69%) |
| 5 | **15** | 455 nm **[Ir-F][PF$_6$]** (10 mol%) | no reaction |
| 6 | **15** | 395 nm | **12** (33%) and **14** (42%) |

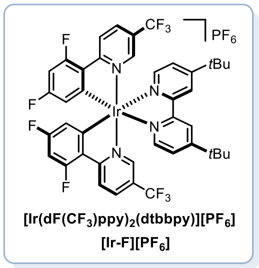


Entry 1: To an oven-dried 10 mL glass tube equipped with a stir bar was added compound **11d** (20.0 mg, 0.051 mmol, 1.0 eq.) in dry MeCN (3 mL). The reaction vessel was sealed with a Teflon screw cap, evacuated and back-filled with $N_2$ (three cycles), and the mixture was stirred at room temperature under irradiation with 15 W violet LED (395 nm) for 24 hours. Upon completion of the reaction, the solution was concentrated under reduced pressure, and the residue was purified by RP-HPLC with 70% MeCN/$H_2O$ to give compound **12** as a white solid (3.2 mg, 16%), compound **13** as a white solid (3.2 mg, 16%), and compound **14** as a white solid (2.2 mg, 11%).

Tetracyclo[3.3.1.0$^{2,8}$.0$^{3,7}$]nonane derivative **12**: HRESIMS *m/z* 389.0755 [M + H]$^+$ (calcd. for $C_{20}H_{22}BrO_3$, 389.0746); **$^1$H NMR** (600 MHz, $CDCl_3$) $\delta_H$ 7.46 (t, $J$ = 9.6 Hz, 2H), 7.05 (d, $J$ = 8.5 Hz, 1H), 6.89 (d, $J$ = 7.4 Hz, 1H), 4.06 (s, 1H), 3.50 (s, 3H), 2.56 (s, 1H), 1.36 (s, 3H), 1.28 (s, 3H), 0.87 (s, 3H), 0.67 (s, 3H); **$^{13}$C NMR** (151 MHz, $CDCl_3$) $\delta_C$ 214.7, 214.0, 136.4, 132.5, 132.2, 131.6, 127.6, 122.2, 76.3, 72.3, 62.2, 58.9, 58.7, 49.3, 45.6, 28.4, 14.6, 11.9, 10.2, 7.0.

Tricyclo[4.2.1.0$^{2,8}$]nonane derivative **13**: HRESIMS *m/z* 389.0757 [M + H]$^+$ (calcd. for $C_{20}H_{22}BrO_3$, 389.0746); **$^1$H NMR** (600 MHz, $CDCl_3$) $\delta_H$ 7.41 (d, $J$ = 8.6 Hz, 2H), 7.00 (s, 1H), 6.69 (s, 1H), 5.68 (s, 1H), 3.60 (s, 3H), 3.01 (s, 1H), 1.49 (s, 3H), 1.42 (s, 3H), 1.40 (s, 3H), 0.96 (s, 3H); **$^{13}$C NMR** (151 MHz, $CDCl_3$) $\delta_C$ 208.8, 205.9, 136.2, 136.0, 129.6, 121.9, 87.9, 58.5, 58.0, 55.4, 53.9, 37.0, 25.0, 17.5, 16.4, 6.6.

Tricyclo[4.2.1.0$^{2,8}$]nonane derivative **14**: HRESIMS *m/z* 389.0758 [M + H]$^+$ (calcd. for $C_{20}H_{22}BrO_3$, 389.0746); **$^1$H NMR** (600 MHz, $CDCl_3$) $\delta_H$ 7.44 (t, $J$ = 7.4 Hz, 2H), 6.95 (d, $J$ = 8.1 Hz, 1H), 6.61 (d, $J$ = 8.4 Hz, 1H), 3.81 (s, 3H), 3.29 (s, 1H), 2.05 (s, 1H), 1.79 (s, 3H), 1.49 (s, 3H), 1.19 (s, 3H), 0.77 (s, 3H); **$^{13}$C NMR** (151 MHz, $CDCl_3$) $\delta_C$ 210.0, 192.2, 166.4, 137.4, 132.8, 132.0, 131.6, 129.7, 121.4, 116.8, 64.0, 57.2, 52.0, 41.3, 41.0, 35.6, 18.2, 17.0, 11.3, 10.9.

Entries 2-3: To an oven-dried 10 mL glass tube equipped with a stir bar was added compound **11d** (5.0 mg, 0.013 mmol, 1.0 eq.) in dry MeCN (1 mL). The reaction vessel was sealed with a Teflon screw cap, evacuated and back-filled with $N_2$ (three cycles), and the mixture was stirred at room

temperature under irradiation with 15 W LEDs (425 nm for entry 2, 455 nm for entry 3) for 24 hours. Upon completion of the reaction, the solution was concentrated under reduced pressure to give a residue, which was directly subjected to $^1$H NMR analysis.

Entry 4: To an oven-dried 10 mL glass tube equipped with a stir bar was added compound **11d** (20.0 mg, 0.051 mmol, 1.0 eq.) and Ir[dF($CF_3$)ppy]$_2$(dtbbpy)$PF_6$ (5.8 mg, 0.0051 mmol, 10 mol%) in dry MeCN (3 mL). The reaction vessel was sealed with a Teflon screw cap, evacuated and back-filled with $N_2$ (three cycles), and the mixture was stirred at room temperature under irradiation with 15 W azure LED (455 nm) for 24 hours. Upon completion of the reaction, the solution was concentrated under reduced pressure, and the residue was purified by flash chromatography (4% EtOAc in PE) to afford compound **13** as a white solid (5.4 mg, 27%), and compound **15** as a white solid (13.8 mg, 69%).

Bicyclo[3.3.1]nonane derivative **15**: HRESIMS *m/z* 389.0762 [M + H]$^+$ (calcd. for $C_{20}H_{22}BrO_3$, 389.0746); **$^1$H NMR** (600 MHz, $CDCl_3$) $\delta_H$ 7.42 (dd, $J$ = 8.2, 2.3 Hz, 1H), 7.35 (dd, $J$ = 8.5, 2.2 Hz, 1H), 6.97 (dd, $J$ = 8.2, 2.4 Hz, 1H), 6.90 (dd, $J$ = 8.5, 2.4 Hz, 1H), 6.50 (t, $J$ = 1.6 Hz, 1H), 3.91 (s, 3H), 3.72 (d, $J$ = 1.8 Hz, 1H), 1.84 – 1.80 (overlap, 6H), 1.13 (s, 3H), 1.10 (s, 3H); **$^{13}$C NMR** (151 MHz, $CDCl_3$) $\delta_C$ 195.3, 194.4, 180.0, 147.9, 137.1, 134.9, 133.1, 131.9, 131.7, 128.6, 121.9, 112.8, 62.0, 62.0, 61.2, 44.4, 20.9, 18.7, 16.1, 10.4.

Entry 5: To an oven-dried 10 mL glass tube equipped with a stir bar was added compound **15** (5.0 mg, 0.013 mmol, 1.0 eq.) and Ir[dF($CF_3$)ppy]$_2$(dtbbpy)$PF_6$ (1.4 mg, 0.0051 mmol, 10 mol%) in dry MeCN (1 mL). The reaction vessel was sealed with a Teflon screw cap, evacuated and back-filled with $N_2$ (three cycles), and the mixture was stirred at room temperature under irradiation with 15 W azure LED (455 nm) for 24 hours. Upon completion of the reaction, the solution was concentrated under reduced pressure to give a residue, which was directly subjected to $^1$H NMR analysis.

Entry 6: To an oven-dried 10 mL glass tube equipped with a stir bar was added compound **15** (20.0 mg, 0.051 mmol, 1.0 eq.) in dry MeCN (3 mL). The reaction vessel was sealed with a Teflon screw cap, evacuated and back-filled with $N_2$ (three cycles), and the mixture was stirred at room temperature under irradiation with 15 W violet LED (395 nm) for 24 hours. Upon completion of the reaction, the solution was concentrated under reduced pressure, and the residue was purified by flash chromatography (5% EtOAc in PE) to afford compound **12** as a white solid (6.6 mg, 33%), and compound **14** as a white solid (8.4 mg, 42%).

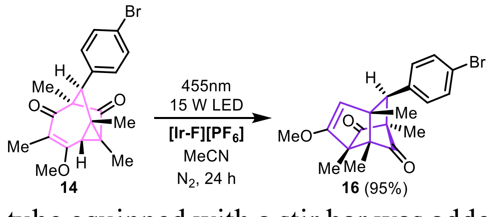


To an oven-dried 10 mL glass tube equipped with a stir bar was added compound **14** (10 mg, 0.026 mmol, 1.0 eq.) and Ir[dF($CF_3$)ppy]$_2$(dtbbpy)$PF_6$ (2.9 mg, 0.0026 mmol, 10 mol%) in dry MeCN (2 mL). The reaction vessel was sealed with a Teflon screw cap, evacuated and back-filled with $N_2$ (three cycles), and the mixture was stirred at room temperature under irradiation with 15 W azure LED (455 nm) for 24 hours. Upon completion of the reaction, the solution was concentrated

under reduced pressure, and the residue was purified by flash chromatography (4% EtOAc in PE) to afford compound **16** as a white solid (9.5 mg, 95%).

Octahydro-1,5-methanopentalene derivative **16**: HRESIMS *m/z* 389.0743 [M + H]$^+$ (calcd. for $C_{20}H_{22}BrO_3$, 389.0746); $^1$**H NMR** (600 MHz, $CDCl_3$) $\delta_H$ 7.38 (t, $J$ = 7.5 Hz, 2H), 6.89 – 6.81 (m, 2H), 4.71 (s, 1H), 3.66 (s, 3H), 3.45 (s, 1H), 1.06 (overlap, 6H), 0.91 (s, 3H), 0.71 (s, 3H); $^{13}$**C NMR** (151 MHz, $CDCl_3$) $\delta_C$ 215.2, 206.2, 158.9, 136.8, 133.4, 131.6, 131.6, 129.1, 121.6, 106.2, 69.6, 66.1, 65.5, 65.0, 57.9, 50.5, 20.2, 10.8, 7.4, 5.9.

## Part 6. Synthesis of Polycyclic Framework Derivatives 17–21 via Cross-Coupling

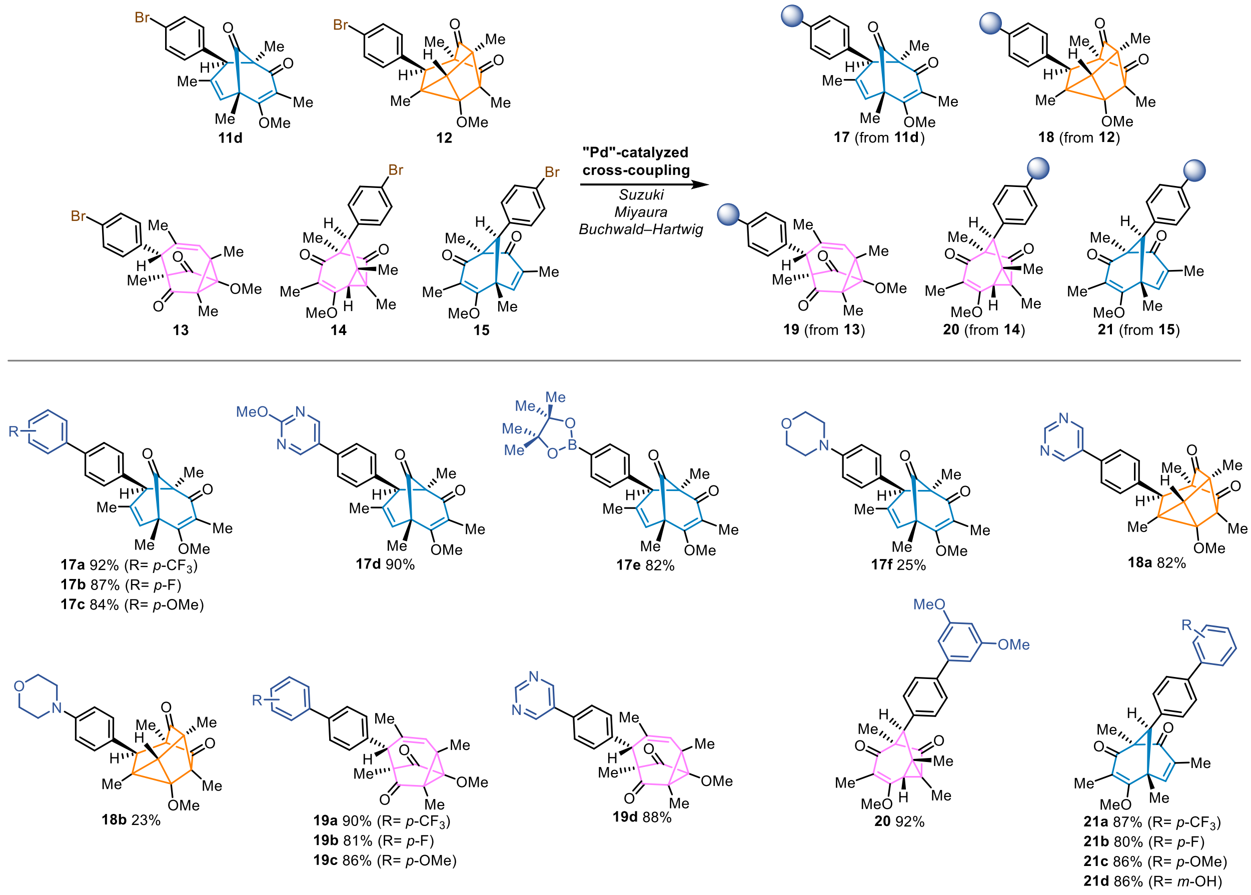


### General Procedure A for Suzuki Coupling:

To an oven-dried 10 mL glass tube equipped with a stir bar was added the polycyclic compound (7.0 mg, 0.018 mmol, 1.0 eq.), corresponding boronate ester or boronic acid (0.027 mmol, 1.5 eq.), $Cs_2CO_3$ (8.8 mg, 0.027 mmol, 1.5 eq.), $Pd(PPh_3)_4$ (2.1 mg, 0.0018 mmol, 10 mol%) in THF/$H_2O$ (0.6 mL, 5:1 v/v). The reaction vessel was sealed with a Teflon screw cap, evacuated and back-filled with $N_2$ (three cycles), and the mixture was heated to 80 °C in an oil bath with stirring for 12 hours. Upon completion of the reaction, the mixture was diluted with $H_2O$ (5 mL) and extracted with EtOAc (2 × 10 mL). The combined organic layers were dried over $Na_2SO_4$, filtered and concentrated under reduced pressure. The resulting crude residue was purified by flash chromatography (PE/EtOAc) to afford corresponding substituted coupling product.

Ocellatusone C analogue **17a**: synthesized from **11d** via procedure A using the corresponding boronate ester, affording 7.5 mg of a white solid in 92% yield. HRESIMS *m/z* 455.1822 $[M + H]^+$ (calcd. for $C_{27}H_{26}F_3O_3$, 455.1828); **$^1$H NMR** (600 MHz, $CDCl_3$) $\delta_H$ 7.71 – 7.64 (overlap, 4H), 7.52 (d, $J$ = 8.7 Hz, 2H), 7.06 (s, 2H), 5.78 (d, $J$ = 1.6 Hz, 1H), 3.98 (s, 3H), 3.82 (s, 1H), 1.92 (s, 3H), 1.48 (s, 3H), 1.45 (s, 3H), 0.91 (s, 3H); **$^{13}$C NMR** (151 MHz, $CDCl_3$) $\delta_C$ 207.2, 202.2, 178.6, 144.3, 139.1, 137.3, 135.9, 133.4, 127.4, 125.9 (d, $J$ = 3.7 Hz), 117.2, 61.9, 60.9, 60.6, 53.7, 21.8, 16.2, 15.7, 10.5; **$^{19}$F NMR** (565 MHz, $CDCl_3$) $\delta_F$ -62.4.

Ocellatusone C analogue **17b**: synthesized from **11d** via procedure A using the corresponding boronate ester, affording 6.3 mg of a white solid in 87% yield. HRESIMS *m/z* 405.1864 $[M + H]^+$ (calcd. for $C_{26}H_{26}FO_3$, 405.1860); **$^1$H NMR** (600 MHz, $CDCl_3$) $\delta_H$ 7.52 (dd, $J$ = 8.7, 5.4 Hz, 2H), 7.45 (d, $J$ = 8.7 Hz, 2H), 7.11 (t, $J$ = 8.7 Hz, 2H), 7.01 (s, 2H), 5.77 (d, $J$ = 1.5 Hz, 1H), 3.97 (s, 3H), 3.80 (s, 1H), 1.92 (s, 3H), 1.47 (d, $J$ = 1.4 Hz, 3H), 1.44 (s, 3H), 0.91 (s, 3H); **$^{13}$C NMR** (151 MHz, $CDCl_3$) $\delta_C$ 207.3, 202.3, 178.7, 162.6 (d, $J$ = 246.3 Hz), 139.6, 136.9 (d, $J$ = 3.2 Hz), 136.1 (d, $J$ = 14.1 Hz), 133.3, 128.7 (d, $J$ = 7.9 Hz), 127.1, 117.2, 115.8 (d, $J$ = 21.3 Hz), 61.9, 60.9, 60.6, 53.6, 21.8, 16.2, 15.7, 10.5; **$^{19}$F NMR** (565 MHz, $CDCl_3$) $\delta_F$ -115.7 (ddd, $J$ = 14.0, 8.9, 5.2 Hz).

Ocellatusone C analogue **17c**: synthesized from **11d** via procedure A using the corresponding boronate ester, affording 6.3 mg of a white solid in 84% yield. HRESIMS *m/z* 417.2066 $[M + H]^+$ (calcd. for $C_{27}H_{29}O_4$, 417.2060); **$^1$H NMR** (600 MHz, $CDCl_3$) $\delta_H$ 7.51 (d, $J$ = 8.7 Hz, 2H), 7.46 (d, $J$ = 8.7 Hz, 2H), 6.96 (overlap, 4H), 5.76 (s, 1H), 3.97 (s, 3H), 3.85 (s, 3H), 3.79 (s, 1H), 1.91 (s, 3H), 1.47 (s, 3H), 1.44 (s, 3H), 0.91 (s, 3H); **$^{13}$C NMR** (151 MHz, $CDCl_3$) $\delta_C$ 207.3, 202.4, 178.8, 159.3, 140.2, 136.2, 135.4, 133.3, 133.1, 128.2, 126.8, 117.2, 116.2, 115.0, 114.3, 61.9, 61.0, 60.7, 55.5, 53.6, 21.8, 16.2, 15.7, 10.5.

Ocellatusone C analogue **17d**: synthesized from **11d** via procedure A using the corresponding boronate ester, affording 6.8 mg of a white solid in 90% yield. HRESIMS *m/z* 419.1969 $[M + H]^+$ (calcd. for $C_{25}H_{27}N_2O_4$, 419.1965); **$^1$H NMR** (600 MHz, $CDCl_3$) $\delta_H$ 8.71 (s, 2H), 7.44 (d, $J$ = 8.7 Hz, 2H), 7.08 (s, 2H), 5.78 (d, $J$ = 1.5 Hz, 1H), 4.06 (s, 3H), 3.98 (s, 3H), 3.82 (s, 1H), 1.92 (s, 3H), 1.47 (d, $J$ = 1.4 Hz, 3H), 1.44 (s, 3H), 0.90 (s, 3H); **$^{13}$C NMR** (151 MHz, $CDCl_3$) $\delta_C$ 207.1, 202.1, 178.6, 165.2, 157.4, 137.3, 135.8, 133.9, 133.5, 127.9, 126.7, 117.2, 61.9, 60.8, 60.6, 55.2, 53.7, 21.8, 16.2, 15.7, 10.5.

Tetracyclo[3.3.1.0$^{2,8}$.0$^{3,7}$]nonane derivative **18a**: synthesized from **12** via procedure A using the corresponding boronate ester, affording 5.7 mg of a white solid in 82% yield. HRESIMS *m/z* 389.1863 $[M + H]^+$ (calcd. for $C_{24}H_{25}N_2O_3$, 389.1859); **$^1$H NMR** (600 MHz, $CDCl_3$) $\delta_H$ 9.21 (s, 1H), 8.96 (s, 2H), 7.56 (dd, $J$ = 18.0, 8.0 Hz, 2H), 7.34 (d, $J$ = 8.0 Hz, 1H), 7.17 (d, $J$ = 7.8 Hz, 1H), 4.17 (s, 1H), 3.53 (s, 3H), 2.62 (s, 1H), 1.38 (s, 3H), 1.31 (s, 3H), 0.92 (s, 3H), 0.72 (s, 3H); **$^{13}$C NMR** (151 MHz, $CDCl_3$) $\delta_C$ 214.9, 214.0, 157.7, 155.0, 138.5, 134.2, 133.9, 132.0, 127.6, 127.1, 127.0, 76.4, 72.5, 62.3, 59.0, 58.8, 49.4, 45.7, 28.4, 14.7, 11.9, 10.3, 7.0.

Tricyclo[4.2.1.0$^{2,8}$]nonane derivative **19a**: synthesized from **13** via procedure A using the corresponding boronate ester, affording 7.4 mg of a white solid in 90% yield. HRESIMS *m/z* 455.1831 $[M + H]^+$ (calcd. for $C_{27}H_{26}F_3O_3$, 455.1828); **$^1$H NMR** (600 MHz, $CDCl_3$) $\delta_H$ 7.68 (s, 4H), 7.52 (d, $J$ = 8.7 Hz, 2H), 7.22 (s, 1H), 6.92 (s, 1H), 5.71 (s, 1H), 3.62 (s, 3H), 3.11 (s, 1H), 1.52 (s, 3H), 1.47 (s, 3H), 1.42 (s, 3H), 1.02 (s, 3H); **$^{13}$C NMR** (151 MHz, $CDCl_3$) $\delta_C$ 209.0, 206.1,

144.3, 139.1, 137.1, 136.5, 129.4, 127.5, 125.8 (q, $J$ = 3.8 Hz), 87.9, 58.5, 58.1, 55.7, 54.2, 37.0, 25.1, 17.5, 16.4, 6.6; $^{19}$**F NMR** (565 MHz, $CDCl_3$) $\delta_F$ -62.4.

Tricyclo[4.2.1.0$^{2,8}$]nonane derivative **19b**: synthesized from **13** via procedure A using the corresponding boronate ester, affording 5.9 mg of a white solid in 81% yield. HRESIMS *m/z* 405.1862 $[M + H]^+$ (calcd. for $C_{26}H_{26}FO_3$, 405.1860); $^1$**H NMR** (600 MHz, $CDCl_3$) $\delta_H$ 7.54 (dd, $J$ = 8.6, 5.5 Hz, 2H), 7.45 (d, $J$ = 8.7 Hz, 2H), 7.17 (s, 1H), 7.11 (t, $J$ = 8.7 Hz, 2H), 6.88 (s, 1H), 5.69 (s, 1H), 3.62 (s, 3H), 3.09 (s, 1H), 1.51 (s, 3H), 1.47 (s, 3H), 1.42 (s, 3H), 1.01 (s, 3H); $^{13}$**C NMR** (151 MHz, $CDCl_3$) $\delta_C$ 209.1, 206.1, 139.6, 136.7, 136.0, 129.2, 128.8 (d, $J$ = 8.0 Hz), 115.7 (d, $J$ = 21.3 Hz), 87.9, 58.5, 58.1, 55.7, 54.2, 37.0, 25.2, 17.5, 16.4, 6.6; $^{19}$**F NMR** (565 MHz, $CDCl_3$) $\delta_F$ -104.76 – -128.26 (m).

Tricyclo[4.2.1.0$^{2,8}$]nonane derivative **19c**: synthesized from **13** via procedure A using the corresponding boronate ester, affording 6.4 mg of a white solid in 86% yield. HRESIMS *m/z* 417.2061 $[M + H]^+$ (calcd. for $C_{27}H_{29}O_4$, 417.2060); $^1$**H NMR** (600 MHz, $CDCl_3$) $\delta_H$ 7.52 (d, $J$ = 8.6 Hz, 2H), 7.46 (d, $J$ = 8.0 Hz, 2H), 7.15 (s, 1H), 6.96 (d, $J$ = 8.6 Hz, 2H), 6.84 (s, 1H), 5.69 (s, 1H), 3.85 (s, 3H), 3.62 (s, 3H), 3.08 (s, 1H), 1.51 (s, 3H), 1.47 (s, 3H), 1.41 (s, 3H), 1.02 (s, 3H); $^{13}$**C NMR** (151 MHz, $CDCl_3$) $\delta_C$ 209.3, 206.1, 159.3, 140.1, 136.8, 135.3, 133.4, 129.1, 128.2, 114.3, 87.9, 58.5, 58.1, 55.8, 55.5, 54.3, 37.0, 25.2, 17.6, 16.4, 6.6.

Tricyclo[4.2.1.0$^{2,8}$]nonane derivative **19d**: synthesized from **13** via procedure A using the corresponding boronate ester, affording 6.1 mg of a white solid in 88% yield. HRESIMS *m/z* 389.1867 $[M + H]^+$ (calcd. for $C_{24}H_{25}N_2O_3$, 389.1859); $^1$**H NMR** (600 MHz, $CDCl_3$) $\delta_H$ 9.20 (s, 1H), 8.95 (s, 2H), 7.51 (d, $J$ = 8.7 Hz, 2H), 7.26 (s, 1H), 6.98 (s, 1H), 5.72 (s, 1H), 3.62 (s, 3H), 3.13 (s, 1H), 1.52 (s, 3H), 1.47 (s, 3H), 1.42 (s, 3H), 1.01 (s, 3H); $^{13}$**C NMR** (151 MHz, $CDCl_3$) $\delta_C$ 208.8, 206.1, 157.7, 155.0, 138.2, 136.2, 134.0, 133.7, 129.7, 126.7, 87.9, 58.5, 58.2, 55.5, 54.2, 37.0, 25.1, 17.5, 16.4, 6.6.

Tricyclo[4.2.1.0$^{2,8}$]nonane derivative **20**: synthesized from **14** via procedure A using the corresponding boronate ester, affording 7.1 mg of a white solid in 88% yield. HRESIMS *m/z* 447.2163 $[M + H]^+$ (calcd. for $C_{28}H_{31}O_5$, 447.2166); $^1$**H NMR** (600 MHz, $CDCl_3$) $\delta_H$ 7.56 – 7.48 (m, 2H), 7.11 (dd, $J$ = 7.8, 2.0 Hz, 1H), 6.80 (dd, $J$ = 8.1, 2.0 Hz, 1H), 6.72 (d, $J$ = 2.2 Hz, 2H), 6.47 (t, $J$ = 2.3 Hz, 1H), 3.85 (s, 6H), 3.82 (s, 3H), 3.36 (s, 1H), 2.07 (s, 1H), 1.81 (s, 3H), 1.52 (s, 3H), 1.24 (s, 3H), 0.83 (s, 3H); $^{13}$**C NMR** (151 MHz, $CDCl_3$) $\delta_C$ 210.4, 192.5, 166.6, 161.2, 142.7, 140.2, 137.6, 131.6, 128.5, 127.4, 127.1, 116.8, 105.4, 99.6, 64.2, 57.2, 55.6, 52.1, 41.5, 41.3, 35.7, 18.4, 17.0, 11.3, 10.9.

Bicyclo[3.3.1]nonane derivative **21a**: synthesized from **15** via procedure A using the corresponding boronate ester, affording 7.1 mg of a white solid in 87% yield. HRESIMS *m/z* 455.1833 $[M + H]^+$ (calcd. for $C_{27}H_{26}F_3O_3$, 455.1828); $^1$**H NMR** (600 MHz, $CDCl_3$) $\delta_H$ 7.67 (q, $J$ = 8.5 Hz, 4H), 7.52 (dd, $J$ = 7.9, 2.1 Hz, 1H), 7.46 (dd, $J$ = 8.2, 2.1 Hz, 1H), 7.20 (dd, $J$ = 8.0, 2.1 Hz, 1H), 7.13 (dd, $J$ = 8.3, 2.0 Hz, 1H), 6.55 (s, 1H), 3.92 (s, 3H), 3.82 (d, $J$ = 1.8 Hz, 1H), 1.87 (d, $J$ = 1.5 Hz, 3H), 1.85 (s, 3H), 1.19 (s, 3H), 1.16 (s, 3H); $^{13}$**C NMR** (151 MHz, $CDCl_3$) $\delta_C$ 195.5, 194.6, 180.1, 148.1, 144.0, 139.1, 138.2, 134.0, 133.1, 129.6 (d, $J$ = 32.2 Hz), 127.7, 127.6, 127.4, 127.2, 125.9 (q, $J$ = 3.9 Hz), 112.9, 62.3, 62.0, 61.4, 44.6, 21.0, 18.8, 16.2, 10.5; $^{19}$**F NMR** (565 MHz, $CDCl_3$) $\delta_F$ -62.4.

Bicyclo[3.3.1]nonane derivative **21b**: synthesized from **15** via procedure A using the corresponding boronate ester, affording 5.8 mg of a white solid in 80% yield. HRESIMS *m/z* 405.1863 $[M + H]^+$ (calcd. for $C_{26}H_{26}FO_3$, 405.1860); **$^1$H NMR** (600 MHz, $CDCl_3$) $\delta_H$ 7.51 (dd, *J* = 8.7, 5.3 Hz, 2H), 7.46 (dd, *J* = 7.9, 2.2 Hz, 1H), 7.40 (dd, *J* = 8.3, 2.2 Hz, 1H), 7.15 (dd, *J* = 8.0, 2.1 Hz, 1H), 7.11 (t, *J* = 8.7 Hz, 2H), 7.08 (dd, *J* = 8.2, 2.1 Hz, 1H), 6.54 (t, *J* = 1.6 Hz, 1H), 3.92 (s, 3H), 3.80 (d, *J* = 1.8 Hz, 1H), 1.86 (d, *J* = 1.5 Hz, 3H), 1.84 (s, 3H), 1.19 (s, 3H), 1.16 (s, 3H); **$^{13}$C NMR** (151 MHz, $CDCl_3$) $\delta_C$ 195.6, 194.7, 180.2, 162.7 (d, *J* = 246.6 Hz), 148.1, 139.6, 137.1, 136.6 (d, *J* = 3.1 Hz), 133.8, 133.0, 128.6 (d, *J* = 7.9 Hz), 127.5, 127.2, 126.9, 115.8 (d, *J* = 21.6 Hz), 112.9, 62.3, 62.0, 61.5, 44.6, 21.0, 18.8, 16.2, 10.4; **$^{19}$F NMR** (565 MHz, $CDCl_3$) $\delta_F$ -115.5 (td, *J* = 8.6, 4.2 Hz).

Bicyclo[3.3.1]nonane derivative **21c**: synthesized from **15** via procedure A using the corresponding boronate ester, affording 6.4 mg of a white solid in 86% yield. HRESIMS *m/z* 417.2060 $[M + H]^+$ (calcd. for $C_{27}H_{29}O_4$, 417.2060); **$^1$H NMR** (600 MHz, $CDCl_3$) $\delta_H$ 7.50 (d, *J* = 8.7 Hz, 2H), 7.46 (dd, *J* = 7.9, 2.1 Hz, 1H), 7.41 (dd, *J* = 8.3, 2.1 Hz, 1H), 7.12 (dd, *J* = 8.0, 2.1 Hz, 1H), 7.05 (dd, *J* = 8.3, 2.1 Hz, 1H), 6.96 (d, *J* = 8.7 Hz, 2H), 6.54 (s, 1H), 3.91 (s, 3H), 3.85 (s, 3H), 3.78 (d, *J* = 1.8 Hz, 1H), 1.86 (d, *J* = 1.5 Hz, 3H), 1.84 (s, 3H), 1.19 (s, 3H), 1.16 (s, 3H); **$^{13}$C NMR** (151 MHz, $CDCl_3$) $\delta_C$ 195.7, 194.8, 180.2, 159.4, 148.2, 140.2, 136.4, 133.8, 133.0, 133.0, 128.1, 127.4, 126.9, 126.5, 114.4, 112.9, 62.4, 62.0, 61.5, 55.5, 44.6, 21.0, 18.8, 16.2, 10.4.

Bicyclo[3.3.1]nonane derivative **21d**: synthesized from **15** via procedure A using the corresponding boronic acid, affording 6.4 mg of a white solid in 88% yield. HRESIMS *m/z* 403.1902 $[M + H]^+$ (calcd. for $C_{26}H_{27}O_4$, 403.19039); **$^1$H NMR** (600 MHz, $CDCl_3$) $\delta_H$ 7.49 (dd, *J* = 7.9, 2.1 Hz, 1H), 7.43 (dd, *J* = 8.3, 2.1 Hz, 1H), 7.29 (t, *J* = 7.9 Hz, 1H), 7.16 – 7.10 (m, 2H), 7.07 (dd, *J* = 8.3, 2.2 Hz, 1H), 7.04 (t, *J* = 2.1 Hz, 1H), 6.82 (dd, *J* = 8.0, 2.3 Hz, 1H), 6.55 (s, 1H), 5.16 (s, 1H), 3.92 (s, 3H), 3.79 (d, *J* = 1.7 Hz, 1H), 1.86 (d, *J* = 1.5 Hz, 3H), 1.84 (s, 3H), 1.19 (s, 3H), 1.16 (s, 3H); **$^{13}$C NMR** (151 MHz, $CDCl_3$) $\delta_C$ 195.8, 194.8, 180.4, 156.1, 148.2, 142.2, 140.1, 137.3, 133.8, 133.0, 130.2, 127.4, 127.4, 127.0, 119.6, 114.5, 114.0, 112.8, 62.3, 62.0, 61.5, 44.6, 21.0, 18.8, 16.2, 10.5.

**Procedure B for Miyaura Borylation Coupling:**
To an oven-dried 10 mL glass tube equipped with a stir bar was added the polycyclic compound **11d** (7.0 mg, 0.018 mmol, 1.0 eq.), $B_2pin_2$ (5.5 mg, 0.022 mmol, 1.2 eq.), KOAc (3.5 mg, 0.036 mmol, 2.0 eq.), $PPh_3$ (0.9 mg, 0.004 mmol, 0.2 eq.), $PdCl_2(PPh_3)_2$ (1.3 mg, 0.0018 mmol, 10 mol%) in dry toluene (0.5 mL). The reaction vessel was sealed with a Teflon screw cap, evacuated and back-filled with $N_2$ (three cycles), and the mixture was heated to 80 °C in an oil bath with stirring for 12 hours. Upon completion of the reaction, the mixture was diluted with $H_2O$ (5 mL) and extracted with EtOAc (2 × 10 mL). The combined organic layers were dried over $Na_2SO_4$, filtered and concentrated under reduced pressure. The resulting crude residue was purified by flash chromatography (10% EtOAc in PE) to afford corresponding substituted coupling product.

Ocellatusone C analogue **17e**: synthesized from **11d** via procedure B, affording 6.4 mg of a brown solid in 82% yield. HRESIMS *m/z* 437.2501 $[M + H]^+$ (calcd. for $C_{26}H_{34}BO_5$, 437.2493); **$^1$H NMR** (600 MHz, $CDCl_3$) $\delta_H$ 7.72 (d, *J* = 8.2 Hz, 2H), 6.96 (s, 2H), 5.74 (d, *J* = 1.5 Hz, 1H), 3.95 (s, 3H), 3.76 (s, 1H), 1.90 (s, 3H), 1.42 (overlap, 6H), 1.33 (overlap, 12H), 0.84 (s, 3H); **$^{13}$C NMR** (151

MHz, $CDCl_3$) $\delta_C$ 207.0, 202.3, 178.8, 140.3, 136.0, 135.0, 133.2, 117.3, 83.9, 61.8, 61.0, 60.8, 53.5, 25.1, 24.9, 21.7, 16.1, 15.7, 10.5.

**General Procedure C for Buchwald–Hartwig Coupling:**
To an oven-dried 10 mL glass tube equipped with a stir bar was added the polycyclic compound (7.0 mg, 0.018 mmol, 1.0 eq.), morpholine (2.4 µL, 0.027 mmol, 1.5 eq.), $Cs_2CO_3$ (8.8 mg, 0.027 mmol, 1.5 eq.), Xantphos (2.1 mg, 0.004 mmol, 0.2 eq.), $Pd_2dba_3$ (1.6 mg, 0.0018 mmol, 10 mol%) in dry dioxane (0.5 mL). The reaction vessel was sealed with a Teflon screw cap, evacuated and back-filled with $N_2$ (three cycles), and the mixture was heated to 80 °C in an oil bath with stirring for 12 hours. Upon completion of the reaction, the mixture was diluted with $H_2O$ (5 mL) and extracted with EtOAc (2 × 10 mL). The combined organic layers were dried over $Na_2SO_4$, filtered and concentrated under reduced pressure. The resulting crude residue was purified by flash chromatography (PE/EtOAc) to afford corresponding substituted coupling product.

Ocellatusone C analogue **17f**: synthesized from **11d** via procedure C, affording 1.8 mg of a yellow oil in 25% yield. HRESIMS *m/z* 396.2177 $[M + H]^+$ (calcd. for $C_{24}H_{30}NO_4$, 396.2169); **$^1$H NMR** (600 MHz, $CDCl_3$) $\delta_H$ 6.88 (s, 4H), 5.71 (s, 1H), 3.95 (s, 3H), 3.89 (s, 4H), 3.69 (s, 1H), 3.17 (s, 4H), 1.90 (s, 3H), 1.43 (s, 3H), 1.41 (s, 3H), 0.86 (s, 3H); **$^{13}$C NMR** (151 MHz, $CDCl_3$) $\delta_C$ 207.4, 202.4, 178.7, 136.3, 132.9, 117.2, 66.8, 61.8, 61.2, 60.3, 53.6, 49.4, 21.7, 16.1, 15.7, 10.5.

Tetracyclo[3.3.1.$0^{2,8}$.$0^{3,7}$]nonane derivative **18b**: synthesized from **12** via procedure C, affording 1.6 mg of a yellow oil in 23% yield. HRESIMS *m/z* 396.2173 $[M + H]^+$ (calcd. for $C_{24}H_{30}NO_4$, 396.2169); **$^1$H NMR** (600 MHz, $CDCl_3$) $\delta_H$ 7.07 (d, $J$ = 8.0 Hz, 1H), 6.91 (d, $J$ = 8.4 Hz, 1H), 6.87 (d, $J$ = 8.2 Hz, 1H), 6.81 (d, $J$ = 8.4 Hz, 1H), 4.03 (s, 1H), 3.88 – 3.83 (m, 4H), 3.50 (s, 3H), 3.16 (dd, $J$ = 6.1, 3.8 Hz, 4H), 2.55 (d, $J$ = 0.9 Hz, 1H), 1.35 (s, 3H), 1.28 (s, 3H), 0.88 (s, 3H), 0.69 (s, 3H); **$^{13}$C NMR** (151 MHz, $CDCl_3$) $\delta_C$ 215.1, 214.6, 131.8, 130.7, 128.7, 126.6, 115.9, 114.8, 76.2, 72.8, 67.1, 63.2, 58.8, 58.7, 49.2, 49.1, 45.8, 28.6, 14.7, 11.9, 10.2, 7.0.

## Part 7. X-ray Crystallographic Data for Compounds 11a, 11d, 11t, 12, 13, 14, 15, and 16

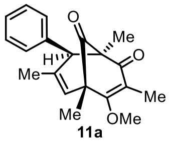


**11a**
CCDC: 2504235

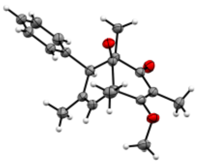

| | |
|---|---|
| Identification code | 1 |
| Empirical formula | $C_{20}H_{22}O_3$ |
| Formula weight | 310.37 |
| Temperature/K | 170.00 |
| Crystal system | monoclinic |
| Space group | $P2_1/n$ |
| a/Å | 9.3708(3) |
| b/Å | 12.4551(4) |
| c/Å | 14.1907(5) |

| | |
|---|---|
| α/° | 90 |
| β/° | 90.524(2) |
| γ/° | 90 |
| Volume/$Å^3$ | 1656.19(10) |
| Z | 4 |
| $\rho_{calc}$g/$cm^3$ | 1.245 |
| μ/$mm^{-1}$ | 0.658 |
| F(000) | 664.0 |
| Crystal size/$mm^3$ | 0.08 × 0.06 × 0.03 |
| Radiation | CuKα (λ = 1.54178) |
| 2Θ range for data collection/° | 9.448 to 149.804 |
| Index ranges | -10 ≤ h ≤ 11, -15 ≤ k ≤ 15, -16 ≤ l ≤ 17 |
| Reflections collected | 12062 |
| Independent reflections | 3363 [$R_{int}$ = 0.0518, $R_{sigma}$ = 0.0411] |
| Data/restraints/parameters | 3363/0/213 |
| Goodness-of-fit on $F^2$ | 1.065 |
| Final R indexes [I>=2σ (I)] | $R_1$ = 0.0427, $wR_2$ = 0.1181 |
| Final R indexes [all data] | $R_1$ = 0.0558, $wR_2$ = 0.1272 |
| Largest diff. peak/hole / e $Å^{-3}$ | 0.24/-0.21 |

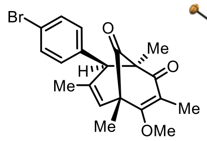

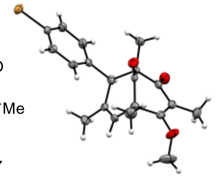

**11d**
CCDC: 2504237

| | |
|---|---|
| Identification code | cu_20241231A_0m |
| Empirical formula | $C_{20}H_{21}BrO_3$ |
| Formula weight | 389.28 |
| Temperature/K | 170.00 |
| Crystal system | triclinic |
| Space group | P-1 |
| a/Å | 8.8555(2) |
| b/Å | 10.1050(2) |
| c/Å | 11.5692(3) |
| α/° | 106.127(2) |
| β/° | 92.716(2) |
| γ/° | 115.363(2) |
| Volume/$Å^3$ | 882.01(4) |
| Z | 2 |
| $\rho_{calc}$g/$cm^3$ | 1.466 |
| μ/$mm^{-1}$ | 3.293 |
| F(000) | 400.0 |

| | |
|---|---|
| Crystal size/mm$^3$ | 0.13 × 0.05 × 0.03 |
| Radiation | CuKα (λ = 1.54178) |
| 2Θ range for data collection/° | 8.106 to 127.782 |
| Index ranges | -8 ≤ h ≤ 10, -11 ≤ k ≤ 9, -13 ≤ l ≤ 13 |
| Reflections collected | 6843 |
| Independent reflections | 2841 [$R_{int}$ = 0.0340, $R_{sigma}$ = 0.0391] |
| Data/restraints/parameters | 2841/0/222 |
| Goodness-of-fit on $F^2$ | 1.082 |
| Final R indexes [I>=2σ (I)] | $R_1$ = 0.0314, $wR_2$ = 0.0753 |
| Final R indexes [all data] | $R_1$ = 0.0352, $wR_2$ = 0.0785 |
| Largest diff. peak/hole / e Å$^{-3}$ | 0.34/-0.42 |

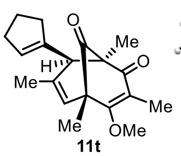

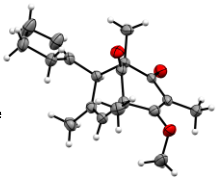

**11t**
CCDC: 2504239

| | |
|---|---|
| Identification code | XB42-2 |
| Empirical formula | $C_{19}H_{24}O_3$ |
| Formula weight | 300.38 |
| Temperature/K | 170(2) |
| Crystal system | monoclinic |
| Space group | Pn |
| a/Å | 12.6071(6) |
| b/Å | 8.8397(5) |
| c/Å | 15.5391(6) |
| α/° | 90 |
| β/° | 109.124(3) |
| γ/° | 90 |
| Volume/Å$^3$ | 1636.15(14) |
| Z | 4 |
| $\rho_{calc}$g/cm$^3$ | 1.219 |
| μ/mm$^{-1}$ | 0.644 |
| F(000) | 648.0 |
| Crystal size/mm$^3$ | 0.12 × 0.09 × 0.01 |
| Radiation | CuKα (λ = 1.54178) |
| 2Θ range for data collection/° | 7.878 to 137.944 |
| Index ranges | -14 ≤ h ≤ 15, -10 ≤ k ≤ 8, -18 ≤ l ≤ 15 |
| Reflections collected | 8257 |
| Independent reflections | 4354 [$R_{int}$ = 0.0501, $R_{sigma}$ = 0.0653] |
| Data/restraints/parameters | 4354/2/407 |
| Goodness-of-fit on $F^2$ | 1.079 |

| | |
|---|---|
| Final R indexes [I>=2σ (I)] | $R_1$ = 0.0508, $wR_2$ = 0.1213 |
| Final R indexes [all data] | $R_1$ = 0.0662, $wR_2$ = 0.1375 |
| Largest diff. peak/hole / e $Å^{-3}$ | 0.26/-0.22 |
| Flack parameter | -0.2(3) |

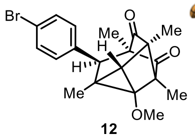

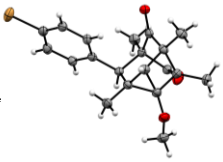

**12**
CCDC: 2504240

| | |
|---|---|
| Identification code | cu_20250108B_0m |
| Empirical formula | $C_{20}H_{21}BrO_3$ |
| Formula weight | 389.28 |
| Temperature/K | 300.00 |
| Crystal system | monoclinic |
| Space group | $P2_1/c$ |
| a/Å | 10.2678(7) |
| b/Å | 12.0632(8) |
| c/Å | 29.850(2) |
| α/° | 90 |
| β/° | 98.883(5) |
| γ/° | 90 |
| Volume/$Å^3$ | 3652.9(4) |
| Z | 8 |
| $\rho_{calc}$g/$cm^3$ | 1.416 |
| μ/$mm^{-1}$ | 3.181 |
| F(000) | 1600.0 |
| Crystal size/$mm^3$ | 0.11 × 0.05 × 0.01 |
| Radiation | CuKα (λ = 1.54178) |
| 2Θ range for data collection/° | 5.994 to 128.348 |
| Index ranges | -11 ≤ h ≤ 11, -14 ≤ k ≤ 11, -34 ≤ l ≤ 34 |
| Reflections collected | 21309 |
| Independent reflections | 5999 [$R_{int}$ = 0.0820, $R_{sigma}$ = 0.0624] |
| Data/restraints/parameters | 5999/0/443 |
| Goodness-of-fit on $F^2$ | 1.072 |
| Final R indexes [I>=2σ (I)] | $R_1$ = 0.0572, $wR_2$ = 0.1565 |
| Final R indexes [all data] | $R_1$ = 0.0887, $wR_2$ = 0.1903 |
| Largest diff. peak/hole / e $Å^{-3}$ | 0.50/-0.79 |

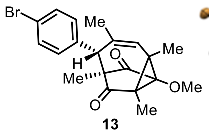

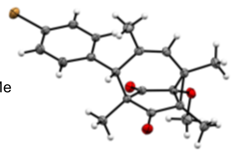

**13**
CCDC: 2504241

| | |
|---|---|
| Identification code | cu_20250211A_0m |
| Empirical formula | $C_{20}H_{21}BrO_3$ |
| Formula weight | 389.291 |
| Temperature/K | 295.00 |
| Crystal system | orthorhombic |
| Space group | Pbca |
| a/Å | 10.3236(5) |
| b/Å | 14.3853(8) |
| c/Å | 24.7888(13) |
| α/° | 90 |
| β/° | 90 |
| γ/° | 90 |
| Volume/Å$^3$ | 3681.3(3) |
| Z | 8 |
| $\rho_{calc}$g/cm$^3$ | 1.405 |
| μ/mm$^{-1}$ | 3.156 |
| F(000) | 1599.1 |
| Crystal size/mm$^3$ | 0.09 × 0.08 × 0.02 |
| Radiation | Cu Kα (λ = 1.54178) |
| 2Θ range for data collection/° | 7.14 to 128 |
| Index ranges | -8 ≤ h ≤ 12, -16 ≤ k ≤ 16, -28 ≤ l ≤ 28 |
| Reflections collected | 25493 |
| Independent reflections | 3048 [$R_{int}$ = 0.0719, $R_{sigma}$ = 0.0386] |
| Data/restraints/parameters | 3048/0/222 |
| Goodness-of-fit on F$^2$ | 1.050 |
| Final R indexes [I>=2σ (I)] | $R_1$ = 0.0500, $wR_2$ = 0.1245 |
| Final R indexes [all data] | $R_1$ = 0.0796, $wR_2$ = 0.1464 |
| Largest diff. peak/hole / e Å$^{-3}$ | 0.86/-0.97 |

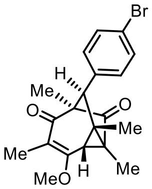


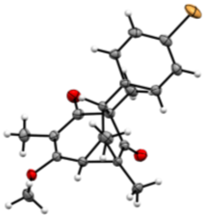

**14**
CCDC: 2504242

| | |
|---|---|
| Identification code | cu_20250109B_0m |
| Empirical formula | $C_{20}H_{21}BrO_3$ |
| Formula weight | 389.28 |
| Temperature/K | 295.00 |
| Crystal system | monoclinic |
| Space group | $P2_1/c$ |
| a/Å | 8.1340(18) |

| | |
|---|---|
| b/Å | 14.579(3) |
| c/Å | 30.759(8) |
| α/° | 90 |
| β/° | 91.220(9) |
| γ/° | 90 |
| Volume/$Å^3$ | 3646.7(14) |
| Z | 8 |
| $\rho_{calc}g/cm^3$ | 1.418 |
| $\mu/mm^{-1}$ | 3.186 |
| F(000) | 1600.0 |
| Crystal size/$mm^3$ | 0.2 × 0.02 × 0.02 |
| Radiation | CuKα (λ = 1.54178) |
| 2Θ range for data collection/° | 5.748 to 127.758 |
| Index ranges | -9 ≤ h ≤ 7, -16 ≤ k ≤ 16, -31 ≤ l ≤ 35 |
| Reflections collected | 20919 |
| Independent reflections | 5996 [$R_{int}$ = 0.0706, $R_{sigma}$ = 0.0588] |
| Data/restraints/parameters | 5996/0/443 |
| Goodness-of-fit on $F^2$ | 1.033 |
| Final R indexes [I>=2σ (I)] | $R_1$ = 0.0532, $wR_2$ = 0.1236 |
| Final R indexes [all data] | $R_1$ = 0.0932, $wR_2$ = 0.1479 |
| Largest diff. peak/hole / e $Å^{-3}$ | 0.66/-0.83 |

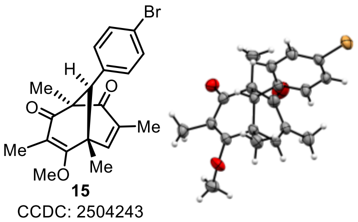


**15**

CCDC: 2504243

| | |
|---|---|
| Identification code | Po |
| Empirical formula | $C_{20}H_{21}BrO_3$ |
| Formula weight | 389.28 |
| Temperature/K | 295(2) |
| Crystal system | triclinic |
| Space group | P-1 |
| a/Å | 12.3554(12) |
| b/Å | 12.3742(13) |
| c/Å | 13.0938(11) |
| α/° | 112.524(5) |
| β/° | 91.962(5) |
| γ/° | 100.649(6) |
| Volume/$Å^3$ | 1805.1(3) |
| Z | 4 |

| | |
|---|---|
| $\rho_{calc}$g/cm$^3$ | 1.432 |
| μ/mm$^{-1}$ | 3.218 |
| F(000) | 800.0 |
| Crystal size/mm$^3$ | 0.08 × 0.05 × 0.04 |
| Radiation | CuKα (λ = 1.54178) |
| 2Θ range for data collection/° | 7.33 to 128.24 |
| Index ranges | -14 ≤ h ≤ 14, -14 ≤ k ≤ 14, -15 ≤ l ≤ 15 |
| Reflections collected | 26615 |
| Independent reflections | 5995 [$R_{int}$ = 0.0504, $R_{sigma}$ = 0.0367] |
| Data/restraints/parameters | 5995/0/441 |
| Goodness-of-fit on F$^2$ | 1.063 |
| Final R indexes [I>=2σ (I)] | $R_1$ = 0.0469, w$R_2$ = 0.1309 |
| Final R indexes [all data] | $R_1$ = 0.0614, w$R_2$ = 0.1446 |
| Largest diff. peak/hole / e Å$^{-3}$ | 0.41/-0.47 |

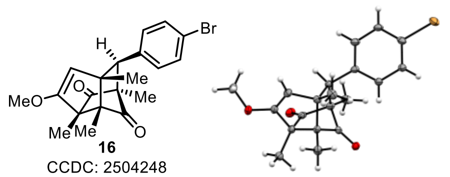

16
CCDC: 2504248

| | |
|---|---|
| Identification code | XB423-1 |
| Empirical formula | $C_{20}H_{21}BrO_3$ |
| Formula weight | 389.28 |
| Temperature/K | 170(2) |
| Crystal system | triclinic |
| Space group | P-1 |
| a/Å | 12.3388(5) |
| b/Å | 12.5950(5) |
| c/Å | 13.9950(6) |
| α/° | 86.031(2) |
| β/° | 72.815(2) |
| γ/° | 61.618(2) |
| Volume/Å$^3$ | 1821.49(13) |
| Z | 4 |
| $\rho_{calc}$g/cm$^3$ | 1.420 |
| μ/mm$^{-1}$ | 3.189 |
| F(000) | 800.0 |
| Crystal size/mm$^3$ | 0.06 × 0.05 × 0.04 |
| Radiation | CuKα (λ = 1.54178) |
| 2Θ range for data collection/° | 6.634 to 136.75 |
| Index ranges | -14 ≤ h ≤ 14, -15 ≤ k ≤ 15, -16 ≤ l ≤ 16 |
| Reflections collected | 18026 |
| Independent reflections | 6585 [$R_{int}$ = 0.0501, $R_{sigma}$ = 0.0516] |

| | |
|---|---|
| Data/restraints/parameters | 6585/0/443 |
| Goodness-of-fit on $F^2$ | 1.076 |
| Final R indexes [I>=2σ (I)] | $R_1 = 0.0555$, $wR_2 = 0.1500$ |
| Final R indexes [all data] | $R_1 = 0.0681$, $wR_2 = 0.1590$ |
| Largest diff. peak/hole / e Å$^{-3}$ | 1.95/-1.16 |

## Part 8. Conversion of 1-1 to Ocellatusone C (1)

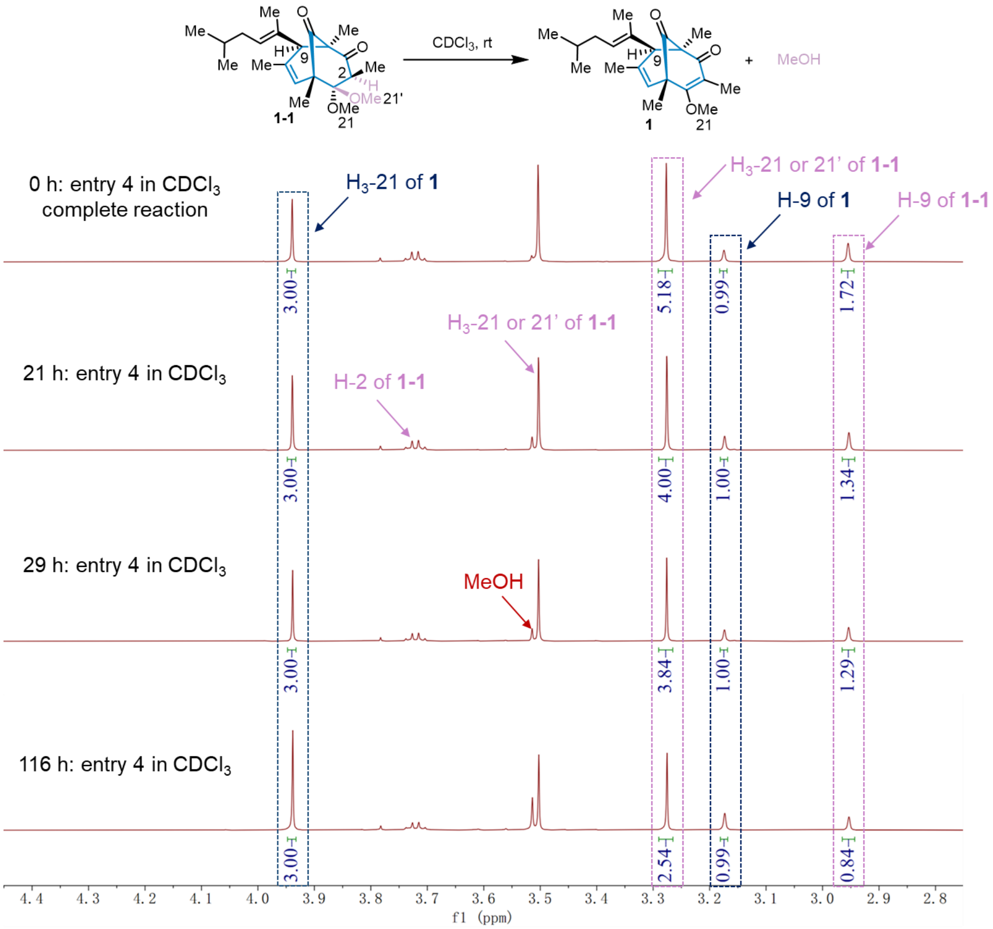


**Fig. S2.** Transformation of compound **1-1** to ocellatusone C (**1**) and methanol in $CDCl_3$. Entry 4 refers to the conditions listed in Table S1, entry 4.

## Part 9. Natural Product Spectral Comparisons

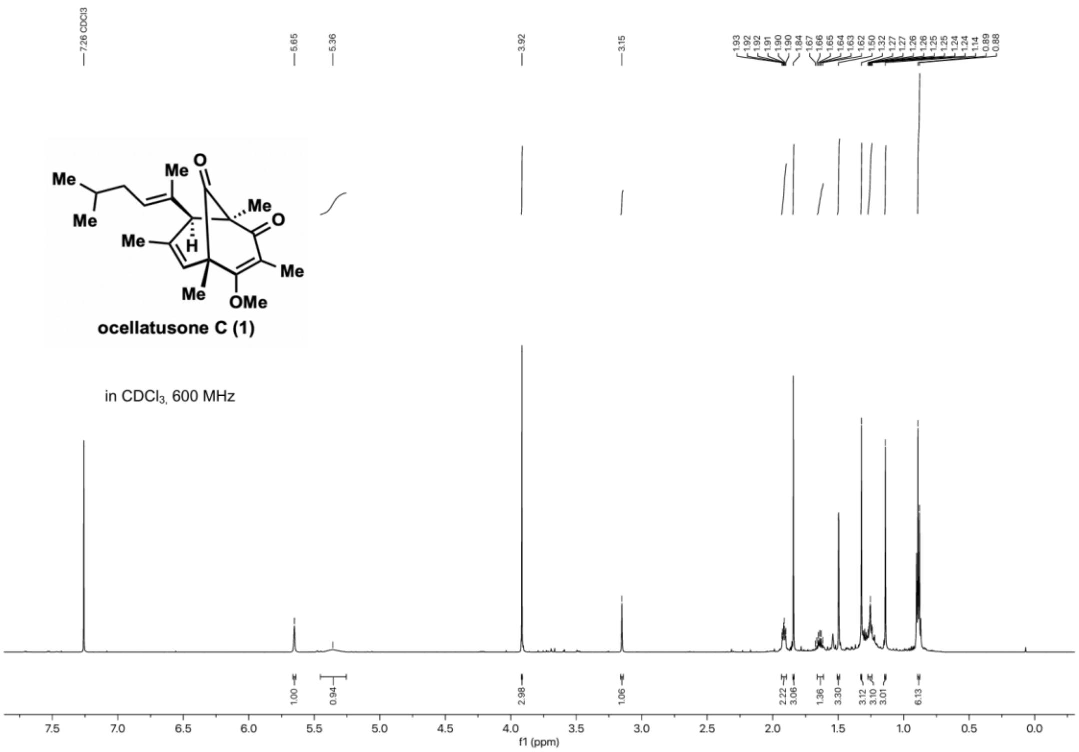


**Fig. S3.** $^{1}$H NMR spectrum of synthetic ocellatusone C (**1**) (Maimone's synthesis).

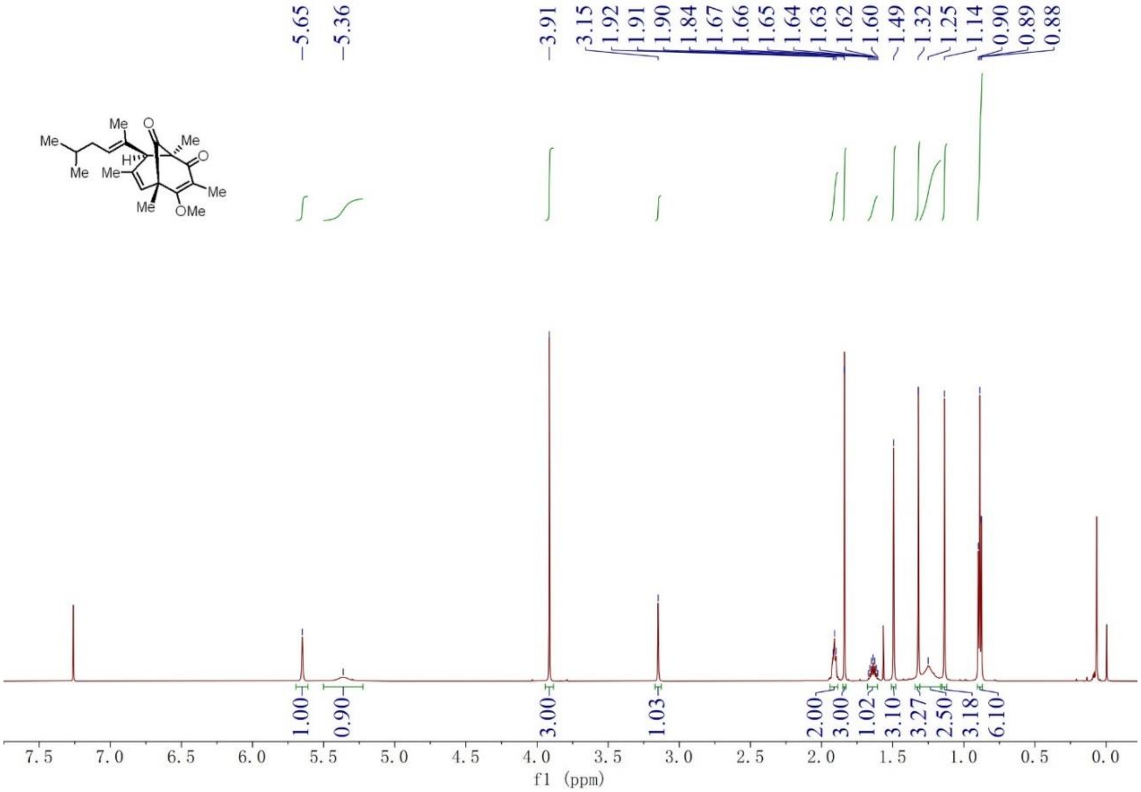


**Fig. S4.** $^{1}$H NMR spectrum of synthetic ocellatusone C (**1**) (Our synthesis).

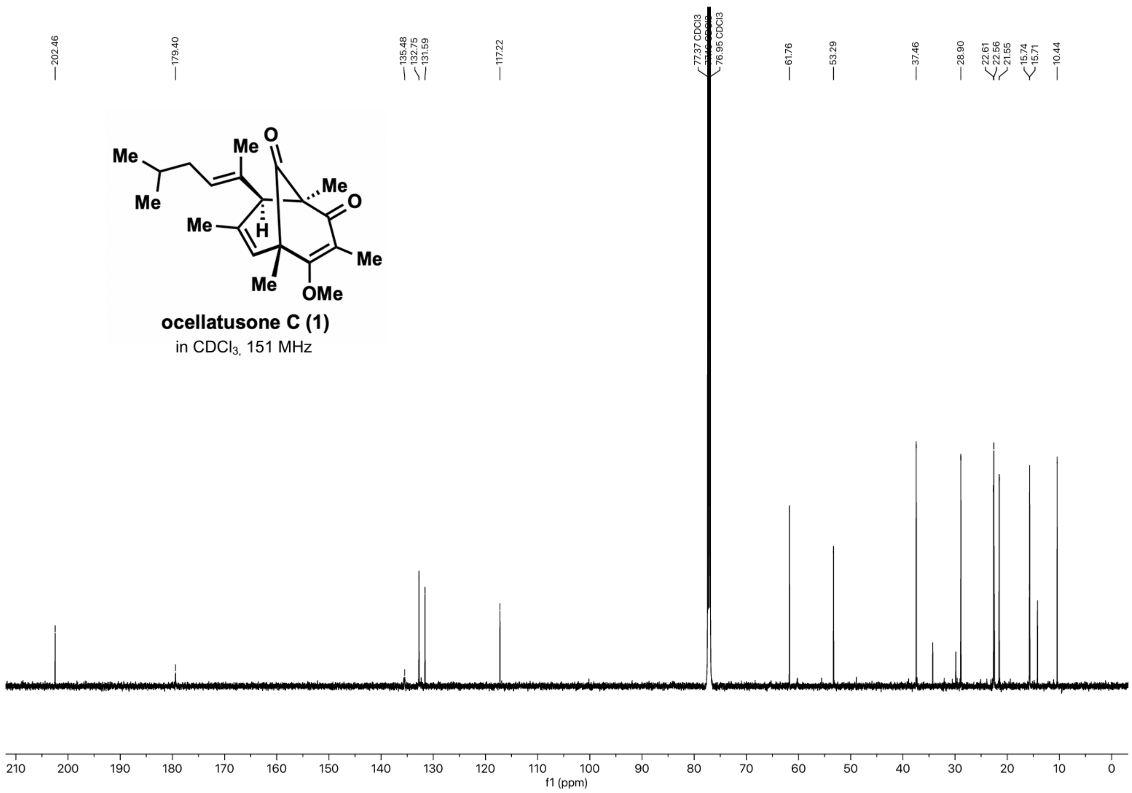


**Fig. S5.** $^{13}$C NMR spectrum of synthetic ocellatusone C (**1**) (Maimone's synthesis).

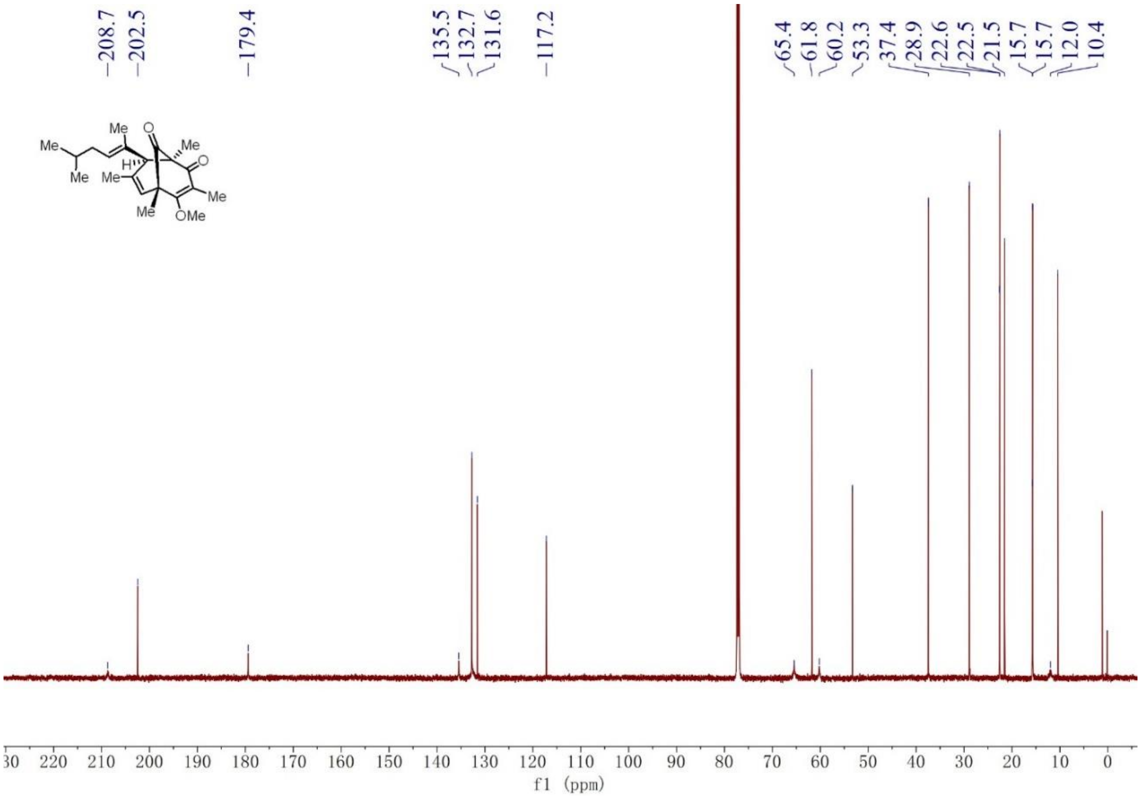


**Fig. S6.** $^{13}$C NMR spectrum of synthetic ocellatusone C (**1**) (Our synthesis).

**Table S3.** Comparison of $^{1}H$ and $^{13}C$ NMR data for ocellatusone C (**1**) from this work, Maimone's synthesis *(24)*, and natural isolate *(23)*.

| This report ($^{1}H$ NMR) 600 MHz | Synthesized (Maimone) ($^{1}H$ NMR) 600 MHz | Natural ($^{1}H$ NMR) 600 MHz | This report ($^{13}C$ NMR) 151 MHz | Synthesized (Maimone) ($^{13}C$ NMR) 151 MHz | Natural ($^{13}C$ NMR) 150 MHz |
|---|---|---|---|---|---|
| - | - | - | 179.4 | 179.4 | 179.4 |
| - | - | - | 117.2 | 117.2 | 117.2 |
| - | - | - | 202.5 | 202.5 | 202.5 |
| - | - | - | 60.2 | 60.1* | 60.1* |
| - | - | - | 208.7 | 208.5* | 208.7* |
| - | - | - | 53.3 | 53.3 | 53.3 |
| 5.65 s | 5.65 s | 5.65 s | 132.7 | 132.8 | 132.8 |
| - | - | - | 135.5 | 135.4* | 135.4* |
| 3.15 s | 3.15 s | 3.15 s | 65.4 | 65.2* | 65.2* |
| - | - | - | 131.6 | 131.6 | 132.0 |
| 5.36 brs | 5.36 brs | 5.38 brs | 135.5 | 135.5 | 135.4* |
| 1.91 m | 1.91 m | 1.91 m | 37.4 | 37.5 | 37.4 |
| 1.64 hept (6.6) | 1.65 m | 1.64 m | 28.9 | 28.9 | 28.9 |
| 0.89 d (6.7) | 0.88 d (6.7) | 0.88 d (6.8) | 22.6 | 22.6 | 22.6 |
| 0.88 d (6.3) | 0.88 d (6.7) | 0.89 d (6.8) | 22.5 | 22.6 | 22.5 |
| 1.84 s | 1.84 s | 1.84 s | 10.4 | 10.4 | 10.4 |
| 1.14 s | 1.14 s | 1.14 s | 15.7 | 15.7 | 15.7 |
| 1.32 s | 1.32 s | 1.32 s | 15.7 | 15.7 | 15.7 |
| 1.49 s | 1.50 s | 1.50 s | 21.5 | 21.6 | 21.6 |
| 1.25 brs | 1.26 m | 1.25 brs | 12.0 | 11.8* | 12.4* |
| 3.91 s | 3.92 s | 3.92 s | 61.8 | 61.8 | 61.8 |

*These resonances were deduced via relevant 2D experiments *(23, 24)*.

## Part 10. Proposed Pathway and DFT Calculations for Ocellatusone C (1)

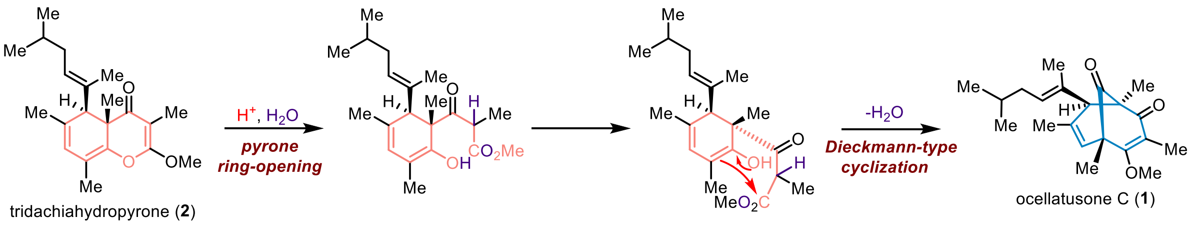


**Fig. S7.** Proposed biosynthetic pathway from tridachiahydropyrone (**2**) to ocellatusone C (**1**).

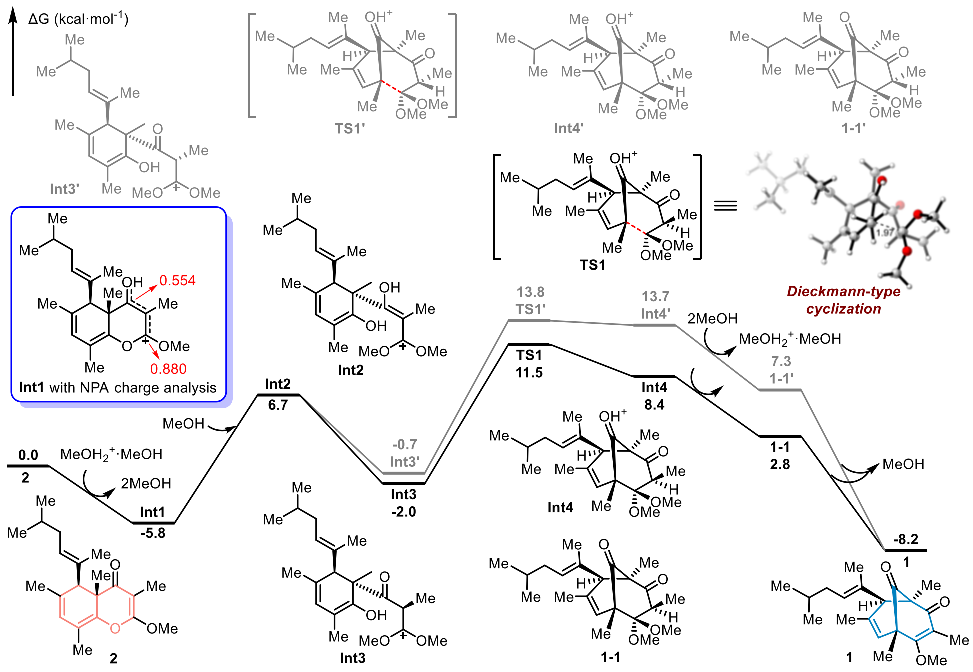

**Fig. S8.** DFT calculated free energy surface for biomimetic synthesis of ocellatusone C (**1**) from tridachiahydropyrone (**2**). Geometry optimized with B3LYP-D3BJ/Def2-SVP in SMD (methanol). Free energy calculated with SMD-B3LYP-D3BJ/Def2-TZVP//SMD-B3LYP-D3BJ/Def2-SVP. NPA charges *(61)* were then computed at SMD-B3LYP-D3BJ/Def2-SVP level of theory on the optimized geometries. Molecular graphics have been produced with CYLview. The red values represent the NPA charges for the relevant atoms. Bond distance in Å. Energy in kcal·mol$^{-1}$.

## Part 11. Studies on Compounds 10v, 10l, and 11l

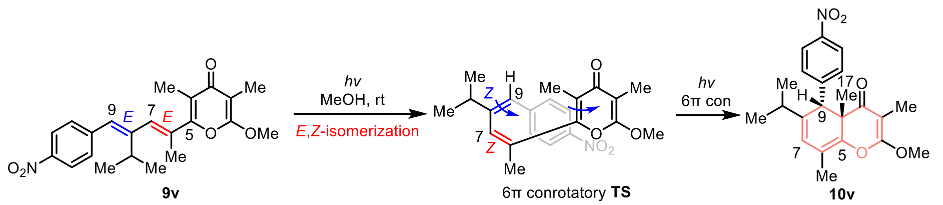

**Fig. S9.** Proposed mechanism for the photochemical formation of **10v**. The natural product tridachiahydropyrone (**2**) is known to form from polyene precursor **7** via photoinduced *E*/*Z* isomerization at C6–C7 double bond followed by 6π conrotatory electrocyclization *(29)*. We propose that **10v** is generated through an additional, secondary photoinduced *E*/*Z* isomerization at C8–C9 double bond, providing a (*Z*,*Z*)-configured intermediate that undergoes 6π conrotatory cyclization.

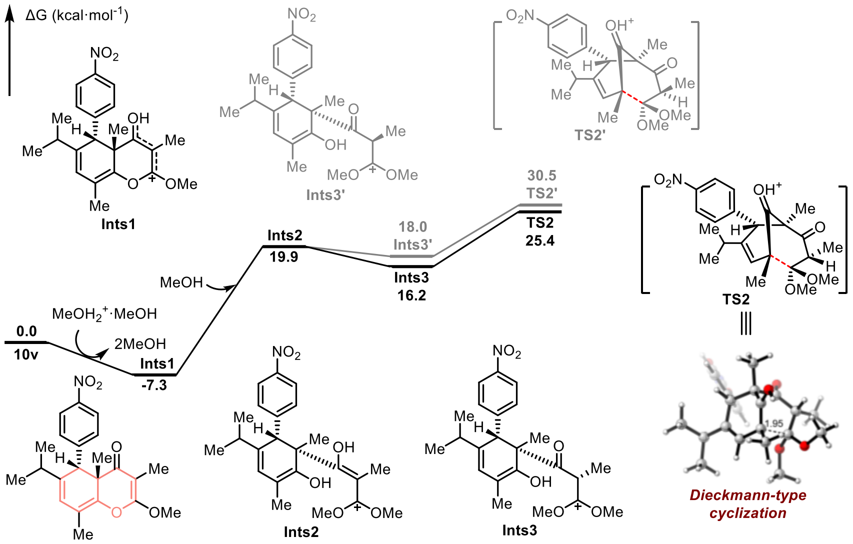


**Fig. S10.** DFT calculated free energy surface for biomimetic synthesis of **11v** from **10v**. Geometry optimized with B3LYP-D3BJ/Def2-SVP in SMD (methanol). Free energy calculated with SMD-B3LYP-D3BJ/Def2-TZVP//SMD-B3LYP-D3BJ/Def2-SVP. Molecular graphics have been produced with CYLview. Bond distance in Å. Energy in kcal·mol$^{-1}$.

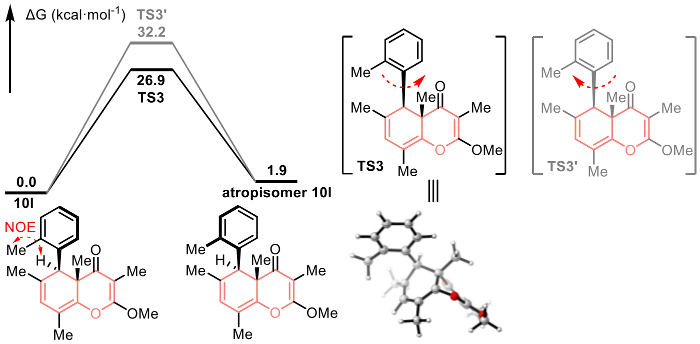


**Fig. S11.** DFT calculated free energy surface for the conversion of **10l** to atropisomer **10l**. Geometry optimized with B3LYP-D3BJ/Def2-SVP in SMD (methanol). Free energy calculated with SMD-B3LYP-D3BJ/Def2-TZVP//SMD-B3LYP-D3BJ/Def2-SVP. Molecular graphics have been produced with CYLview. Energy in kcal·mol$^{-1}$.

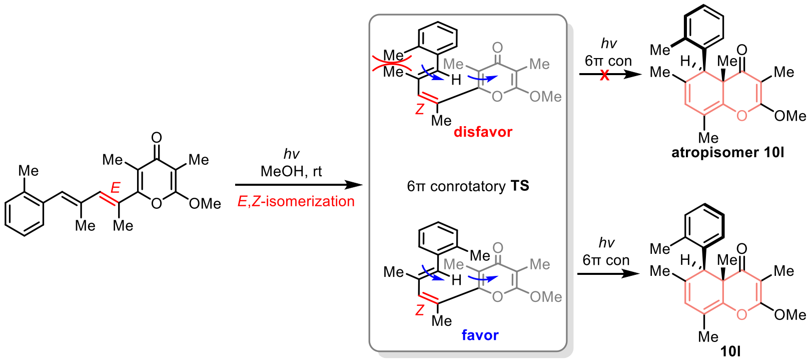


**Fig. S12.** Proposed mechanism for the selective photochemical formation of **10l**.

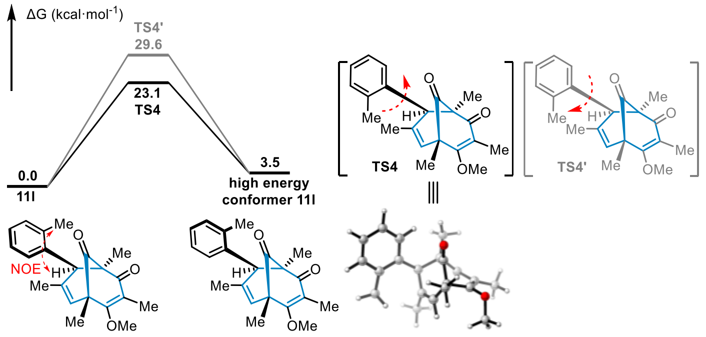


**Fig. S13.** DFT calculated free energy surface for the conversion of **11l** to high energy conformer **11l**. Geometry optimized with B3LYP-D3BJ/Def2-SVP in SMD (methanol). Free energy calculated with SMD-B3LYP-D3BJ/Def2-TZVP//SMD-B3LYP-D3BJ/Def2-SVP. Molecular graphics have been produced with CYLview. Energy in kcal·mol$^{-1}$.

## Part 12. NMR Evidence for Restricted Aryl Rotation in 10 and 11 Series Analogues

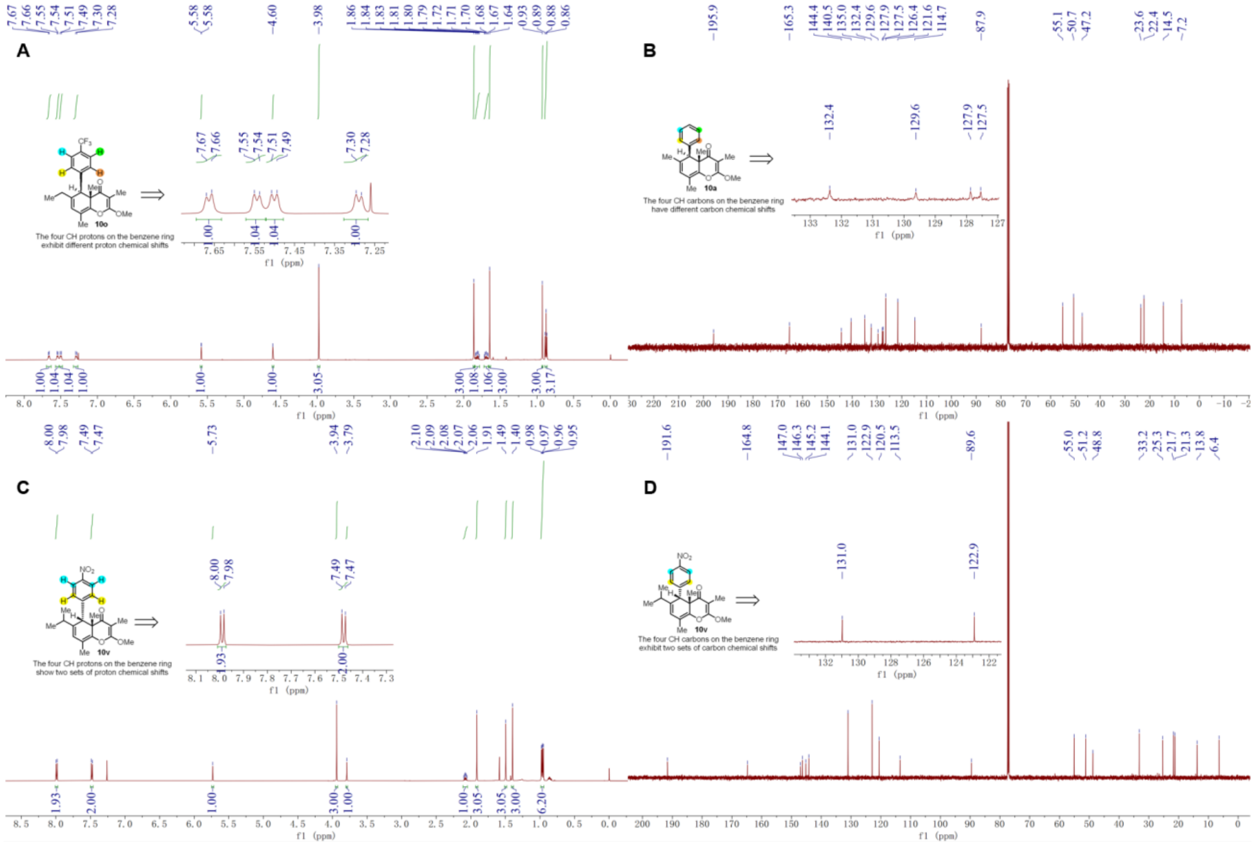


**Fig. S14.** Evidence for restricted rotation about the C–C bond connecting the aryl ring to the bicyclic core in **10a–10r**. **10o** and **10a** are shown as representative examples from the series. (A) $^{1}$H NMR spectrum of **10o** showing four nonequivalent aromatic proton signals. (B) $^{13}$C NMR spectrum of **10a** showing four nonequivalent aromatic carbon signals. (C–D) $^{1}$H and $^{13}$C NMR spectra of the corresponding trans-isomer **10v**, in which the symmetric aryl protons and carbons appear as equivalent signals, indicating free rotation upon relief of steric clash with the adjacent methyl group. Insets: expanded regions of key signals.

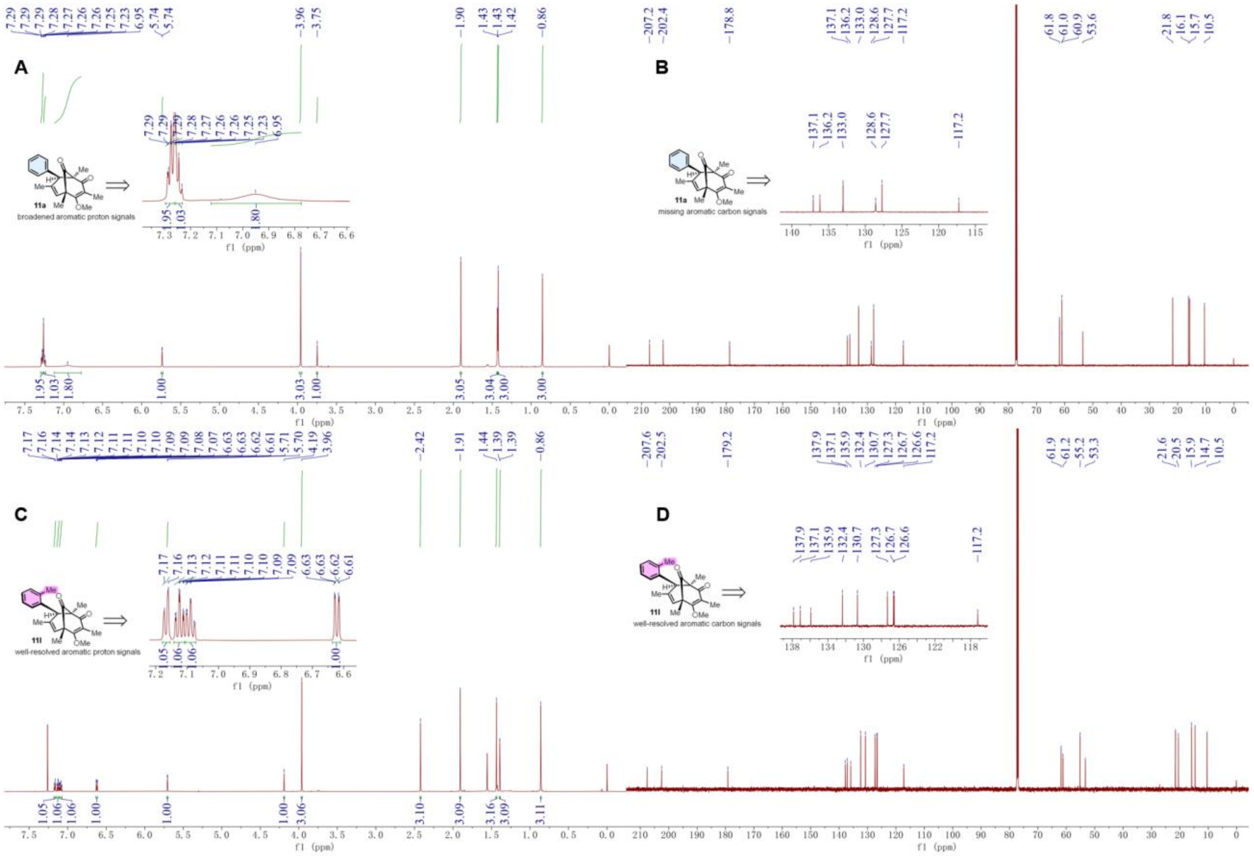

**Fig. S15.** Comparison of selected NMR spectra revealing signal anomalies in phenyl-substituted [3.3.1] bicyclic derivatives. (A) $^{1}$H NMR spectrum of **11a** showing a broadened signal at 6.95 ppm in the aromatic region. (B) $^{13}$C NMR spectrum of **11a** displaying only 17 distinct carbon signals, with three aromatic carbon signals missing. (C) $^{1}$H NMR spectrum of the *ortho*-methyl substituted derivative **11l**, exhibiting well-resolved signals for all four aromatic protons. (D) $^{13}$C NMR spectrum of **11l**, showing all carbon signals well-resolved. The restoration of normal NMR line shapes in **11l** demonstrates that increased steric hindrance around the phenyl ring raises the rotational energy barrier, effectively locking the conformation.

## Part 13. Mechanism Study of the Formation of Compounds 12–16

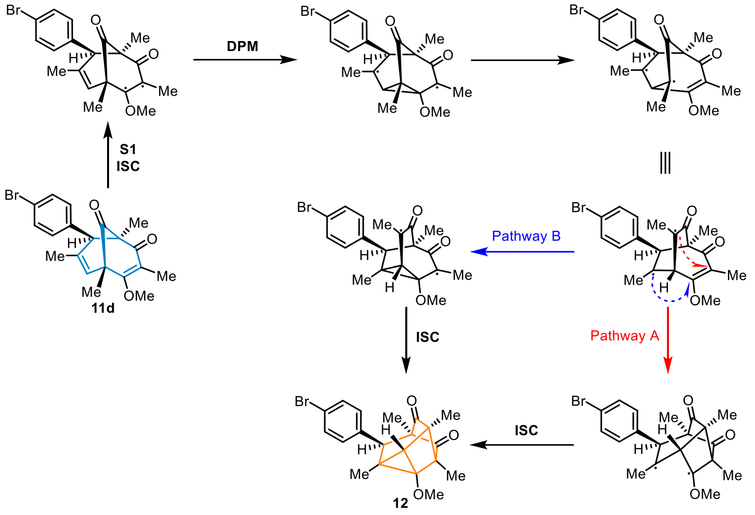


**Fig. S16.** Proposed mechanism for the formation of product **12** from compound **11d** via a triplet pathway.

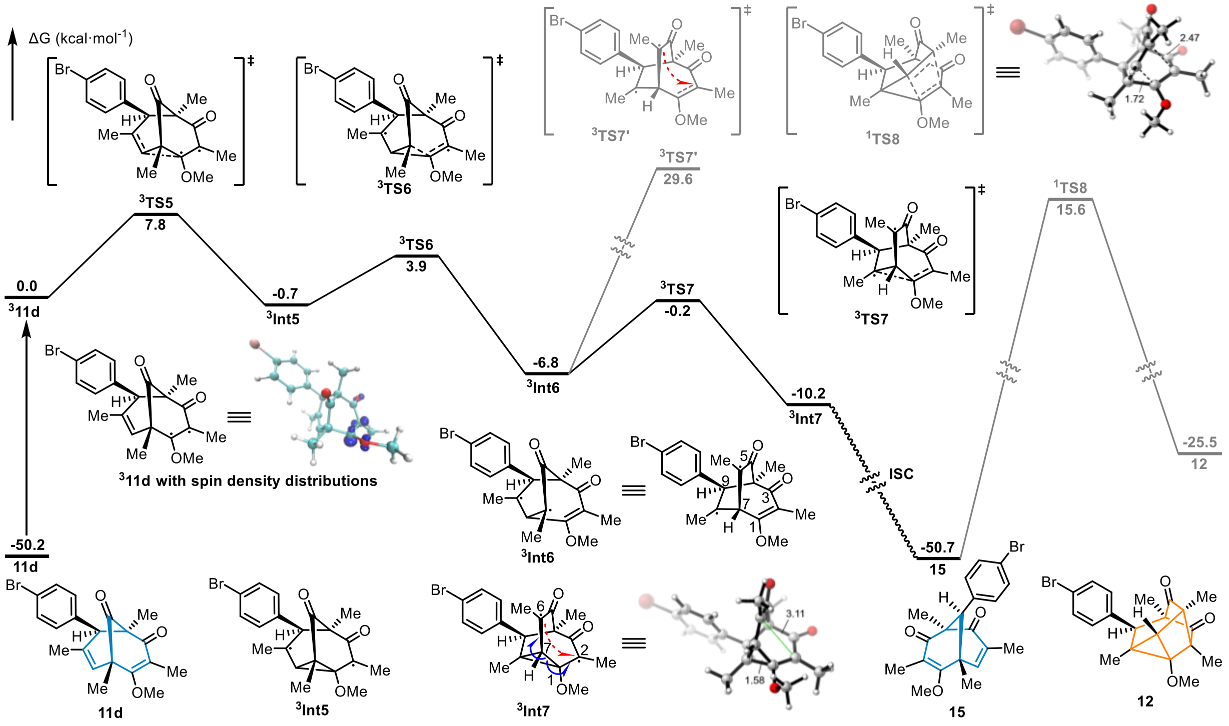


**Fig. S17.** DFT calculated free energy surface for the photoinduced triplet state reaction pathway of **11d**. Geometry optimized with (U)B3LYP-D3BJ/Def2-SVP in SMD (MeCN). Free energy calculated with SMD-(U)B3LYP-D3BJ/Def2-TZVP//SMD-(U)B3LYP-D3BJ/Def2-SVP. Molecular graphics have been produced with CYLview. The spin densities of $^3$**11d** were studied by Multiwfn and the results were visualized by VMD. Bond distance in Å. Energy in kcal·mol$^{-1}$.

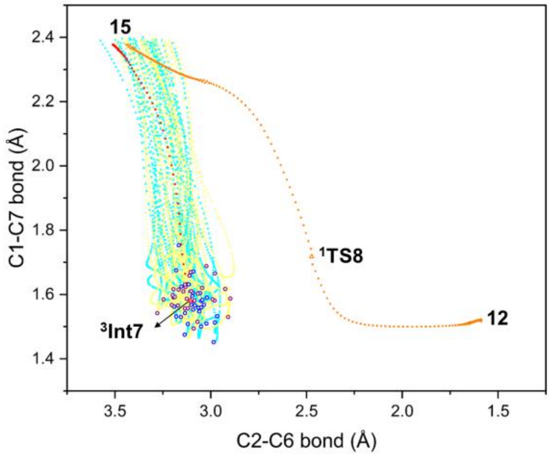


**Fig. S18.** The study of dynamic behaviors of $^3$Int7. Calculated at the (U)B3LYP-D3BJ/def2-SVP/SMD(MeCN) level of theory. All the paths and trajectories are projected to the plane with respect to the C2–C6 and C1–C7 distances in Å. Spontaneous relaxation path for $^3$Int7 structure on the open- or closed-shell singlet state and the IRC path for $^1$TS8 are shown by red and orange curves, respectively. The overlays of the BOMD simulation trajectories with OSS sampling and T-sampling are shown by green and yellow curves, respectively. The red and orange triangles show the positions of $^3$Int7 (3.11, 1.58) and $^1$TS8 (2.47, 1.72), respectively. The blue and pink circles show the positions of initial geometries for all OSS sampling and T-sampling trajectories, respectively.

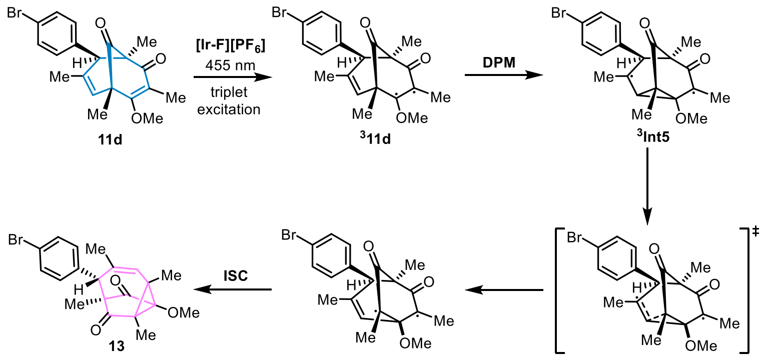


**Fig. S19.** Proposed mechanism for the formation of product **13** from compound **11d** via a triplet pathway.

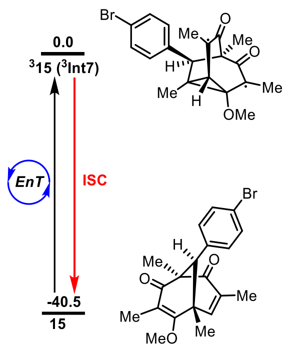


**Fig. S20.** DFT calculated free energy surface for the ineffective photocycle of **15** via triplet state. Geometry optimized with (U)B3LYP-D3BJ/Def2-SVP in SMD (MeCN). Free energy calculated with SMD-(U)B3LYP-D3BJ/Def2-TZVP//SMD-(U)B3LYP-D3BJ/Def2-SVP. Energy in kcal·mol$^{-1}$.

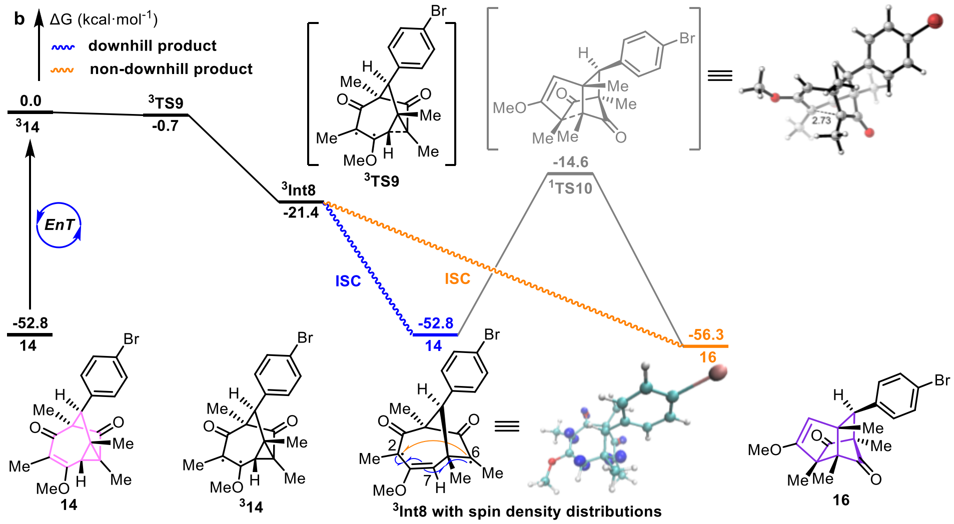


**Fig. S21.** DFT calculated free energy surface for the photoinduced triplet state reaction pathway of **14**. Geometry optimized with (U)B3LYP-D3BJ/Def2-SVP in SMD (MeCN). Free energy calculated with SMD-(U)B3LYP-D3BJ/Def2-TZVP//SMD-(U)B3LYP-D3BJ/Def2-SVP. Molecular graphics have been produced with CYLview. The spin densities of $^3$**Int8** were studied by Multiwfn and the results were visualized by VMD. Bond distance in Å. Energy in kcal·mol$^{-1}$.

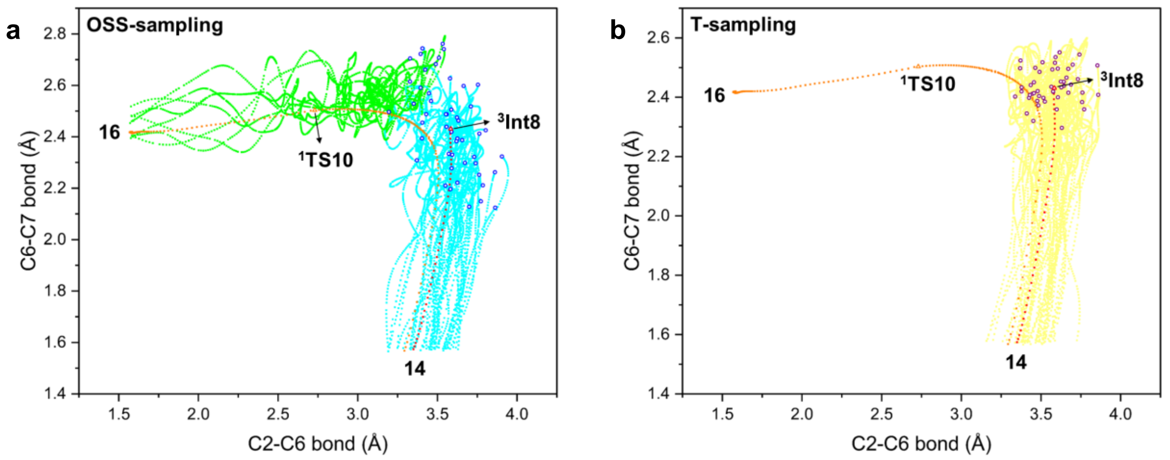


**Fig. S22.** The study of dynamic behaviors of $^3$Int8 based on OSS-sampling (a) and T-sampling (b). All the paths and trajectories are projected to the plane with respect to the C2–C6 and C6–C7 distances in Å. Spontaneous relaxation path for $^3$Int8 structure on the open- or closed-shell singlet state and the IRC path for $^1$TS10 are shown by red and orange curves, respectively. The overlays of the BOMD simulation trajectories with OSS-sampling (a) are shown by green (leading to **16**) and cyan (leading to **14**) curves. The overlays of the BOMD simulation trajectories with T-sampling (b) are shown by yellow curves. The red and orange triangles show the positions of $^3$Int8 (3.58, 2.43) and $^1$TS10 (2.73, 2.50), respectively. The blue and pink circles show the positions of initial geometries for all OSS sampling (a) and T-sampling (b) trajectories, respectively.

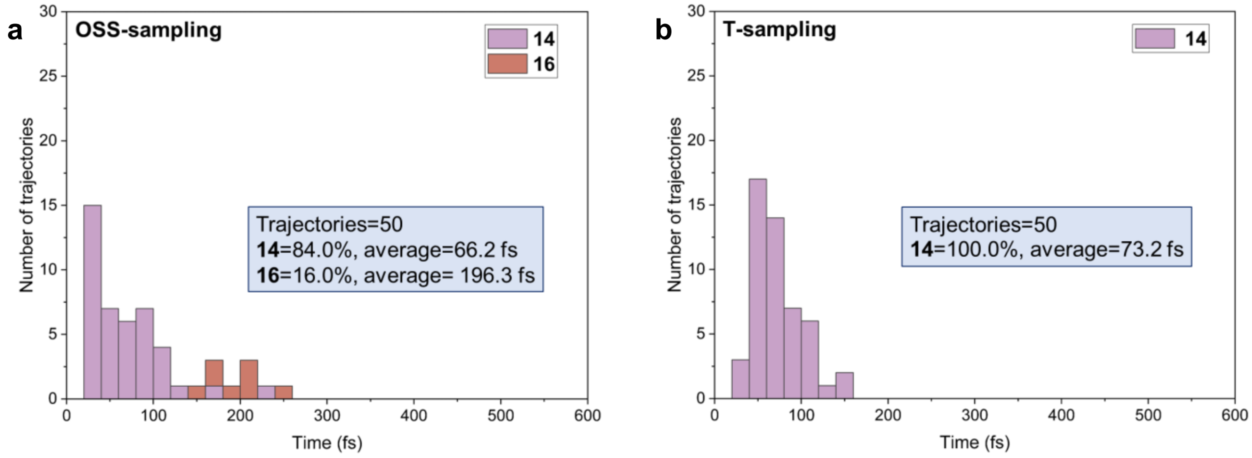


**Fig. S23.** Product formation timing histograms of OSS-sampling (a) and T-sampling (b). Trajectories were sorted into 20 fs wide bins, with purple bins corresponding to trajectories forming **14** and reddish brown bins corresponding to trajectories forming **16**.

**Table S4.** TD-DFT calculation results of $\mathbf{S_1}$, and $\mathbf{T_1}$ of compounds **11d**, **14** and **15** under B3LYP-D3BJ at Def2-TZVP level with a SMD solvation model of MeCN.

| substrate | $S_1$ excitation energy (eV) | *f* | $T_1$ excitation energy (eV) | $T_1$ characteristic |
|---|---|---|---|---|
| **11d** | 3.7848 | 0.0033 | 2.9260 | $\pi_{C=C} > \pi_{C=C}^*$ |
| **14** | 3.1933 | 0.0012 | 2.6260 | $\pi_{C=C} > \pi_{C=C}^*$ |
| **15** | 3.7271 | 0.0408 | 2.6928 | $\pi_{C=C} > \pi_{C=C}^*$ |

| substrate | $S_1$ analysis | $T_1$ analysis |
|---|---|---|
| **11d** | 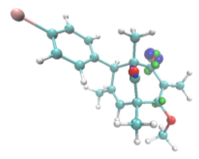 | 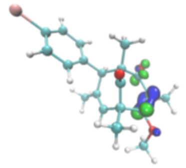 |
| **14** | 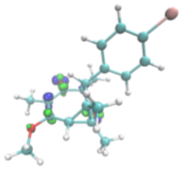 | 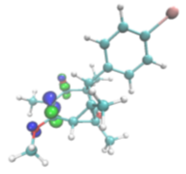 |
| **15** | 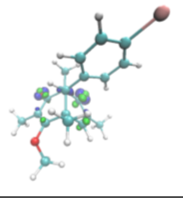 | 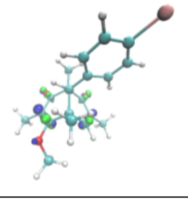 |

**Fig. S24.** The electron-hole analysis *(62)* of $S_1$ and $T_1$ of compounds **11d**, **14** and **15**. The excited state types of $S_1$ and $T_1$ calculated from SMD(MeCN)-B3LYP-D3BJ/Def2-TZVP were analyzed by Multiwfn and visualized by VMD, with the blue part representing the hole and the green part representing the electron.

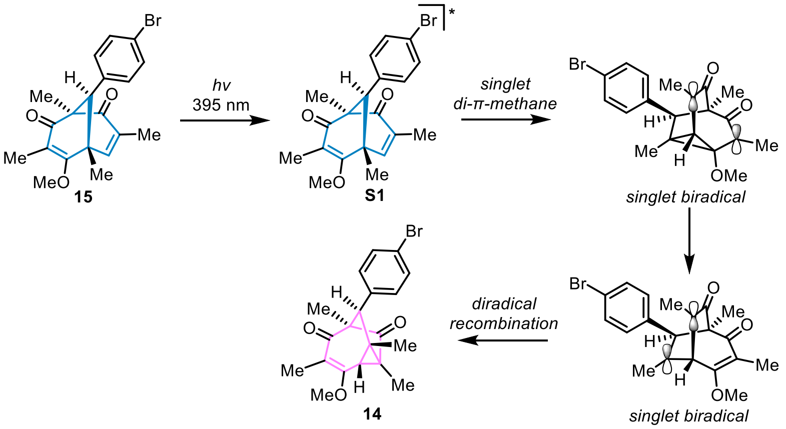


**Fig. S25.** Proposed mechanism for the formation of product **14** from compound **15** via a singlet pathway.

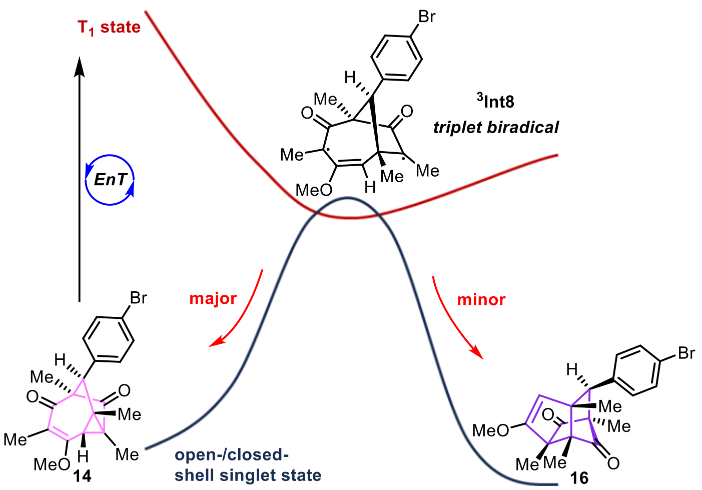


**Fig. S26.** Proposed mechanism for the formation of product **16** from compound **14** via a vinylcyclopropane-cyclopentene (VCP-CP) rearrangement featuring post-spin crossing dynamic effects.

## Part 14. Evaluation of Anti-malarial Activity

**Table S5.** Anti-malarial activity assay of polycyclic compounds.[a]

| cmpd | Inhibition rate (%) | | cmpd | Inhibition rate (%) | | cmpd | Inhibition rate (%) | |
|---|---|---|---|---|---|---|---|---|
| | 10 μM | 5 μM | | 10 μM | 5 μM | | 10 μM | 5 μM |
| **1** | 78 | NT | **11o** | 94 | 69 | **17e** | 94 | 90 |
| **11a** | 67 | NT | **11p** | 94 | 83 | **17f** | 89.5 | 49.5 |
| **11b** | 85 | 26.5 | **11q** | 94.5 | 84.5 | **18a** | -9.5 | NT |
| **11c** | 83 | 37.5 | **11r** | 89 | 34 | **18b** | 76 | NT |
| **11d** | 90 | 14 | **11s** | 29 | NT | **19a** | 86 | 28.5 |
| **11e** | 94 | 45.5 | **11t** | 87 | 19 | **19b** | NT | NT |
| **11f** | 90.5 | 29 | **12** | 16 | NT | **19c** | 55.5 | NT |
| **11g** | 91 | 81.5 | **13** | 61.5 | NT | **19d** | 26.5 | NT |
| **11h** | 96.5 | 65 | **14** | 49.5 | NT | **20** | 72 | NT |
| **11i** | 5.5 | NT | **15** | 25 | NT | **21a** | 27 | NT |
| **11j** | 83.5 | 9 | **16** | -12 | NT | **21b** | 75.5 | NT |
| **11k** | 92 | 90.5 | **17a** | 88 | 62.5 | **21c** | 93.5 | 25.5 |
| **11l** | 33 | NT | **17b** | 88 | 59 | **21d** | 25.5 | NT |
| **11m** | 91 | 46.5 | **17c** | 91 | 32.5 | | | |
| **11n** | 97.5 | 61.5 | **17d** | 72.5 | NT | | | |

[a]Under a compound concentration of 10 μM, initial screening for antimalarial activity was conducted. Compounds exhibiting an inhibition rate greater than 80% at 10 μM were further evaluated for their inhibition rates at 5 μM. NT indicates "not tested."

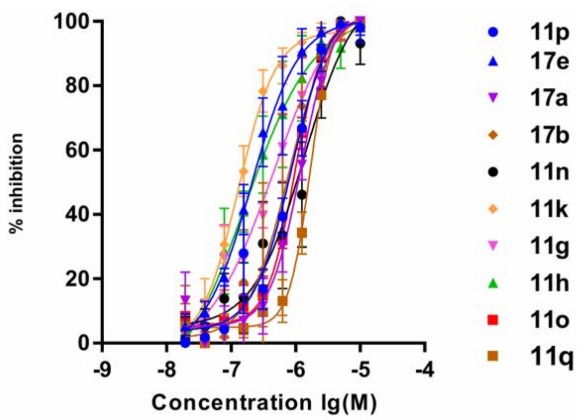


| cmpd | 11g | 11h | 11k | 11n | 11o | 11p | 11q | 17a | 17b | 17e |
|---|---|---|---|---|---|---|---|---|---|---|
| $IC_{50}$ (μM) | 0.45 | 0.17 | 0.12 | 1.31 | 0.98 | 0.87 | 1.69 | 1.15 | 0.77 | 0.21 |

**Fig. S27.** Anti-malarial $IC_{50}$ values of polycyclic compounds.

# Part 15. Energies and Cartesian Coordinates of the Optimized Structures

tridachiahydropyrone **2**

B3LYP-D3BJ/Def2-SVP Electronic Energy: -1043.298765 a.u.

B3LYP-D3BJ/Def2-SVP Gibbs free Energy: -1042.891435 a.u.

B3LYP-D3BJ/Def2-TZVP Electronic Energy: -1044.447128 a.u.

| | | | |
|---|---|---|---|
| O | 2.752402 | 0.309731 | -1.044398 |
| C | 3.458644 | -0.602353 | -0.360919 |
| O | 4.668247 | -0.829259 | -0.842634 |
| C | 2.991488 | -1.260792 | 0.75402 |
| O | 1.081704 | -1.731328 | 2.055199 |
| C | 1.582271 | -1.172526 | 1.076085 |
| C | 0.70154 | -0.414187 | 0.03719 |
| C | 1.569016 | 0.723438 | -0.41007 |
| C | 1.43219 | 2.011335 | -0.045836 |
| C | 0.326057 | 2.353249 | 0.85463 |
| H | 0.302776 | 3.378125 | 1.23802 |
| C | -0.591329 | 1.458283 | 1.270324 |
| C | -0.645833 | 0.061806 | 0.675109 |
| H | -0.844803 | -0.65039 | 1.489117 |
| C | -1.845504 | -0.012801 | -0.27025 |
| C | -2.789571 | -0.949263 | -0.066953 |
| H | -2.649863 | -1.621535 | 0.789709 |
| C | -4.042069 | -1.187868 | -0.859789 |
| H | -4.1316 | -2.271999 | -1.052713 |
| H | -3.990387 | -0.697519 | -1.845859 |
| C | -5.333391 | -0.722762 | -0.148629 |
| H | -5.347438 | -1.190136 | 0.854095 |
| C | -5.357766 | 0.795758 | 0.038524 |
| H | -4.485012 | 1.147668 | 0.609831 |
| H | -6.264032 | 1.11584 | 0.578766 |
| H | -5.349655 | 1.312593 | -0.936882 |
| C | -6.5698 | -1.203128 | -0.911457 |
| H | -7.499155 | -0.900468 | -0.401105 |
| H | -6.582292 | -2.301187 | -1.010892 |
| H | -6.594316 | -0.775638 | -1.929191 |
| C | -1.912289 | 1.023624 | -1.361783 |
| H | -2.600277 | 0.738085 | -2.168897 |
| H | -0.920841 | 1.207549 | -1.80348 |
| H | -2.25858 | 1.991693 | -0.960767 |
| C | 0.451014 | -1.433692 | -1.102125 |
| H | 1.394671 | -1.826496 | -1.504725 |
| H | -0.106915 | -0.975471 | -1.92918 |
| H | -0.134077 | -2.280594 | -0.717876 |
| C | -1.6671 | 1.801738 | 2.254168 |
| H | -1.580749 | 2.838541 | 2.611551 |
| H | -1.62509 | 1.125472 | 3.12656 |
| H | -2.669014 | 1.668117 | 1.811481 |
| C | 2.410368 | 3.087908 | -0.41424 |
| H | 3.181166 | 2.733491 | -1.1104 |
| H | 2.91008 | 3.477253 | 0.489969 |
| H | 1.885396 | 3.941043 | -0.876234 |
| C | 5.210249 | 0.03817 | -1.852054 |
| H | 6.213759 | -0.350694 | -2.062063 |
| H | 5.278199 | 1.070726 | -1.47904 |
| H | 4.596808 | 0.007403 | -2.763394 |
| C | 3.877802 | -2.172237 | 1.55724 |
| H | 3.769439 | -3.229224 | 1.253778 |
| H | 4.938107 | -1.902078 | 1.452222 |
| H | 3.608956 | -2.115522 | 2.62316 |

**$MeOH_2^+$·MeOH**

B3LYP-D3BJ/Def2-SVP Electronic Energy: -231.742348 a.u.

B3LYP-D3BJ/Def2-SVP Gibbs free Energy: -231.657626 a.u.

B3LYP-D3BJ/Def2-TZVP Electronic Energy: -232.017516 a.u.

| | | | |
|---|---|---|---|
| C | -1.82683 | 0.635207 | 0.098806 |
| H | -2.915568 | 0.496885 | 0.139539 |
| H | -1.468577 | 1.044448 | 1.051562 |
| H | -1.555424 | 1.306505 | -0.728831 |
| O | -1.200858 | -0.657101 | -0.06024 |
| H | -1.477444 | -1.077071 | -0.894543 |
| H | -0.000014 | -0.651038 | -0.000054 |
| C | 1.826736 | 0.635287 | -0.098805 |
| H | 2.915493 | 0.497155 | -0.139651 |
| H | 1.468314 | 1.044501 | -1.051512 |
| H | 1.555286 | 1.306498 | 0.728886 |
| O | 1.200979 | -0.657139 | 0.060235 |
| H | 1.47753 | -1.07693 | 0.894636 |

**MeOH**

B3LYP-D3BJ/Def2-SVP Electronic Energy: -115.642188 a.u.

B3LYP-D3BJ/Def2-SVP Gibbs free Energy: -115.613941 a.u.

B3LYP-D3BJ/Def2-TZVP Electronic Energy: -115.786315 a.u.

| | | | |
|---|---|---|---|
| C | 0.659279 | -0.019044 | 0 |
| H | 1.101463 | 0.99026 | -0.000754 |
| H | 1.034928 | -0.550897 | 0.895882 |
| H | 1.034787 | -0.552175 | -0.89517 |
| O | -0.748784 | 0.123844 | 0.000004 |
| H | -1.136576 | -0.763676 | 0.000011 |

**Int1**

B3LYP-D3BJ/Def2-SVP Electronic Energy: -1043.741818 a.u.

B3LYP-D3BJ/Def2-SVP Gibbs free Energy: -1043.319514 a.u.

B3LYP-D3BJ/Def2-TZVP Electronic Energy: -1044.888001 a.u.

| | | | |
|---|---|---|---|
| O | 2.785943 | 0.406661 | -0.981278 |
| C | 3.457463 | -0.542186 | -0.375851 |
| O | 4.644338 | -0.792117 | -0.827041 |
| C | 2.963861 | -1.273966 | 0.731967 |
| O | 0.980762 | -1.825275 | 1.927634 |
| C | 1.601662 | -1.178928 | 0.972718 |
| C | 0.709217 | -0.387695 | 0.014942 |
| C | 1.563819 | 0.782871 | -0.377408 |

| | | | |
|---|---|---|---|
| C | 1.397778 | 2.063136 | -0.004156 |
| C | 0.280894 | 2.375434 | 0.891245 |
| H | 0.237093 | 3.39483 | 1.285006 |
| C | -0.614491 | 1.453681 | 1.294848 |
| C | -0.649714 | 0.068981 | 0.672191 |
| H | -0.867503 | -0.654445 | 1.467094 |
| C | -1.842516 | 0.010825 | -0.285769 |
| C | -2.772239 | -0.94511 | -0.109978 |
| H | -2.62175 | -1.643416 | 0.723498 |
| C | -4.024096 | -1.172774 | -0.905713 |
| H | -4.099723 | -2.250964 | -1.133718 |
| H | -3.983762 | -0.648357 | -1.874387 |
| C | -5.316328 | -0.748634 | -0.170739 |
| H | -5.316955 | -1.248997 | 0.815995 |
| C | -5.359087 | 0.762544 | 0.066072 |
| H | -4.486761 | 1.107865 | 0.642152 |
| H | -6.265314 | 1.052345 | 0.623092 |
| H | -5.365578 | 1.310694 | -0.892015 |
| C | -6.551328 | -1.220547 | -0.940923 |
| H | -7.48068 | -0.947347 | -0.41433 |
| H | -6.549823 | -2.314776 | -1.076424 |
| H | -6.588841 | -0.759938 | -1.943624 |
| C | -1.928308 | 1.084021 | -1.339584 |
| H | -2.602205 | 0.805586 | -2.160632 |
| H | -0.940687 | 1.312353 | -1.768531 |
| H | -2.306358 | 2.026533 | -0.908041 |
| C | 0.451238 | -1.343069 | -1.183229 |
| H | 1.394325 | -1.692416 | -1.625288 |
| H | -0.125005 | -0.833127 | -1.964214 |
| H | -0.116285 | -2.219477 | -0.843464 |
| C | -1.692571 | 1.755992 | 2.287958 |
| H | -1.628945 | 2.789447 | 2.658083 |
| H | -1.626816 | 1.068206 | 3.149478 |
| H | -2.692132 | 1.601987 | 1.847444 |
| C | 2.356278 | 3.159977 | -0.356794 |
| H | 3.134325 | 2.83322 | -1.057845 |
| H | 2.84358 | 3.542351 | 0.556571 |
| H | 1.809581 | 4.00739 | -0.802424 |
| C | 5.206088 | 0.013455 | -1.891213 |
| H | 6.19541 | -0.415681 | -2.081313 |
| H | 5.291419 | 1.056595 | -1.558253 |
| H | 4.572005 | -0.056572 | -2.784862 |
| C | 3.90304 | -2.19531 | 1.462991 |
| H | 3.485854 | -2.53442 | 2.422714 |
| H | 4.13134 | -3.091173 | 0.862457 |
| H | 4.85357 | -1.688073 | 1.683909 |
| H | 1.58431 | -2.360572 | 2.475908 |

**Int2**

B3LYP-D3BJ/Def2-SVP Electronic Energy: -1159.396887 a.u.

B3LYP-D3BJ/Def2-SVP Gibbs free Energy: -1158.922942 a.u.

B3LYP-D3BJ/Def2-TZVP Electronic Energy: -1160.677722 a.u.

| | | | |
|---|---|---|---|
| C | 3.411031 | 0.087384 | 0.318621 |
| O | 4.157494 | -0.034389 | -0.731213 |
| C | 2.653294 | 1.278586 | 0.648727 |
| O | 0.733888 | 2.358651 | 1.437223 |
| C | 1.335928 | 1.245507 | 1.001219 |
| C | 0.344662 | 0.072098 | 0.925151 |
| C | 0.939877 | -1.1077 | 0.167701 |
| C | 1.097311 | -1.040735 | -1.17992 |
| C | 0.491777 | 0.092369 | -1.884876 |
| H | 0.780232 | 0.249575 | -2.929132 |
| C | -0.466194 | 0.864168 | -1.326606 |
| C | -0.885071 | 0.599536 | 0.112222 |
| H | -1.156131 | 1.560589 | 0.561756 |
| C | -2.136454 | -0.269749 | 0.200254 |
| C | -3.233801 | 0.25906 | 0.77581 |
| H | -3.155591 | 1.290146 | 1.144736 |
| C | -4.583726 | -0.366642 | 0.966736 |
| H | -4.869183 | -0.25643 | 2.028744 |
| H | -4.561588 | -1.448921 | 0.762121 |
| C | -5.690682 | 0.281958 | 0.10543 |
| H | -5.669888 | 1.370352 | 0.303431 |
| C | -5.437554 | 0.069297 | -1.388728 |
| H | -4.452968 | 0.458043 | -1.693716 |
| H | -6.203311 | 0.576637 | -1.998484 |
| H | -5.466363 | -1.004686 | -1.642823 |
| C | -7.067854 | -0.246028 | 0.512336 |
| H | -7.870091 | 0.229274 | -0.076035 |
| H | -7.274188 | -0.05609 | 1.578692 |
| H | -7.135243 | -1.335769 | 0.348235 |
| C | -2.083299 | -1.66055 | -0.374925 |
| H | -3.07521 | -2.128573 | -0.419351 |
| H | -1.42828 | -2.319578 | 0.218894 |
| H | -1.670796 | -1.644944 | -1.39568 |
| C | -0.052092 | -0.299099 | 2.363326 |
| H | 0.832291 | -0.588178 | 2.948441 |
| H | -0.759199 | -1.138811 | 2.366668 |
| H | -0.531783 | 0.559213 | 2.853433 |
| C | -1.18761 | 1.953794 | -2.056543 |
| H | -0.881976 | 2.015955 | -3.111777 |
| H | -1.004596 | 2.934612 | -1.582108 |
| H | -2.279282 | 1.791312 | -2.01839 |
| C | 1.740425 | -2.118459 | -2.00652 |
| H | 2.231665 | -2.899091 | -1.406184 |
| H | 2.510051 | -1.685946 | -2.667379 |
| H | 1.001293 | -2.614667 | -2.65981 |
| C | 4.010299 | 0.831151 | -1.881995 |
| H | 4.763257 | 1.626468 | -1.817002 |
| H | 2.997347 | 1.248443 | -1.918092 |
| H | 4.202403 | 0.1982 | -2.755532 |
| C | 3.418863 | 2.590104 | 0.647246 |
| H | 2.993008 | 3.315291 | -0.065844 |
| H | 3.403205 | 3.04369 | 1.654337 |

| | | | |
|---|---|---|---|
| H | 4.477982 | 2.447593 | 0.396636 |
| H | 1.36427 | 3.088065 | 1.560721 |
| O | 3.54532 | -0.830429 | 1.218044 |
| C | 4.315679 | -2.025884 | 0.94162 |
| H | 4.001458 | -2.466233 | -0.011735 |
| H | 5.381658 | -1.763125 | 0.907711 |
| H | 4.106383 | -2.701009 | 1.777288 |
| O | 1.225172 | -2.188503 | 0.933871 |
| H | 1.495303 | -2.935051 | 0.37611 |

**Int3**

B3LYP-D3BJ/Def2-SVP Electronic Energy: -1159.408788 a.u.

B3LYP-D3BJ/Def2-SVP Gibbs free Energy: -1158.935882 a.u.

B3LYP-D3BJ/Def2-TZVP Electronic Energy: -1160.690636 a.u.

| | | | |
|---|---|---|---|
| C | -3.392498 | -0.220983 | -0.114996 |
| O | -4.127205 | 0.828157 | -0.038145 |
| C | -2.546537 | -0.600481 | -1.277813 |
| O | -1.00093 | -2.39265 | -1.479952 |
| C | -1.288004 | -1.389912 | -0.866684 |
| C | -0.331144 | -0.867777 | 0.242286 |
| C | -0.962238 | 0.227456 | 1.080546 |
| C | -1.039205 | 1.506498 | 0.623731 |
| C | -0.358819 | 1.840975 | -0.629504 |
| H | -0.578877 | 2.816281 | -1.074644 |
| C | 0.560922 | 1.033407 | -1.200805 |
| C | 0.904851 | -0.289255 | -0.534095 |
| H | 1.129932 | -1.022738 | -1.320172 |
| C | 2.170319 | -0.162593 | 0.31124 |
| C | 3.250587 | -0.883004 | -0.045775 |
| H | 3.151712 | -1.52704 | -0.929229 |
| C | 4.610316 | -0.898535 | 0.587892 |
| H | 4.910215 | -1.950784 | 0.741066 |
| H | 4.597423 | -0.429377 | 1.584698 |
| C | 5.696346 | -0.211377 | -0.271117 |
| H | 5.662933 | -0.670397 | -1.277233 |
| C | 5.425925 | 1.286825 | -0.426998 |
| H | 4.431498 | 1.477424 | -0.860702 |
| H | 6.175371 | 1.759973 | -1.08275 |
| H | 5.468177 | 1.797651 | 0.550738 |
| C | 7.086125 | -0.462772 | 0.317305 |
| H | 7.87312 | 0.00135 | -0.299868 |
| H | 7.305569 | -1.540947 | 0.388632 |
| H | 7.165612 | -0.037955 | 1.333283 |
| C | 2.142389 | 0.786929 | 1.47924 |
| H | 3.128819 | 0.8953 | 1.947551 |
| H | 1.436591 | 0.451305 | 2.257172 |
| H | 1.807547 | 1.787272 | 1.162201 |
| C | 0.075559 | -2.085123 | 1.087031 |
| H | -0.808749 | -2.571045 | 1.52255 |
| H | 0.742441 | -1.785462 | 1.905023 |
| H | 0.600379 | -2.815065 | 0.457583 |
| C | 1.329688 | 1.395589 | -2.433031 |
| H | 1.069167 | 2.40034 | -2.797785 |
| H | 1.142619 | 0.670037 | -3.244571 |
| H | 2.415836 | 1.366666 | -2.236845 |
| C | -1.687454 | 2.611509 | 1.411064 |
| H | -2.461447 | 2.243395 | 2.103189 |
| H | -2.16663 | 3.339748 | 0.739018 |
| H | -0.941429 | 3.166471 | 2.008213 |
| C | -4.116986 | 1.863618 | -1.056165 |
| H | -4.560884 | 1.462571 | -1.975902 |
| H | -3.090895 | 2.207294 | -1.227316 |
| H | -4.732289 | 2.670045 | -0.645332 |
| C | -3.405352 | -1.396249 | -2.27569 |
| H | -2.803841 | -1.615925 | -3.167573 |
| H | -3.737006 | -2.343534 | -1.827787 |
| H | -4.287721 | -0.815038 | -2.577694 |
| H | -2.179662 | 0.317737 | -1.760688 |
| O | -3.536639 | -1.112288 | 0.797078 |
| C | -4.428301 | -0.89398 | 1.922307 |
| H | -4.164458 | 0.041773 | 2.427855 |
| H | -5.461716 | -0.861164 | 1.552825 |
| H | -4.270098 | -1.755149 | 2.578514 |
| O | -1.392282 | -0.179847 | 2.300669 |
| H | -1.691735 | 0.577405 | 2.828955 |

**Int3’**

B3LYP-D3BJ/Def2-SVP Electronic Energy: -1159.407030 a.u.

B3LYP-D3BJ/Def2-SVP Gibbs free Energy: -1158.933552 a.u.

B3LYP-D3BJ/Def2-TZVP Electronic Energy: -1160.689135 a.u.

| | | | |
|---|---|---|---|
| C | -3.39193 | 0.097187 | 0.170444 |
| O | -3.391193 | -0.11263 | 1.431969 |
| C | -2.85979 | 1.3063 | -0.53813 |
| O | -1.039819 | 2.672323 | 0.20258 |
| C | -1.36553 | 1.627893 | -0.312966 |
| C | -0.314539 | 0.659247 | -0.901277 |
| C | -0.932774 | -0.703387 | -1.134467 |
| C | -0.998534 | -1.623059 | -0.137267 |
| C | -0.395426 | -1.292608 | 1.156993 |
| H | -0.656823 | -1.931639 | 2.006314 |
| C | 0.464453 | -0.266729 | 1.322593 |
| C | 0.861057 | 0.565797 | 0.113007 |
| H | 1.039505 | 1.594632 | 0.451269 |
| C | 2.179374 | 0.063473 | -0.471117 |
| C | 3.26629 | 0.851475 | -0.363021 |
| H | 3.129127 | 1.825754 | 0.123669 |
| C | 4.678248 | 0.550536 | -0.773528 |
| H | 5.091191 | 1.438248 | -1.284488 |
| H | 4.721148 | -0.27667 | -1.500306 |
| C | 5.603685 | 0.210745 | 0.418545 |
| H | 5.507096 | 1.029528 | 1.156338 |
| C | 5.18826 | -1.092995 | 1.104248 |
| H | 4.140326 | -1.061363 | 1.441279 |
| H | 5.818899 | -1.295615 | 1.985661 |

| | | | |
|---|---|---|---|
| H | 5.292924 | -1.949477 | 0.415784 |
| C | 7.064021 | 0.151947 | -0.034386 |
| H | 7.737774 | -0.066451 | 0.810675 |
| H | 7.386439 | 1.105185 | -0.484975 |
| H | 7.209363 | -0.640435 | -0.789397 |
| C | 2.202849 | -1.313853 | -1.07988 |
| H | 3.212477 | -1.613332 | -1.387937 |
| H | 1.548188 | -1.378711 | -1.964552 |
| H | 1.833625 | -2.063594 | -0.36222 |
| C | 0.139614 | 1.334904 | -2.213092 |
| H | -0.712475 | 1.544003 | -2.874907 |
| H | 0.842548 | 0.688892 | -2.755352 |
| H | 0.641813 | 2.283701 | -1.979374 |
| C | 1.089161 | 0.089262 | 2.634841 |
| H | 0.834845 | -0.637098 | 3.421333 |
| H | 0.757602 | 1.089802 | 2.967574 |
| H | 2.188324 | 0.14084 | 2.548762 |
| C | -1.583797 | -2.994505 | -0.334648 |
| H | -2.392079 | -3.018603 | -1.083094 |
| H | -1.998191 | -3.381548 | 0.608383 |
| H | -0.808577 | -3.709814 | -0.664551 |
| C | -2.80349 | 0.738557 | 2.453573 |
| H | -3.613978 | 1.360431 | 2.854293 |
| H | -1.992487 | 1.350213 | 2.053642 |
| H | -2.427127 | 0.053189 | 3.219257 |
| C | -3.759445 | 2.514961 | -0.234102 |
| H | -3.66487 | 2.845871 | 0.808306 |
| H | -3.458104 | 3.345381 | -0.887697 |
| H | -4.810572 | 2.268091 | -0.440365 |
| H | -2.944501 | 1.048574 | -1.60394 |
| O | -4.085755 | -0.710082 | -0.550414 |
| C | -4.841557 | -1.785532 | 0.070611 |
| H | -4.183653 | -2.389324 | 0.704759 |
| H | -5.653055 | -1.343643 | 0.664255 |
| H | -5.237078 | -2.371363 | -0.764793 |
| O | -1.418904 | -0.882877 | -2.389214 |
| H | -1.835463 | -1.755231 | -2.47481 |

**TS1**

B3LYP-D3BJ/Def2-SVP Electronic Energy: -1159.392363 a.u.

B3LYP-D3BJ/Def2-SVP Gibbs free Energy: -1158.918006 a.u.

B3LYP-D3BJ/Def2-TZVP Electronic Energy: -1160.670610 a.u.

| | | | |
|---|---|---|---|
| C | -2.99218 | 0.151147 | -0.097123 |
| O | -3.871671 | 1.163214 | -0.132658 |
| C | -2.34761 | -0.300678 | -1.402176 |
| O | -1.067708 | -2.246043 | -1.919952 |
| C | -1.236712 | -1.314551 | -1.169594 |
| C | -0.225557 | -1.113483 | 0.014578 |
| C | -0.955471 | -0.481344 | 1.148275 |
| C | -1.494829 | 0.820307 | 0.986594 |
| C | -0.690289 | 1.718272 | 0.108433 |
| H | -1.006423 | 2.762327 | 0.075266 |
| C | 0.381013 | 1.320204 | -0.596172 |
| C | 0.874683 | -0.111701 | -0.537593 |
| H | 1.085262 | -0.44859 | -1.563255 |
| C | 2.182165 | -0.203133 | 0.23921 |
| C | 3.263619 | -0.724861 | -0.367911 |
| H | 3.141385 | -1.052112 | -1.40835 |
| C | 4.646158 | -0.895854 | 0.186614 |
| H | 4.981215 | -1.924922 | -0.03458 |
| H | 4.656363 | -0.79164 | 1.283477 |
| C | 5.679649 | 0.084056 | -0.414528 |
| H | 5.623277 | -0.007865 | -1.515495 |
| C | 5.361176 | 1.534188 | -0.044073 |
| H | 4.349705 | 1.824432 | -0.368731 |
| H | 6.076773 | 2.230171 | -0.511934 |
| H | 5.416891 | 1.682147 | 1.048447 |
| C | 7.095898 | -0.29786 | 0.020457 |
| H | 7.846552 | 0.377884 | -0.421582 |
| H | 7.348242 | -1.327276 | -0.283182 |
| H | 7.199257 | -0.238416 | 1.118038 |
| C | 2.163781 | 0.330993 | 1.644737 |
| H | 3.124027 | 0.194028 | 2.157302 |
| H | 1.390538 | -0.163361 | 2.25784 |
| H | 1.927531 | 1.407938 | 1.652002 |
| C | 0.369467 | -2.473956 | 0.377223 |
| H | -0.426452 | -3.195237 | 0.608086 |
| H | 1.028696 | -2.390045 | 1.250027 |
| H | 0.954535 | -2.857714 | -0.466961 |
| C | 1.185789 | 2.260005 | -1.441382 |
| H | 0.779976 | 3.281398 | -1.412367 |
| H | 1.205495 | 1.918109 | -2.490864 |
| H | 2.23534 | 2.290021 | -1.103968 |
| C | -2.119732 | 1.499717 | 2.188055 |
| H | -2.774286 | 0.835969 | 2.773245 |
| H | -2.731203 | 2.352052 | 1.86367 |
| H | -1.329085 | 1.883036 | 2.853054 |
| C | -3.702302 | 2.338088 | -0.932764 |
| H | -3.117033 | 2.143374 | -1.840316 |
| H | -3.219379 | 3.126953 | -0.339996 |
| H | -4.7138 | 2.661174 | -1.212101 |
| C | -3.407053 | -0.841429 | -2.363157 |
| H | -2.936654 | -1.132025 | -3.312122 |
| H | -3.904819 | -1.722695 | -1.933599 |
| H | -4.166663 | -0.074547 | -2.569105 |
| H | -1.841584 | 0.562007 | -1.861131 |
| O | -3.506384 | -0.916999 | 0.560038 |
| C | -4.739769 | -0.813806 | 1.283196 |
| H | -4.709649 | -0.013511 | 2.034683 |
| H | -5.576971 | -0.639457 | 0.591563 |
| H | -4.863804 | -1.783807 | 1.780698 |
| O | -1.194332 | -1.237936 | 2.172314 |
| H | -1.683857 | -0.773617 | 2.878909 |

**TS1’**

B3LYP-D3BJ/Def2-SVP Electronic Energy: -1159.388943 a.u.

B3LYP-D3BJ/Def2-SVP Gibbs free Energy: -1158.914242 a.u.

B3LYP-D3BJ/Def2-TZVP Electronic Energy: -1160.667277 a.u.

| | | | |
|---|---|---|---|
| C | -3.045425 | -0.181555 | 0.045206 |
| O | -3.437287 | -0.533986 | 1.285796 |
| C | -2.742469 | 1.297127 | -0.255108 |
| O | -0.990964 | 2.684084 | 0.640849 |
| C | -1.307687 | 1.716691 | -0.007286 |
| C | -0.221405 | 0.901847 | -0.79175 |
| C | -0.884818 | -0.35041 | -1.248144 |
| C | -1.444368 | -1.195376 | -0.24803 |
| C | -0.702473 | -1.159819 | 1.048398 |
| H | -1.076268 | -1.844084 | 1.813025 |
| C | 0.373451 | -0.394072 | 1.293226 |
| C | 0.903872 | 0.558094 | 0.239067 |
| H | 1.124371 | 1.517559 | 0.72875 |
| C | 2.192828 | 0.054272 | -0.391827 |
| C | 3.316156 | 0.778392 | -0.23722 |
| H | 3.235516 | 1.706105 | 0.343778 |
| C | 4.696138 | 0.465112 | -0.732734 |
| H | 5.085786 | 1.355688 | -1.25787 |
| H | 4.682983 | -0.352515 | -1.47111 |
| C | 5.687809 | 0.102926 | 0.396249 |
| H | 5.652192 | 0.918365 | 1.143036 |
| C | 5.291141 | -1.197855 | 1.097934 |
| H | 4.26983 | -1.144519 | 1.506755 |
| H | 5.975411 | -1.424117 | 1.932218 |
| H | 5.326985 | -2.049551 | 0.396521 |
| C | 7.115333 | 0.022072 | -0.148172 |
| H | 7.836791 | -0.209885 | 0.652625 |
| H | 7.424054 | 0.97168 | -0.615656 |
| H | 7.200218 | -0.769885 | -0.912707 |
| C | 2.112613 | -1.250851 | -1.136658 |
| H | 3.101558 | -1.645055 | -1.402616 |
| H | 1.540485 | -1.143932 | -2.07449 |
| H | 1.596057 | -2.015523 | -0.534208 |
| C | 0.274763 | 1.791359 | -1.935592 |
| H | -0.558147 | 2.123537 | -2.572386 |
| H | 0.995495 | 1.248527 | -2.561792 |
| H | 0.770242 | 2.676078 | -1.513964 |
| C | 1.115791 | -0.434578 | 2.593084 |
| H | 0.690806 | -1.177027 | 3.283736 |
| H | 1.093098 | 0.555344 | 3.081387 |
| H | 2.178764 | -0.676643 | 2.426294 |
| C | -1.890175 | -2.585033 | -0.657099 |
| H | -2.530537 | -2.5807 | -1.550862 |
| H | -2.453894 | -3.051909 | 0.161724 |
| H | -1.007147 | -3.212016 | -0.858709 |
| C | -3.02384 | 0.128893 | 2.489189 |
| H | -3.89259 | 0.66203 | 2.902335 |
| H | -2.197301 | 0.827447 | 2.326033 |
| H | -2.699659 | -0.644798 | 3.196634 |
| C | -3.779188 | 2.261326 | 0.31247 |
| H | -3.656992 | 2.426781 | 1.389871 |
| H | -3.678329 | 3.235281 | -0.188004 |
| H | -4.792974 | 1.880673 | 0.122056 |
| H | -2.814425 | 1.329534 | -1.3564 |
| O | -3.925339 | -0.638151 | -0.888477 |
| C | -5.0082 | -1.49301 | -0.501119 |
| H | -4.661818 | -2.403871 | 0.004149 |
| H | -5.708787 | -0.954809 | 0.154462 |
| H | -5.511168 | -1.761444 | -1.438953 |
| O | -1.048868 | -0.51571 | -2.520383 |
| H | -1.50003 | -1.355542 | -2.742457 |

**Int4**

B3LYP-D3BJ/Def2-SVP Electronic Energy: -1159.398890 a.u.

B3LYP-D3BJ/Def2-SVP Gibbs free Energy: -1158.923582 a.u.

B3LYP-D3BJ/Def2-TZVP Electronic Energy: -1160.676482 a.u.

| | | | |
|---|---|---|---|
| C | -2.890781 | 0.201358 | -0.103519 |
| O | -3.904988 | 1.109913 | -0.327344 |
| C | -2.224352 | -0.329743 | -1.393871 |
| O | -1.04199 | -2.396259 | -1.638891 |
| C | -1.184981 | -1.368162 | -1.023273 |
| C | -0.214576 | -1.085786 | 0.182758 |
| C | -0.99112 | -0.433106 | 1.248078 |
| C | -1.773879 | 0.77861 | 0.907956 |
| C | -0.821625 | 1.749289 | 0.225768 |
| H | -1.135455 | 2.794383 | 0.242456 |
| C | 0.321752 | 1.400995 | -0.379993 |
| C | 0.846112 | -0.018326 | -0.396164 |
| H | 1.006724 | -0.313961 | -1.444163 |
| C | 2.190286 | -0.124114 | 0.307197 |
| C | 3.214677 | -0.698113 | -0.350621 |
| H | 3.016804 | -1.052492 | -1.370097 |
| C | 4.6224 | -0.90081 | 0.121149 |
| H | 4.901089 | -1.951493 | -0.075535 |
| H | 4.709675 | -0.750483 | 1.208966 |
| C | 5.649911 | 0.008666 | -0.590452 |
| H | 5.519371 | -0.131624 | -1.67989 |
| C | 5.408296 | 1.485474 | -0.270942 |
| H | 4.390238 | 1.800435 | -0.548342 |
| H | 6.118948 | 2.130604 | -0.813252 |
| H | 5.536532 | 1.679972 | 0.80815 |
| C | 7.076057 | -0.408898 | -0.226783 |
| H | 7.820876 | 0.214609 | -0.748254 |
| H | 7.269834 | -1.46076 | -0.494344 |
| H | 7.252411 | -0.301951 | 0.857788 |
| C | 2.276948 | 0.455476 | 1.691993 |
| H | 3.251779 | 0.270484 | 2.159951 |
| H | 1.504322 | 0.038467 | 2.35877 |
| H | 2.115323 | 1.546308 | 1.67173 |
| C | 0.440779 | -2.38904 | 0.624959 |

| | | | |
|---|---|---|---|
| H | -0.329183 | -3.124623 | 0.897608 |
| H | 1.094244 | -2.227906 | 1.490171 |
| H | 1.041059 | -2.798312 | -0.195473 |
| C | 1.179404 | 2.408375 | -1.0886 |
| H | 0.729424 | 3.410657 | -1.063082 |
| H | 1.324182 | 2.112918 | -2.141896 |
| H | 2.183541 | 2.462487 | -0.637493 |
| C | -2.459168 | 1.412445 | 2.117328 |
| H | -3.180731 | 0.721237 | 2.578729 |
| H | -3.009089 | 2.308055 | 1.803195 |
| H | -1.713525 | 1.721134 | 2.866513 |
| C | -3.678188 | 2.299619 | -1.07617 |
| H | -3.010329 | 2.13456 | -1.93445 |
| H | -3.260185 | 3.094745 | -0.439968 |
| H | -4.660838 | 2.622358 | -1.447072 |
| C | -3.223008 | -0.884598 | -2.404189 |
| H | -2.702496 | -1.169101 | -3.329182 |
| H | -3.728833 | -1.778376 | -2.012162 |
| H | -3.981558 | -0.130306 | -2.652786 |
| H | -1.663082 | 0.498203 | -1.855723 |
| O | -3.430546 | -0.893586 | 0.602612 |
| C | -4.839049 | -1.11093 | 0.632144 |
| H | -5.356249 | -0.327329 | 1.20664 |
| H | -5.275562 | -1.168714 | -0.375652 |
| H | -4.974721 | -2.076995 | 1.137826 |
| O | -0.975631 | -0.948223 | 2.411671 |
| H | -1.53466 | -0.464031 | 3.057419 |

**Int4'**

B3LYP-D3BJ/Def2-SVP Electronic Energy: -1159.390561 a.u.

B3LYP-D3BJ/Def2-SVP Gibbs free Energy: -1158.914791 a.u.

B3LYP-D3BJ/Def2-TZVP Electronic Energy: -1160.668489 a.u.

| | | | |
|---|---|---|---|
| C | -3.002919 | -0.178216 | 0.011814 |
| O | -3.469099 | -0.686535 | 1.204163 |
| C | -2.699733 | 1.346615 | -0.021901 |
| O | -0.90619 | 2.528883 | 1.068552 |
| C | -1.26159 | 1.693209 | 0.274271 |
| C | -0.200464 | 0.991651 | -0.650373 |
| C | -0.895474 | -0.132679 | -1.310264 |
| C | -1.641994 | -1.05135 | -0.441262 |
| C | -0.813198 | -1.336853 | 0.797569 |
| H | -1.210085 | -2.14511 | 1.41496 |
| C | 0.325565 | -0.720151 | 1.143271 |
| C | 0.899639 | 0.412119 | 0.320334 |
| H | 1.109233 | 1.252771 | 0.99837 |
| C | 2.20201 | 0.043609 | -0.368392 |
| C | 3.302193 | 0.767395 | -0.092437 |
| H | 3.19296 | 1.589697 | 0.626258 |
| C | 4.689904 | 0.580141 | -0.627082 |
| H | 5.041601 | 1.552067 | -1.017717 |
| H | 4.70311 | -0.121635 | -1.476264 |
| C | 5.699749 | 0.097549 | 0.438468 |
| H | 5.630002 | 0.78944 | 1.298817 |
| C | 5.361757 | -1.310041 | 0.934666 |
| H | 4.335771 | -1.366301 | 1.331539 |
| H | 6.049909 | -1.625302 | 1.736164 |
| H | 5.444597 | -2.04537 | 0.115457 |
| C | 7.127942 | 0.162246 | -0.1063 |
| H | 7.860724 | -0.159843 | 0.651856 |
| H | 7.394671 | 1.184922 | -0.420295 |
| H | 7.244153 | -0.497868 | -0.983584 |
| C | 2.16465 | -1.128684 | -1.310609 |
| H | 3.168261 | -1.491977 | -1.565568 |
| H | 1.660292 | -0.868734 | -2.256802 |
| H | 1.603505 | -1.968631 | -0.870464 |
| C | 0.353288 | 2.04097 | -1.615431 |
| H | -0.457249 | 2.505071 | -2.196828 |
| H | 1.070008 | 1.589352 | -2.313693 |
| H | 0.866782 | 2.821828 | -1.039141 |
| C | 1.104297 | -1.106505 | 2.364431 |
| H | 0.621348 | -1.927755 | 2.912567 |
| H | 1.212448 | -0.242415 | 3.042371 |
| H | 2.125774 | -1.419207 | 2.090042 |
| C | -2.061071 | -2.358631 | -1.111494 |
| H | -2.709169 | -2.193877 | -1.983874 |
| H | -2.619541 | -2.971093 | -0.391324 |
| H | -1.169811 | -2.92668 | -1.41817 |
| C | -3.021712 | -0.254639 | 2.490609 |
| H | -3.874834 | 0.192923 | 3.023005 |
| H | -2.202519 | 0.471105 | 2.44213 |
| H | -2.671067 | -1.133758 | 3.049255 |
| C | -3.724631 | 2.209571 | 0.700025 |
| H | -3.602392 | 2.190014 | 1.789908 |
| H | -3.62165 | 3.254225 | 0.372171 |
| H | -4.740461 | 1.87129 | 0.450711 |
| H | -2.772476 | 1.564539 | -1.103885 |
| O | -3.980067 | -0.378919 | -0.965375 |
| C | -5.107054 | -1.208047 | -0.688658 |
| H | -4.822974 | -2.225102 | -0.382502 |
| H | -5.751144 | -0.76597 | 0.086691 |
| H | -5.664606 | -1.261279 | -1.634039 |
| O | -0.880753 | -0.195955 | -2.583828 |
| H | -1.363218 | -0.966797 | -2.953737 |

**1-1**

B3LYP-D3BJ/Def2-SVP Electronic Energy: -1158.976803 a.u.

B3LYP-D3BJ/Def2-SVP Gibbs free Energy: -1158.514215 a.u.

B3LYP-D3BJ/Def2-TZVP Electronic Energy: -1160.256001 a.u.

| | | | |
|---|---|---|---|
| C | -2.886871 | 0.214191 | -0.077995 |
| O | -3.902451 | 1.122128 | -0.379235 |
| C | -2.236803 | -0.399898 | -1.342599 |
| O | -1.156619 | -2.523479 | -1.468438 |
| C | -1.219503 | -1.442967 | -0.914457 |
| C | -0.230846 | -1.106916 | 0.224412 |

| | | | |
|---|---|---|---|
| C | -0.978395 | -0.396422 | 1.344821 |
| C | -1.783816 | 0.810614 | 0.88808 |
| C | -0.820439 | 1.736228 | 0.169245 |
| H | -1.103529 | 2.791299 | 0.135862 |
| C | 0.320499 | 1.349419 | -0.421867 |
| C | 0.823353 | -0.082295 | -0.39333 |
| H | 0.98703 | -0.400628 | -1.436084 |
| C | 2.18294 | -0.162175 | 0.295296 |
| C | 3.213228 | -0.721945 | -0.364309 |
| H | 3.015677 | -1.084138 | -1.381556 |
| C | 4.627921 | -0.904603 | 0.101518 |
| H | 4.927507 | -1.946873 | -0.109345 |
| H | 4.714363 | -0.770234 | 1.191785 |
| C | 5.639286 | 0.033834 | -0.595054 |
| H | 5.497751 | -0.077005 | -1.686695 |
| C | 5.38831 | 1.499069 | -0.232159 |
| H | 4.360405 | 1.808168 | -0.477281 |
| H | 6.078309 | 2.167195 | -0.77352 |
| H | 5.539488 | 1.667374 | 0.848352 |
| C | 7.074213 | -0.37808 | -0.259864 |
| H | 7.806942 | 0.270589 | -0.767846 |
| H | 7.277493 | -1.418441 | -0.563194 |
| H | 7.260557 | -0.304418 | 0.825884 |
| C | 2.277328 | 0.427596 | 1.676386 |
| H | 3.276106 | 0.306814 | 2.114232 |
| H | 1.549157 | -0.030159 | 2.363858 |
| H | 2.047591 | 1.506266 | 1.656133 |
| C | 0.425351 | -2.393534 | 0.713212 |
| H | -0.344179 | -3.108704 | 1.038676 |
| H | 1.091805 | -2.199892 | 1.56155 |
| H | 1.010518 | -2.857794 | -0.090543 |
| C | 1.19676 | 2.323007 | -1.156977 |
| H | 0.754143 | 3.329319 | -1.17888 |
| H | 1.357873 | 1.988346 | -2.196683 |
| H | 2.195447 | 2.39262 | -0.695387 |
| C | -2.449934 | 1.510879 | 2.069773 |
| H | -3.141501 | 0.831775 | 2.586631 |
| H | -3.010705 | 2.391947 | 1.728738 |
| H | -1.689732 | 1.842295 | 2.792075 |
| C | -3.630181 | 2.273218 | -1.161679 |
| H | -2.970627 | 2.05662 | -2.016259 |
| H | -3.180858 | 3.078023 | -0.558787 |
| H | -4.598587 | 2.623706 | -1.547057 |
| C | -3.247605 | -0.972277 | -2.329168 |
| H | -2.737376 | -1.315977 | -3.240175 |
| H | -3.778788 | -1.833376 | -1.898734 |
| H | -3.987049 | -0.212199 | -2.615907 |
| H | -1.654861 | 0.393854 | -1.837563 |
| O | -3.475709 | -0.839627 | 0.665409 |
| C | -4.886333 | -1.00695 | 0.685263 |
| H | -5.390446 | -0.183908 | 1.216412 |
| H | -5.318814 | -1.098133 | -0.32279 |
| H | -5.064303 | -1.944002 | 1.232547 |
| O | -0.911169 | -0.749058 | 2.506045 |

**1-1’**

B3LYP-D3BJ/Def2-SVP Electronic Energy: -1158.969680 a.u.

B3LYP-D3BJ/Def2-SVP Gibbs free Energy: -1158.506695 a.u.

B3LYP-D3BJ/Def2-TZVP Electronic Energy: -1160.249213 a.u.

| | | | |
|---|---|---|---|
| C | -2.997073 | -0.194483 | -0.018147 |
| O | -3.512271 | -0.685504 | 1.185541 |
| C | -2.703014 | 1.340792 | -0.019102 |
| O | -0.951202 | 2.477186 | 1.173275 |
| C | -1.270238 | 1.679133 | 0.314032 |
| C | -0.209488 | 1.027069 | -0.603639 |
| C | -0.895064 | -0.087908 | -1.376779 |
| C | -1.697522 | -1.017646 | -0.480822 |
| C | -0.839684 | -1.372816 | 0.719774 |
| H | -1.210957 | -2.217183 | 1.307317 |
| C | 0.310593 | -0.783055 | 1.081324 |
| C | 0.880525 | 0.404505 | 0.330167 |
| H | 1.100561 | 1.189809 | 1.069893 |
| C | 2.194815 | 0.075276 | -0.365362 |
| C | 3.293055 | 0.782163 | -0.042177 |
| H | 3.179352 | 1.560801 | 0.723434 |
| C | 4.684643 | 0.631065 | -0.582548 |
| H | 5.041312 | 1.627292 | -0.901321 |
| H | 4.700392 | -0.006076 | -1.481398 |
| C | 5.691217 | 0.06927 | 0.446484 |
| H | 5.615746 | 0.691793 | 1.358037 |
| C | 5.355703 | -1.373545 | 0.830867 |
| H | 4.327115 | -1.463536 | 1.214335 |
| H | 6.039304 | -1.74749 | 1.61089 |
| H | 5.447369 | -2.043531 | -0.041721 |
| C | 7.122279 | 0.179066 | -0.08375 |
| H | 7.852516 | -0.200993 | 0.64977 |
| H | 7.388587 | 1.223853 | -0.314474 |
| H | 7.244156 | -0.408904 | -1.010287 |
| C | 2.177054 | -1.043992 | -1.372207 |
| H | 3.185049 | -1.406889 | -1.610741 |
| H | 1.701964 | -0.729298 | -2.315648 |
| H | 1.589521 | -1.897215 | -0.997113 |
| C | 0.350079 | 2.118162 | -1.518476 |
| H | -0.458239 | 2.605773 | -2.084982 |
| H | 1.06262 | 1.693965 | -2.23813 |
| H | 0.866968 | 2.880807 | -0.919103 |
| C | 1.108985 | -1.254841 | 2.261023 |
| H | 0.622786 | -2.097824 | 2.773219 |
| H | 1.250996 | -0.435456 | 2.987347 |
| H | 2.11877 | -1.572235 | 1.949756 |
| C | -2.082442 | -2.298782 | -1.221292 |
| H | -2.677247 | -2.079148 | -2.116356 |
| H | -2.656921 | -2.96555 | -0.562829 |
| H | -1.17267 | -2.833378 | -1.533536 |

| | | | |
|---|---|---|---|
| C | -2.991396 | -0.32852 | 2.461328 |
| H | -3.822713 | 0.04787 | 3.07884 |
| H | -2.21143 | 0.440087 | 2.412211 |
| H | -2.563614 | -1.217013 | 2.9497 |
| C | -3.744026 | 2.178748 | 0.708611 |
| H | -3.651611 | 2.11339 | 1.800331 |
| H | -3.631989 | 3.237261 | 0.430426 |
| H | -4.754746 | 1.856071 | 0.420531 |
| H | -2.764719 | 1.577773 | -1.096825 |
| O | -4.01439 | -0.339034 | -0.986386 |
| C | -5.100517 | -1.224278 | -0.749225 |
| H | -4.778356 | -2.241987 | -0.48361 |
| H | -5.770093 | -0.848009 | 0.040585 |
| H | -5.657837 | -1.266767 | -1.696473 |
| O | -0.819993 | -0.215195 | -2.584002 |

**1**

B3LYP-D3BJ/Def2-SVP Electronic Energy: -1043.312102 a.u.
B3LYP-D3BJ/Def2-SVP Gibbs free Energy: -1042.903338 a.u.
B3LYP-D3BJ/Def2-TZVP Electronic Energy: -1044.461650 a.u.

| | | | |
|---|---|---|---|
| C | 3.185572 | -0.102901 | -0.045521 |
| O | 4.418588 | -0.651528 | -0.143208 |
| C | 2.931587 | 1.110248 | -0.602593 |
| O | 1.28428 | 2.697406 | -1.120162 |
| C | 1.594671 | 1.70358 | -0.472801 |
| C | 0.559492 | 1.096496 | 0.505123 |
| C | 1.281086 | 0.154786 | 1.442013 |
| C | 2.116376 | -0.897561 | 0.717099 |
| C | 1.22213 | -1.597642 | -0.301104 |
| H | 1.581338 | -2.571586 | -0.647689 |
| C | 0.097074 | -1.076598 | -0.811938 |
| C | -0.465783 | 0.260369 | -0.362577 |
| H | -0.656036 | 0.867206 | -1.261759 |
| C | -1.817098 | 0.077038 | 0.324217 |
| C | -2.886477 | 0.726288 | -0.170414 |
| H | -2.723478 | 1.352719 | -1.057124 |
| C | -4.303414 | 0.697229 | 0.321907 |
| H | -4.643872 | 1.740272 | 0.45461 |
| H | -4.376946 | 0.218539 | 1.311517 |
| C | -5.281015 | -0.005383 | -0.646374 |
| H | -5.155162 | 0.461599 | -1.641458 |
| C | -4.962845 | -1.496125 | -0.780638 |
| H | -3.923544 | -1.661485 | -1.10506 |
| H | -5.626218 | -1.980945 | -1.515785 |
| H | -5.09831 | -2.014888 | 0.184447 |
| C | -6.728995 | 0.211433 | -0.202143 |
| H | -7.438236 | -0.265652 | -0.89862 |
| H | -6.978913 | 1.284014 | -0.148754 |
| H | -6.902255 | -0.221979 | 0.798438 |
| C | -1.858243 | -0.847322 | 1.510729 |
| H | -2.881708 | -1.034783 | 1.859242 |
| H | -1.284557 | -0.440145 | 2.359012 |
| H | -1.399422 | -1.819154 | 1.265434 |
| C | -0.119543 | 2.243616 | 1.253557 |
| H | 0.631449 | 2.860414 | 1.770221 |
| H | -0.823441 | 1.864003 | 2.004114 |
| H | -0.668374 | 2.881371 | 0.548864 |
| C | -0.712226 | -1.803598 | -1.847079 |
| H | -0.221124 | -2.733777 | -2.167936 |
| H | -0.872057 | -1.165873 | -2.733879 |
| H | -1.714295 | -2.055815 | -1.461293 |
| C | 2.728811 | -1.882951 | 1.712631 |
| H | 3.404686 | -1.372196 | 2.412739 |
| H | 3.291302 | -2.670702 | 1.194496 |
| H | 1.927364 | -2.361603 | 2.293628 |
| C | 4.596842 | -1.702561 | -1.103331 |
| H | 4.322503 | -1.349881 | -2.110706 |
| H | 3.998507 | -2.592006 | -0.852915 |
| H | 5.662717 | -1.964325 | -1.080646 |
| C | 3.932217 | 1.86308 | -1.429904 |
| H | 3.613358 | 1.903703 | -2.484925 |
| H | 4.015655 | 2.906164 | -1.084896 |
| H | 4.922324 | 1.392934 | -1.381816 |
| O | 1.216561 | 0.223462 | 2.652536 |

**10v**

B3LYP-D3BJ/Def2-SVP Electronic Energy: -1283.352565 a.u.
B3LYP-D3BJ/Def2-SVP Gibbs free Energy: -1282.979135 a.u.
B3LYP-D3BJ/Def2-TZVP Electronic Energy: -1284.776885 a.u.

| | | | |
|---|---|---|---|
| O | -2.719851 | -0.883335 | -0.69517 |
| C | -2.505711 | -1.989263 | 0.015283 |
| O | -3.188288 | -3.037581 | -0.408489 |
| C | -1.667277 | -2.08267 | 1.102307 |
| O | -0.416801 | -0.866033 | 2.671972 |
| C | -1.086965 | -0.881089 | 1.630991 |
| C | -1.371923 | 0.449194 | 0.914976 |
| C | -1.961761 | 0.258226 | -0.460182 |
| C | -1.891485 | 1.193942 | -1.426251 |
| C | -1.090213 | 2.393503 | -1.15538 |
| H | -1.189365 | 3.216475 | -1.866772 |
| C | -0.236574 | 2.484431 | -0.115648 |
| C | -0.061397 | 1.28738 | 0.807969 |
| C | -2.39802 | 1.208659 | 1.794057 |
| H | -3.345393 | 0.650565 | 1.843463 |
| H | -2.599392 | 2.20533 | 1.375831 |
| H | -2.003261 | 1.32254 | 2.814756 |
| C | 0.62184 | 3.690471 | 0.195678 |
| H | 1.609954 | 3.296653 | 0.49959 |
| C | -2.562107 | 1.071514 | -2.763109 |
| H | -3.14623 | 0.147168 | -2.857407 |
| H | -1.81324 | 1.094658 | -3.573786 |
| H | -3.236495 | 1.928199 | -2.933239 |
| C | -3.946706 | -2.958633 | -1.626784 |
| H | -4.391271 | -3.952435 | -1.75645 |

| | | | |
|---|---|---|---|
| H | -3.289543 | -2.726601 | -2.47738 |
| H | -4.738144 | -2.200347 | -1.544195 |
| C | -1.455936 | -3.387711 | 1.818571 |
| H | -2.156774 | -3.514912 | 2.663132 |
| H | -1.587553 | -4.245643 | 1.144247 |
| H | -0.438448 | -3.424653 | 2.235656 |
| H | 0.143062 | 1.649382 | 1.822926 |
| C | 1.16026 | 0.480098 | 0.384812 |
| C | 2.160264 | 0.188389 | 1.32716 |
| C | 1.320414 | 0.01805 | -0.93358 |
| C | 3.288209 | -0.549134 | 0.978576 |
| H | 2.048547 | 0.541176 | 2.353482 |
| C | 2.435037 | -0.730045 | -1.300525 |
| H | 0.568781 | 0.248299 | -1.687366 |
| C | 3.407008 | -1.005105 | -0.335595 |
| H | 4.065476 | -0.771652 | 1.708468 |
| H | 2.558201 | -1.093837 | -2.319798 |
| O | 5.432324 | -2.011126 | 0.13983 |
| O | 4.671704 | -2.182697 | -1.869472 |
| N | 4.58345 | -1.785712 | -0.713937 |
| C | 0.048942 | 4.455761 | 1.40295 |
| H | -0.094016 | 3.800938 | 2.276392 |
| H | 0.726897 | 5.271377 | 1.7028 |
| H | -0.928432 | 4.898828 | 1.149405 |
| C | 0.834564 | 4.630917 | -0.990394 |
| H | -0.107773 | 5.110403 | -1.301964 |
| H | 1.536234 | 5.435376 | -0.718133 |
| H | 1.248556 | 4.097566 | -1.860759 |

**Ints1**

B3LYP-D3BJ/Def2-SVP Electronic Energy: -1283.798255 a.u.

B3LYP-D3BJ/Def2-SVP Gibbs free Energy: -1283.409725 a.u.

B3LYP-D3BJ/Def2-TZVP Electronic Energy: -1285.220329 a.u.

| | | | |
|---|---|---|---|
| O | -2.673845 | -0.938111 | -0.723103 |
| C | -2.437142 | -2.021661 | -0.039419 |
| O | -3.06053 | -3.087907 | -0.421616 |
| C | -1.564962 | -2.092694 | 1.074687 |
| O | -0.39922 | -0.789462 | 2.672049 |
| C | -1.095572 | -0.893436 | 1.562115 |
| C | -1.390777 | 0.438192 | 0.913684 |
| C | -1.970206 | 0.244034 | -0.466356 |
| C | -1.936337 | 1.187254 | -1.423329 |
| C | -1.157474 | 2.399366 | -1.148313 |
| H | -1.27491 | 3.224421 | -1.853401 |
| C | -0.291709 | 2.491181 | -0.118927 |
| C | -0.087116 | 1.297047 | 0.802866 |
| C | -2.423661 | 1.175541 | 1.807007 |
| H | -3.361147 | 0.602311 | 1.854088 |
| H | -2.635746 | 2.16714 | 1.384198 |
| H | -2.028263 | 1.299277 | 2.825516 |
| C | 0.559298 | 3.703785 | 0.184474 |
| H | 1.546536 | 3.317997 | 0.499624 |
| C | -2.608238 | 1.056273 | -2.756268 |
| H | -3.180897 | 0.125277 | -2.852154 |
| H | -1.857944 | 1.093284 | -3.564437 |
| H | -3.290042 | 1.907805 | -2.918549 |
| C | -3.928201 | -3.055182 | -1.581112 |
| H | -4.32953 | -4.070472 | -1.663767 |
| H | -3.341693 | -2.793776 | -2.472095 |
| H | -4.734639 | -2.32759 | -1.420522 |
| C | -1.298666 | -3.420775 | 1.728243 |
| H | -2.082711 | -3.683926 | 2.457501 |
| H | -1.256372 | -4.2197 | 0.975297 |
| H | -0.329744 | -3.415969 | 2.251041 |
| H | 0.112728 | 1.667699 | 1.815589 |
| C | 1.145425 | 0.509116 | 0.372924 |
| C | 2.157509 | 0.244315 | 1.309497 |
| C | 1.299373 | 0.039347 | -0.943044 |
| C | 3.293727 | -0.479181 | 0.956831 |
| H | 2.052401 | 0.609569 | 2.332426 |
| C | 2.422837 | -0.693995 | -1.312925 |
| H | 0.538886 | 0.251013 | -1.693426 |
| C | 3.406329 | -0.944902 | -0.353702 |
| H | 4.082097 | -0.681328 | 1.680657 |
| H | 2.543397 | -1.064307 | -2.330113 |
| O | 5.448651 | -1.920248 | 0.114324 |
| O | 4.677311 | -2.114466 | -1.889031 |
| N | 4.592712 | -1.7124 | -0.735824 |
| C | -0.029348 | 4.477379 | 1.37921 |
| H | -0.172342 | 3.832667 | 2.26016 |
| H | 0.640764 | 5.301669 | 1.671989 |
| H | -1.008196 | 4.90923 | 1.113246 |
| C | 0.774006 | 4.631203 | -1.011376 |
| H | -0.167602 | 5.106342 | -1.331127 |
| H | 1.473551 | 5.438928 | -0.744171 |
| H | 1.192289 | 4.088647 | -1.873847 |
| H | -0.253376 | -1.651107 | 3.105444 |

**Ints2**

B3LYP-D3BJ/Def2-SVP Electronic Energy: -1399.433170 a.u.

B3LYP-D3BJ/Def2-SVP Gibbs free Energy: -1398.991974 a.u.

B3LYP-D3BJ/Def2-TZVP Electronic Energy: -1400.987728 a.u.

| | | | |
|---|---|---|---|
| O | 1.929154 | -2.843956 | 0.617106 |
| C | -1.246449 | -1.76956 | 0.231255 |
| O | -2.535209 | -1.635822 | 0.268976 |
| C | -0.71107 | -2.038161 | -1.074115 |
| O | 0.841433 | -1.907186 | -2.795935 |
| C | 0.562489 | -1.70444 | -1.513453 |
| C | 1.722251 | -0.966936 | -0.836425 |
| C | 2.06768 | -1.495983 | 0.543157 |
| C | 2.63123 | -0.702797 | 1.483177 |
| C | 2.858743 | 0.709228 | 1.166692 |
| H | 3.488506 | 1.260184 | 1.870083 |
| C | 2.375253 | 1.332439 | 0.074034 |

| | | | |
|---|---|---|---|
| C | 1.423958 | 0.59084 | -0.844393 |
| C | 3.023346 | -1.169092 | -1.659431 |
| H | 3.287955 | -2.234775 | -1.702697 |
| H | 3.837079 | -0.62273 | -1.165137 |
| H | 2.920492 | -0.78579 | -2.682296 |
| C | 2.658283 | 2.773778 | -0.300428 |
| H | 1.701504 | 3.201898 | -0.653881 |
| C | 3.144946 | -1.210302 | 2.805271 |
| H | 2.876247 | -2.257704 | 3.01337 |
| H | 2.750347 | -0.605063 | 3.638942 |
| H | 4.246087 | -1.140081 | 2.856865 |
| C | -3.426288 | -1.624233 | 1.409228 |
| H | -4.41563 | -1.822082 | 0.981307 |
| H | -3.418406 | -0.634104 | 1.877988 |
| H | -3.159822 | -2.417981 | 2.115496 |
| C | -1.697821 | -2.558285 | -2.100608 |
| H | -1.253822 | -3.36279 | -2.711782 |
| H | -2.580087 | -2.993597 | -1.618783 |
| H | -2.047011 | -1.760199 | -2.779057 |
| H | 1.62109 | 0.893298 | -1.882629 |
| C | -0.018152 | 1.001411 | -0.575438 |
| C | -0.941761 | 1.010333 | -1.636252 |
| C | -0.460908 | 1.359397 | 0.705717 |
| C | -2.282612 | 1.310325 | -1.422919 |
| H | -0.605589 | 0.751267 | -2.642707 |
| C | -1.799288 | 1.662635 | 0.943006 |
| H | 0.248457 | 1.384851 | 1.532485 |
| C | -2.69699 | 1.611662 | -0.123297 |
| H | -3.004237 | 1.289725 | -2.23862 |
| H | -2.148882 | 1.924114 | 1.940897 |
| O | -4.865977 | 1.984818 | -0.822686 |
| O | -4.494656 | 1.886766 | 1.299134 |
| N | -4.115144 | 1.848087 | 0.133845 |
| C | 3.645975 | 2.841126 | -1.479405 |
| H | 3.304619 | 2.241916 | -2.337979 |
| H | 3.771201 | 3.880084 | -1.826234 |
| H | 4.635223 | 2.460347 | -1.175257 |
| C | 3.14921 | 3.635406 | 0.86299 |
| H | 4.134969 | 3.302229 | 1.226784 |
| H | 3.256438 | 4.684109 | 0.542774 |
| H | 2.447837 | 3.609134 | 1.712067 |
| H | 0.094128 | -2.307937 | -3.273098 |
| O | -0.460833 | -1.708753 | 1.248597 |
| C | -0.725121 | -1.308912 | 2.612044 |
| H | -1.129836 | -2.166541 | 3.162529 |
| H | 0.2569 | -1.034179 | 3.010796 |
| H | -1.400755 | -0.451232 | 2.649851 |
| H | 2.238284 | -3.169187 | 1.477022 |

**Ints3**

B3LYP-D3BJ/Def2-SVP Electronic Energy: -1399.437143 a.u.

B3LYP-D3BJ/Def2-SVP Gibbs free Energy: -1398.996233 a.u.

B3LYP-D3BJ/Def2-TZVP Electronic Energy: -1400.993196 a.u.

| | | | |
|---|---|---|---|
| O | 2.030559 | -2.807581 | 0.5134 |
| C | -1.178977 | -2.011004 | 0.203193 |
| O | -2.365831 | -1.591932 | -0.003728 |
| C | -0.430654 | -2.52098 | -0.998936 |
| O | 0.526796 | -1.391389 | -2.848014 |
| C | 0.609993 | -1.559595 | -1.652827 |
| C | 1.77164 | -0.884678 | -0.889938 |
| C | 2.07788 | -1.447962 | 0.482358 |
| C | 2.534371 | -0.668959 | 1.491164 |
| C | 2.778958 | 0.751612 | 1.228633 |
| H | 3.365761 | 1.284545 | 1.981096 |
| C | 2.358922 | 1.391462 | 0.119915 |
| C | 1.447757 | 0.65574 | -0.841543 |
| C | 3.053186 | -1.081386 | -1.737254 |
| H | 3.329827 | -2.145139 | -1.768071 |
| H | 3.878136 | -0.514117 | -1.283957 |
| H | 2.895892 | -0.723249 | -2.763409 |
| C | 2.658558 | 2.835845 | -0.222055 |
| H | 1.714616 | 3.273982 | -0.597945 |
| C | 2.932965 | -1.220687 | 2.834762 |
| H | 2.502511 | -2.2141 | 3.038687 |
| H | 2.590049 | -0.5524 | 3.641426 |
| H | 4.03015 | -1.305155 | 2.934274 |
| C | -3.472268 | -1.500794 | 0.945899 |
| H | -4.354886 | -1.375233 | 0.310822 |
| H | -3.349378 | -0.634258 | 1.600229 |
| H | -3.528761 | -2.438939 | 1.509156 |
| C | -1.392355 | -3.067085 | -2.050843 |
| H | -0.820644 | -3.578747 | -2.835867 |
| H | -2.083638 | -3.790031 | -1.594788 |
| H | -1.976726 | -2.261182 | -2.515093 |
| H | 1.636141 | 1.010578 | -1.863645 |
| C | -0.012137 | 1.005246 | -0.553428 |
| C | -0.920017 | 1.134846 | -1.620056 |
| C | -0.469259 | 1.248005 | 0.752426 |
| C | -2.252499 | 1.469471 | -1.396922 |
| H | -0.577568 | 0.970031 | -2.64244 |
| C | -1.79605 | 1.588437 | 0.996063 |
| H | 0.226734 | 1.180416 | 1.587888 |
| C | -2.673935 | 1.68499 | -0.084574 |
| H | -2.957562 | 1.561422 | -2.222169 |
| H | -2.15091 | 1.778661 | 2.008198 |
| O | -4.80314 | 2.24541 | -0.784266 |
| O | -4.47193 | 1.989509 | 1.331237 |
| N | -4.07872 | 1.999443 | 0.170424 |
| C | 3.682546 | 2.915967 | -1.368798 |
| H | 3.363893 | 2.33304 | -2.247097 |
| H | 3.824049 | 3.959725 | -1.693967 |
| H | 4.659766 | 2.524317 | -1.040586 |
| C | 3.117254 | 3.673268 | 0.971557 |
| H | 4.087116 | 3.32403 | 1.362129 |

| | | | |
|---|---|---|---|
| H | 3.244206 | 4.726242 | 0.673665 |
| H | 2.387316 | 3.638332 | 1.79589 |
| O | -0.591778 | -2.123568 | 1.327422 |
| C | -0.991348 | -1.642661 | 2.643647 |
| H | -1.735592 | -2.329285 | 3.061486 |
| H | -0.066585 | -1.67294 | 3.228544 |
| H | -1.366529 | -0.618874 | 2.571752 |
| H | 2.376191 | -3.142857 | 1.355545 |
| H | 0.202874 | -3.323106 | -0.58779 |

**Ints3'**

B3LYP-D3BJ/Def2-SVP Electronic Energy: -1399.433815 a.u.

B3LYP-D3BJ/Def2-SVP Gibbs free Energy: -1398.992396 a.u.

B3LYP-D3BJ/Def2-TZVP Electronic Energy: -1400.990923 a.u.

| | | | |
|---|---|---|---|
| O | 2.15678 | -2.672584 | 0.387188 |
| C | -1.14174 | -1.895216 | 0.110439 |
| O | -2.39095 | -1.634506 | 0.037765 |
| C | -0.507777 | -2.196958 | -1.215645 |
| O | 0.920877 | -1.604529 | -2.938671 |
| C | 0.780018 | -1.503498 | -1.737795 |
| C | 1.834765 | -0.717089 | -0.927711 |
| C | 2.142747 | -1.314517 | 0.4274 |
| C | 2.534049 | -0.549798 | 1.473027 |
| C | 2.664952 | 0.895608 | 1.269862 |
| H | 3.195492 | 1.443749 | 2.052625 |
| C | 2.20331 | 1.548107 | 0.184962 |
| C | 1.3733 | 0.783999 | -0.828696 |
| C | 3.160324 | -0.747862 | -1.722961 |
| H | 3.535699 | -1.77854 | -1.796031 |
| H | 3.908823 | -0.134052 | -1.203517 |
| H | 3.019746 | -0.350549 | -2.736659 |
| C | 2.376746 | 3.028943 | -0.083202 |
| H | 1.403639 | 3.396933 | -0.459787 |
| C | 2.960041 | -1.117757 | 2.800916 |
| H | 2.657833 | -2.167635 | 2.939492 |
| H | 2.513289 | -0.542556 | 3.628608 |
| H | 4.055831 | -1.066839 | 2.931955 |
| C | -3.396978 | -1.628914 | 1.097112 |
| H | -4.341862 | -1.495077 | 0.561397 |
| H | -3.225709 | -0.793659 | 1.781111 |
| H | -3.37601 | -2.596791 | 1.609743 |
| C | -0.33415 | -3.736666 | -1.296633 |
| H | 0.417741 | -4.08403 | -0.578234 |
| H | -1.295559 | -4.230446 | -1.093567 |
| H | -0.016899 | -3.992573 | -2.316607 |
| H | 1.560223 | 1.194203 | -1.830821 |
| C | -0.114439 | 1.017207 | -0.575202 |
| C | -1.002443 | 1.058264 | -1.665111 |
| C | -0.619389 | 1.249939 | 0.715283 |
| C | -2.360508 | 1.299149 | -1.478936 |
| H | -0.622969 | 0.899519 | -2.676651 |
| C | -1.97268 | 1.500302 | 0.92207 |
| H | 0.060043 | 1.246414 | 1.566992 |
| C | -2.827805 | 1.510132 | -0.180995 |
| H | -3.051282 | 1.321033 | -2.320928 |
| H | -2.364009 | 1.684638 | 1.921633 |
| O | -4.973151 | 1.906783 | -0.941591 |
| O | -4.679416 | 1.695809 | 1.184592 |
| N | -4.258317 | 1.723986 | 0.034172 |
| C | 3.410175 | 3.258734 | -1.200733 |
| H | 3.164203 | 2.693928 | -2.113476 |
| H | 3.46059 | 4.325997 | -1.472194 |
| H | 4.413166 | 2.942503 | -0.868958 |
| C | 2.73326 | 3.84739 | 1.157663 |
| H | 3.723761 | 3.570793 | 1.554497 |
| H | 2.767626 | 4.92047 | 0.910698 |
| H | 1.994404 | 3.707253 | 1.962636 |
| O | -0.441146 | -2.039883 | 1.159172 |
| C | -0.773927 | -1.792063 | 2.555643 |
| H | -1.461866 | -2.568529 | 2.905618 |
| H | 0.186476 | -1.866069 | 3.073737 |
| H | -1.191087 | -0.786405 | 2.65993 |
| H | 2.504987 | -3.041931 | 1.214124 |
| H | -1.263179 | -1.912992 | -1.958749 |

**TS2**

B3LYP-D3BJ/Def2-SVP Electronic Energy: -1399.426726 a.u.

B3LYP-D3BJ/Def2-SVP Gibbs free Energy: -1398.984530 a.u.

B3LYP-D3BJ/Def2-TZVP Electronic Energy: -1400.979904 a.u.

| | | | |
|---|---|---|---|
| O | -2.773895 | -0.108409 | 2.595224 |
| C | -2.59555 | -0.823057 | -0.77871 |
| O | -1.90845 | -0.440303 | -1.875751 |
| C | -1.847984 | -1.957317 | -0.081887 |
| O | 0.421024 | -2.193892 | 0.61408 |
| C | -0.58356 | -1.526365 | 0.635111 |
| C | -0.644856 | -0.26696 | 1.565864 |
| C | -2.069095 | 0.185401 | 1.552773 |
| C | -2.596699 | 0.758731 | 0.361834 |
| C | -1.636324 | 1.702281 | -0.28758 |
| H | -2.095008 | 2.374647 | -1.01129 |
| C | -0.336296 | 1.812541 | 0.012852 |
| C | 0.298383 | 0.90385 | 1.051621 |
| C | -0.188892 | -0.728594 | 2.958101 |
| H | -0.798471 | -1.568592 | 3.318263 |
| H | -0.268865 | 0.099499 | 3.676103 |
| H | 0.855215 | -1.05974 | 2.905935 |
| C | 0.54607 | 2.901028 | -0.581543 |
| H | 1.42468 | 2.404366 | -1.025582 |
| C | -4.010866 | 1.31101 | 0.4087 |
| H | -4.742033 | 0.624235 | 0.856031 |
| H | -4.35777 | 1.533961 | -0.608398 |
| H | -4.020825 | 2.25071 | 0.984404 |
| C | -2.595762 | 0.205602 | -2.95423 |
| H | -1.810929 | 0.563223 | -3.63182 |

| | | | |
|---|---|---|---|
| H | -3.199107 | 1.054155 | -2.604989 |
| H | -3.243103 | -0.51379 | -3.476767 |
| H | 0.465254 | 1.514672 | 1.952657 |
| C | 1.662761 | 0.401346 | 0.613274 |
| C | 2.747975 | 0.441695 | 1.498958 |
| C | 1.862905 | -0.087699 | -0.689875 |
| C | 4.007797 | -0.009415 | 1.109245 |
| H | 2.607457 | 0.835301 | 2.507476 |
| C | 3.110604 | -0.538812 | -1.099998 |
| H | 1.025034 | -0.121756 | -1.389063 |
| C | 4.169233 | -0.495472 | -0.187992 |
| H | 4.854633 | 0.017402 | 1.793723 |
| H | 3.270377 | -0.921203 | -2.107136 |
| O | 6.407358 | -0.925001 | 0.197556 |
| O | 5.61566 | -1.388893 | -1.752782 |
| N | 5.488238 | -0.968638 | -0.609976 |
| C | 1.072878 | 3.838844 | 0.519013 |
| H | 1.682442 | 3.307944 | 1.265805 |
| H | 1.707087 | 4.623095 | 0.076213 |
| H | 0.238571 | 4.331834 | 1.045501 |
| C | -0.125352 | 3.702277 | -1.696893 |
| H | -0.978299 | 4.291427 | -1.322164 |
| H | 0.597239 | 4.408556 | -2.134439 |
| H | -0.490043 | 3.05037 | -2.506107 |
| O | -3.900997 | -1.041975 | -1.058733 |
| C | -4.72072 | -1.805287 | -0.168517 |
| H | -4.552447 | -1.521642 | 0.879805 |
| H | -5.758733 | -1.582059 | -0.445053 |
| H | -4.528954 | -2.881559 | -0.297546 |
| H | -3.703573 | 0.191019 | 2.526003 |
| C | -1.593833 | -3.123508 | -1.035227 |
| H | -2.525418 | -3.413829 | -1.541089 |
| H | -0.852779 | -2.84755 | -1.797707 |
| H | -1.214336 | -3.989414 | -0.475672 |
| H | -2.487461 | -2.299761 | 0.750615 |

**TS2’**

B3LYP-D3BJ/Def2-SVP Electronic Energy: -1399.416553 a.u.

B3LYP-D3BJ/Def2-SVP Gibbs free Energy: -1398.975450 a.u.

B3LYP-D3BJ/Def2-TZVP Electronic Energy: -1400.970733 a.u.

| | | | |
|---|---|---|---|
| O | -2.801355 | -0.246981 | 2.591931 |
| C | -2.435446 | -0.84294 | -0.845785 |
| O | -1.670311 | -0.385453 | -1.846443 |
| C | -1.71076 | -1.986778 | -0.148534 |
| O | 0.445804 | -2.266653 | 0.815398 |
| C | -0.534918 | -1.569801 | 0.722794 |
| C | -0.644514 | -0.288989 | 1.622305 |
| C | -2.077428 | 0.133097 | 1.583988 |
| C | -2.593572 | 0.789694 | 0.443656 |
| C | -1.658669 | 1.775482 | -0.151738 |
| H | -2.126884 | 2.498873 | -0.81735 |
| C | -0.349107 | 1.85215 | 0.12728 |
| C | 0.295674 | 0.883169 | 1.103307 |
| C | -0.199842 | -0.703747 | 3.033874 |
| H | -0.779132 | -1.562226 | 3.399003 |
| H | -0.331804 | 0.13558 | 3.730822 |
| H | 0.858922 | -0.988301 | 3.011074 |
| C | 0.539775 | 2.961006 | -0.416631 |
| H | 1.390897 | 2.474669 | -0.92315 |
| C | -4.046762 | 1.205989 | 0.454495 |
| H | -4.718605 | 0.394942 | 0.768409 |
| H | -4.357819 | 1.517164 | -0.551125 |
| H | -4.183322 | 2.065979 | 1.132265 |
| C | -2.206789 | 0.488032 | -2.855577 |
| H | -1.421233 | 1.214346 | -3.094056 |
| H | -3.107234 | 1.006342 | -2.505706 |
| H | -2.440948 | -0.108286 | -3.749301 |
| C | -2.642744 | -2.901426 | 0.669357 |
| H | -3.202487 | -2.35819 | 1.439727 |
| H | -3.361975 | -3.393341 | 0.001205 |
| H | -2.042653 | -3.682234 | 1.157825 |
| H | 0.505025 | 1.460676 | 2.016753 |
| C | 1.635017 | 0.373101 | 0.602858 |
| C | 2.755691 | 0.388066 | 1.443868 |
| C | 1.772864 | -0.106851 | -0.711397 |
| C | 3.99155 | -0.076607 | 0.998157 |
| H | 2.661721 | 0.772239 | 2.461434 |
| C | 2.996346 | -0.572007 | -1.176589 |
| H | 0.905852 | -0.124161 | -1.374632 |
| C | 4.092326 | -0.551767 | -0.309211 |
| H | 4.866016 | -0.069411 | 1.647439 |
| H | 3.109279 | -0.947432 | -2.192683 |
| O | 6.337157 | -1.018506 | -0.018629 |
| O | 5.461121 | -1.448438 | -1.940563 |
| N | 5.386051 | -1.039523 | -0.789067 |
| C | 1.125541 | 3.809409 | 0.726682 |
| H | 1.751356 | 3.218769 | 1.412309 |
| H | 1.758697 | 4.611047 | 0.31492 |
| H | 0.320768 | 4.279763 | 1.316049 |
| C | -0.145855 | 3.85656 | -1.448692 |
| H | -0.97695 | 4.428953 | -1.00527 |
| H | 0.578285 | 4.582324 | -1.850149 |
| H | -0.543676 | 3.277719 | -2.296472 |
| O | -3.751947 | -1.007923 | -1.083632 |
| C | -4.153873 | -1.858884 | -2.182449 |
| H | -3.338402 | -2.52959 | -2.487236 |
| H | -5.009087 | -2.450095 | -1.829389 |
| H | -4.463363 | -1.238619 | -3.03556 |
| H | -3.735789 | 0.031563 | 2.518466 |
| H | -1.258591 | -2.586508 | -0.952775 |

**10l**

B3LYP-D3BJ/Def2-SVP Electronic Energy: -1039.700474 a.u.

B3LYP-D3BJ/Def2-SVP Gibbs free Energy: -1039.352675 a.u.

B3LYP-D3BJ/Def2-TZVP Electronic Energy: -1040.838943 a.u.

| | | | |
|---|---|---|---|
| O | 2.307182 | 0.652672 | -0.850585 |
| C | 3.000941 | -0.425533 | -0.462698 |
| O | 4.214341 | -0.507383 | -0.978467 |
| C | 2.51908 | -1.385227 | 0.398887 |
| O | 0.594998 | -2.21122 | 1.479132 |
| C | 1.110786 | -1.380156 | 0.725696 |
| C | 0.244489 | -0.324114 | -0.020866 |
| C | 1.1214 | 0.886895 | -0.13569 |
| C | 0.961174 | 2.058978 | 0.503019 |
| C | -0.169971 | 2.18707 | 1.427081 |
| H | -0.218421 | 3.103161 | 2.023721 |
| C | -1.081533 | 1.214096 | 1.614246 |
| C | -1.090199 | -0.039092 | 0.757691 |
| H | -1.203762 | -0.891137 | 1.439351 |
| C | -0.023237 | -0.934363 | -1.422809 |
| H | 0.914077 | -1.218744 | -1.919757 |
| H | -0.548105 | -0.21638 | -2.065788 |
| H | -0.644024 | -1.835726 | -1.329845 |
| C | -2.173164 | 1.313089 | 2.636556 |
| H | -2.136359 | 2.26682 | 3.183523 |
| H | -2.091959 | 0.489096 | 3.367993 |
| H | -3.169017 | 1.216396 | 2.171342 |
| C | 1.929981 | 3.200627 | 0.402572 |
| H | 2.721157 | 3.016532 | -0.335211 |
| H | 2.403484 | 3.388704 | 1.381959 |
| H | 1.400483 | 4.12786 | 0.125545 |
| C | 4.770096 | 0.616344 | -1.681435 |
| H | 5.776114 | 0.301967 | -1.983725 |
| H | 4.833161 | 1.492881 | -1.019981 |
| H | 4.168667 | 0.857321 | -2.569102 |
| C | 3.390056 | -2.507509 | 0.892036 |
| H | 3.273868 | -3.42085 | 0.281091 |
| H | 4.453617 | -2.230402 | 0.878677 |
| H | 3.113726 | -2.774349 | 1.923501 |
| C | -2.326456 | -0.007149 | -0.140067 |
| C | -3.205447 | -1.109806 | -0.218564 |
| C | -2.588388 | 1.135049 | -0.910558 |
| C | -4.313985 | -1.026677 | -1.078293 |
| C | -3.692301 | 1.203245 | -1.76211 |
| H | -1.906635 | 1.986145 | -0.841946 |
| C | -4.562308 | 0.112921 | -1.846132 |
| H | -4.995837 | -1.879434 | -1.142148 |
| H | -3.871825 | 2.103278 | -2.356015 |
| H | -5.433123 | 0.148292 | -2.506185 |
| C | -2.98558 | -2.36048 | 0.594875 |
| H | -1.981371 | -2.784663 | 0.439306 |
| H | -3.075334 | -2.158359 | 1.675868 |
| H | -3.72735 | -3.13021 | 0.336559 |

**TS3**

B3LYP-D3BJ/Def2-SVP Electronic Energy: -1039.660478 a.u.

B3LYP-D3BJ/Def2-SVP Gibbs free Energy: -1039.309938 a.u.

B3LYP-D3BJ/Def2-TZVP Electronic Energy: -1040.798798 a.u.

| | | | |
|---|---|---|---|
| O | -1.879175 | -1.318185 | 0.399236 |
| C | -2.922652 | -0.578821 | -0.025474 |
| O | -4.10154 | -1.155811 | 0.124993 |
| C | -2.796659 | 0.674943 | -0.583858 |
| O | -1.299267 | 2.216085 | -1.576497 |
| C | -1.483663 | 1.137889 | -1.012381 |
| C | -0.337107 | 0.086886 | -0.841699 |
| C | -0.704033 | -0.545454 | 0.467895 |
| C | -0.330762 | -0.02419 | 1.65784 |
| C | 0.418845 | 1.236759 | 1.612151 |
| H | 0.381211 | 1.852476 | 2.517001 |
| C | 0.945244 | 1.729739 | 0.471447 |
| C | 1.066578 | 0.767943 | -0.705129 |
| H | 1.222728 | 1.34938 | -1.627425 |
| C | -0.548541 | -0.83278 | -2.080851 |
| H | -0.337007 | -1.886609 | -1.868429 |
| H | 0.076719 | -0.494043 | -2.917416 |
| H | -1.592127 | -0.790305 | -2.421978 |
| C | 1.30757 | 3.171988 | 0.292179 |
| H | 0.47056 | 3.678843 | -0.21987 |
| H | 2.188699 | 3.315068 | -0.349529 |
| H | 1.475493 | 3.681458 | 1.252991 |
| C | -0.893196 | -0.487088 | 2.969167 |
| H | -1.39622 | -1.459312 | 2.885033 |
| H | -1.624096 | 0.246847 | 3.353166 |
| H | -0.095121 | -0.56799 | 3.725352 |
| C | -4.215812 | -2.347309 | 0.920469 |
| H | -5.282136 | -2.602609 | 0.911463 |
| H | -3.883321 | -2.157479 | 1.951669 |
| H | -3.629331 | -3.166392 | 0.481127 |
| C | -4.006648 | 1.495121 | -0.937251 |
| H | -4.317432 | 1.346075 | -1.98737 |
| H | -4.864783 | 1.245936 | -0.296996 |
| H | -3.784403 | 2.566424 | -0.817347 |
| C | 2.282554 | -0.200966 | -0.536074 |
| C | 3.445581 | 0.075121 | 0.247285 |
| C | 2.279459 | -1.424853 | -1.233498 |
| C | 4.453731 | -0.906054 | 0.33835 |
| C | 3.293843 | -2.377754 | -1.138912 |
| H | 1.461167 | -1.660071 | -1.896893 |
| C | 4.393642 | -2.128357 | -0.324499 |
| H | 5.328503 | -0.681016 | 0.954154 |
| H | 3.212101 | -3.307987 | -1.707246 |
| H | 5.200742 | -2.857804 | -0.218483 |
| C | 3.75983 | 1.36185 | 0.977471 |
| H | 3.703898 | 2.234013 | 0.314255 |
| H | 3.0902 | 1.551628 | 1.82613 |
| H | 4.786994 | 1.319081 | 1.367019 |

**TS3’**

B3LYP-D3BJ/Def2-SVP Electronic Energy: -1039.651946 a.u.
B3LYP-D3BJ/Def2-SVP Gibbs free Energy: -1039.301281 a.u.
B3LYP-D3BJ/Def2-TZVP Electronic Energy: -1040.790553 a.u.

| | | | |
|---|---|---|---|
| O | -1.836385 | -1.299074 | 0.473885 |
| C | -2.887457 | -0.592266 | 0.010095 |
| O | -4.057409 | -1.189384 | 0.156201 |
| C | -2.77481 | 0.647628 | -0.579834 |
| O | -1.297825 | 2.18579 | -1.605892 |
| C | -1.46416 | 1.124689 | -1.007374 |
| C | -0.303245 | 0.086124 | -0.803603 |
| C | -0.680221 | -0.497104 | 0.524375 |
| C | -0.379219 | 0.131488 | 1.686074 |
| C | 0.323871 | 1.413705 | 1.579443 |
| H | 0.248499 | 2.083475 | 2.442133 |
| C | 0.886299 | 1.839008 | 0.42766 |
| C | 1.081135 | 0.787864 | -0.668972 |
| H | 1.263178 | 1.30125 | -1.626301 |
| C | -0.509939 | -0.826165 | -2.044342 |
| H | 0.223863 | -0.580576 | -2.820152 |
| H | -1.503832 | -0.666583 | -2.484903 |
| H | -0.444624 | -1.889544 | -1.805731 |
| C | 1.314214 | 3.250466 | 0.178243 |
| H | 0.624493 | 3.708411 | -0.552291 |
| H | 2.319537 | 3.309821 | -0.270276 |
| H | 1.296392 | 3.855819 | 1.09732 |
| C | -0.989394 | -0.25461 | 3.001321 |
| H | -1.395421 | -1.274605 | 2.986411 |
| H | -1.811517 | 0.437446 | 3.258732 |
| H | -0.245245 | -0.183419 | 3.811053 |
| C | -4.16464 | -2.348062 | 0.999587 |
| H | -5.225222 | -2.625823 | 0.980221 |
| H | -3.857734 | -2.107632 | 2.028219 |
| H | -3.552996 | -3.173016 | 0.608059 |
| C | -3.99278 | 1.435256 | -0.977282 |
| H | -4.269015 | 1.266776 | -2.034165 |
| H | -4.862881 | 1.173596 | -0.358703 |
| H | -3.799219 | 2.513011 | -0.864716 |
| C | 2.39847 | -0.009617 | -0.31924 |
| C | 2.830072 | -1.297475 | -0.750268 |
| C | 3.318882 | 0.707474 | 0.482686 |
| C | 4.097862 | -1.769229 | -0.344793 |
| C | 4.57138 | 0.230496 | 0.861665 |
| H | 3.059084 | 1.696368 | 0.845503 |
| C | 4.972791 | -1.036161 | 0.448193 |
| H | 4.397865 | -2.765484 | -0.679447 |
| H | 5.219969 | 0.854904 | 1.481536 |
| H | 5.944748 | -1.4478 | 0.731969 |
| C | 2.064936 | -2.266491 | -1.612921 |
| H | 2.68833 | -3.146944 | -1.821454 |
| H | 1.155804 | -2.625826 | -1.112889 |
| H | 1.774613 | -1.834394 | -2.57797 |

**atropisomer 10l**
B3LYP-D3BJ/Def2-SVP Electronic Energy: -1039.698643 a.u.
B3LYP-D3BJ/Def2-SVP Gibbs free Energy: -1039.349742 a.u.
B3LYP-D3BJ/Def2-TZVP Electronic Energy: -1040.837025 a.u.

| | | | |
|---|---|---|---|
| O | -2.419677 | -0.524636 | -0.744317 |
| C | -3.016347 | 0.596108 | -0.324709 |
| O | -4.242458 | 0.763692 | -0.78789 |
| C | -2.433772 | 1.514304 | 0.517865 |
| O | -0.43313 | 2.149232 | 1.579276 |
| C | -1.02653 | 1.386023 | 0.811053 |
| C | -0.255133 | 0.285759 | 0.021216 |
| C | -1.209166 | -0.865207 | -0.122827 |
| C | -1.046906 | -2.119108 | 0.334437 |
| C | 0.175729 | -2.429706 | 1.076852 |
| H | 0.263594 | -3.441329 | 1.484078 |
| C | 1.152307 | -1.531527 | 1.300092 |
| C | 1.065641 | -0.102495 | 0.793225 |
| H | 1.036067 | 0.511934 | 1.706268 |
| C | 0.018464 | 0.917976 | -1.37103 |
| H | 0.57435 | 0.224518 | -2.014066 |
| H | 0.60617 | 1.839087 | -1.262184 |
| H | -0.921068 | 1.173072 | -1.879907 |
| C | 2.365581 | -1.864699 | 2.11655 |
| H | 2.488514 | -1.143292 | 2.944046 |
| H | 3.289595 | -1.803196 | 1.517531 |
| H | 2.298344 | -2.876677 | 2.542267 |
| C | -2.072578 | -3.203165 | 0.172113 |
| H | -2.941702 | -2.877022 | -0.412804 |
| H | -2.425153 | -3.547497 | 1.159792 |
| H | -1.624974 | -4.080398 | -0.325226 |
| C | -4.906648 | -0.316931 | -1.463476 |
| H | -5.899931 | 0.068704 | -1.722263 |
| H | -5.003408 | -1.187242 | -0.797893 |
| H | -4.36372 | -0.599445 | -2.376252 |
| C | -3.198814 | 2.692528 | 1.054108 |
| H | -2.987097 | 3.614976 | 0.48416 |
| H | -4.283446 | 2.519577 | 1.021089 |
| H | -2.90996 | 2.888344 | 2.098316 |
| C | 2.350096 | 0.341891 | 0.093239 |
| C | 2.9736 | -0.397462 | -0.939549 |
| C | 2.920881 | 1.558739 | 0.497249 |
| C | 4.148046 | 0.115247 | -1.515911 |
| C | 4.08802 | 2.055574 | -0.085775 |
| H | 2.421186 | 2.133424 | 1.281081 |
| C | 4.708837 | 1.32459 | -1.100484 |
| H | 4.630054 | -0.453391 | -2.316432 |
| H | 4.506495 | 3.007764 | 0.250555 |
| H | 5.62323 | 1.694284 | -1.572075 |
| C | 2.441648 | -1.71707 | -1.441468 |
| H | 2.738045 | -2.547116 | -0.778831 |
| H | 1.34545 | -1.737039 | -1.502464 |
| H | 2.843751 | -1.936551 | -2.441947 |

**11l**

B3LYP-D3BJ/Def2-SVP Electronic Energy: -1039.712361 a.u.

B3LYP-D3BJ/Def2-SVP Gibbs free Energy: -1039.363303 a.u.

B3LYP-D3BJ/Def2-TZVP Electronic Energy: -1040.852715 a.u.

| | | | |
|---|---|---|---|
| O | -0.312799 | -1.105651 | -2.307921 |
| O | -0.837035 | 2.887995 | -0.222907 |
| O | -4.022871 | -0.55028 | -0.071483 |
| C | -0.612248 | -0.572421 | -1.259633 |
| C | 2.226263 | -0.101377 | 0.243805 |
| C | -0.97415 | -1.287046 | 1.071668 |
| H | -1.456395 | -1.981918 | 1.765885 |
| C | -1.639806 | -1.157659 | -0.295341 |
| C | 0.815901 | 0.391896 | 0.549031 |
| H | 0.904076 | 1.34609 | 1.084368 |
| C | 0.005387 | 0.718231 | -0.770833 |
| C | -1.118807 | 1.705366 | -0.384798 |
| C | 0.092525 | -0.576789 | 1.466164 |
| C | 2.415031 | -1.406687 | -0.233536 |
| H | 1.553344 | -2.06722 | -0.347806 |
| C | 0.871841 | 1.368154 | -1.851225 |
| H | 0.250314 | 1.653583 | -2.713473 |
| H | 1.357791 | 2.271511 | -1.463163 |
| H | 1.642594 | 0.672319 | -2.203489 |
| C | -2.751846 | -0.103647 | -0.194974 |
| C | -2.499889 | 1.231537 | -0.234401 |
| C | 3.685387 | -1.885784 | -0.557411 |
| H | 3.806039 | -2.905703 | -0.931336 |
| C | -2.161752 | -2.499604 | -0.808875 |
| H | -1.320156 | -3.193985 | -0.943842 |
| H | -2.864568 | -2.950855 | -0.096551 |
| H | -2.670458 | -2.382754 | -1.776187 |
| C | 3.347169 | 0.74046 | 0.424377 |
| C | 4.618924 | 0.241788 | 0.096442 |
| H | 5.489504 | 0.88913 | 0.233538 |
| C | 0.662619 | -0.697456 | 2.850279 |
| H | 0.10762 | -1.427719 | 3.457044 |
| H | 1.721706 | -1.003405 | 2.81846 |
| H | 0.632342 | 0.278888 | 3.365055 |
| C | -3.560771 | 2.275428 | -0.041856 |
| H | -3.509073 | 3.034326 | -0.838973 |
| H | -4.56277 | 1.828559 | -0.041087 |
| H | -3.413144 | 2.808582 | 0.912334 |
| C | -4.431525 | -1.034099 | 1.215924 |
| H | -5.49648 | -1.285385 | 1.127423 |
| H | -3.869449 | -1.932767 | 1.513869 |
| H | -4.297244 | -0.249772 | 1.978251 |
| C | 4.795137 | -1.053372 | -0.394657 |
| H | 5.797623 | -1.412124 | -0.642359 |
| C | 3.218552 | 2.139606 | 0.973871 |
| H | 2.543857 | 2.765801 | 0.369356 |
| H | 2.811772 | 2.133388 | 1.999223 |
| H | 4.199144 | 2.635765 | 1.007823 |

**TS4**

B3LYP-D3BJ/Def2-SVP Electronic Energy: -1039.677846 a.u.

B3LYP-D3BJ/Def2-SVP Gibbs free Energy: -1039.326559 a.u.

B3LYP-D3BJ/Def2-TZVP Electronic Energy: -1040.818053 a.u.

| | | | |
|---|---|---|---|
| O | -0.052734 | 1.351119 | -2.04211 |
| O | -1.549228 | 1.42733 | 2.337859 |
| O | -3.543992 | -0.578344 | -1.406831 |
| C | -0.363944 | 0.763458 | -1.028019 |
| C | 2.251262 | 0.180509 | 0.281208 |
| C | -0.634666 | -1.468283 | 0.129955 |
| H | -1.194682 | -2.404425 | 0.225941 |
| C | -1.07514 | -0.592429 | -1.035492 |
| C | 0.890533 | 0.275202 | 1.022841 |
| H | 1.083785 | 0.653466 | 2.040271 |
| C | -0.156202 | 1.282797 | 0.384229 |
| C | -1.491863 | 1.112143 | 1.158338 |
| C | 0.145449 | -1.055034 | 1.142245 |
| C | 2.663877 | 1.242919 | -0.544364 |
| H | 2.027346 | 2.107264 | -0.672457 |
| C | 0.166139 | 2.789021 | 0.465807 |
| H | -0.749125 | 3.36732 | 0.66098 |
| H | 0.874489 | 3.001913 | 1.278003 |
| H | 0.573493 | 3.169611 | -0.478233 |
| C | -2.508035 | -0.139602 | -0.656512 |
| C | -2.693544 | 0.675672 | 0.412015 |
| C | 3.876577 | 1.251634 | -1.234439 |
| H | 4.131035 | 2.109594 | -1.861947 |
| C | -0.991301 | -1.264703 | -2.401897 |
| H | 0.065304 | -1.426959 | -2.66163 |
| H | -1.493587 | -2.241566 | -2.393942 |
| H | -1.448632 | -0.642079 | -3.182812 |
| C | 3.152352 | -0.915331 | 0.415702 |
| C | 4.367484 | -0.891456 | -0.293847 |
| H | 5.042577 | -1.744143 | -0.183193 |
| C | 0.076748 | -1.729166 | 2.486528 |
| H | -0.692787 | -1.209176 | 3.085982 |
| H | -0.223739 | -2.784459 | 2.402636 |
| H | 1.00895 | -1.668072 | 3.060228 |
| C | -4.035506 | 1.061123 | 0.957569 |
| H | -4.090151 | 2.147621 | 1.133336 |
| H | -4.83975 | 0.773089 | 0.268085 |
| H | -4.214558 | 0.571218 | 1.929247 |
| C | -4.053464 | -1.886024 | -1.103674 |
| H | -4.879027 | -2.068009 | -1.803973 |
| H | -3.283564 | -2.661393 | -1.23596 |
| H | -4.429985 | -1.919042 | -0.068587 |
| C | 4.741662 | 0.167971 | -1.117162 |
| H | 5.694874 | 0.144292 | -1.651441 |
| C | 2.930831 | -2.119432 | 1.294388 |
| H | 2.816309 | -1.835648 | 2.35026 |

| | | | |
|---|---|---|---|
| H | 2.0384 | -2.692032 | 1.006404 |
| H | 3.795181 | -2.795163 | 1.228941 |

**TS4’**

B3LYP-D3BJ/Def2-SVP Electronic Energy: -1039.668384 a.u.

B3LYP-D3BJ/Def2-SVP Gibbs free Energy: -1039.317137 a.u.

B3LYP-D3BJ/Def2-TZVP Electronic Energy: -1040.807714 a.u.

| | | | |
|---|---|---|---|
| O | -0.242143 | 1.41568 | -1.992605 |
| O | -1.502778 | 1.314689 | 2.413851 |
| O | -3.618174 | -0.567126 | -1.322783 |
| C | -0.434513 | 0.756194 | -0.991157 |
| C | 2.314147 | -0.06092 | 0.398739 |
| C | -0.664391 | -1.531241 | 0.073241 |
| H | -1.211119 | -2.478196 | 0.128424 |
| C | -1.137305 | -0.602186 | -1.033155 |
| C | 0.892292 | 0.169306 | 1.020419 |
| H | 1.063868 | 0.507821 | 2.055028 |
| C | -0.13432 | 1.198965 | 0.426877 |
| C | -1.472848 | 1.026654 | 1.227497 |
| C | 0.140226 | -1.171845 | 1.085815 |
| C | 2.956268 | -1.194961 | 0.953717 |
| H | 2.449971 | -1.761952 | 1.727123 |
| C | 0.218959 | 2.678025 | 0.61202 |
| H | 1.198967 | 2.938172 | 0.214099 |
| H | -0.532432 | 3.314734 | 0.119321 |
| H | 0.214393 | 2.908455 | 1.685849 |
| C | -2.556469 | -0.160449 | -0.592701 |
| C | -2.699515 | 0.62149 | 0.505953 |
| C | 4.216776 | -1.64558 | 0.580408 |
| H | 4.638456 | -2.53249 | 1.060122 |
| C | -1.099494 | -1.212227 | -2.431152 |
| H | -0.052252 | -1.362869 | -2.732734 |
| H | -1.602384 | -2.188566 | -2.450302 |
| H | -1.581552 | -0.555099 | -3.167267 |
| C | 3.054667 | 0.662773 | -0.580066 |
| C | 4.3279 | 0.172237 | -0.9587 |
| H | 4.874631 | 0.730137 | -1.723227 |
| C | 0.121381 | -1.947481 | 2.377171 |
| H | 1.096222 | -2.041386 | 2.874501 |
| H | -0.529746 | -1.405771 | 3.08796 |
| H | -0.301604 | -2.953745 | 2.237604 |
| C | -4.020265 | 0.995491 | 1.108486 |
| H | -4.061212 | 2.075238 | 1.324534 |
| H | -4.847545 | 0.737088 | 0.43468 |
| H | -4.172238 | 0.471872 | 2.06709 |
| C | -4.114576 | -1.889437 | -1.063032 |
| H | -4.952026 | -2.048236 | -1.754707 |
| H | -3.343069 | -2.654088 | -1.23959 |
| H | -4.471362 | -1.964924 | -0.023171 |
| C | 4.917215 | -0.957414 | -0.408763 |
| H | 5.904642 | -1.287041 | -0.741748 |
| C | 2.704927 | 1.970374 | -1.254577 |
| H | 3.319013 | 2.087707 | -2.158959 |
| H | 1.662464 | 2.069292 | -1.556342 |
| H | 2.954314 | 2.821309 | -0.598229 |

**high energy conformer 11l**

B3LYP-D3BJ/Def2-SVP Electronic Energy: -1039.708777 a.u.

B3LYP-D3BJ/Def2-SVP Gibbs free Energy: -1039.358516 a.u.

B3LYP-D3BJ/Def2-TZVP Electronic Energy: -1040.848365 a.u.

| | | | |
|---|---|---|---|
| O | -0.579326 | -0.771691 | -2.521489 |
| O | -0.739544 | 2.99119 | -0.245381 |
| O | -4.000459 | -0.338416 | 0.251928 |
| C | -0.738775 | -0.394851 | -1.378442 |
| C | 2.277309 | 0.198675 | 0.394472 |
| C | -0.973062 | -1.480889 | 0.811321 |
| H | -1.397784 | -2.32144 | 1.367803 |
| C | -1.741465 | -1.047384 | -0.431348 |
| C | 0.762239 | 0.320004 | 0.554984 |
| H | 0.63786 | 1.156268 | 1.261354 |
| C | 0.007876 | 0.772992 | -0.771309 |
| C | -1.056022 | 1.809736 | -0.32645 |
| C | 0.140446 | -0.878717 | 1.252312 |
| C | 3.058152 | 1.213112 | 0.97007 |
| H | 2.552865 | 2.034224 | 1.485647 |
| C | 0.935174 | 1.436024 | -1.787907 |
| H | 0.353019 | 1.812681 | -2.64184 |
| H | 1.463212 | 2.280253 | -1.328735 |
| H | 1.677807 | 0.724292 | -2.167063 |
| C | -2.73633 | 0.04838 | -0.033097 |
| C | -2.412499 | 1.368641 | 0.015814 |
| C | 4.452615 | 1.202214 | 0.902206 |
| H | 5.029722 | 2.009292 | 1.360484 |
| C | -2.434346 | -2.226603 | -1.11632 |
| H | -1.67801 | -2.949995 | -1.45275 |
| H | -3.117266 | -2.742045 | -0.429046 |
| H | -3.006174 | -1.893398 | -1.993989 |
| C | 2.925877 | -0.867179 | -0.274914 |
| C | 4.329647 | -0.868031 | -0.326253 |
| H | 4.832394 | -1.69385 | -0.837289 |
| C | 0.831945 | -1.331118 | 2.507002 |
| H | 0.997645 | -0.480067 | 3.189891 |
| H | 0.244794 | -2.096122 | 3.035386 |
| H | 1.827213 | -1.750586 | 2.285158 |
| C | -3.377571 | 2.433283 | 0.450131 |
| H | -3.465661 | 3.214876 | -0.322006 |
| H | -4.372452 | 2.014829 | 0.644734 |
| H | -3.018721 | 2.933623 | 1.364906 |
| C | -4.227951 | -1.033449 | 1.485802 |
| H | -5.316597 | -1.136429 | 1.583746 |
| H | -3.769226 | -2.033847 | 1.488094 |
| H | -3.833774 | -0.447648 | 2.331668 |
| C | 5.093962 | 0.149243 | 0.249198 |
| H | 6.184831 | 0.115261 | 0.187136 |

| | | | |
|---|---|---|---|
| C | 2.179589 | -1.984498 | -0.956615 |
| H | 1.389809 | -2.409988 | -0.321949 |
| H | 1.69026 | -1.632065 | -1.877438 |
| H | 2.867994 | -2.79429 | -1.238323 |

**11d**

B3LYP-D3BJ/Def2-SVP Electronic Energy: -3573.693355 a.u.

B3LYP-D3BJ/Def2-SVP Gibbs free Energy: -3573.383790 a.u.

B3LYP-D3BJ/Def2-TZVP Electronic Energy: -3575.104181 a.u.

| | | | |
|---|---|---|---|
| O | -0.942722 | -0.646023 | -2.267753 |
| O | -2.393991 | 2.894285 | 0.204749 |
| O | -4.866609 | -1.033438 | -0.480841 |
| C | -1.420473 | -0.301528 | -1.2104 |
| C | 1.133313 | 0.409304 | 0.569637 |
| C | -1.107666 | 1.014545 | -0.530198 |
| C | -2.430136 | 1.725465 | -0.147808 |
| C | 1.583961 | -0.828065 | 0.087002 |
| H | 0.872841 | -1.637282 | -0.088548 |
| C | -0.344805 | 0.67947 | 0.809253 |
| H | -0.40411 | 1.59278 | 1.421707 |
| C | -0.986574 | -0.459654 | 1.579496 |
| C | 3.43792 | 1.208562 | 0.569288 |
| H | 4.159411 | 2.003759 | 0.763823 |
| C | -2.398363 | -1.166561 | -0.411209 |
| C | -3.706953 | 0.998838 | -0.248291 |
| C | -3.702844 | -0.353926 | -0.373953 |
| C | -0.306672 | 1.948646 | -1.437569 |
| H | -0.909073 | 2.237588 | -2.311949 |
| H | -0.028897 | 2.860103 | -0.892579 |
| H | 0.603037 | 1.455684 | -1.80103 |
| C | -1.868966 | -1.296149 | 1.013243 |
| H | -2.29563 | -2.115908 | 1.59969 |
| C | -2.581916 | -2.529917 | -1.078059 |
| H | -1.606978 | -3.030695 | -1.162503 |
| H | -3.246963 | -3.174832 | -0.488927 |
| H | -3.000429 | -2.423771 | -2.088237 |
| C | -4.963481 | 1.807847 | -0.109341 |
| H | -5.852513 | 1.192939 | -0.297287 |
| H | -5.039627 | 2.238483 | 0.902893 |
| H | -4.960357 | 2.656528 | -0.811821 |
| C | -5.328639 | -1.720584 | 0.687457 |
| H | -6.28213 | -2.192552 | 0.416232 |
| H | -4.620977 | -2.497399 | 1.016349 |
| H | -5.491071 | -1.008126 | 1.513023 |
| C | 2.077828 | 1.415764 | 0.811646 |
| H | 1.746354 | 2.383589 | 1.195412 |
| C | 2.938459 | -1.054987 | -0.164676 |
| H | 3.272896 | -2.023309 | -0.540708 |
| C | 3.855699 | -0.02928 | 0.077231 |
| Br | 5.713613 | -0.328799 | -0.261394 |
| C | -0.545781 | -0.606424 | 3.007635 |
| H | 0.547534 | -0.736964 | 3.073726 |
| H | -0.787984 | 0.305394 | 3.581196 |
| H | -1.028662 | -1.464811 | 3.496776 |

**[3]11d**

UB3LYP-D3BJ/Def2-SVP Electronic Energy: -3573.609769 a.u.

UB3LYP-D3BJ/Def2-SVP Gibbs free Energy: -3573.304201 a.u.

UB3LYP-D3BJ/Def2-TZVP Electronic Energy: -3575.020204 a.u.

| | | | |
|---|---|---|---|
| O | -0.986448 | -1.815491 | -1.223546 |
| O | -2.603723 | 2.517857 | -1.043845 |
| O | -4.215466 | -1.751413 | -0.421923 |
| C | -1.39662 | -0.917904 | -0.515498 |
| C | 1.114848 | 0.755845 | 0.21896 |
| C | -1.230306 | 0.555668 | -0.841281 |
| C | -2.575973 | 1.321671 | -0.736461 |
| C | 1.565765 | -0.486993 | 0.687963 |
| H | 0.869508 | -1.173179 | 1.173106 |
| C | -0.345498 | 1.161806 | 0.315663 |
| H | -0.389111 | 2.252147 | 0.166408 |
| C | -0.941668 | 0.846012 | 1.675255 |
| C | 3.386926 | 1.26034 | -0.515059 |
| H | 4.096462 | 1.946504 | -0.980285 |
| C | -2.210306 | -1.154814 | 0.762427 |
| C | -3.729255 | 0.65613 | -0.154577 |
| C | -3.593966 | -0.762562 | 0.269985 |
| C | -0.620809 | 0.772866 | -2.222693 |
| H | -1.301172 | 0.396927 | -3.001763 |
| H | -0.452217 | 1.84425 | -2.396965 |
| H | 0.332742 | 0.238718 | -2.321225 |
| C | -1.782323 | -0.186918 | 1.850779 |
| H | -2.214438 | -0.376358 | 2.838825 |
| C | -2.129449 | -2.604197 | 1.236212 |
| H | -1.100392 | -2.842089 | 1.545346 |
| H | -2.797757 | -2.7599 | 2.096647 |
| H | -2.419438 | -3.2934 | 0.433852 |
| C | -4.959555 | 1.449379 | 0.107453 |
| H | -5.681036 | 1.34282 | -0.72506 |
| H | -5.470095 | 1.096845 | 1.016872 |
| H | -4.719947 | 2.519195 | 0.187828 |
| C | -5.448028 | -1.49693 | -1.087546 |
| H | -5.764915 | -2.453233 | -1.524174 |
| H | -6.216749 | -1.148995 | -0.378844 |
| H | -5.327635 | -0.757364 | -1.895085 |
| C | 2.044541 | 1.620203 | -0.37684 |
| H | 1.713871 | 2.595276 | -0.742443 |
| C | 2.903253 | -0.867046 | 0.558131 |
| H | 3.236786 | -1.837886 | 0.928296 |
| C | 3.803825 | 0.012679 | -0.046793 |
| Br | 5.638034 | -0.495601 | -0.229806 |
| C | -0.529349 | 1.754472 | 2.796071 |
| H | 0.569369 | 1.777349 | 2.895885 |
| H | -0.846195 | 2.791665 | 2.589489 |
| H | -0.96004 | 1.440888 | 3.75809 |

**$^3$TS5**

UB3LYP-D3BJ/Def2-SVP Electronic Energy: -3573.597489 a.u.

UB3LYP-D3BJ/Def2-SVP Gibbs free Energy: -3573.293253 a.u.

UB3LYP-D3BJ/Def2-TZVP Electronic Energy: -3575.006395 a.u.

| | | | |
|---|---|---|---|
| O | -0.847818 | -0.648641 | -2.455235 |
| O | -2.578845 | 2.89588 | -0.232123 |
| O | -4.531018 | -1.50689 | -0.197361 |
| C | -1.306855 | -0.322579 | -1.380212 |
| C | 1.090654 | 0.551793 | 0.466655 |
| C | -1.126129 | 1.056383 | -0.777433 |
| C | -2.503031 | 1.659776 | -0.387821 |
| C | 1.520139 | -0.759222 | 0.210552 |
| H | 0.79521 | -1.572257 | 0.140601 |
| C | -0.38556 | 0.900616 | 0.599342 |
| H | -0.434162 | 1.902736 | 1.054733 |
| C | -1.131818 | -0.067523 | 1.486926 |
| C | 3.418707 | 1.28293 | 0.420832 |
| H | 4.157605 | 2.081168 | 0.508808 |
| C | -2.129477 | -1.276747 | -0.530169 |
| C | -3.628013 | 0.792871 | -0.168466 |
| C | -3.486898 | -0.659285 | -0.235849 |
| C | -0.389178 | 1.988942 | -1.731123 |
| H | -0.972646 | 2.134534 | -2.652327 |
| H | -0.239909 | 2.968646 | -1.25931 |
| H | 0.586479 | 1.571298 | -2.012107 |
| C | -1.856496 | -1.123184 | 0.939156 |
| H | -2.278576 | -1.894261 | 1.588617 |
| C | -2.15235 | -2.695102 | -1.067797 |
| H | -1.127709 | -3.092785 | -1.10726 |
| H | -2.759009 | -3.344988 | -0.423741 |
| H | -2.570416 | -2.717821 | -2.084323 |
| C | -4.952896 | 1.406133 | 0.12674 |
| H | -5.751083 | 0.934665 | -0.468936 |
| H | -5.227766 | 1.268254 | 1.188131 |
| H | -4.920721 | 2.483262 | -0.077961 |
| C | -5.367595 | -1.584826 | 0.963965 |
| H | -5.676915 | -2.635461 | 1.064179 |
| H | -4.814171 | -1.279713 | 1.865327 |
| H | -6.264495 | -0.957994 | 0.850775 |
| C | 2.058416 | 1.560889 | 0.573073 |
| H | 1.743725 | 2.587248 | 0.777106 |
| C | 2.875172 | -1.058098 | 0.052194 |
| H | 3.191078 | -2.083327 | -0.147661 |
| C | 3.814839 | -0.029968 | 0.157675 |
| Br | 5.673141 | -0.427294 | -0.053914 |
| C | -0.962578 | 0.08502 | 2.963711 |
| H | 0.107346 | 0.104866 | 3.238724 |
| H | -1.384678 | 1.047679 | 3.307472 |
| H | -1.451971 | -0.726155 | 3.522207 |

**$^3$Int5**

UB3LYP-D3BJ/Def2-SVP Electronic Energy: -3573.612949 a.u.

UB3LYP-D3BJ/Def2-SVP Gibbs free Energy: -3573.309582 a.u.

UB3LYP-D3BJ/Def2-TZVP Electronic Energy: -3575.019161 a.u.

| | | | |
|---|---|---|---|
| O | -0.774613 | -0.084761 | -2.589498 |
| O | -2.714841 | 2.962345 | -0.027668 |
| O | -4.415394 | -1.486441 | -0.197555 |
| C | -1.281164 | 0.000814 | -1.486604 |
| C | 1.043251 | 0.485709 | 0.555094 |
| C | -1.133601 | 1.23709 | -0.605945 |
| C | -2.537697 | 1.751191 | -0.200772 |
| C | 1.465805 | -0.751926 | 0.045837 |
| H | 0.73488 | -1.524958 | -0.198069 |
| C | -0.436082 | 0.805822 | 0.742649 |
| H | -0.482951 | 1.698 | 1.389656 |
| C | -1.17937 | -0.319076 | 1.407982 |
| C | 3.37828 | 1.180868 | 0.697277 |
| H | 4.122524 | 1.935104 | 0.958296 |
| C | -2.07675 | -1.115513 | -0.898825 |
| C | -3.599353 | 0.791446 | -0.020083 |
| C | -3.319237 | -0.652047 | -0.094464 |
| C | -0.359885 | 2.336356 | -1.322625 |
| H | -0.913188 | 2.680546 | -2.208242 |
| H | -0.212341 | 3.195282 | -0.655351 |
| H | 0.618358 | 1.969071 | -1.657097 |
| C | -2.053028 | -1.194442 | 0.618023 |
| H | -2.211335 | -2.199362 | 1.0208 |
| C | -2.172949 | -2.374604 | -1.72059 |
| H | -1.168188 | -2.796734 | -1.876784 |
| H | -2.795878 | -3.125285 | -1.218551 |
| H | -2.607869 | -2.163212 | -2.709261 |
| C | -4.974139 | 1.268347 | 0.28575 |
| H | -5.727184 | 0.668101 | -0.246994 |
| H | -5.194715 | 1.167419 | 1.36567 |
| H | -5.078161 | 2.329729 | 0.026095 |
| C | -4.962391 | -1.9671 | 1.023931 |
| H | -5.903576 | -2.47495 | 0.770334 |
| H | -4.289476 | -2.691821 | 1.513106 |
| H | -5.176396 | -1.15049 | 1.732461 |
| C | 2.017443 | 1.439387 | 0.880786 |
| H | 1.708675 | 2.406609 | 1.284865 |
| C | 2.820321 | -1.029081 | -0.148721 |
| H | 3.131309 | -1.996061 | -0.547413 |
| C | 3.767307 | -0.055315 | 0.178252 |
| Br | 5.625145 | -0.42621 | -0.081068 |
| C | -0.978483 | -0.556811 | 2.865476 |
| H | 0.092309 | -0.524443 | 3.137712 |
| H | -1.471644 | 0.227176 | 3.475108 |
| H | -1.386127 | -1.528206 | 3.18423 |

**$^3$TS6**

UB3LYP-D3BJ/Def2-SVP Electronic Energy: -3573.603388 a.u.

UB3LYP-D3BJ/Def2-SVP Gibbs free Energy: -3573.300701 a.u.

UB3LYP-D3BJ/Def2-TZVP Electronic Energy: -3575.011073 a.u.

| | | | |
|---|---|---|---|
| O | -0.95802 | 0.188749 | -2.665814 |
| O | -2.668043 | 3.003446 | 0.115496 |
| O | -4.519731 | -1.30633 | 0.004482 |
| C | -1.318523 | 0.106372 | -1.496414 |
| C | 1.062599 | 0.438773 | 0.576314 |
| C | -1.124762 | 1.266112 | -0.511358 |
| C | -2.516028 | 1.794053 | -0.063889 |
| C | 1.480654 | -0.724648 | -0.088382 |
| H | 0.74522 | -1.448684 | -0.444102 |
| C | -0.415913 | 0.743552 | 0.79335 |
| H | -0.45655 | 1.582863 | 1.508364 |
| C | -1.15226 | -0.422954 | 1.379919 |
| C | 3.402021 | 1.084307 | 0.834941 |
| H | 4.1493 | 1.789788 | 1.202137 |
| C | -1.975793 | -1.081701 | -0.959198 |
| C | -3.612151 | 0.855947 | 0.106323 |
| C | -3.407993 | -0.553894 | 0.144296 |
| C | -0.336037 | 2.398233 | -1.159944 |
| H | -0.892671 | 2.819434 | -2.008257 |
| H | -0.154791 | 3.201893 | -0.434456 |
| H | 0.627477 | 2.03174 | -1.536358 |
| C | -2.092603 | -1.193186 | 0.528461 |
| H | -2.227652 | -2.224576 | 0.870828 |
| C | -2.299102 | -2.234504 | -1.851794 |
| H | -1.705629 | -3.125223 | -1.582498 |
| H | -3.362972 | -2.514366 | -1.776712 |
| H | -2.084221 | -1.966038 | -2.894902 |
| C | -5.008374 | 1.385197 | 0.20133 |
| H | -5.585545 | 1.15032 | -0.71108 |
| H | -5.557581 | 0.931719 | 1.042292 |
| H | -4.98352 | 2.474983 | 0.326249 |
| C | -4.689538 | -2.484176 | 0.795997 |
| H | -5.739517 | -2.780109 | 0.671829 |
| H | -4.044751 | -3.307502 | 0.451954 |
| H | -4.492333 | -2.277693 | 1.859625 |
| C | 2.040988 | 1.330651 | 1.035869 |
| H | 1.736105 | 2.239318 | 1.560906 |
| C | 2.834631 | -0.98676 | -0.304778 |
| H | 3.141876 | -1.894302 | -0.827112 |
| C | 3.786515 | -0.075459 | 0.160288 |
| Br | 5.644245 | -0.428775 | -0.124812 |
| C | -1.055933 | -0.714551 | 2.836395 |
| H | -0.060124 | -0.457127 | 3.233939 |
| H | -1.79101 | -0.119253 | 3.418101 |
| H | -1.257737 | -1.775179 | 3.057979 |

**$^3$Int6**

UB3LYP-D3BJ/Def2-SVP Electronic Energy: -3573.619631 a.u.

UB3LYP-D3BJ/Def2-SVP Gibbs free Energy: -3573.316155 a.u.

UB3LYP-D3BJ/Def2-TZVP Electronic Energy: -3575.028925 a.u.

| | | | |
|---|---|---|---|
| O | 0.958199 | 0.946378 | 2.755725 |
| O | 2.672585 | 2.991063 | -0.303585 |
| O | 4.592556 | -1.213201 | -0.688304 |
| C | 1.297502 | 0.492427 | 1.65702 |
| C | -1.02342 | 0.208937 | -0.579732 |
| C | 1.110004 | 1.304263 | 0.360926 |
| C | 2.508101 | 1.785933 | -0.139855 |
| C | -1.526102 | -0.656344 | 0.404706 |
| H | -0.839647 | -1.220018 | 1.039568 |
| C | 0.476226 | 0.404577 | -0.767236 |
| H | 0.599009 | 0.968667 | -1.709223 |
| C | 1.174946 | -0.914178 | -0.901269 |
| C | -3.315432 | 0.746834 | -1.220982 |
| H | -4.011105 | 1.291928 | -1.861203 |
| C | 1.897851 | -0.804645 | 1.5028 |
| C | 3.607178 | 0.850711 | -0.426814 |
| C | 3.510263 | -0.51507 | -0.324892 |
| C | 0.240589 | 2.526467 | 0.639669 |
| H | 0.741035 | 3.201989 | 1.343695 |
| H | 0.045671 | 3.081792 | -0.287891 |
| H | -0.717922 | 2.222815 | 1.079373 |
| C | 2.238682 | -1.244853 | 0.109705 |
| H | 2.426682 | -2.323332 | 0.105767 |
| C | 2.218411 | -1.669318 | 2.662269 |
| H | 1.689724 | -2.637279 | 2.583709 |
| H | 3.297234 | -1.910769 | 2.680957 |
| H | 1.942285 | -1.181942 | 3.606524 |
| C | 4.906203 | 1.4481 | -0.906403 |
| H | 5.765576 | 1.027544 | -0.361155 |
| H | 5.077873 | 1.237057 | -1.976668 |
| H | 4.889431 | 2.536298 | -0.772595 |
| C | 4.675658 | -2.632103 | -0.558576 |
| H | 5.692777 | -2.897228 | -0.874643 |
| H | 4.527668 | -2.951985 | 0.484164 |
| H | 3.951986 | -3.139797 | -1.214348 |
| C | -1.935922 | 0.89868 | -1.38845 |
| H | -1.564353 | 1.572165 | -2.164715 |
| C | -2.899671 | -0.817955 | 0.592647 |
| H | -3.273194 | -1.491666 | 1.36562 |
| C | -3.785284 | -0.110602 | -0.22522 |
| Br | -5.669516 | -0.328945 | 0.019053 |
| C | 1.129971 | -1.655253 | -2.190834 |
| H | 0.159891 | -1.518903 | -2.695536 |
| H | 1.908593 | -1.292799 | -2.895325 |
| H | 1.3026 | -2.735011 | -2.05049 |

**$^3$TS7**

UB3LYP-D3BJ/Def2-SVP Electronic Energy: -3573.610718 a.u.

UB3LYP-D3BJ/Def2-SVP Gibbs free Energy: -3573.306337 a.u.

UB3LYP-D3BJ/Def2-TZVP Electronic Energy: -3575.019317 a.u.

| | | | |
|---|---|---|---|
| O | 1.017641 | 1.801571 | 2.450213 |
| O | 2.981265 | 2.865978 | -0.690115 |
| O | 4.017346 | -1.700365 | -0.837526 |

| | | | |
|---|---|---|---|
| C | 1.307538 | 1.022812 | 1.534728 |
| C | -0.92289 | 0.11944 | -0.596461 |
| C | 1.186278 | 1.440804 | 0.059882 |
| C | 2.636296 | 1.705878 | -0.442034 |
| C | -1.439161 | -0.46531 | 0.571302 |
| H | -0.767645 | -0.831886 | 1.349721 |
| C | 0.575294 | 0.290412 | -0.81328 |
| H | 0.705951 | 0.597221 | -1.864659 |
| C | 1.281572 | -1.016682 | -0.611257 |
| C | -3.206137 | 0.430338 | -1.401349 |
| H | -3.892449 | 0.777768 | -2.175416 |
| C | 1.812674 | -0.304645 | 1.782029 |
| C | 3.530597 | 0.583539 | -0.654805 |
| C | 3.218109 | -0.743968 | -0.311369 |
| C | 0.364809 | 2.719077 | -0.057429 |
| H | 0.864868 | 3.541332 | 0.466313 |
| H | 0.247417 | 3.004861 | -1.112852 |
| H | -0.631507 | 2.582048 | 0.383498 |
| C | 2.098142 | -1.184656 | 0.62305 |
| H | 2.225361 | -2.228258 | 0.926715 |
| C | 2.047795 | -0.801719 | 3.158823 |
| H | 1.414916 | -1.684254 | 3.368086 |
| H | 3.093114 | -1.141406 | 3.276014 |
| H | 1.832858 | -0.022155 | 3.900972 |
| C | 4.817752 | 0.84818 | -1.385508 |
| H | 5.654688 | 0.278172 | -0.954547 |
| H | 4.749422 | 0.556074 | -2.449489 |
| H | 5.04495 | 1.921333 | -1.347329 |
| C | 4.432884 | -2.802117 | -0.029262 |
| H | 5.276115 | -3.265502 | -0.558801 |
| H | 4.770041 | -2.46351 | 0.963241 |
| H | 3.634092 | -3.551602 | 0.086803 |
| C | -1.825516 | 0.554582 | -1.576593 |
| H | -1.444104 | 1.005044 | -2.496146 |
| C | -2.815192 | -0.596668 | 0.766454 |
| H | -3.198398 | -1.051506 | 1.681365 |
| C | -3.689744 | -0.14348 | -0.224529 |
| Br | -5.575948 | -0.320061 | 0.032804 |
| C | 1.021807 | -2.172957 | -1.513162 |
| H | 0.635019 | -1.83699 | -2.487248 |
| H | 1.939285 | -2.757974 | -1.688716 |
| H | 0.276727 | -2.861285 | -1.071614 |

**$^{3}$TS7'**

UB3LYP-D3BJ/Def2-SVP Electronic Energy: -3573.558939 a.u.

UB3LYP-D3BJ/Def2-SVP Gibbs free Energy: -3573.257515 a.u.

UB3LYP-D3BJ/Def2-TZVP Electronic Energy: -3574.968818 a.u.

| | | | |
|---|---|---|---|
| O | -0.89673 | -2.069731 | 1.857733 |
| O | -2.72054 | -2.006963 | -1.991061 |
| O | -4.722358 | 1.435349 | -0.272373 |
| C | -1.322868 | -1.225229 | 1.074539 |
| C | 1.10518 | 0.169273 | -0.583364 |
| C | -1.031055 | -1.232403 | -0.430857 |
| C | -2.456544 | -1.349733 | -1.009084 |
| C | 1.590244 | 0.318755 | 0.724954 |
| H | 0.889683 | 0.439609 | 1.553878 |
| C | -0.391497 | 0.143447 | -0.85948 |
| H | -0.509677 | 0.204722 | -1.958415 |
| C | -1.078444 | 1.324267 | -0.22628 |
| C | 3.405696 | 0.038171 | -1.382652 |
| H | 4.111773 | -0.065718 | -2.208311 |
| C | -2.34823 | -0.234187 | 1.369864 |
| C | -3.578856 | -0.686109 | -0.205839 |
| C | -3.57144 | 0.74294 | -0.233766 |
| C | -0.190527 | -2.422505 | -0.872378 |
| H | -0.710204 | -3.36483 | -0.649072 |
| H | 0.001862 | -2.383007 | -1.954015 |
| H | 0.770924 | -2.433581 | -0.343573 |
| C | -2.317905 | 1.099095 | 0.571941 |
| H | -2.503605 | 1.961712 | 1.231727 |
| C | -3.008078 | -0.203664 | 2.708937 |
| H | -2.400095 | 0.377092 | 3.427561 |
| H | -3.993839 | 0.286112 | 2.650697 |
| H | -3.13385 | -1.218097 | 3.114115 |
| C | -4.868037 | -1.453061 | -0.150393 |
| H | -5.543606 | -1.011795 | 0.596565 |
| H | -5.379434 | -1.421548 | -1.12693 |
| H | -4.687079 | -2.509166 | 0.098065 |
| C | -4.640545 | 2.856655 | -0.210117 |
| H | -5.652278 | 3.237697 | -0.401602 |
| H | -4.310435 | 3.197491 | 0.784647 |
| H | -3.949292 | 3.246317 | -0.975273 |
| C | 2.029 | 0.033817 | -1.626785 |
| H | 1.669716 | -0.078705 | -2.652733 |
| C | 2.960139 | 0.319979 | 0.991614 |
| H | 3.321748 | 0.433941 | 2.014993 |
| C | 3.858535 | 0.178165 | -0.070034 |
| Br | 5.738443 | 0.184515 | 0.282806 |
| C | -0.798376 | 2.684775 | -0.762765 |
| H | 0.25421 | 2.797992 | -1.068061 |
| H | -1.41498 | 2.88938 | -1.665006 |
| H | -1.038364 | 3.472427 | -0.030198 |

**$^{3}$Int7**

UB3LYP-D3BJ/Def2-SVP Electronic Energy: -3573.629908 a.u.

UB3LYP-D3BJ/Def2-SVP Gibbs free Energy: -3573.323929 a.u.

UB3LYP-D3BJ/Def2-TZVP Electronic Energy: -3575.036818 a.u.

| | | | |
|---|---|---|---|
| O | 1.015556 | 2.228751 | 2.064728 |
| O | 3.150594 | 2.675569 | -0.951413 |
| O | 3.649502 | -2.053461 | -0.704602 |
| C | 1.299764 | 1.282692 | 1.320033 |
| C | -0.879474 | 0.009837 | -0.620883 |
| C | 1.210411 | 1.426376 | -0.21382 |
| C | 2.678757 | 1.567556 | -0.670418 |

| | | | |
|---|---|---|---|
| C | -1.398322 | -0.358782 | 0.630978 |
| H | -0.730531 | -0.59388 | 1.460196 |
| C | 0.610328 | 0.143429 | -0.875461 |
| H | 0.738482 | 0.269785 | -1.961999 |
| C | 1.385319 | -1.110003 | -0.477417 |
| C | -3.165779 | 0.201243 | -1.460785 |
| H | -3.851081 | 0.418723 | -2.281657 |
| C | 1.765154 | 0.012489 | 1.823931 |
| C | 3.474219 | 0.362847 | -0.745596 |
| C | 2.896487 | -0.939274 | -0.38289 |
| C | 0.426468 | 2.677806 | -0.588233 |
| H | 0.933486 | 3.570449 | -0.205098 |
| H | 0.347663 | 2.767039 | -1.682265 |
| H | -0.586137 | 2.645553 | -0.164958 |
| C | 1.995516 | -1.095986 | 0.907075 |
| H | 2.144295 | -2.072358 | 1.374053 |
| C | 1.923241 | -0.207207 | 3.286443 |
| H | 1.19075 | -0.951751 | 3.650945 |
| H | 2.92117 | -0.621846 | 3.512899 |
| H | 1.778305 | 0.728094 | 3.842016 |
| C | 4.870501 | 0.45239 | -1.253536 |
| H | 5.593703 | 0.478347 | -0.416215 |
| H | 5.127626 | -0.421406 | -1.86994 |
| H | 5.006715 | 1.37802 | -1.827453 |
| C | 4.641542 | -2.440033 | 0.23721 |
| H | 5.236052 | -3.233736 | -0.236496 |
| H | 5.308884 | -1.606172 | 0.507773 |
| H | 4.186802 | -2.838778 | 1.160366 |
| C | -1.784659 | 0.281317 | -1.65624 |
| H | -1.404214 | 0.565332 | -2.640464 |
| C | -2.775097 | -0.445171 | 0.847909 |
| H | -3.15782 | -0.73421 | 1.82807 |
| C | -3.650061 | -0.161315 | -0.203208 |
| Br | -5.536908 | -0.27951 | 0.084028 |
| C | 0.861117 | -2.415597 | -1.027854 |
| H | 0.756848 | -2.351274 | -2.123306 |
| H | 1.541412 | -3.244163 | -0.795188 |
| H | -0.128426 | -2.643466 | -0.605389 |

**15**

B3LYP-D3BJ/Def2-SVP Electronic Energy: -3573.695167 a.u.

B3LYP-D3BJ/Def2-SVP Gibbs free Energy: -3573.384701 a.u.

B3LYP-D3BJ/Def2-TZVP Electronic Energy: -3575.105840 a.u.

| | | | |
|---|---|---|---|
| O | 1.592317 | 2.374877 | 2.196158 |
| O | 3.377762 | 2.827602 | -0.71669 |
| O | 3.877001 | -1.813026 | -1.155165 |
| C | 1.617805 | 1.389624 | 1.477916 |
| C | -0.631582 | 0.060993 | -0.477588 |
| C | 1.456951 | 1.531059 | -0.051793 |
| C | 2.893364 | 1.711828 | -0.615149 |
| C | -1.271647 | 0.142011 | 0.770678 |
| H | -0.712894 | 0.380449 | 1.673749 |
| C | 0.859524 | 0.254549 | -0.688465 |
| H | 0.992404 | 0.357857 | -1.77482 |
| C | 1.728665 | -0.970542 | -0.269078 |
| C | -2.80087 | -0.450977 | -1.492178 |
| H | -3.391871 | -0.67782 | -2.381019 |
| C | 1.81136 | 0.032429 | 2.052562 |
| C | 3.665922 | 0.515458 | -0.99479 |
| C | 3.143585 | -0.725353 | -0.814798 |
| C | 0.642977 | 2.784493 | -0.373628 |
| H | 1.166447 | 3.675341 | -0.009528 |
| H | 0.506781 | 2.876016 | -1.462049 |
| H | -0.347587 | 2.741002 | 0.098496 |
| C | 1.830898 | -1.044053 | 1.242788 |
| H | 1.959595 | -2.039202 | 1.680449 |
| C | 1.938682 | -0.050572 | 3.545052 |
| H | 1.041578 | 0.35716 | 4.039761 |
| H | 2.079387 | -1.09031 | 3.872876 |
| H | 2.791122 | 0.55153 | 3.899854 |
| C | 5.054539 | 0.751765 | -1.516373 |
| H | 5.685766 | 1.228106 | -0.7478 |
| H | 5.526742 | -0.187957 | -1.827782 |
| H | 5.033494 | 1.442641 | -2.374342 |
| C | 4.472644 | -2.564216 | -0.094682 |
| H | 5.115932 | -3.318356 | -0.56748 |
| H | 5.084745 | -1.909927 | 0.547932 |
| H | 3.718186 | -3.074873 | 0.525274 |
| C | -1.425676 | -0.231615 | -1.597807 |
| H | -0.957435 | -0.292014 | -2.583202 |
| C | -2.645937 | -0.071853 | 0.899685 |
| H | -3.118545 | -0.000309 | 1.880603 |
| C | -3.401552 | -0.371496 | -0.235405 |
| Br | -5.28322 | -0.663738 | -0.066862 |
| C | 1.156027 | -2.276199 | -0.834786 |
| H | 1.067528 | -2.222082 | -1.9301 |
| H | 1.79298 | -3.134006 | -0.582825 |
| H | 0.157547 | -2.46242 | -0.416109 |

**[1]TS8**

UB3LYP-D3BJ/Def2-SVP Electronic Energy: -3573.586436 a.u.

UB3LYP-D3BJ/Def2-SVP Gibbs free Energy: -3573.278919 a.u.

UB3LYP-D3BJ/Def2-TZVP Electronic Energy: -3574.997215 a.u.

| | | | |
|---|---|---|---|
| O | -1.384536 | -2.539658 | 1.502357 |
| O | -3.170899 | -2.440705 | -1.545082 |
| O | -3.752587 | 1.956037 | -0.299323 |
| C | -1.489757 | -1.491316 | 0.886359 |
| C | 0.906152 | 0.065473 | -0.62099 |
| C | -1.201271 | -1.350582 | -0.603012 |
| C | -2.69847 | -1.410377 | -1.03255 |
| C | 1.385654 | -0.017728 | 0.696685 |
| H | 0.696016 | -0.101079 | 1.536932 |
| C | -0.574016 | 0.023615 | -0.943397 |
| H | -0.673015 | 0.15143 | -2.032273 |

| | | | |
|---|---|---|---|
| C | -1.333571 | 1.201341 | -0.301 |
| C | 3.216043 | 0.204087 | -1.40424 |
| H | 3.926155 | 0.290693 | -2.228375 |
| C | -2.071282 | -0.281966 | 1.582724 |
| C | -3.443804 | -0.343549 | -0.473363 |
| C | -2.80357 | 1.016488 | -0.214 |
| C | -0.403503 | -2.51909 | -1.154941 |
| H | -0.966554 | -3.44908 | -1.015646 |
| H | -0.229812 | -2.382447 | -2.233367 |
| H | 0.57109 | -2.613872 | -0.656157 |
| C | -1.761693 | 1.025198 | 1.15191 |
| H | -1.867177 | 1.879664 | 1.820152 |
| C | -2.744977 | -0.513125 | 2.881064 |
| H | -1.968131 | -0.616088 | 3.662306 |
| H | -3.385388 | 0.336324 | 3.154975 |
| H | -3.316814 | -1.451156 | 2.874143 |
| C | -4.932558 | -0.445239 | -0.356524 |
| H | -5.2185 | -1.506684 | -0.394701 |
| H | -5.298018 | -0.009764 | 0.589008 |
| H | -5.476628 | 0.072363 | -1.169012 |
| C | -3.610405 | 3.348527 | -0.023475 |
| H | -3.095958 | 3.870568 | -0.841233 |
| H | -4.635275 | 3.732681 | 0.062267 |
| H | -3.08403 | 3.517468 | 0.92713 |
| C | 1.842269 | 0.177345 | -1.657995 |
| H | 1.491731 | 0.246178 | -2.690777 |
| C | 2.754255 | 0.009398 | 0.97275 |
| H | 3.105876 | -0.056747 | 2.003713 |
| C | 3.660977 | 0.119641 | -0.084116 |
| Br | 5.537445 | 0.155275 | 0.283931 |
| C | -0.735686 | 2.547601 | -0.67573 |
| H | -0.941449 | 2.781528 | -1.731132 |
| H | -1.099268 | 3.368948 | -0.051241 |
| H | 0.354025 | 2.504605 | -0.544879 |

**12**

B3LYP-D3BJ/Def2-SVP Electronic Energy: -3573.662017 a.u.

B3LYP-D3BJ/Def2-SVP Gibbs free Energy: -3573.350747 a.u.

B3LYP-D3BJ/Def2-TZVP Electronic Energy: -3575.066500 a.u.

| | | | |
|---|---|---|---|
| O | 0.50307 | 1.728863 | 2.030643 |
| O | 3.063235 | 2.200854 | -1.530779 |
| O | 3.667832 | -1.855085 | -1.029283 |
| C | 1.17122 | 1.197959 | 1.172407 |
| C | -0.981889 | -0.024458 | -0.705993 |
| C | 1.070093 | 1.454722 | -0.330572 |
| C | 2.550414 | 1.467803 | -0.71578 |
| C | -1.465516 | -0.48342 | 0.529783 |
| H | -0.772665 | -0.778899 | 1.318068 |
| C | 0.500731 | 0.123864 | -0.980487 |
| H | 0.622827 | 0.250045 | -2.068388 |
| C | 1.323503 | -1.097529 | -0.556201 |
| C | -3.287721 | 0.228318 | -1.468342 |
| H | -3.995371 | 0.504173 | -2.25181 |
| C | 2.296974 | 0.198245 | 1.380964 |
| C | 3.241222 | 0.410794 | 0.123051 |
| C | 2.818553 | -1.010437 | -0.363623 |
| C | 0.302195 | 2.699156 | -0.723891 |
| H | 0.804768 | 3.596507 | -0.333001 |
| H | 0.242367 | 2.789918 | -1.818324 |
| H | -0.717675 | 2.675592 | -0.317099 |
| C | 1.912649 | -1.206037 | 0.840348 |
| H | 1.742038 | -2.074817 | 1.47948 |
| C | 2.908048 | 0.207115 | 2.763139 |
| H | 2.134074 | -0.02161 | 3.512255 |
| H | 3.700513 | -0.550732 | 2.849423 |
| H | 3.336595 | 1.191469 | 3.005681 |
| C | 4.720866 | 0.644218 | 0.329214 |
| H | 4.903401 | 1.654652 | 0.725039 |
| H | 5.155488 | -0.082851 | 1.027801 |
| H | 5.249793 | 0.555162 | -0.631457 |
| C | 4.448271 | -2.725894 | -0.221781 |
| H | 3.809407 | -3.410715 | 0.362333 |
| H | 5.074414 | -3.318922 | -0.903781 |
| H | 5.102317 | -2.175655 | 0.47402 |
| C | -1.912379 | 0.323261 | -1.694979 |
| H | -1.55692 | 0.677322 | -2.665666 |
| C | -2.835725 | -0.587449 | 0.776116 |
| H | -3.193529 | -0.949226 | 1.741388 |
| C | -3.737561 | -0.226206 | -0.227801 |
| Br | -5.616114 | -0.363485 | 0.101321 |
| C | 0.857738 | -2.403754 | -1.167102 |
| H | 0.883072 | -2.342945 | -2.267236 |
| H | 1.499035 | -3.242182 | -0.862549 |
| H | -0.173499 | -2.635445 | -0.862676 |

**14**

B3LYP-D3BJ/Def2-SVP Electronic Energy: -3573.688089 a.u.

B3LYP-D3BJ/Def2-SVP Gibbs free Energy: -3573.376458 a.u.

B3LYP-D3BJ/Def2-TZVP Electronic Energy: -3575.095542 a.u.

| | | | |
|---|---|---|---|
| Br | -5.710811 | -0.039354 | -0.231391 |
| O | 4.747487 | -0.442514 | -1.259113 |
| O | 1.893412 | 0.17402 | 2.893653 |
| O | 2.232896 | 3.107485 | 0.296783 |
| C | -0.997742 | 0.102108 | -0.373183 |
| C | 3.598456 | -0.101498 | -0.640181 |
| C | 3.443874 | 1.254153 | -0.470465 |
| C | 1.866384 | -1.330875 | 1.022753 |
| C | 0.513782 | 0.177454 | -0.428769 |
| H | 0.778302 | 0.632556 | -1.394846 |
| C | 1.135318 | 1.029204 | 0.740358 |
| C | 1.689969 | -0.023168 | 1.715147 |
| C | 1.174757 | -1.205779 | -0.325398 |
| C | 2.686778 | -1.206187 | -0.267852 |
| H | 3.086051 | -2.181272 | -0.538528 |

| | | | |
|---|---|---|---|
| C | -1.765906 | 0.764805 | -1.337809 |
| H | -1.265365 | 1.321836 | -2.133794 |
| C | 2.300693 | 1.88773 | 0.181984 |
| C | -1.668711 | -0.60759 | 0.636848 |
| H | -1.099397 | -1.133249 | 1.406904 |
| C | 0.163009 | 1.949568 | 1.47534 |
| H | -0.292818 | 2.667084 | 0.780702 |
| H | 0.704115 | 2.511868 | 2.246571 |
| H | -0.635237 | 1.376407 | 1.961995 |
| C | -3.061681 | -0.655476 | 0.686268 |
| H | -3.564721 | -1.211898 | 1.47867 |
| C | -3.800247 | 0.019082 | -0.290803 |
| C | 1.961047 | -2.572825 | 1.865662 |
| H | 2.819222 | -2.507515 | 2.552484 |
| H | 2.08508 | -3.470045 | 1.243226 |
| H | 1.050964 | -2.696992 | 2.474256 |
| C | 4.533051 | 2.167369 | -0.978333 |
| H | 5.49952 | 1.954099 | -0.491882 |
| H | 4.262157 | 3.211423 | -0.781184 |
| H | 4.694735 | 2.040074 | -2.06153 |
| C | -3.16401 | 0.731528 | -1.306903 |
| H | -3.743617 | 1.255255 | -2.068835 |
| C | 0.516772 | -2.374246 | -1.019514 |
| H | 1.14248 | -3.275868 | -0.956572 |
| H | 0.364226 | -2.141131 | -2.085994 |
| H | -0.46534 | -2.607525 | -0.584823 |
| C | 5.146885 | -1.787844 | -1.498278 |
| H | 5.237389 | -2.36144 | -0.562342 |
| H | 6.135831 | -1.719544 | -1.970331 |
| H | 4.459578 | -2.303743 | -2.18726 |

**$^{3}$14**

UB3LYP-D3BJ/Def2-SVP Electronic Energy: -3573.597645 a.u.

UB3LYP-D3BJ/Def2-SVP Gibbs free Energy: -3573.292014 a.u.

UB3LYP-D3BJ/Def2-TZVP Electronic Energy: -3575.005409 a.u.

| | | | |
|---|---|---|---|
| Br | -5.712676 | -0.053089 | -0.038484 |
| O | 4.936335 | -0.25248 | -0.453355 |
| O | 2.061012 | -0.500621 | 2.621183 |
| O | 2.137821 | 3.071206 | 0.672517 |
| C | -1.016108 | 0.210217 | -0.403538 |
| C | 3.6349 | 0.018577 | -0.790667 |
| C | 3.345686 | 1.439794 | -0.483632 |
| C | 1.826735 | -1.519625 | 0.459812 |
| C | 0.489135 | 0.330302 | -0.522733 |
| H | 0.688529 | 1.026347 | -1.350636 |
| C | 1.174525 | 0.863817 | 0.794375 |
| C | 1.759004 | -0.414572 | 1.447736 |
| C | 1.166907 | -1.022499 | -0.817976 |
| C | 2.665025 | -1.070886 | -0.809678 |
| H | 3.041412 | -1.972868 | -1.299477 |
| C | -1.84342 | 1.087969 | -1.114374 |
| H | -1.39318 | 1.843194 | -1.76357 |
| C | 2.26468 | 1.87508 | 0.371288 |
| C | -1.623782 | -0.75046 | 0.422821 |
| H | -1.009536 | -1.450926 | 0.992633 |
| C | 0.240899 | 1.550244 | 1.790895 |
| H | -0.263028 | 2.406959 | 1.325176 |
| H | 0.826539 | 1.915752 | 2.644966 |
| H | -0.520562 | 0.857024 | 2.168104 |
| C | -3.011264 | -0.835011 | 0.537094 |
| H | -3.464448 | -1.588507 | 1.183291 |
| C | -3.809193 | 0.057061 | -0.185857 |
| C | 1.880714 | -2.936808 | 0.95127 |
| H | 2.696766 | -3.060652 | 1.680302 |
| H | 2.045246 | -3.642641 | 0.124815 |
| H | 0.93928 | -3.213325 | 1.455427 |
| C | 4.254648 | 2.452617 | -1.081247 |
| H | 5.303566 | 2.212009 | -0.835944 |
| H | 4.009848 | 3.464774 | -0.737988 |
| H | 4.187782 | 2.407848 | -2.184892 |
| C | -3.237033 | 1.022338 | -1.013819 |
| H | -3.862746 | 1.716403 | -1.577366 |
| C | 0.46317 | -1.963365 | -1.771376 |
| H | 1.071769 | -2.857881 | -1.966096 |
| H | 0.288921 | -1.458168 | -2.735592 |
| H | -0.511512 | -2.290094 | -1.384076 |
| C | 5.401096 | -1.592684 | -0.425651 |
| H | 4.854011 | -2.198595 | 0.315036 |
| H | 6.461016 | -1.546636 | -0.141418 |
| H | 5.314718 | -2.069394 | -1.41696 |

**$^{3}$TS9**

UB3LYP-D3BJ/Def2-SVP Electronic Energy: -3573.596716 a.u.

UB3LYP-D3BJ/Def2-SVP Gibbs free Energy: -3573.292281 a.u.

UB3LYP-D3BJ/Def2-TZVP Electronic Energy: -3575.005402 a.u.

| | | | |
|---|---|---|---|
| Br | -5.722583 | -0.04105 | -0.140297 |
| O | 4.938228 | -0.290603 | -0.817482 |
| O | 2.032895 | -0.228133 | 2.780292 |
| O | 2.221197 | 3.079393 | 0.634902 |
| C | -1.014458 | 0.162146 | -0.384143 |
| C | 3.597376 | -0.051359 | -0.783443 |
| C | 3.348085 | 1.380673 | -0.495009 |
| C | 1.77179 | -1.463077 | 0.743089 |
| C | 0.493743 | 0.263062 | -0.468391 |
| H | 0.724846 | 0.863887 | -1.359921 |
| C | 1.147771 | 0.92621 | 0.802749 |
| C | 1.717704 | -0.276205 | 1.600518 |
| C | 1.172986 | -1.11782 | -0.599962 |
| C | 2.665552 | -1.12031 | -0.679541 |
| H | 3.055974 | -2.088164 | -0.993675 |
| C | -1.813385 | 0.948231 | -1.223314 |
| H | -1.338116 | 1.61814 | -1.944317 |
| C | 2.274784 | 1.872394 | 0.340588 |
| C | -1.654802 | -0.690142 | 0.531845 |

| | | | |
|---|---|---|---|
| H | -1.063161 | -1.3171 | 1.202196 |
| C | 0.193202 | 1.715325 | 1.697888 |
| H | -0.28312 | 2.533257 | 1.140804 |
| H | 0.756944 | 2.149318 | 2.533721 |
| H | -0.590888 | 1.06933 | 2.11084 |
| C | -3.045651 | -0.756969 | 0.61041 |
| H | -3.523601 | -1.42528 | 1.328426 |
| C | -3.814517 | 0.04275 | -0.240779 |
| C | 1.919179 | -2.827299 | 1.340404 |
| H | 2.744409 | -2.842473 | 2.06934 |
| H | 2.114721 | -3.591142 | 0.574663 |
| H | 0.998565 | -3.116599 | 1.877433 |
| C | 4.293877 | 2.360712 | -1.0932 |
| H | 5.322741 | 2.163132 | -0.745877 |
| H | 4.00892 | 3.386904 | -0.835919 |
| H | 4.321337 | 2.240745 | -2.191671 |
| C | -3.209983 | 0.898144 | -1.161251 |
| H | -3.812865 | 1.519284 | -1.825673 |
| C | 0.470663 | -2.162204 | -1.444086 |
| H | 1.084821 | -3.069042 | -1.540824 |
| H | 0.292475 | -1.766138 | -2.456927 |
| H | -0.499697 | -2.450207 | -1.018121 |
| C | 5.433764 | -1.614741 | -0.91993 |
| H | 5.128743 | -2.229032 | -0.055998 |
| H | 6.528575 | -1.532337 | -0.936534 |
| H | 5.094134 | -2.102124 | -1.849911 |

**[3]Int8**

UB3LYP-D3BJ/Def2-SVP Electronic Energy: -3573.630448 a.u.

UB3LYP-D3BJ/Def2-SVP Gibbs free Energy: -3573.324184 a.u.

UB3LYP-D3BJ/Def2-TZVP Electronic Energy: -3575.040133 a.u.

| | | | |
|---|---|---|---|
| Br | -5.680218 | -0.161717 | -0.341777 |
| O | 4.675393 | -1.163999 | -1.231278 |
| O | 1.788781 | 1.681438 | 2.62738 |
| O | 2.667451 | 2.93923 | -0.614159 |
| C | -0.96118 | -0.016754 | -0.316195 |
| C | 3.517346 | -0.643996 | -0.729115 |
| C | 3.493961 | 0.781564 | -0.885078 |
| C | 1.516561 | -0.537865 | 1.796118 |
| C | 0.550696 | 0.031091 | -0.351398 |
| H | 0.843655 | -0.04323 | -1.406151 |
| C | 1.156542 | 1.325561 | 0.274442 |
| C | 1.527597 | 0.896827 | 1.714213 |
| C | 1.237887 | -1.144379 | 0.440481 |
| C | 2.577249 | -1.489295 | -0.172969 |
| H | 2.830796 | -2.547165 | -0.126254 |
| C | -1.676127 | -0.062401 | -1.519986 |
| H | -1.131702 | -0.064175 | -2.467627 |
| C | 2.468592 | 1.736961 | -0.436441 |
| C | -1.693421 | -0.016599 | 0.884219 |
| H | -1.174637 | 0.017679 | 1.844566 |
| C | 0.228835 | 2.54014 | 0.311869 |
| H | -0.073093 | 2.837273 | -0.703236 |
| H | 0.751958 | 3.382862 | 0.777911 |
| H | -0.675068 | 2.327541 | 0.894862 |
| C | -3.087156 | -0.060647 | 0.88612 |
| H | -3.635131 | -0.060838 | 1.829597 |
| C | -3.768958 | -0.103656 | -0.333938 |
| C | 1.825892 | -1.303046 | 3.0206 |
| H | 2.078823 | -0.623775 | 3.846985 |
| H | 2.668589 | -1.997545 | 2.850612 |
| H | 0.967745 | -1.929279 | 3.3245 |
| C | 4.684789 | 1.385865 | -1.57151 |
| H | 5.610289 | 1.170473 | -1.01071 |
| H | 4.563679 | 2.469352 | -1.665588 |
| H | 4.830824 | 0.939801 | -2.569097 |
| C | -3.074159 | -0.104944 | -1.542545 |
| H | -3.60824 | -0.139019 | -2.493419 |
| C | 0.411108 | -2.437591 | 0.504691 |
| H | 0.991265 | -3.234734 | 0.992996 |
| H | 0.158197 | -2.773868 | -0.512451 |
| H | -0.52226 | -2.30304 | 1.063944 |
| C | 4.932664 | -2.554152 | -1.179468 |
| H | 4.968284 | -2.927025 | -0.141232 |
| H | 5.917311 | -2.698041 | -1.64446 |
| H | 4.181053 | -3.132602 | -1.743761 |

**[3]TS10**

UB3LYP-D3BJ/Def2-SVP Electronic Energy: -3573.620538 a.u.

UB3LYP-D3BJ/Def2-SVP Gibbs free Energy: -3573.312211 a.u.

UB3LYP-D3BJ/Def2-TZVP Electronic Energy: -3575.031365 a.u.

| | | | |
|---|---|---|---|
| Br | -5.513443 | -0.537964 | 0.016193 |
| O | 4.836374 | -1.098445 | -1.468264 |
| O | 1.647226 | 1.994092 | 2.284923 |
| O | 2.953625 | 2.765857 | -1.040854 |
| C | -0.827949 | -0.102932 | -0.369562 |
| C | 3.704904 | -0.717222 | -0.819118 |
| C | 3.759589 | 0.676946 | -0.396682 |
| C | 2.012391 | -0.242689 | 1.488445 |
| C | 0.670623 | 0.015025 | -0.534263 |
| H | 0.885929 | -0.140878 | -1.598048 |
| C | 1.235145 | 1.390665 | -0.077352 |
| C | 1.60591 | 1.141364 | 1.404231 |
| C | 1.513852 | -1.052635 | 0.305441 |
| C | 2.659773 | -1.542631 | -0.550526 |
| H | 2.605343 | -2.552748 | -0.955741 |
| C | -1.615702 | -0.44698 | -1.476196 |
| H | -1.136066 | -0.615518 | -2.443684 |
| C | 2.656693 | 1.661226 | -0.610719 |
| C | -1.477205 | 0.107998 | 0.859622 |
| H | -0.902798 | 0.377634 | 1.748144 |
| C | 0.346799 | 2.600024 | -0.310729 |
| H | 0.156889 | 2.745341 | -1.384865 |
| H | 0.837046 | 3.502929 | 0.075277 |

| | | | |
|---|---|---|---|
| H | -0.617938 | 2.488259 | 0.199238 |
| C | -2.860699 | -0.017813 | 0.982532 |
| H | -3.34309 | 0.151365 | 1.946563 |
| C | -3.61601 | -0.362872 | -0.142346 |
| C | 2.646918 | -0.844415 | 2.67693 |
| H | 3.040021 | -0.068875 | 3.349674 |
| H | 3.45882 | -1.530757 | 2.378301 |
| H | 1.919914 | -1.454031 | 3.245944 |
| C | 5.11932 | 1.282111 | -0.237698 |
| H | 5.79564 | 0.585132 | 0.27811 |
| H | 5.064641 | 2.230449 | 0.314652 |
| H | 5.574958 | 1.499263 | -1.221467 |
| C | -3.004376 | -0.579727 | -1.376525 |
| H | -3.59586 | -0.847579 | -2.253607 |
| C | 0.70049 | -2.276075 | 0.763901 |
| H | 1.368915 | -3.012788 | 1.234236 |
| H | 0.219572 | -2.755141 | -0.102273 |
| H | -0.082628 | -2.010459 | 1.484896 |
| C | 4.981748 | -2.460087 | -1.830212 |
| H | 4.896797 | -3.12033 | -0.94927 |
| H | 5.983761 | -2.562265 | -2.268123 |
| H | 4.228963 | -2.764018 | -2.577743 |

**16**

B3LYP-D3BJ/Def2-SVP Electronic Energy: -3573.694551 a.u.

B3LYP-D3BJ/Def2-SVP Gibbs free Energy: -3573.381561 a.u.

B3LYP-D3BJ/Def2-TZVP Electronic Energy: -3575.102542 a.u.

| | | | |
|---|---|---|---|
| Br | 5.724648 | 0.269159 | 0.062039 |
| O | -4.591986 | 1.650352 | -0.35028 |
| O | -1.054439 | -2.872719 | 0.722095 |
| O | -3.096077 | -0.715063 | -2.410753 |
| C | 1.018799 | 0.21135 | -0.329539 |
| C | -3.459634 | 1.105082 | 0.109649 |
| C | -3.330427 | -0.407255 | 0.019718 |
| C | -2.199747 | -0.70889 | 1.070929 |
| C | -0.485466 | 0.266975 | -0.459412 |
| H | -0.707621 | 1.06176 | -1.1816 |
| C | -1.138625 | -1.074751 | -0.98521 |
| C | -1.392044 | -1.77392 | 0.354909 |
| C | -1.308434 | 0.587744 | 0.888227 |
| C | -2.33564 | 1.663853 | 0.592741 |
| H | -2.117999 | 2.727625 | 0.687011 |
| C | 1.778987 | 1.259281 | -0.870479 |
| H | 1.271987 | 2.074555 | -1.392638 |
| C | -2.596513 | -0.717791 | -1.31205 |
| C | 1.704032 | -0.823847 | 0.329899 |
| H | 1.158763 | -1.663135 | 0.761305 |
| C | -0.421367 | -1.8236 | -2.082526 |
| H | -0.301491 | -1.184902 | -2.970659 |
| H | -0.997441 | -2.713249 | -2.378775 |
| H | 0.574409 | -2.154028 | -1.758283 |
| C | 3.094563 | -0.814149 | 0.449119 |
| H | 3.604614 | -1.629891 | 0.96399 |
| C | 3.819447 | 0.248228 | -0.096647 |
| C | -2.658944 | -1.042116 | 2.470644 |
| H | -3.279167 | -1.950509 | 2.469876 |
| H | -3.250782 | -0.220346 | 2.901072 |
| H | -1.799228 | -1.23025 | 3.129659 |
| C | -4.63361 | -1.183755 | 0.137294 |
| H | -5.165304 | -0.926347 | 1.064582 |
| H | -4.444241 | -2.268231 | 0.128662 |
| H | -5.284848 | -0.940563 | -0.714237 |
| C | 3.171596 | 1.291031 | -0.759162 |
| H | 3.739004 | 2.118733 | -1.187771 |
| C | -0.427749 | 0.929292 | 2.08282 |
| H | -1.049134 | 1.200606 | 2.949154 |
| H | 0.217093 | 1.790085 | 1.847969 |
| H | 0.223812 | 0.093805 | 2.374372 |
| C | -4.6582 | 3.070237 | -0.35573 |
| H | -4.549365 | 3.47336 | 0.665795 |
| H | -5.643817 | 3.343286 | -0.754617 |
| H | -3.868031 | 3.4985 | -0.995891 |

**Part 16. Analytical Data and Spectra**

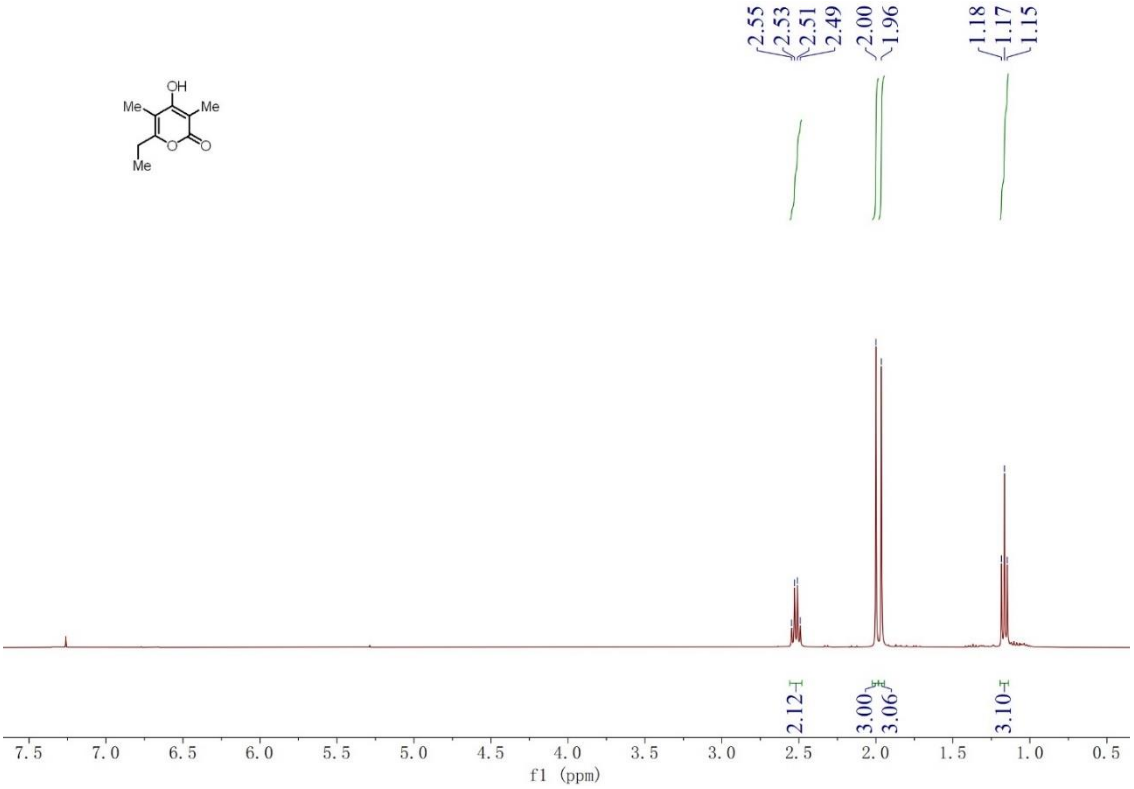


$^{1}$H NMR spectrum of compound **SI-1** (400MHz, $CDCl_3$)

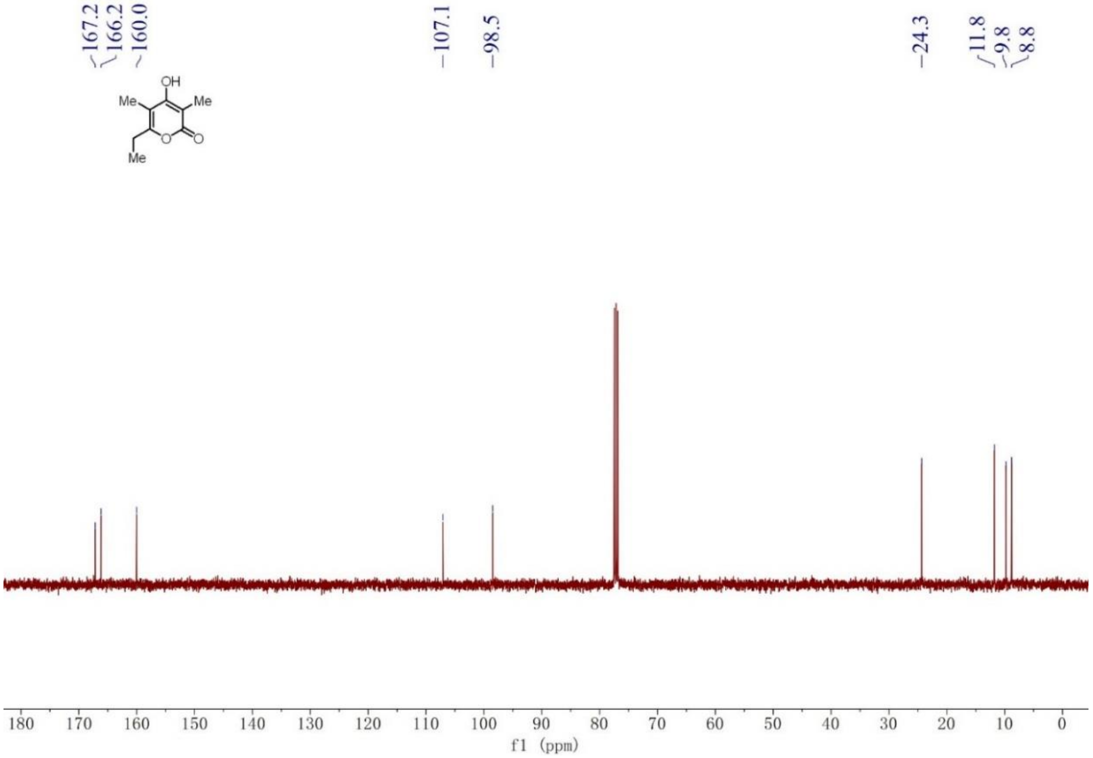


$^{13}$C NMR spectrum of compound **SI-1** (101MHz, $CDCl_3$)

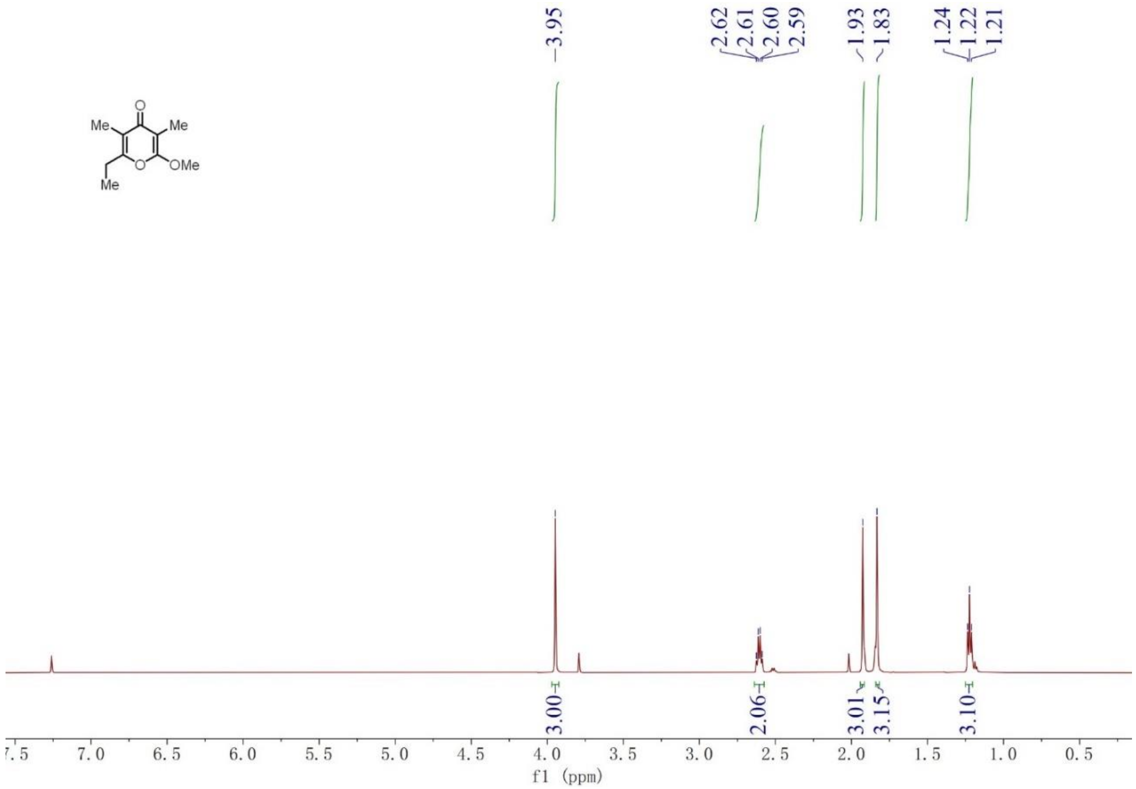


$^{1}H$ NMR spectrum of compound **3** (600MHz, $CDCl_3$)

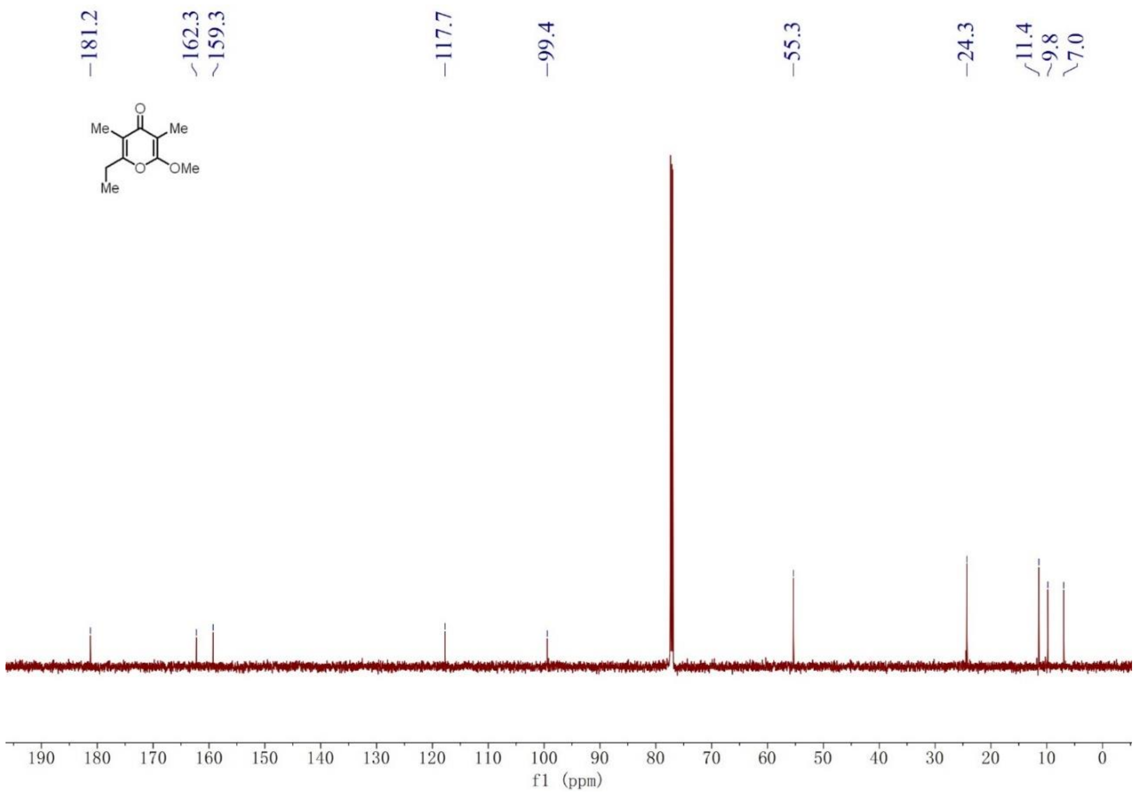


$^{13}C$ NMR spectrum of compound **3** (151MHz, $CDCl_3$)

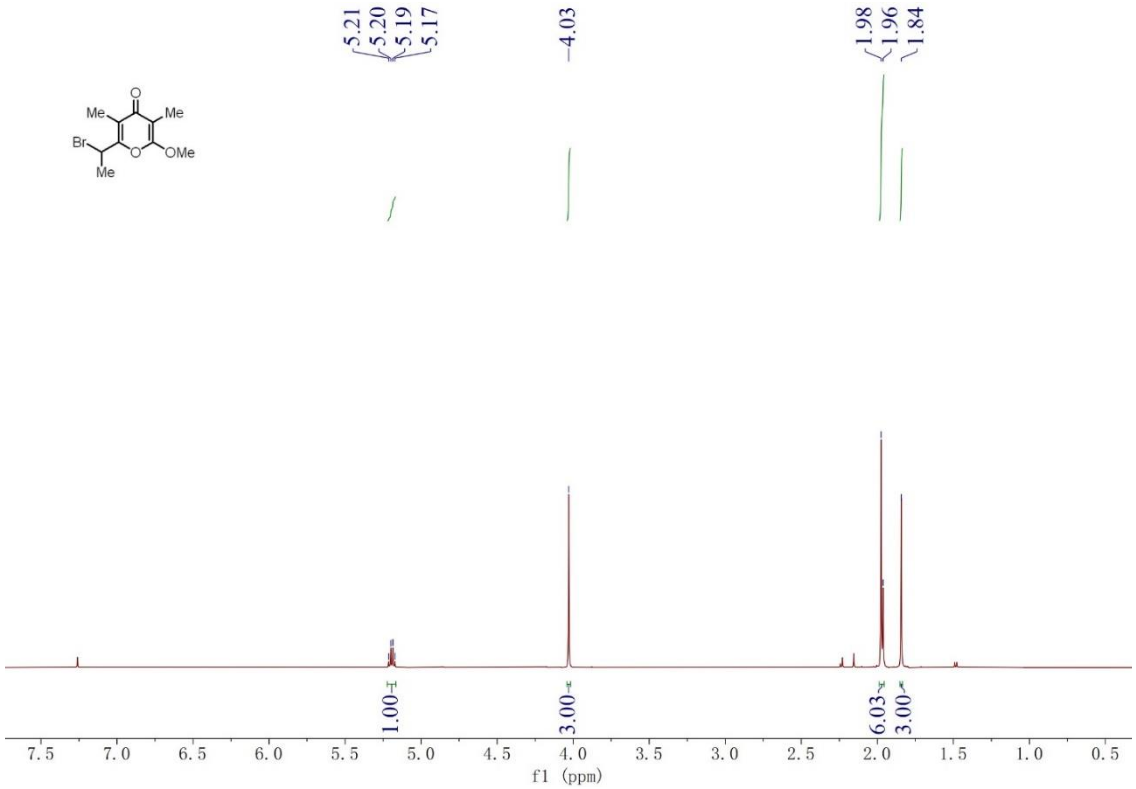


$^{1}H$ NMR spectrum of compound **4** (500MHz, $CDCl_3$)

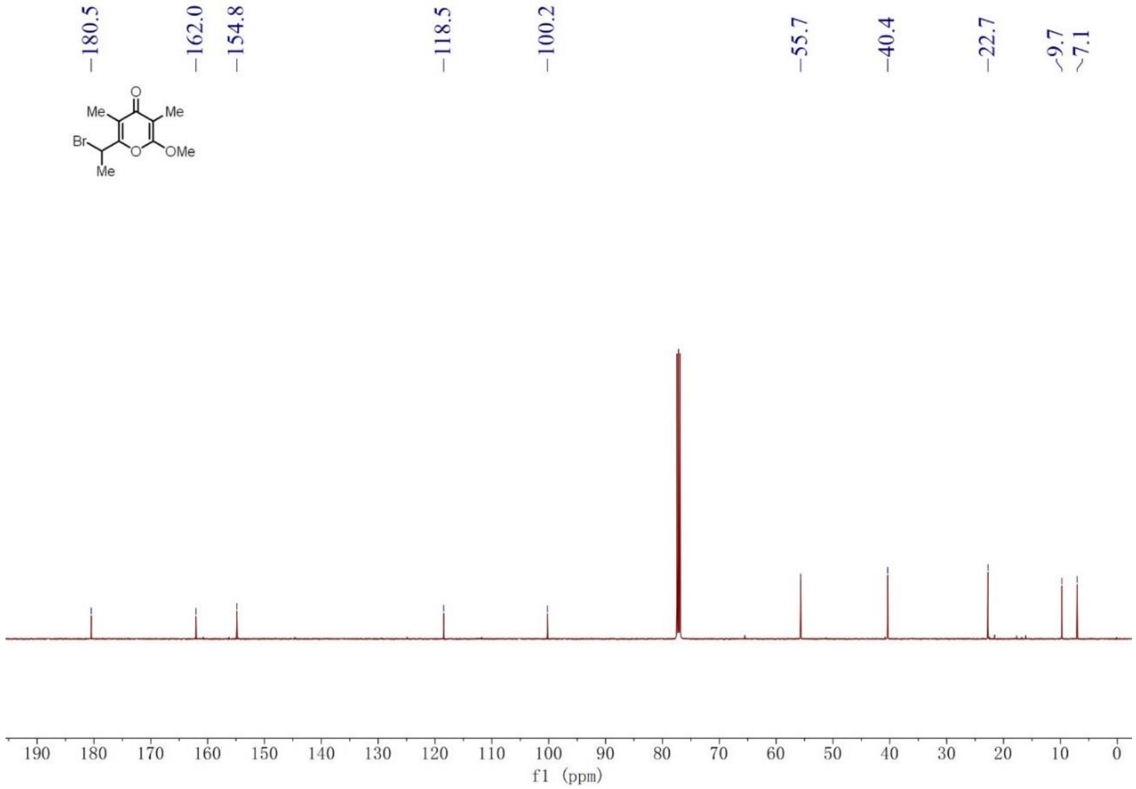


$^{13}C$ NMR spectrum of compound **4** (126MHz, $CDCl_3$)

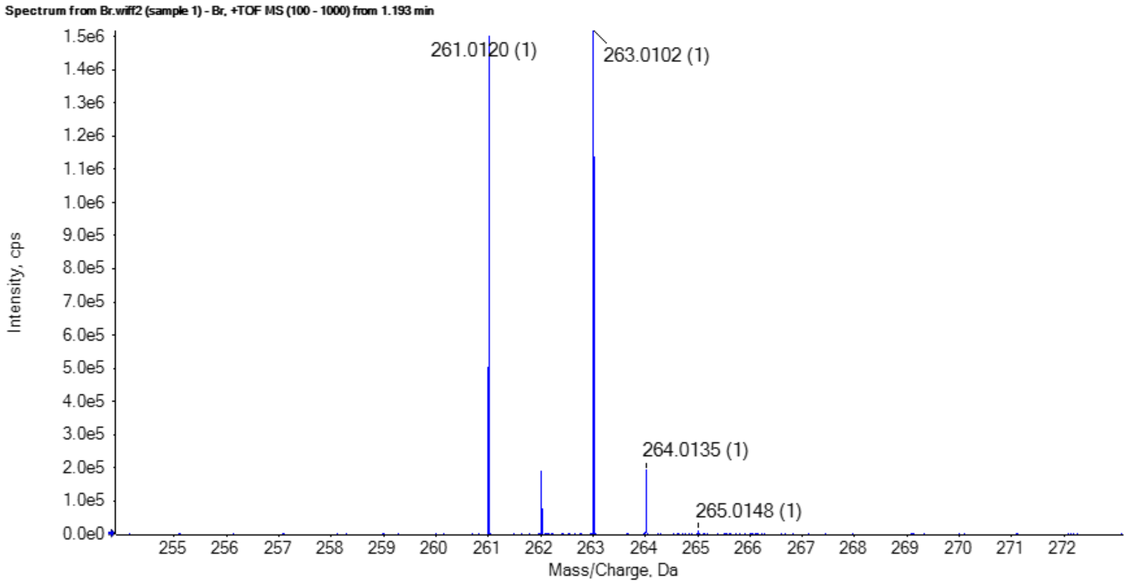


## Formula Calculator Results

| Measured m/z | Cal m/z | Error(mmu) | Error(ppm) | Ion Formula | Ion |
|---|---|---|---|---|---|
| 261.0120 | 261.0121 | -0.1 | -0.2 | C10H14BrO3 | $[M+H]^+$ |

HRESIMS spectrum of compound **4**

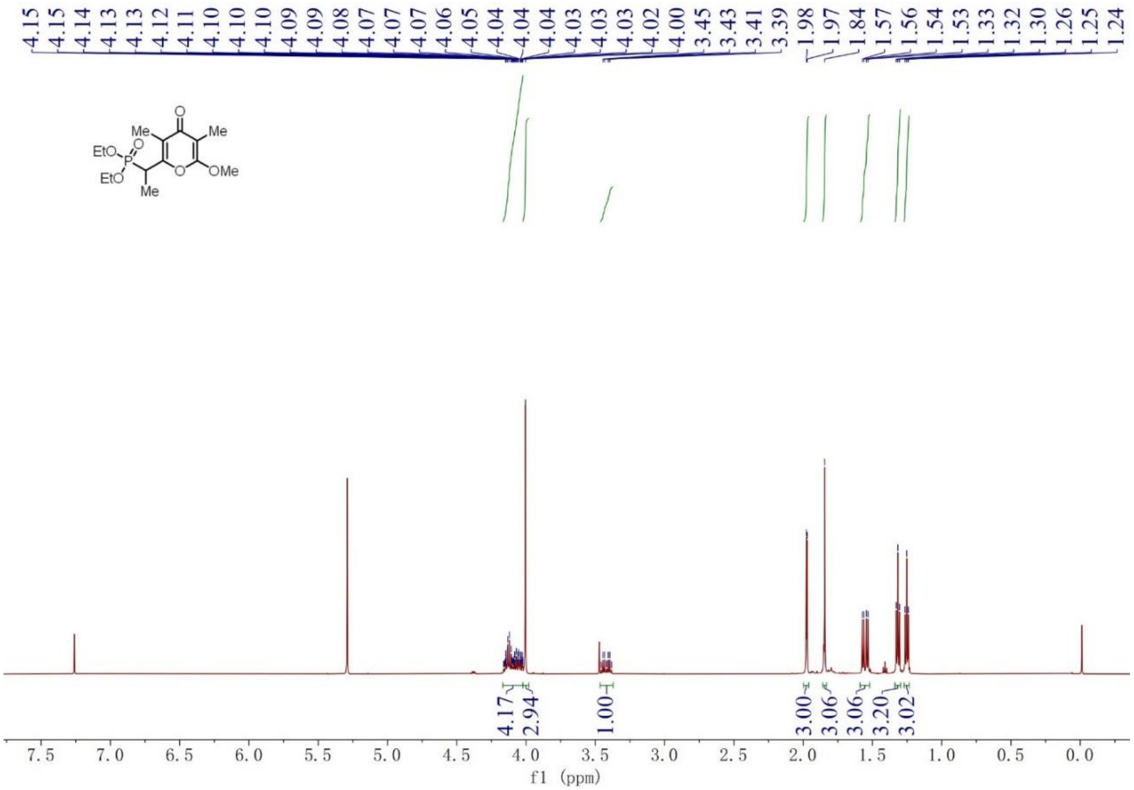


$^1$H NMR spectrum of compound **5** (600MHz, $CDCl_3$)

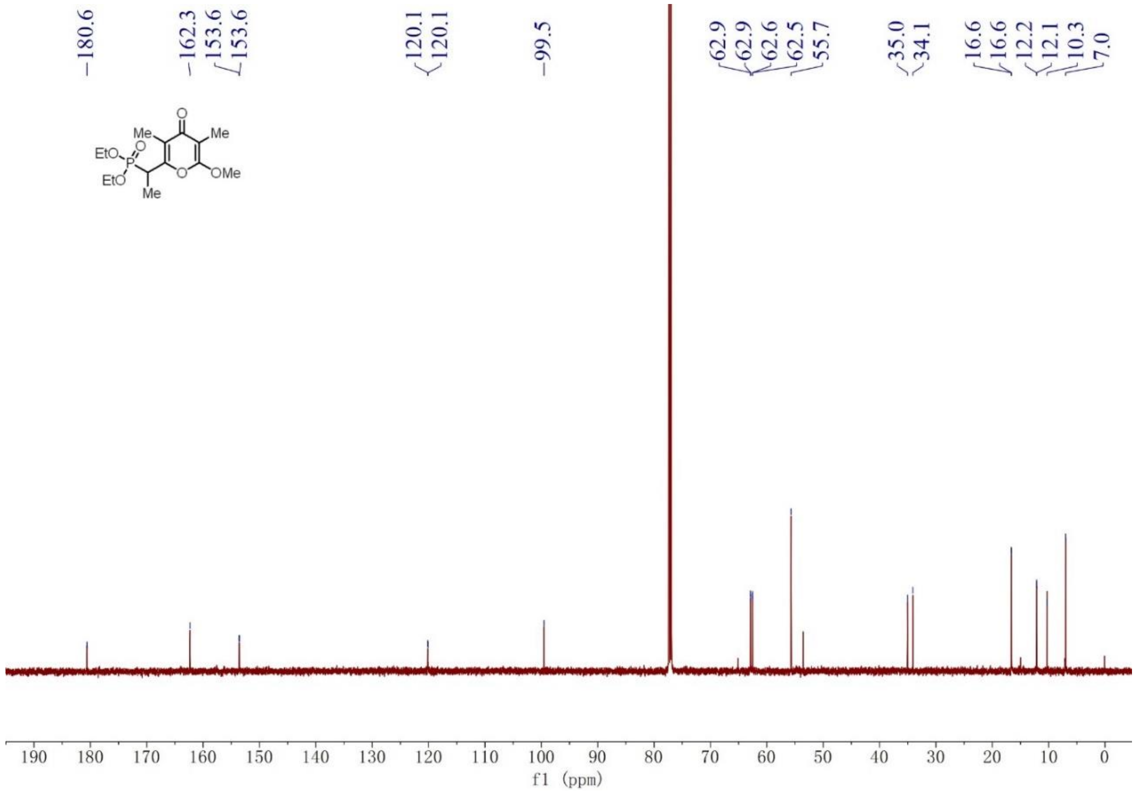


$^{13}C$ NMR spectrum of compound **5** (151MHz, $CDCl_3$)

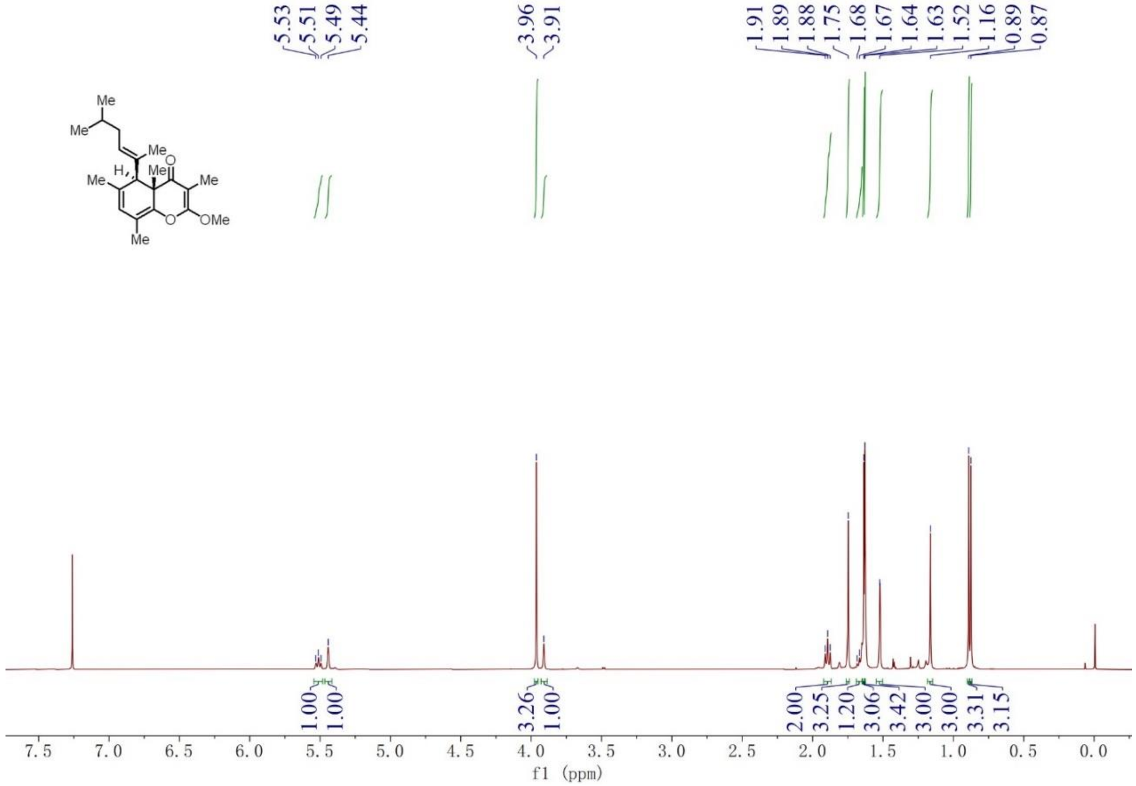


$^{1}H$ NMR spectrum of compound **2** (400MHz, $CDCl_3$)

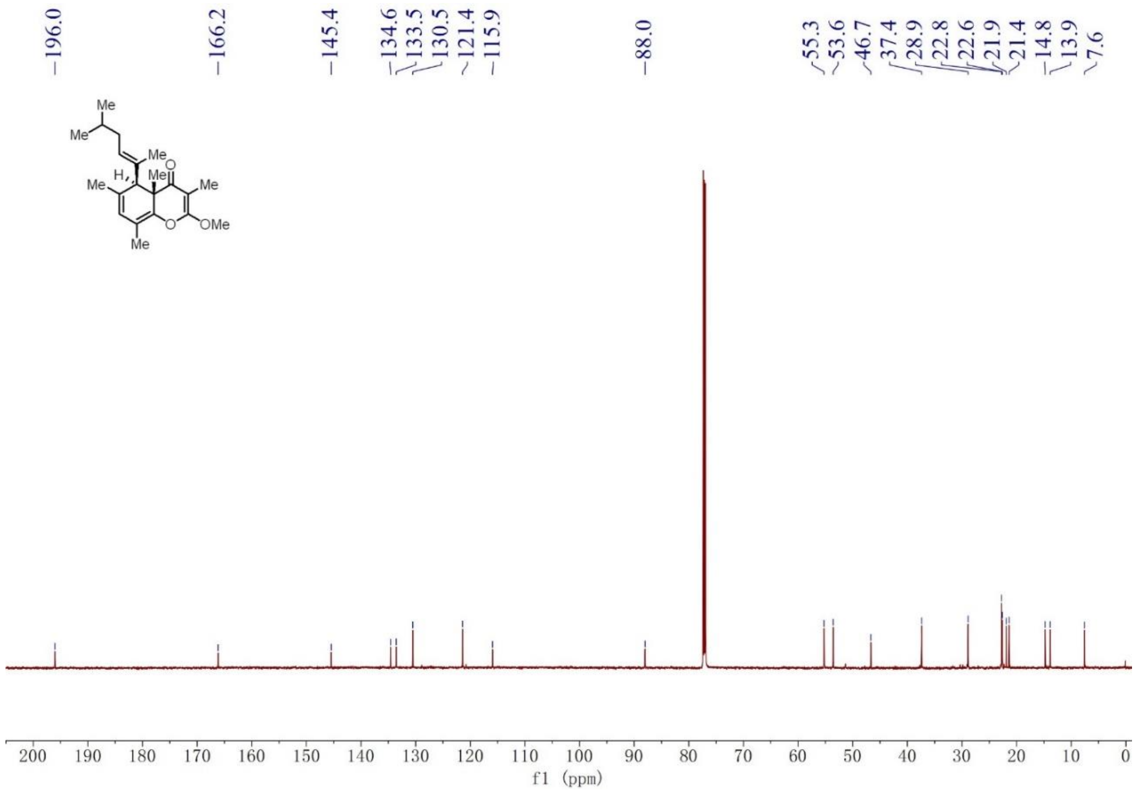


$^{13}$C NMR spectrum of compound **2** (151MHz, $CDCl_3$)

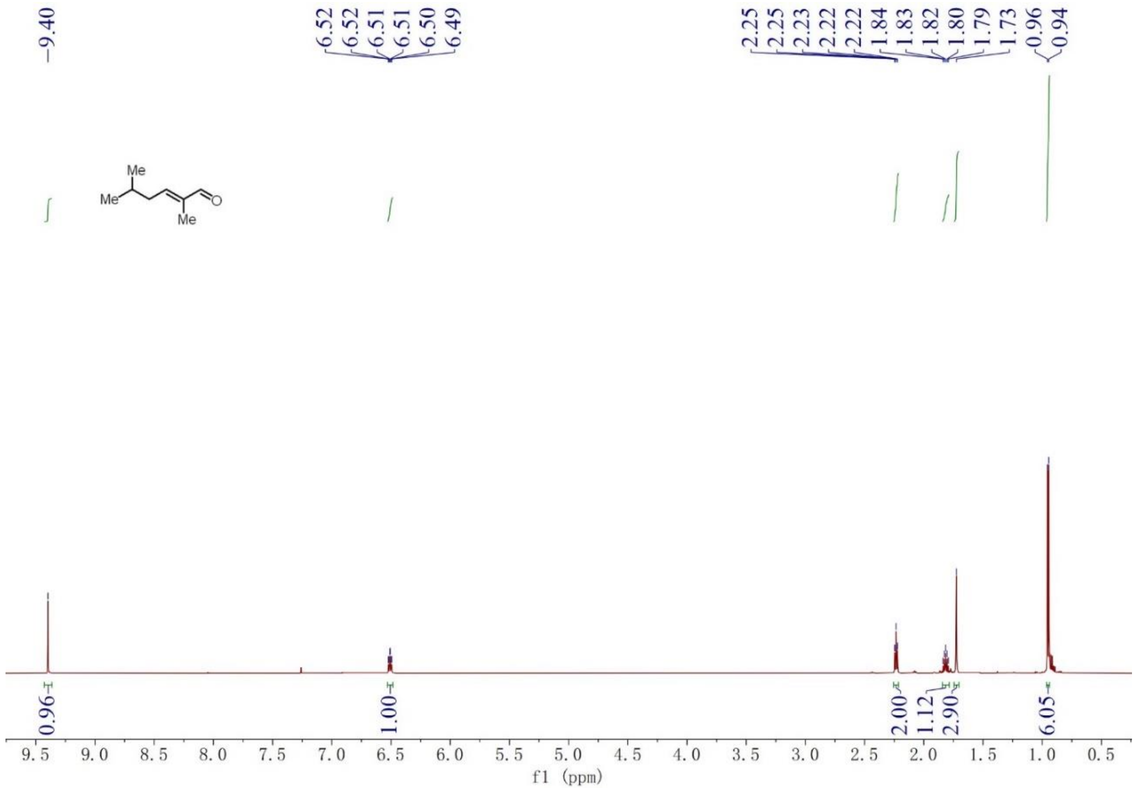


$^{1}$H NMR spectrum of compound **SI-6** (600MHz, $CDCl_3$)

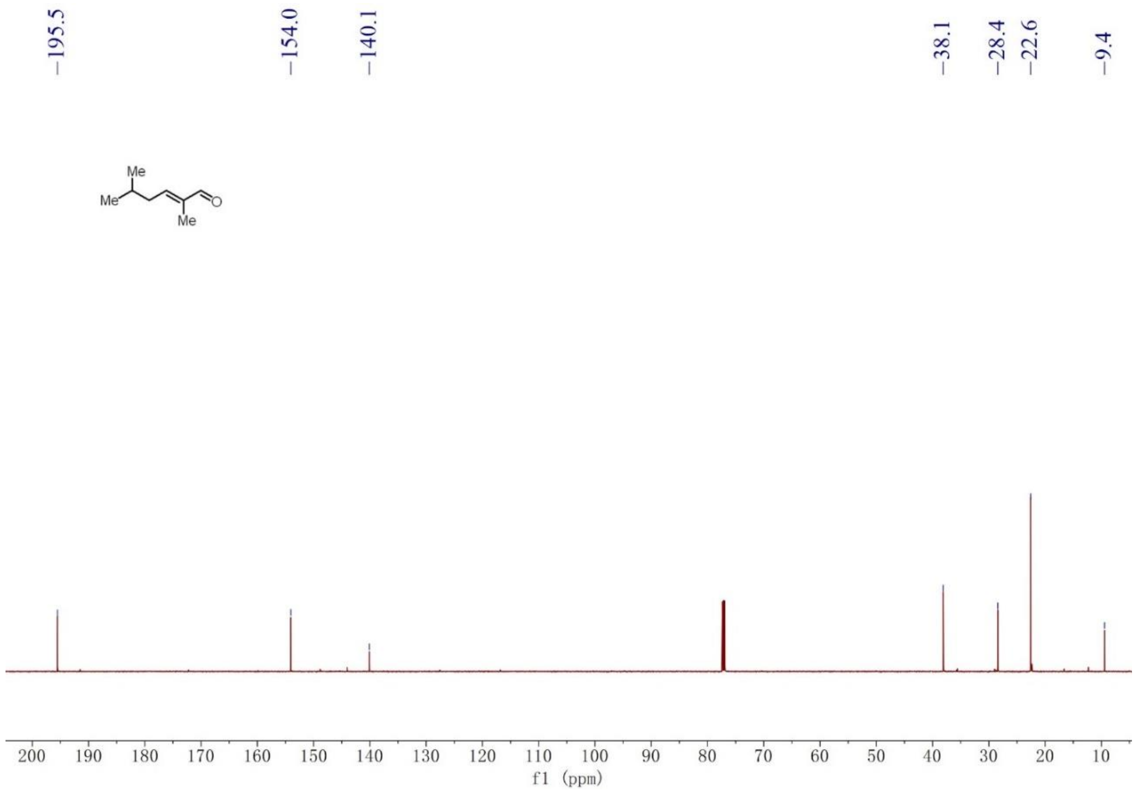


$^{13}$C NMR spectrum of compound **SI-6** (151MHz, $CDCl_3$)

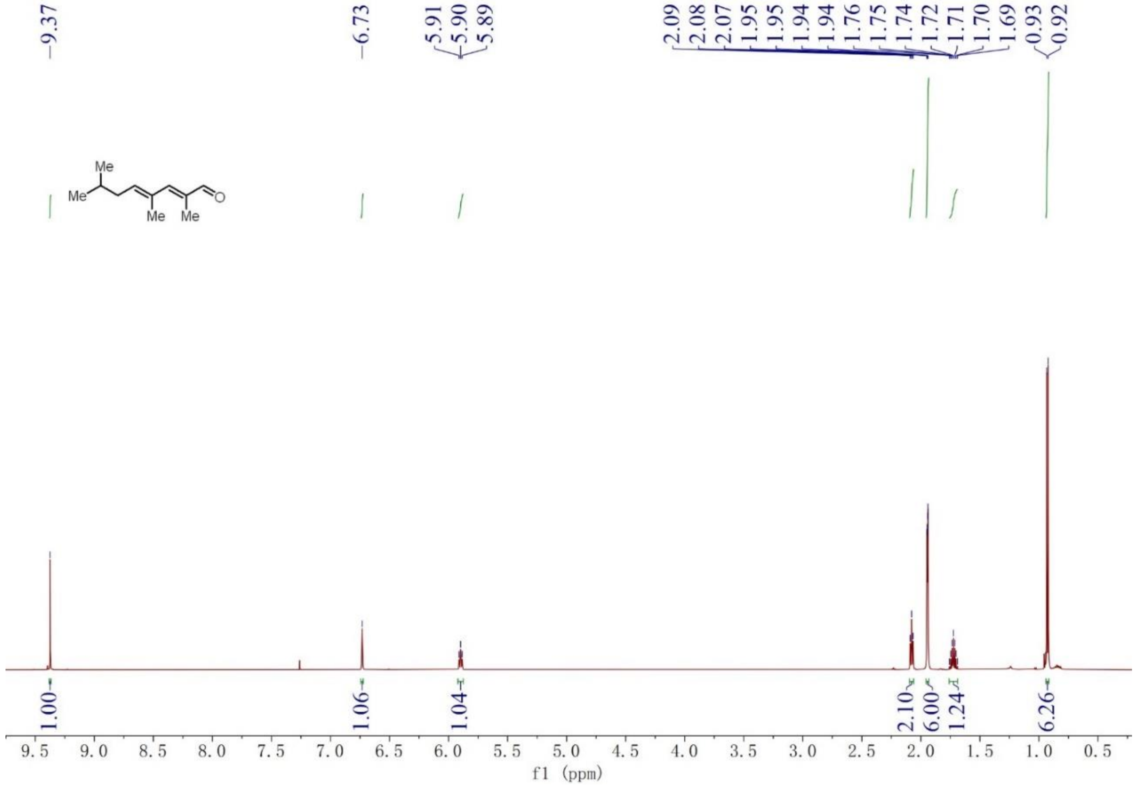


$^{1}$H NMR spectrum of compound **6** (600MHz, $CDCl_3$)

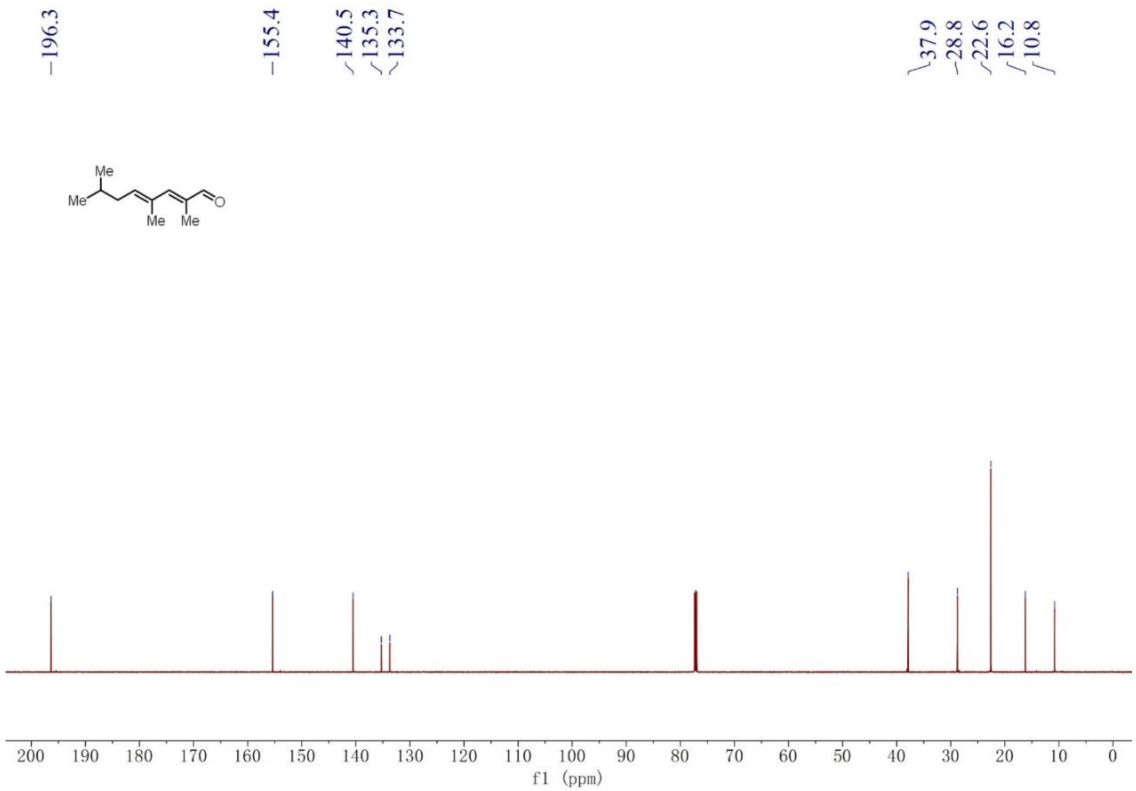


$^{13}$C NMR spectrum of compound **6** (151MHz, $CDCl_3$)

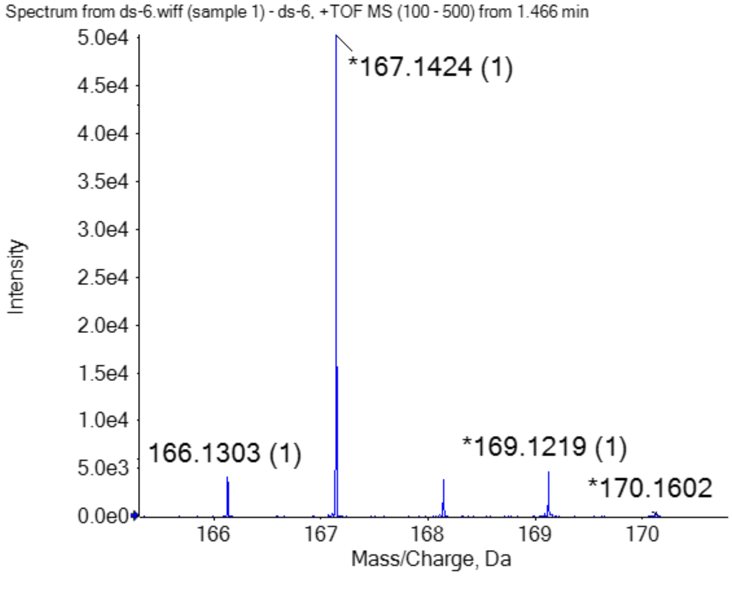


**Formula Calculator Results**

| Measured m/z | Cal m/z | Error(mmu) | Error(ppm) | Ion Formula | Ion |
|---|---|---|---|---|---|
| 167.1424 | 167.1430 | -0.6 | -3.8 | C11H19O | $[M+H]^+$ |

HRESIMS spectrum of compound **6**

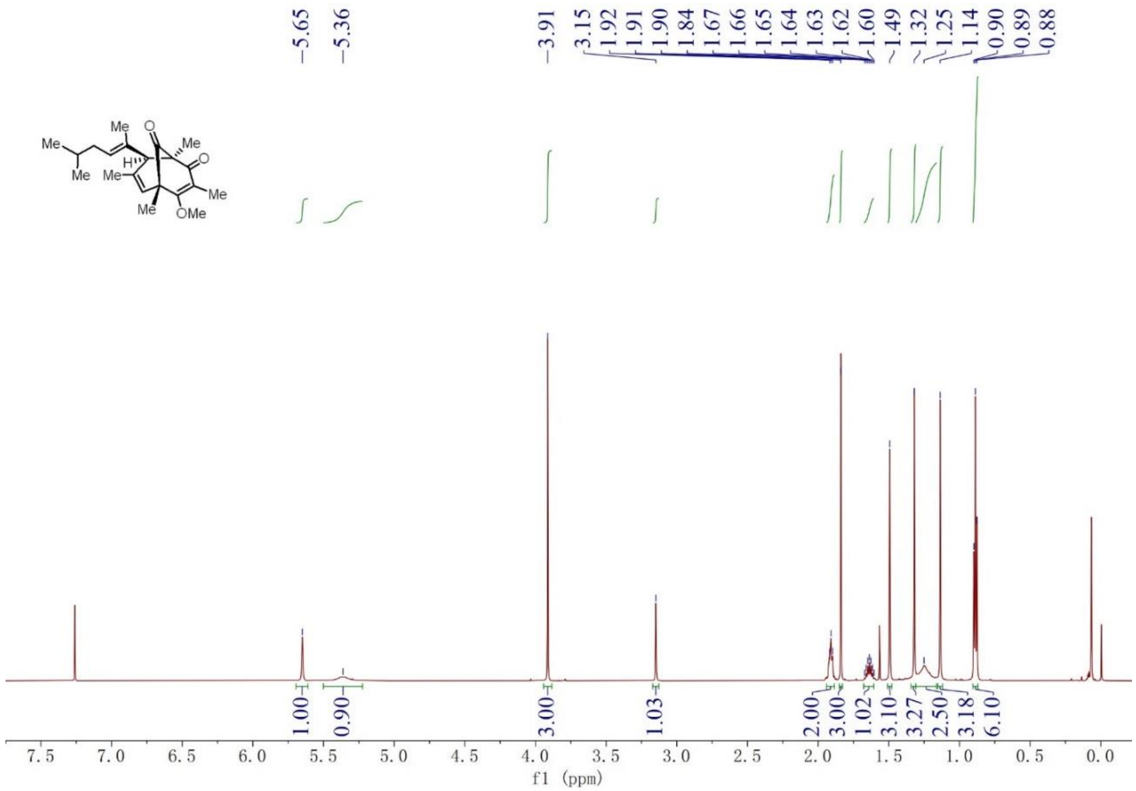


$^{1}$H NMR spectrum of compound **1** (600MHz, $CDCl_3$)

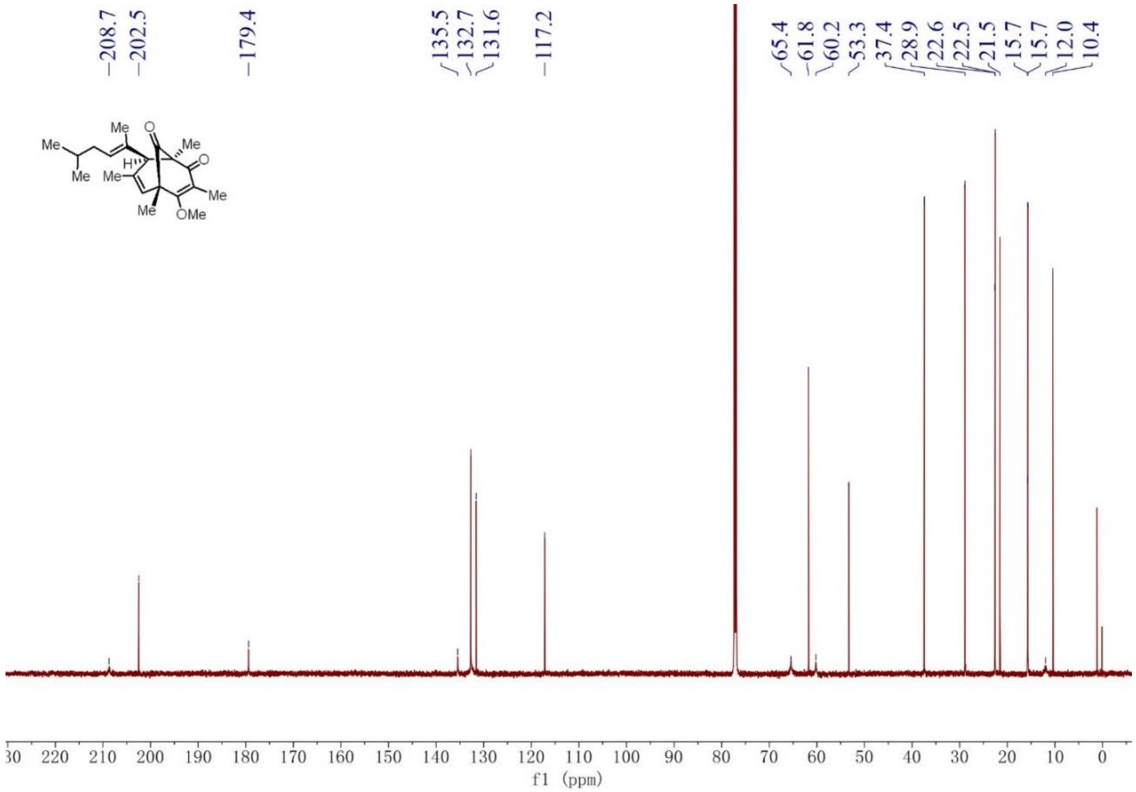


$^{13}$C NMR spectrum of compound **1** (151MHz, $CDCl_3$)

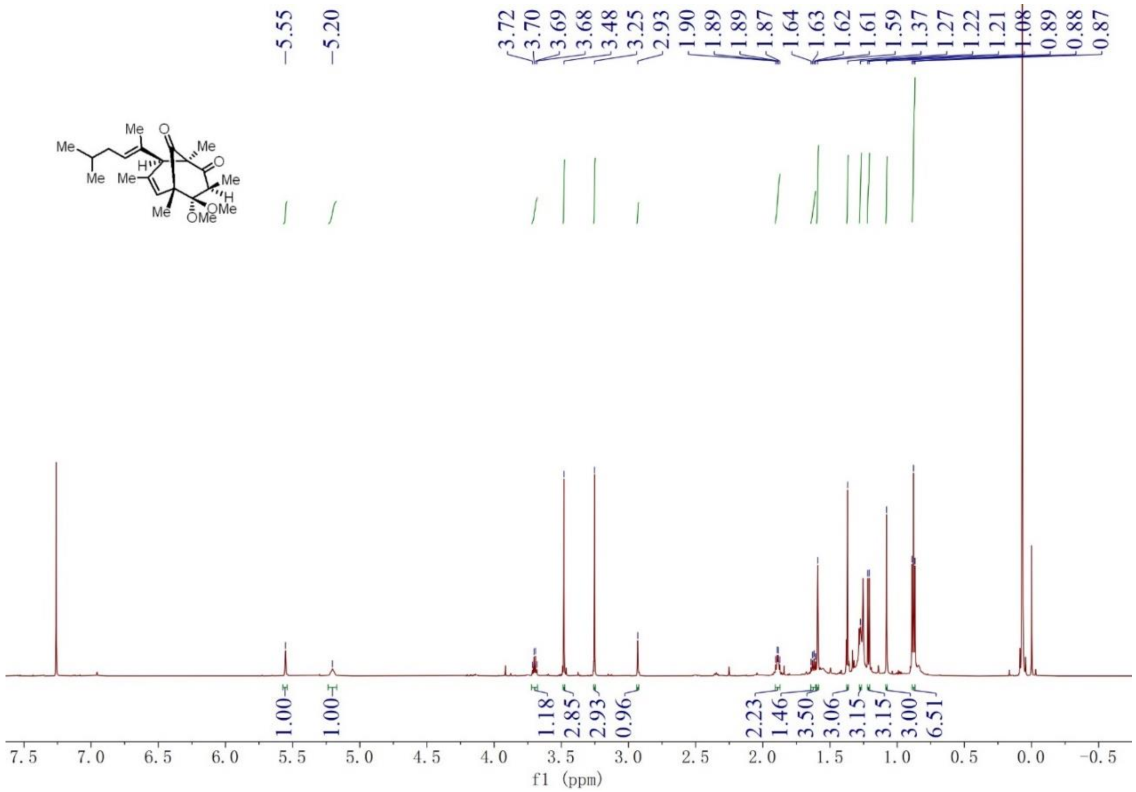


$^1H$ NMR spectrum of compound **1-1** (600MHz, $CDCl_3$)

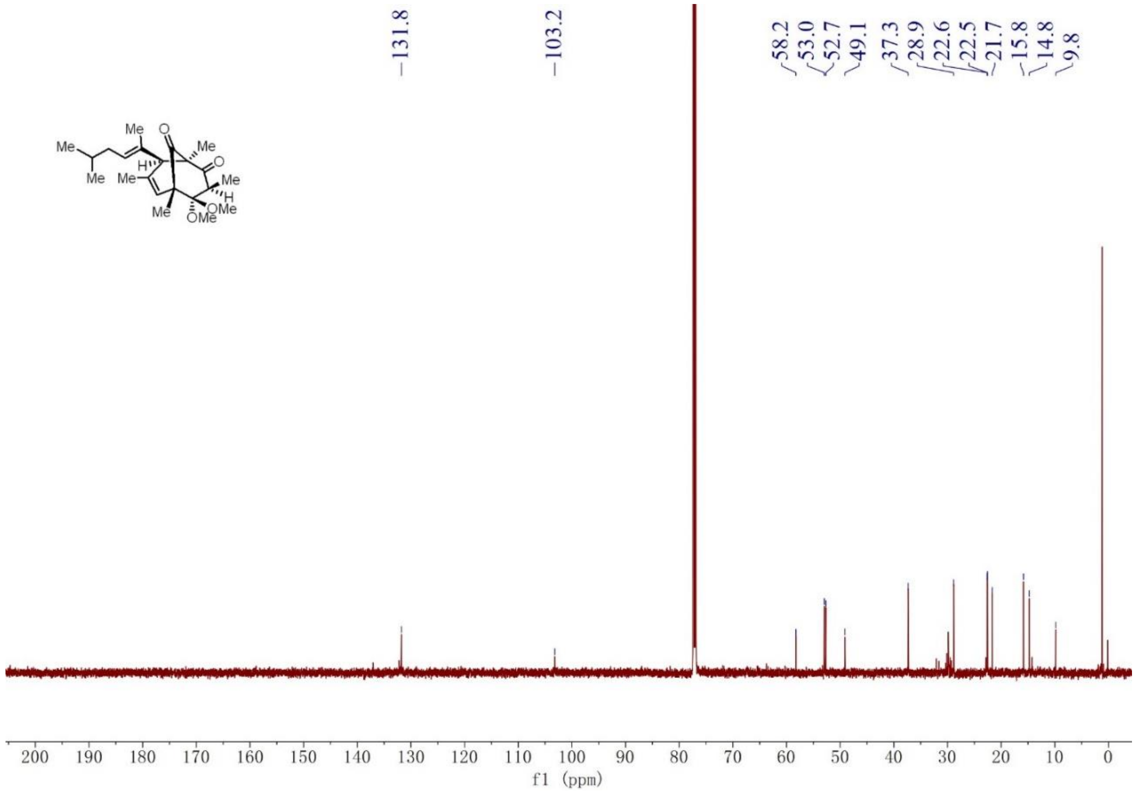


$^{13}C$ NMR spectrum of compound **1-1** (151MHz, $CDCl_3$)

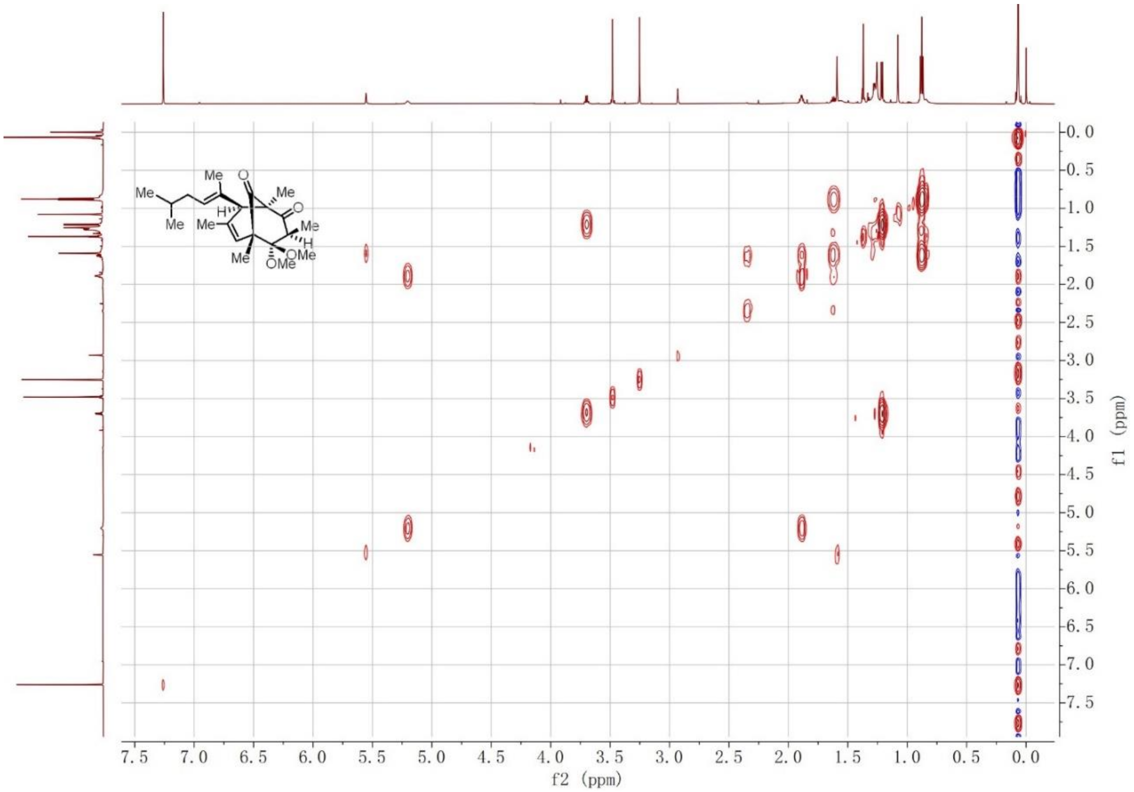


$^{1}$H-$^{1}$H COSY spectrum of compound **1-1** (600 MHz, $CDCl_3$)

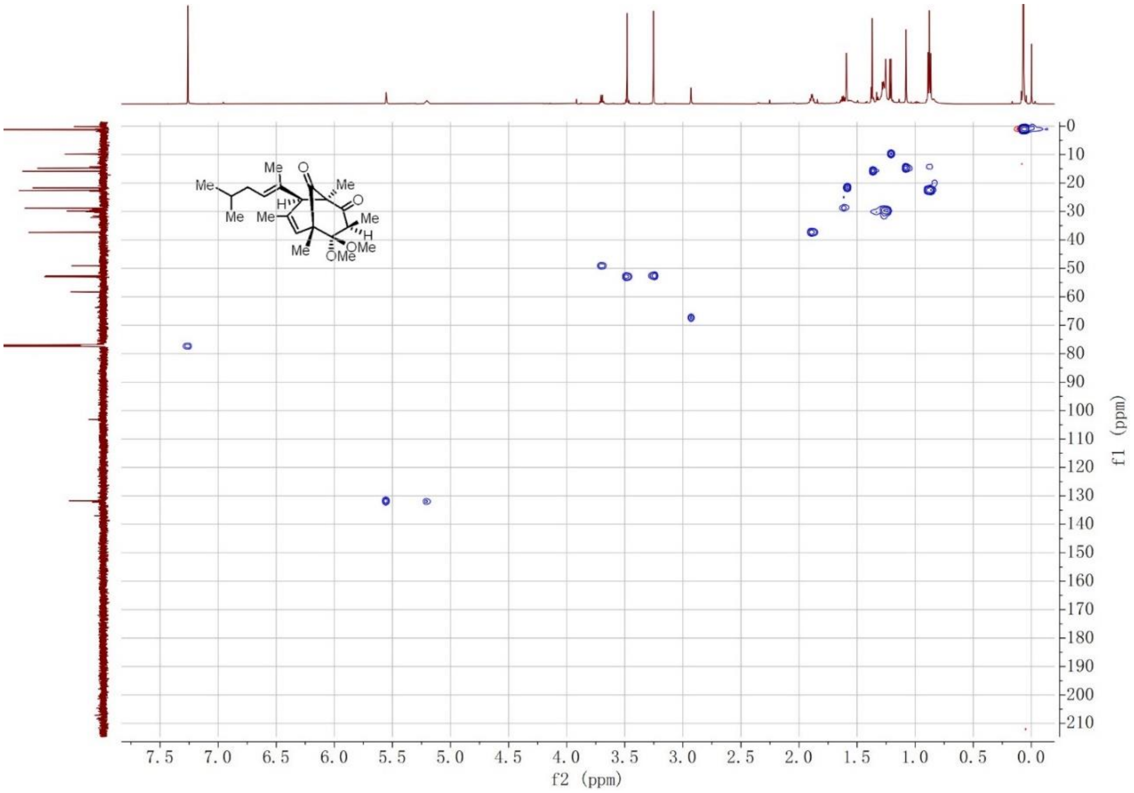


HSQC spectrum of compound **1-1** (600 MHz, $CDCl_3$)

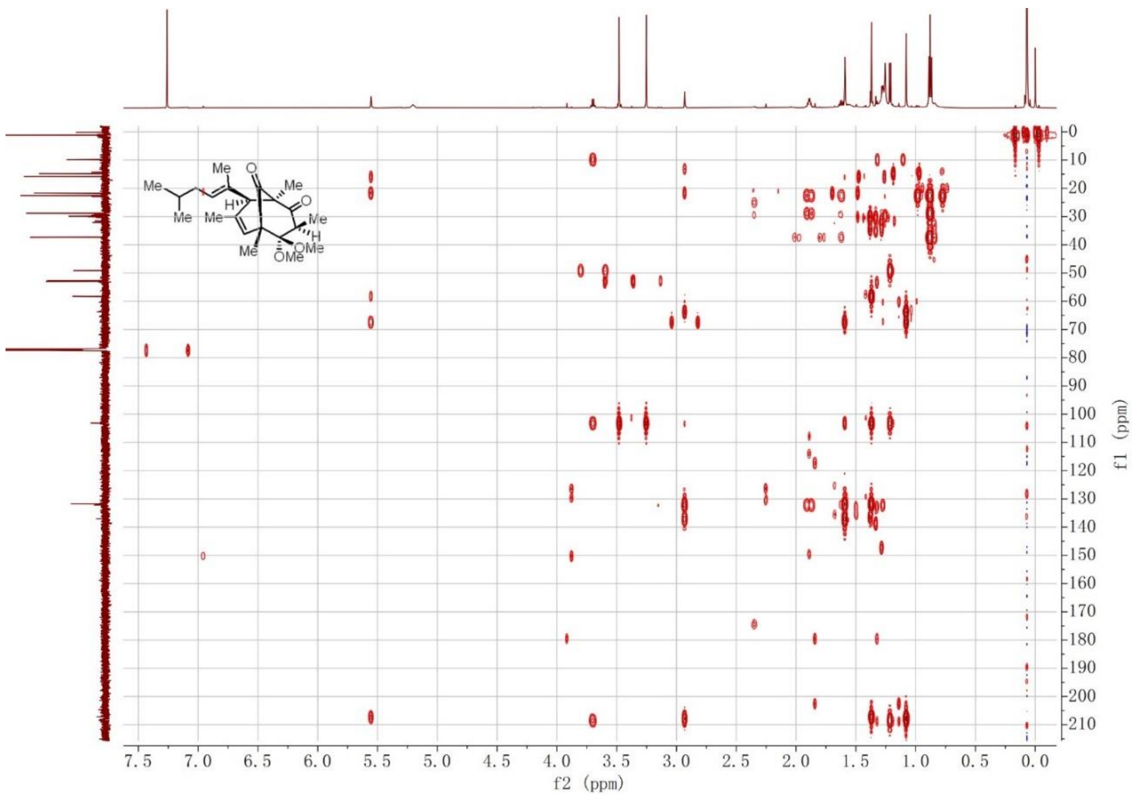


HMBC spectrum of compound **1-1** (600 MHz, $CDCl_3$)

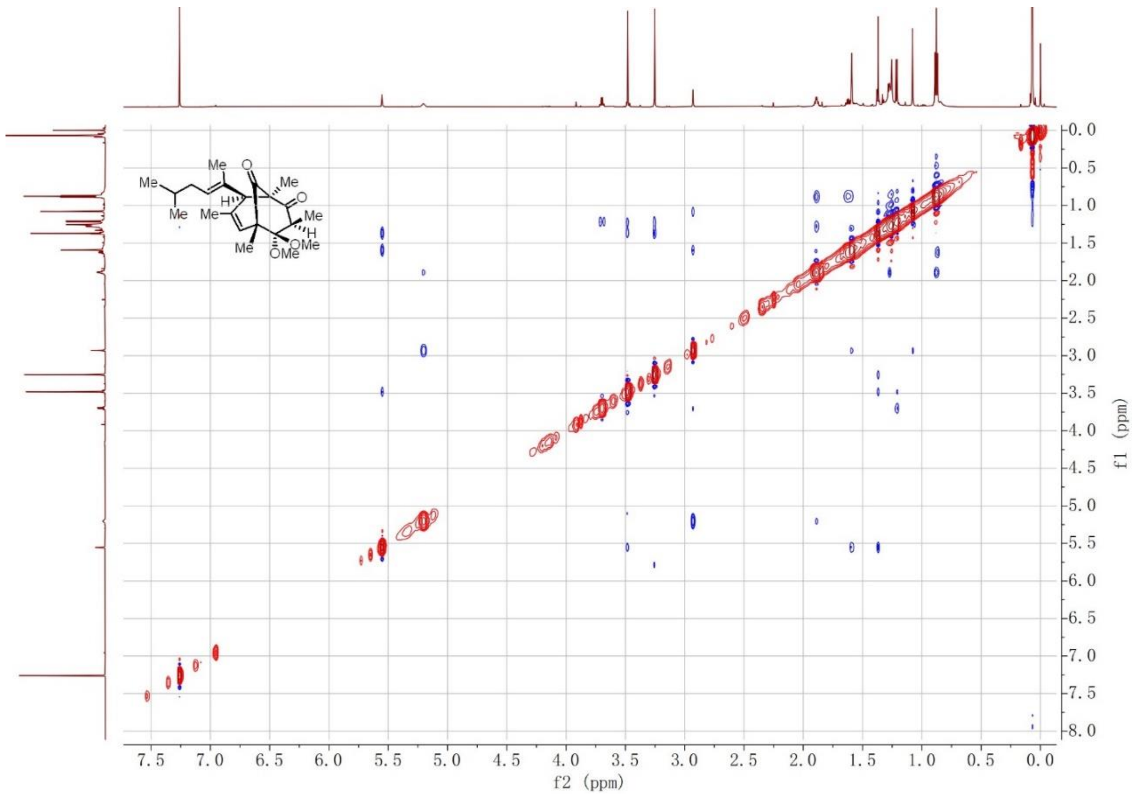


NOESY spectrum of compound **1-1** (600 MHz, $CDCl_3$)

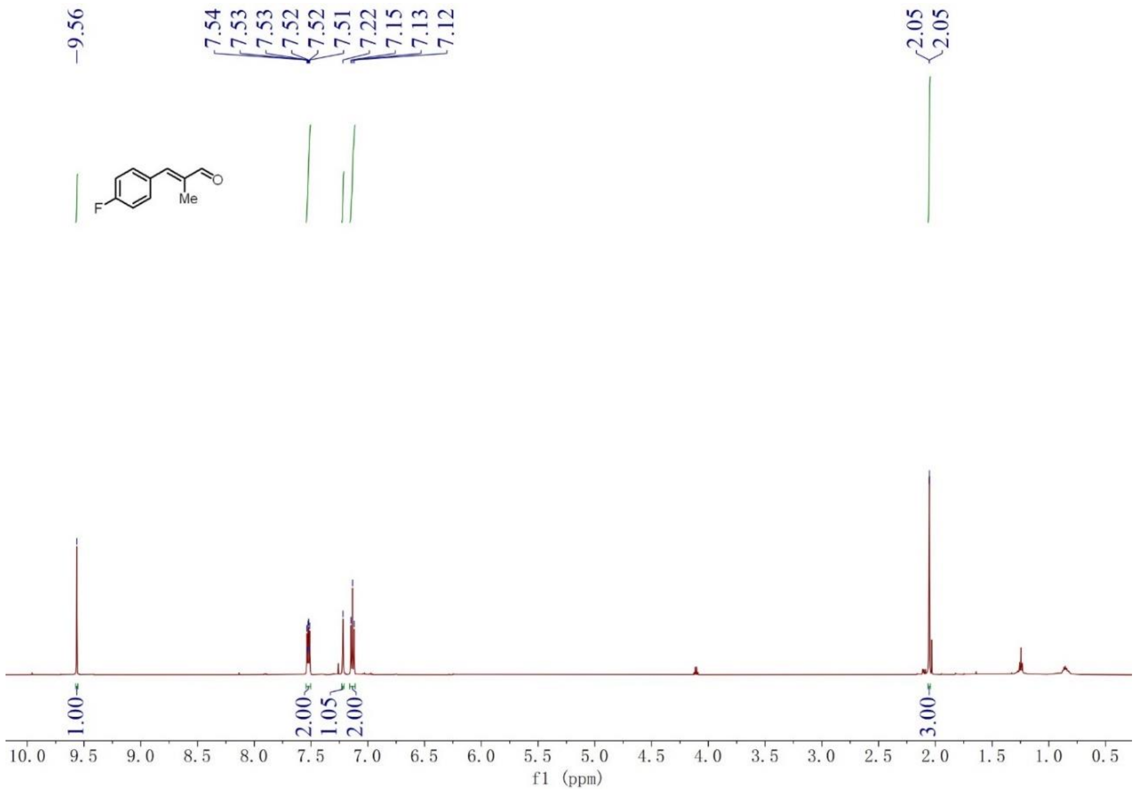


$^{1}$H NMR spectrum of compound **8b** (600MHz, $CDCl_3$)

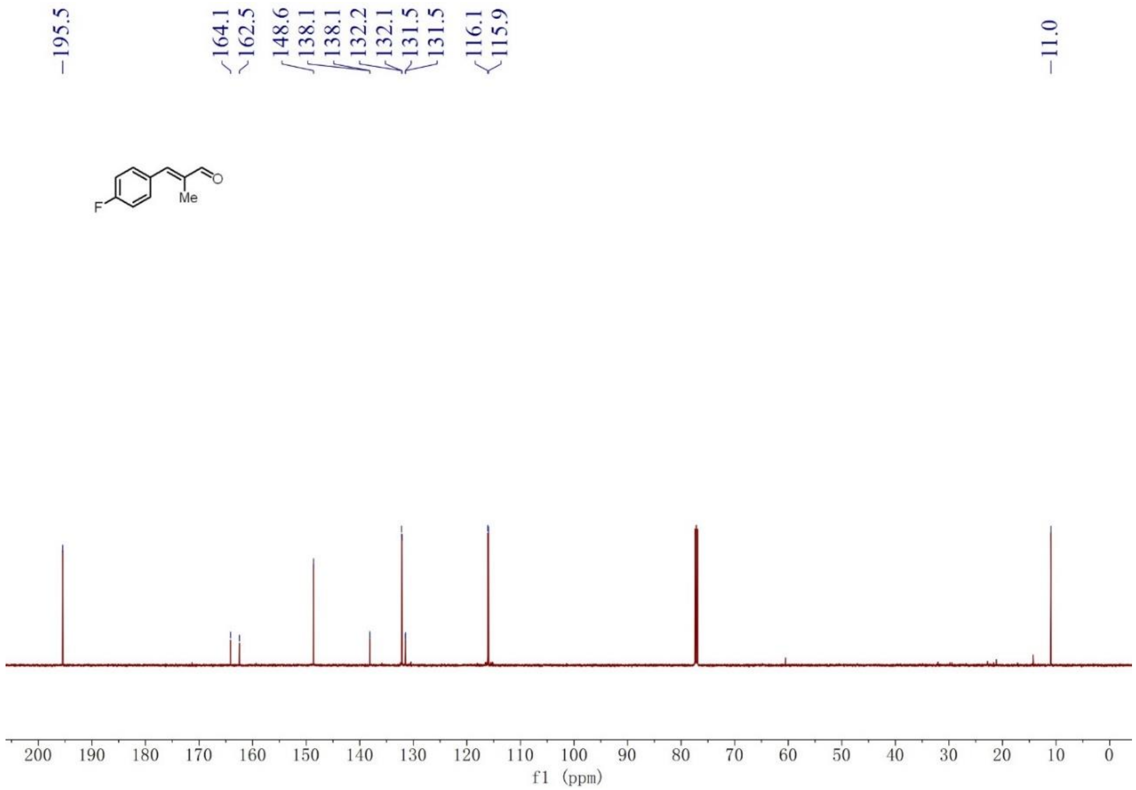


$^{13}$C NMR spectrum of compound **8b** (151MHz, $CDCl_3$)

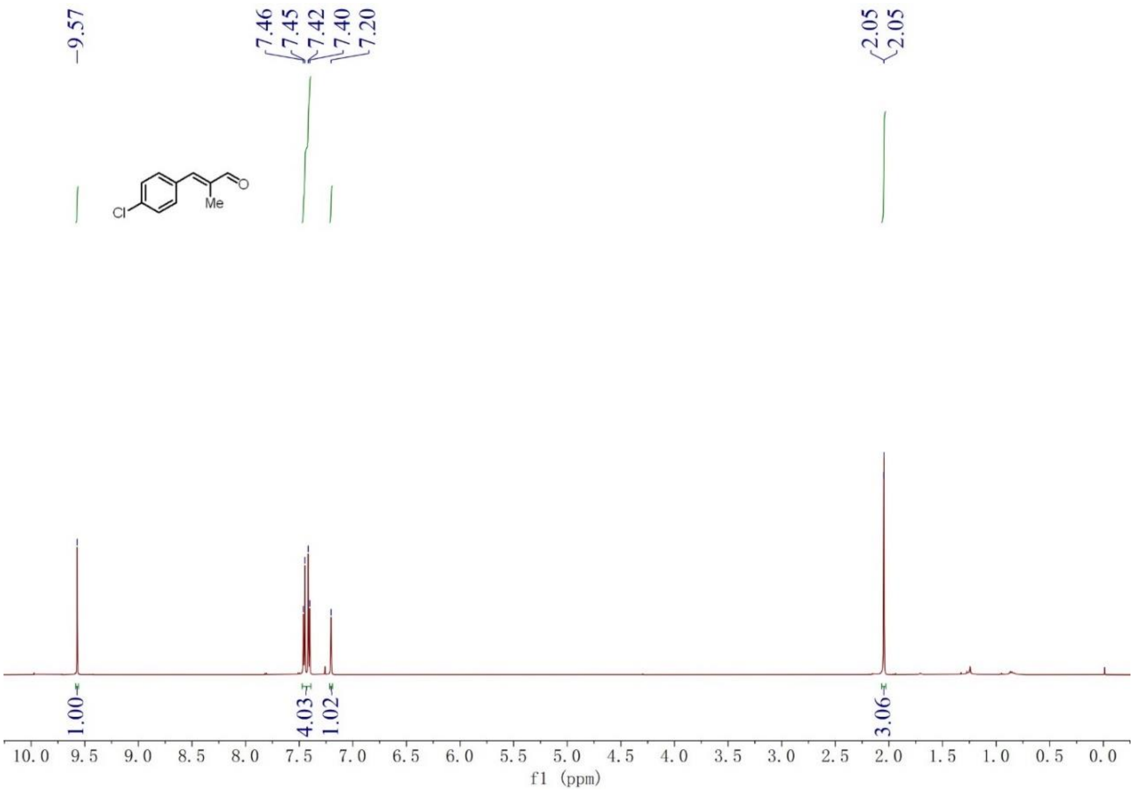


$^{1}H$ NMR spectrum of compound **8c** (600MHz, $CDCl_3$)

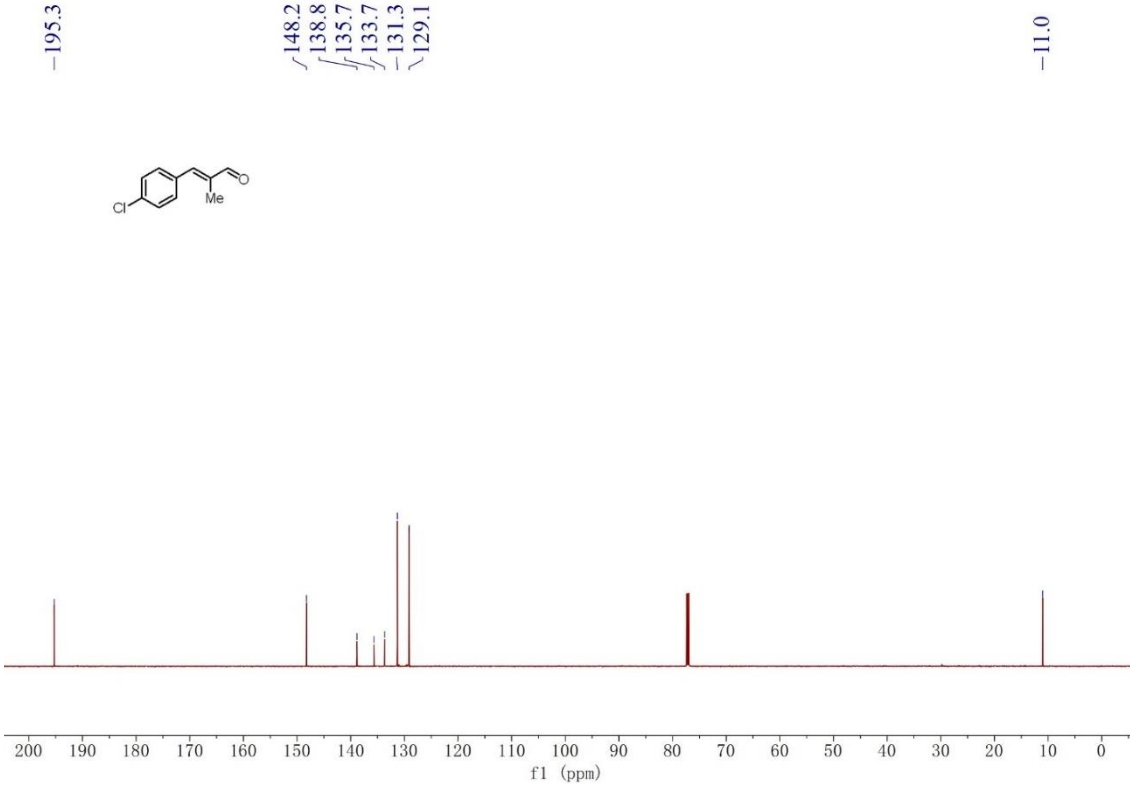


$^{13}C$ NMR spectrum of compound **8c** (151MHz, $CDCl_3$)

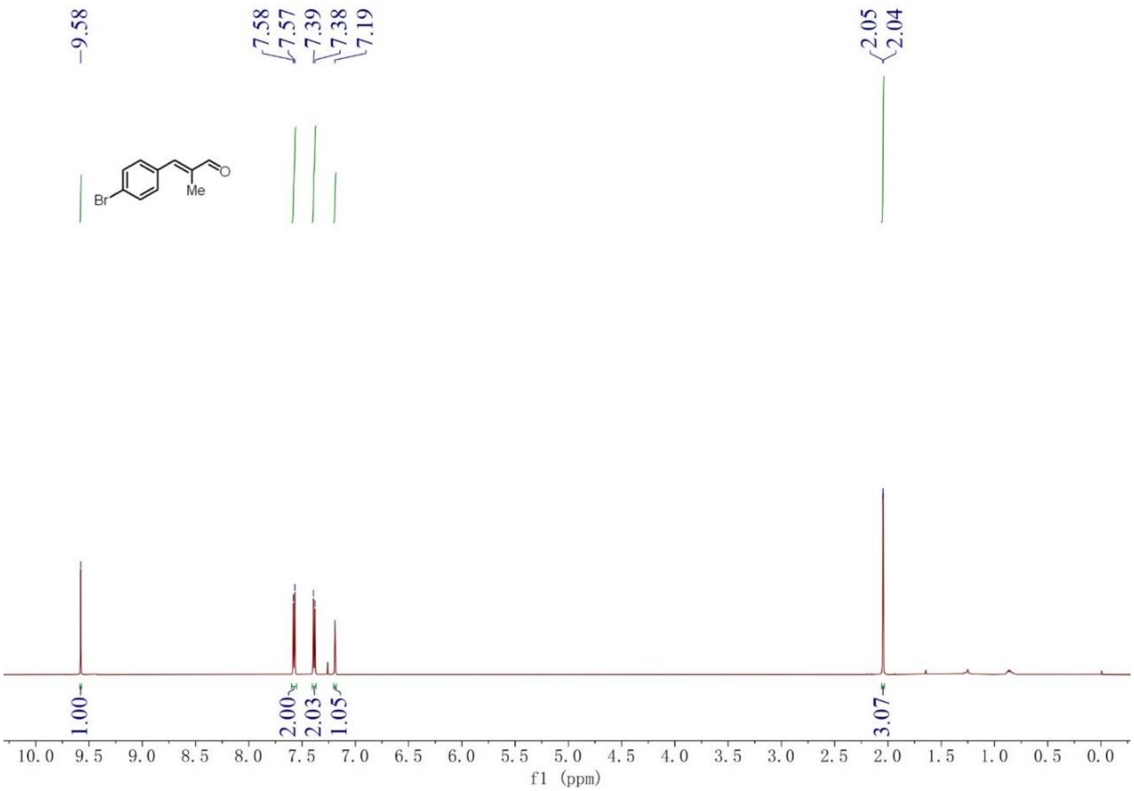

$^{1}$H NMR spectrum of compound **8d** (600MHz, $CDCl_3$)

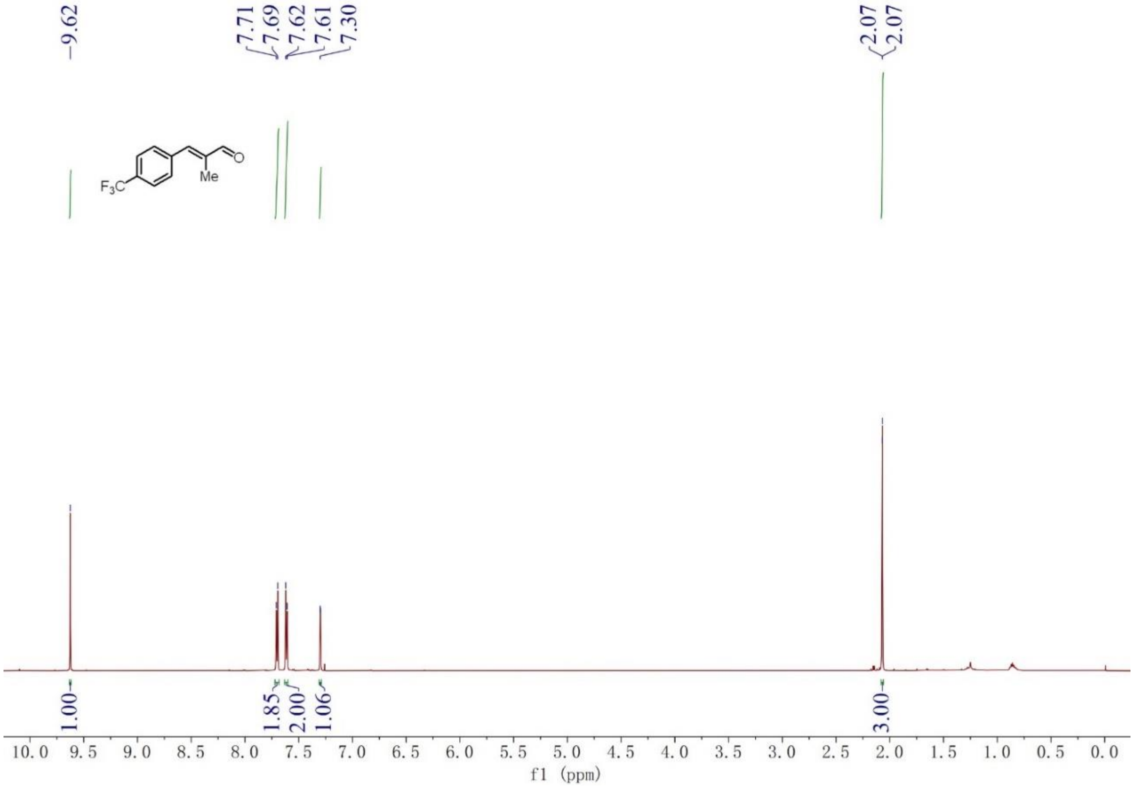

$^{1}$H NMR spectrum of compound **8e** (600MHz, $CDCl_3$)

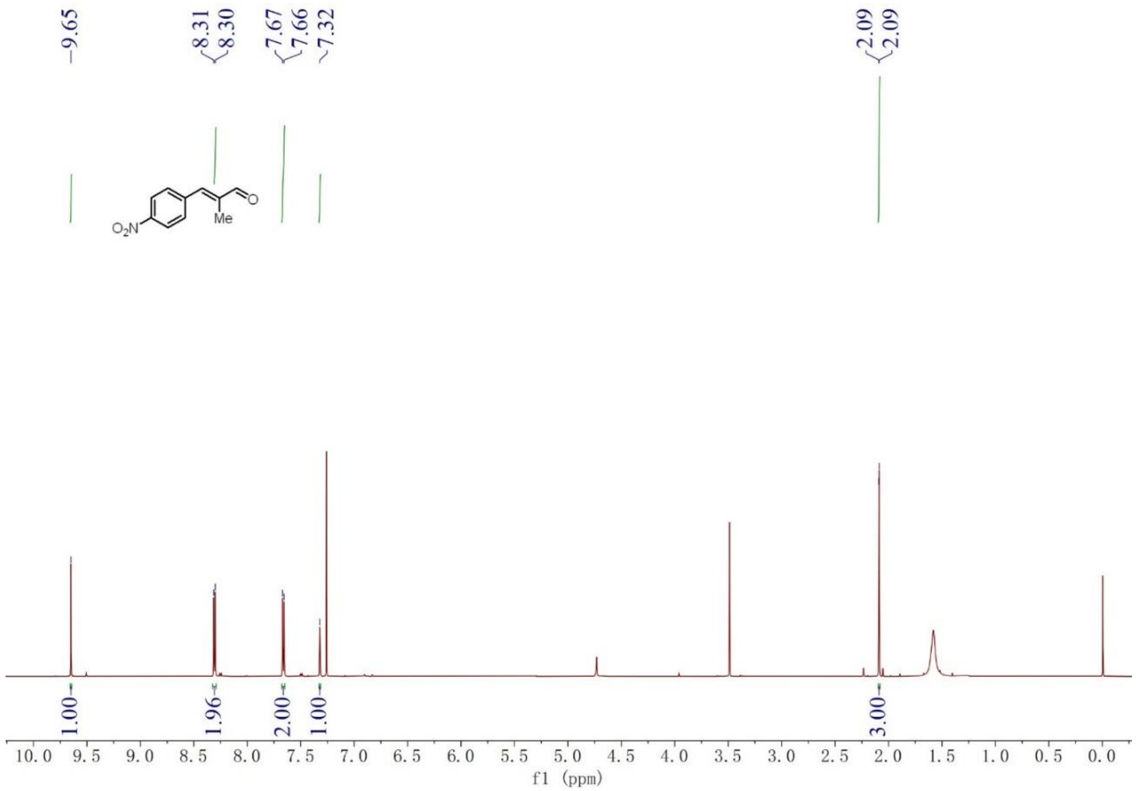


$^{1}$H NMR spectrum of compound **8f** (600MHz, $CDCl_3$)

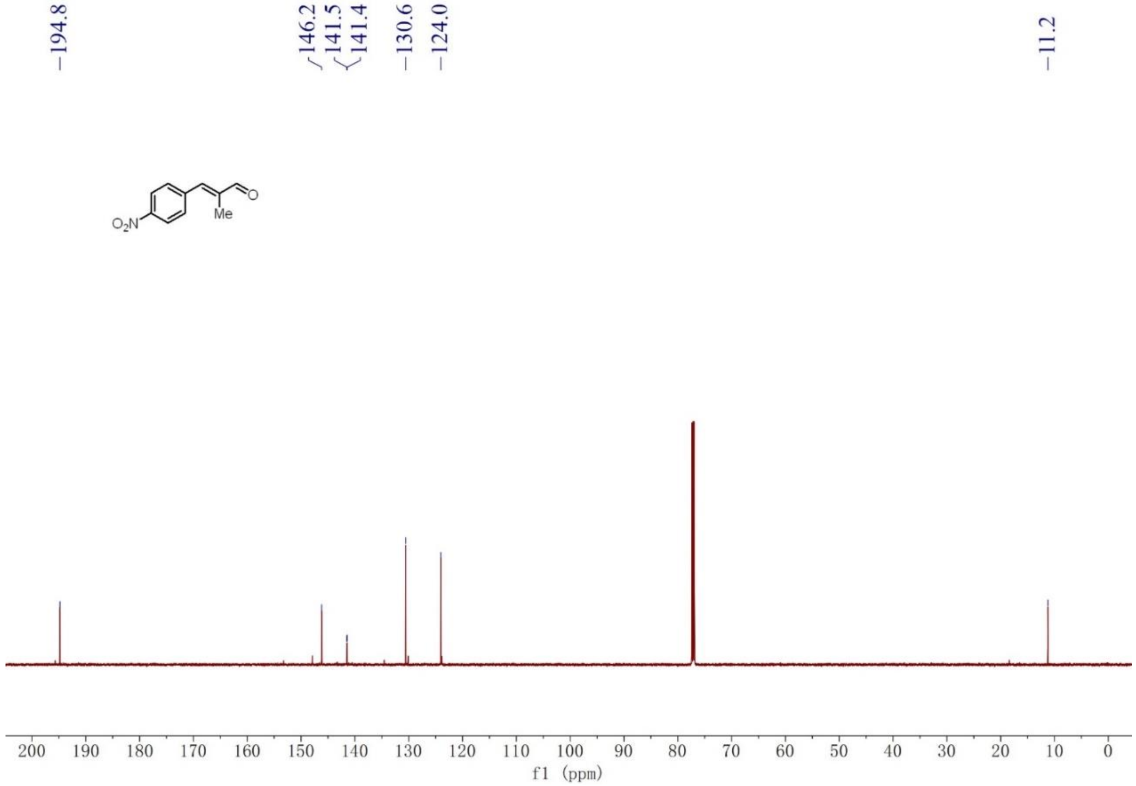


$^{13}$C NMR spectrum of compound **8f** (151MHz, $CDCl_3$)

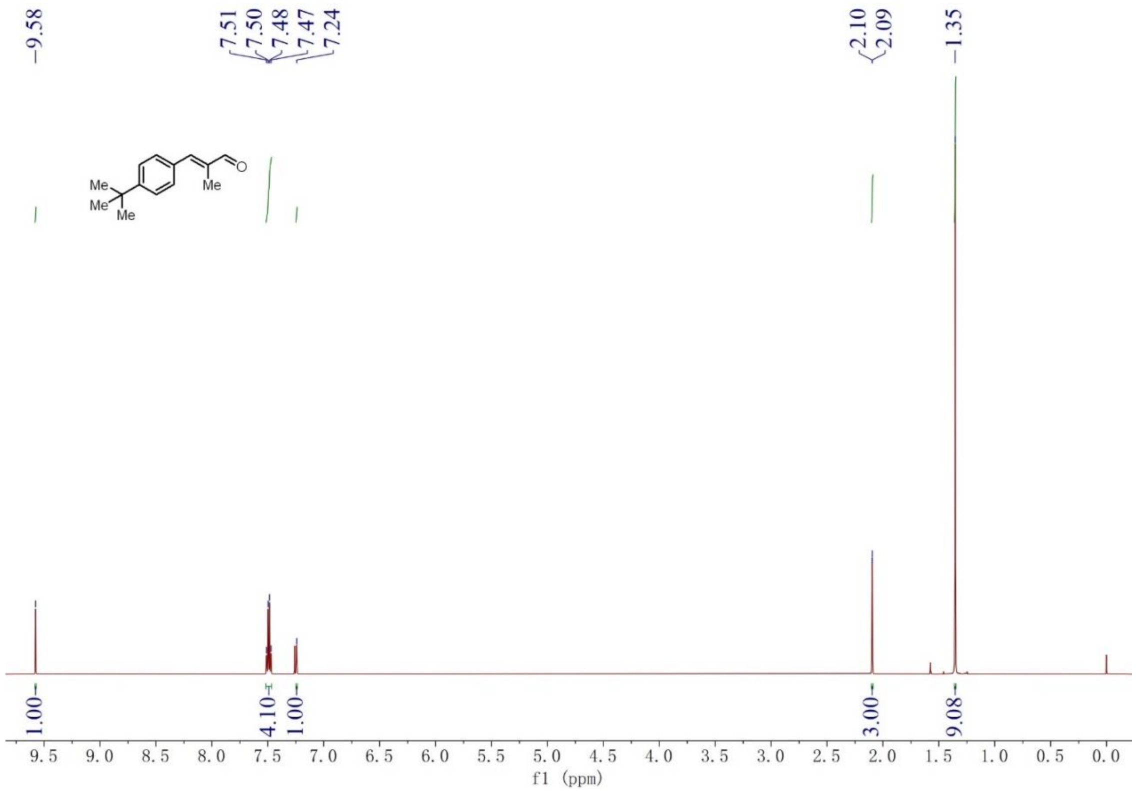


$^{1}H$ NMR spectrum of compound **8g** (600MHz, $CDCl_3$)

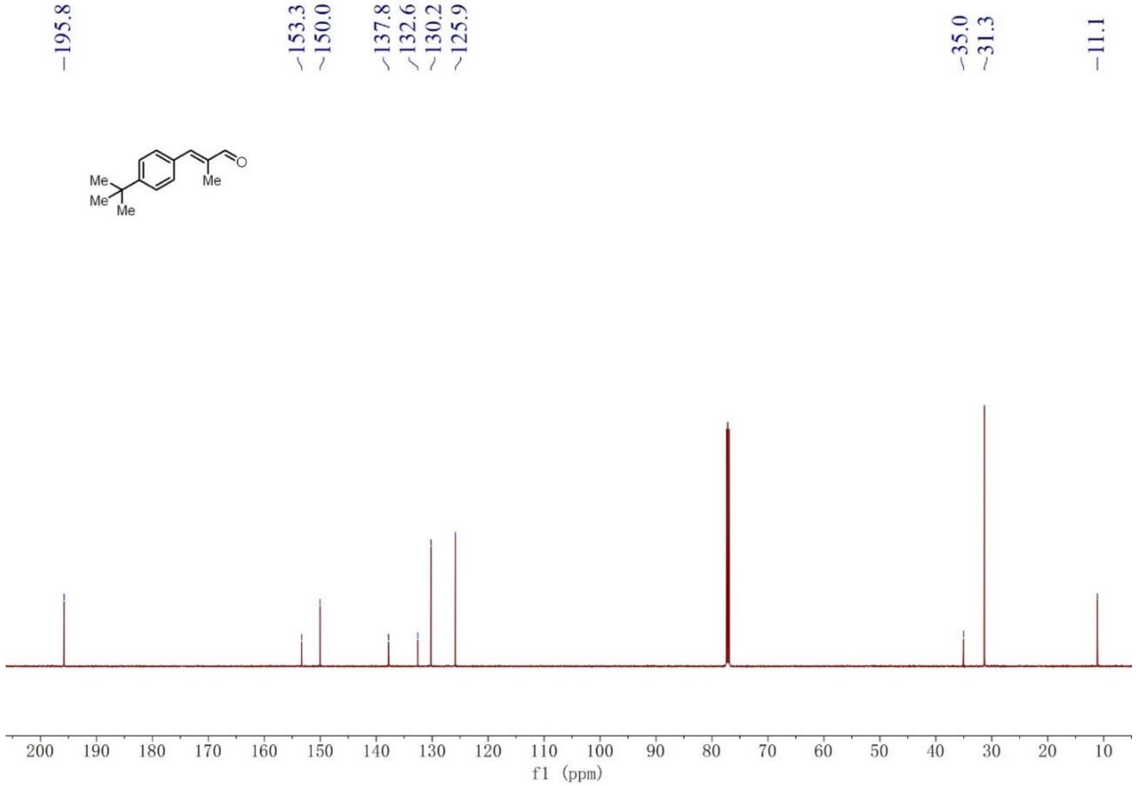


$^{13}C$ NMR spectrum of compound **8g** (151MHz, $CDCl_3$)

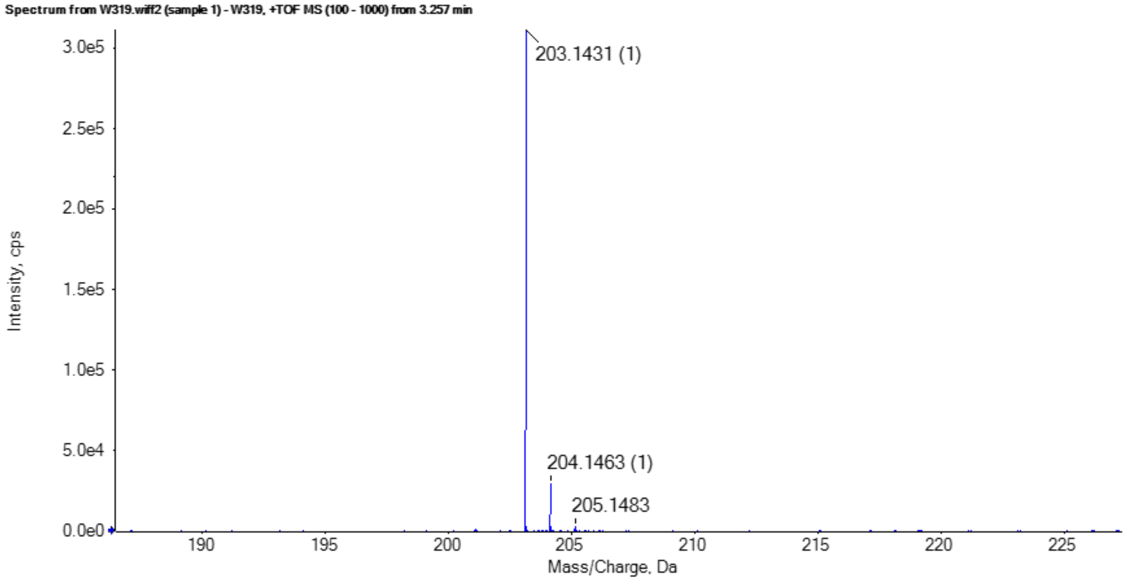


**Formula Calculator Results**

| Measured m/z | Cal m/z | Error(mmu) | Error(ppm) | Ion Formula | Ion |
|---|---|---|---|---|---|
| 203.1431 | 203.1430 | 0.1 | 0.4 | C14H19O | $[M+H]^+$ |

HRESIMS spectrum of compound **8g**

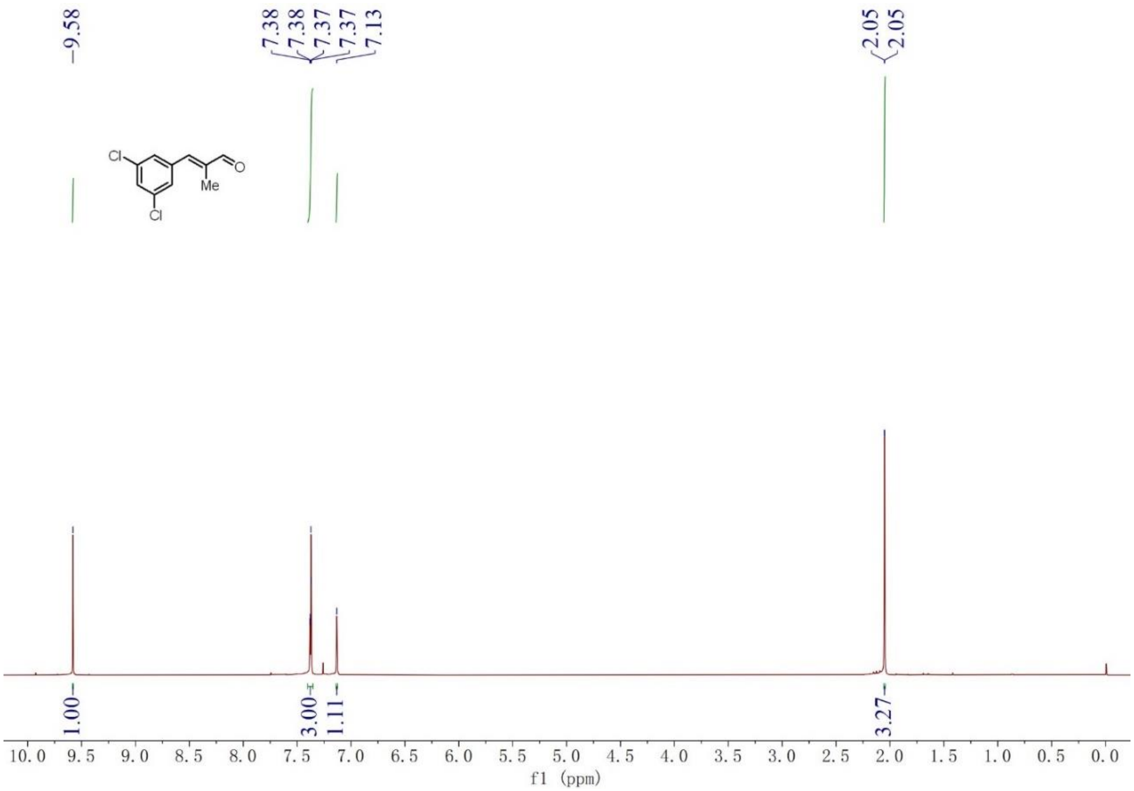


$^1$H NMR spectrum of compound **8h** (600MHz, $CDCl_3$)

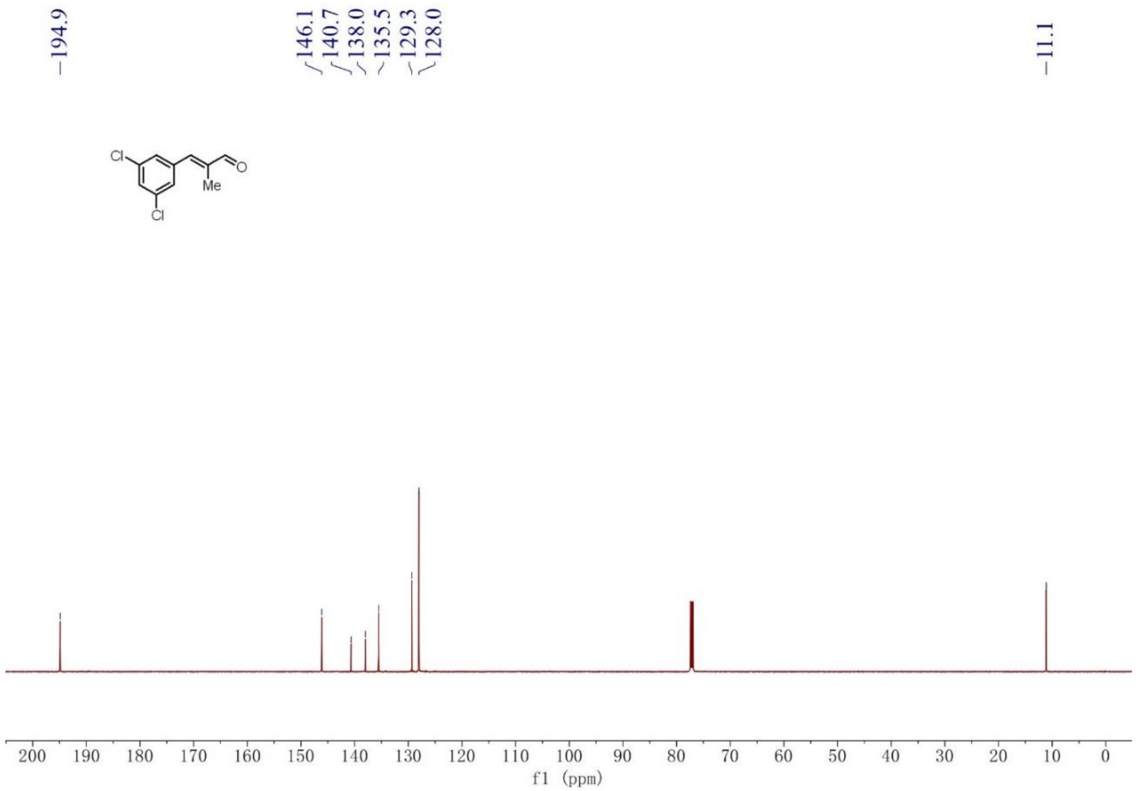


$^{13}C$ NMR spectrum of compound **8h** (151MHz, $CDCl_3$)

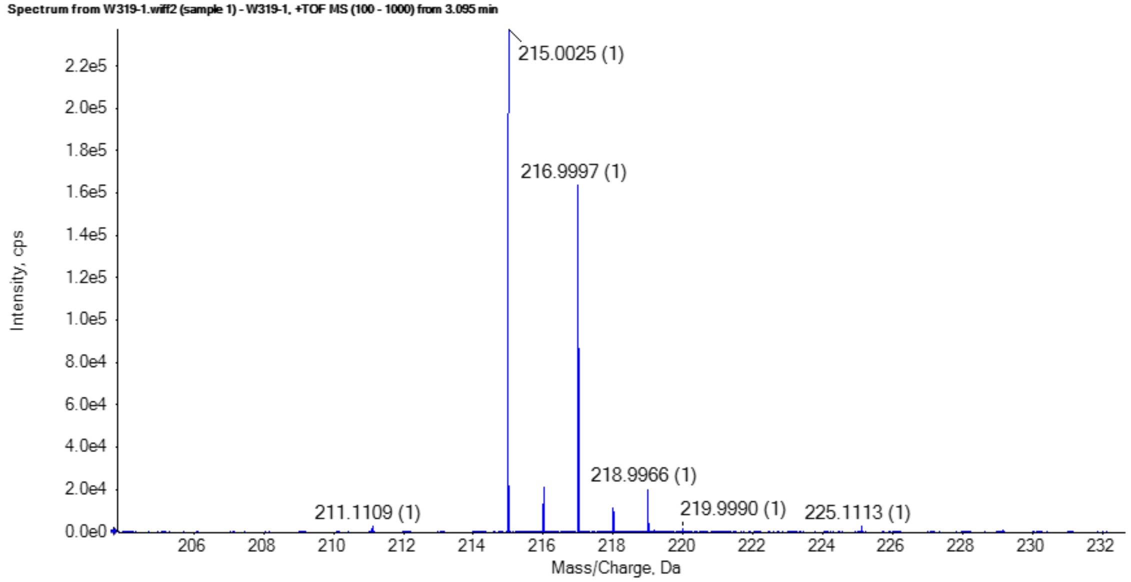


**Formula Calculator Results**

| Measured m/z | Cal m/z | Error(mmu) | Error(ppm) | Ion Formula | Ion |
|---|---|---|---|---|---|
| 215.0025 | 215.0025 | 0 | 0 | C10H9Cl2O | $[M+H]^+$ |

HRESIMS spectrum of compound **8h**

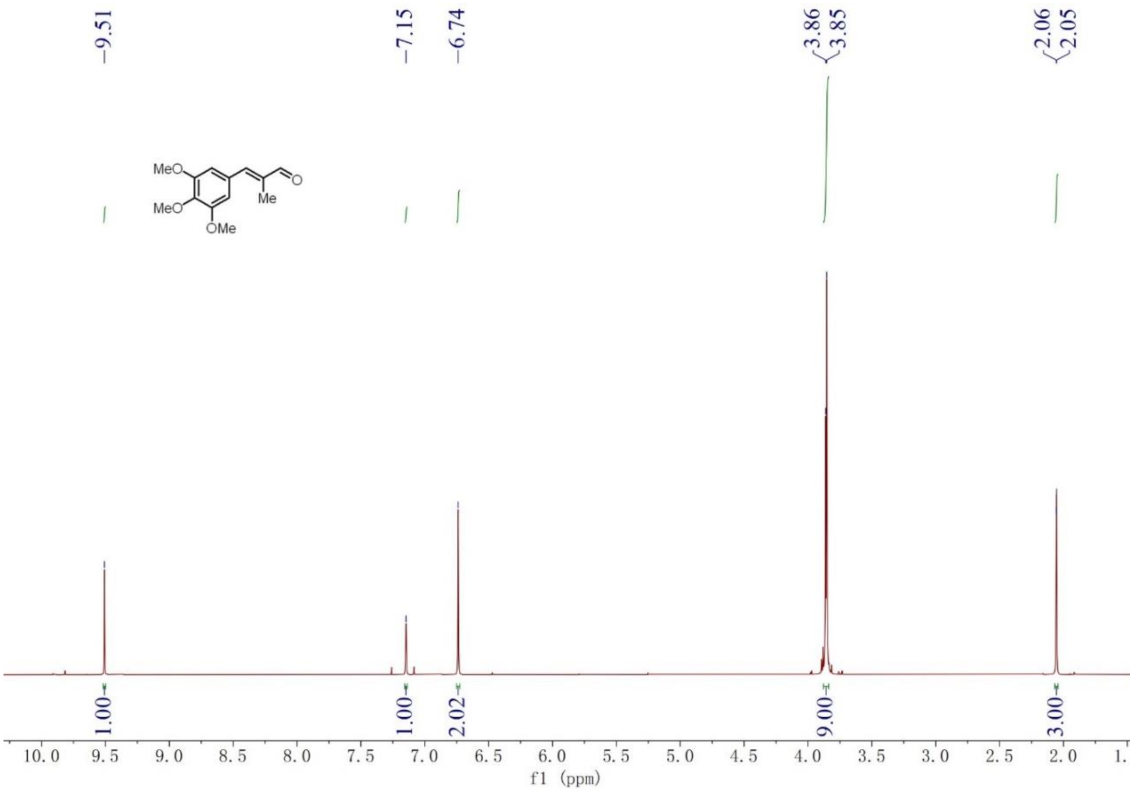


$^{1}H$ NMR spectrum of compound **8i** (600MHz, $CDCl_3$)

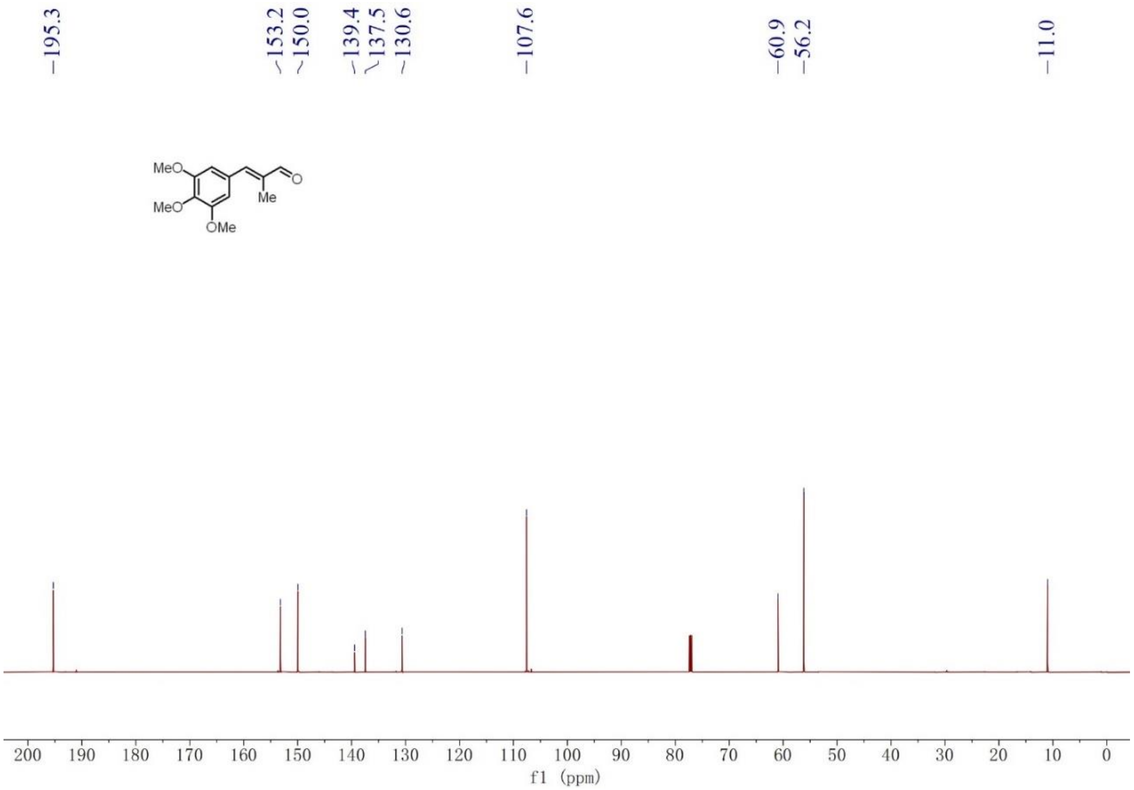


$^{13}C$ NMR spectrum of compound **8i** (151MHz, $CDCl_3$)

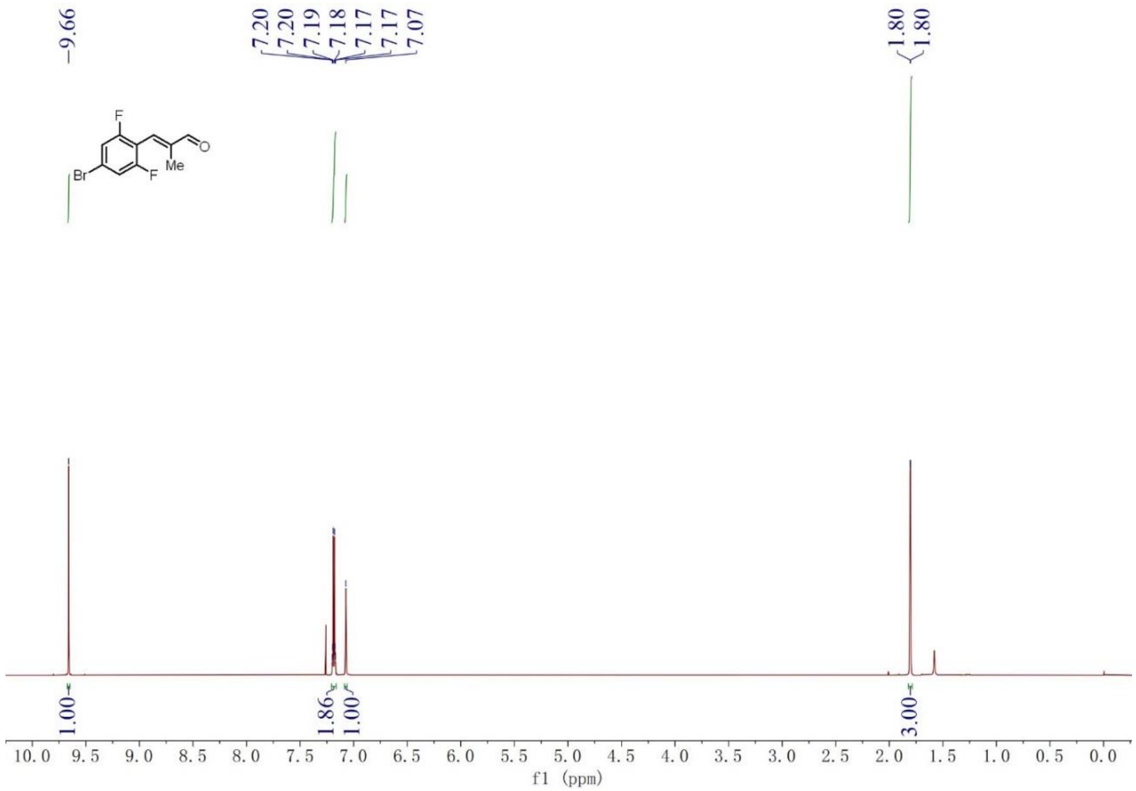


$^{1}$H NMR spectrum of compound **8j** (600MHz, $CDCl_3$)

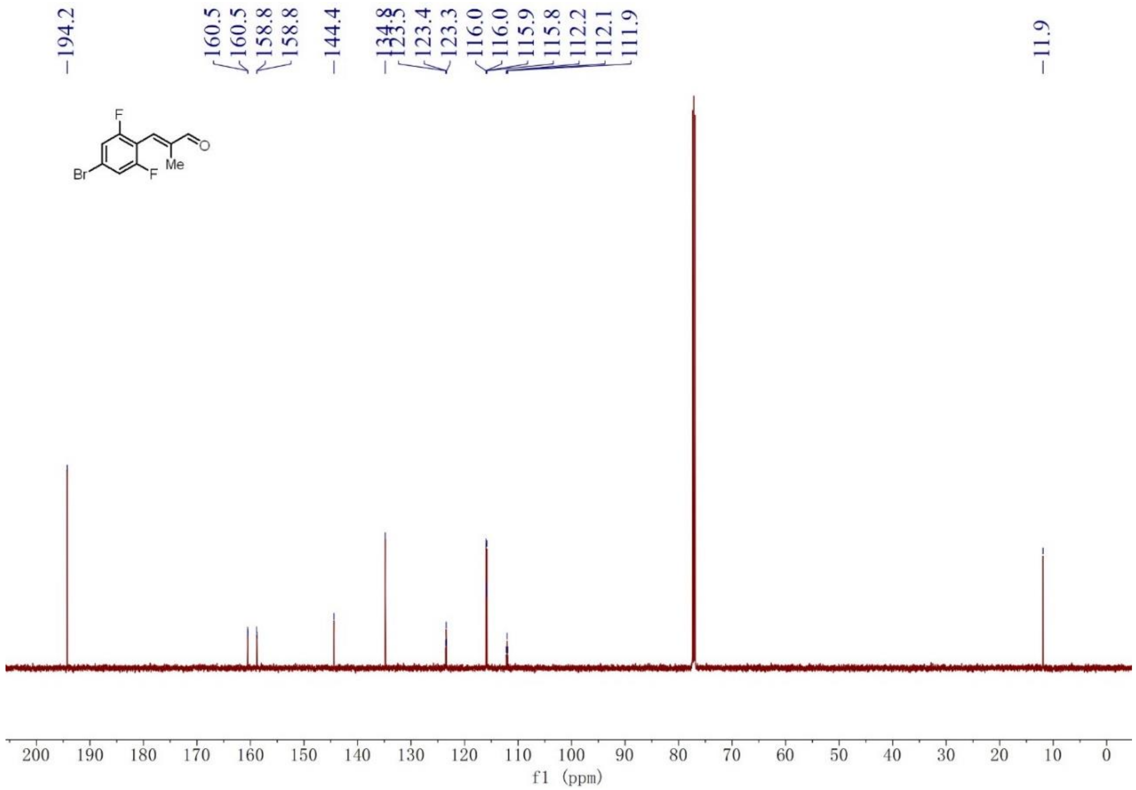


$^{13}$C NMR spectrum of compound **8j** (151MHz, $CDCl_3$)

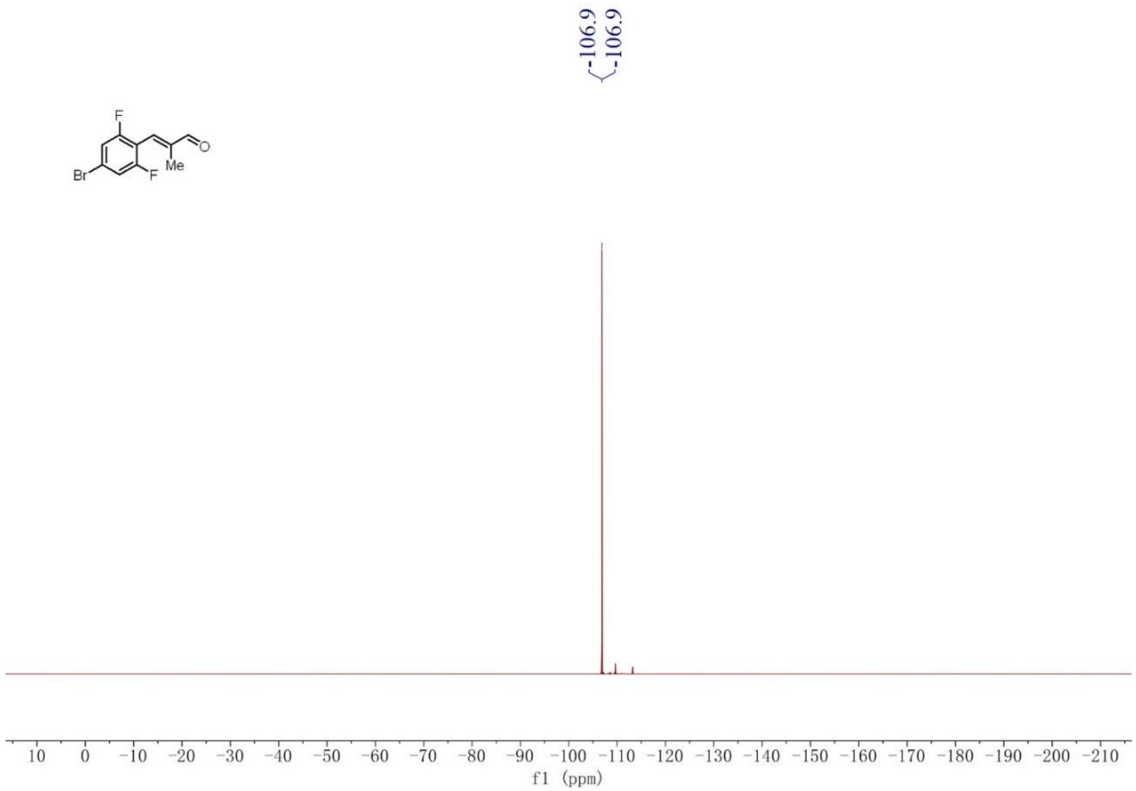


$^{19}$F NMR spectrum of compound **8j** (565MHz, $CDCl_3$)

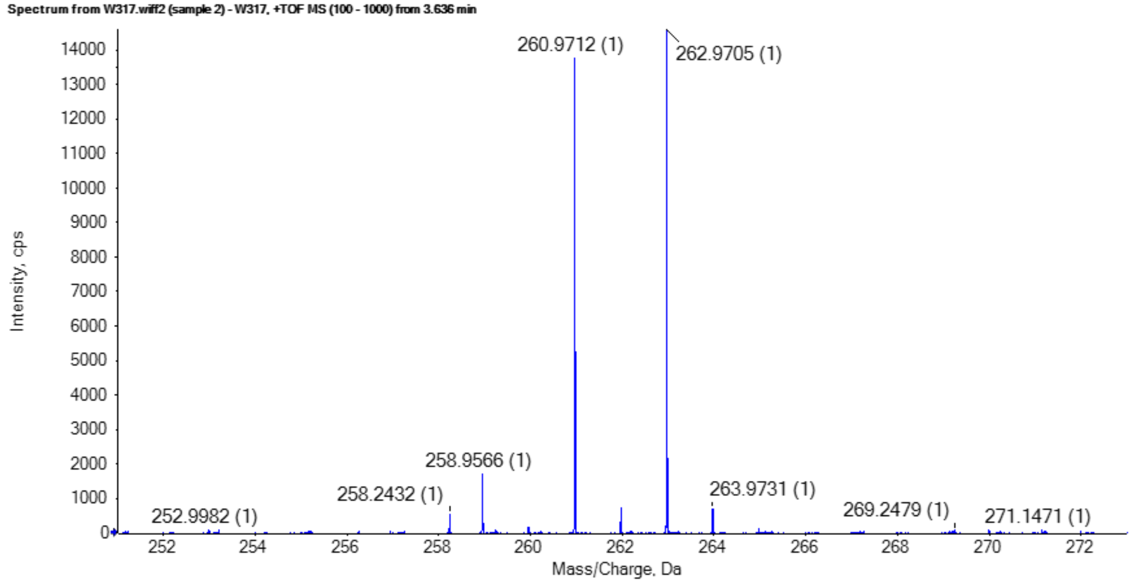


**Formula Calculator Results**

| Measured m/z | Cal m/z | Error(mmu) | Error(ppm) | Ion Formula | Ion |
|---|---|---|---|---|---|
| 260.9712 | 260.9721 | -0.9 | -3.6 | C10H8BrF2O | $[M+H]^+$ |

HRESIMS spectrum of compound **8j**

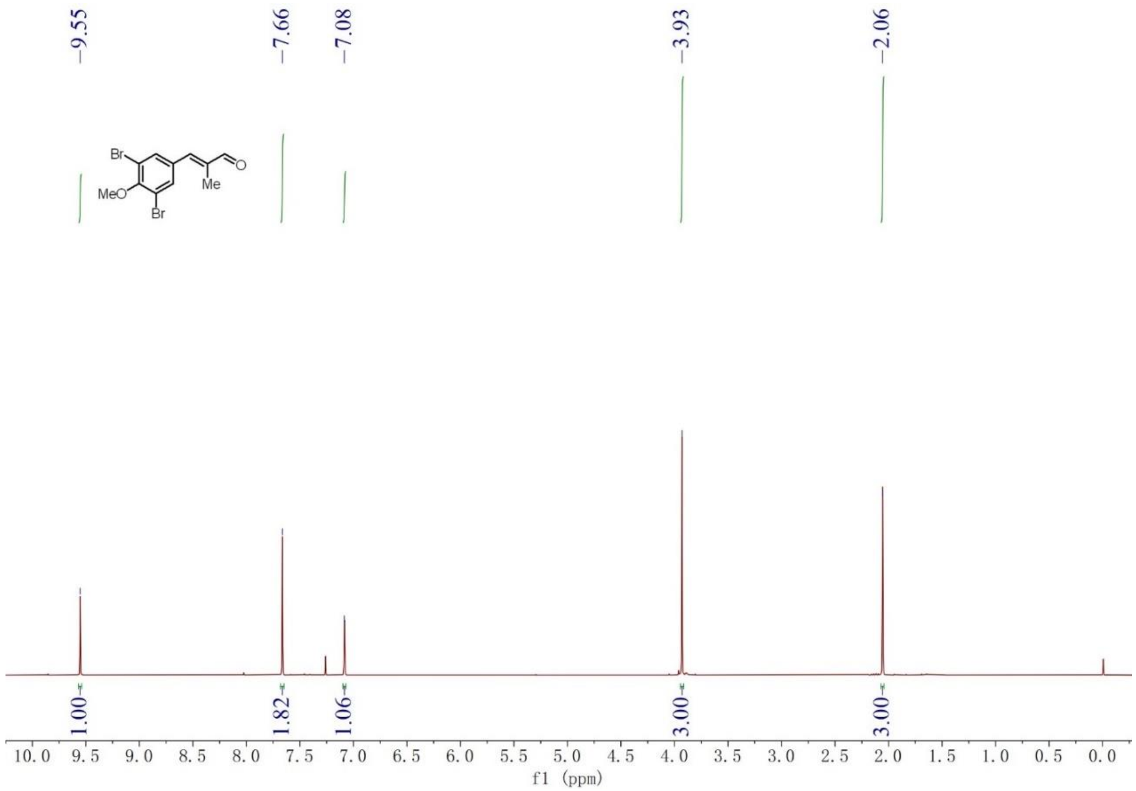


$^{1}H$ NMR spectrum of compound **8k** (600MHz, $CDCl_3$)

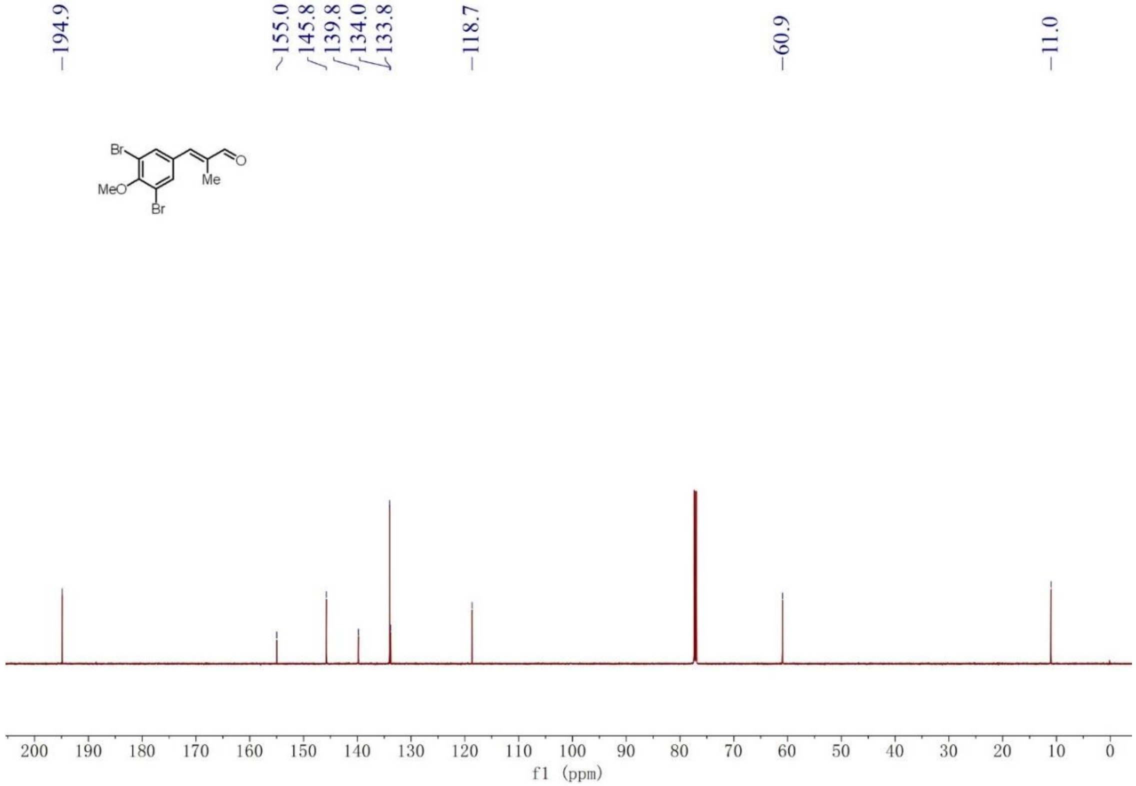


$^{13}C$ NMR spectrum of compound **8k** (151MHz, $CDCl_3$)

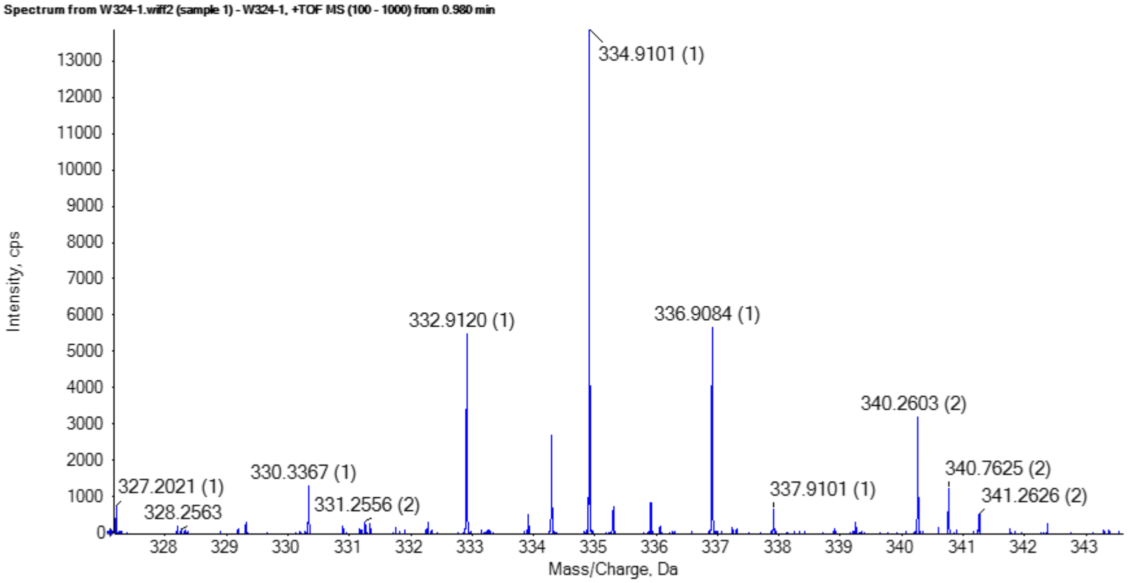


**Formula Calculator Results**

| Measured m/z | Cal m/z | Error(mmu) | Error(ppm) | Ion Formula | Ion |
|---|---|---|---|---|---|
| 332.3120 | 332.3120 | 0 | 0 | C11H11Br2O2 | $[M+H]^+$ |

HRESIMS spectrum of compound **8k**

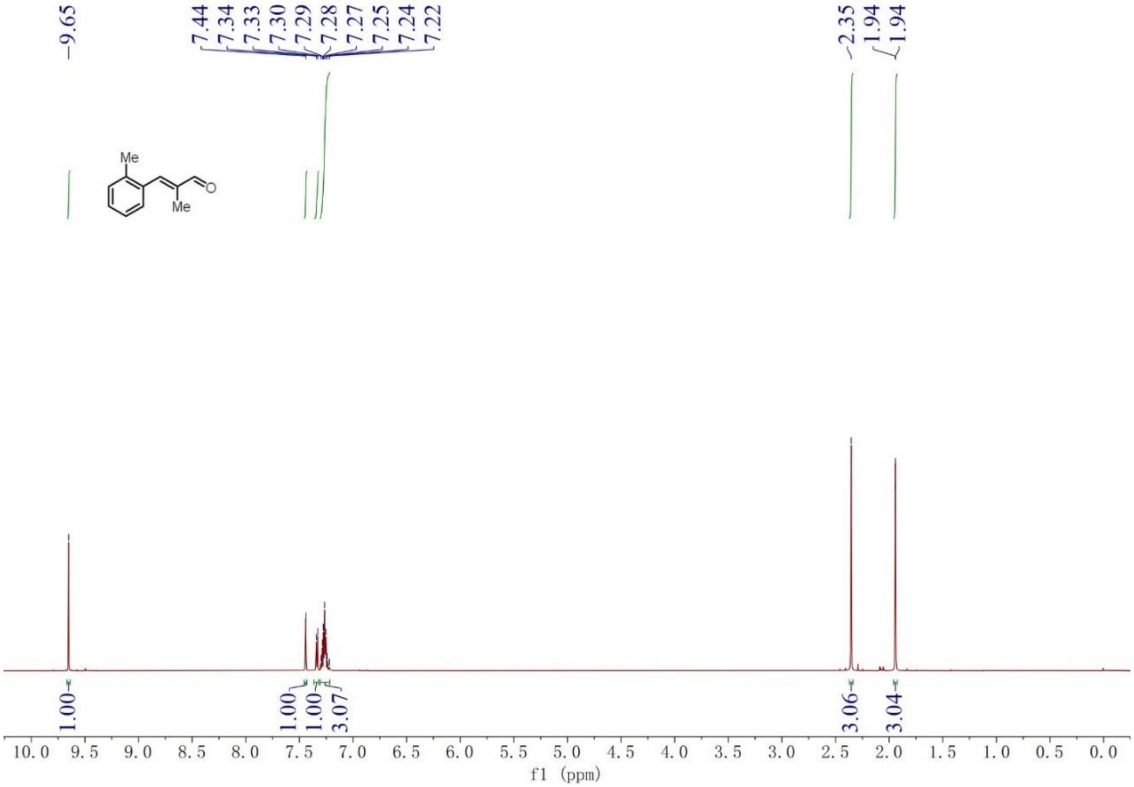


$^1$H NMR spectrum of compound **8l** (600MHz, $CDCl_3$)

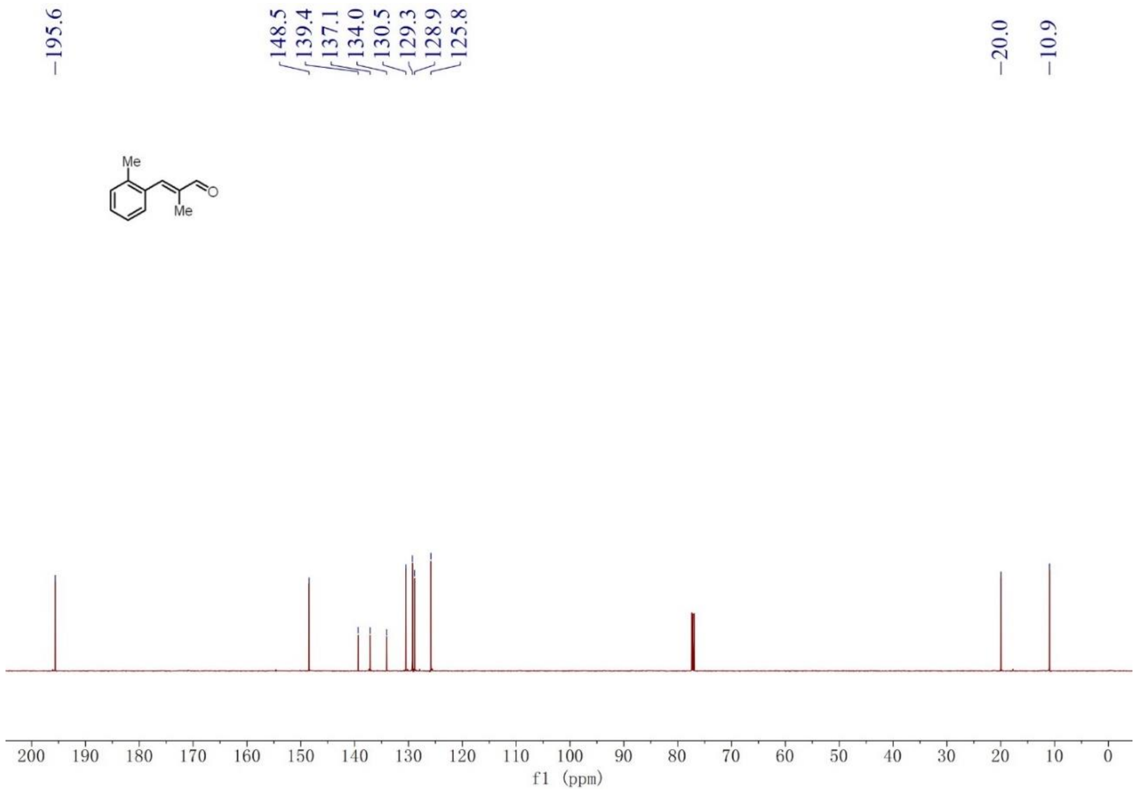


$^{13}C$ NMR spectrum of compound **8l** (151MHz, $CDCl_3$)

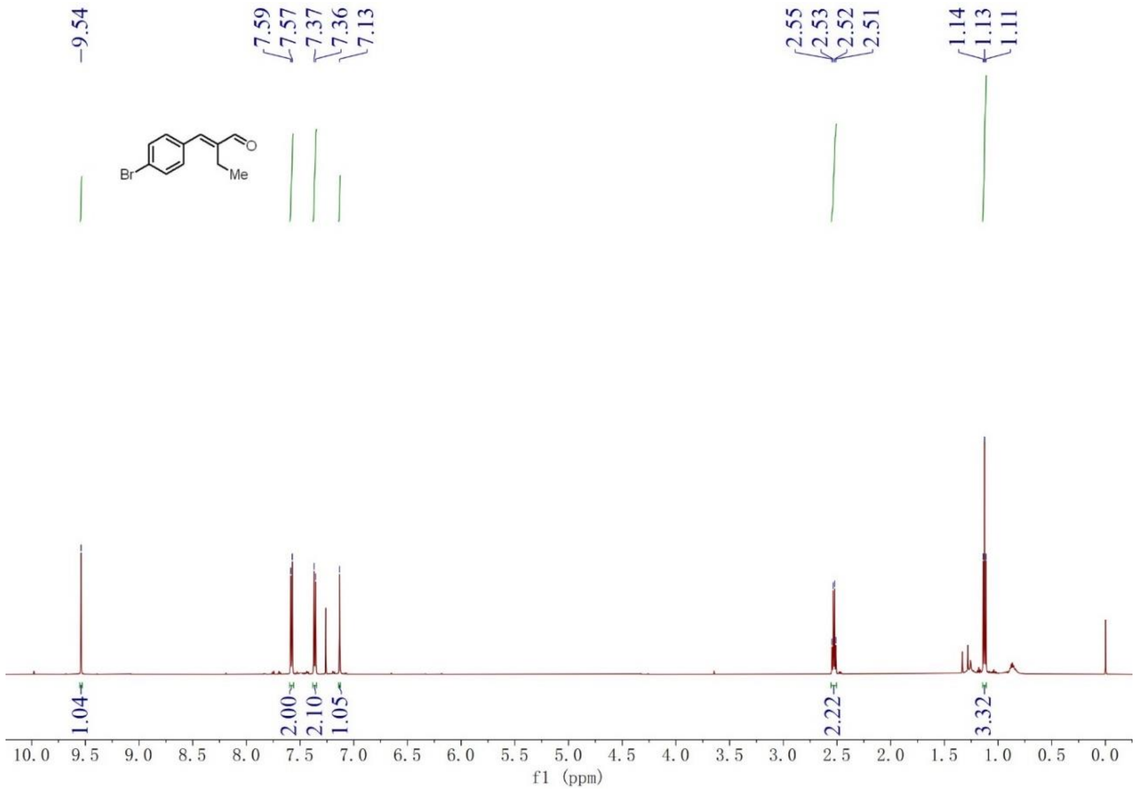


$^{1}H$ NMR spectrum of compound **8m** (600MHz, $CDCl_3$)

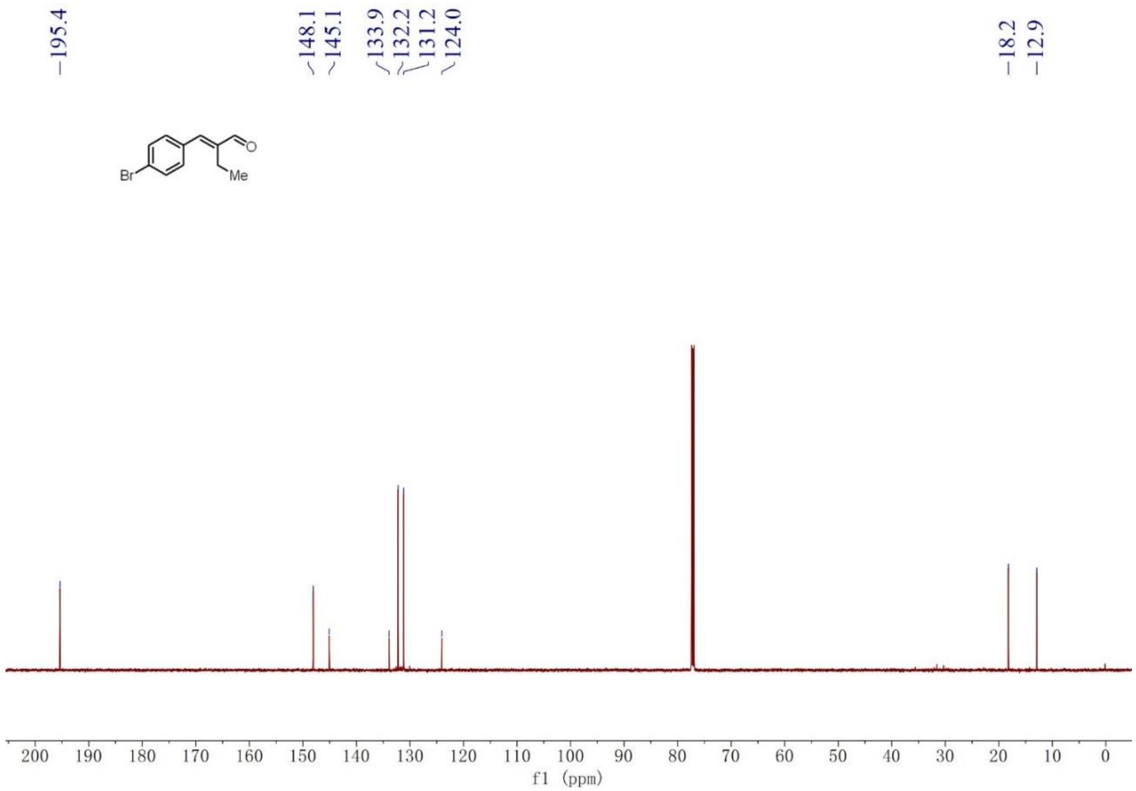

$^{13}C$ NMR spectrum of compound **8m** (151MHz, $CDCl_3$)

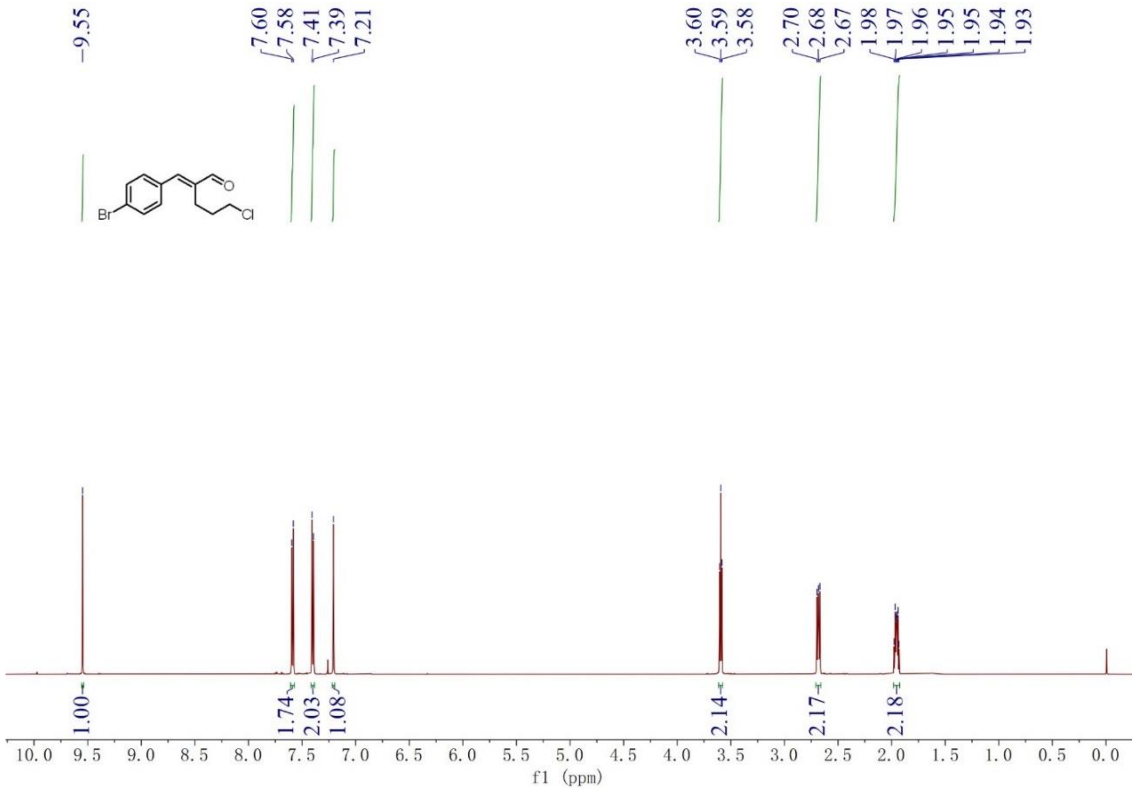

$^{1}H$ NMR spectrum of compound **8n** (600MHz, $CDCl_3$)

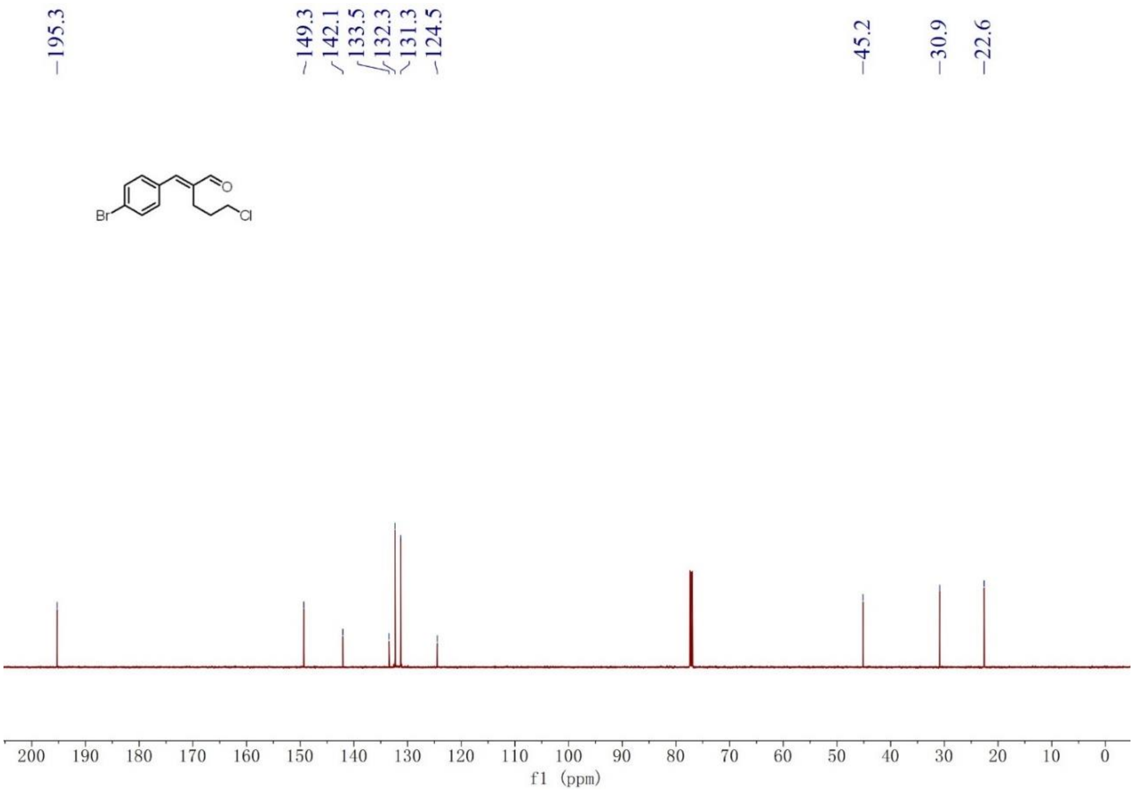


$^{13}$C NMR spectrum of compound **8n** (151MHz, $CDCl_3$)

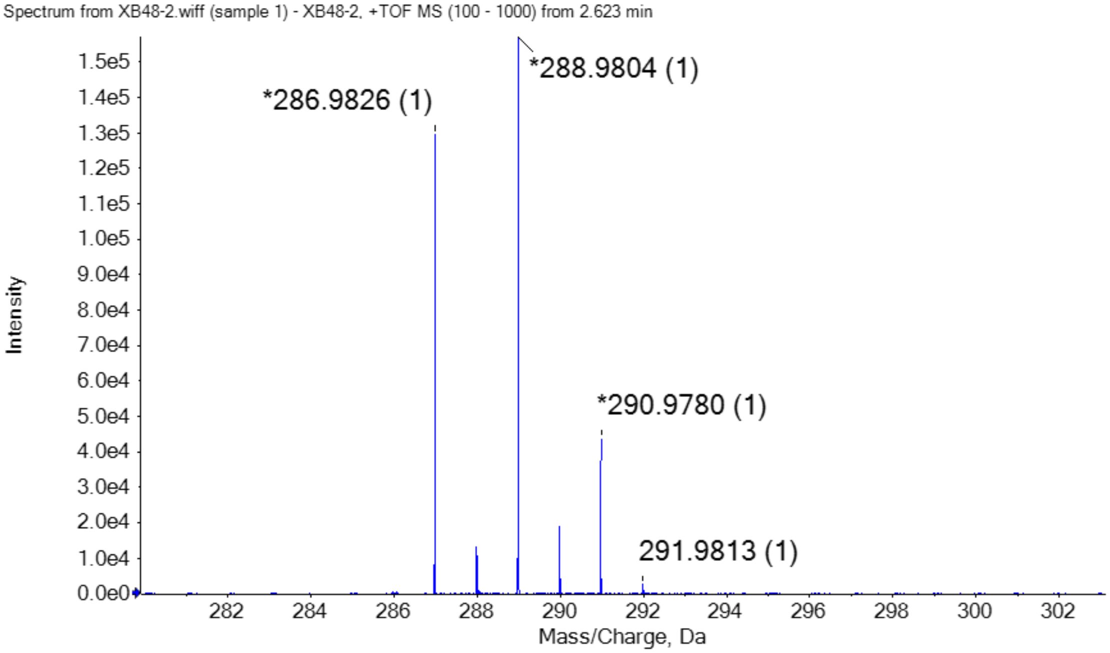


**Formula Calculator Results**

| Measured m/z | Cal m/z | Error(mmu) | Error(ppm) | Ion Formula | Ion |
|---|---|---|---|---|---|
| 286.9826 | 286.9832 | -0.7 | -2.4 | C12H13BrClO | $[M+H]^+$ |

HRESIMS spectrum of compound **8n**

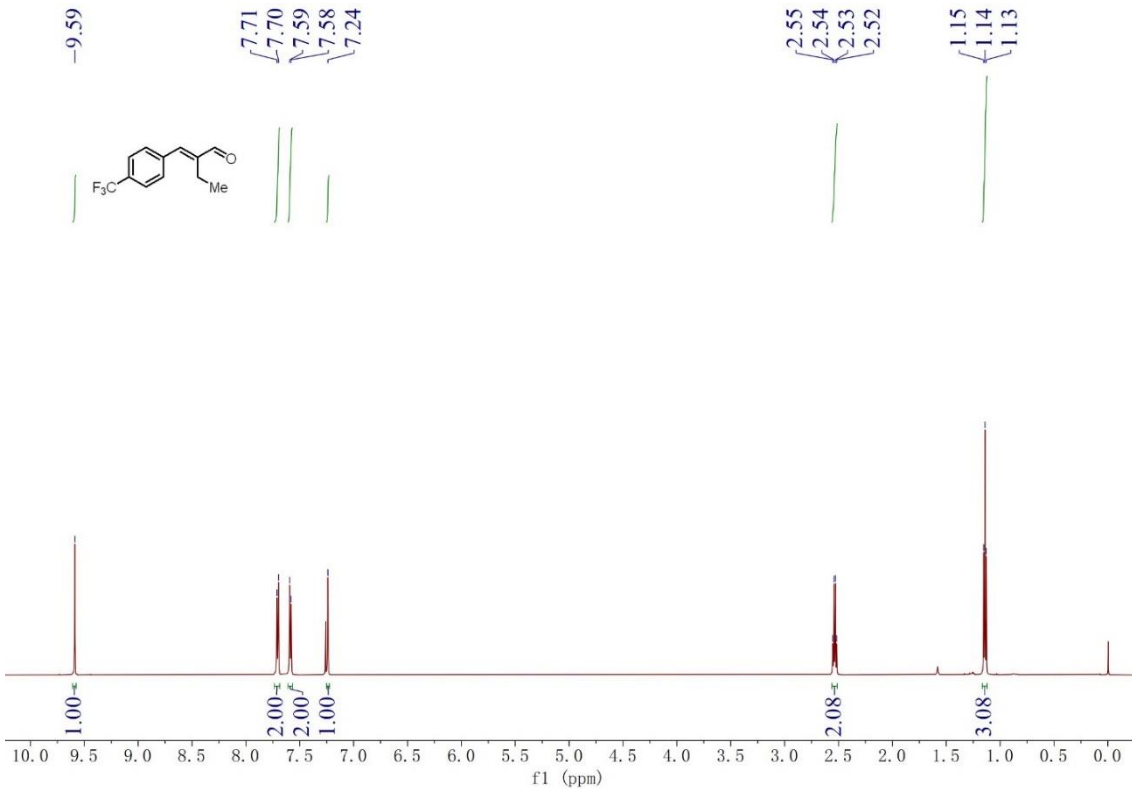


$^{1}$H NMR spectrum of compound **8o** (600MHz, $CDCl_3$)

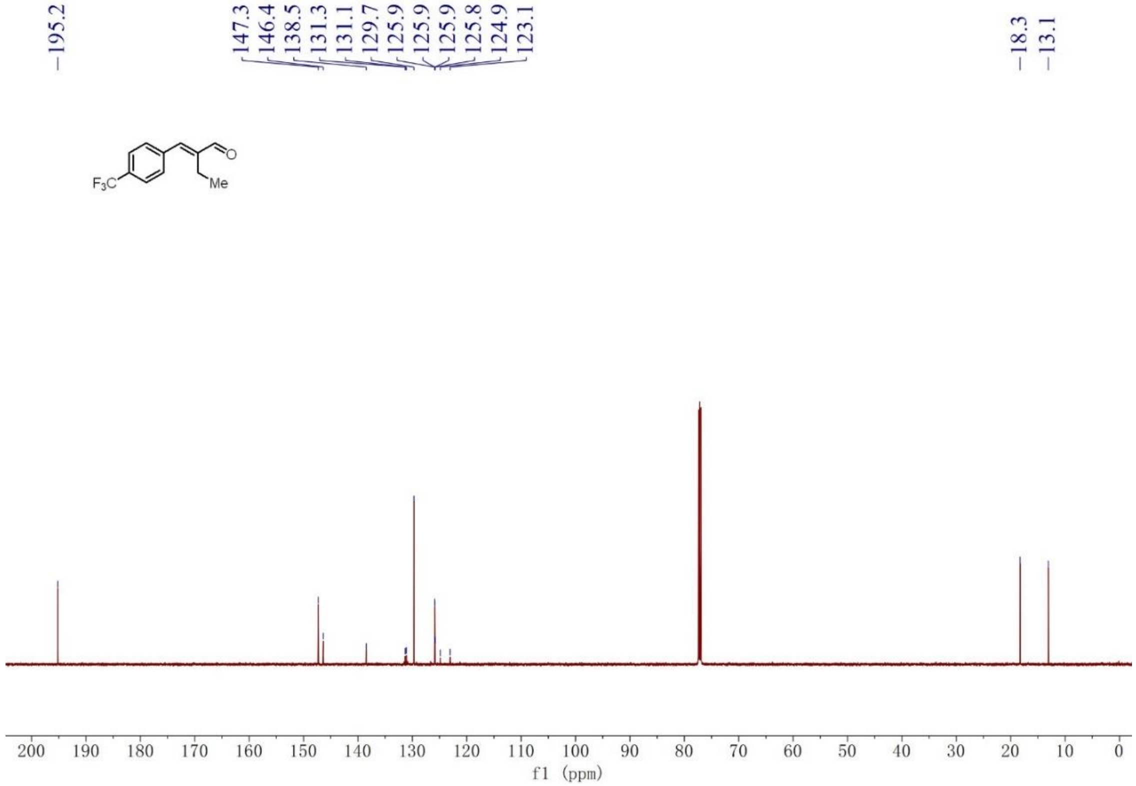


$^{13}$C NMR spectrum of compound **8o** (151MHz, $CDCl_3$)

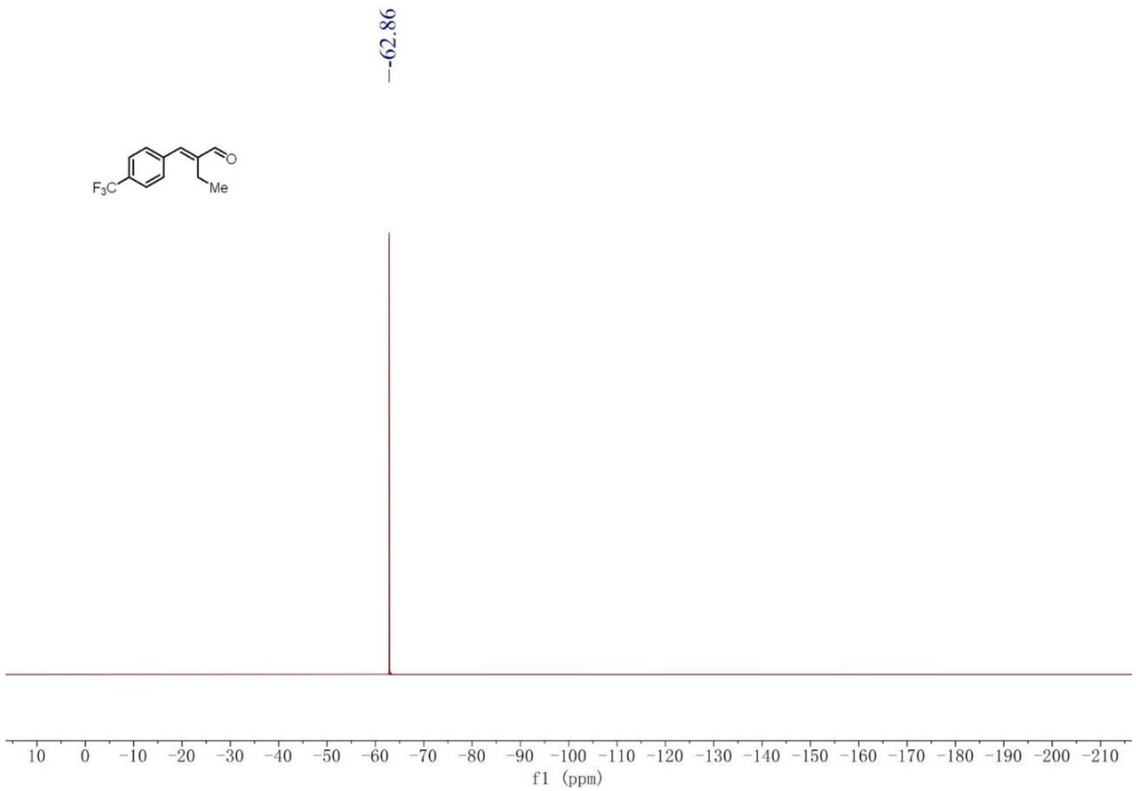


$^{19}$F NMR spectrum of compound **8o** (565MHz, $CDCl_3$)

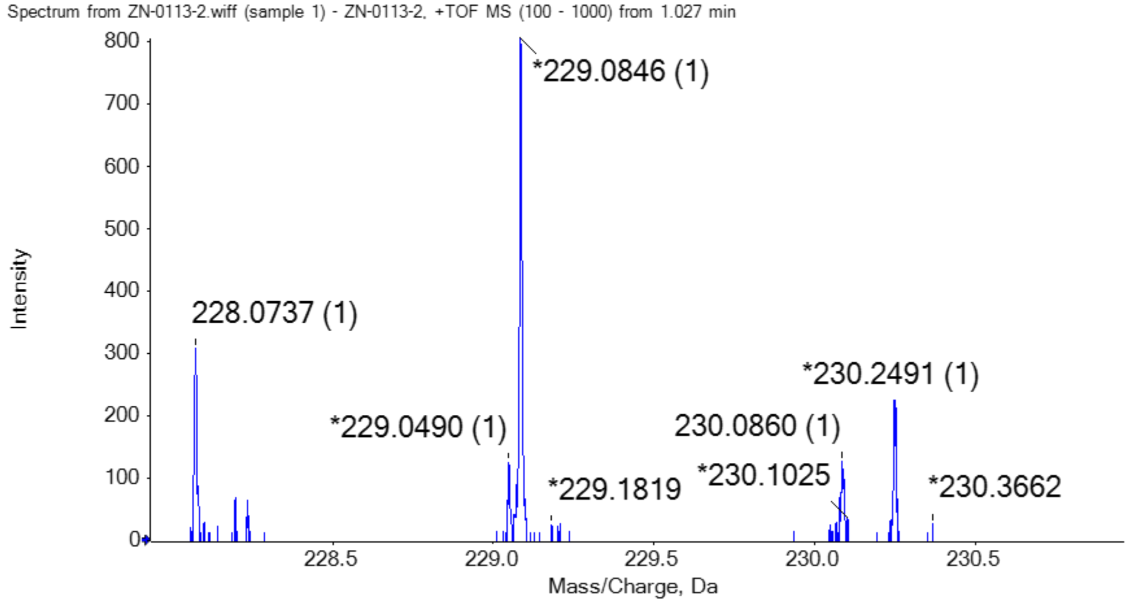


**Formula Calculator Results**

| Measured m/z | Cal m/z | Error(mmu) | Error(ppm) | Ion Formula | Ion |
|---|---|---|---|---|---|
| 229.0846 | 229.0834 | 1.1 | 4.9 | C12H12F3O | $[M+H]^+$ |

HRESIMS spectrum of compound **8o**

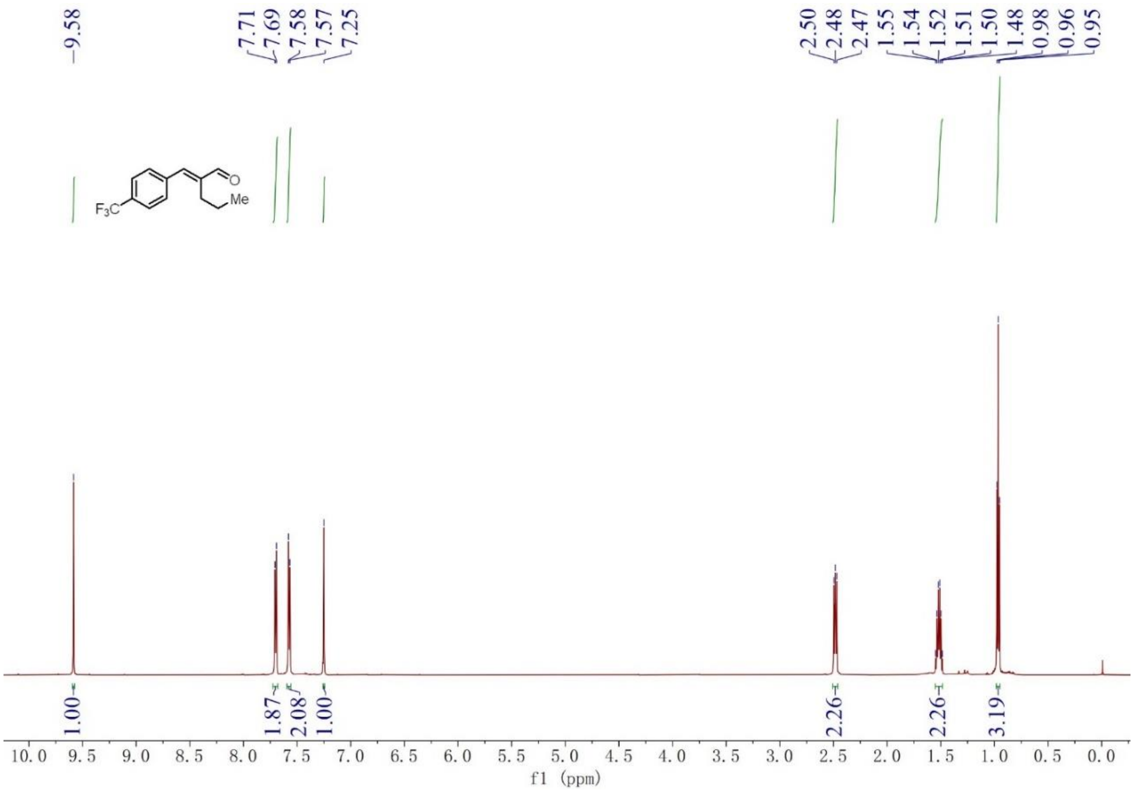


$^{1}$H NMR spectrum of compound **8p** (600MHz, $CDCl_3$)

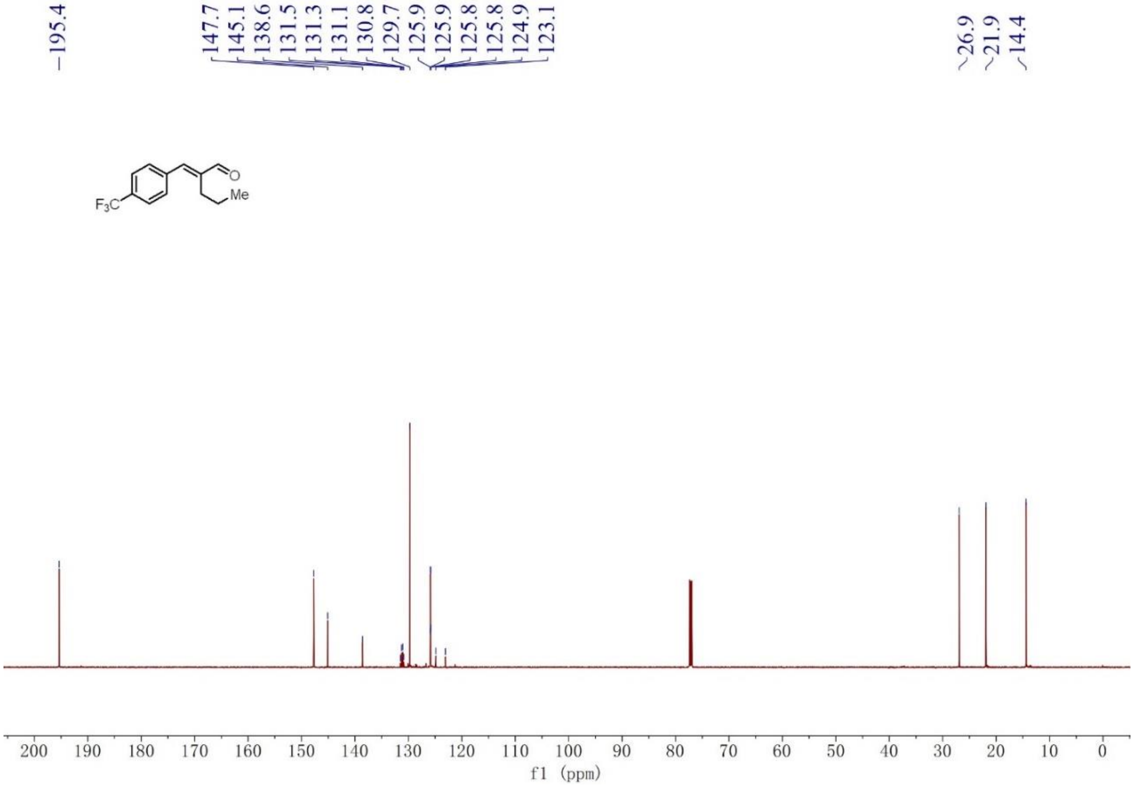


$^{13}$C NMR spectrum of compound **8p** (151MHz, $CDCl_3$)

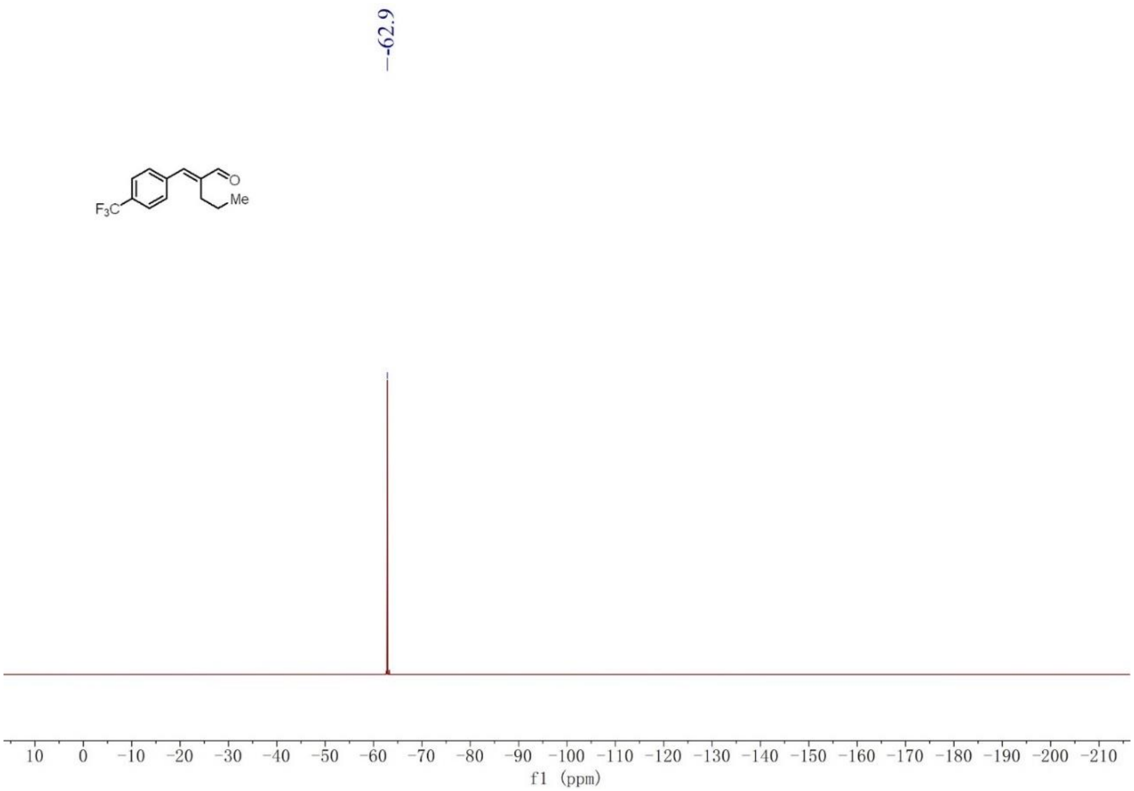


$^{19}$F NMR spectrum of compound **8p** (565MHz, $CDCl_3$)

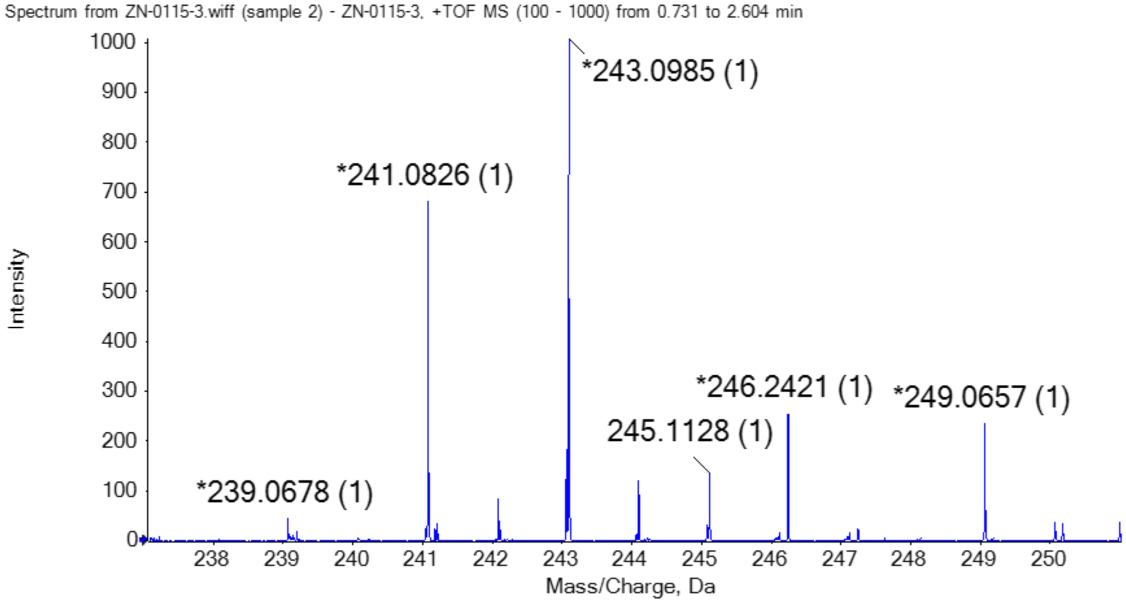


**Formula Calculator Results**

| Measured m/z | Cal m/z | Error(mmu) | Error(ppm) | Ion Formula | Ion |
|---|---|---|---|---|---|
| 243.0985 | 243.0991 | -0.6 | -2.6 | C13H14F3O | $[M+H]^+$ |

HRESIMS spectrum of compound **8p**

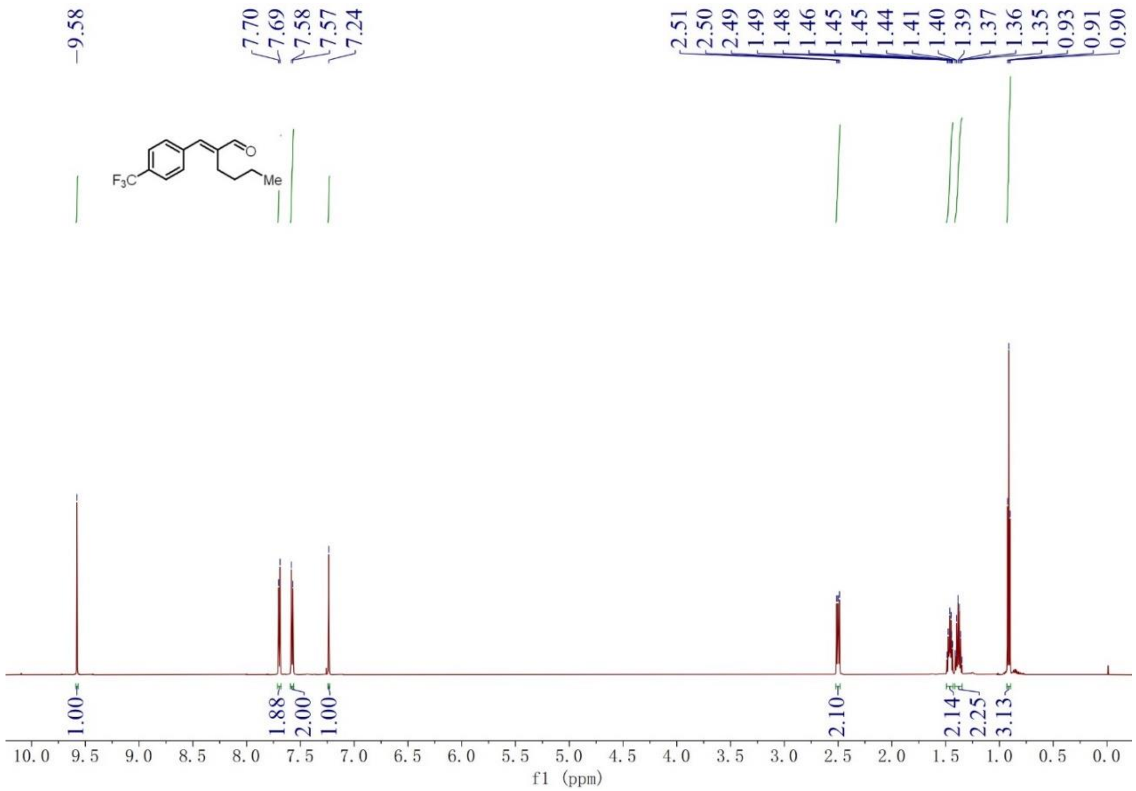


$^{1}$H NMR spectrum of compound **8q** (600MHz, $CDCl_3$)

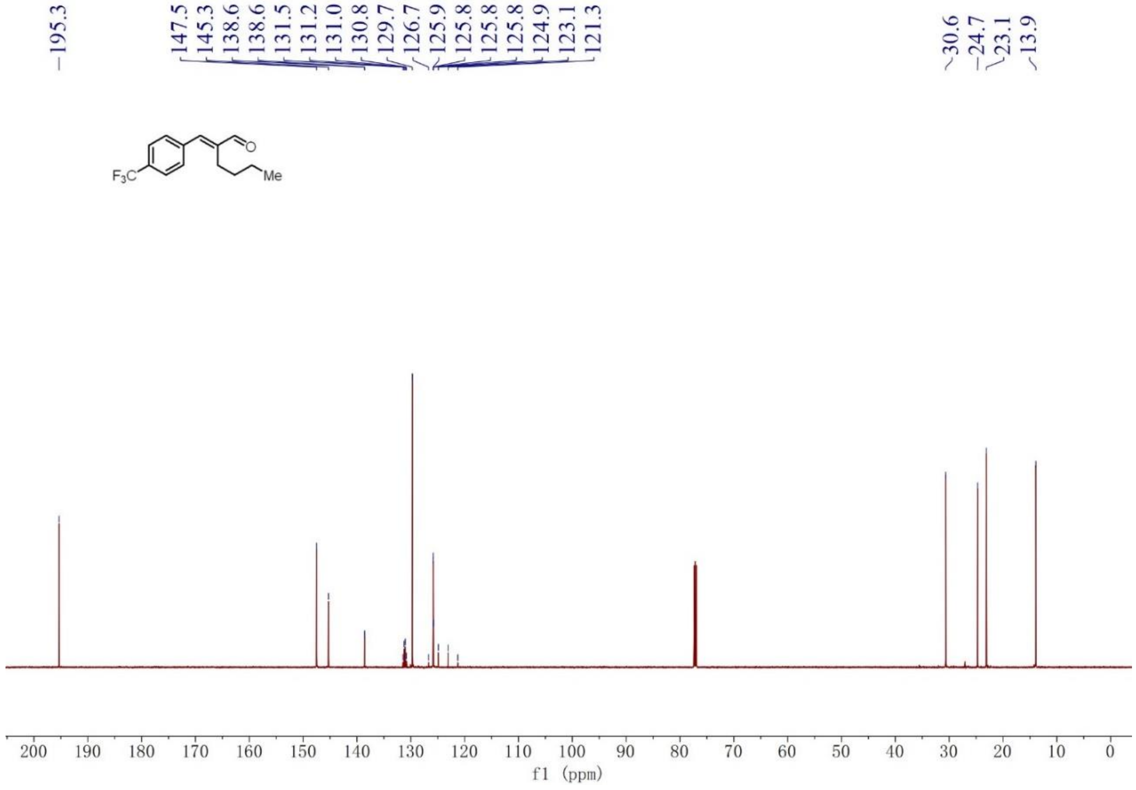


$^{13}$C NMR spectrum of compound **8q** (151MHz, $CDCl_3$)

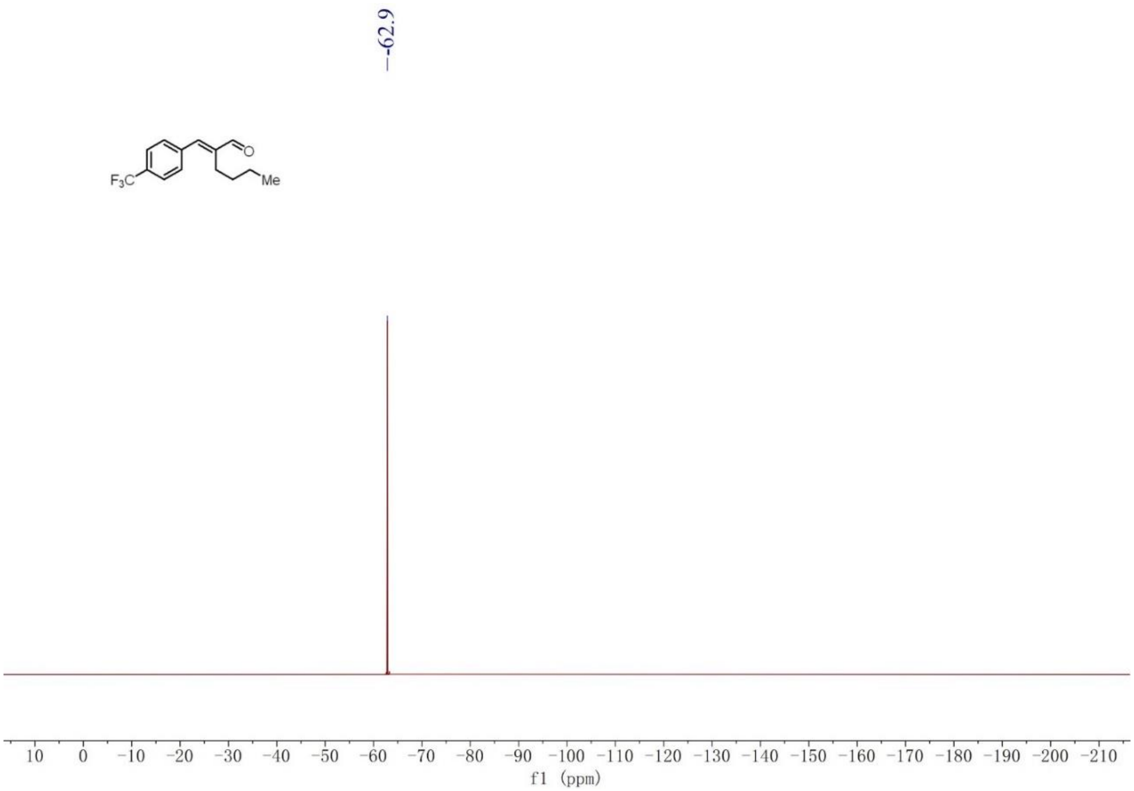


$^{19}F$ NMR spectrum of compound **8q** (565MHz, $CDCl_3$)

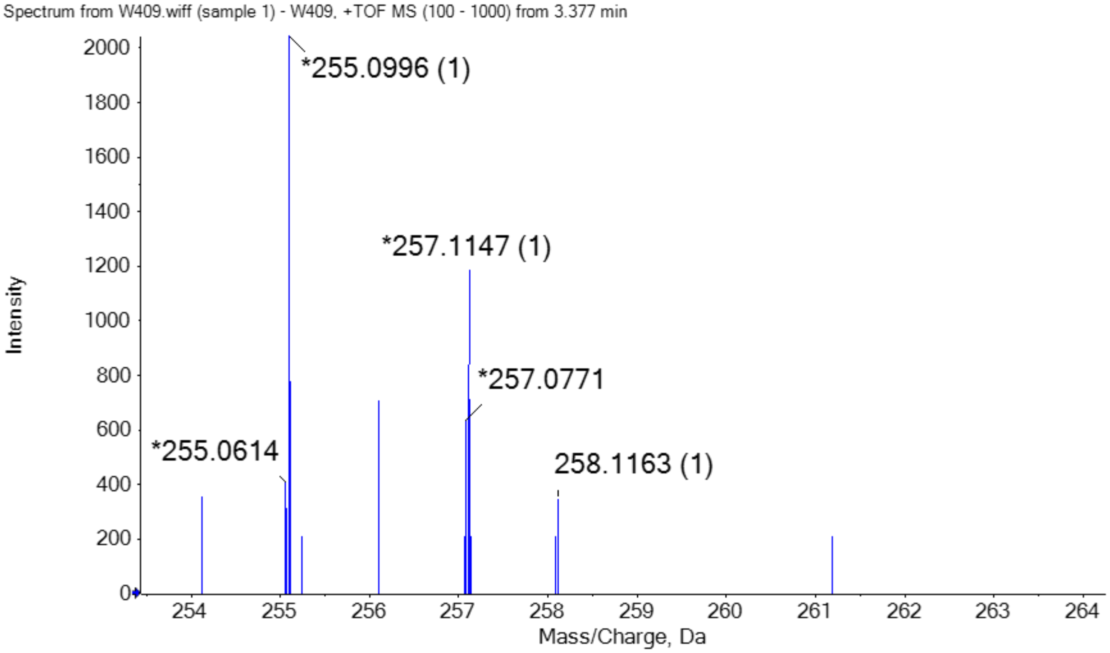


**Formula Calculator Results**

| Measured m/z | Cal m/z | Error(mmu) | Error(ppm) | Ion Formula | Ion |
|---|---|---|---|---|---|
| 257.1147 | 257.1147 | -0.1 | -0.3 | C14H16F3O | $[M+H]^+$ |

HRESIMS spectrum of compound **8q**

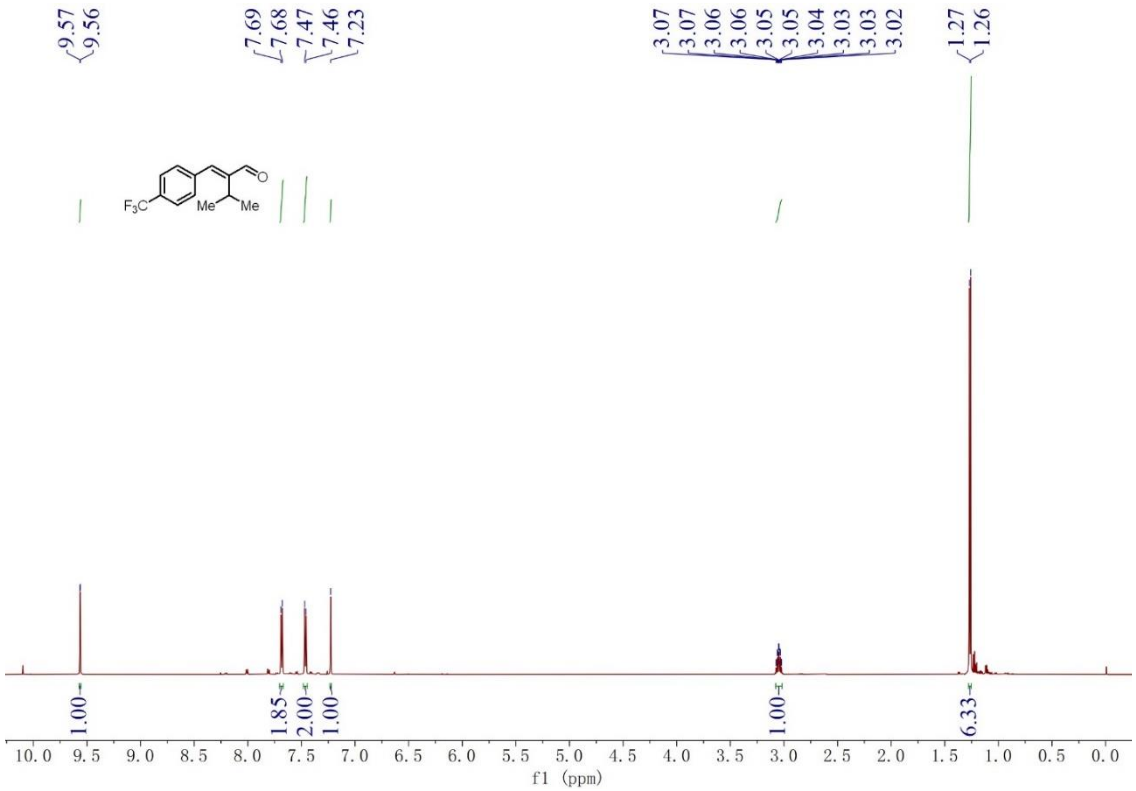


$^{1}H$ NMR spectrum of compound **8r** (600MHz, $CDCl_3$)

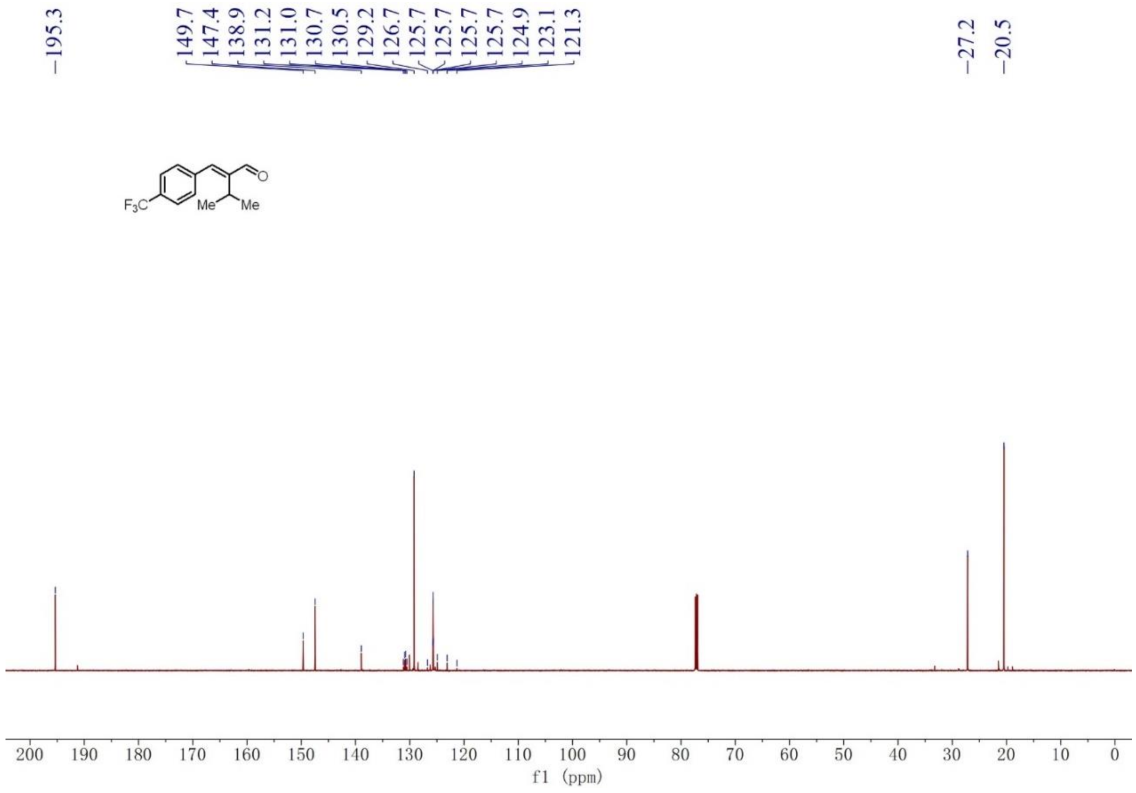


$^{13}C$ NMR spectrum of compound **8r** (151MHz, $CDCl_3$)

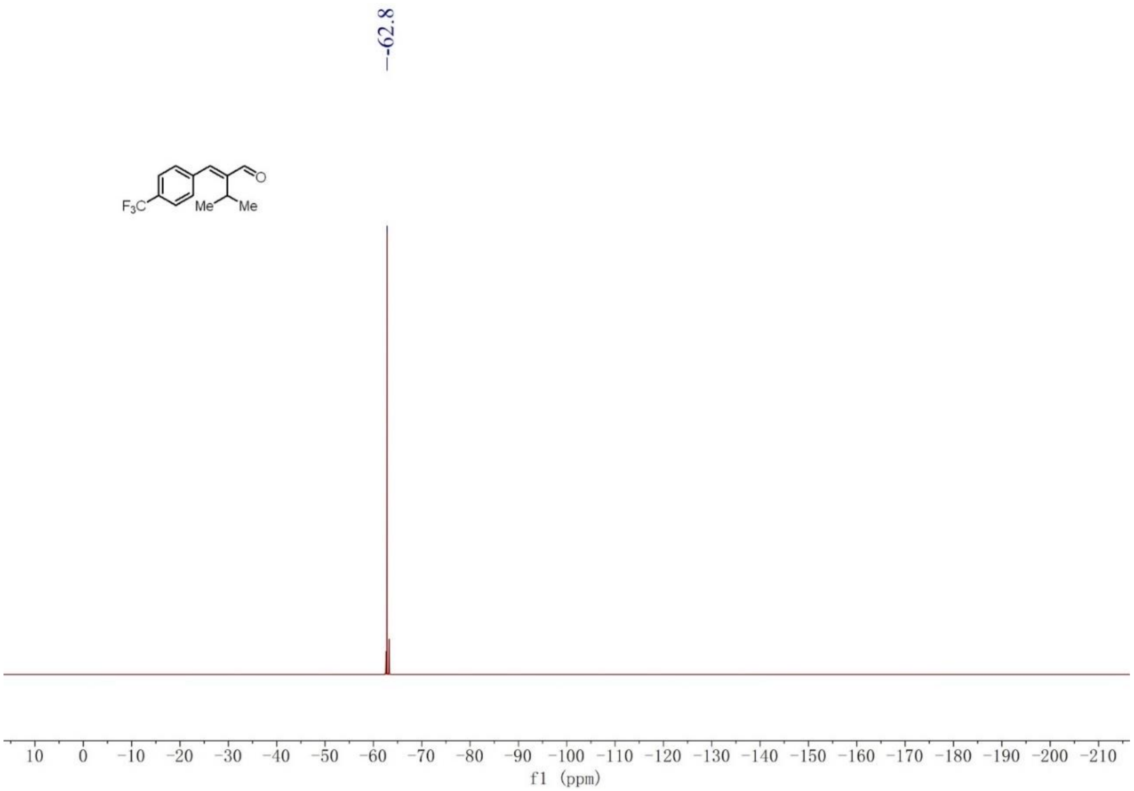


$^{19}$F NMR spectrum of compound **8r** (565MHz, $CDCl_3$)

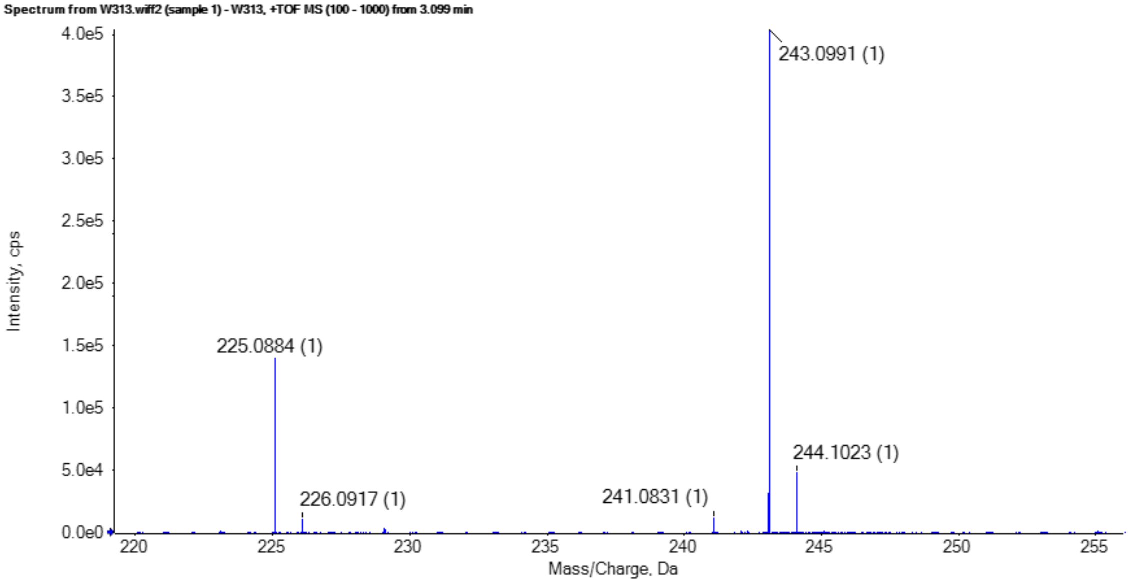


## Formula Calculator Results

| Measured m/z | Cal m/z | Error(mmu) | Error(ppm) | Ion Formula | Ion |
|---|---|---|---|---|---|
| 243.0991 | 243.0991 | 0 | 0 | C13H14F3O | $[M+H]^+$ |

HRESIMS spectrum of compound **8r**

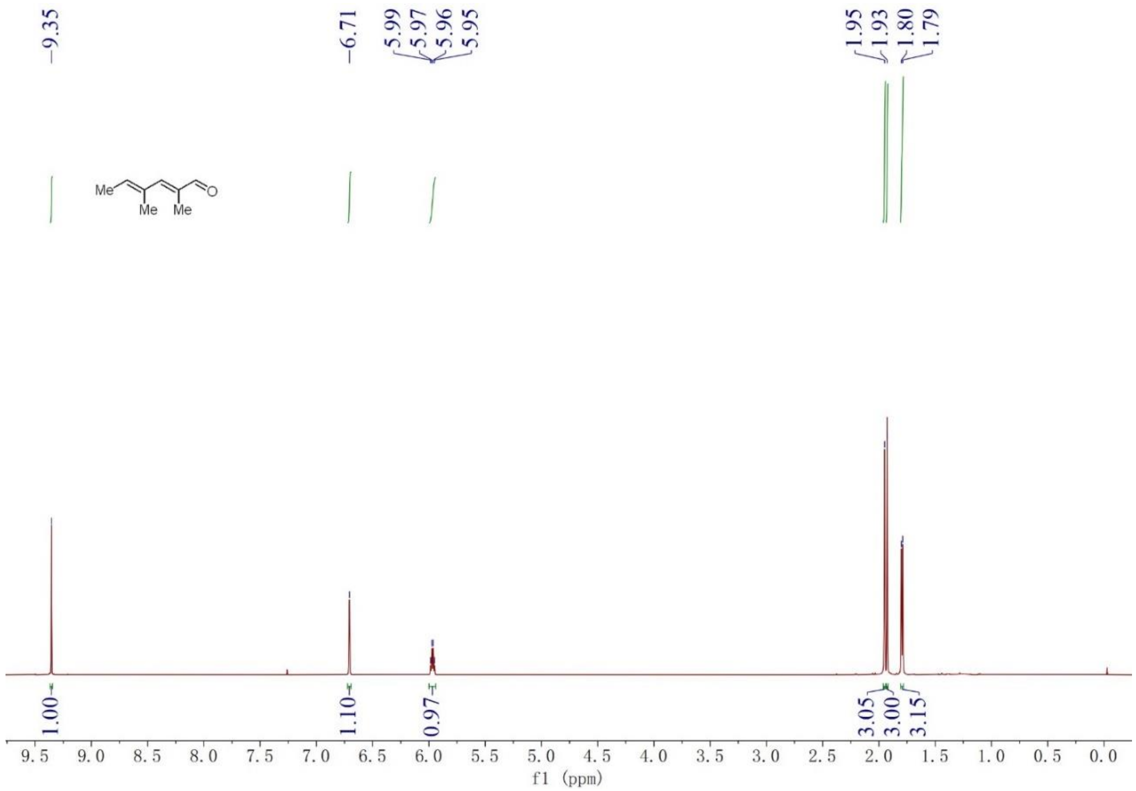


$^{1}H$ NMR spectrum of compound **8s** (600MHz, $CDCl_3$)

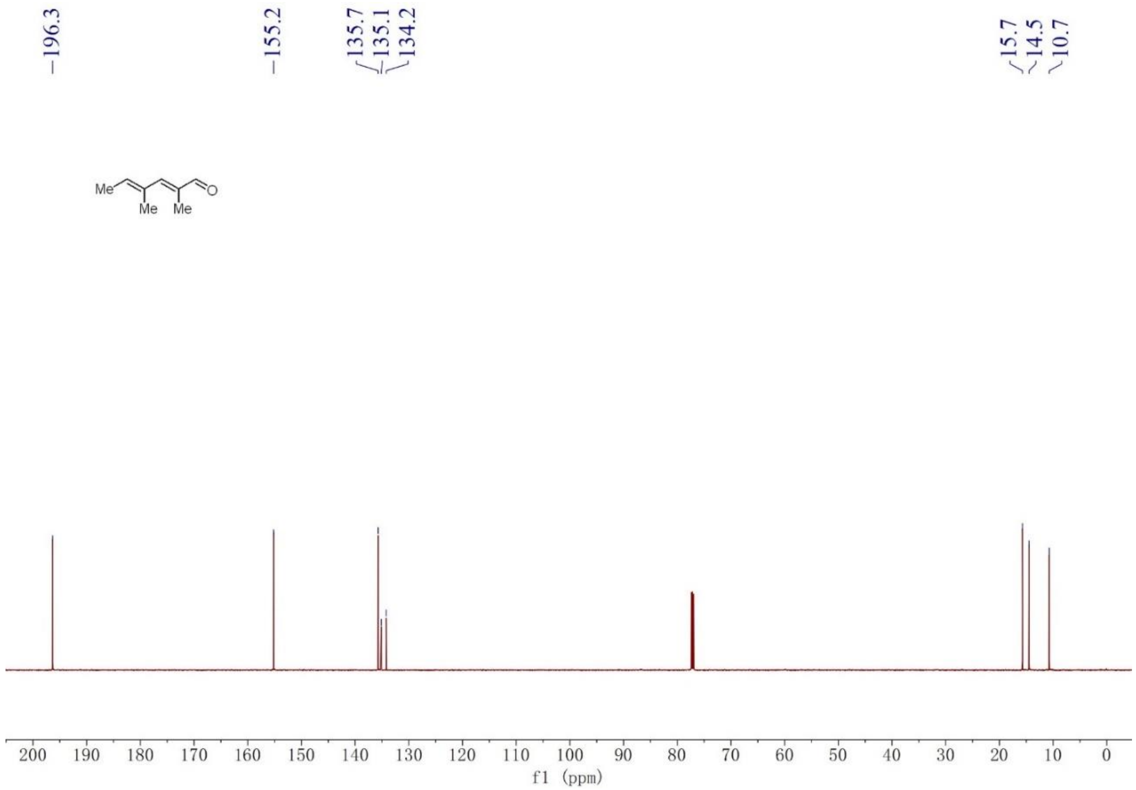


$^{13}C$ NMR spectrum of compound **8s** (151MHz, $CDCl_3$)

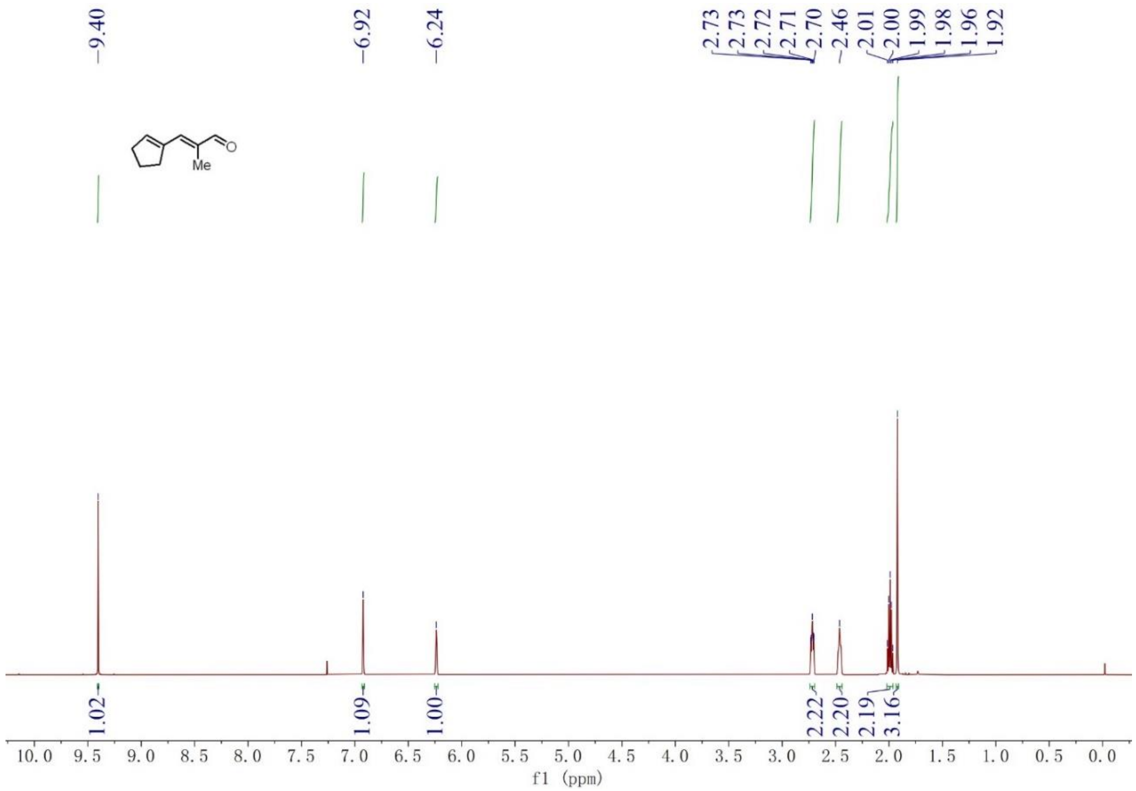


$^{1}$H NMR spectrum of compound **8t** (600MHz, $CDCl_3$)

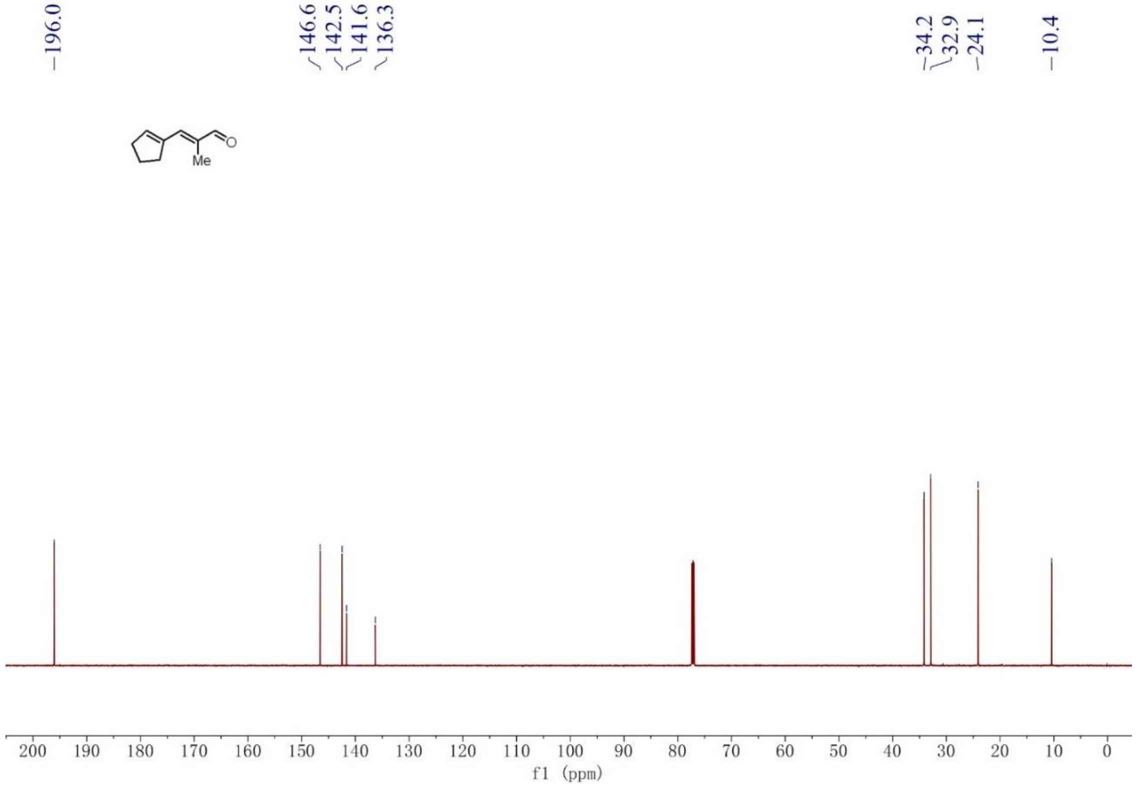


$^{13}$C NMR spectrum of compound **8t** (151MHz, $CDCl_3$)

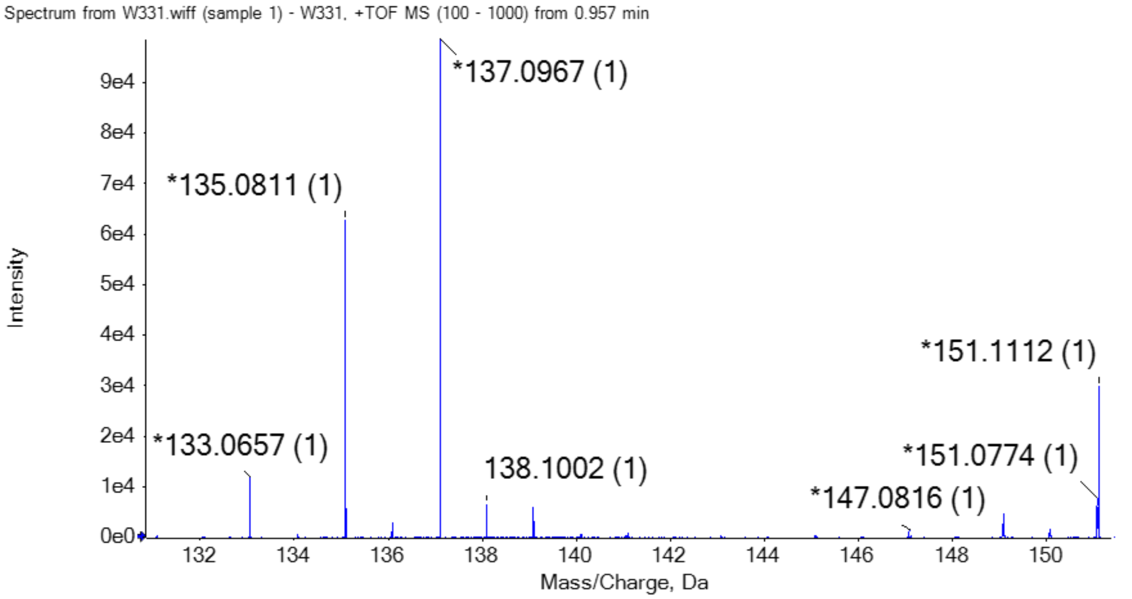


## Formula Calculator Results

| Measure m/z | Cal m/z | Error(mmu) | Error(ppm) | Ion Formula | Ion |
|---|---|---|---|---|---|
| 137.0967 | 137.0960 | 0.6 | 4.4 | C9H13O | $[M+H]^+$ |

HRESIMS spectrum of compound **8t**

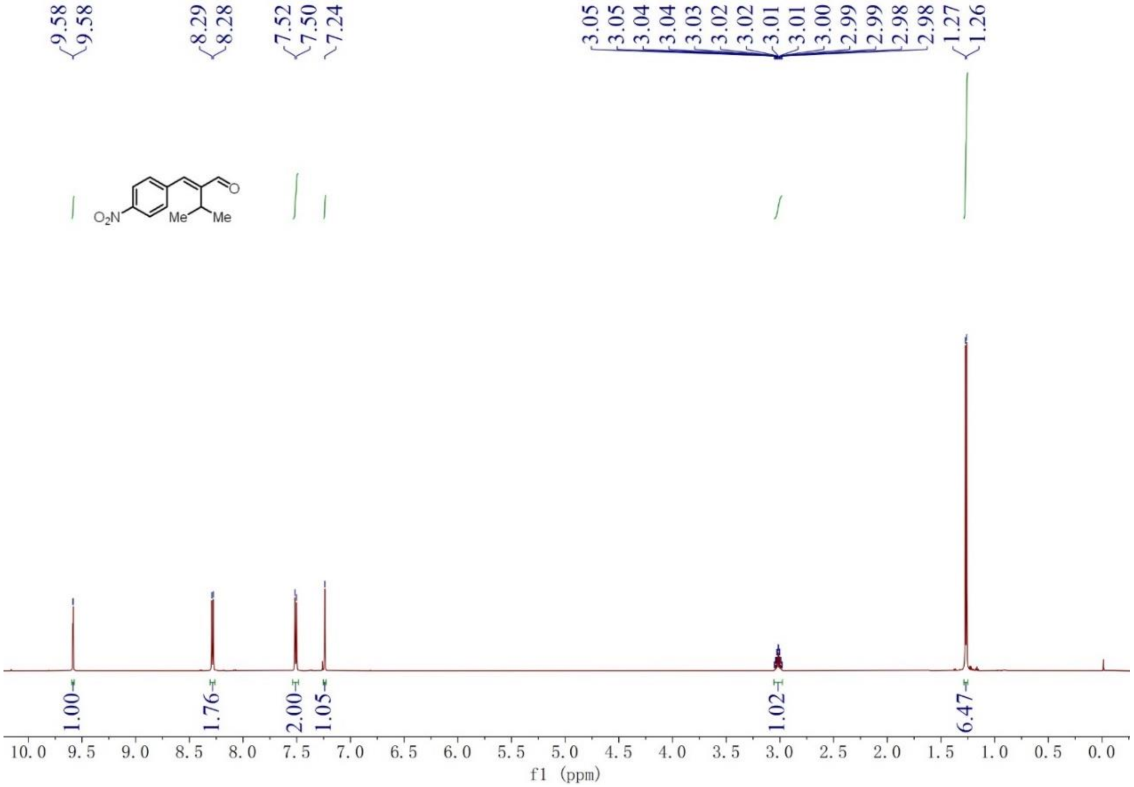


$^1$H NMR spectrum of compound **8v** (600MHz, $CDCl_3$)

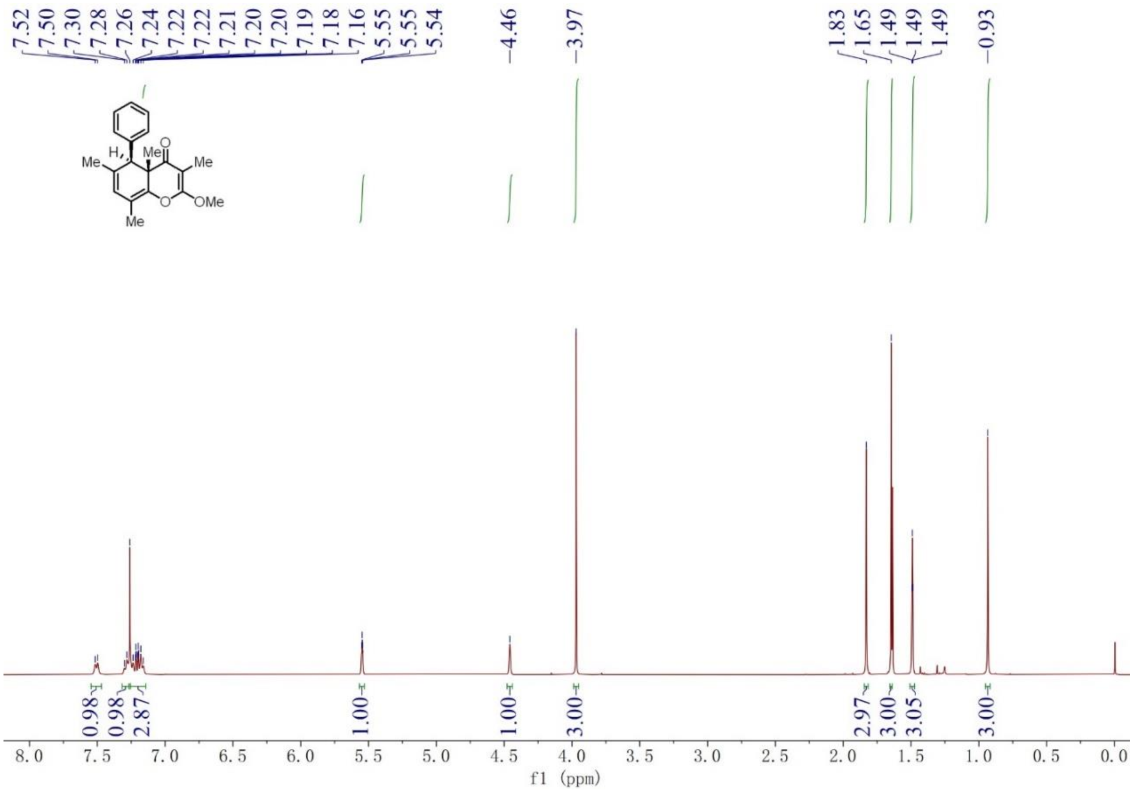

$^{1}H$ NMR spectrum of compound **10a** (400MHz, $CDCl_3$)

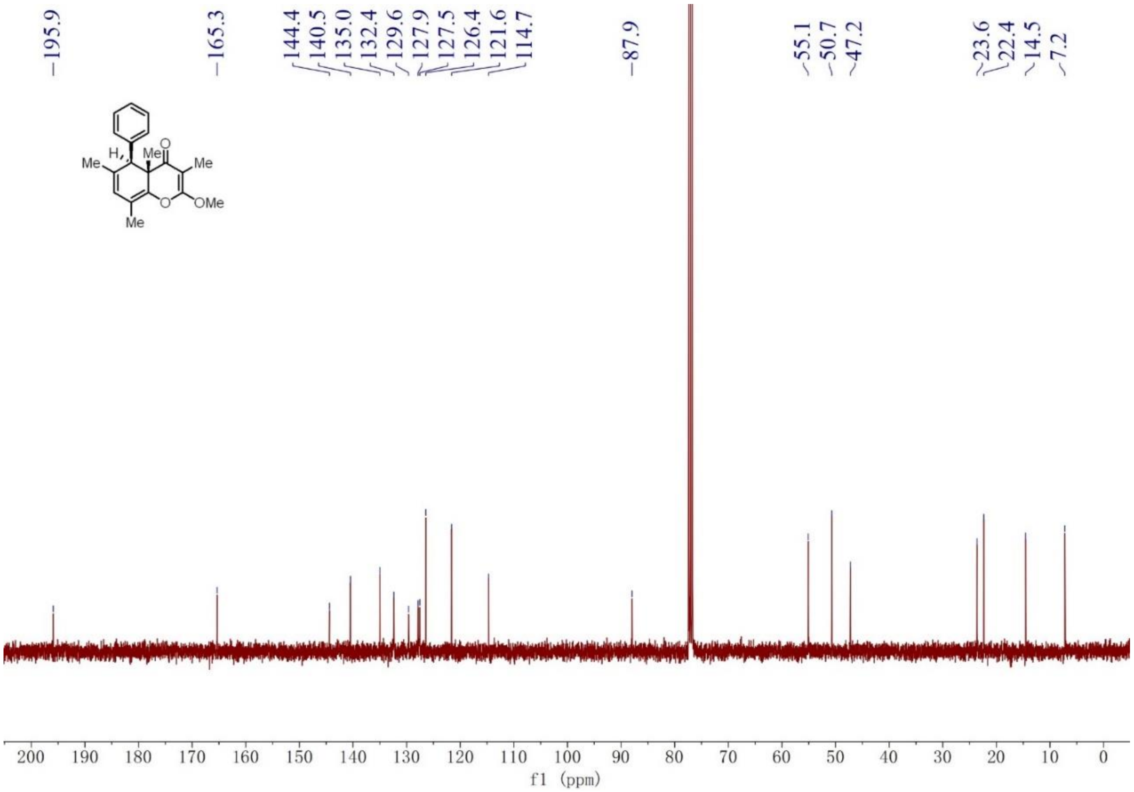

$^{13}C$ NMR spectrum of compound **10a** (101MHz, $CDCl_3$)

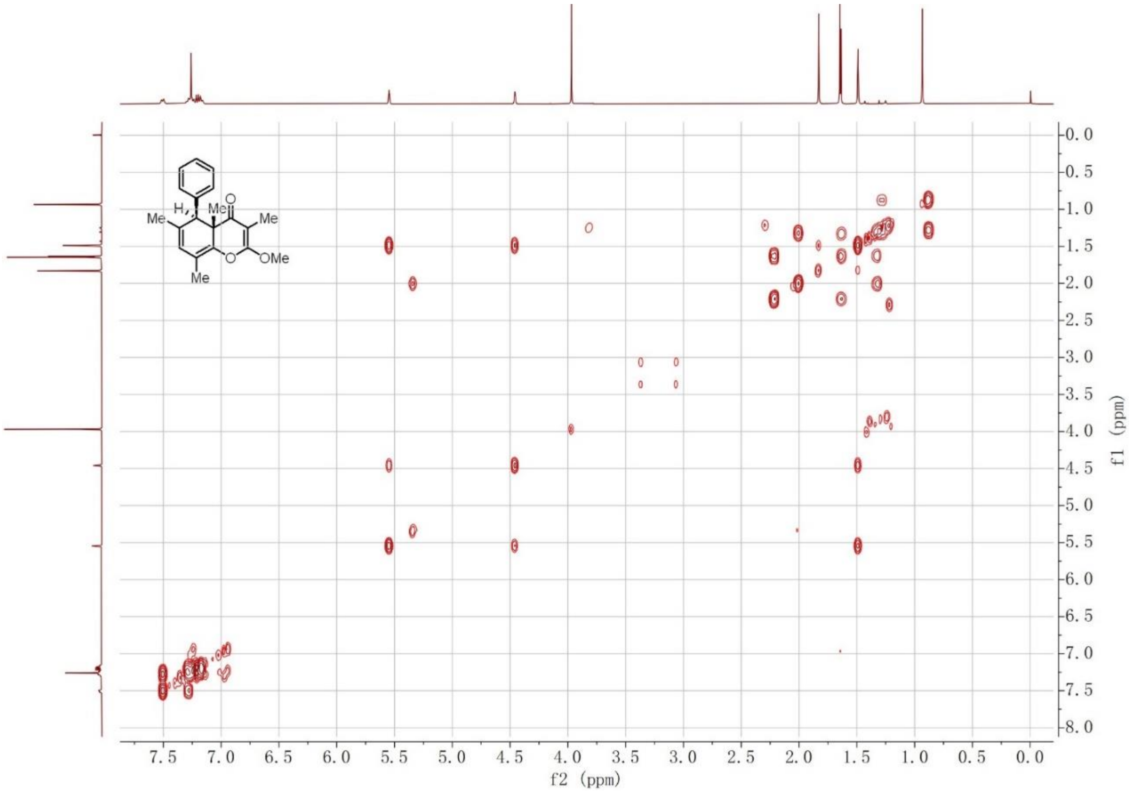

$^1$H-$^1$H COSY spectrum of compound **10a** (600 MHz, $CDCl_3$)

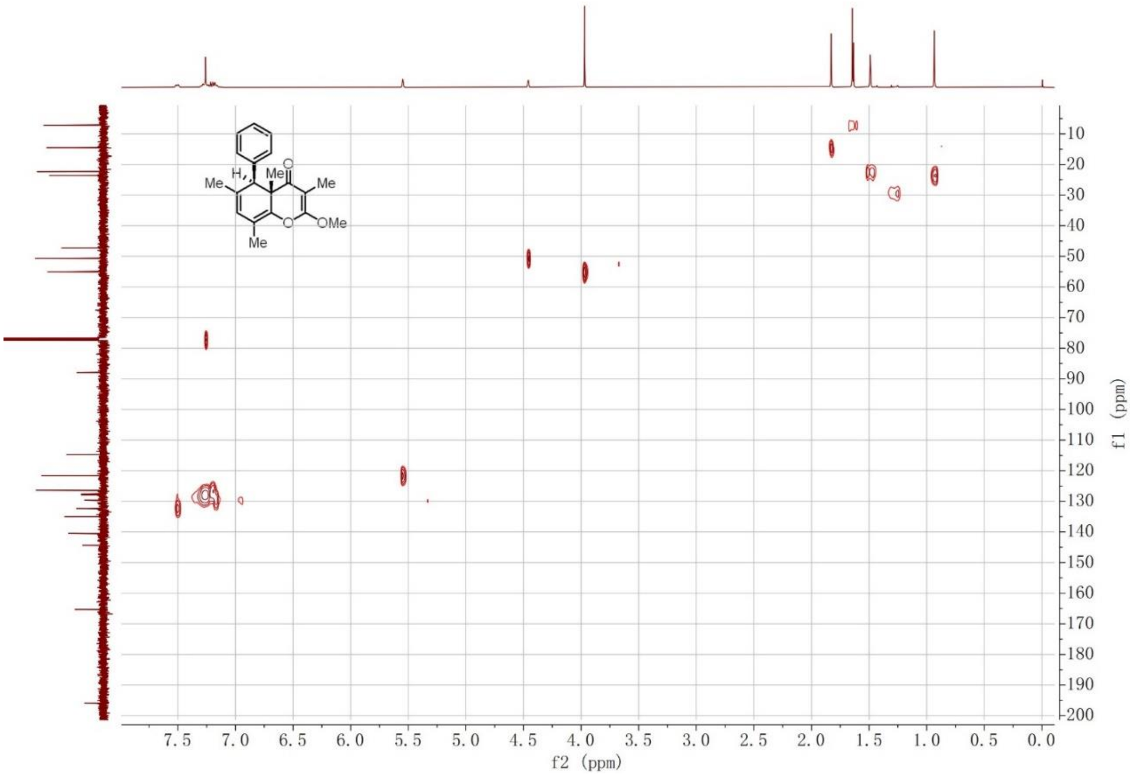

HSQC spectrum of compound **10a** (600 MHz, $CDCl_3$)

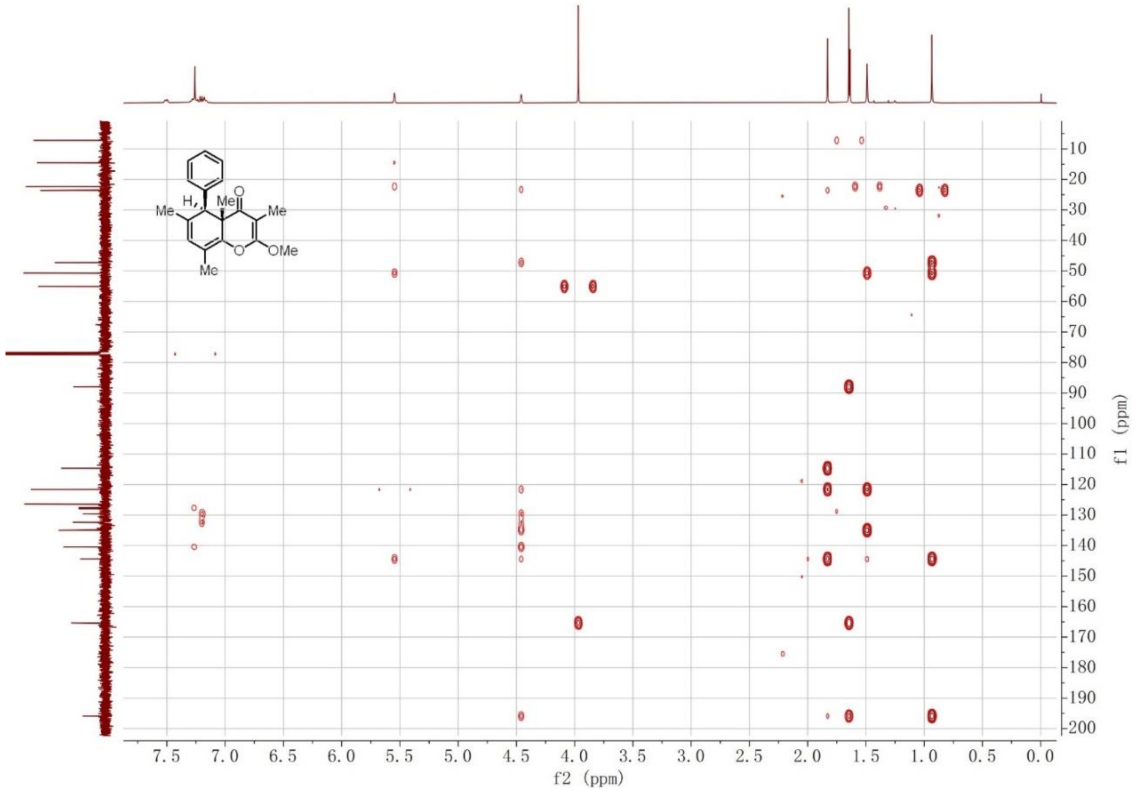

HMBC spectrum of compound **10a** (600 MHz, $CDCl_3$)

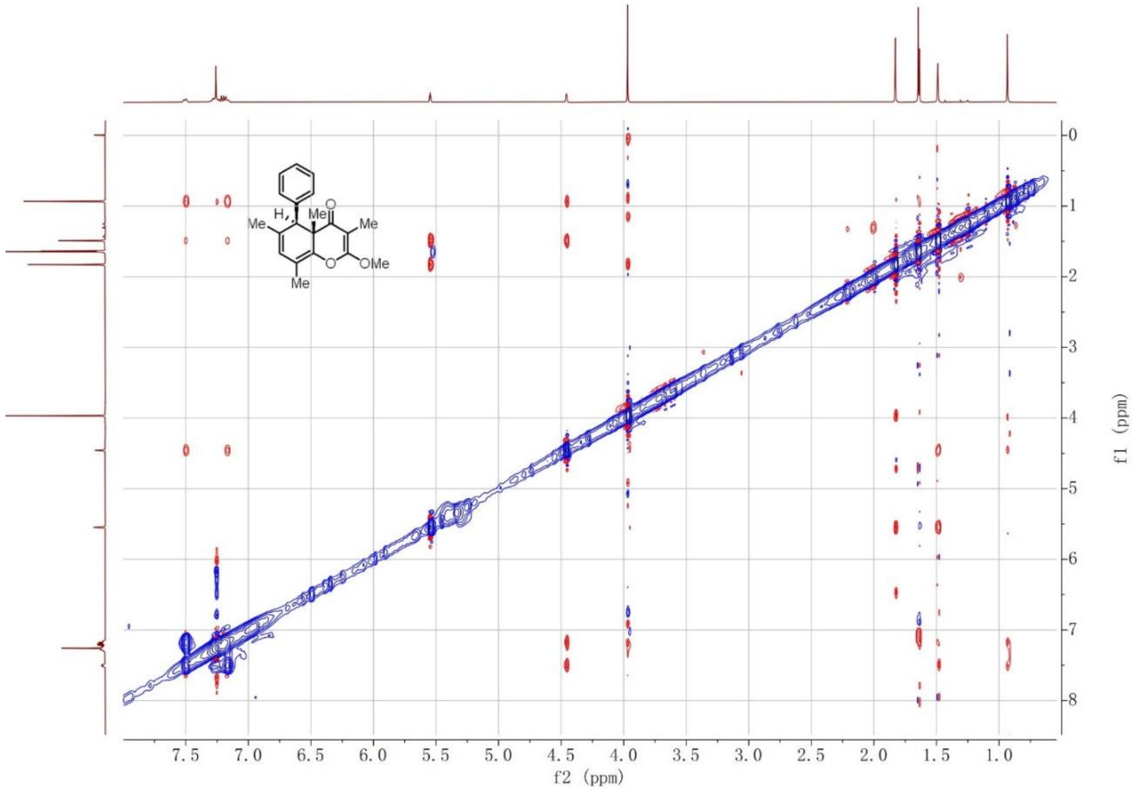

NOESY spectrum of compound **10a** (600 MHz, $CDCl_3$)

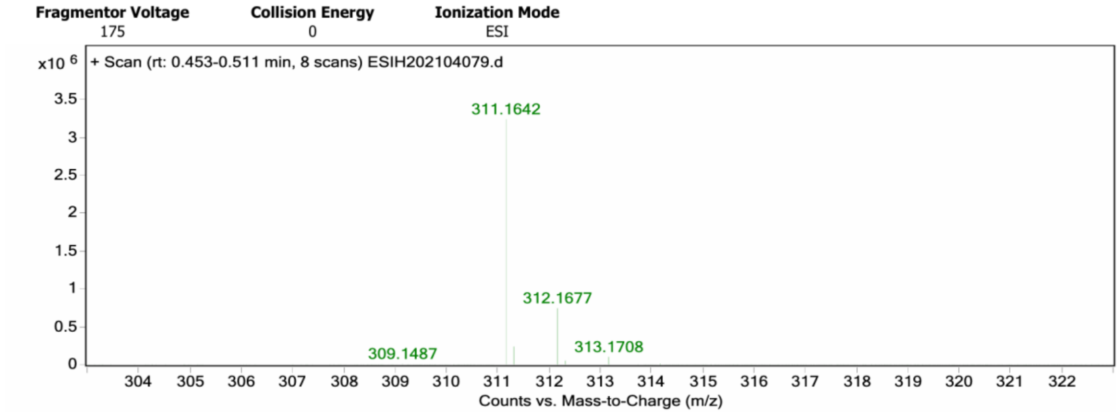


**Formula Calculator Results**

| m/z | Calc m/z | Diff (mDa) | Diff (ppm) | Ion Formula | Ion |
|---|---|---|---|---|---|
| 311.1642 | 311.1642 | -0.02 | -0.07 | C20 H23 O3 | (M+H)+ |

HRESIMS spectrum of compound **10a**

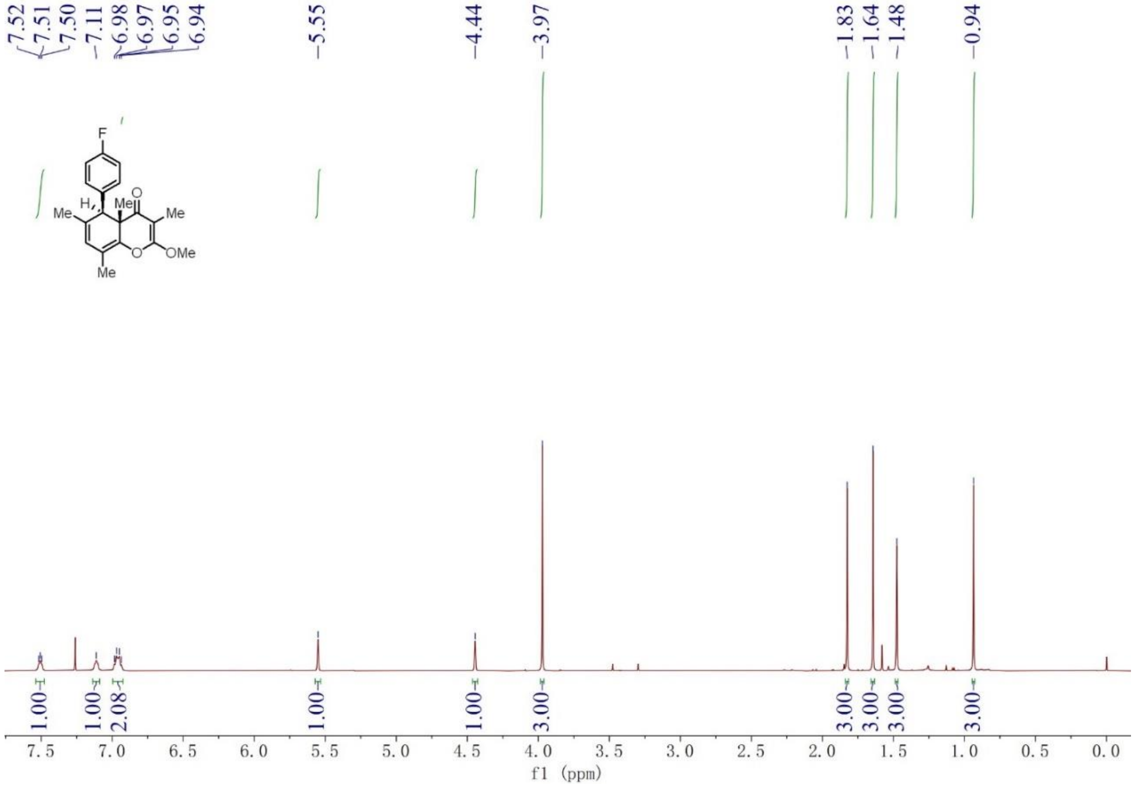


$^{1}$H NMR spectrum of compound **10b** (600MHz, $CDCl_3$)

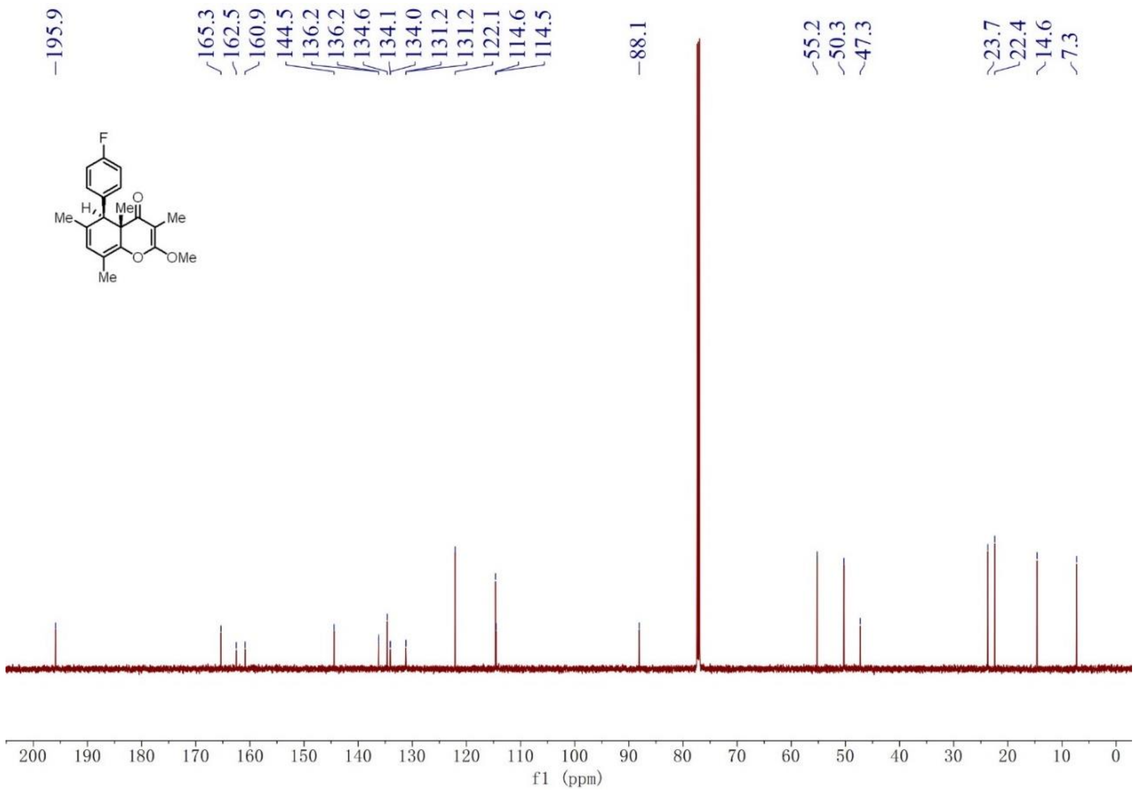

$^{13}$C NMR spectrum of compound **10b** (151MHz, $CDCl_3$)

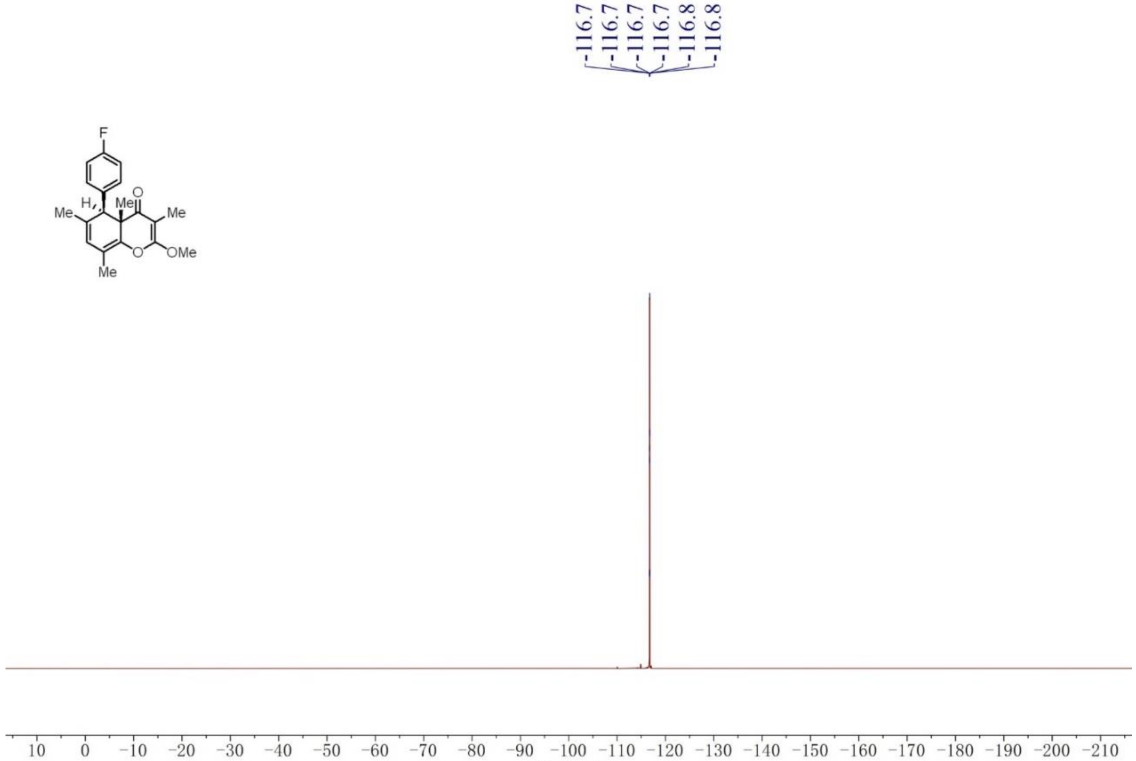

$^{19}$F NMR spectrum of compound **10b** (565MHz, $CDCl_3$)

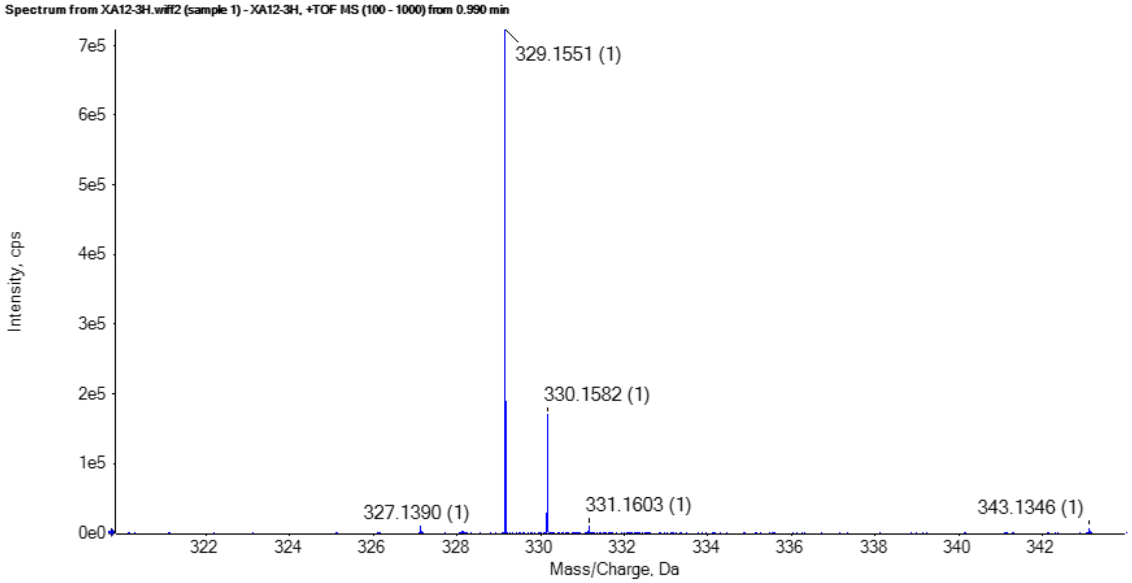


**Formula Calculator Results**

| Measured m/z | Cal m/z | Error(mmu) | Error(ppm) | Ion Formula | Ion |
|---|---|---|---|---|---|
| 329.1551 | 329.1548 | 0.4 | 1.1 | C20H22FO3 | $[M+H]^+$ |

HRESIMS spectrum of compound **10b**

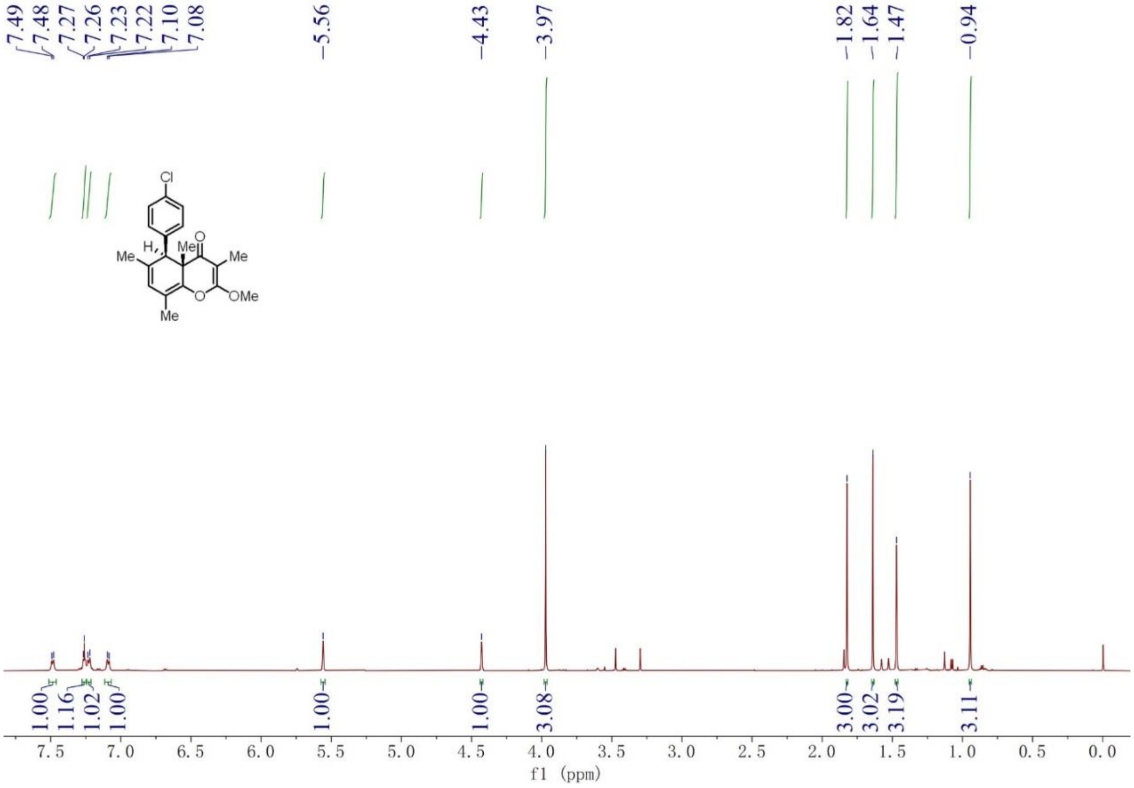


$^{1}$H NMR spectrum of compound **10c** (600MHz, $CDCl_3$)

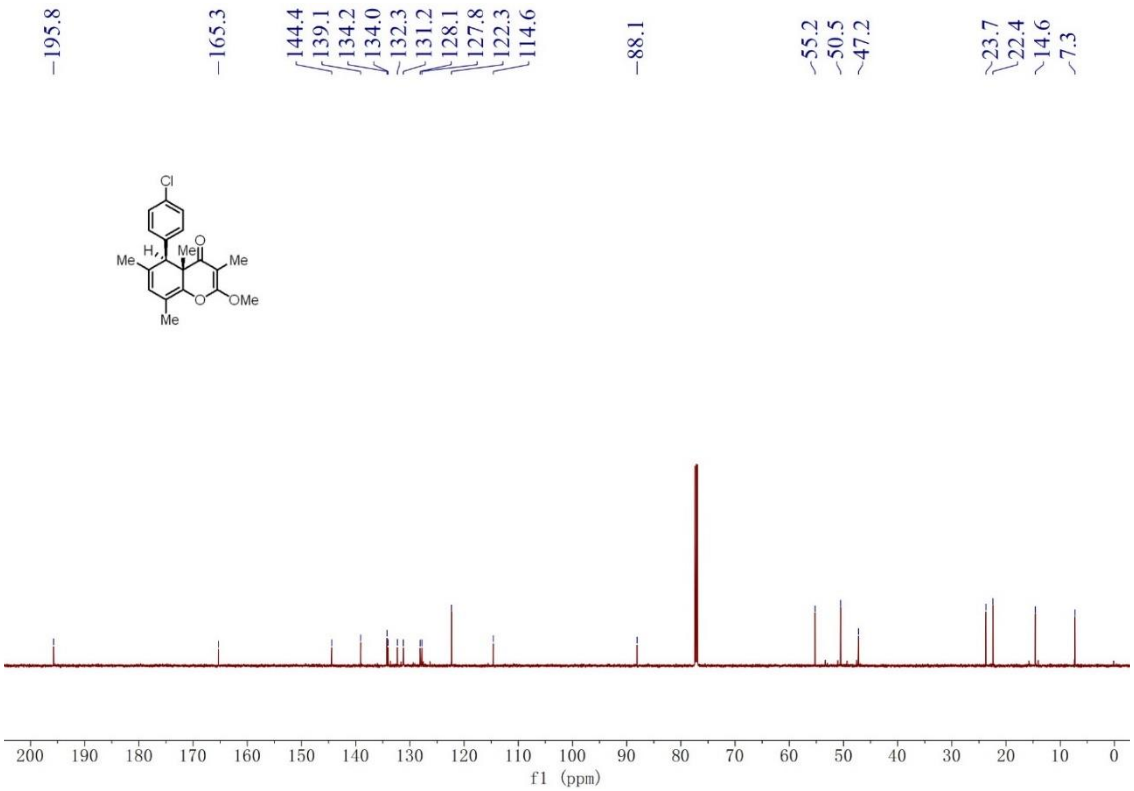

$^{13}C$ NMR spectrum of compound **10c** (151MHz, $CDCl_3$)

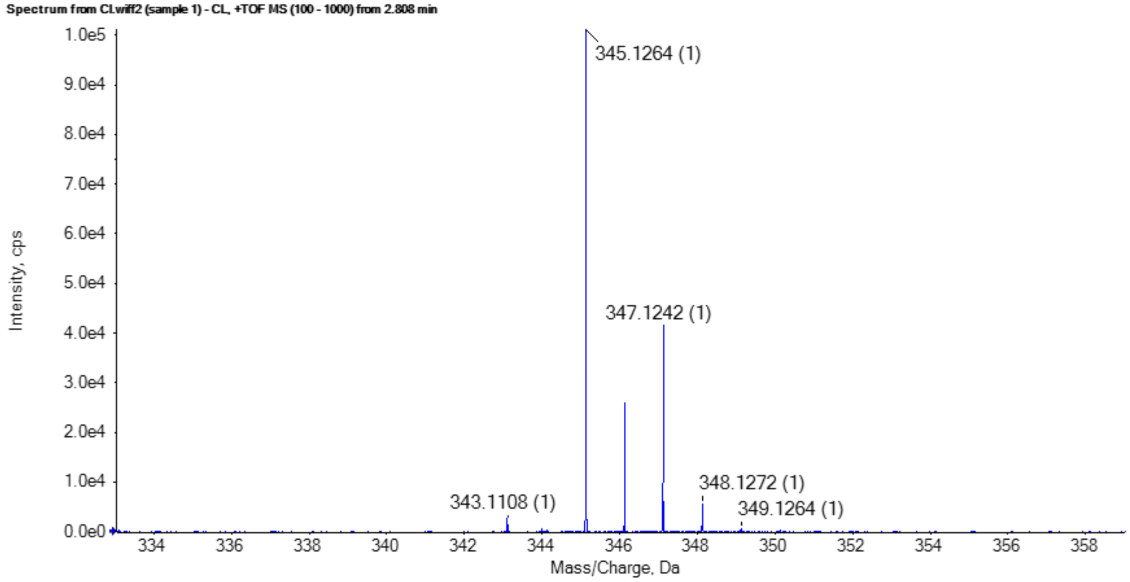


**Formula Calculator Results**

| Measured m/z | Cal m/z | Error(mmu) | Error(ppm) | Ion Formula | Ion |
|---|---|---|---|---|---|
| 345.1264 | 345.1252 | 1.2 | 3.4 | C20H22ClO3 | [M+H]$^+$ |

HRESIMS spectrum of compound **10c**

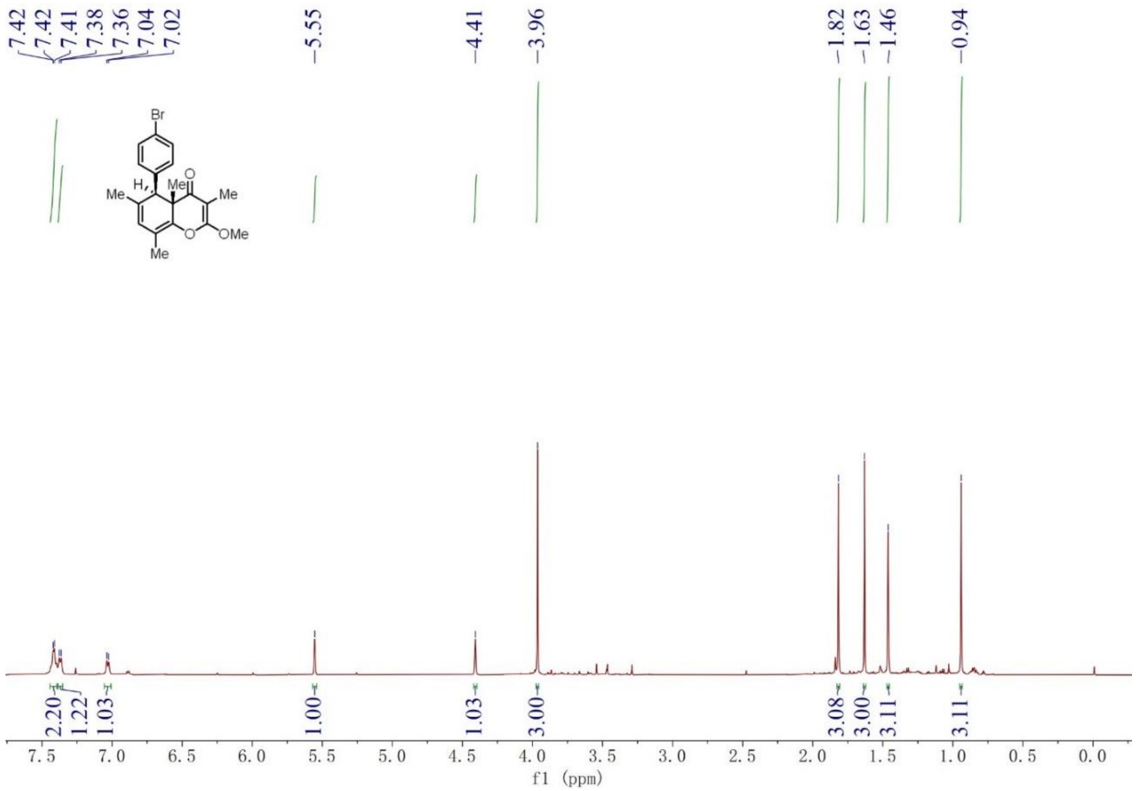


$^{1}H$ NMR spectrum of compound **10d** (600MHz, $CDCl_3$)

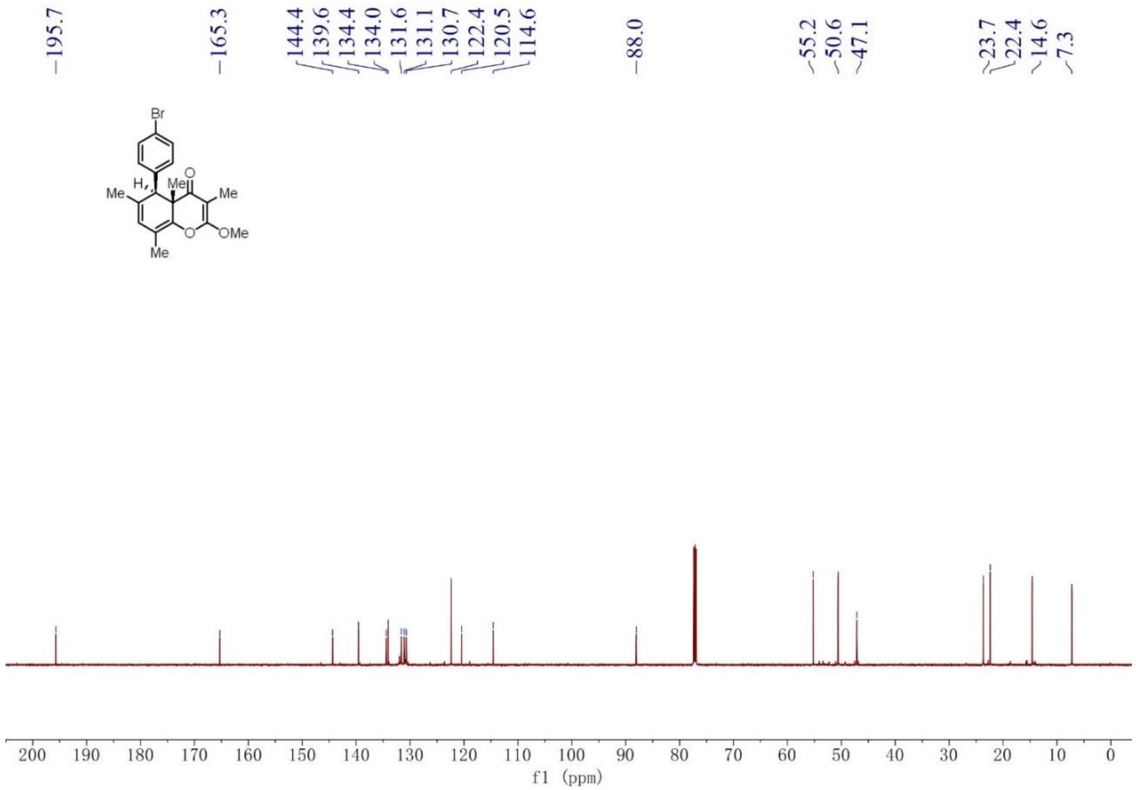


$^{13}C$ NMR spectrum of compound **10d** (151MHz, $CDCl_3$)

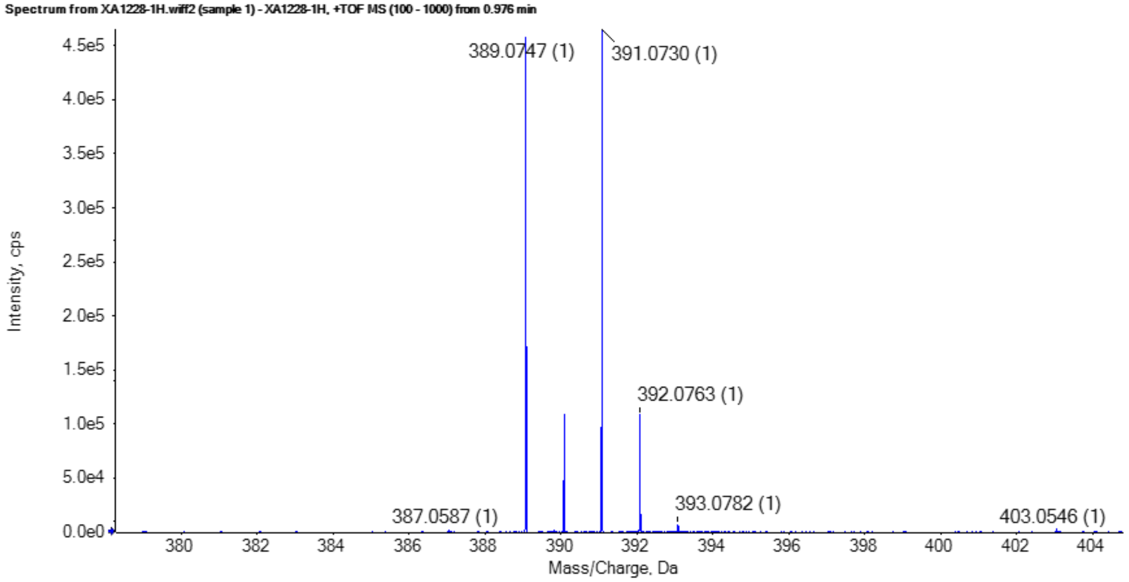


**Formula Calculator Results**

| Measured m/z | Cal m/z | Error(mmu) | Error(ppm) | Ion Formula | Ion |
|---|---|---|---|---|---|
| 389.0747 | 389.0747 | 0 | 0 | C20H22BrO3 | $[M+H]^+$ |

HRESIMS spectrum of compound **10d**

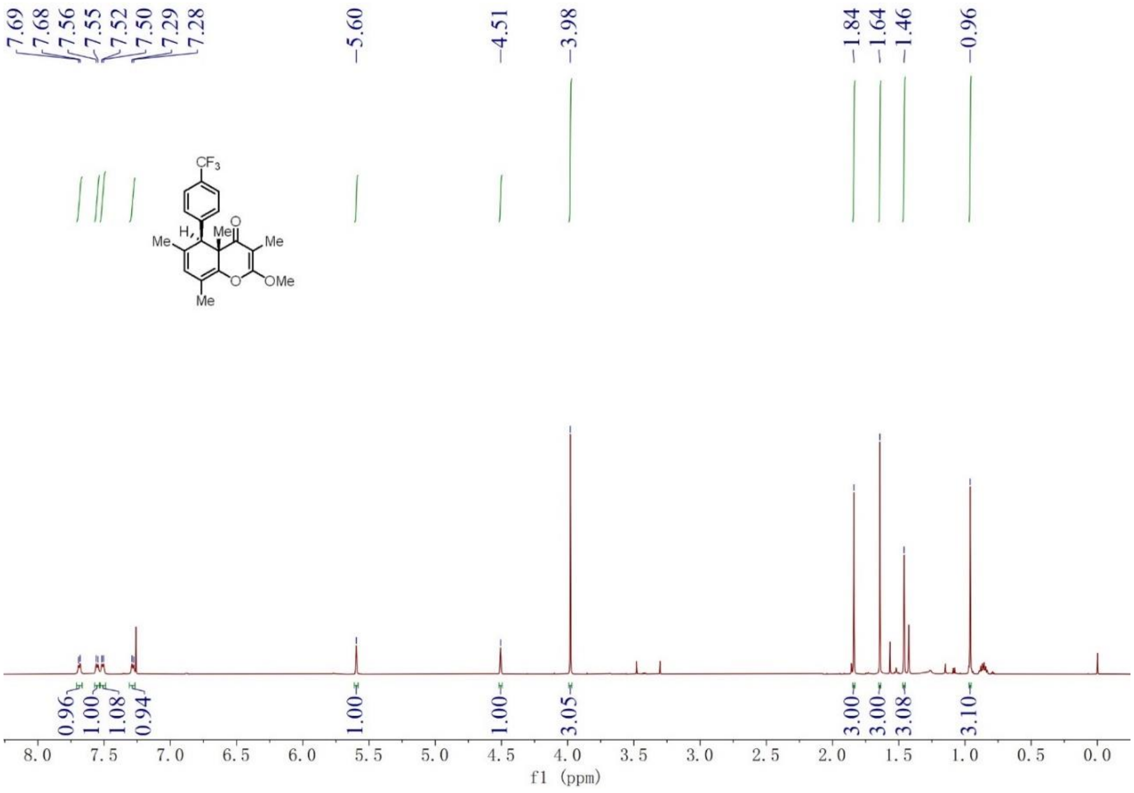


$^1$H NMR spectrum of compound **10e** (600MHz, $CDCl_3$)

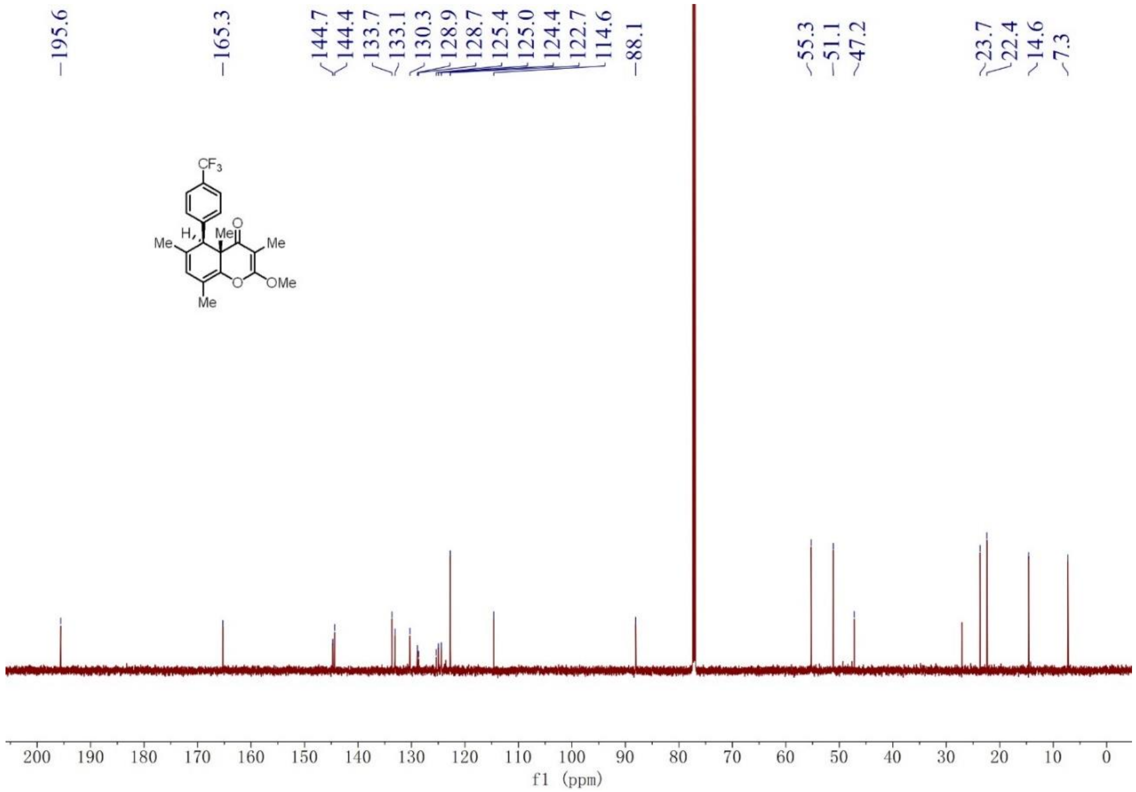


$^{13}C$ NMR spectrum of compound **10e** (151MHz, $CDCl_3$)

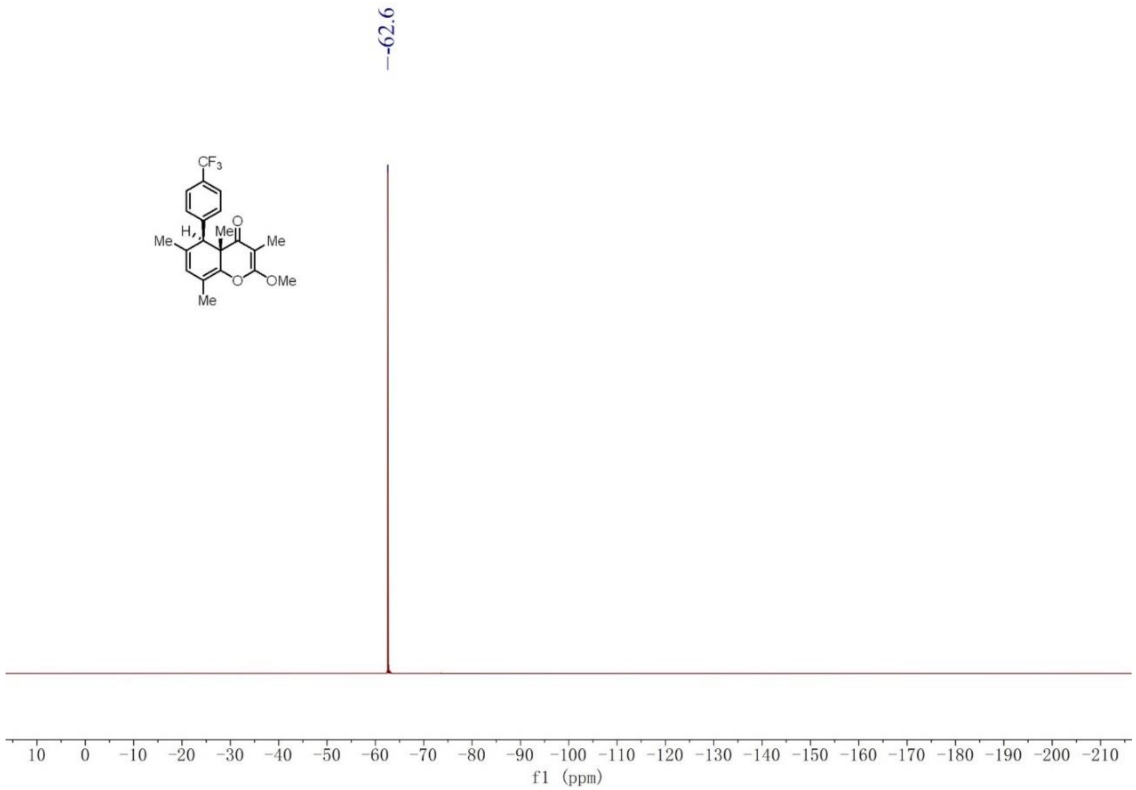


$^{19}F$ NMR spectrum of compound **10e** (565MHz, $CDCl_3$)

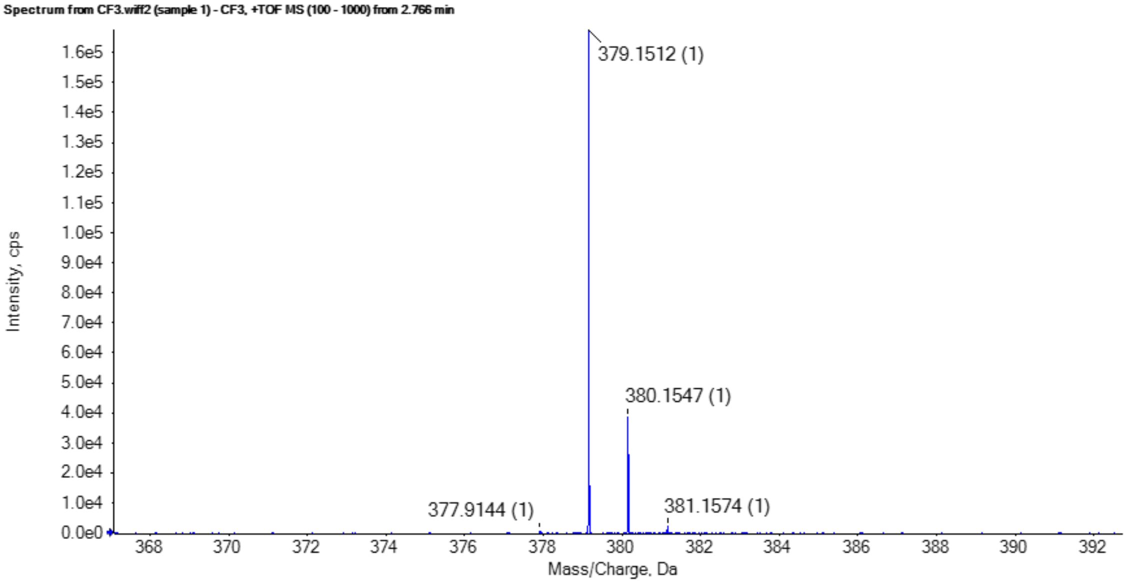


## Formula Calculator Results

| Measured m/z | Cal m/z | Error(mmu) | Error(ppm) | Ion Formula | Ion |
|---|---|---|---|---|---|
| 379.1512 | 379.1516 | -0.4 | -0.9 | C21H22F3O3 | $[M+H]^+$ |

HRESIMS spectrum of compound **10e**

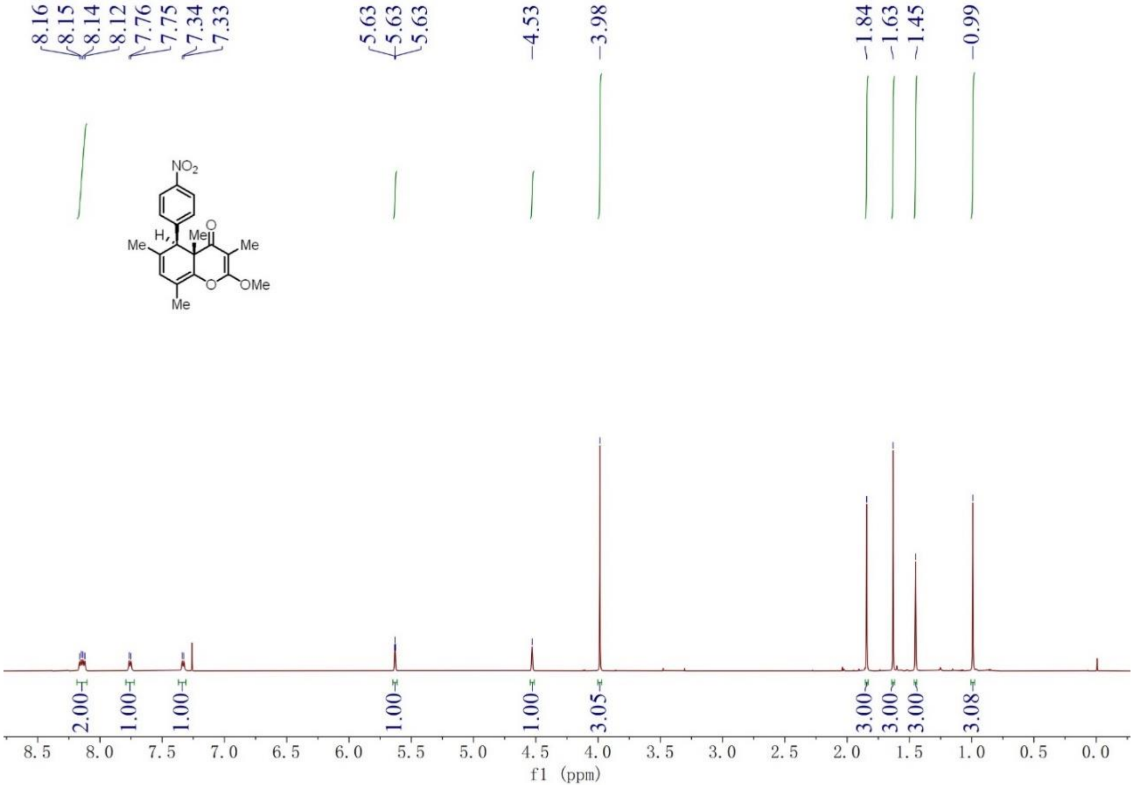


$^1$H NMR spectrum of compound **10f** (600MHz, $CDCl_3$)

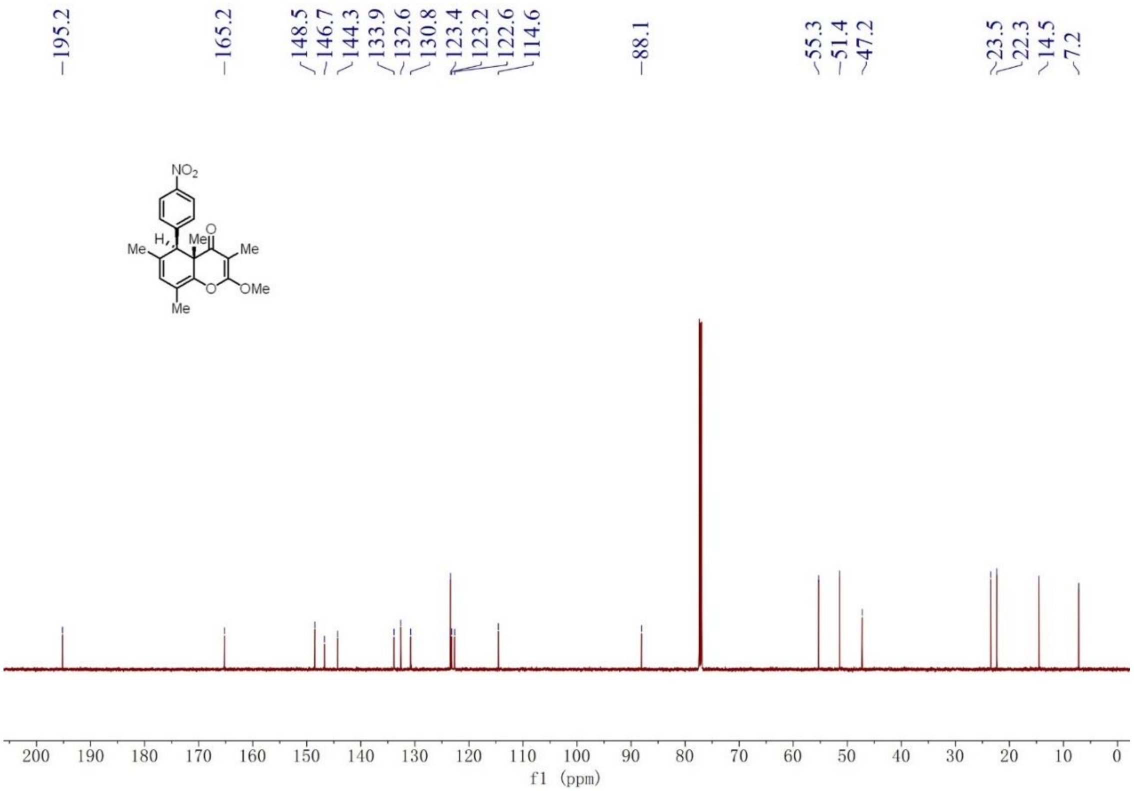


$^{13}$C NMR spectrum of compound **10f** (151MHz, $CDCl_3$)

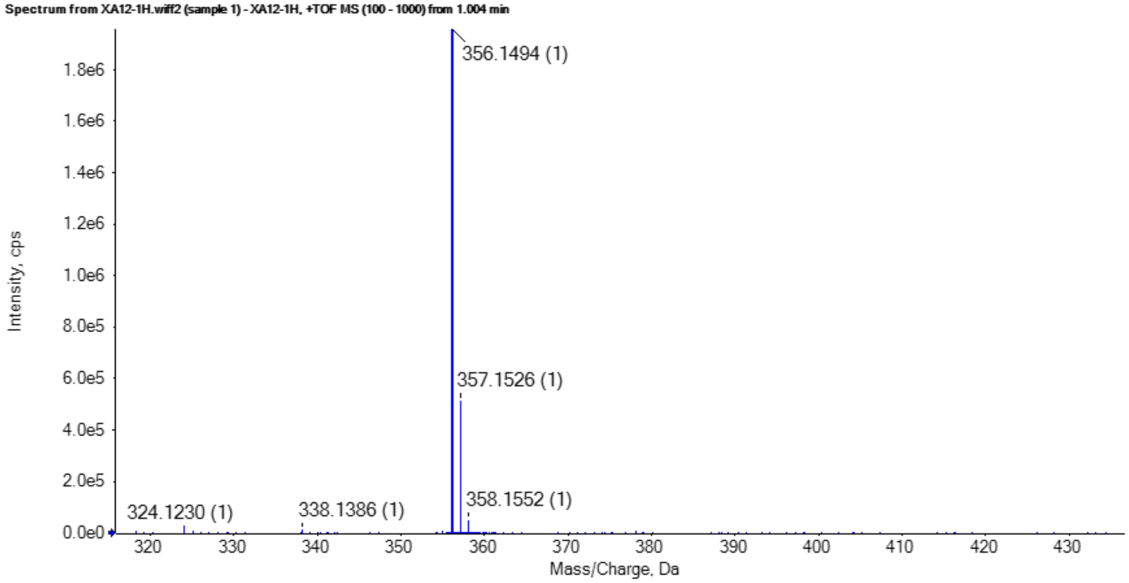


## Formula Calculator Results

| Measured m/z | Cal m/z | Error(mmu) | Error(ppm) | Ion Formula | Ion |
|---|---|---|---|---|---|
| 356.1494 | 356.1493 | 0.1 | 0.3 | C20H22NO5 | $[M+H]^+$ |

HRESIMS spectrum of compound **10f**

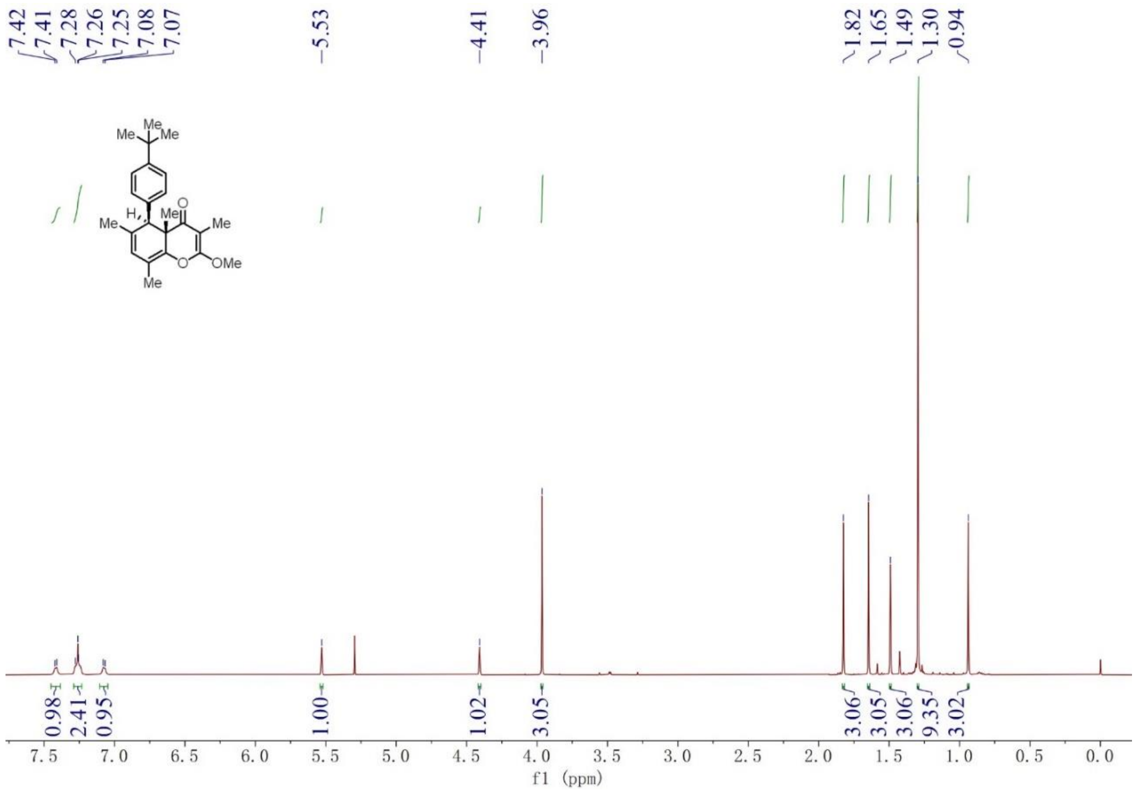

$^{1}$H NMR spectrum of compound **10g** (600MHz, $CDCl_3$)

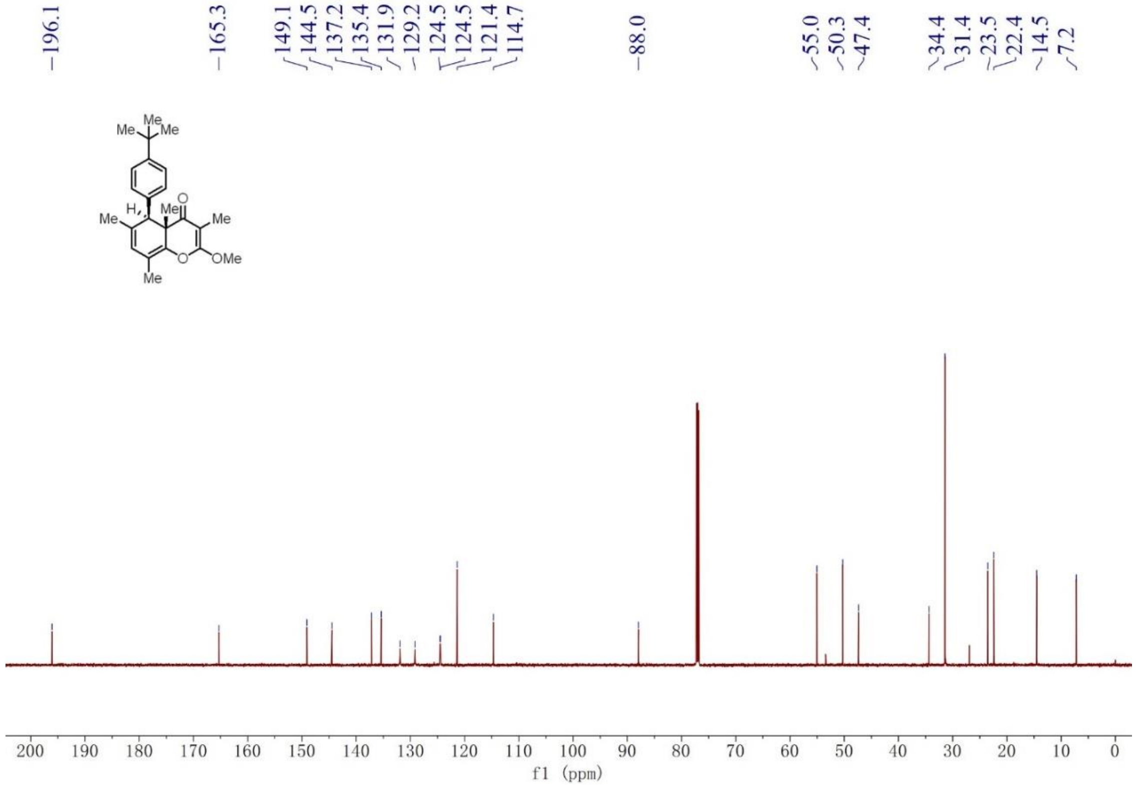

$^{13}$C NMR spectrum of compound **10g** (151MHz, $CDCl_3$)

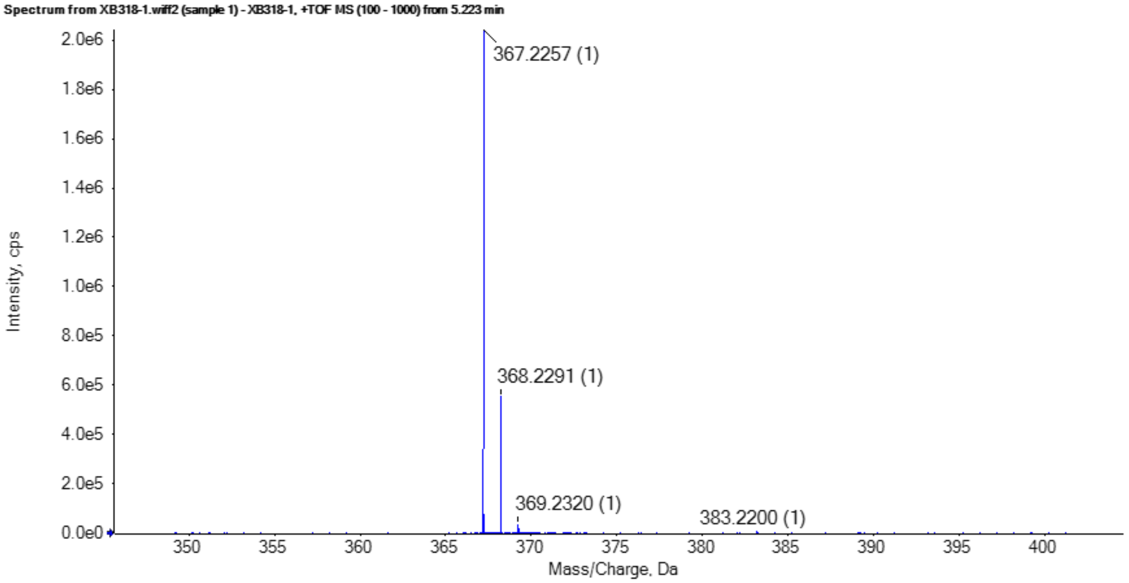


**Formula Calculator Results**

| Measured m/z | Cal m/z | Error(mmu) | Error(ppm) | Ion Formula | Ion |
|---|---|---|---|---|---|
| 367.2257 | 367.2268 | -1.1 | -3.0 | C24H31O3 | $[M+H]^+$ |

HRESIMS spectrum of compound **10g**

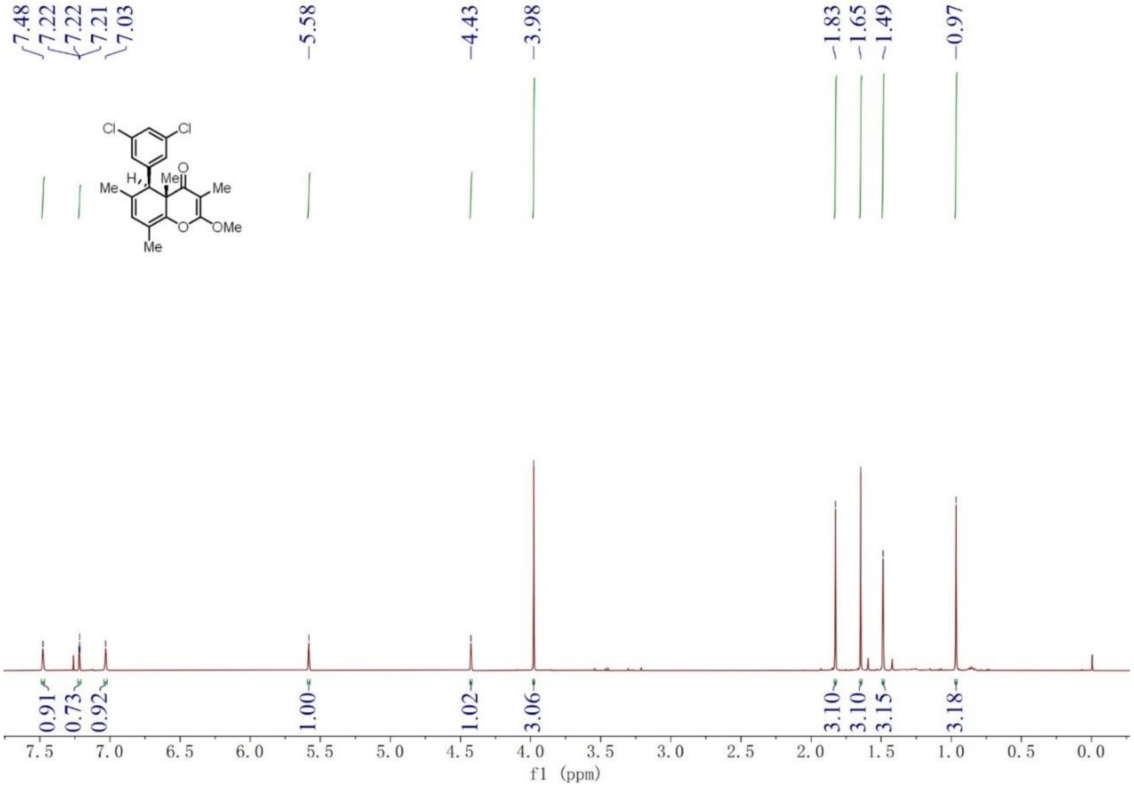


$^1$H NMR spectrum of compound **10h** (600MHz, $CDCl_3$)

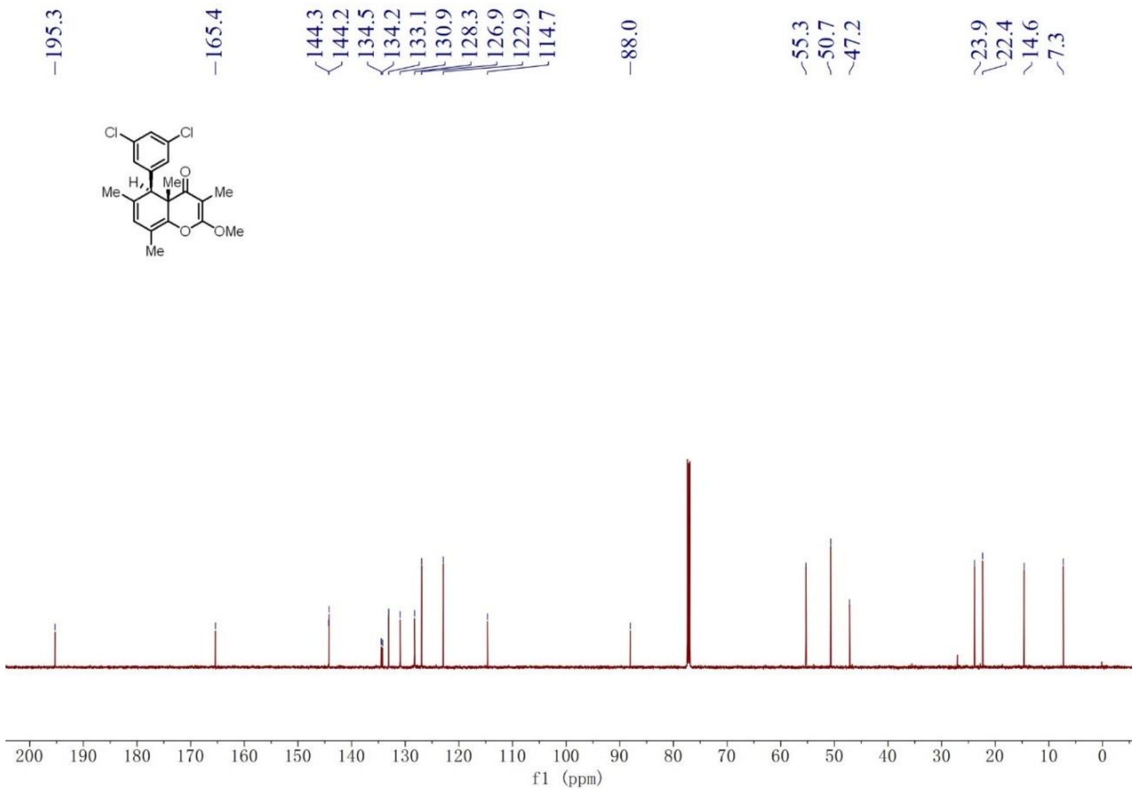


$^{13}$C NMR spectrum of compound **10h** (151MHz, $CDCl_3$)

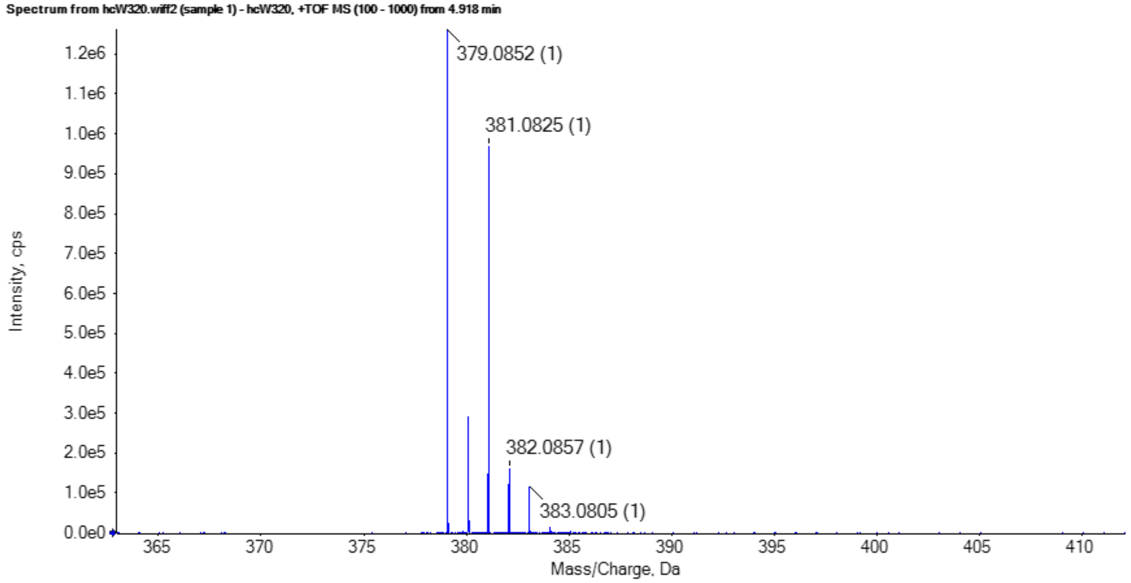


## Formula Calculator Results

| Measured m/z | Cal m/z | Error(mmu) | Error(ppm) | Ion Formula | Ion |
|---|---|---|---|---|---|
| 379.0852 | 379.0862 | -1.0 | -2.7 | C20H21Cl2O3 | $[M+H]^+$ |

HRESIMS spectrum of compound **10h**

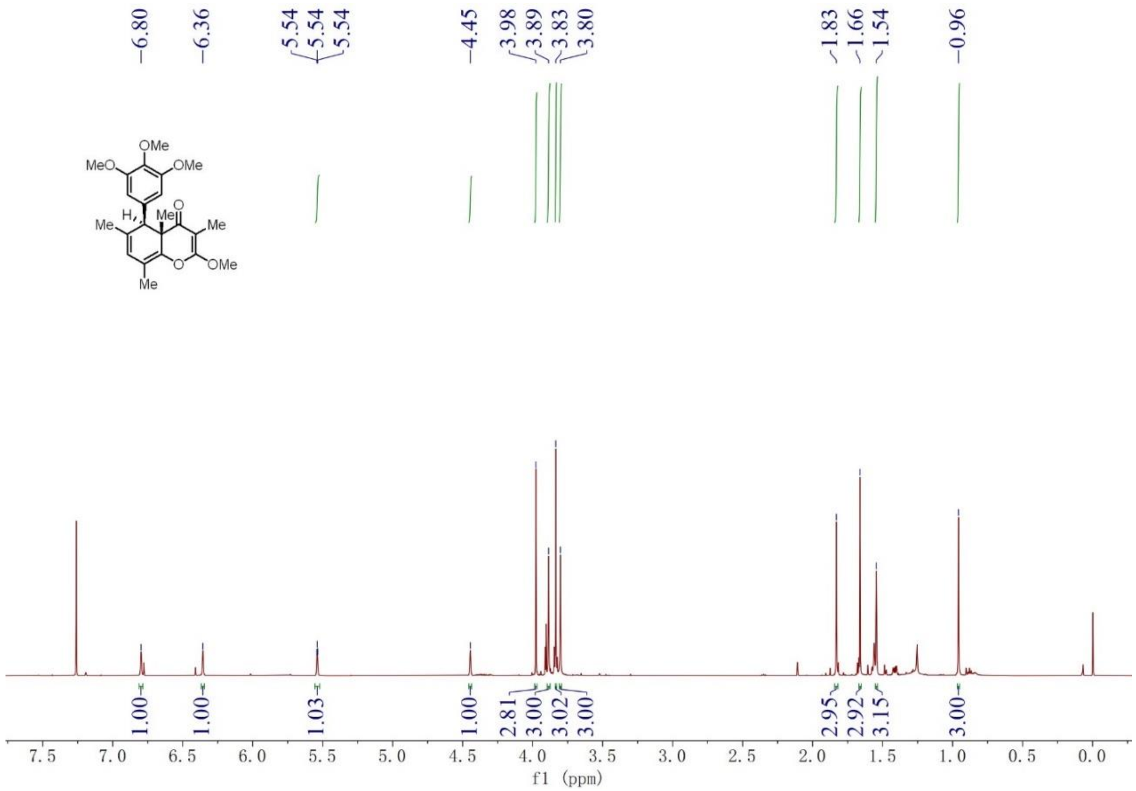

$^{1}$H NMR spectrum of compound **10i** (600MHz, $CDCl_3$)

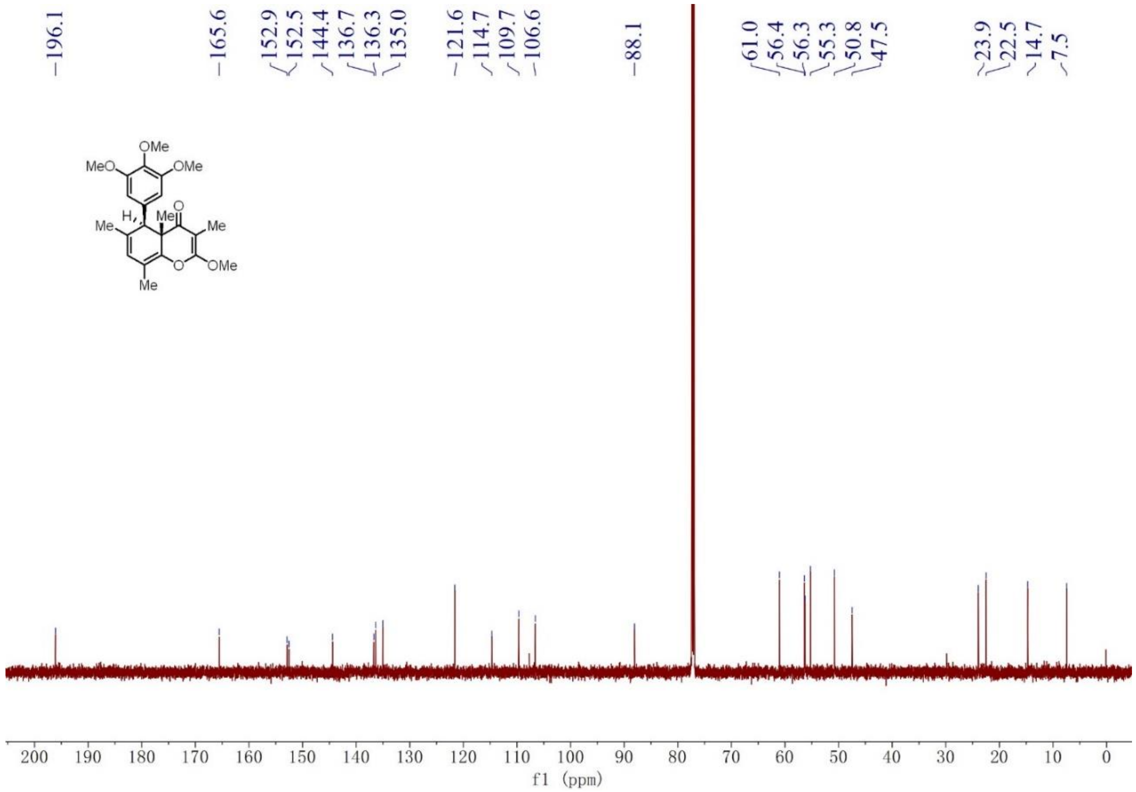

$^{13}$C NMR spectrum of compound **10i** (151MHz, $CDCl_3$)

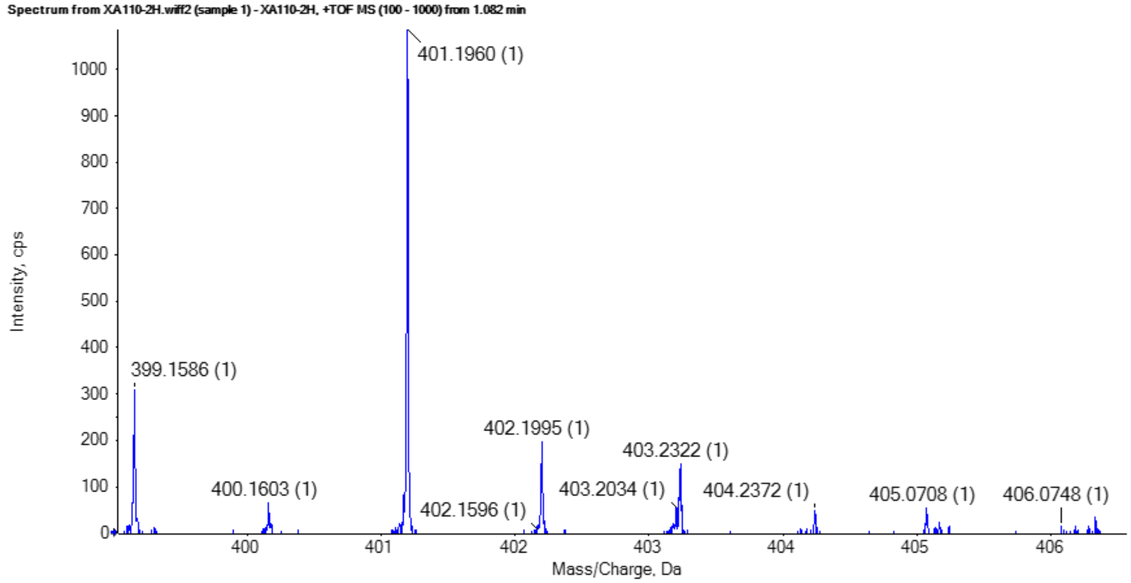


**Formula Calculator Results**

| Measured m/z | Cal m/z | Error(mmu) | Error(ppm) | Ion Formula | Ion |
|---|---|---|---|---|---|
| 401.1960 | 401.1959 | 0.1 | 0.3 | $C_{23}H_{29}O_6$ | $[M+H]^+$ |

HRESIMS spectrum of compound **10i**

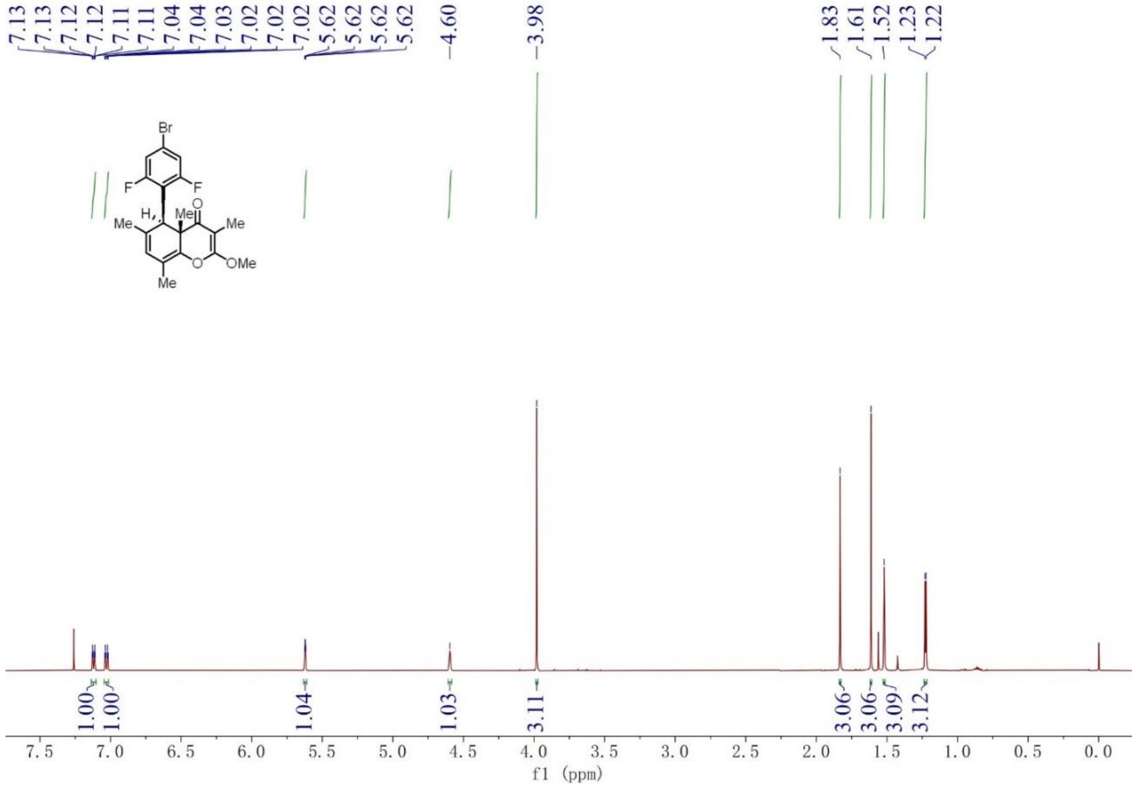


$^{1}$H NMR spectrum of compound **10j** (600MHz, $CDCl_3$)

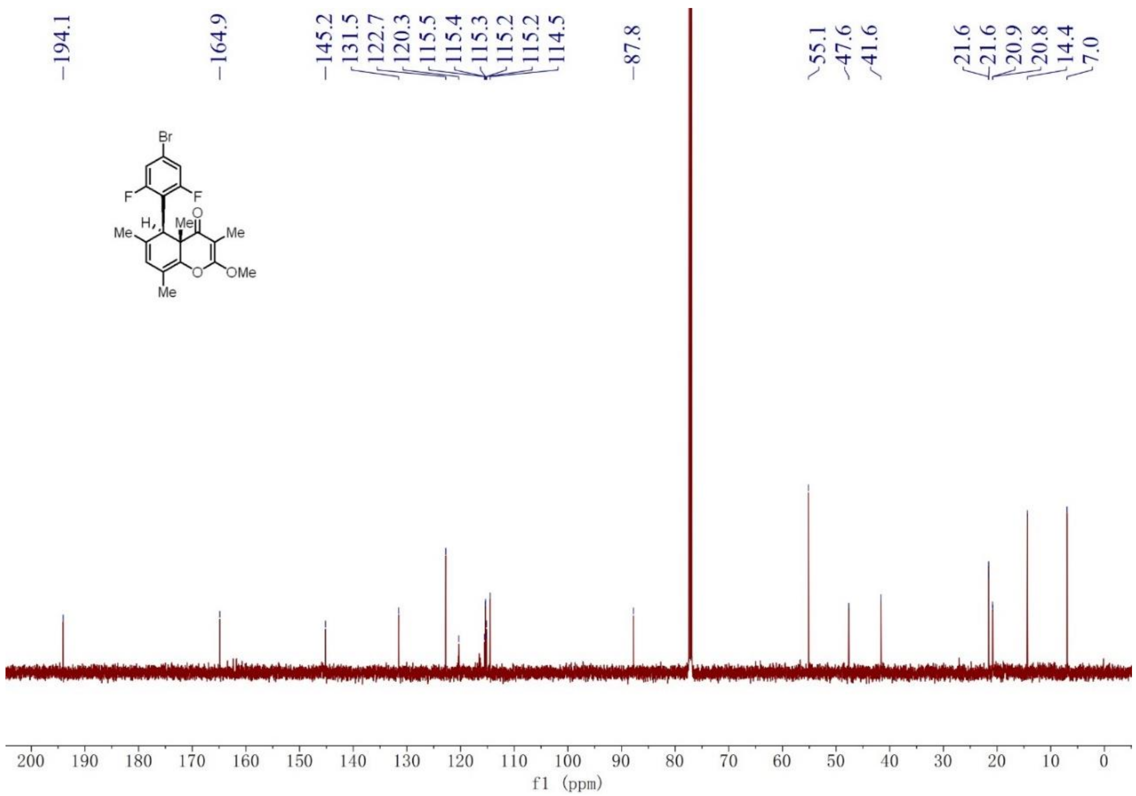


$^{13}$C NMR spectrum of compound **10j** (151MHz, $CDCl_3$)

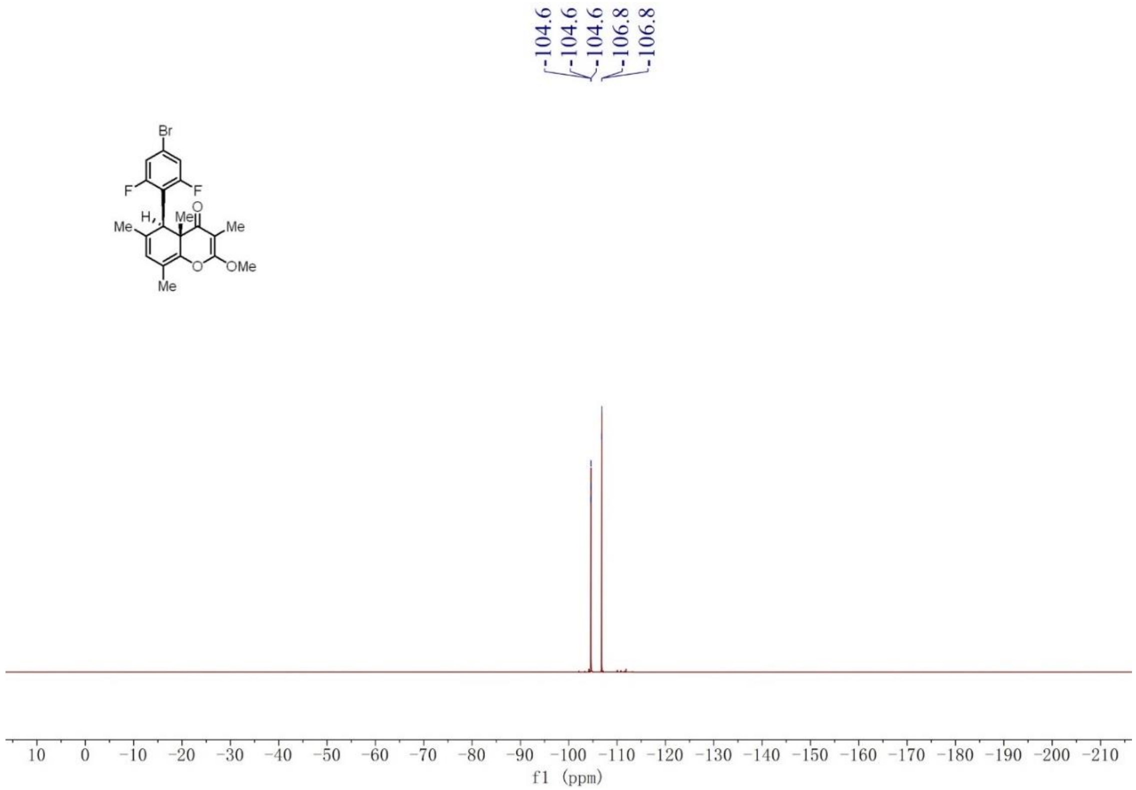


$^{19}$F NMR spectrum of compound **10j** (565MHz, $CDCl_3$)

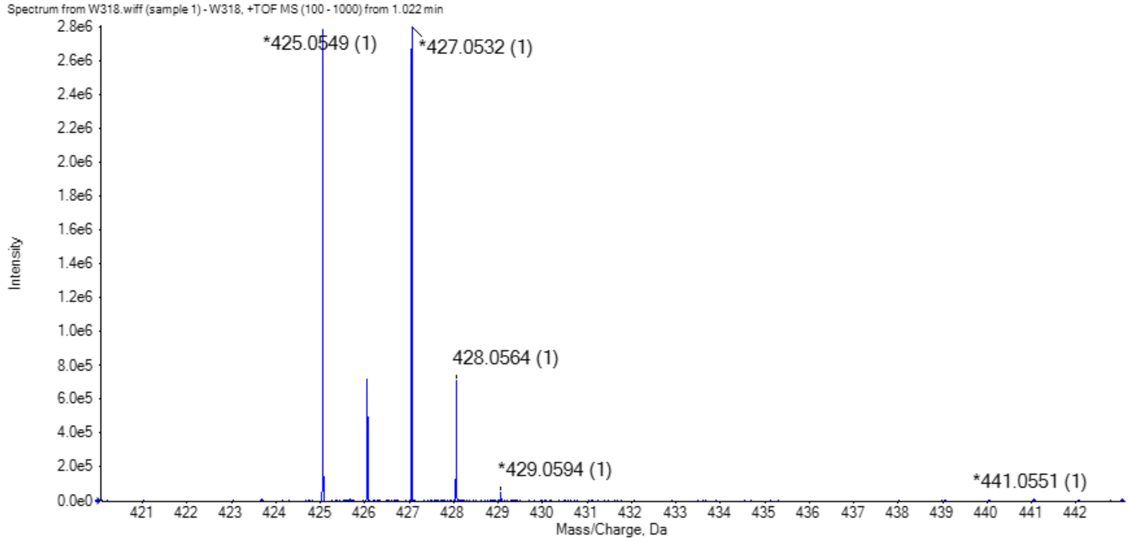


**Formula Calculator Results**

| Measured m/z | Cal m/z | Error(mmu) | Error(ppm) | Ion Formula | Ion |
|---|---|---|---|---|---|
| 425.05494 | 425.05584 | -0.9 | -2.1 | C20H20BrF2O3 | $[M+H]^+$ |

HRESIMS spectrum of compound **10j**

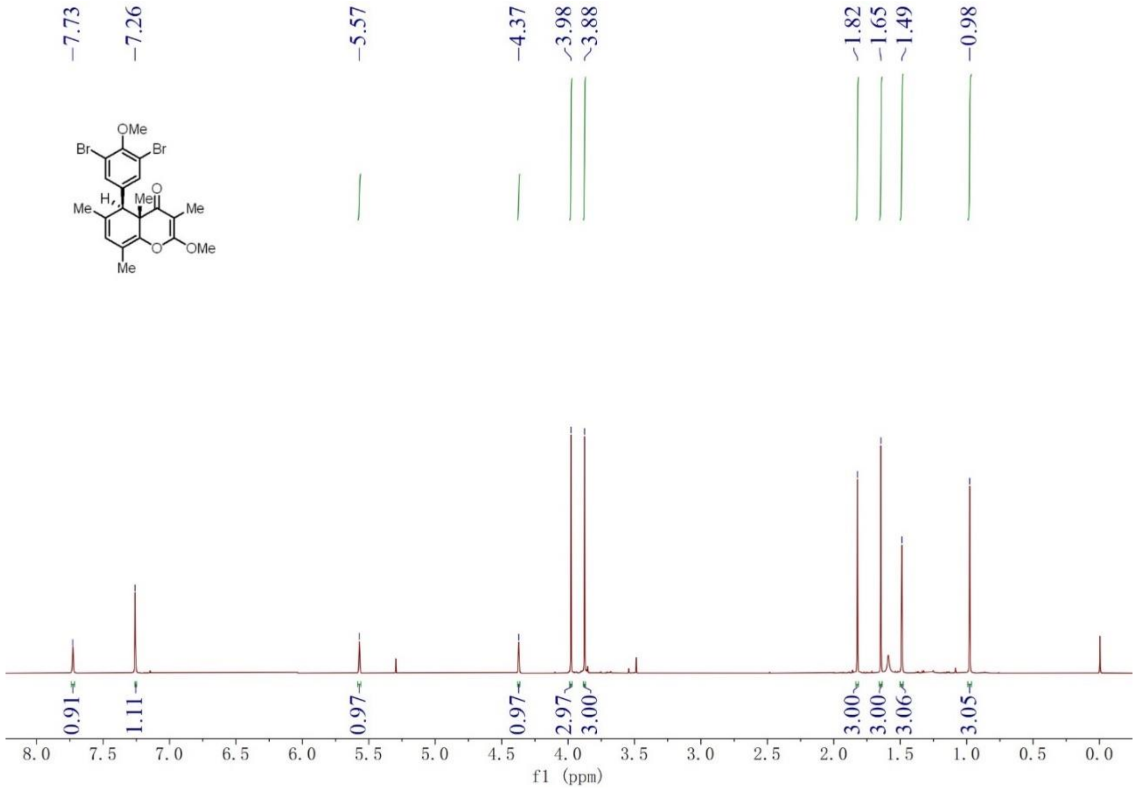


$^1$H NMR spectrum of compound **10k** (600MHz, $CDCl_3$)

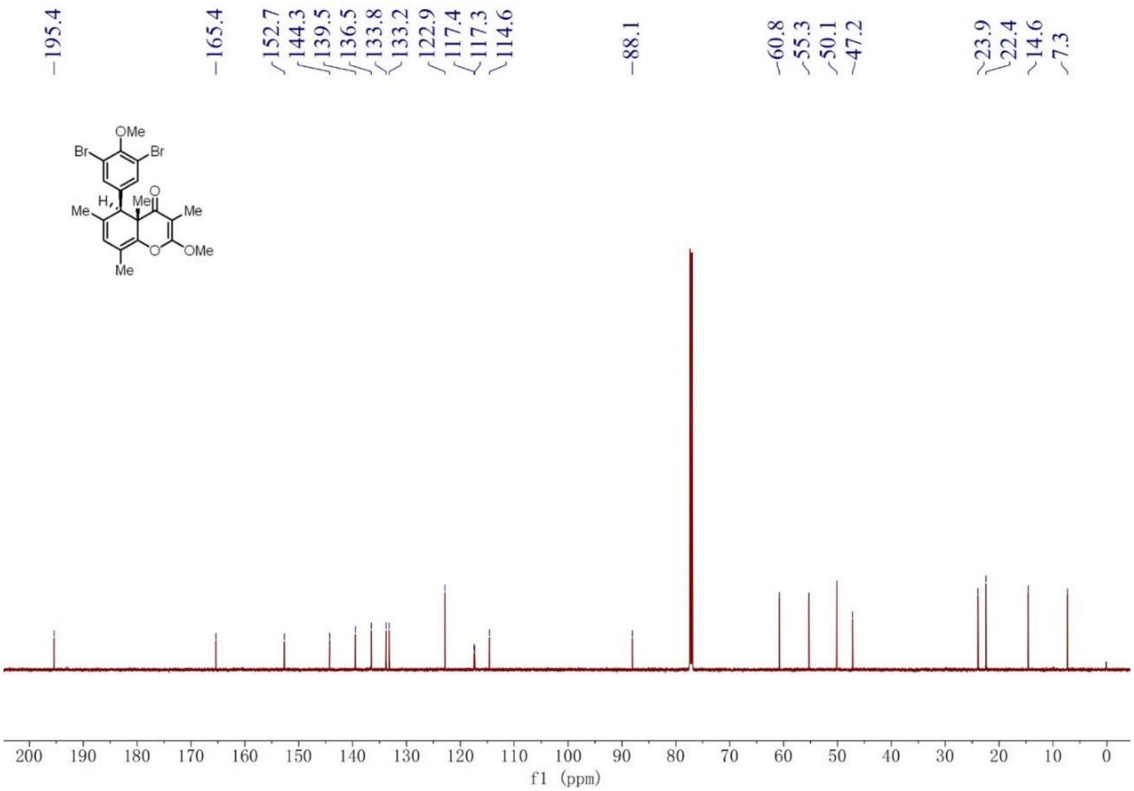


$^{13}$C NMR spectrum of compound **10k** (151MHz, $CDCl_3$)

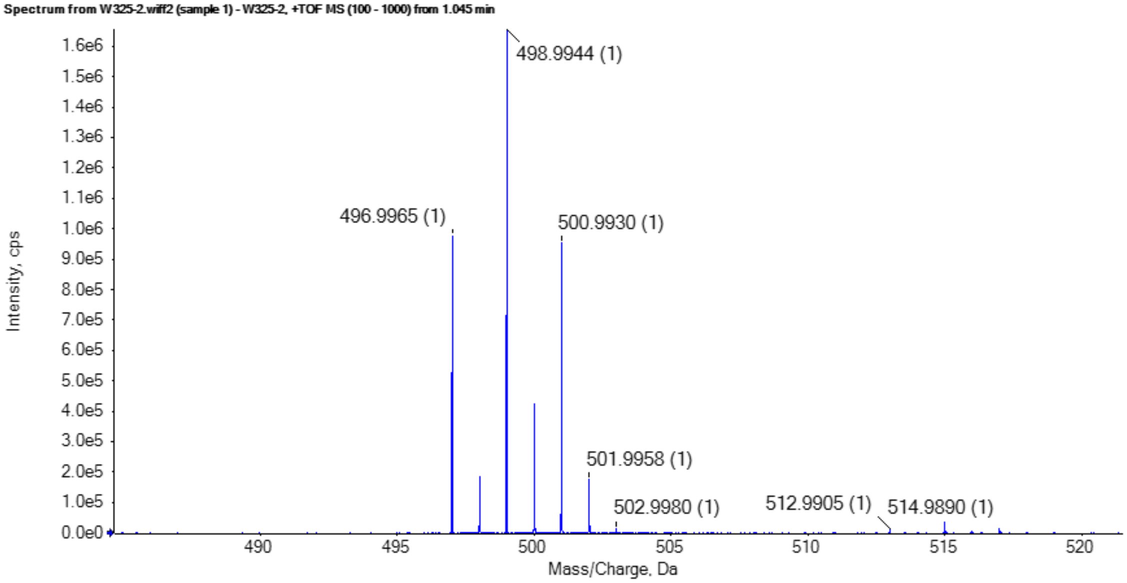


## Formula Calculator Results

| Measured m/z | Cal m/z | Error(mmu) | Error(ppm) | Ion Formula | Ion |
|---|---|---|---|---|---|
| 496.9965 | 496.9958 | 0.7 | 1.4 | C21H23Br2O4 | $[M+H]^+$ |

HRESIMS spectrum of compound **10k**

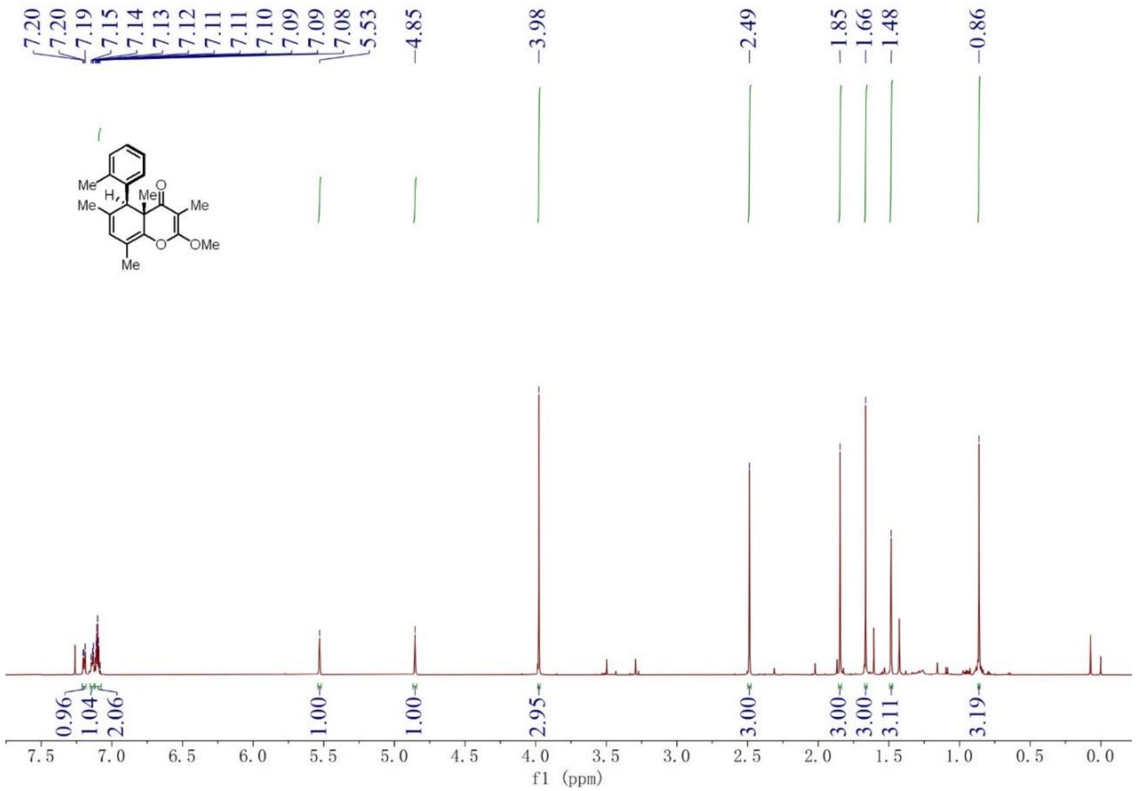


$^{1}H$ NMR spectrum of compound **10l** (600MHz, $CDCl_3$)

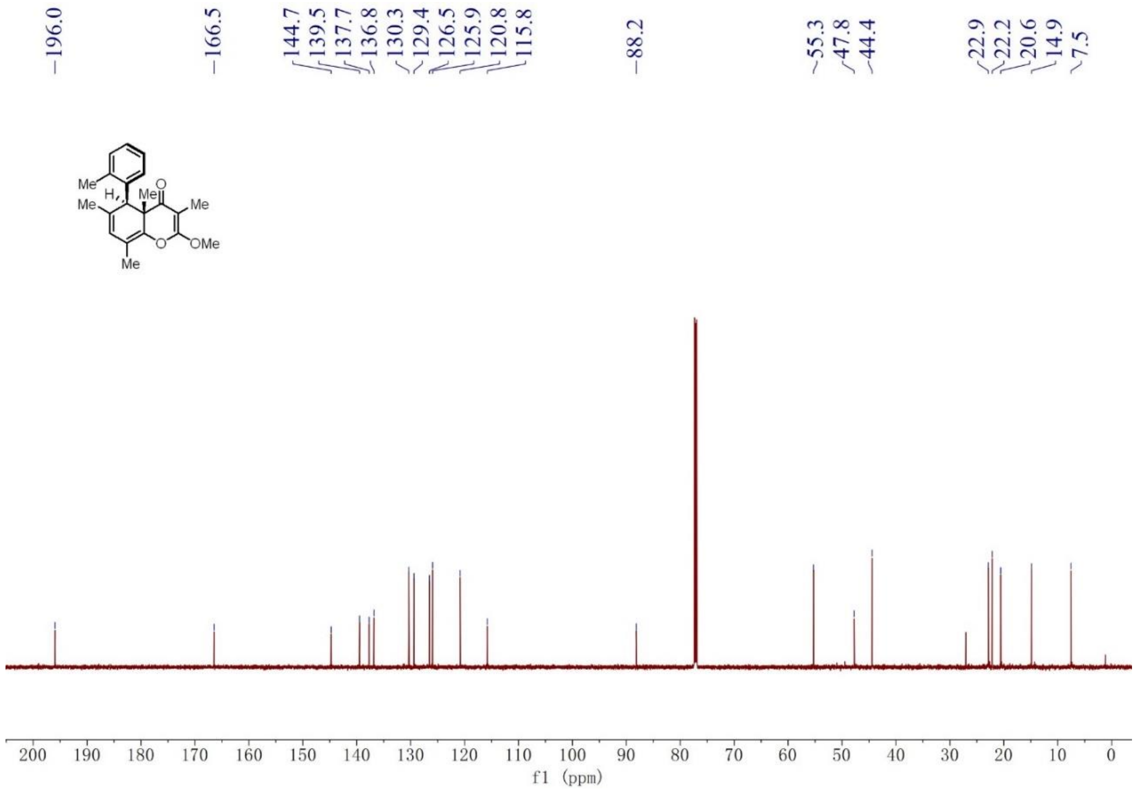


$^{13}C$ NMR spectrum of compound **10l** (151MHz, $CDCl_3$)

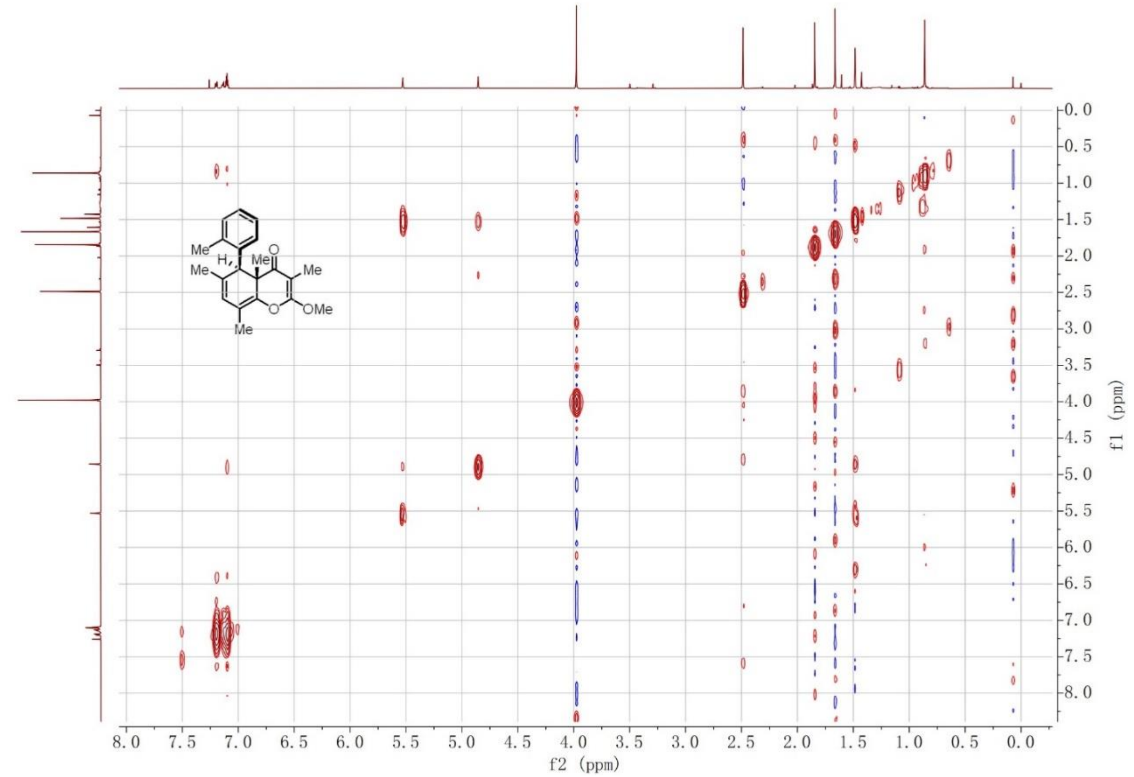


$^{1}$H-$^{1}$H COSY spectrum of compound **10l** (600 MHz, $CDCl_3$)

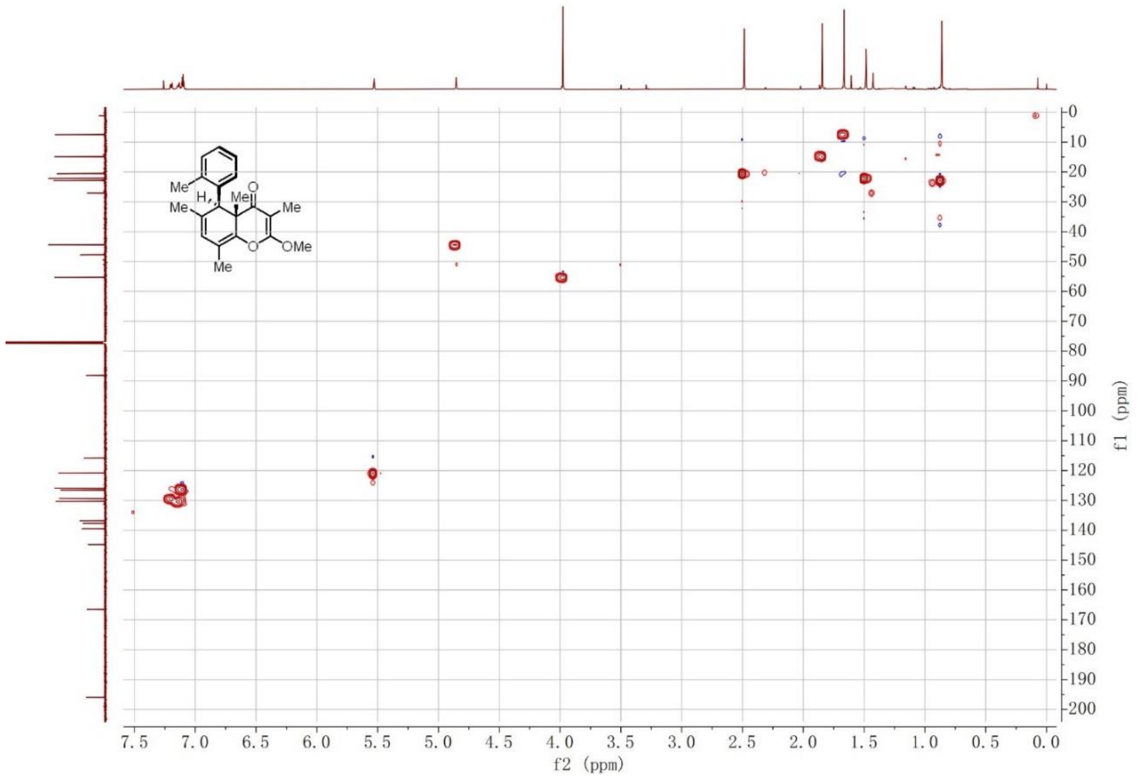


HSQC spectrum of compound **10l** (600 MHz, $CDCl_3$)

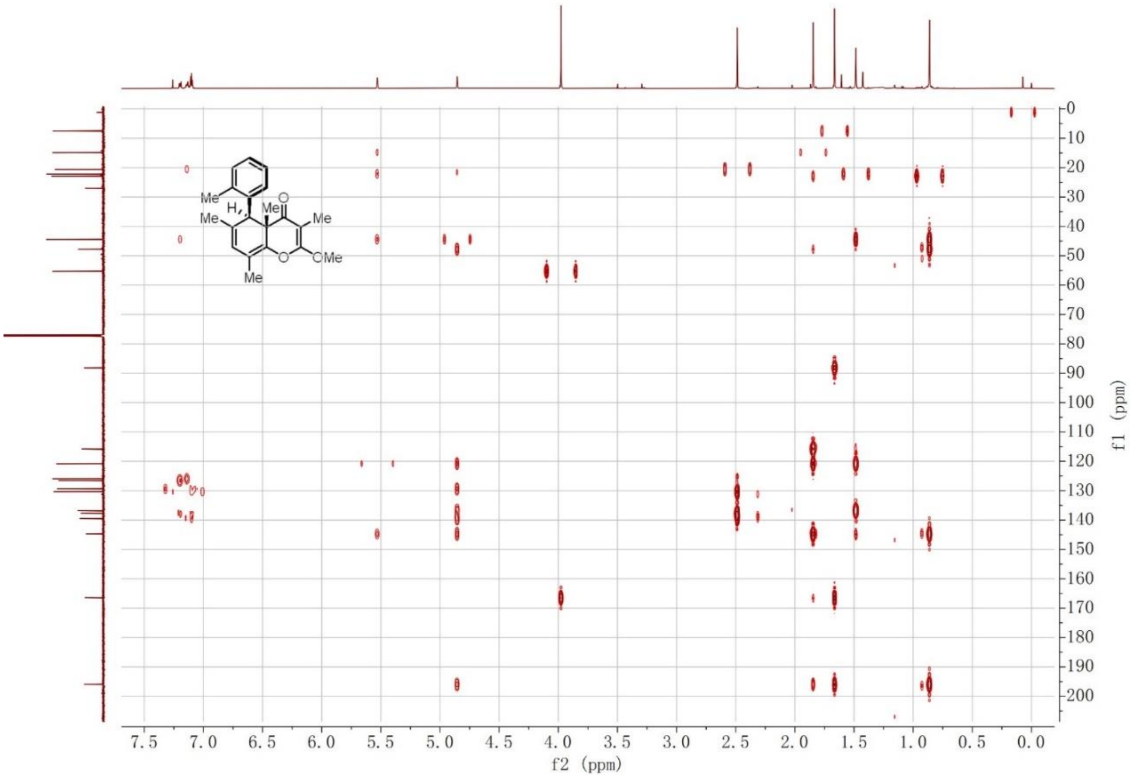

HMBC spectrum of compound **10l** (600 MHz, $CDCl_3$)

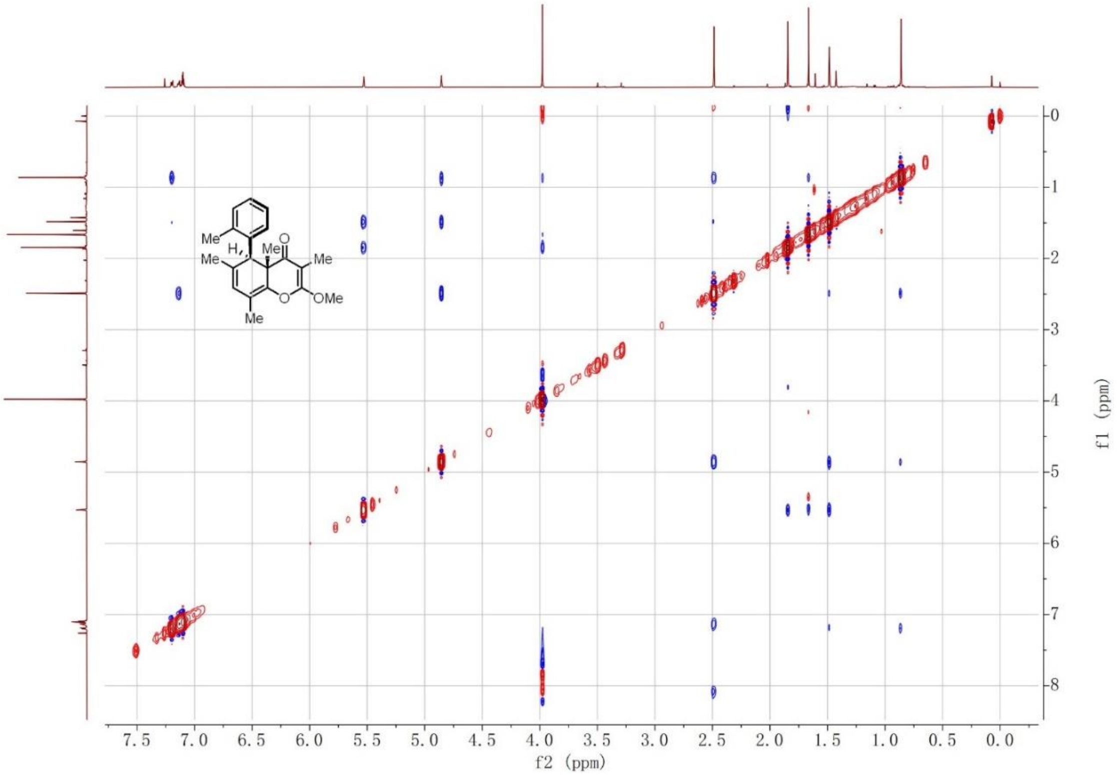

NOESY spectrum of compound **10l** (600 MHz, $CDCl_3$)

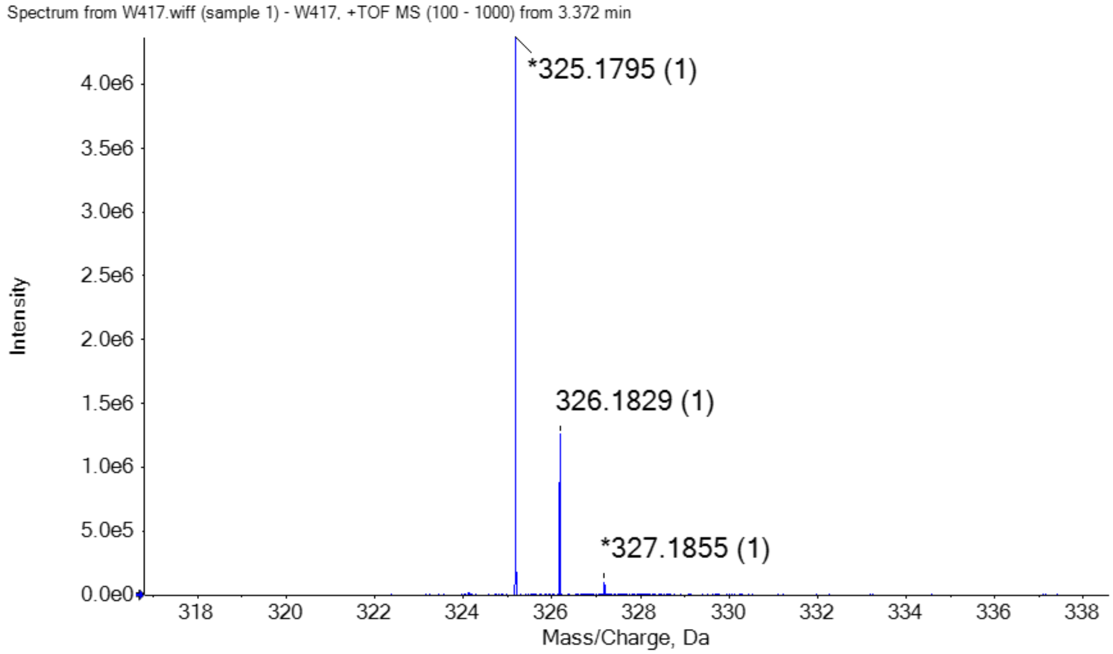


**Formula Calculator Results**

| Measured m/z | Cal m/z | Error(mmu) | Error(ppm) | Ion Formula | Ion |
|---|---|---|---|---|---|
| 325.1795 | 325.1798 | -0.3 | -1.0 | C21H25O3 | $[M+H]^+$ |

HRESIMS spectrum of compound **10l**

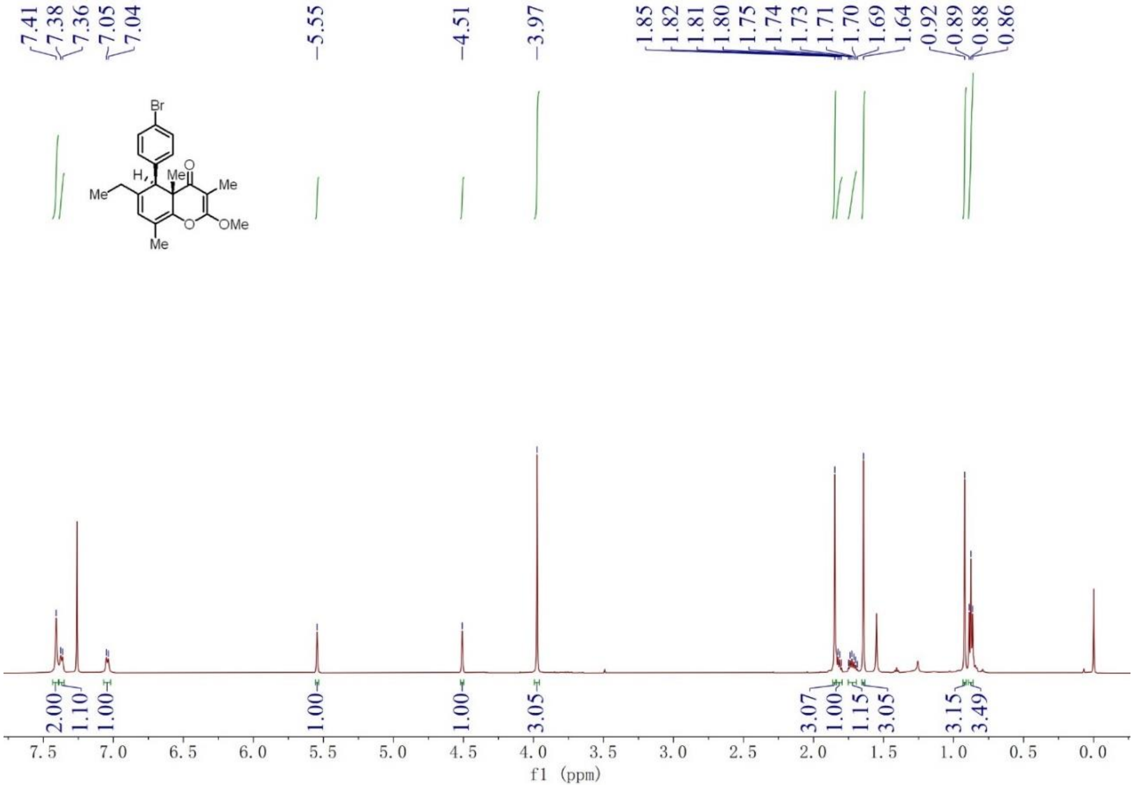


$^1$H NMR spectrum of compound **10m** (600MHz, $CDCl_3$)

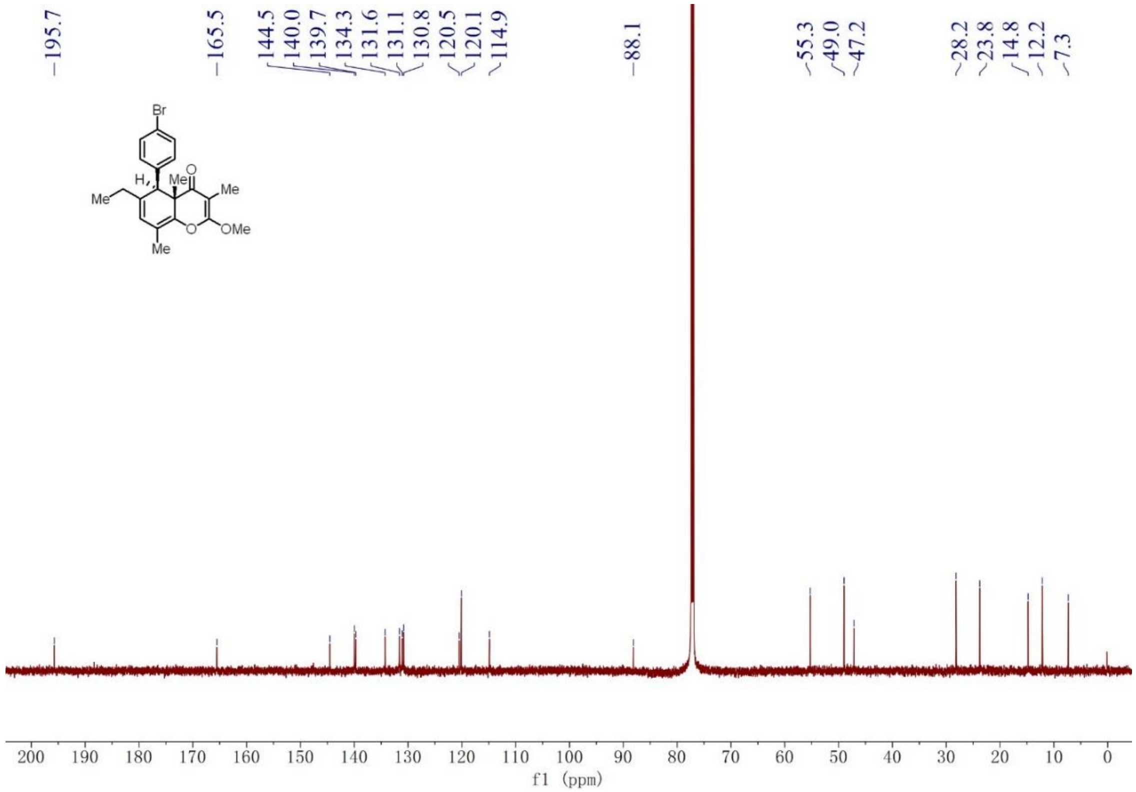


$^{13}C$ NMR spectrum of compound **10m** (151MHz, $CDCl_3$)

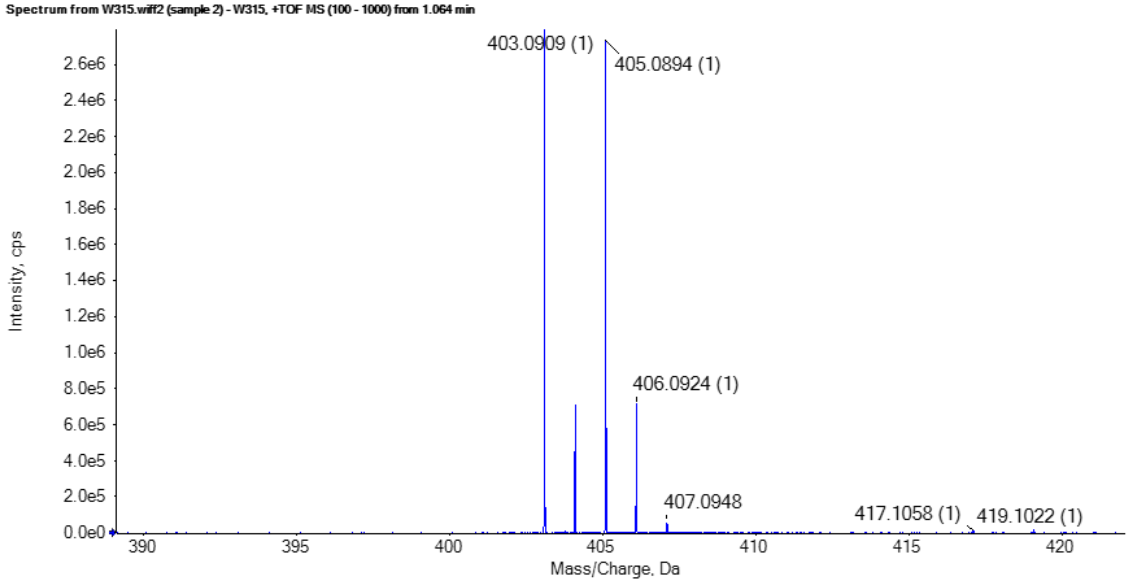


**Formula Calculator Results**

| Measured m/z | Cal m/z | Error(mmu) | Error(ppm) | Ion Formula | Ion |
|---|---|---|---|---|---|
| 403.0909 | 403.0903 | 0.6 | 1.5 | C21H24BrO3 | $[M+H]^+$ |

HRESIMS spectrum of compound **10m**

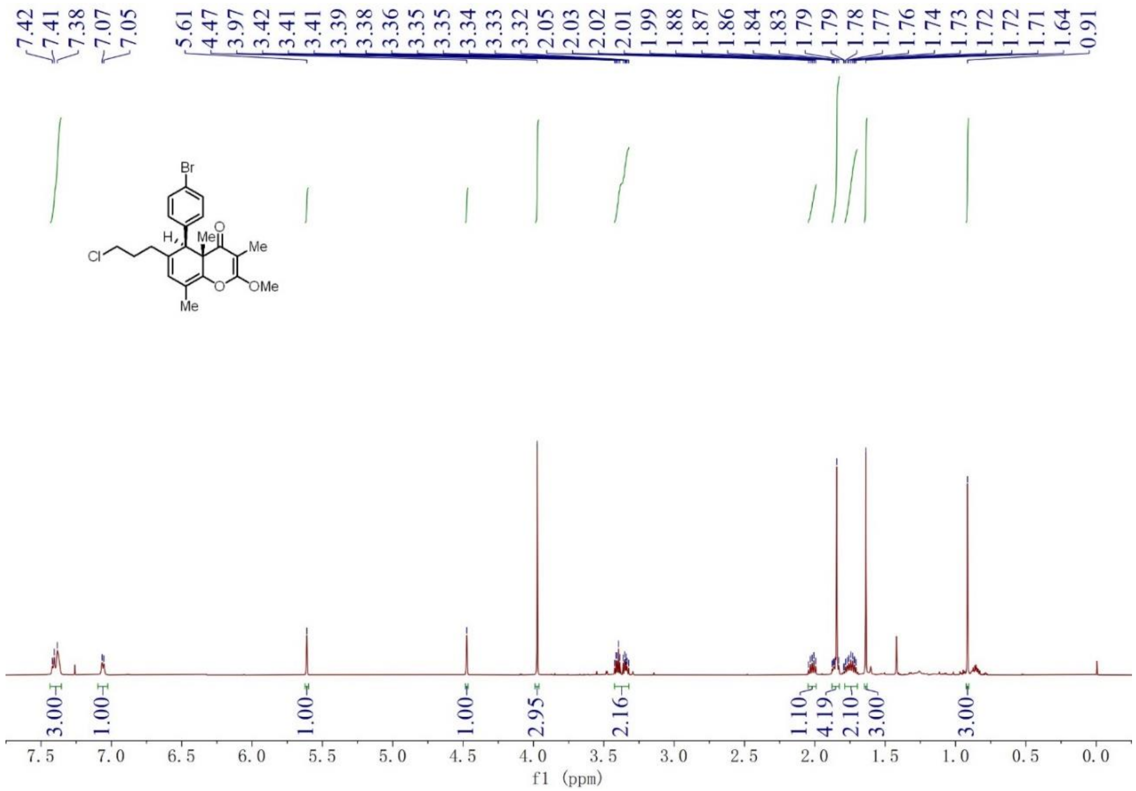


$^{1}H$ NMR spectrum of compound **10n** (600MHz, $CDCl_3$)

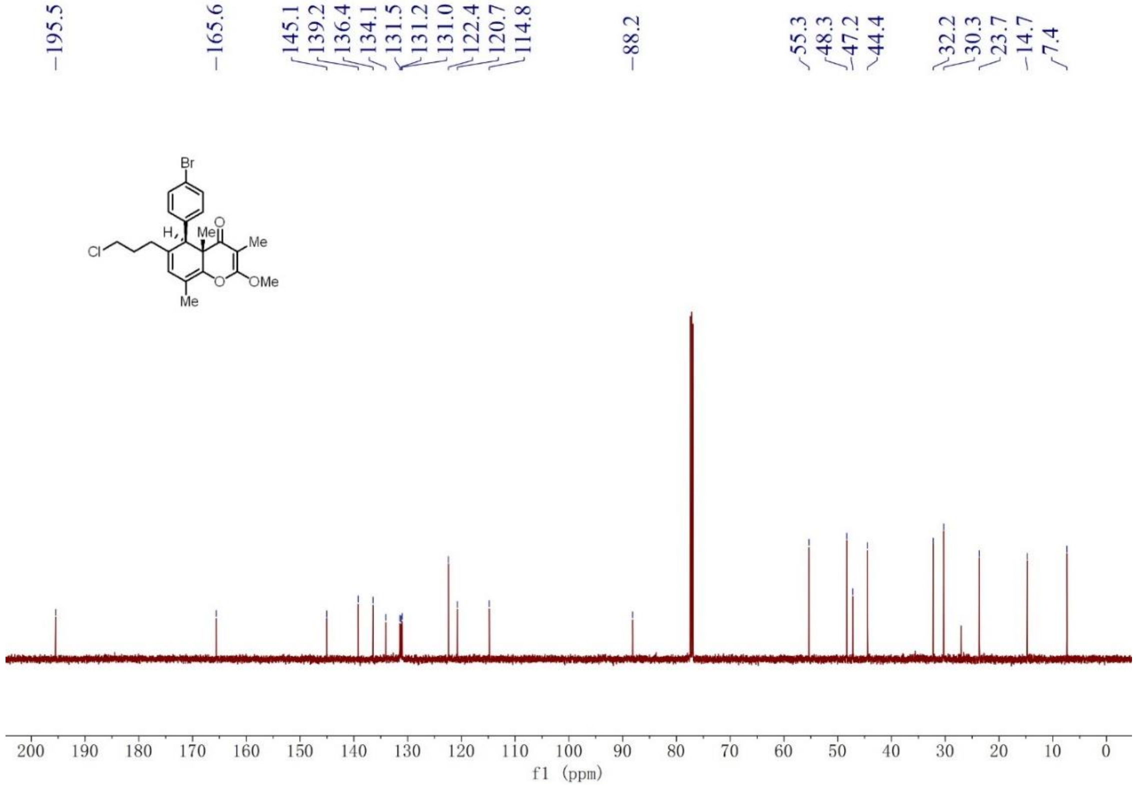


$^{13}C$ NMR spectrum of compound **10n** (151MHz, $CDCl_3$)

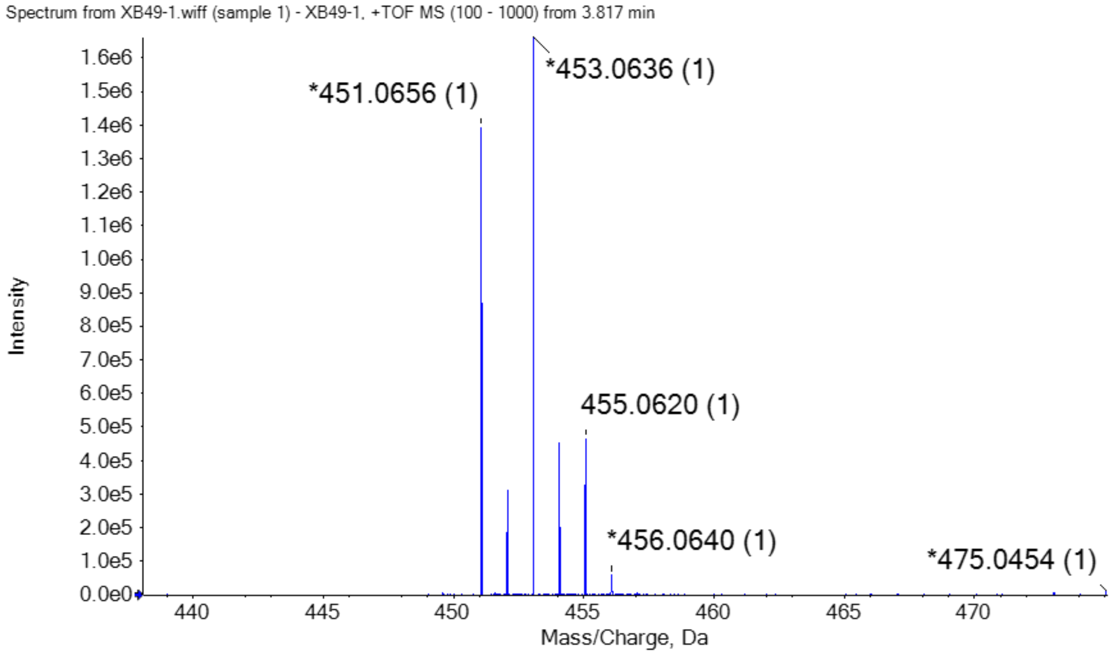


**Formula Calculator Results**

| Measured m/z | Cal m/z | Error(mmu) | Error(ppm) | Ion Formula | Ion |
|---|---|---|---|---|---|
| 451.0656 | 451.0670 | -1.4 | -3.1 | C22H25BrClO3 | $[M+H]^+$ |

HRESIMS spectrum of compound **10n**

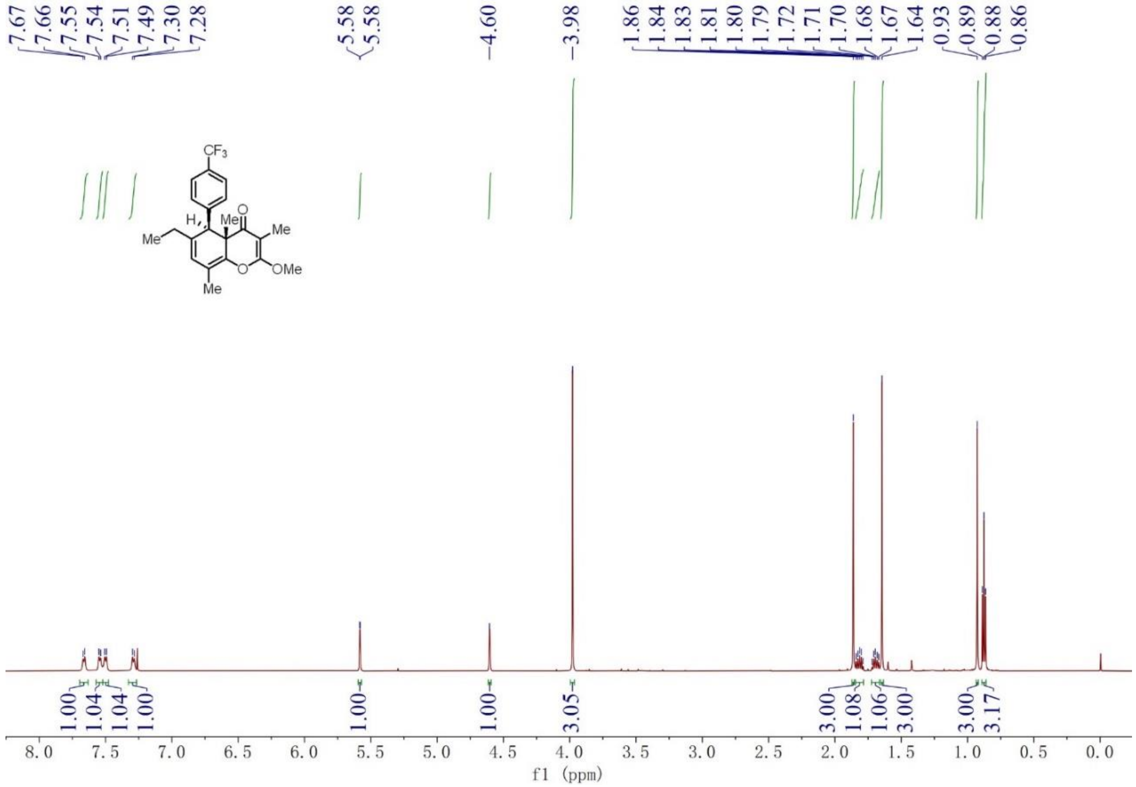


$^1$H NMR spectrum of compound **10o** (600MHz, $CDCl_3$)

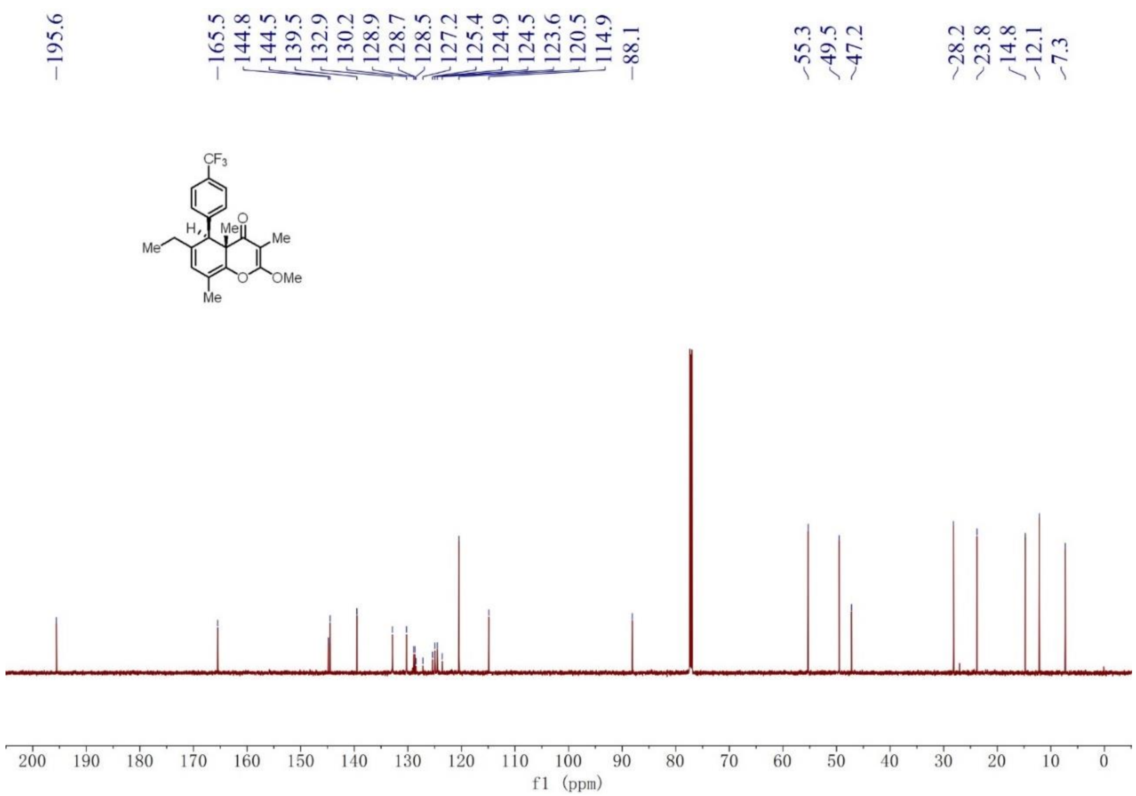


$^{13}C$ NMR spectrum of compound **10o** (151MHz, $CDCl_3$)

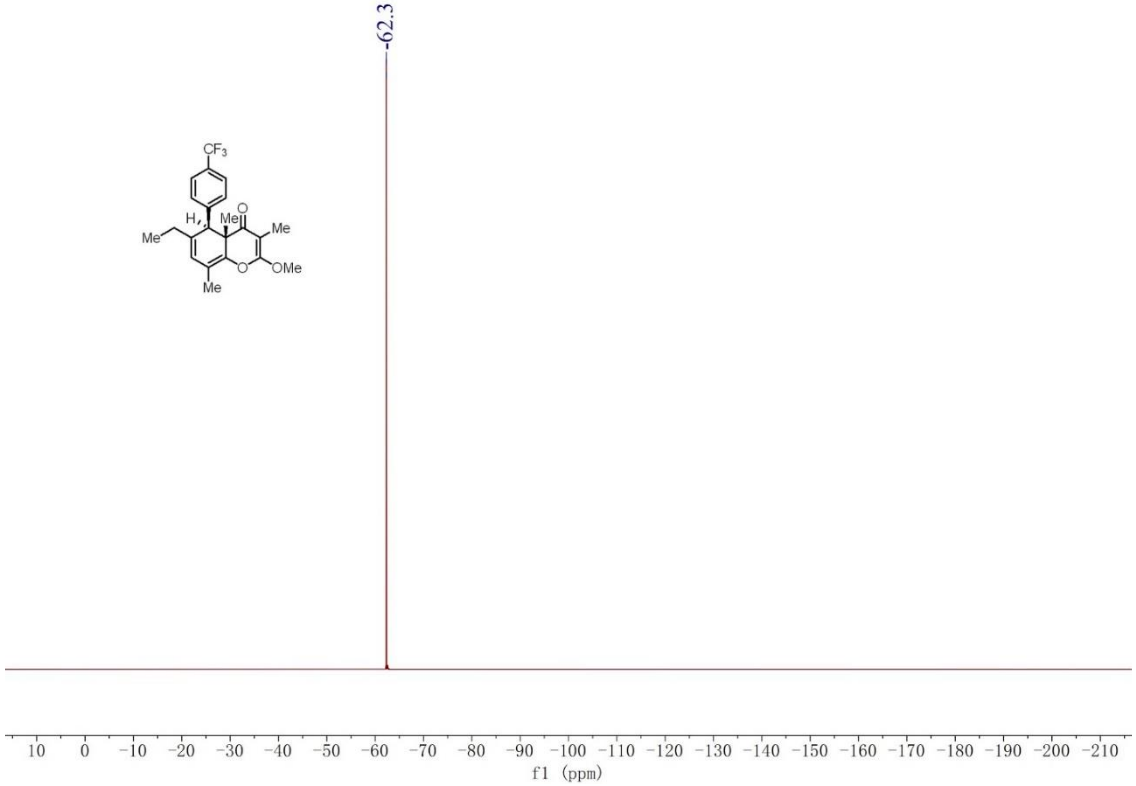


$^{19}F$ NMR spectrum of compound **10o** (565MHz, $CDCl_3$)

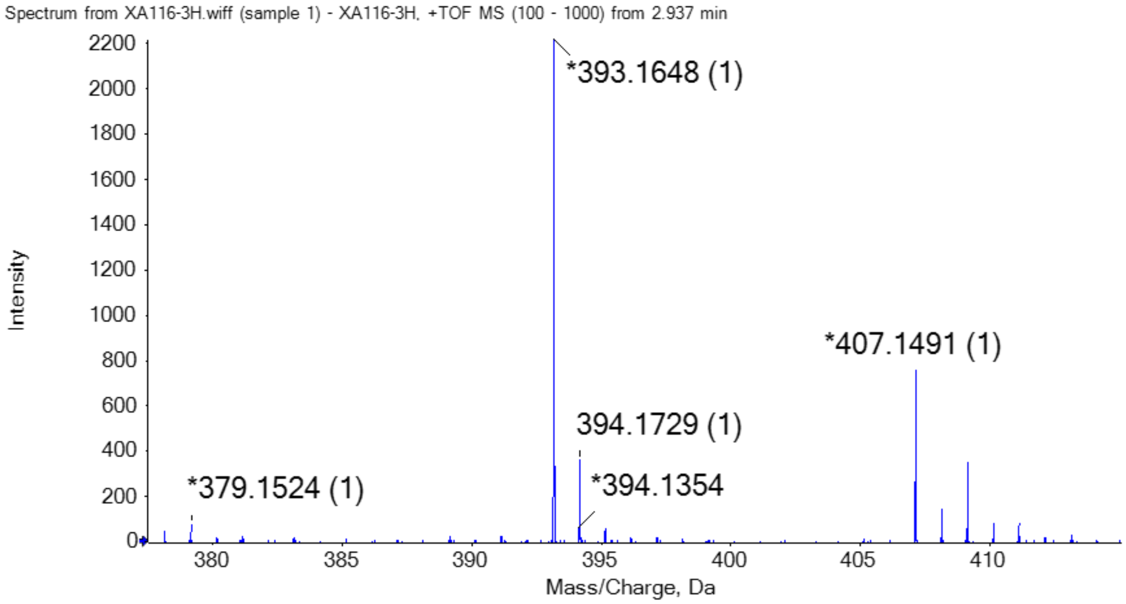


**Formula Calculator Results**

| Measured m/z | Cal m/z | Error(mmu) | Error(ppm) | Ion Formula | Ion |
|---|---|---|---|---|---|
| 393.1648 | 393.1672 | -2.4 | -6.1 | C22H24F3O3 | $[M+H]^+$ |

HRESIMS spectrum of compound **10o**

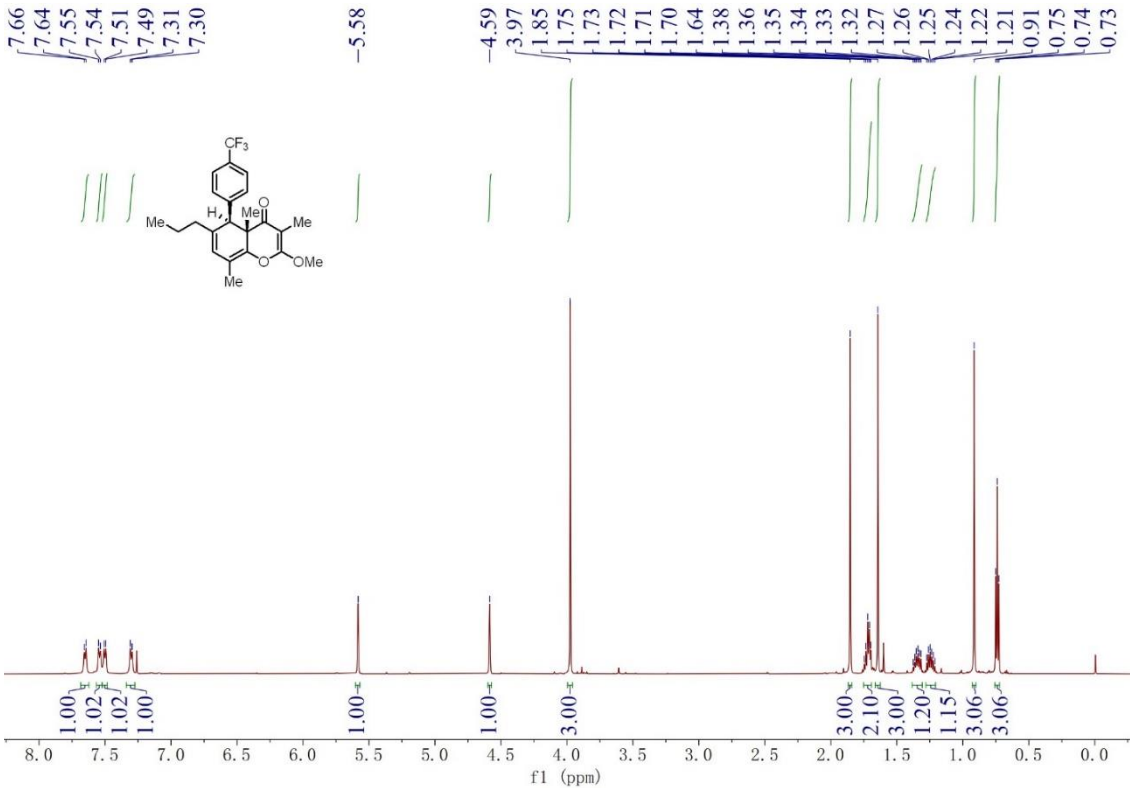


$^{1}$H NMR spectrum of compound **10p** (600MHz, $CDCl_3$)

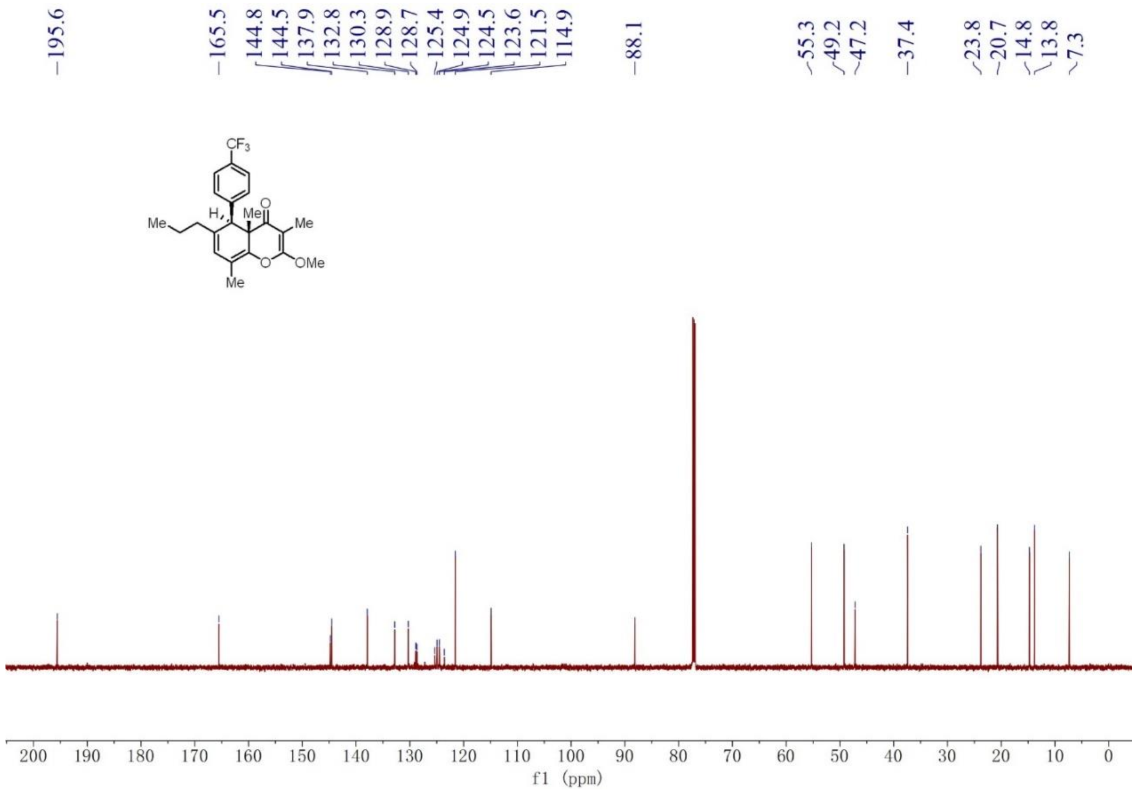


$^{13}$C NMR spectrum of compound **10p** (151MHz, $CDCl_3$)

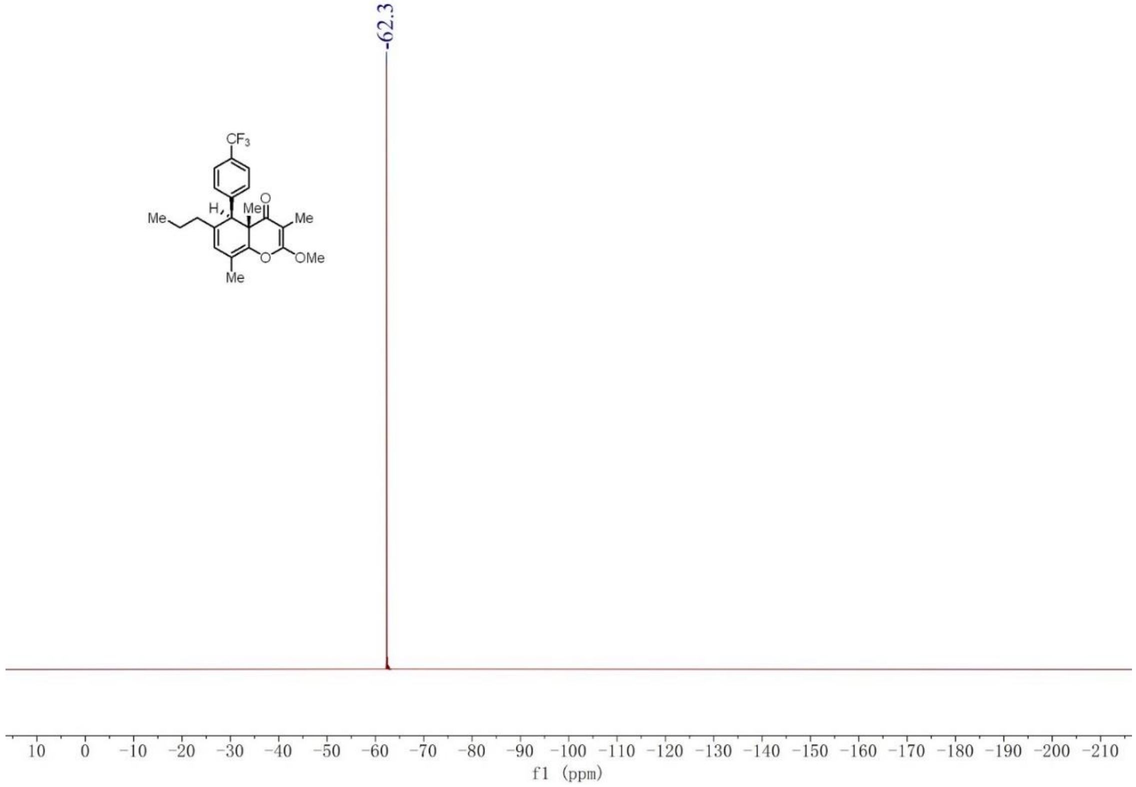


$^{19}$F NMR spectrum of compound **10p** (565MHz, $CDCl_3$)

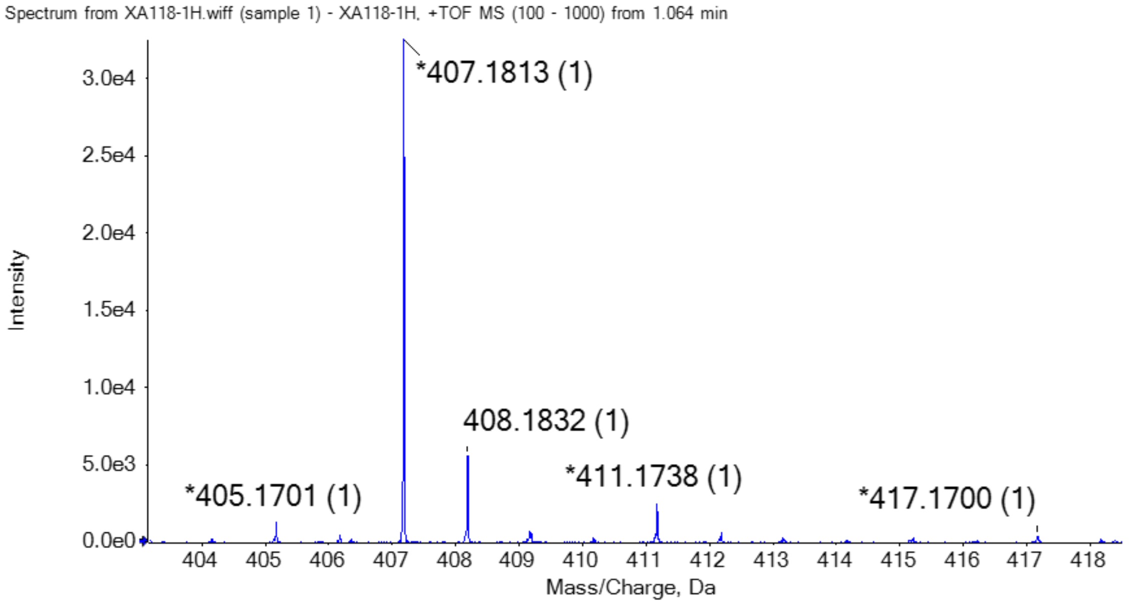


**Formula Calculator Results**

| Measured m/z | Cal m/z | Error(mmu) | Error(ppm) | Ion Formula | Ion |
|---|---|---|---|---|---|
| 407.1813 | 407.1828 | -1.6 | -3.8 | C23H26F3O3 | $[M+H]^+$ |

HRESIMS spectrum of compound **10p**

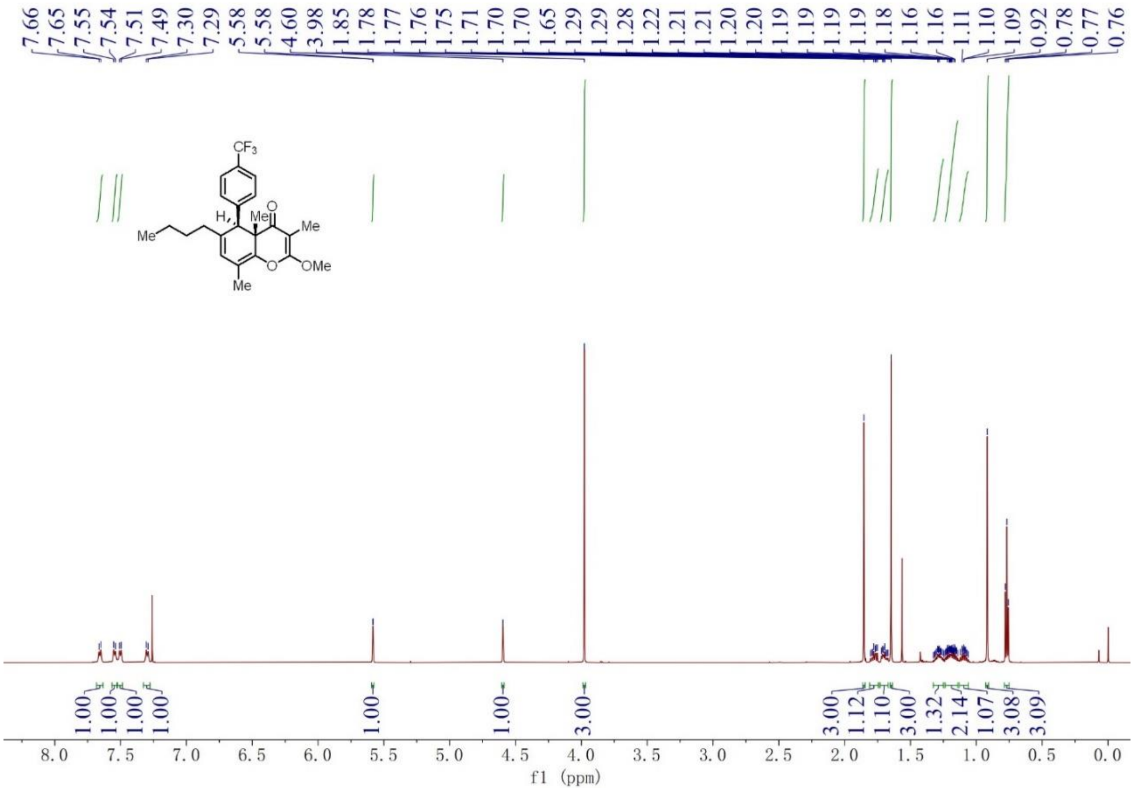

$^1$H NMR spectrum of compound **10q** (600MHz, $CDCl_3$)

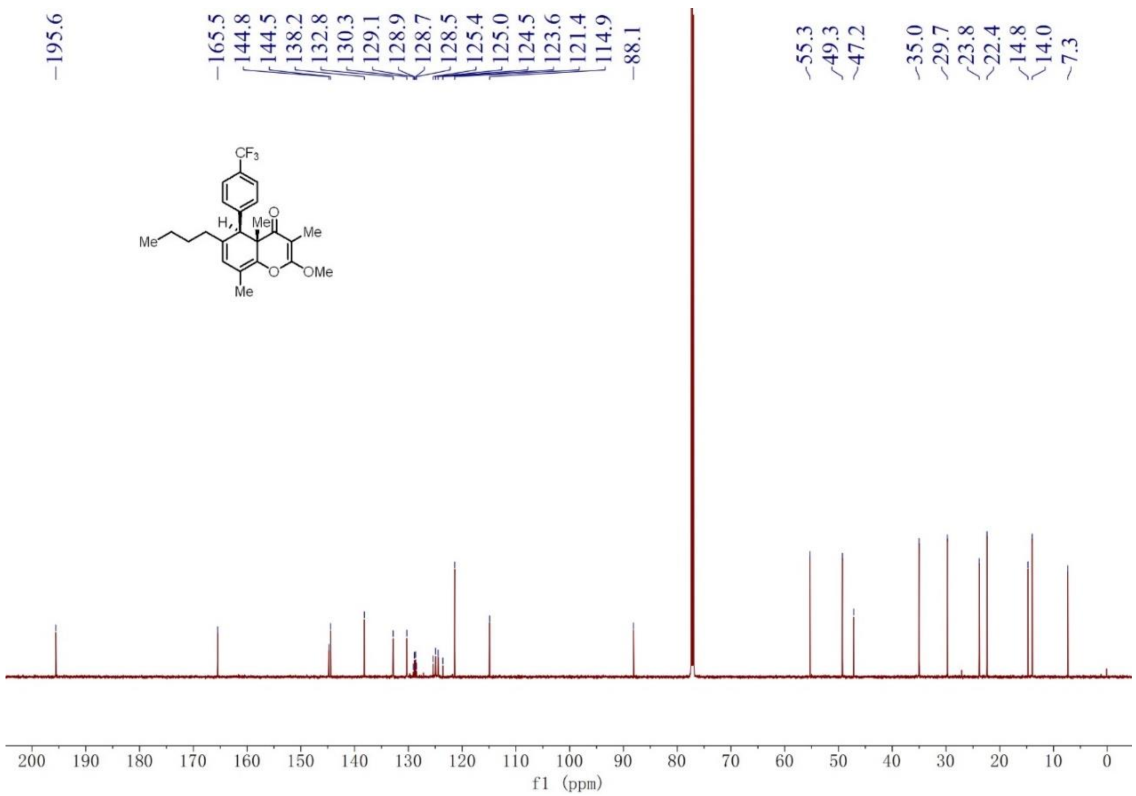


$^{13}C$ NMR spectrum of compound **10q** (151MHz, $CDCl_3$)

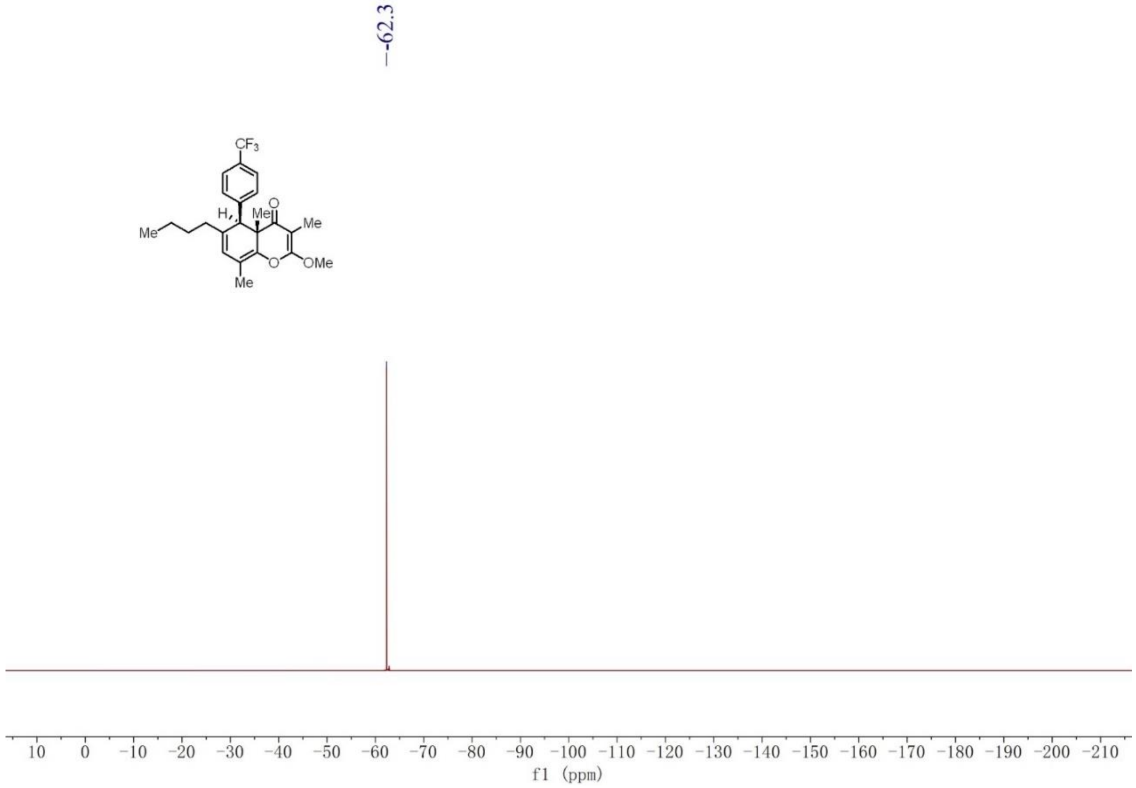


$^{19}F$ NMR spectrum of compound **10q** (565MHz, $CDCl_3$)

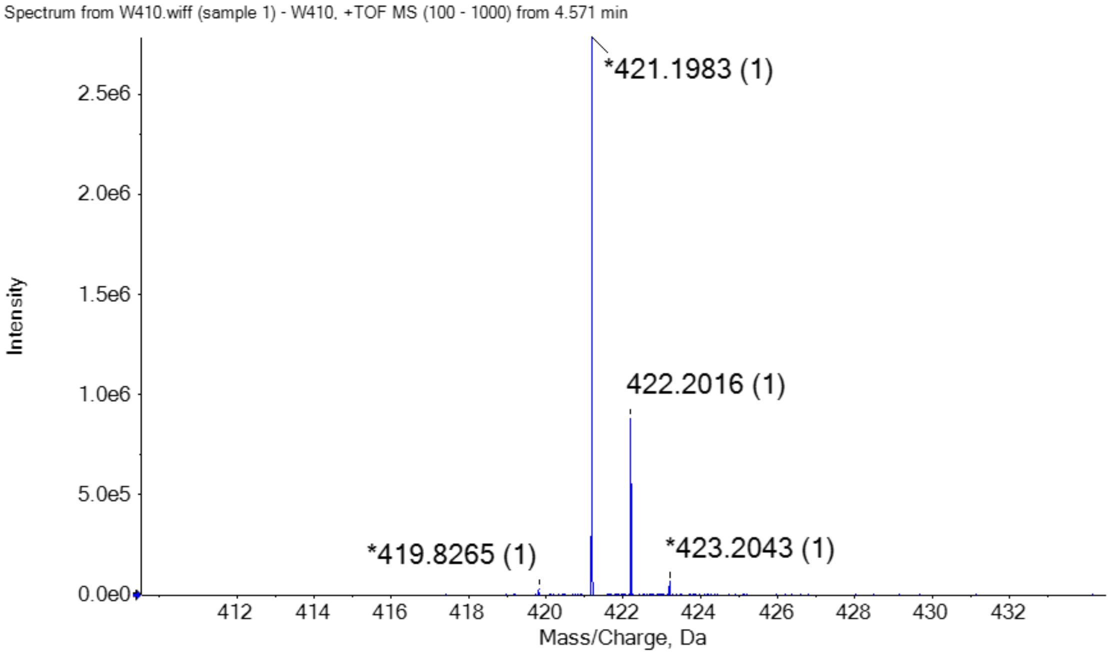


**Formula Calculator Results**

| Measured m/z | Cal m/z | Error(mmu) | Error(ppm) | Ion Formula | Ion |
|---|---|---|---|---|---|
| 421.1983 | 421.1985 | -0.2 | -0.5 | C24H28F3O3 | $[M+H]^+$ |

HRESIMS spectrum of compound **10q**

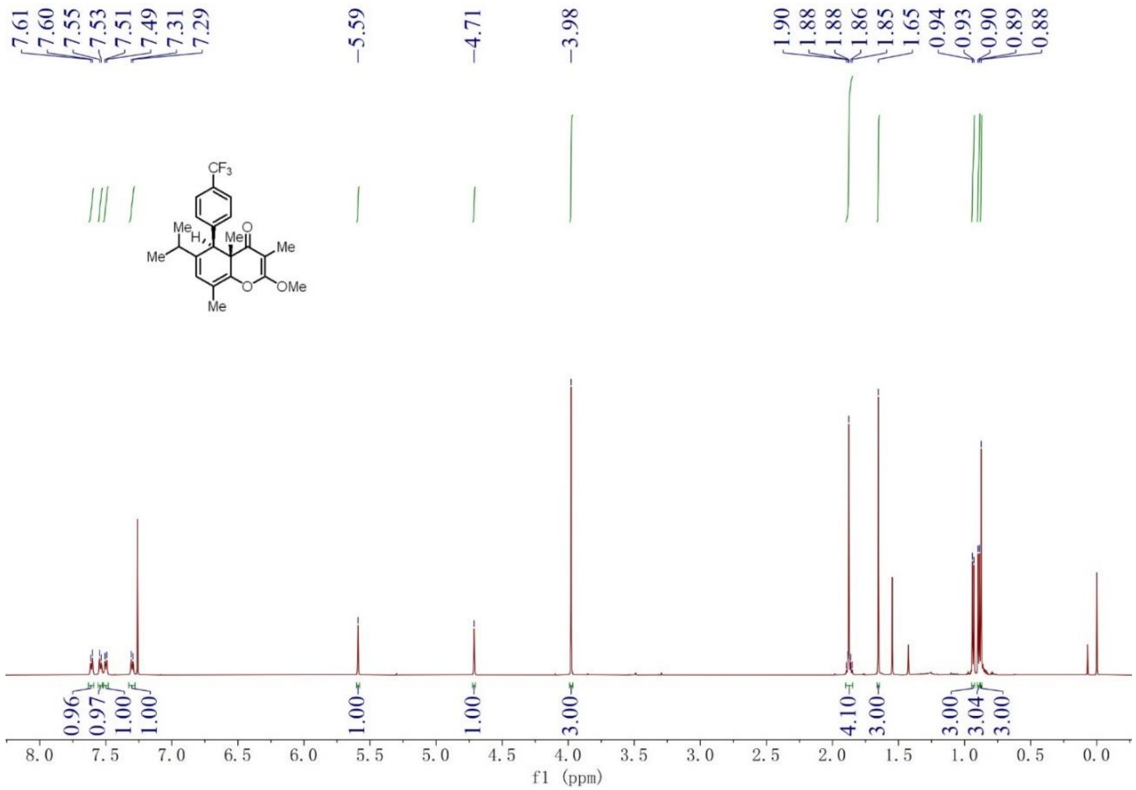


$^1$H NMR spectrum of compound **10r** (600MHz, $CDCl_3$)

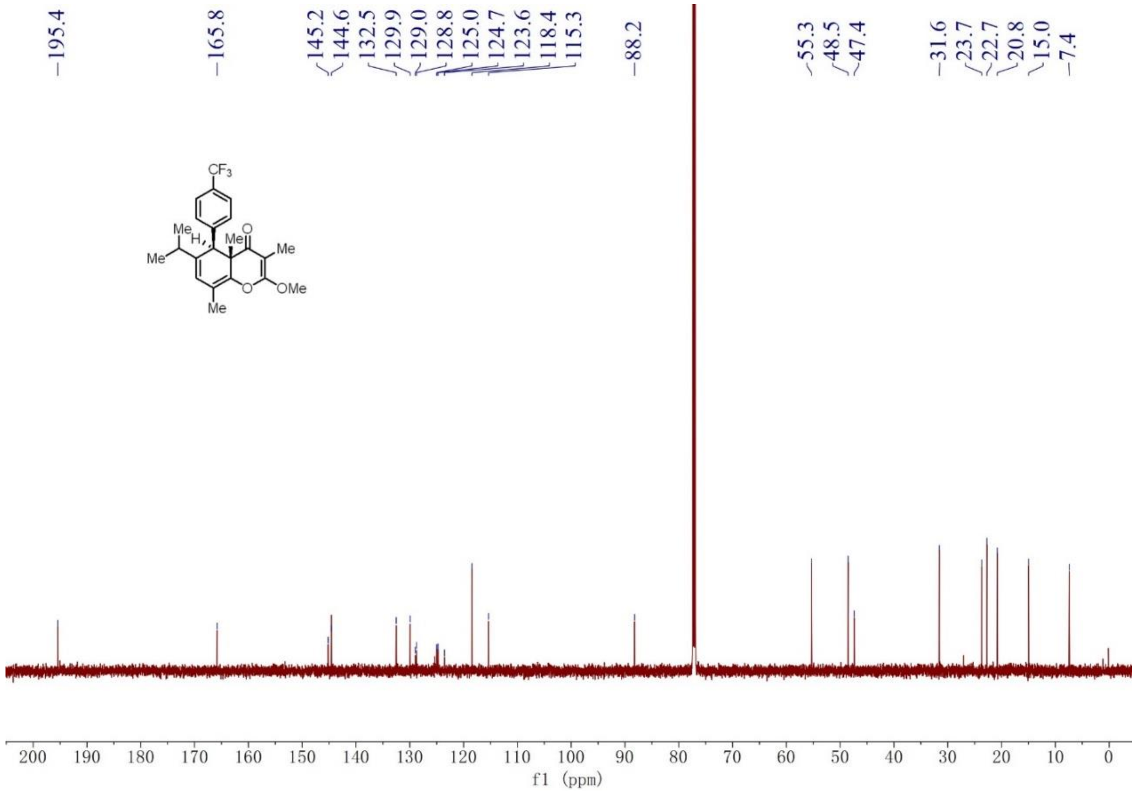


$^{13}C$ NMR spectrum of compound **10r** (151MHz, $CDCl_3$)

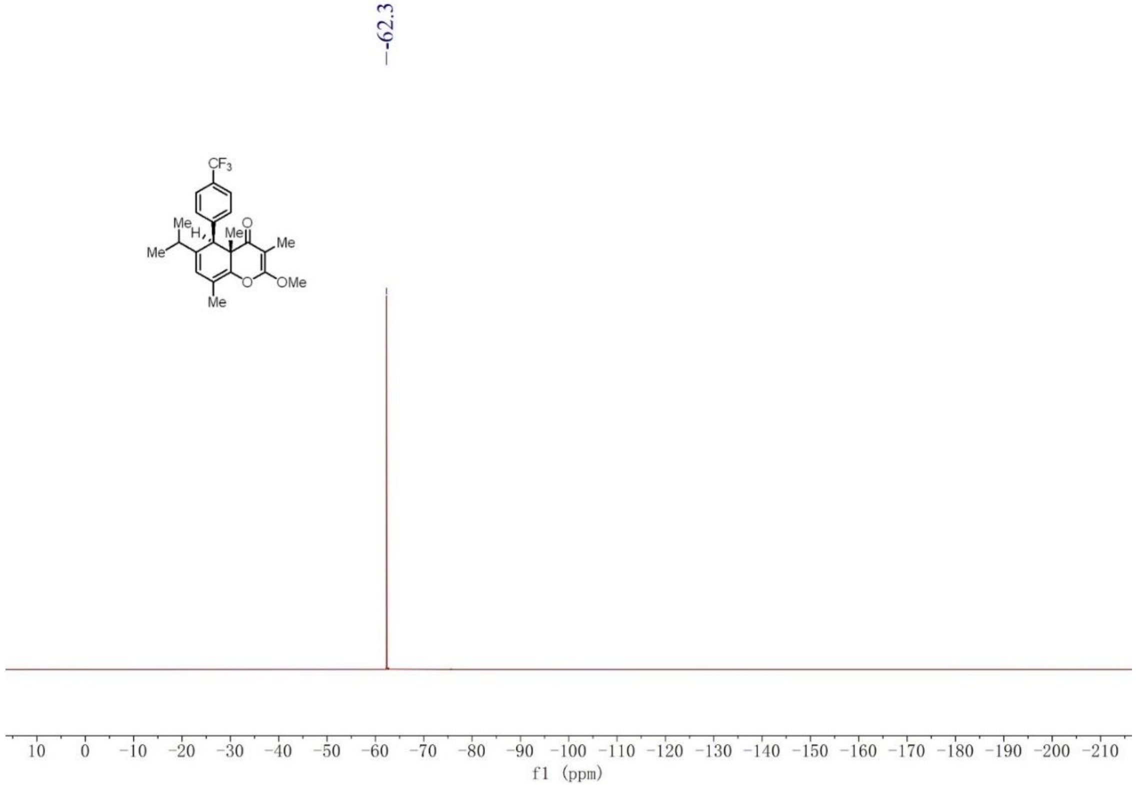


$^{19}F$ NMR spectrum of compound **10r** (565MHz, $CDCl_3$)

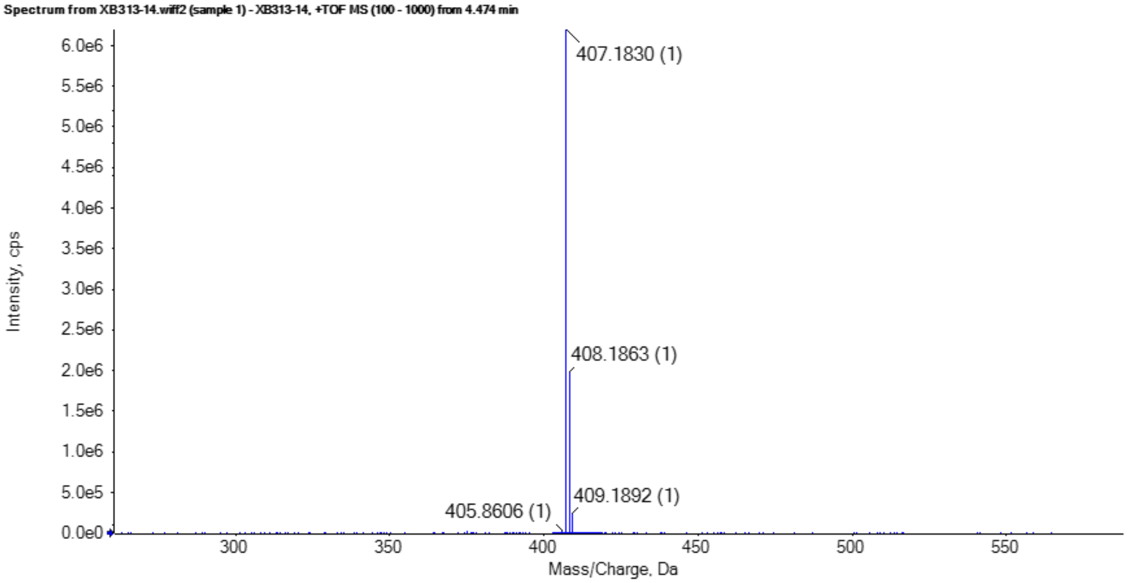


**Formula Calculator Results**

| Measured m/z | Cal m/z | Error(mmu) | Error(ppm) | Ion Formula | Ion |
|---|---|---|---|---|---|
| 407.1830 | 407.1828 | 0.2 | 0.4 | C23H26F3O3 | $[M+H]^+$ |

HRESIMS spectrum of compound **10r**

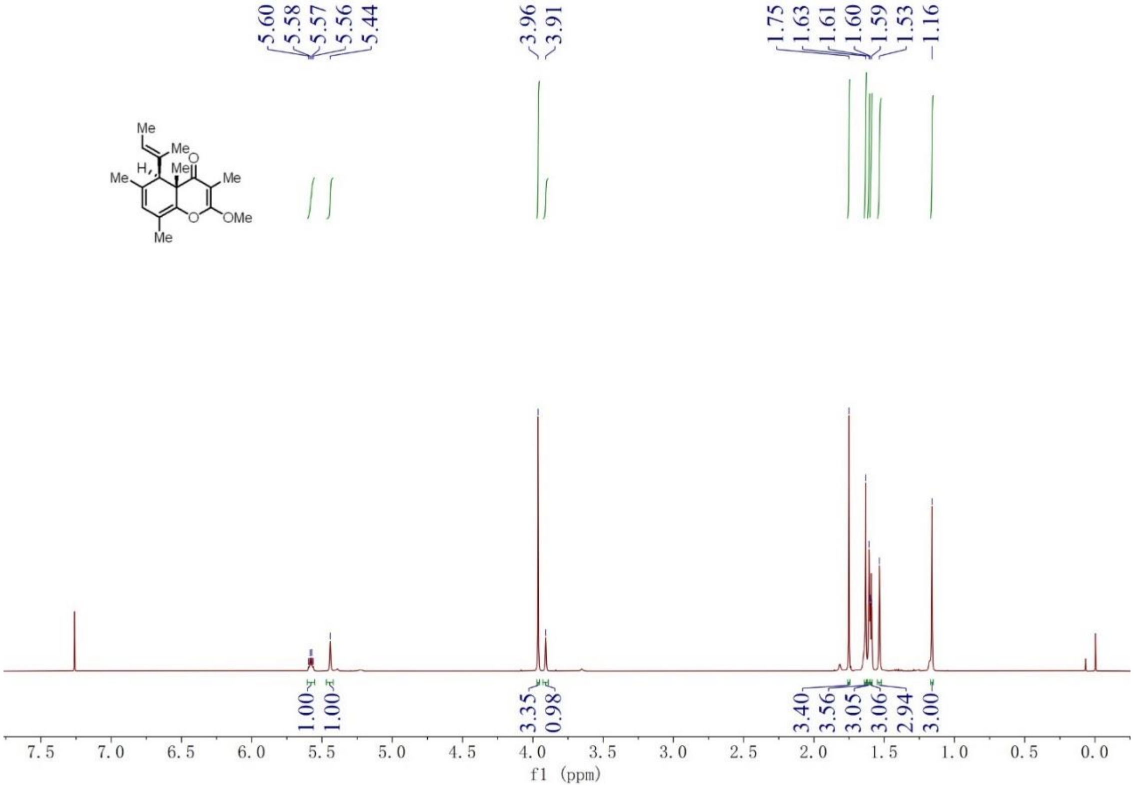

$^1$H NMR spectrum of compound **10s** (600MHz, $CDCl_3$)

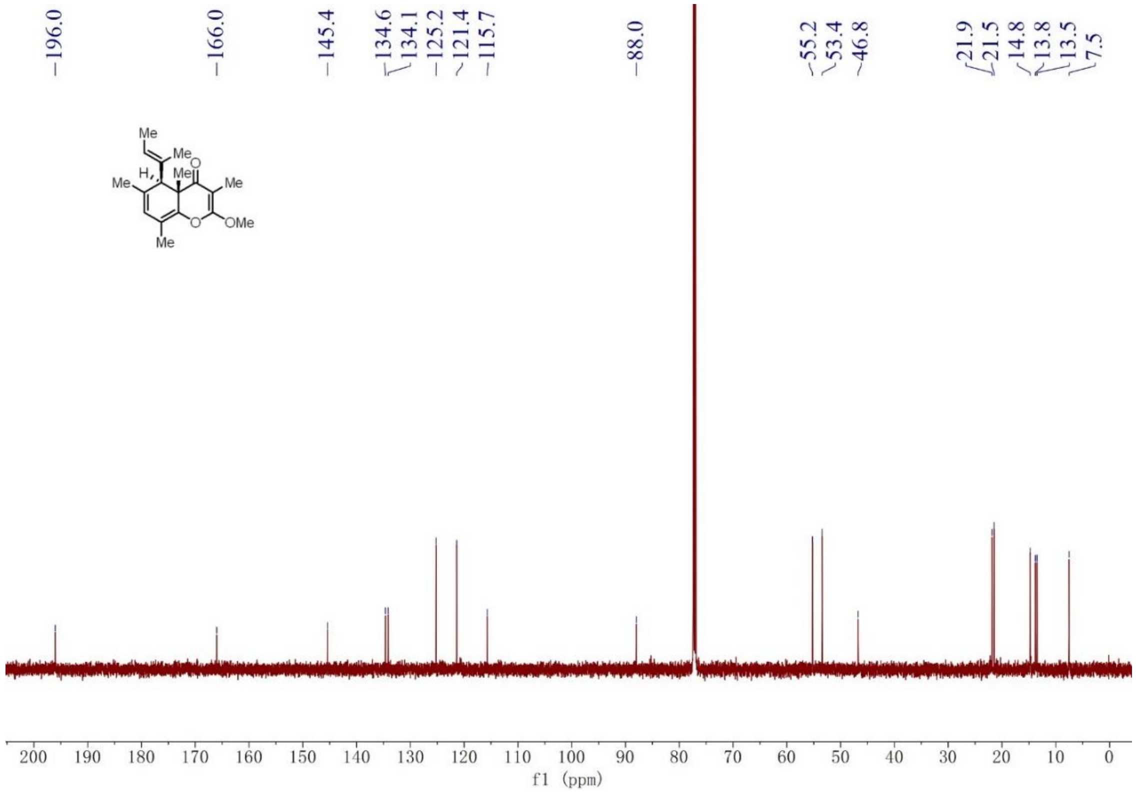


$^{13}C$ NMR spectrum of compound **10s** (151MHz, $CDCl_3$)

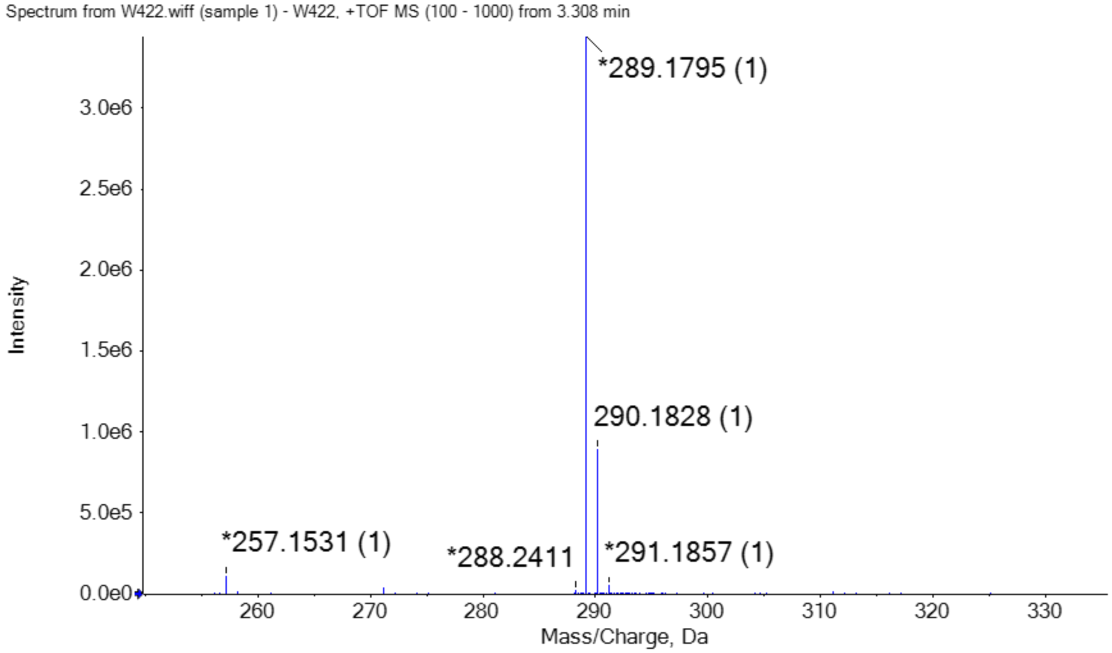


**Formula Calculator Results**

| Measured m/z | Cal m/z | Error(mmu) | Error(ppm) | Ion Formula | Ion |
|---|---|---|---|---|---|
| 289.1795 | 289.1798 | -0.3 | -1.1 | C18H25O3 | [M+H]$^+$ |

HRESIMS spectrum of compound **10s**

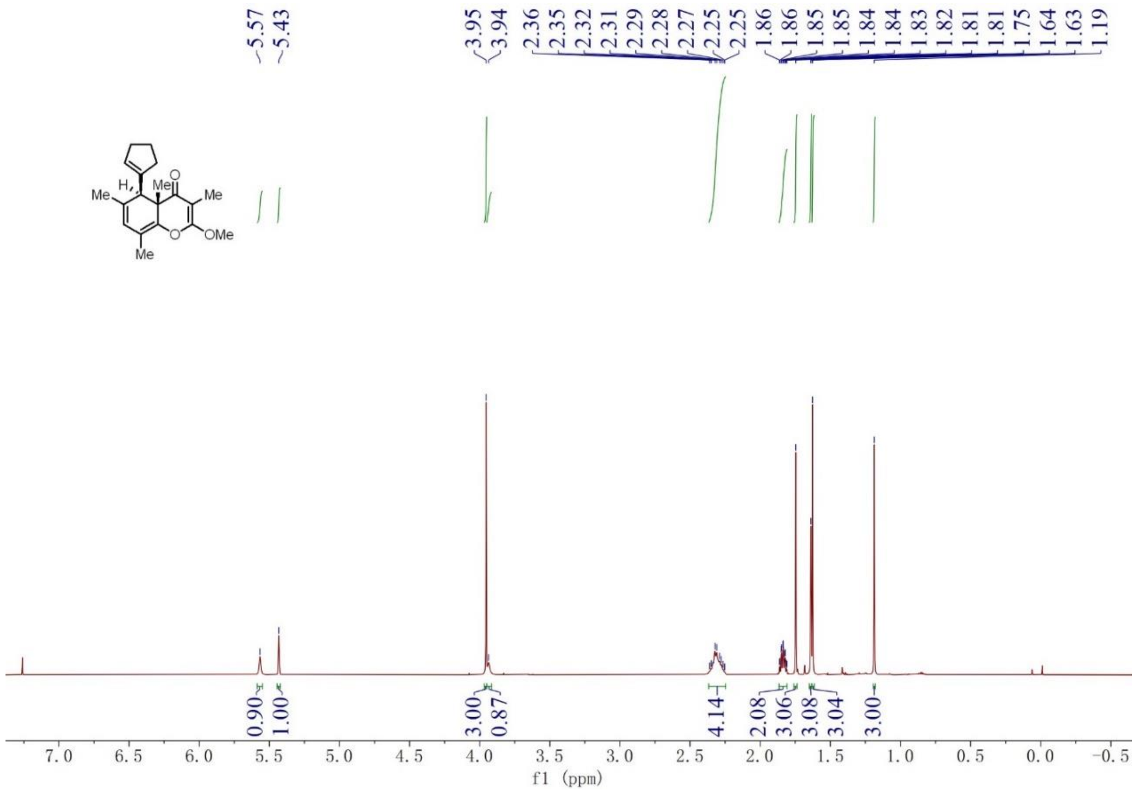


$^{1}H$ NMR spectrum of compound **10t** (600MHz, $CDCl_3$)

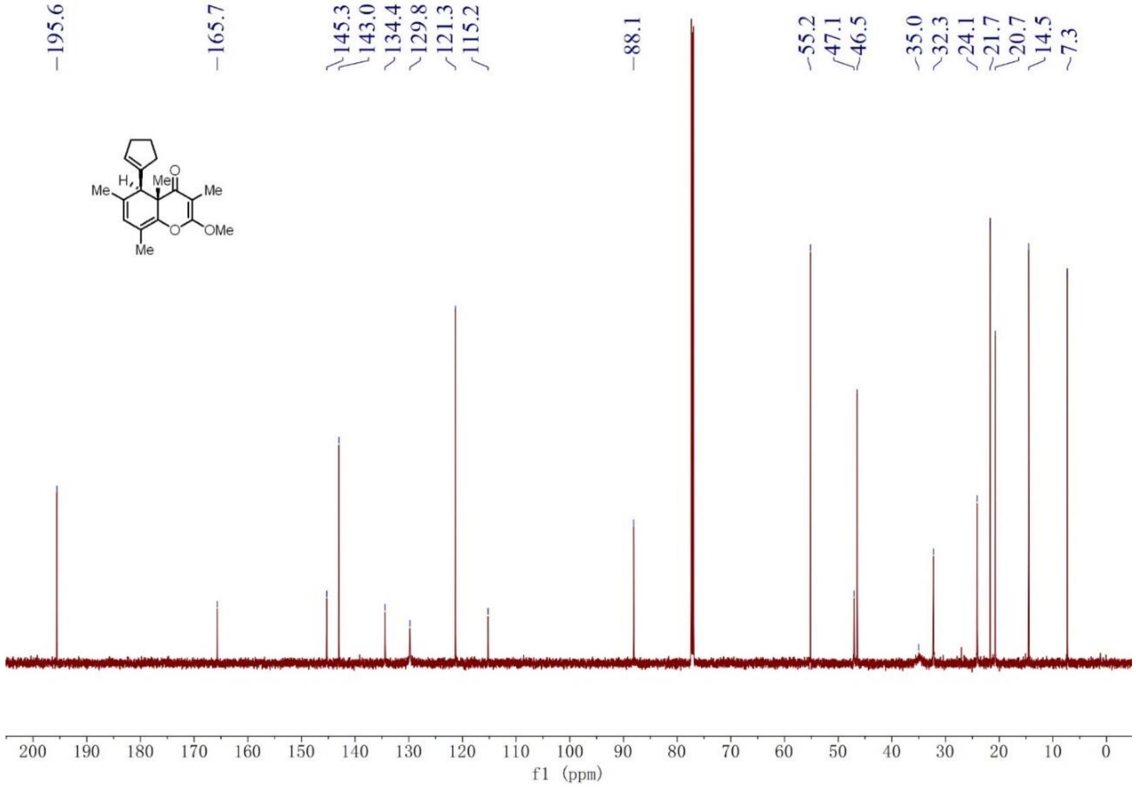


$^{13}C$ NMR spectrum of compound **10t** (151MHz, $CDCl_3$)

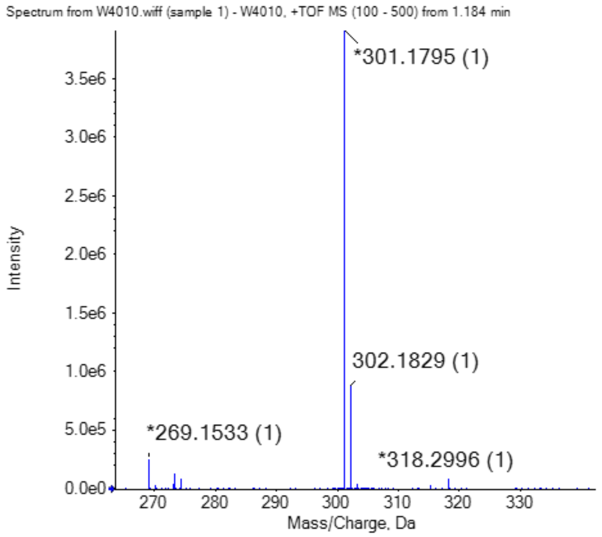


**Formula Calculator Results**

| Measure m/z | Cal m/z | Error(mmu) | Error(ppm) | Ion Formula | Ion |
|---|---|---|---|---|---|
| 301.1795 | 301.1798 | -0.3 | -1.1 | C19H25O3 | [M+H]$^+$ |

HRESIMS spectrum of compound **10t**

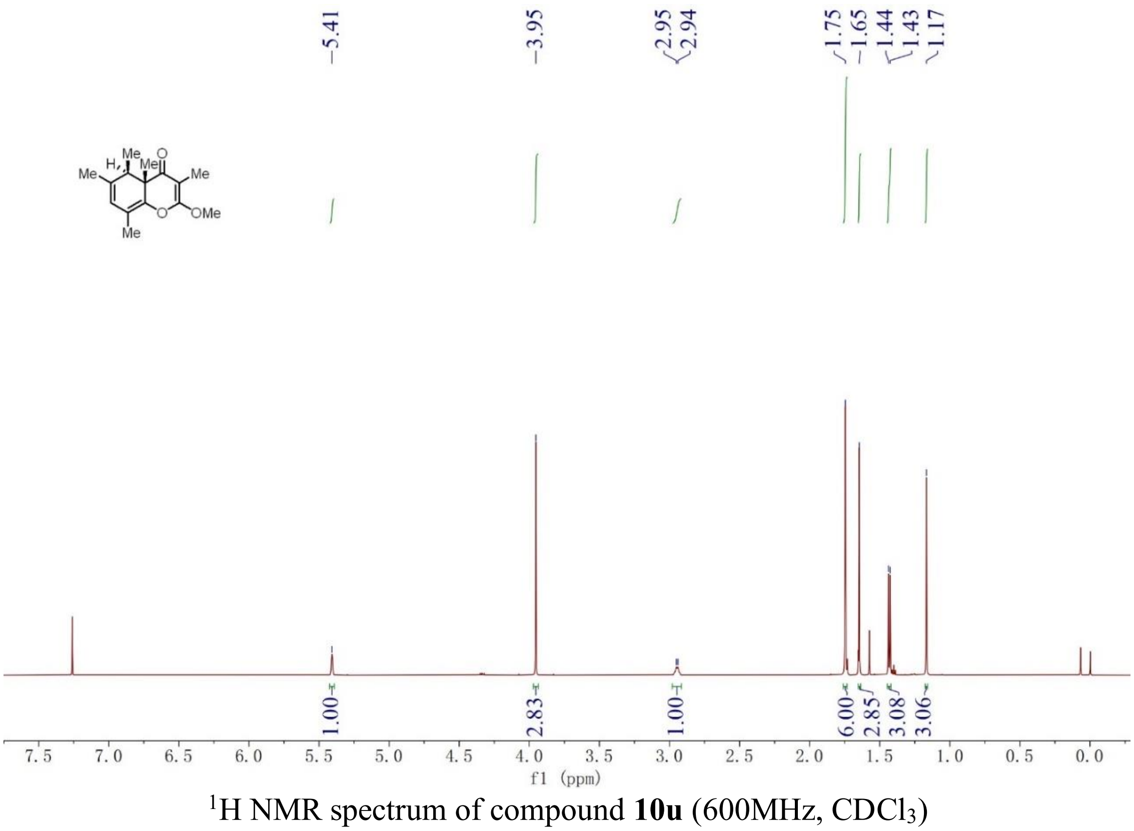


$^1$H NMR spectrum of compound **10u** (600MHz, $CDCl_3$)

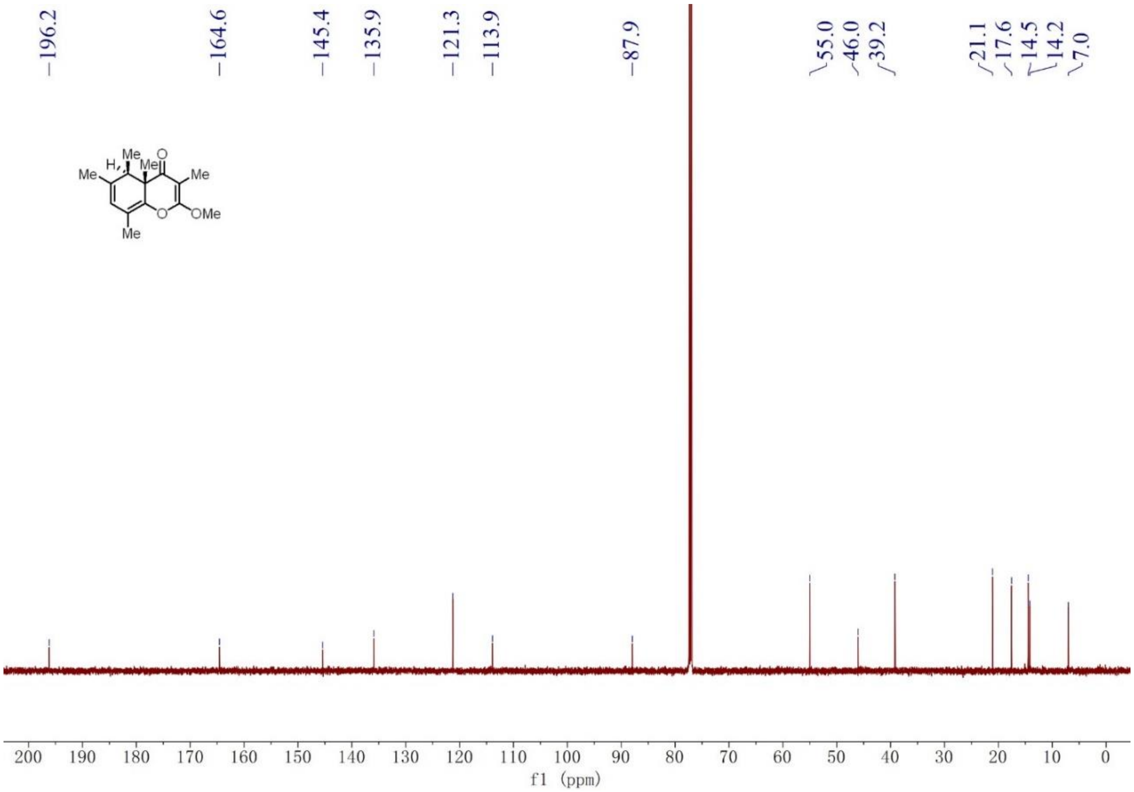


$^{13}$C NMR spectrum of compound **10u** (151MHz, $CDCl_3$)

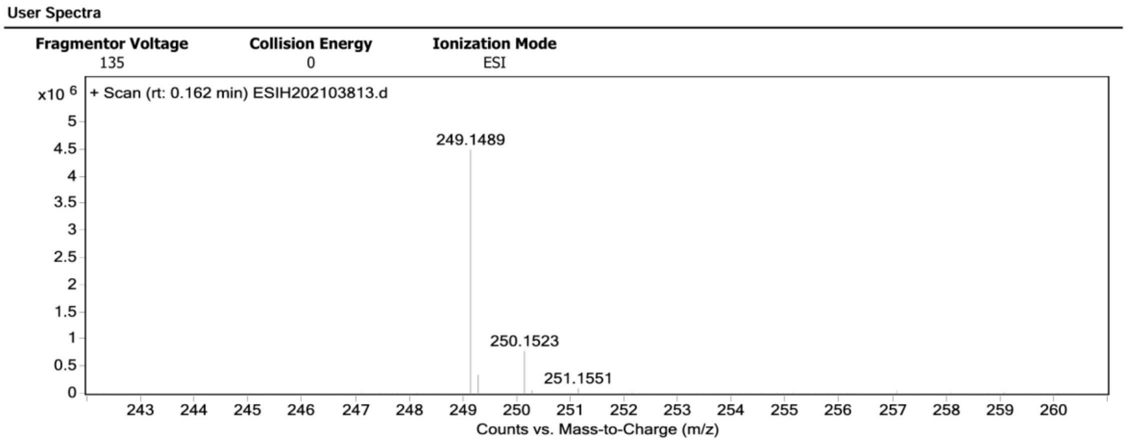


**Formula Calculator Results**

| m/z | Calc m/z | Diff (mDa) | Diff (ppm) | Ion Formula | Ion |
|---|---|---|---|---|---|
| 249.1489 | 249.1485 | -0.37 | -1.48 | C15 H21 O3 | (M+H)+ |

HRESIMS spectrum of compound **10u**

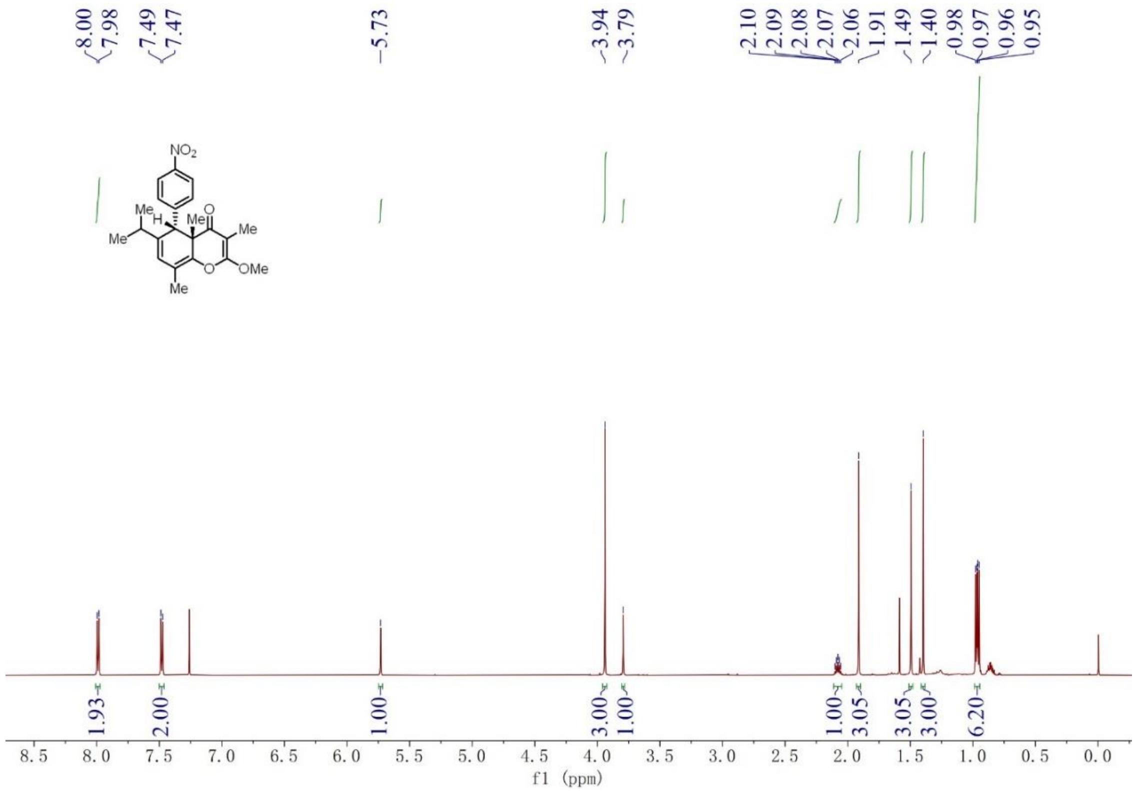

$^{1}H$ NMR spectrum of compound **10v** (600MHz, $CDCl_3$)

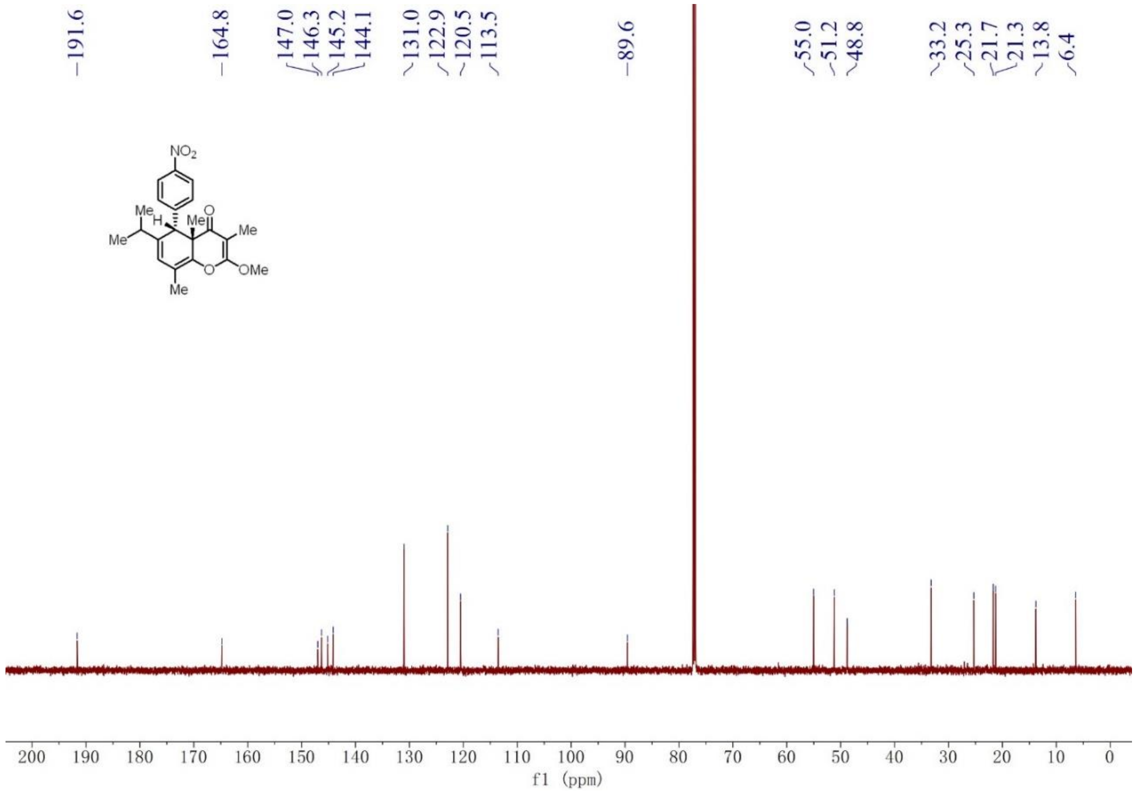

$^{13}C$ NMR spectrum of compound **10v** (151MHz, $CDCl_3$)

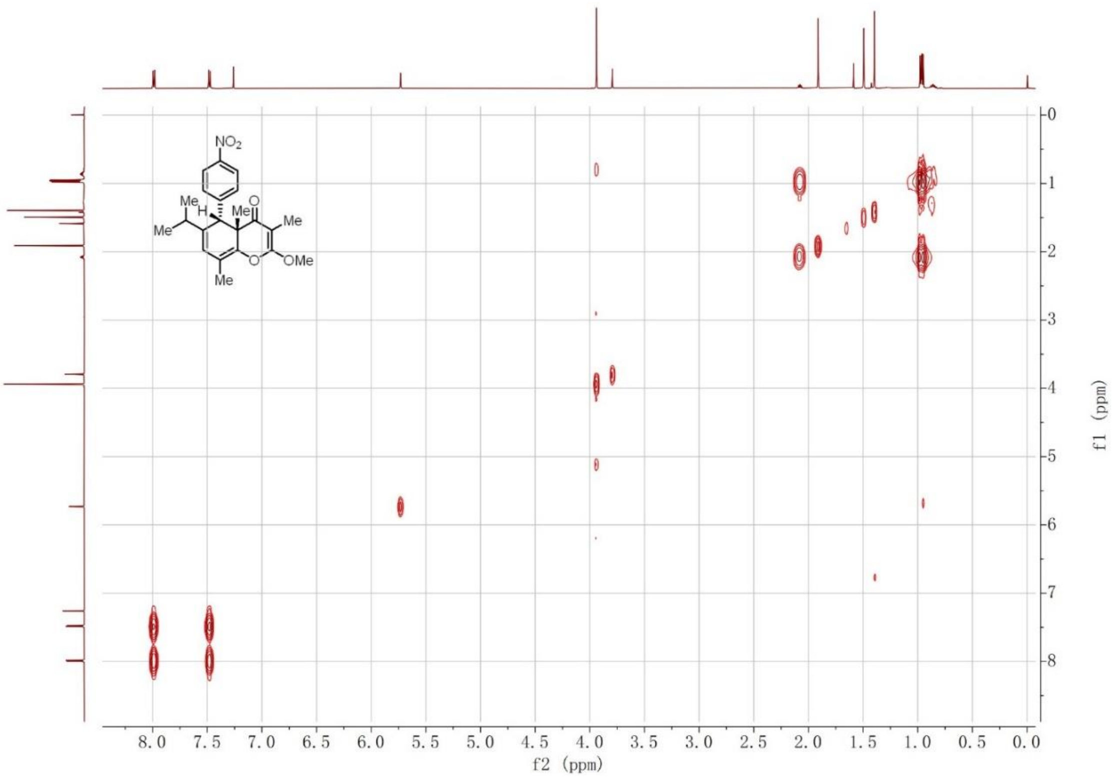

$^{1}$H-$^{1}$H COSY spectrum of compound **10v** (600 MHz, $CDCl_3$)

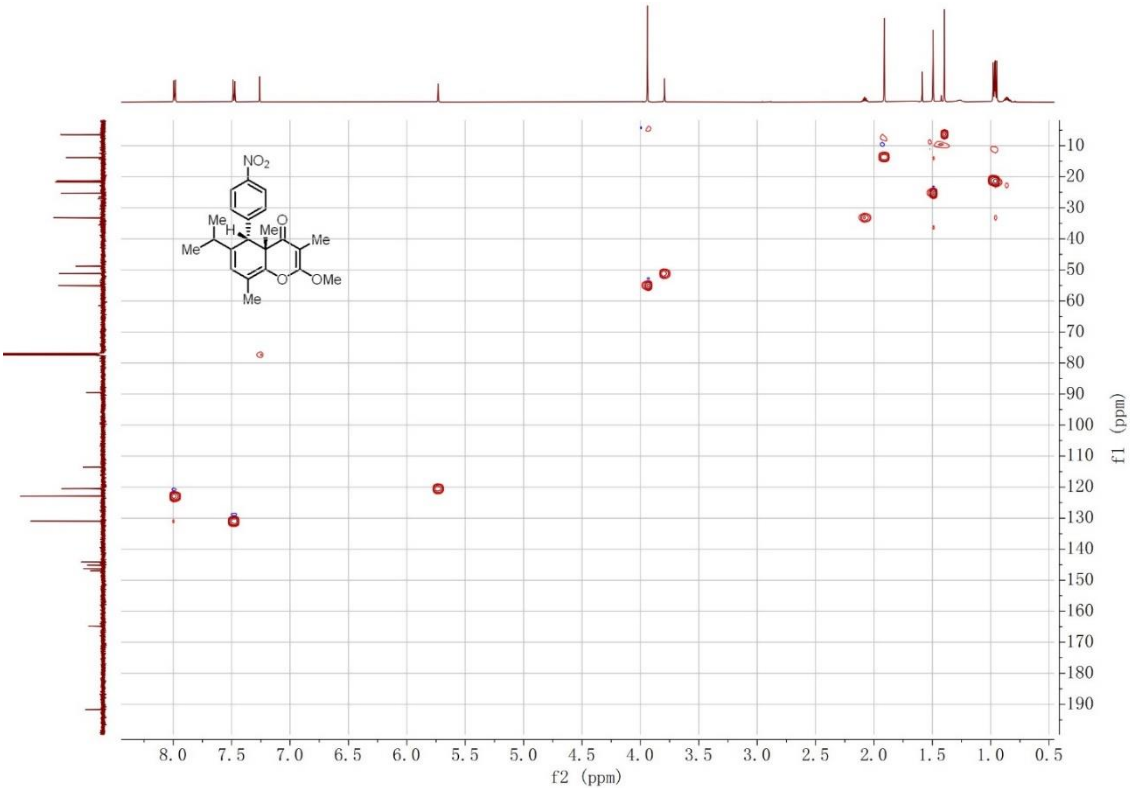

HSQC spectrum of compound **10v** (600 MHz, $CDCl_3$)

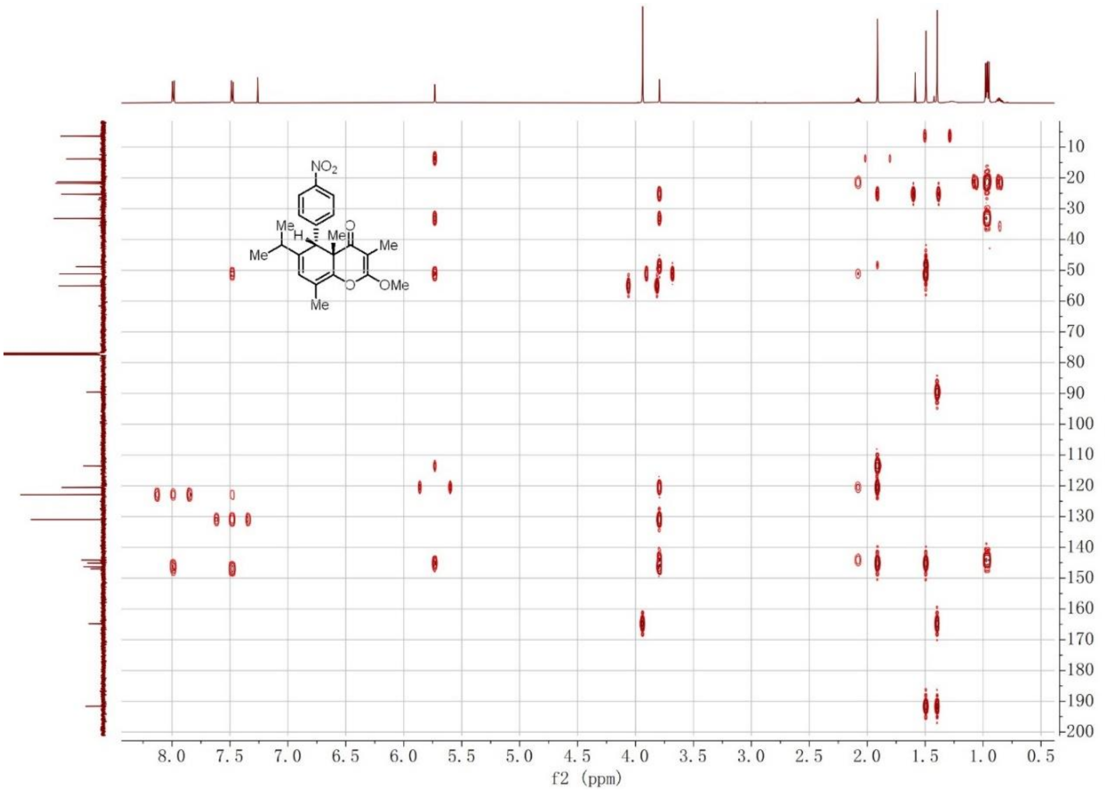


HMBC spectrum of compound **10v** (600 MHz, $CDCl_3$)

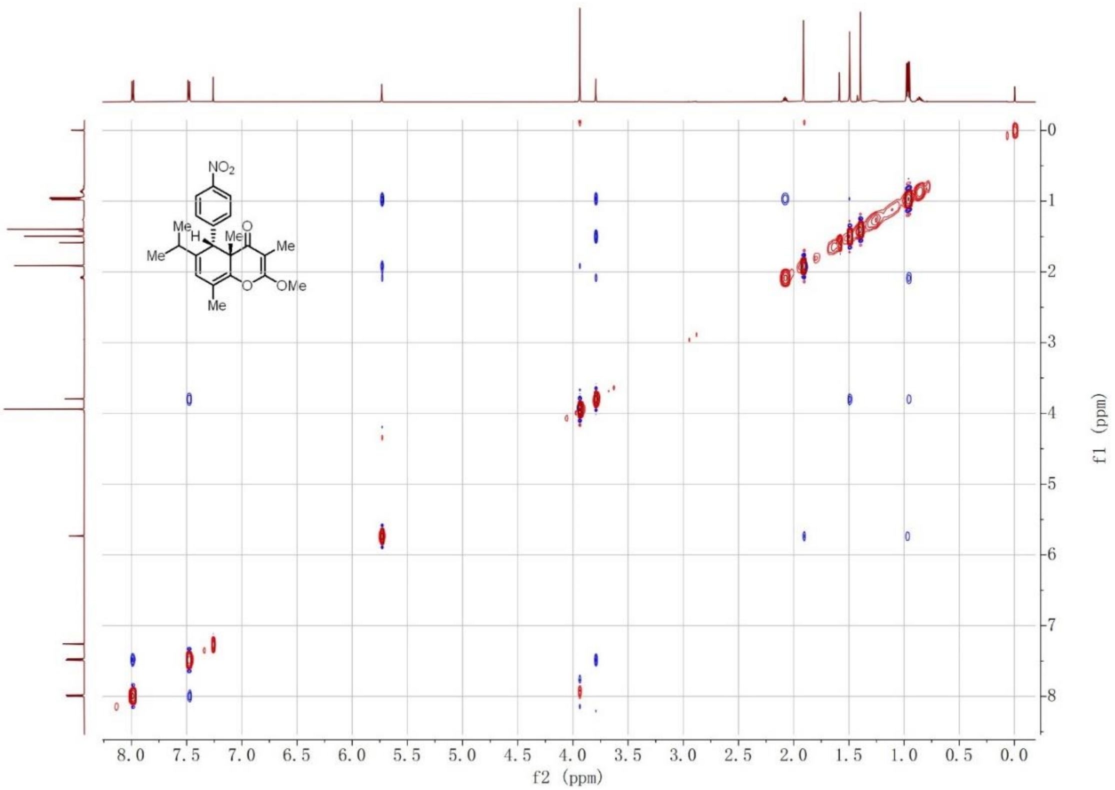


NOESY spectrum of compound **10v** (600 MHz, $CDCl_3$)

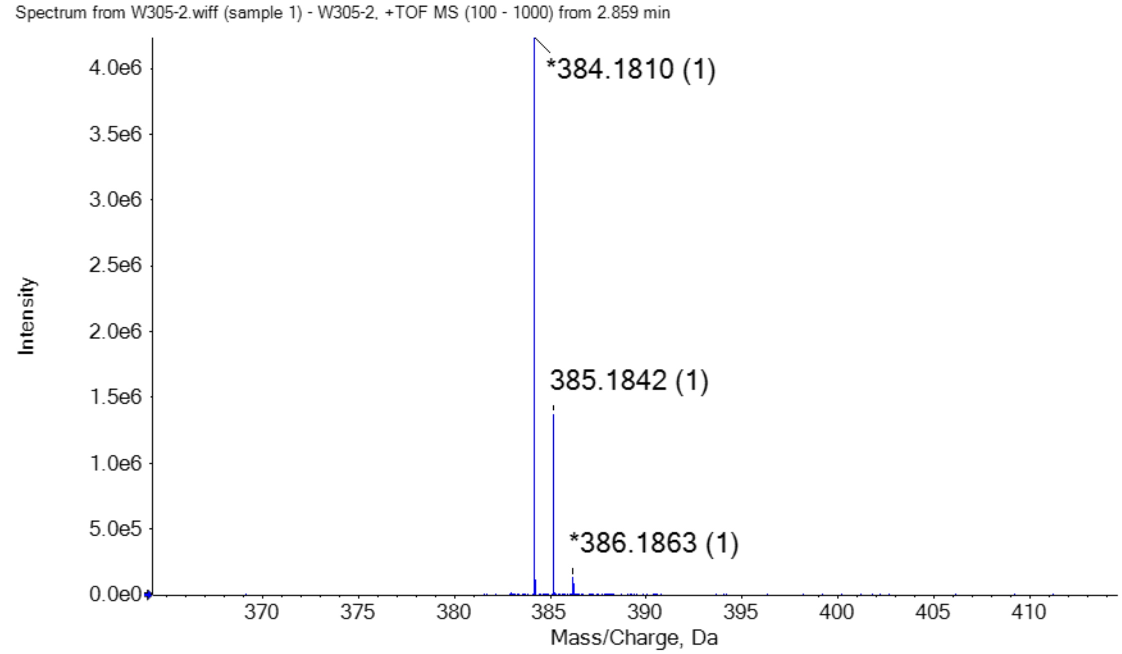


**Formula Calculator Results**

| Measured m/z | Cal m/z | Error(mmu) | Error(ppm) | Ion Formula | Ion |
|---|---|---|---|---|---|
| 384.1810 | 384.1805 | 0.5 | 1.2 | C22H26NO5 | $[M+H]^+$ |

HRESIMS spectrum of compound **10v**

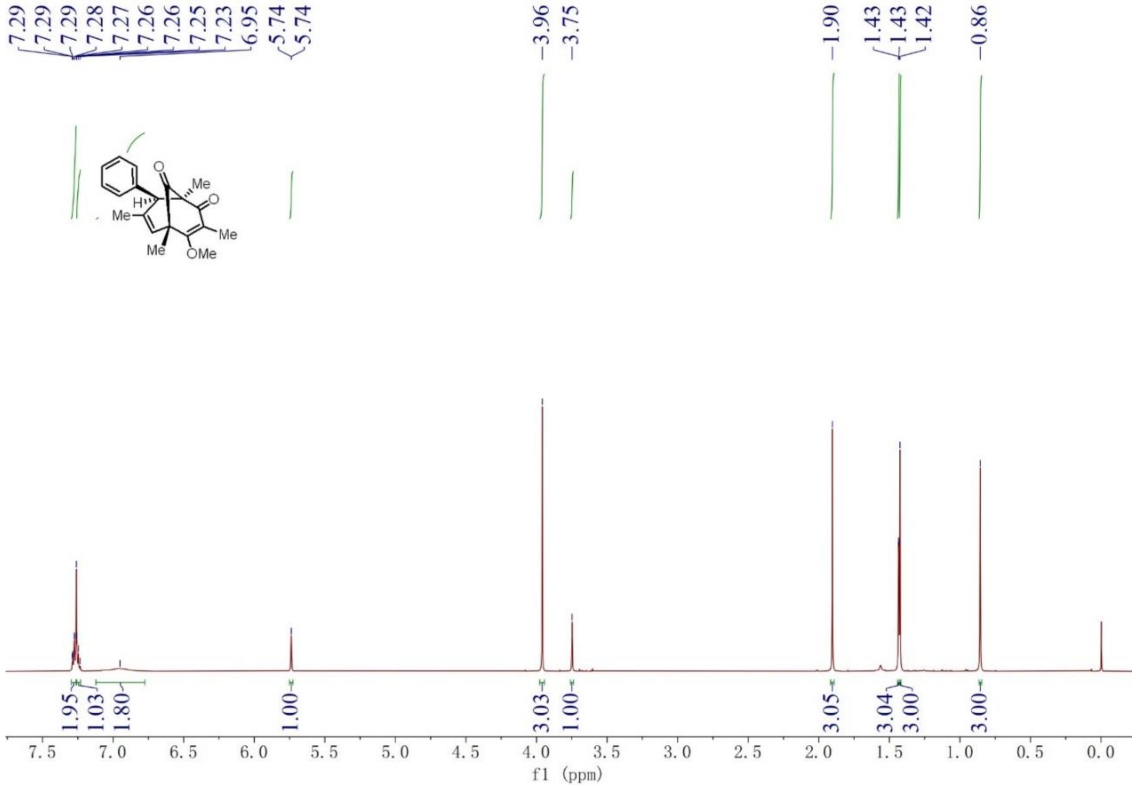


$^{1}$H NMR spectrum of compound **11a** (600MHz, $CDCl_3$)

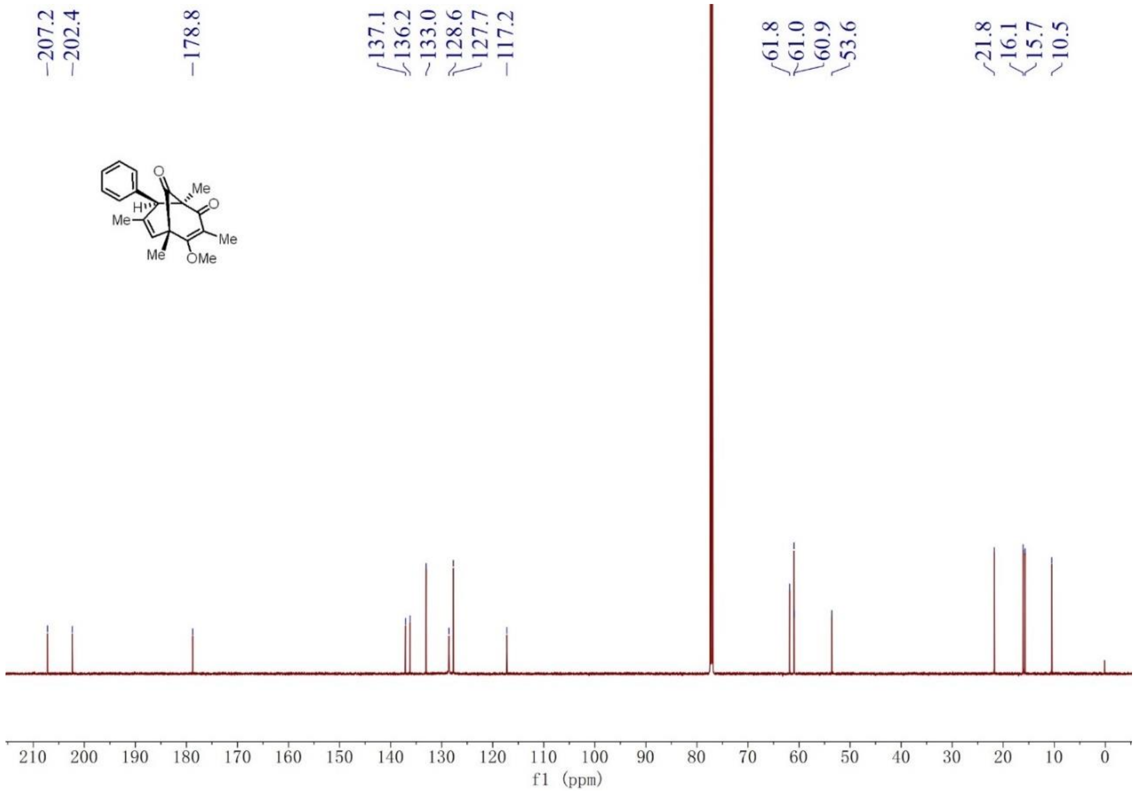


$^{13}$C NMR spectrum of compound **11a** (151MHz, $CDCl_3$)

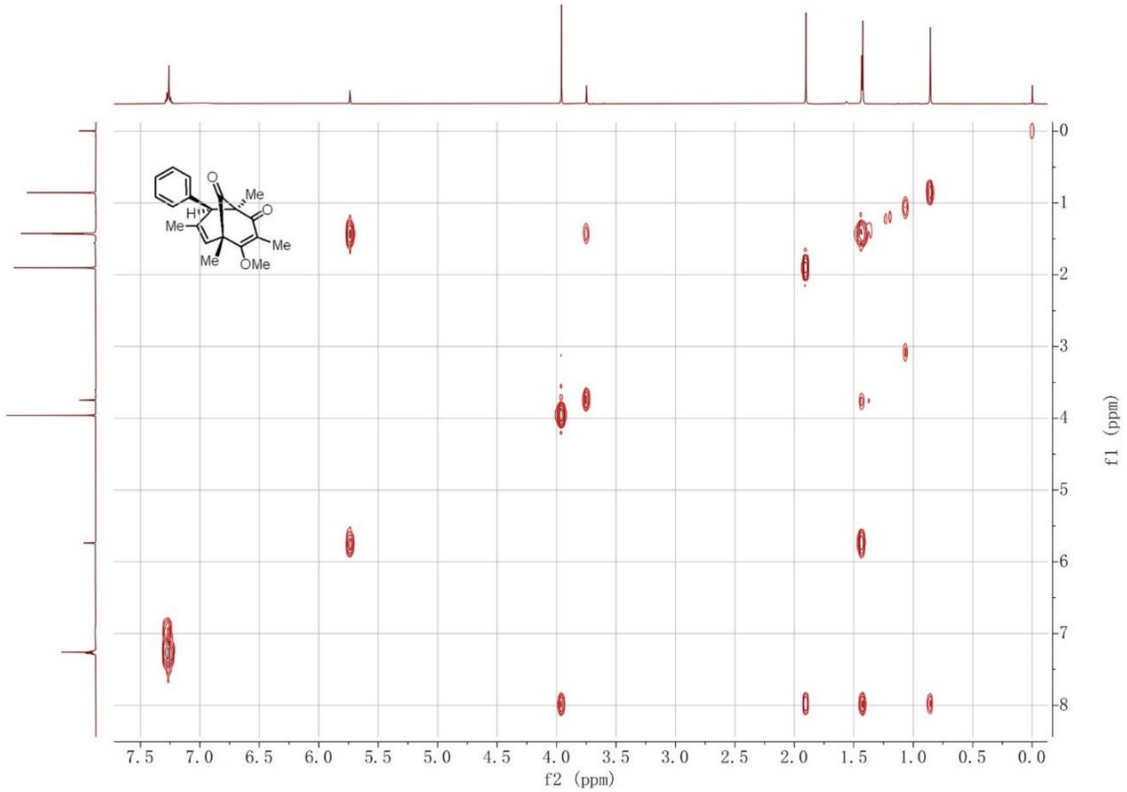


$^{1}$H-$^{1}$H COSY spectrum of compound **11a** (600 MHz, $CDCl_3$)

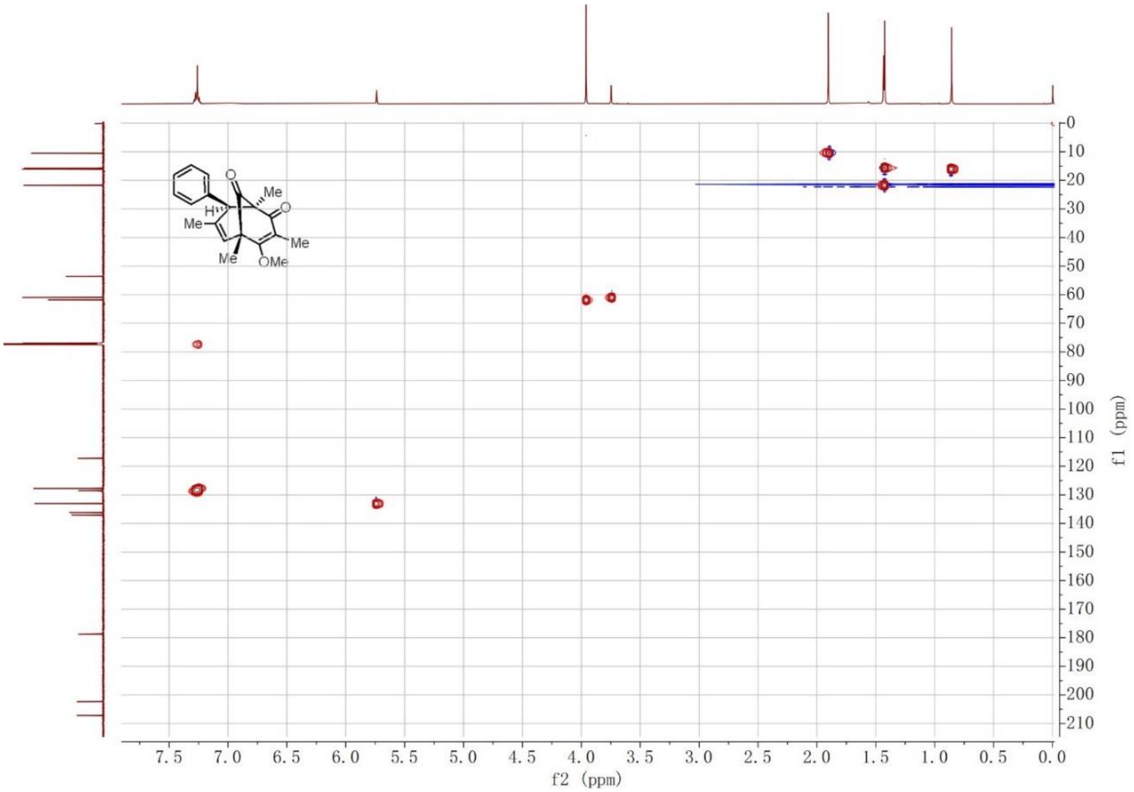

HSQC spectrum of compound **11a** (600 MHz, $CDCl_3$)

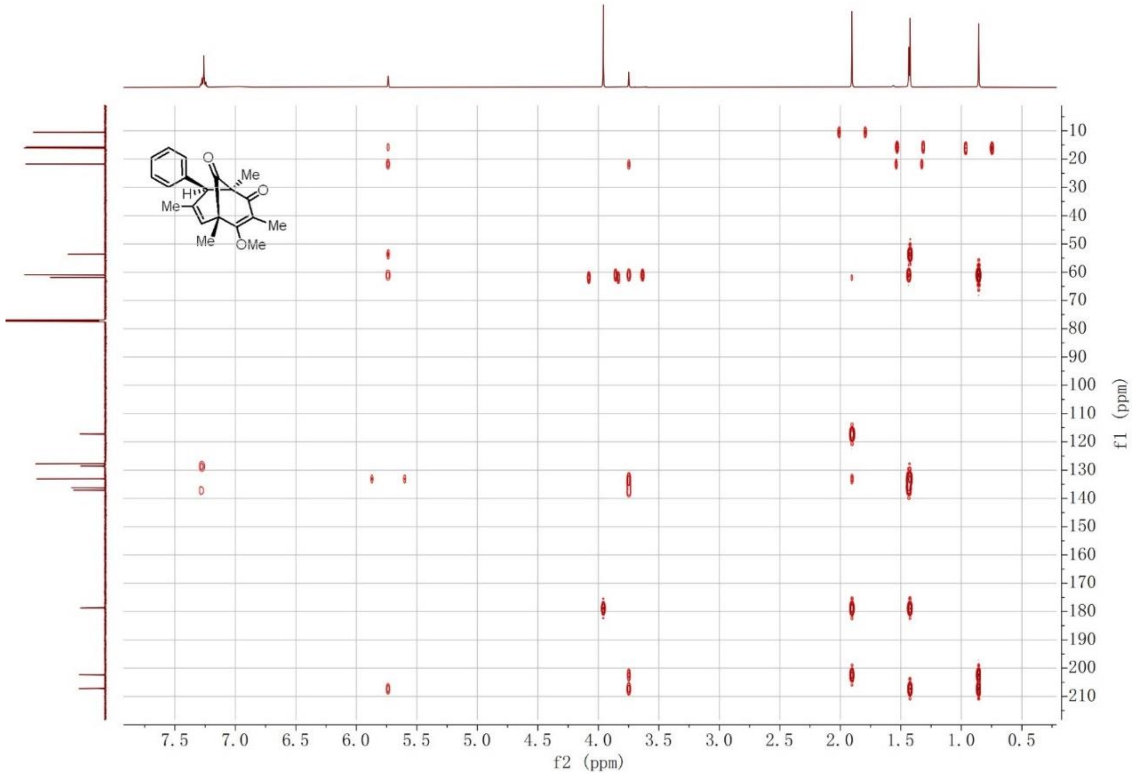

HMBC spectrum of compound **11a** (600 MHz, $CDCl_3$)

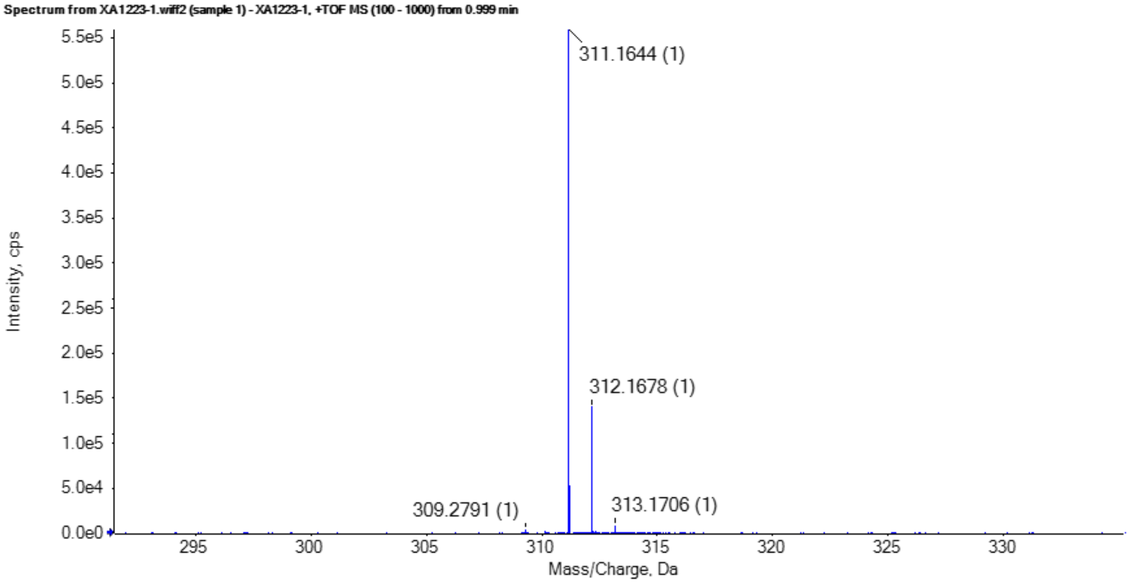


**Formula Calculator Results**

| Measured m/z | Cal m/z | Error(mmu) | Error(ppm) | Ion Formula | Ion |
|---|---|---|---|---|---|
| 311.1644 | 311.1642 | 0.3 | 0.9 | C20H23O3 | $[M+H]^+$ |

HRESIMS spectrum of compound **11a**

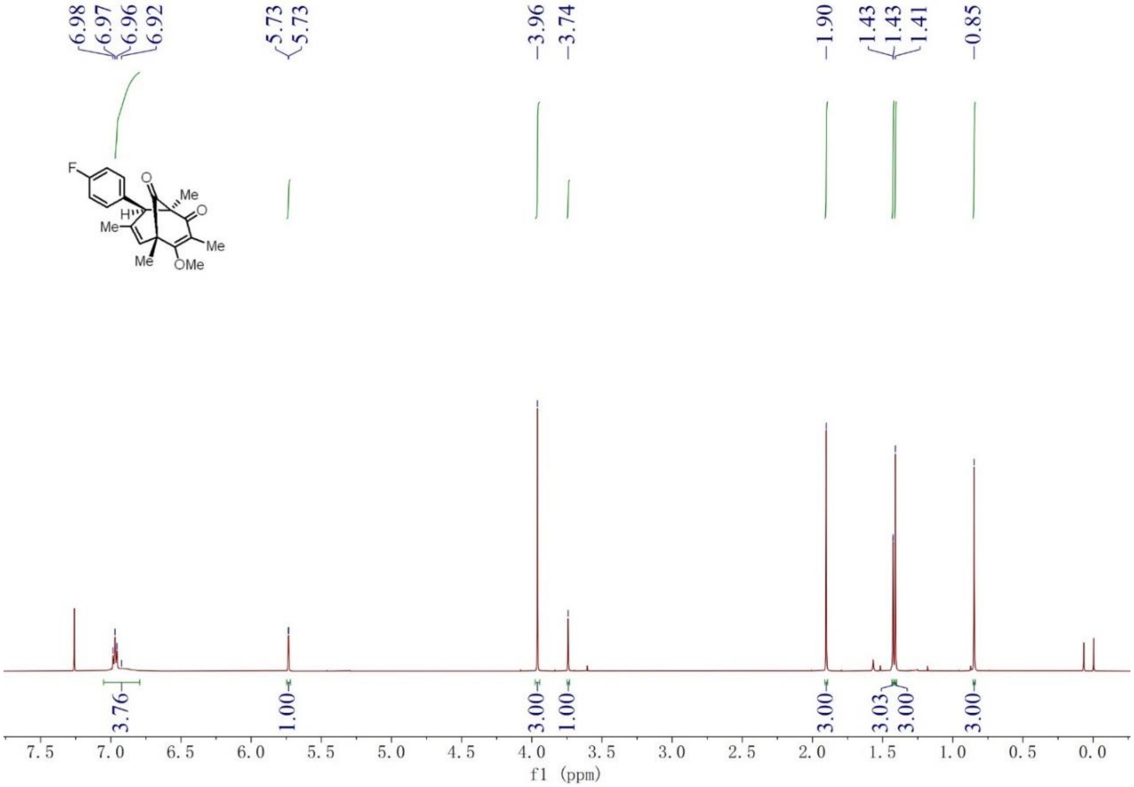


$^1$H NMR spectrum of compound **11b** (600MHz, $CDCl_3$)

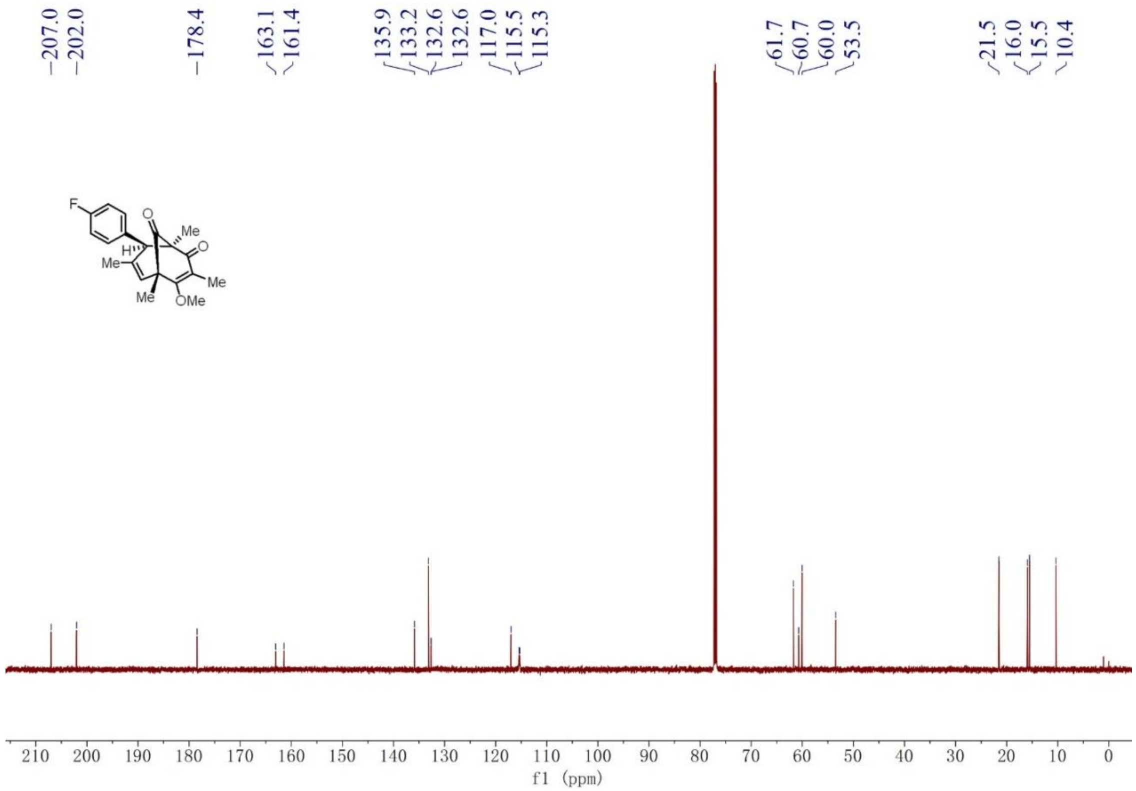


$^{13}C$ NMR spectrum of compound **11b** (151MHz, $CDCl_3$)

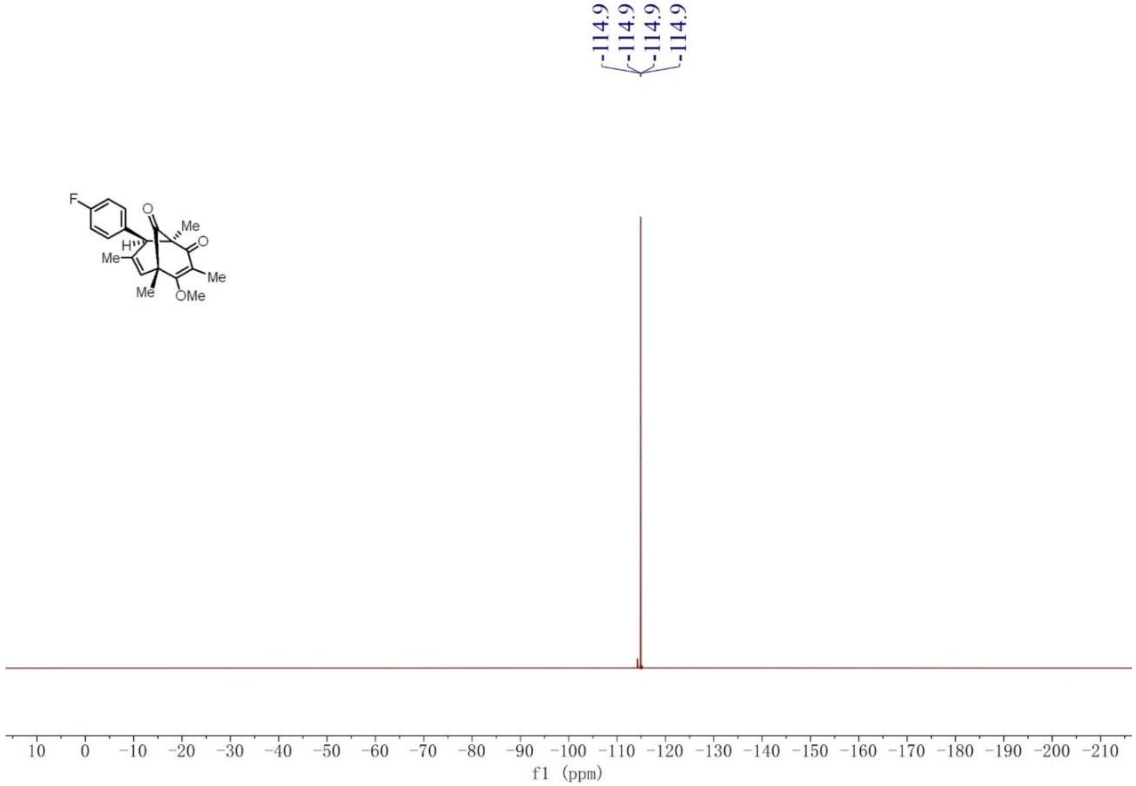


$^{19}F$ NMR spectrum of compound **11b** (565MHz, $CDCl_3$)

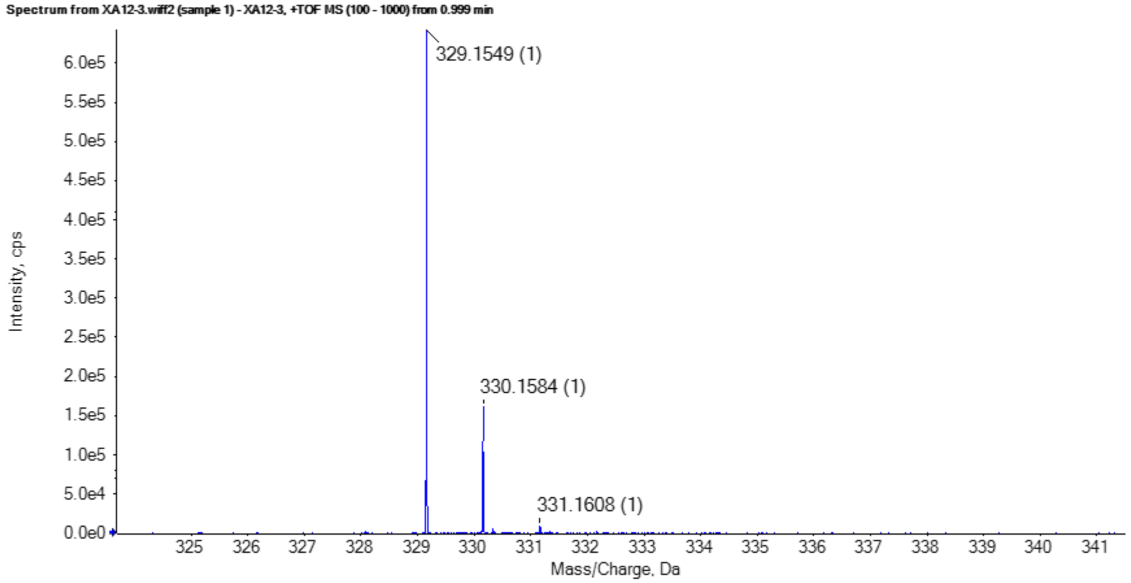


## Formula Calculator Results

| Measured m/z | Cal m/z | Error(mmu) | Error(ppm) | Ion Formula | Ion |
|---|---|---|---|---|---|
| 329.1549 | 329.1548 | 0.1 | 0.4 | $C_{20}H_{22}FO_3$ | $[M+H]^+$ |

HRESIMS spectrum of compound **11b**

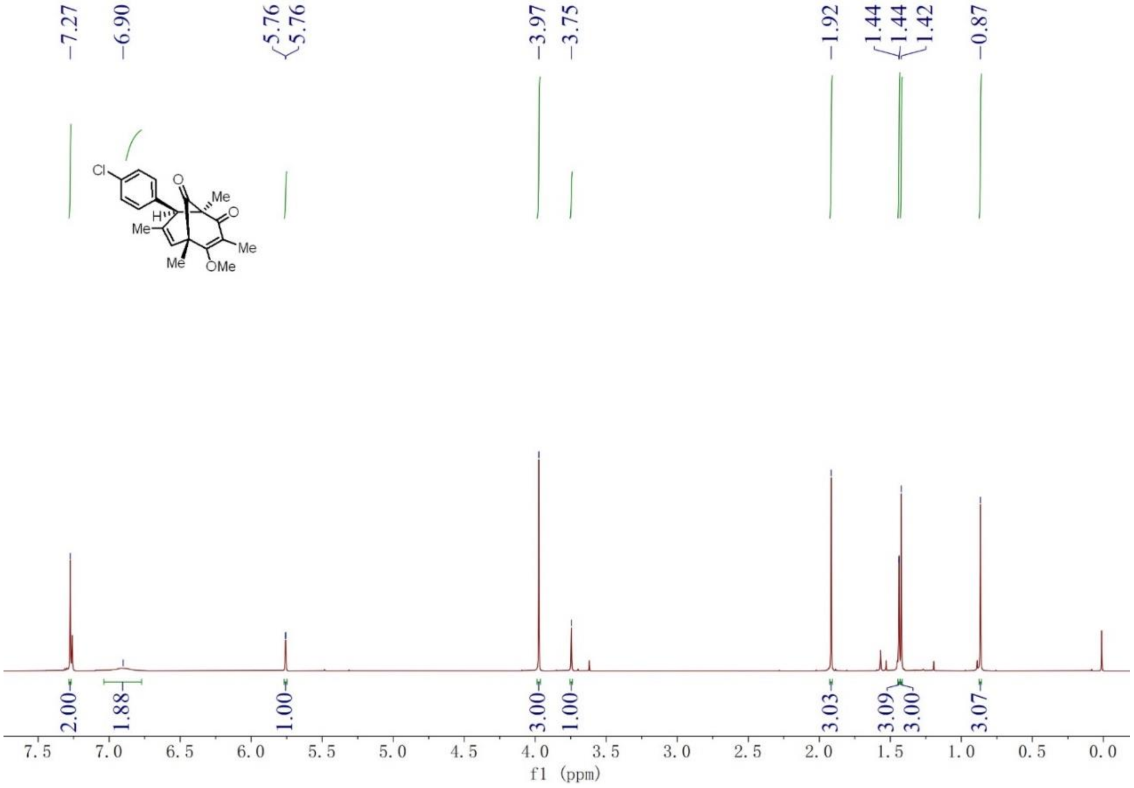


$^1$H NMR spectrum of compound **11c** (600MHz, $CDCl_3$)

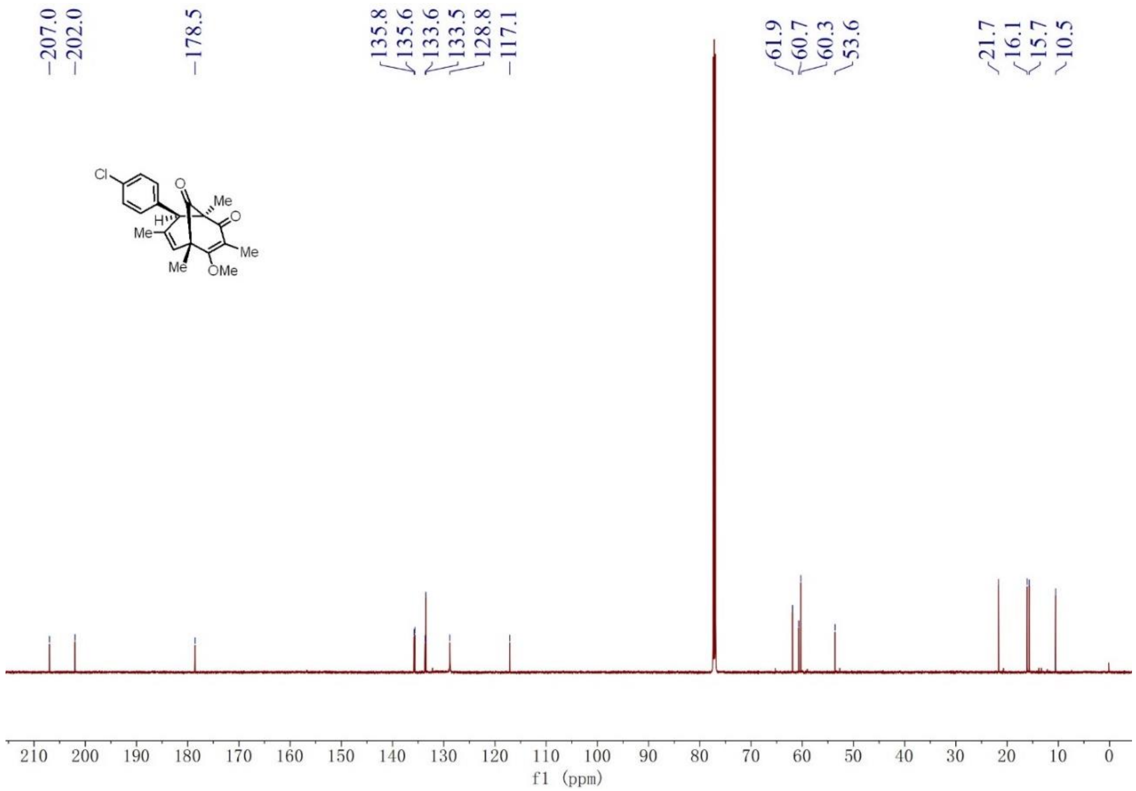


$^{13}$C NMR spectrum of compound **11c** (151MHz, $CDCl_3$)

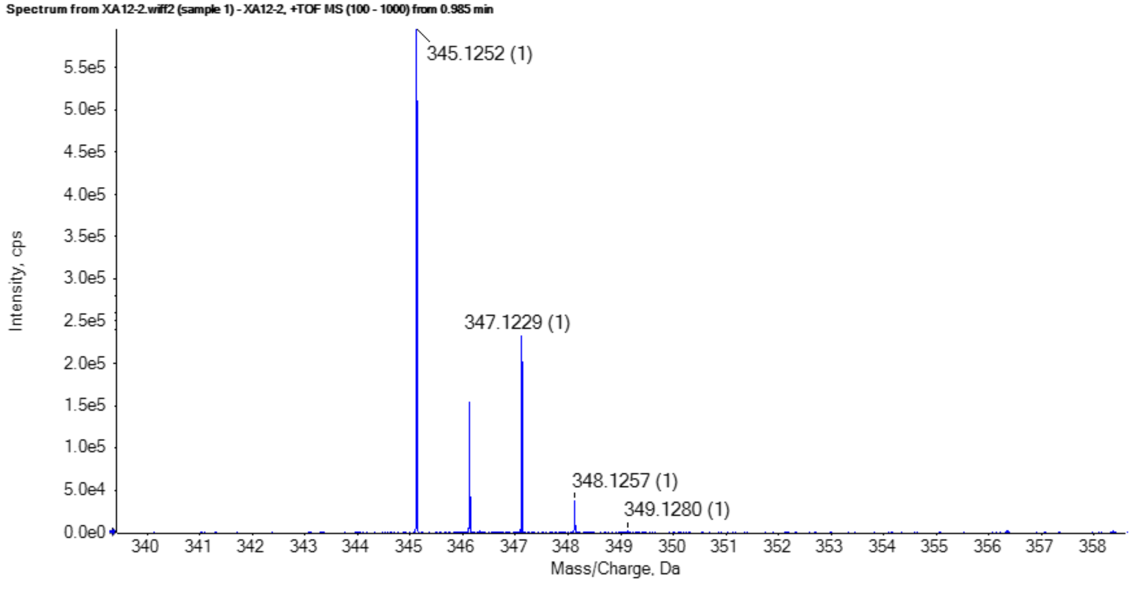


**Formula Calculator Results**

| Measured m/z | Cal m/z | Error(mmu) | Error(ppm) | Ion Formula | Ion |
|---|---|---|---|---|---|
| 345.1252 | 345.1252 | 0 | 0 | C20H22ClO3 | $[M+H]^+$ |

HRESIMS spectrum of compound **11c**

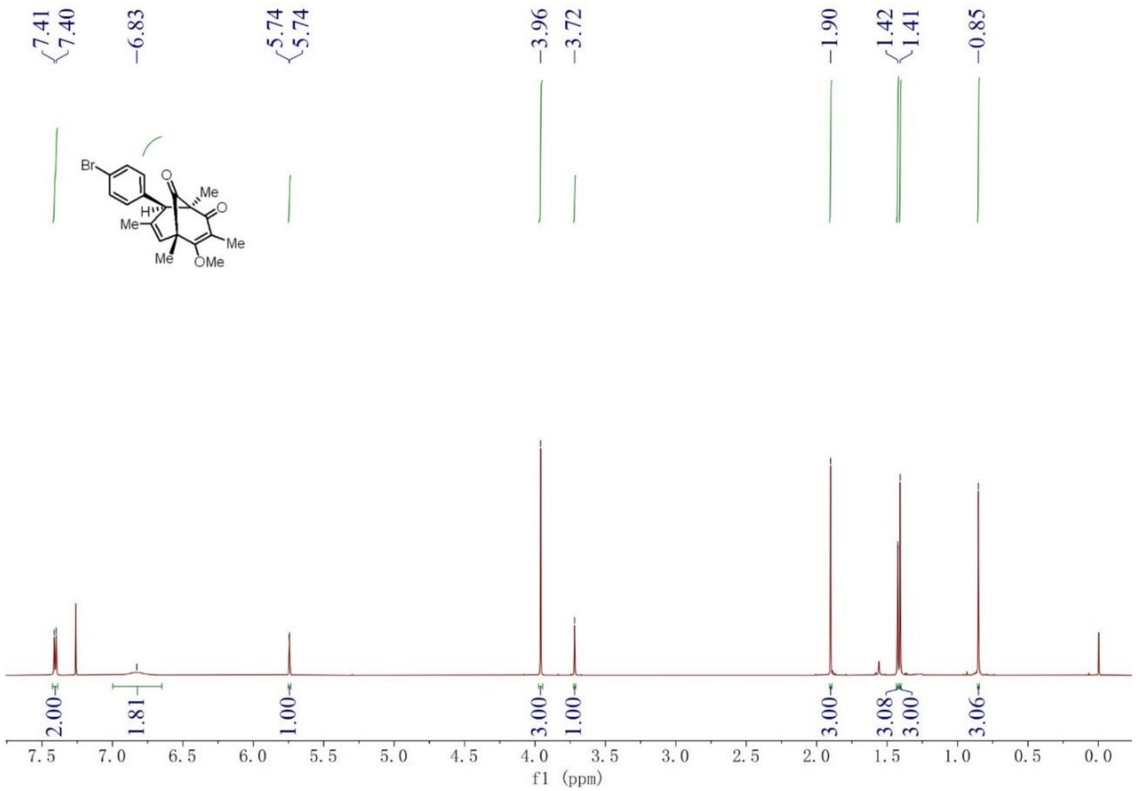


$^{1}H$ NMR spectrum of compound **11d** (600MHz, $CDCl_3$)

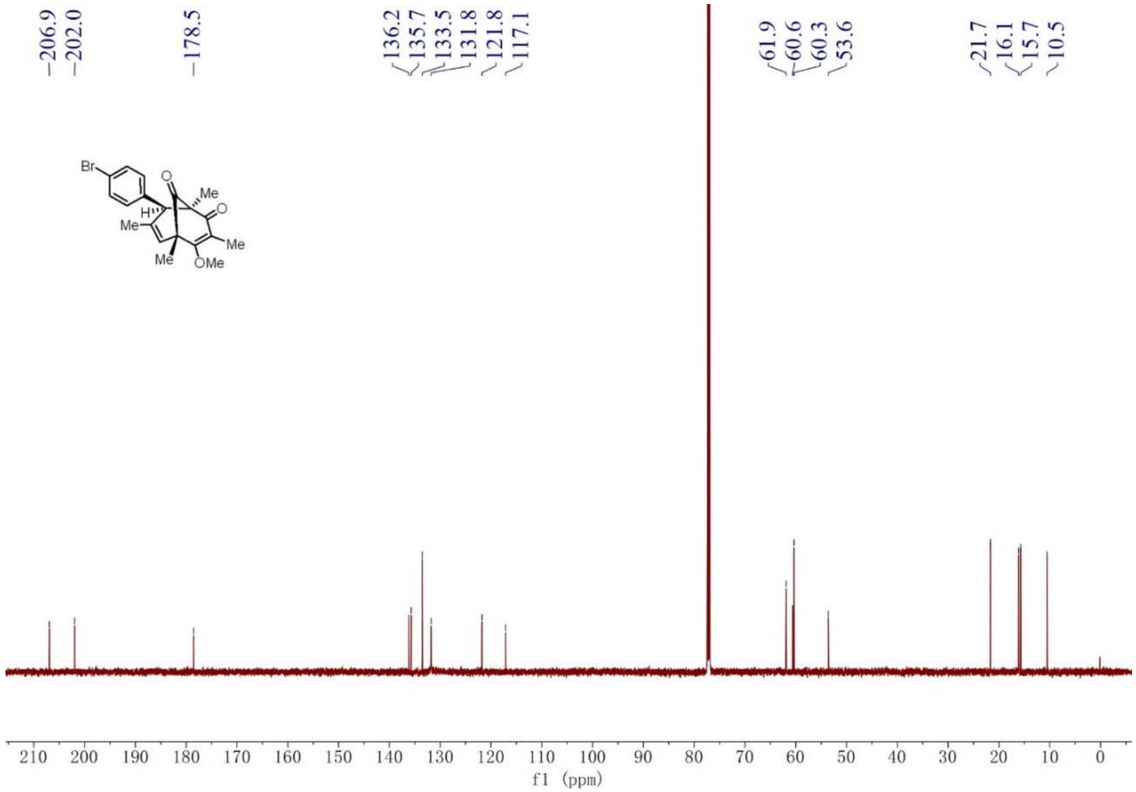


$^{13}C$ NMR spectrum of compound **11d** (151MHz, $CDCl_3$)

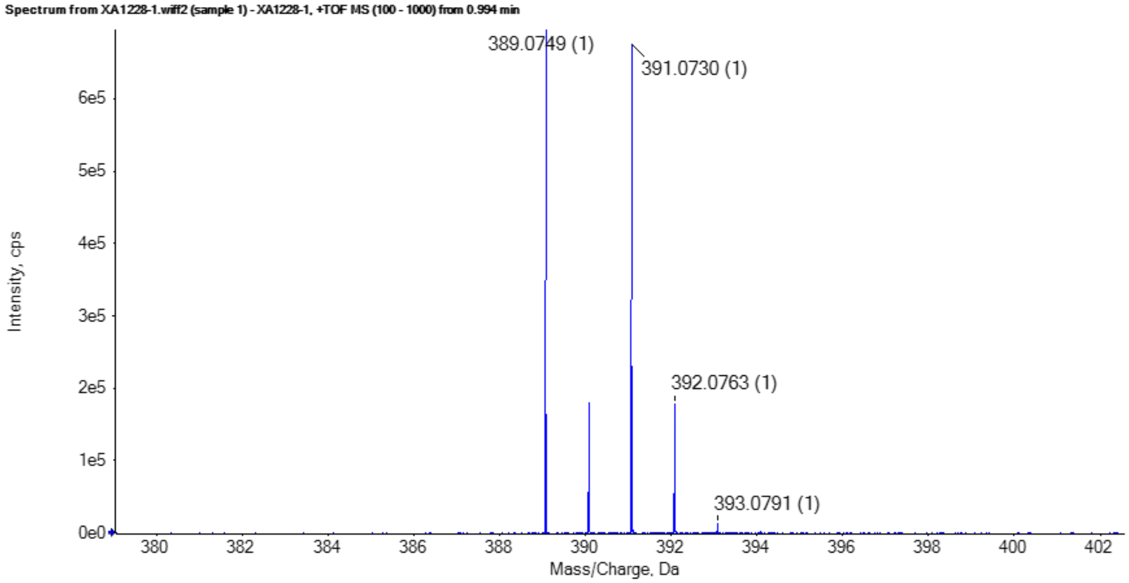


## Formula Calculator Results

| Measured m/z | Cal m/z | Error(mmu) | Error(ppm) | Ion Formula | Ion |
|---|---|---|---|---|---|
| 389.0749 | 389.0747 | 0.2 | 0.6 | C20H22BrO3 | $[M+H]^+$ |

HRESIMS spectrum of compound **11d**

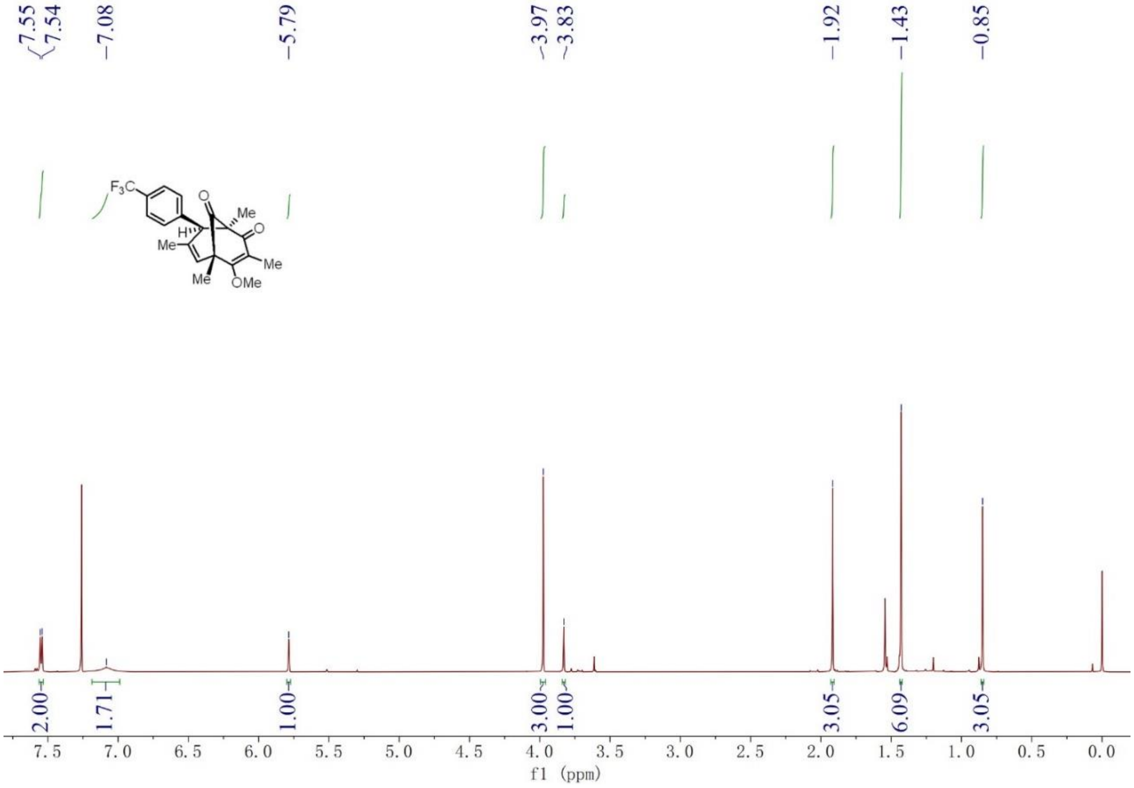


$^1$H NMR spectrum of compound **11e** (600MHz, $CDCl_3$)

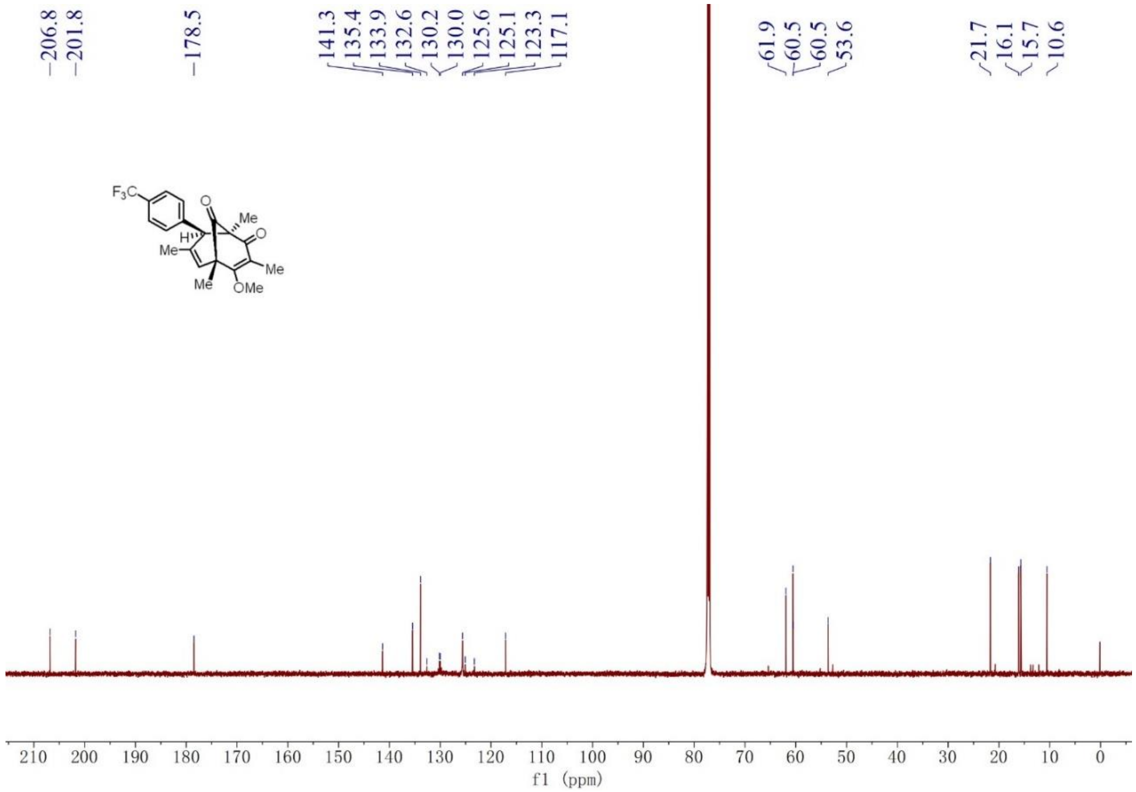


$^{13}$C NMR spectrum of compound **11e** (151MHz, $CDCl_3$)

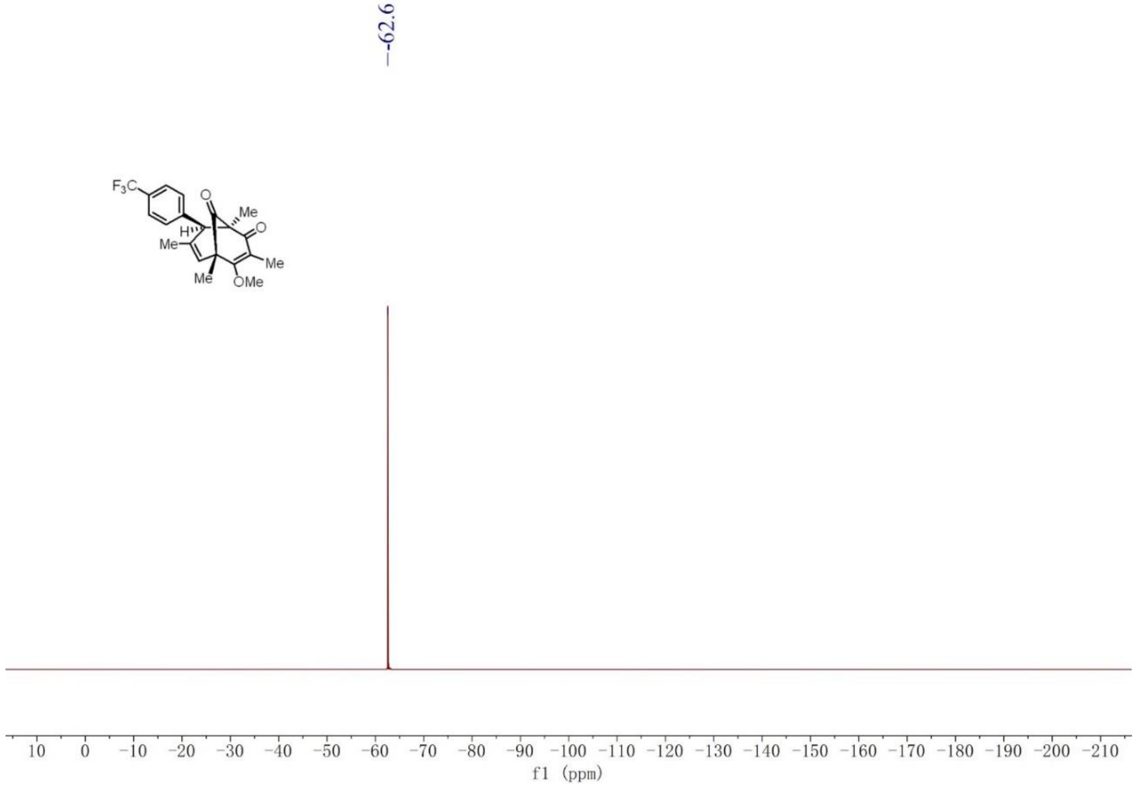


$^{19}$F NMR spectrum of compound **11e** (565MHz, $CDCl_3$)

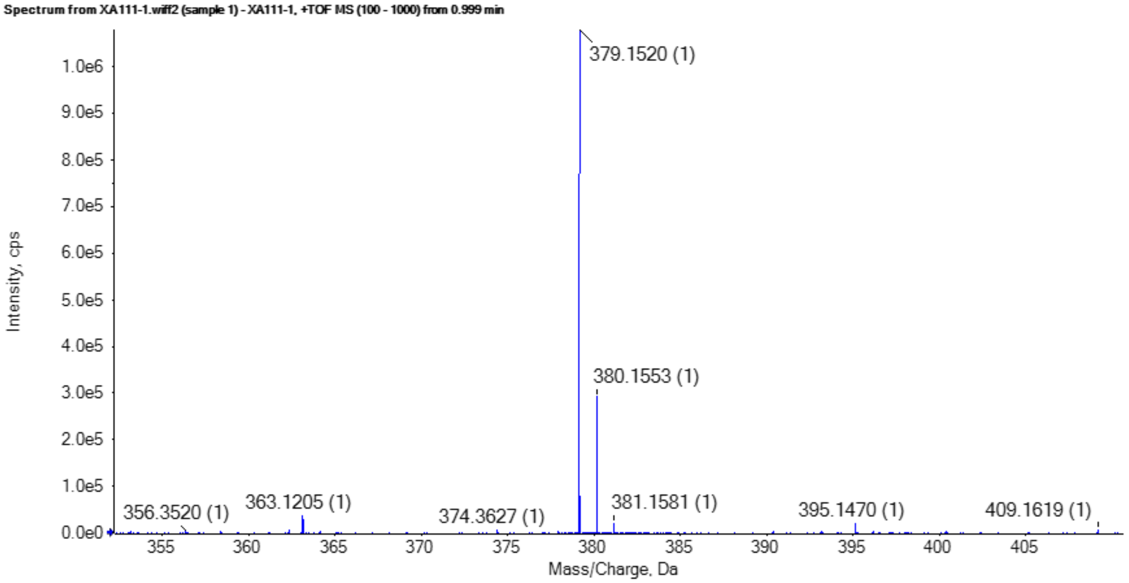


## Formula Calculator Results

| Measured m/z | Cal m/z | Error(mmu) | Error(ppm) | Ion Formula | Ion |
|---|---|---|---|---|---|
| 379.1520 | 379.1516 | 0.4 | 1.0 | C21H22F3O3 | $[M+H]^+$ |

HRESIMS spectrum of compound **11e**

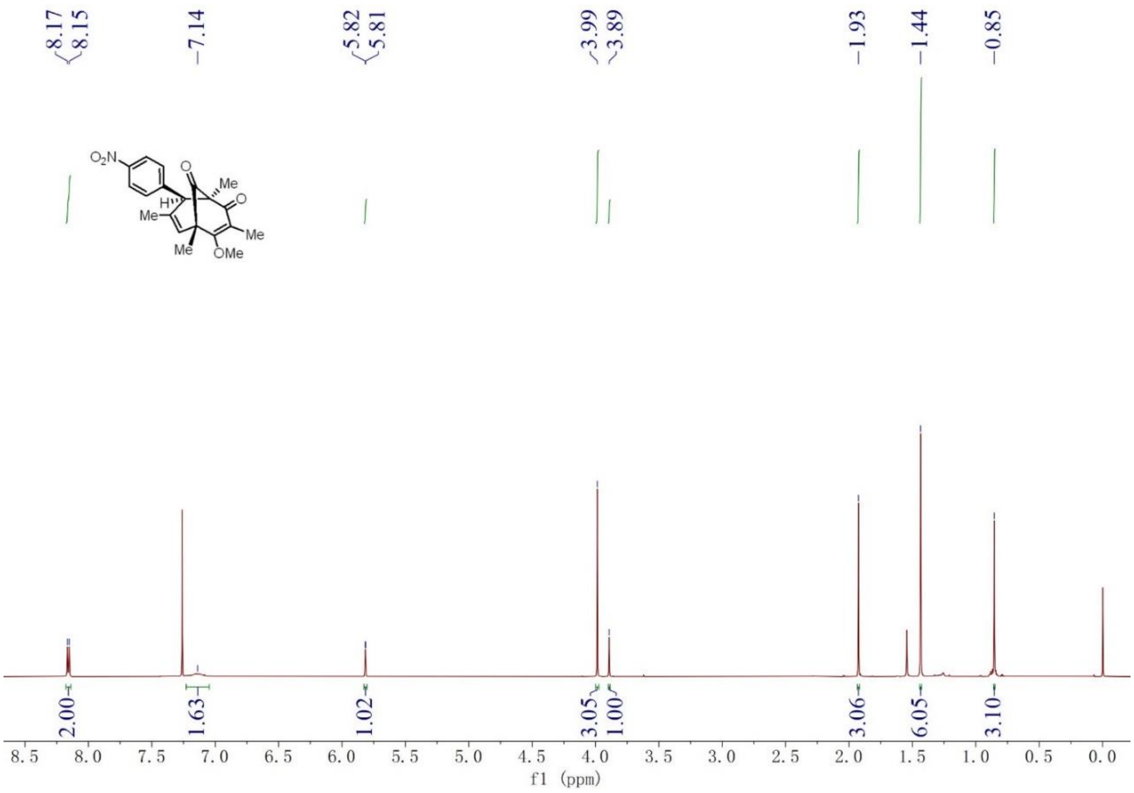


$^1$H NMR spectrum of compound **11f** (600MHz, $CDCl_3$)

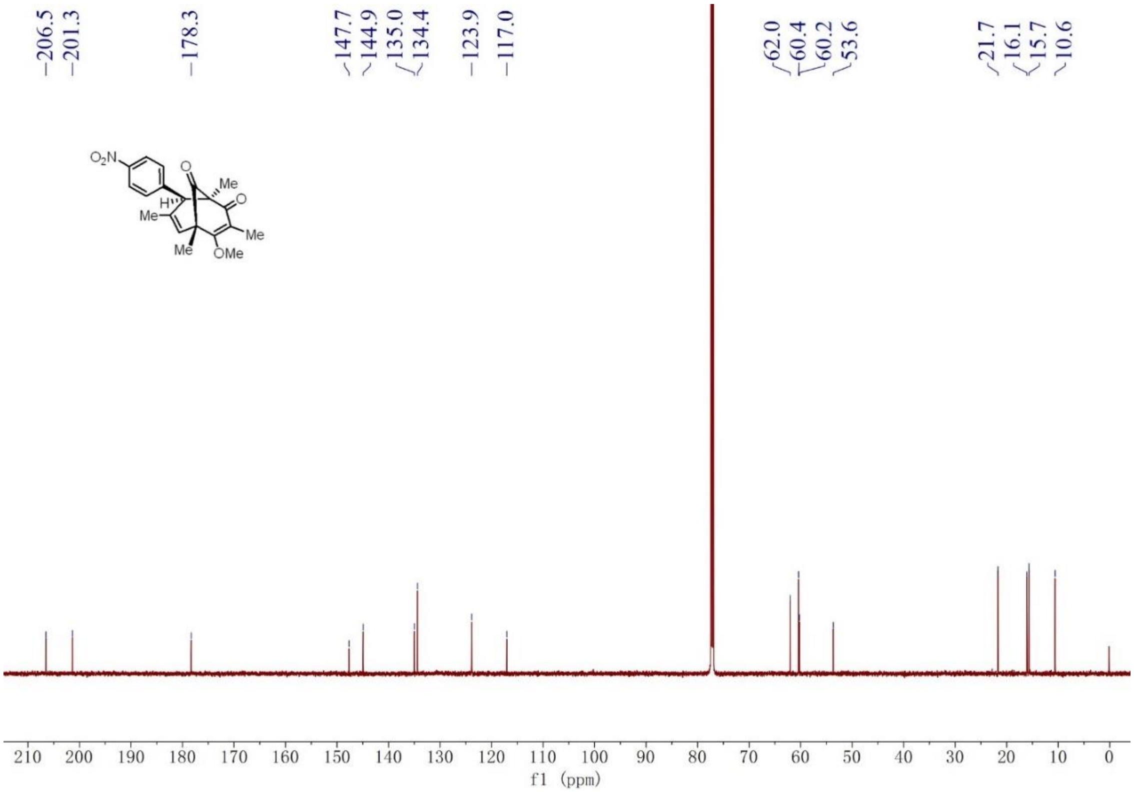


$^{13}$C NMR spectrum of compound **11f** (151MHz, $CDCl_3$)

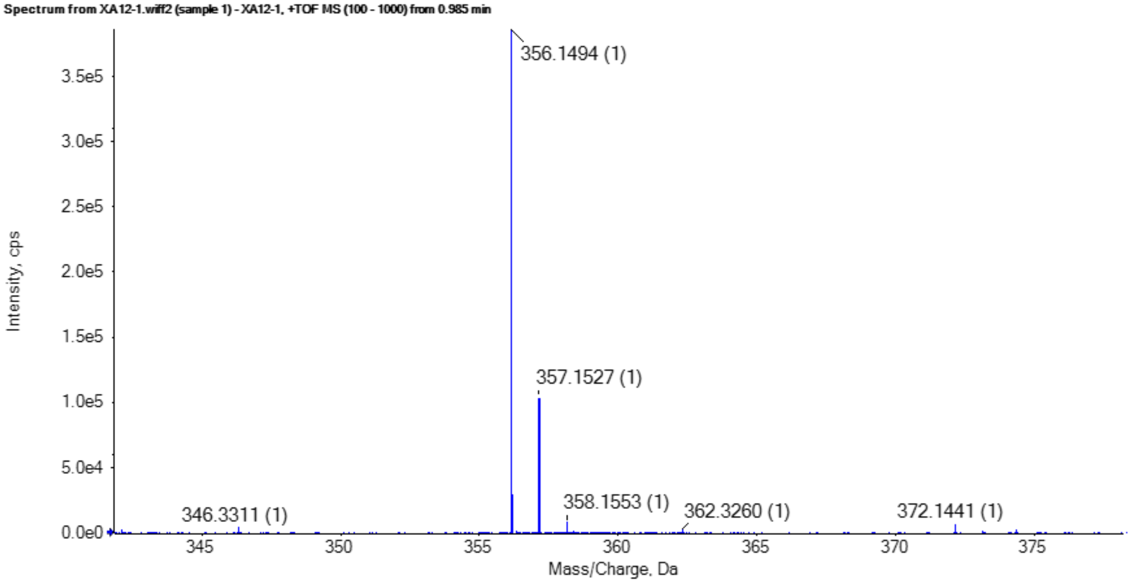


**Formula Calculator Results**

| Measured m/z | Cal m/z | Error(mmu) | Error(ppm) | Ion Formula | Ion |
|---|---|---|---|---|---|
| 356.1494 | 356.1493 | 0.1 | 0.3 | C20H22NO5 | $[M+H]^+$ |

HRESIMS spectrum of compound **11f**

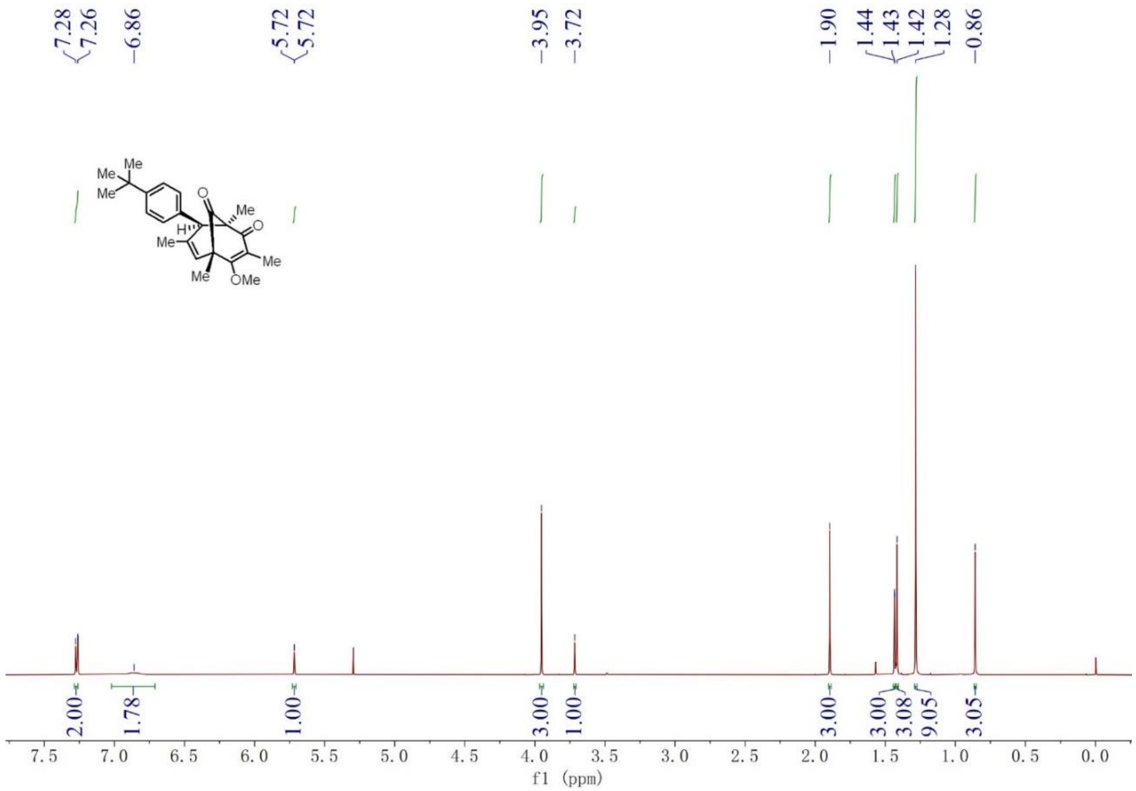


$^{1}$H NMR spectrum of compound **11g** (600MHz, $CDCl_3$)

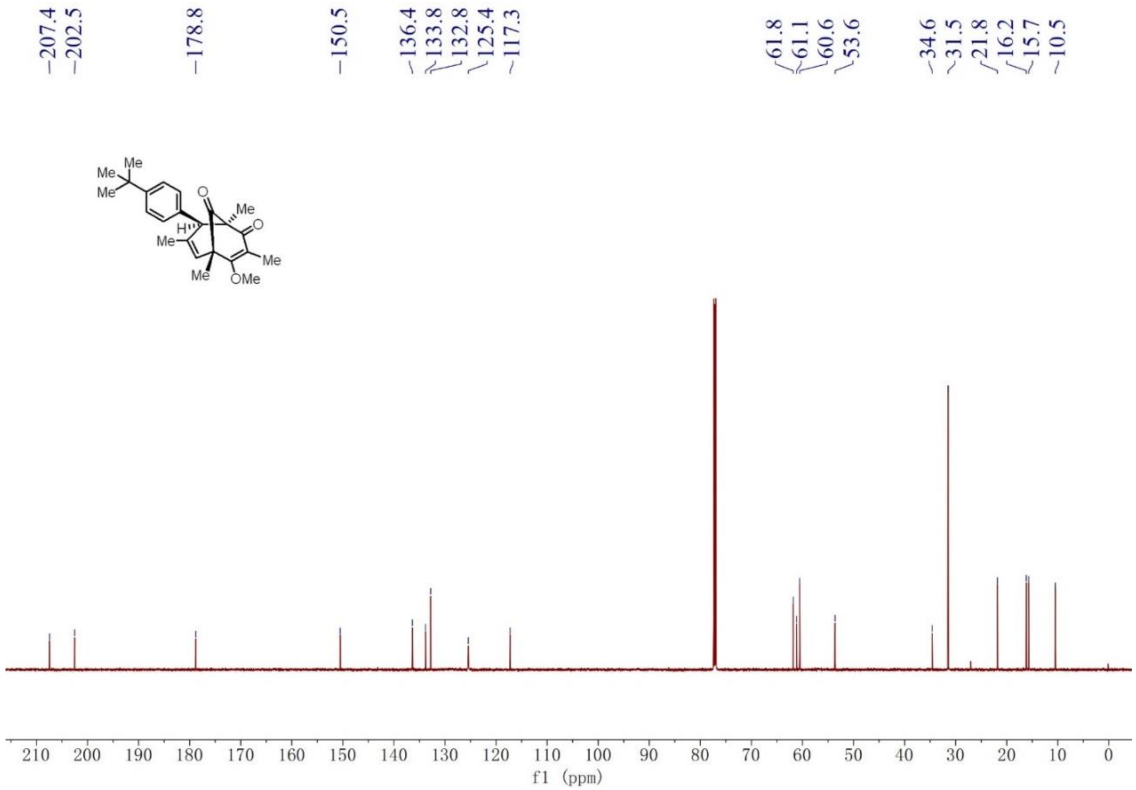


$^{13}$C NMR spectrum of compound **11g** (151MHz, $CDCl_3$)

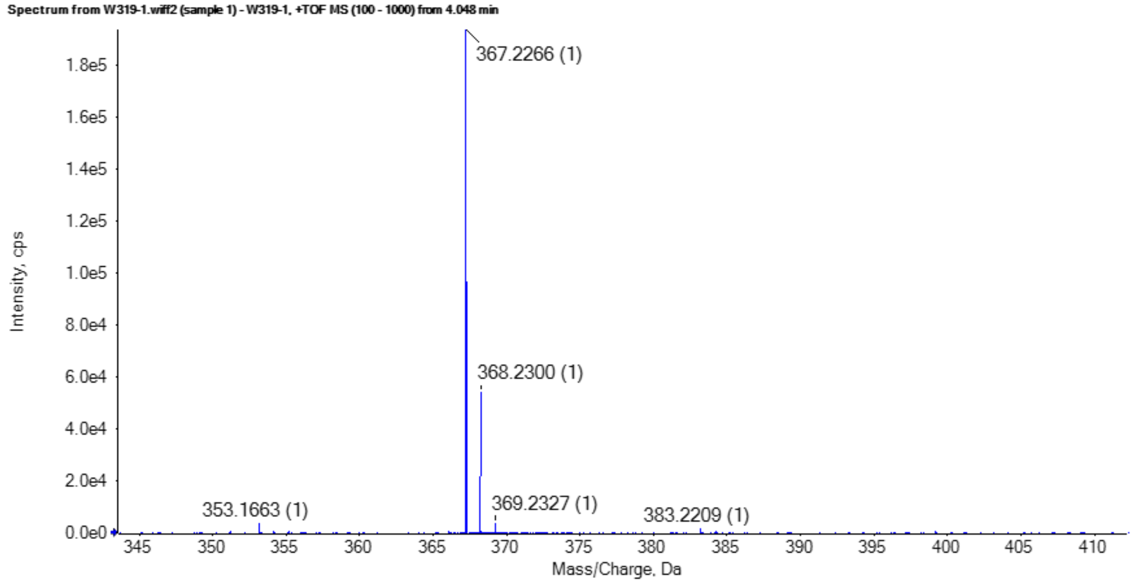


## Formula Calculator Results

| Measured m/z | Cal m/z | Error(mmu) | Error(ppm) | Ion Formula | Ion |
|---|---|---|---|---|---|
| 367.2226 | 367.2268 | -0.2 | -0.6 | C24H31O3 | $[M+H]^+$ |

HRESIMS spectrum of compound **11g**

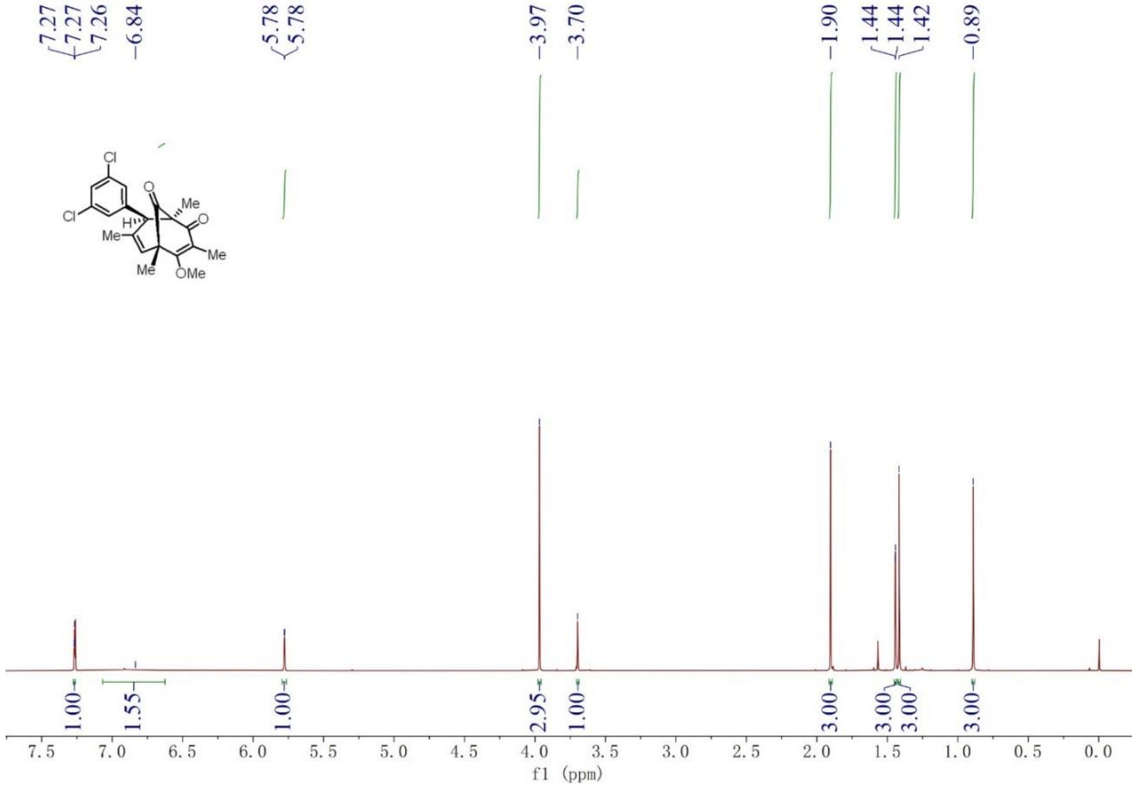


$^{1}$H NMR spectrum of compound **11h** (600MHz, $CDCl_3$)

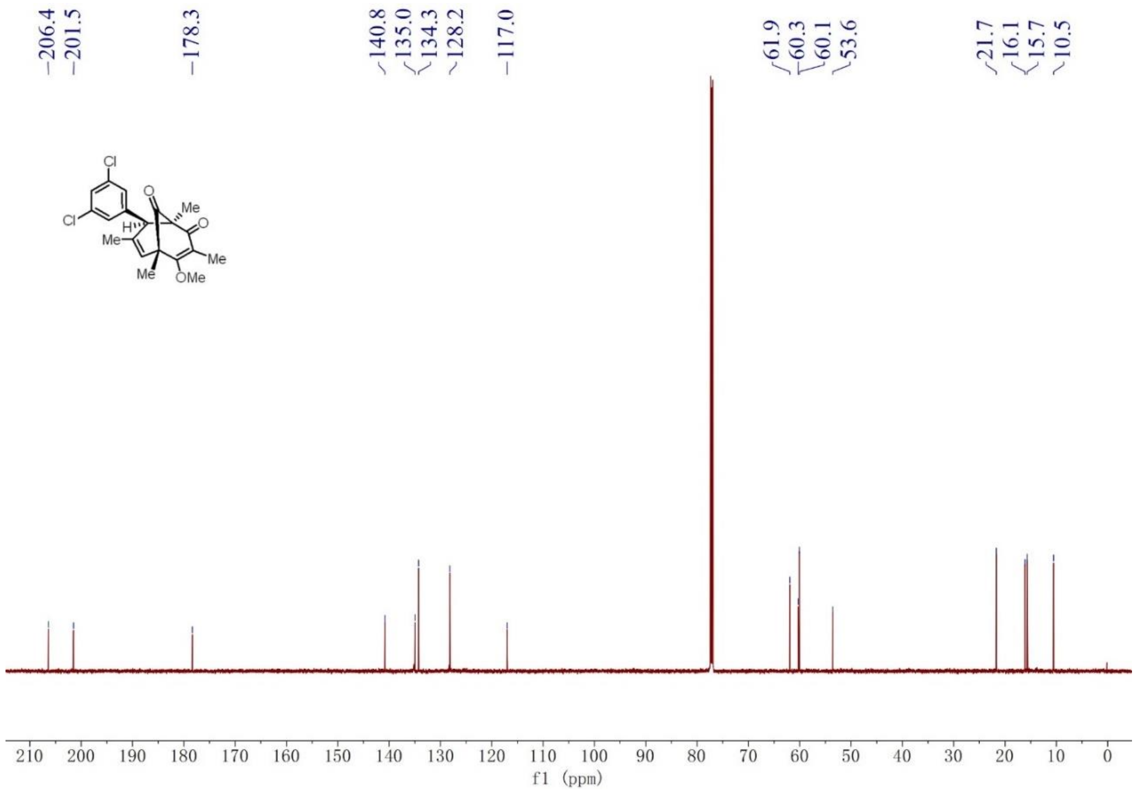


$^{13}$C NMR spectrum of compound **11h** (151MHz, $CDCl_3$)

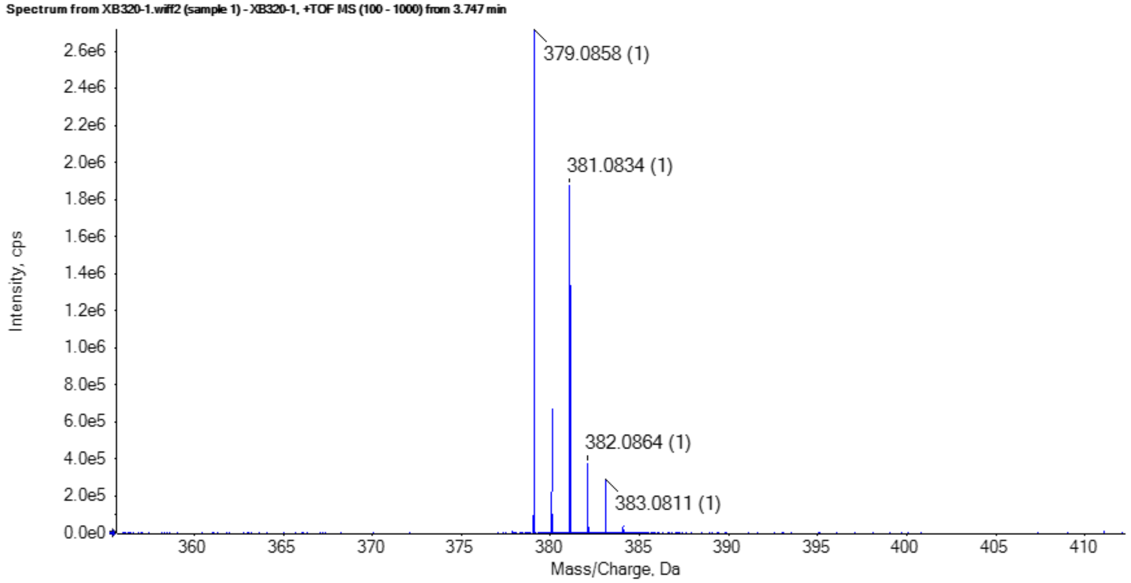


## Formula Calculator Results

| Measured m/z | Cal m/z | Error(mmu) | Error(ppm) | Ion Formula | Ion |
|---|---|---|---|---|---|
| 379.0858 | 379.0862 | -0.4 | -1.1 | C20H21Cl2O3 | $[M+H]^+$ |

HRESIMS spectrum of compound **11h**

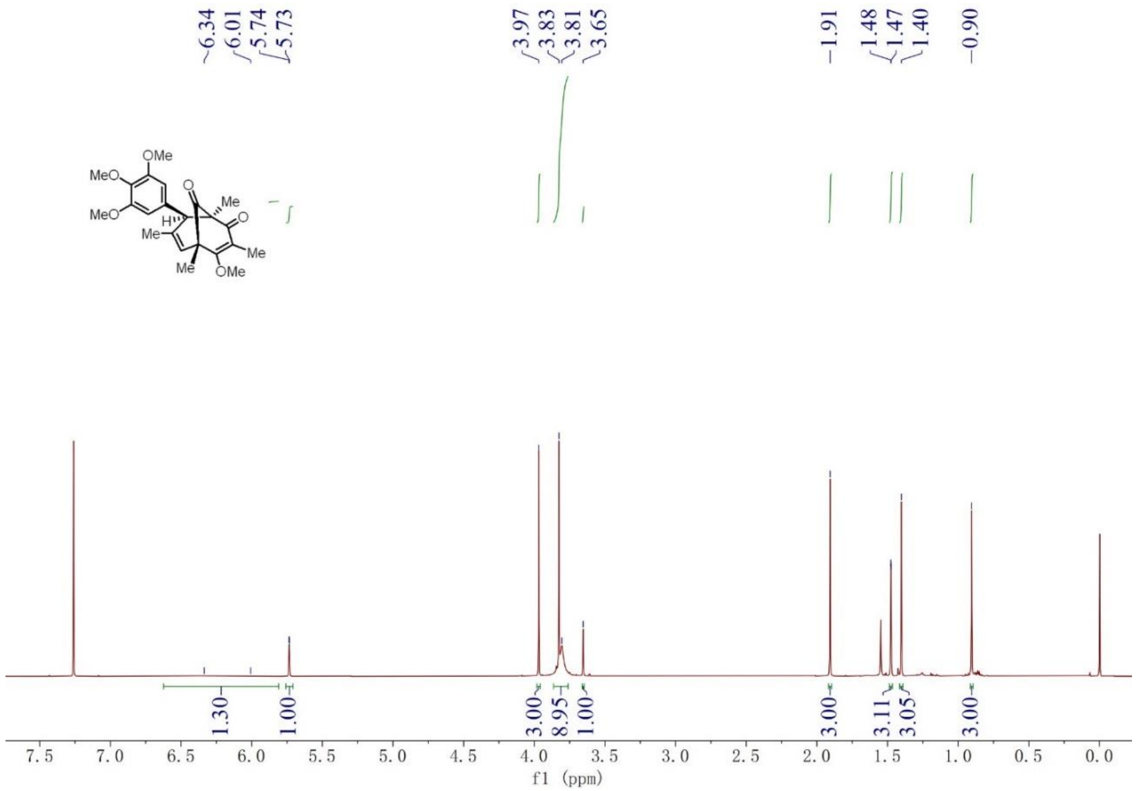


$^{1}$H NMR spectrum of compound **11i** (600MHz, $CDCl_3$)

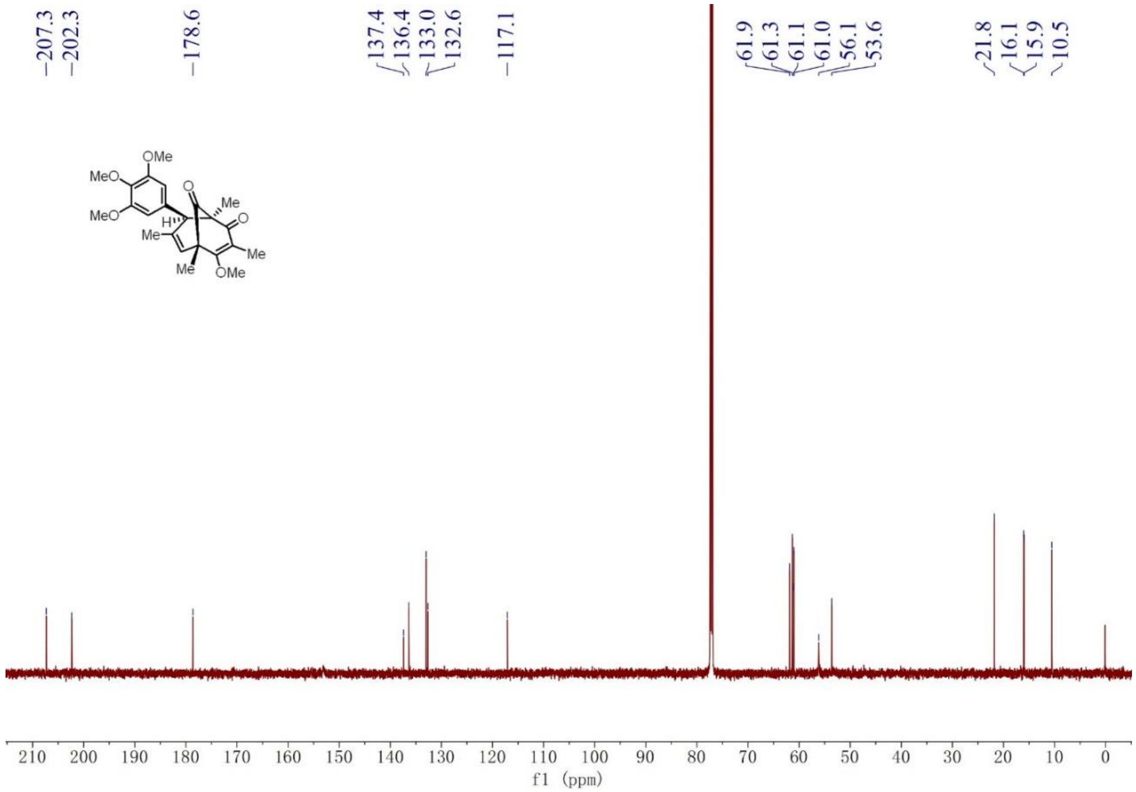


$^{13}$C NMR spectrum of compound **11i** (151MHz, $CDCl_3$)

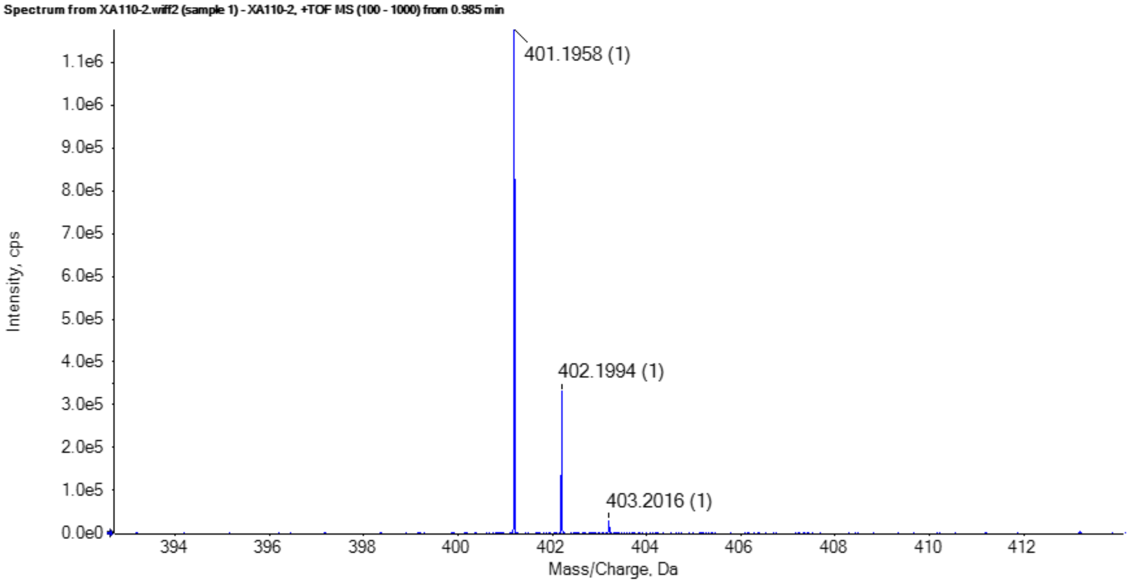


**Formula Calculator Results**

| Measured m/z | Cal m/z | Error(mmu) | Error(ppm) | Ion Formula | Ion |
|---|---|---|---|---|---|
| 401.1958 | 401.1959 | -0.1 | -0.2 | C23H29O6 | $[M+H]^+$ |

HRESIMS spectrum of compound **11i**

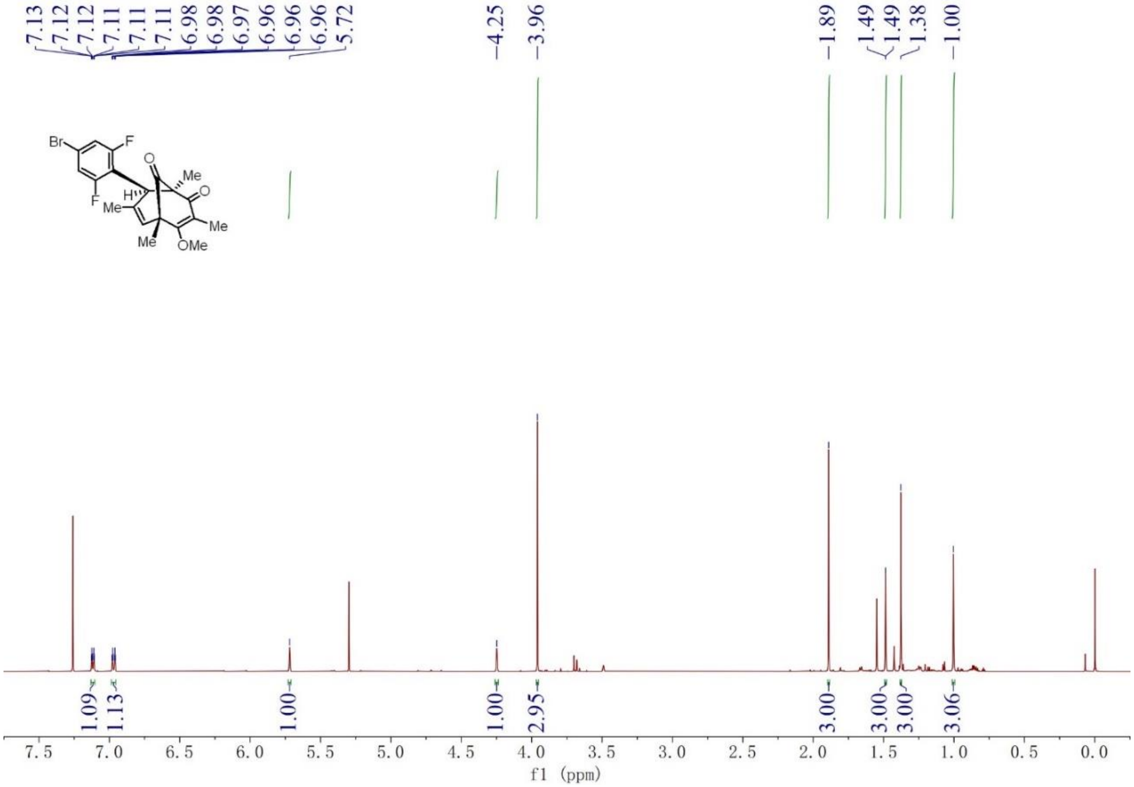


$^{1}$H NMR spectrum of compound **11j** (600MHz, $CDCl_3$)

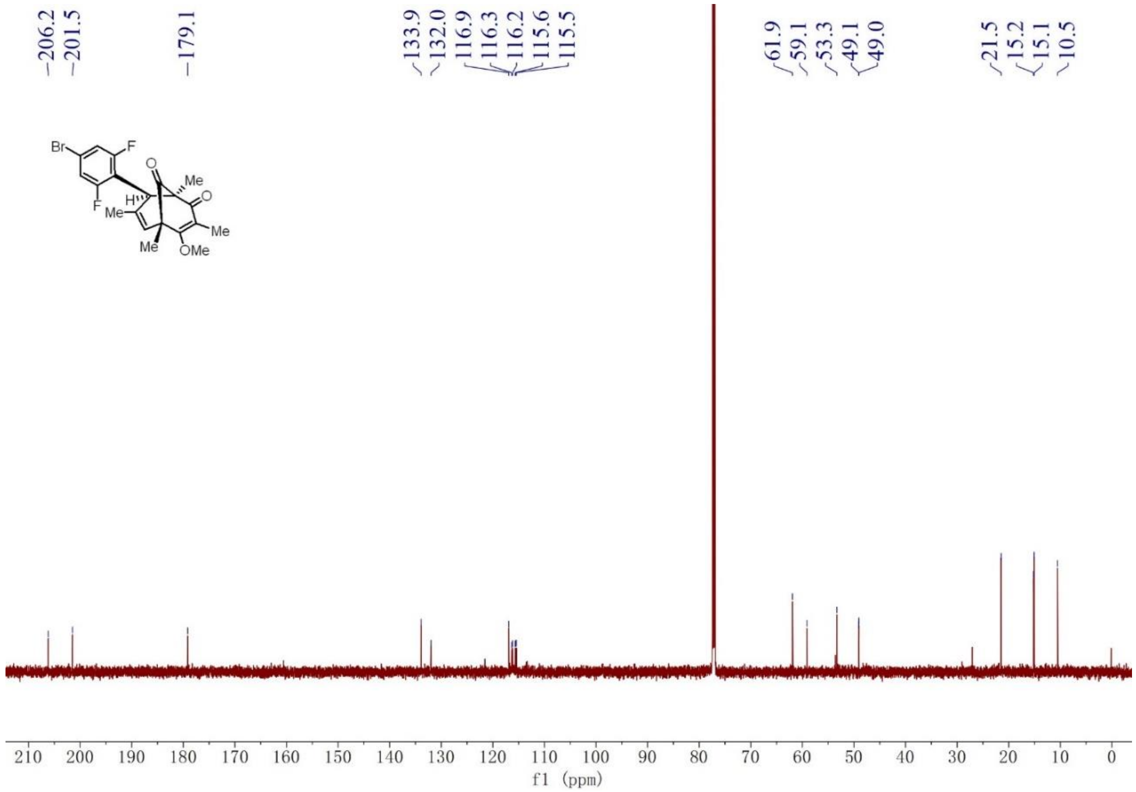

$^{13}C$ NMR spectrum of compound **11j** (151MHz, $CDCl_3$)

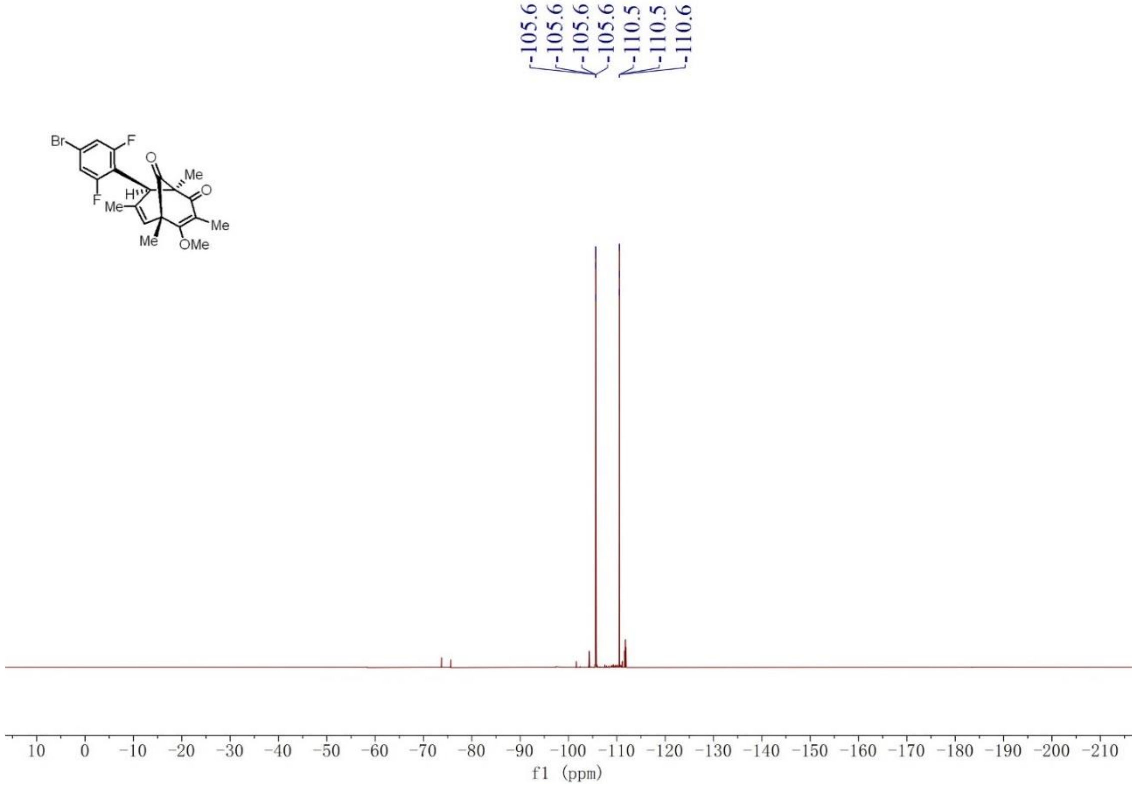

$^{19}F$ NMR spectrum of compound **11j** (565MHz, $CDCl_3$)

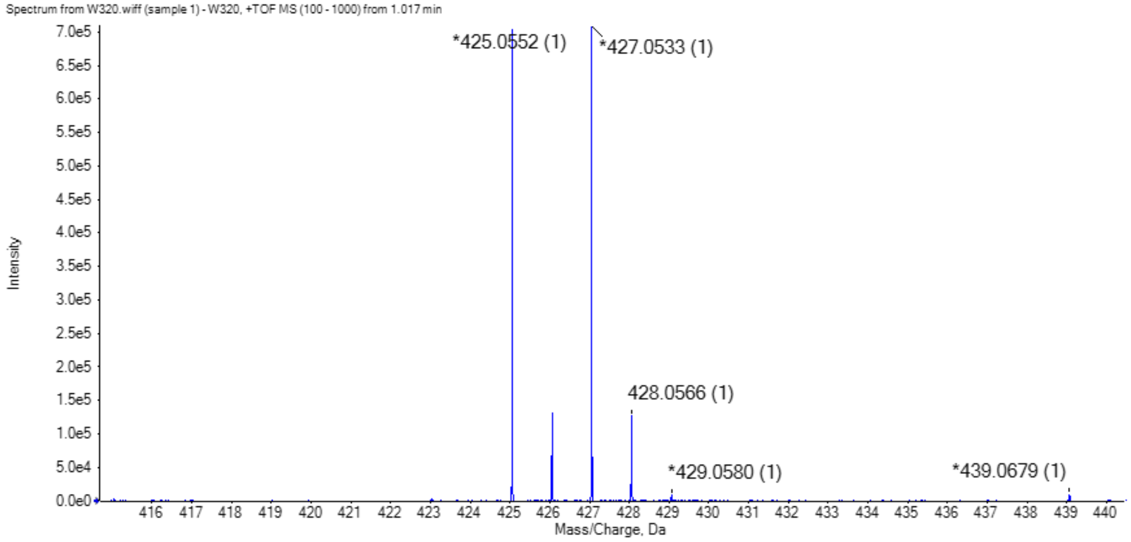


**Formula Calculator Results**

| Measured m/z | Cal m/z | Error(mmu) | Error(ppm) | Ion Formula | Ion |
|---|---|---|---|---|---|
| 425.05516 | 425.05584 | -0.7 | -1.6 | C20H20BrF2O3 | $[M+H]^+$ |

HRESIMS spectrum of compound **11j**

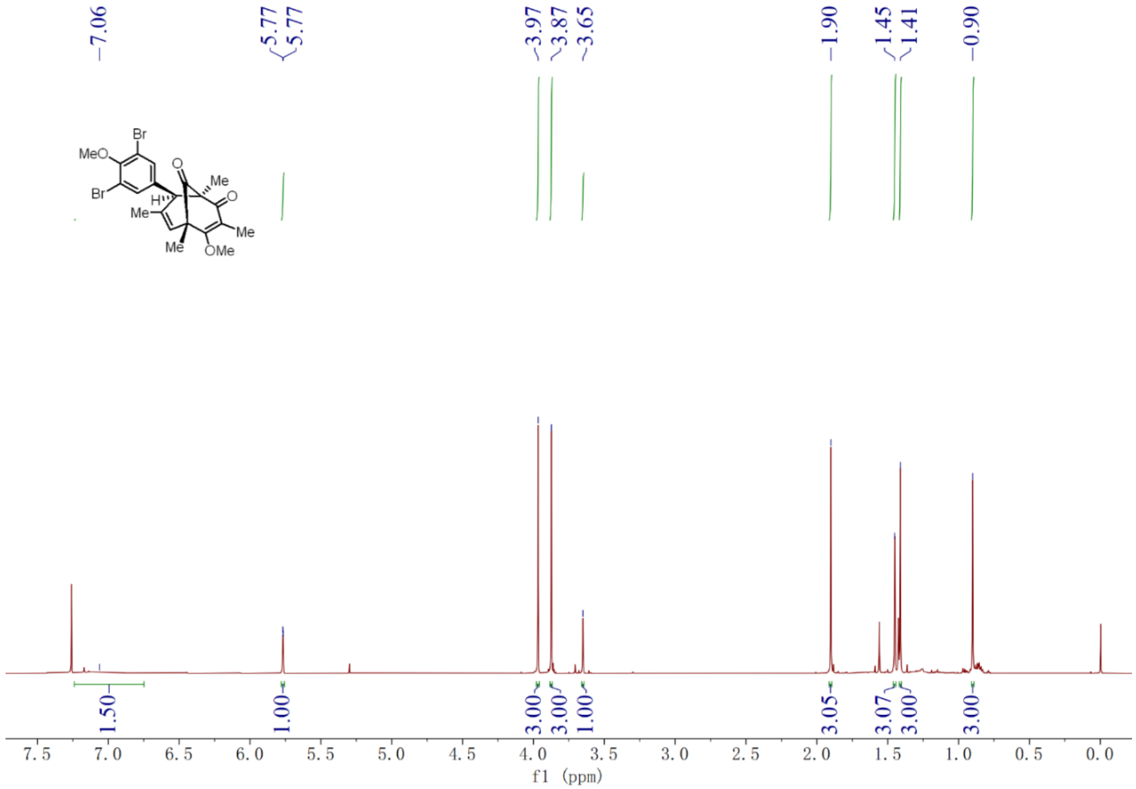


$^1$H NMR spectrum of compound **11k** (600MHz, $CDCl_3$)

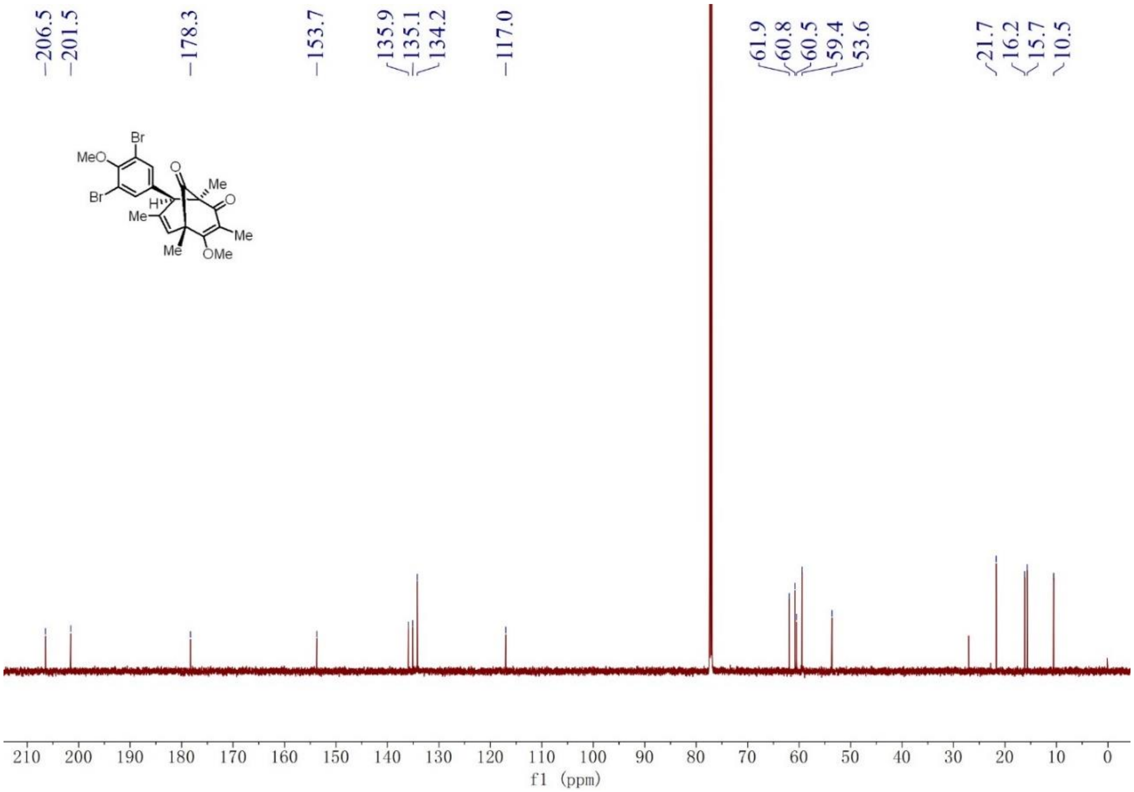


$^{13}C$ NMR spectrum of compound **11k** (151MHz, $CDCl_3$)

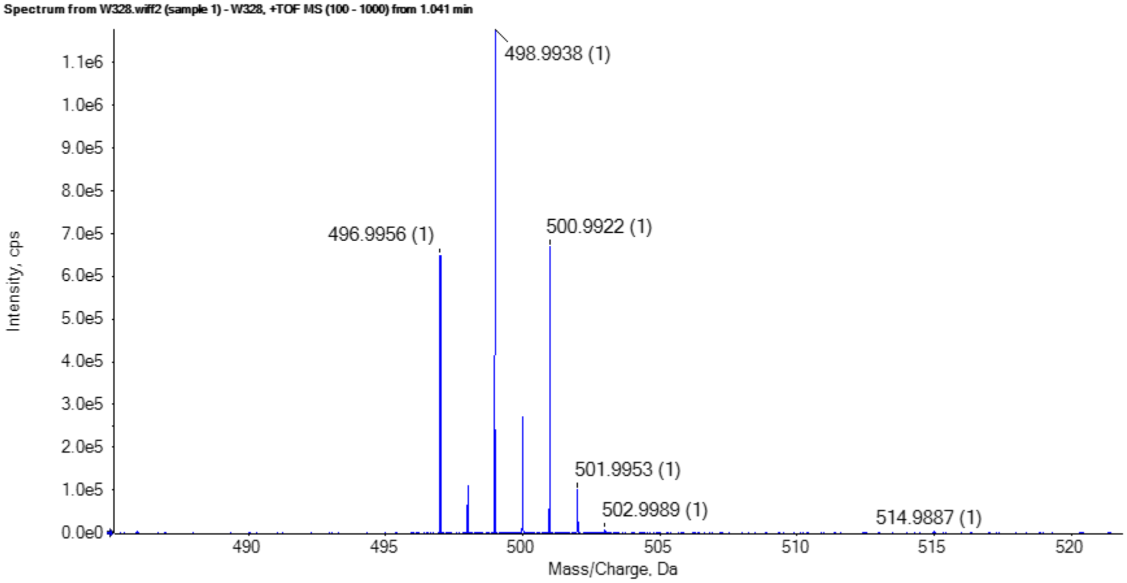


## Formula Calculator Results

| Measured m/z | Cal m/z | Error(mmu) | Error(ppm) | Ion Formula | Ion |
|---|---|---|---|---|---|
| 496.9956 | 496.9958 | -0.2 | -0.3 | C21H23Br2O4 | $[M+H]^+$ |

HRESIMS spectrum of compound **11k**

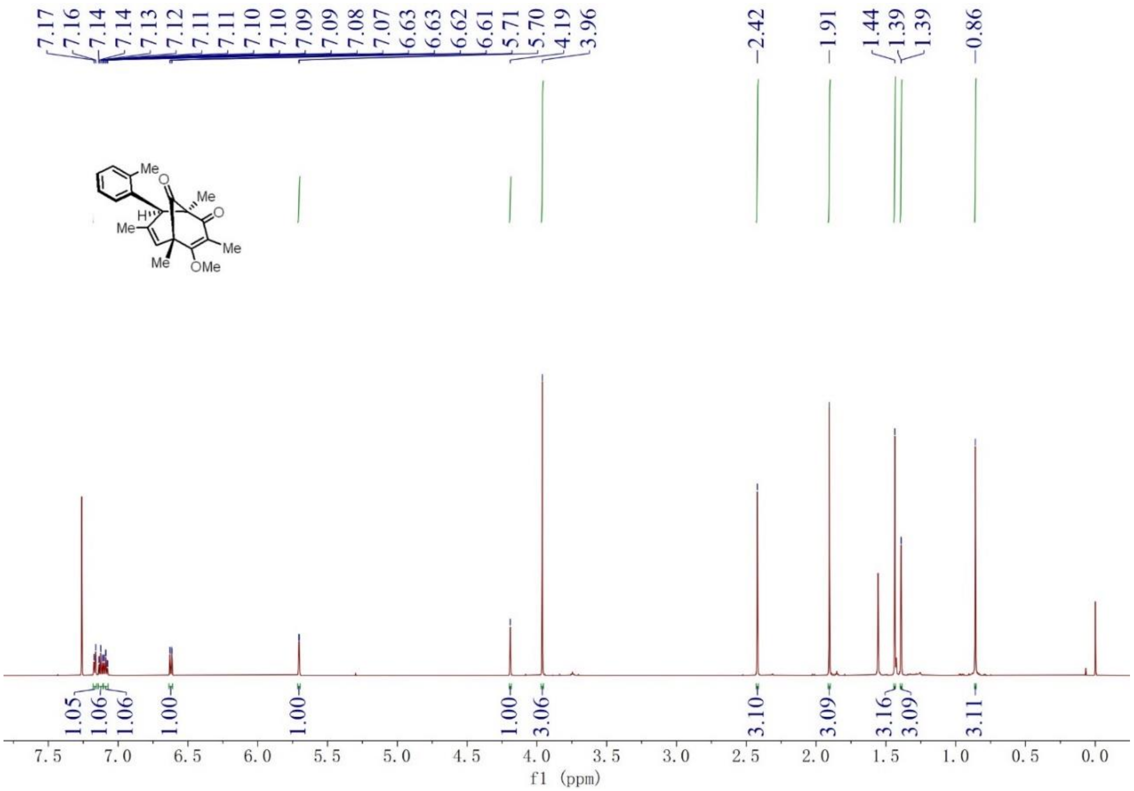


$^{1}$H NMR spectrum of compound **11l** (600MHz, $CDCl_3$)

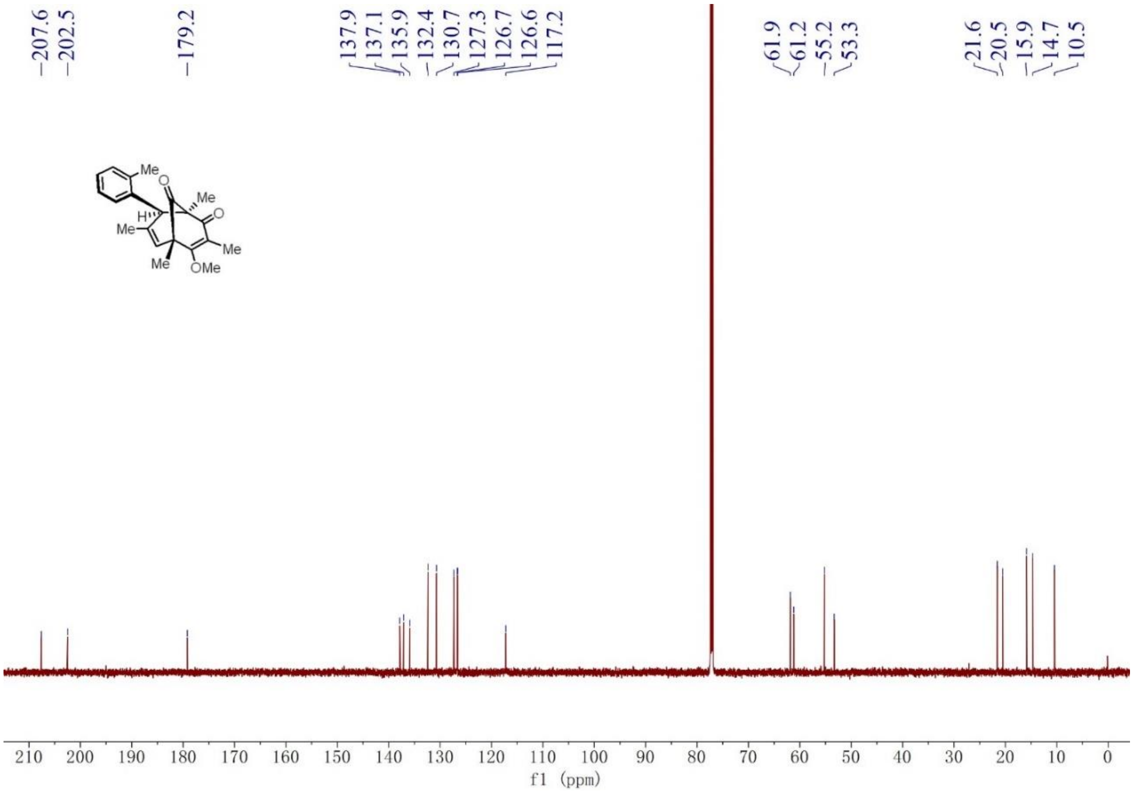


$^{13}$C NMR spectrum of compound **11l** (151MHz, $CDCl_3$)

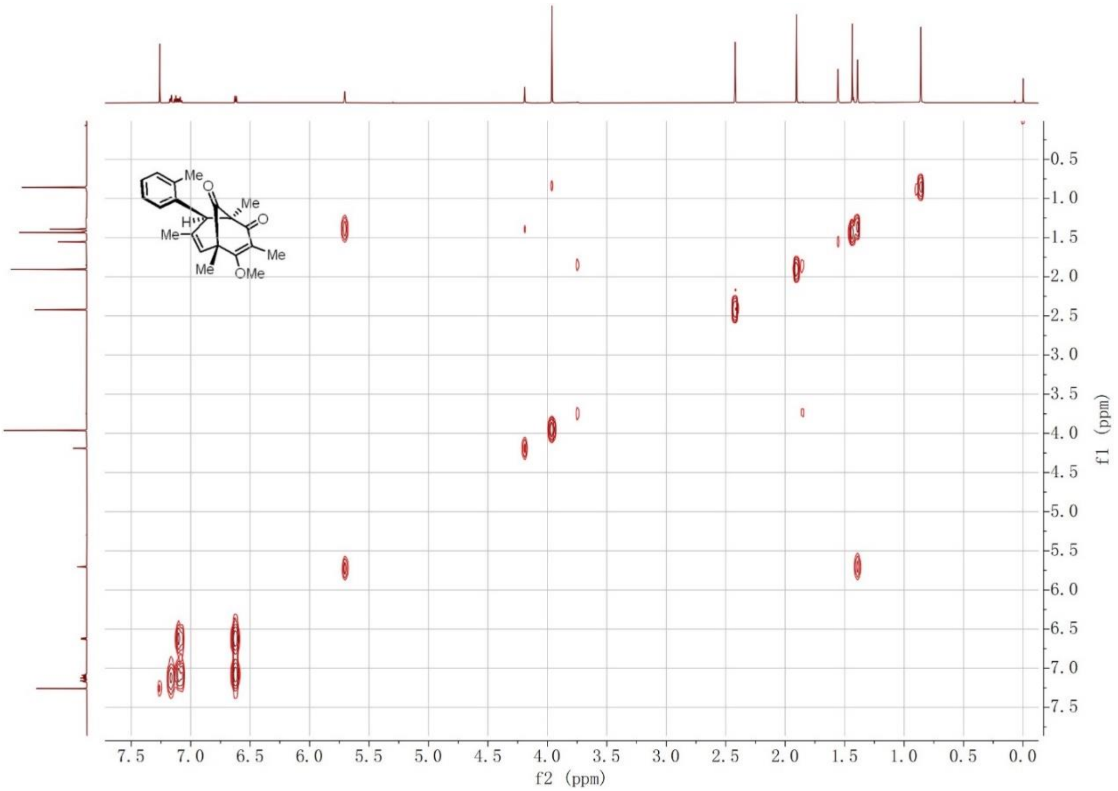

$^1H$-$^1H$ COSY spectrum of compound **11l** (600 MHz, $CDCl_3$)

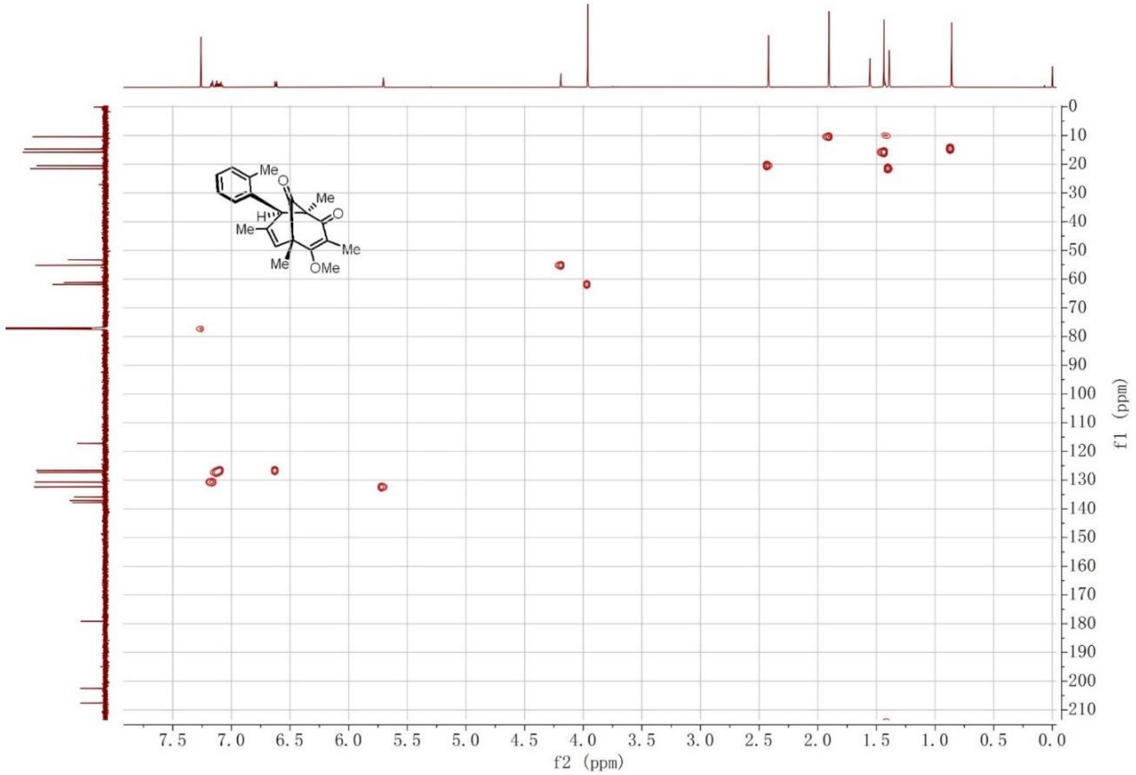

HSQC spectrum of compound **11l** (600 MHz, $CDCl_3$)

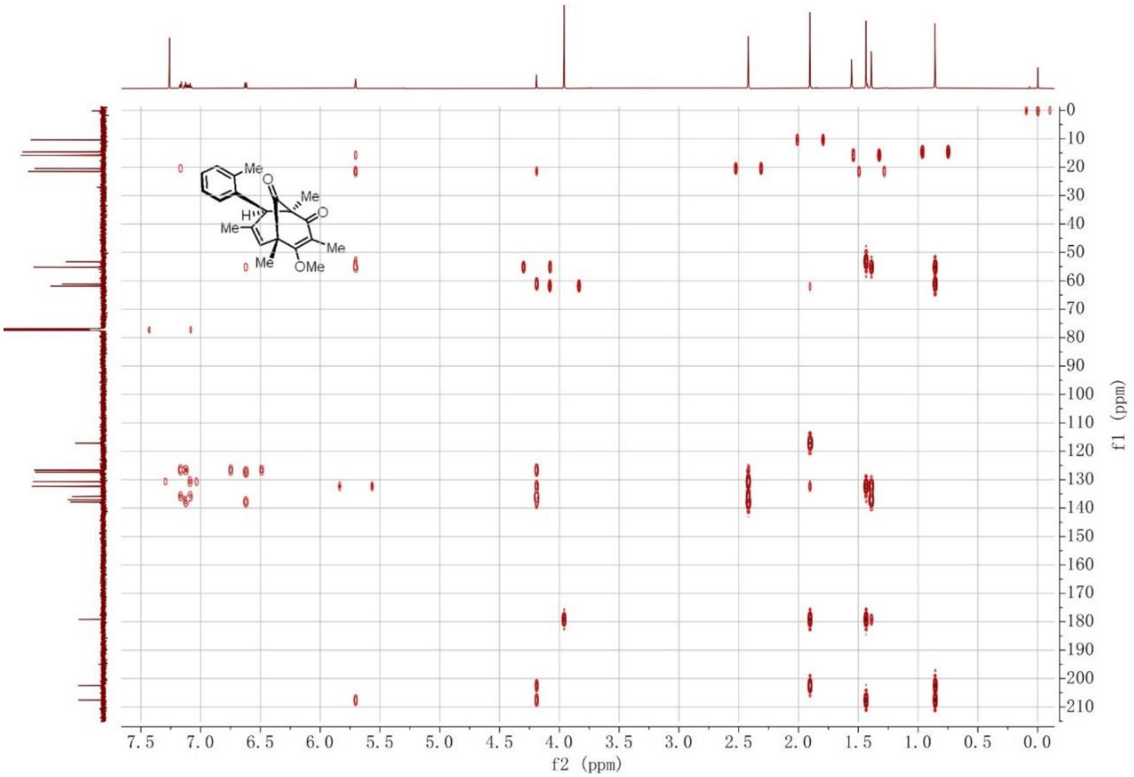

HMBC spectrum of compound **11l** (600 MHz, $CDCl_3$)

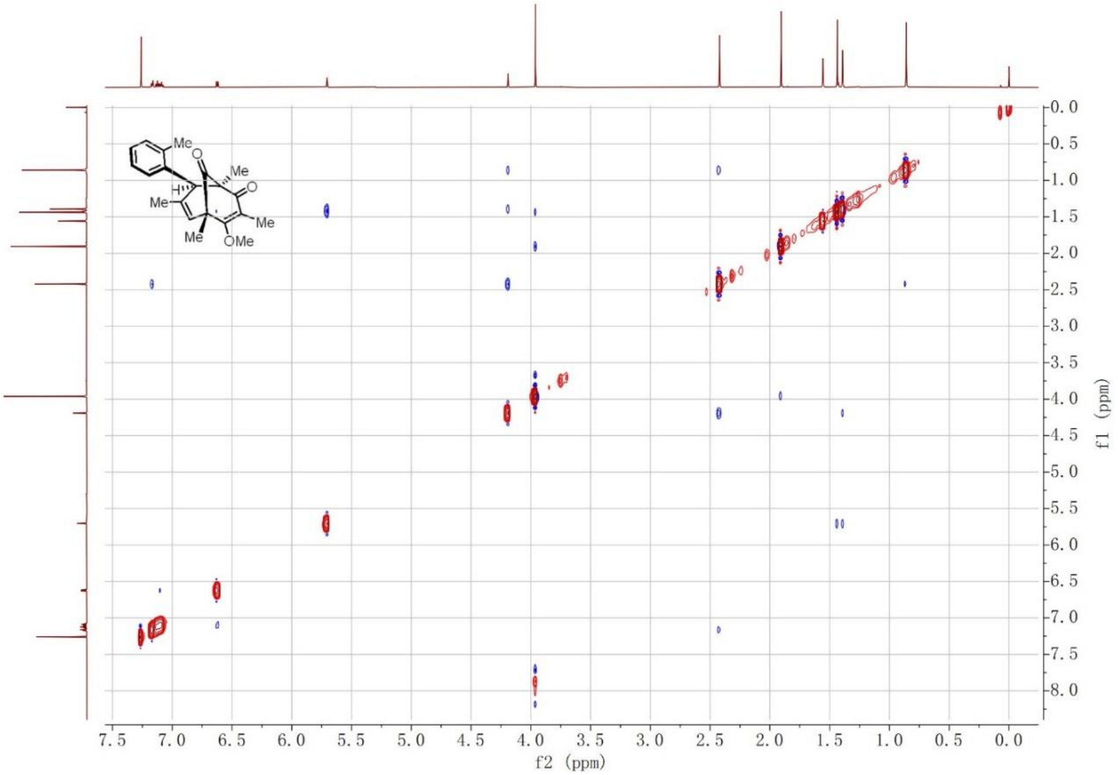

NOESY spectrum of compound **11l** (600 MHz, $CDCl_3$)

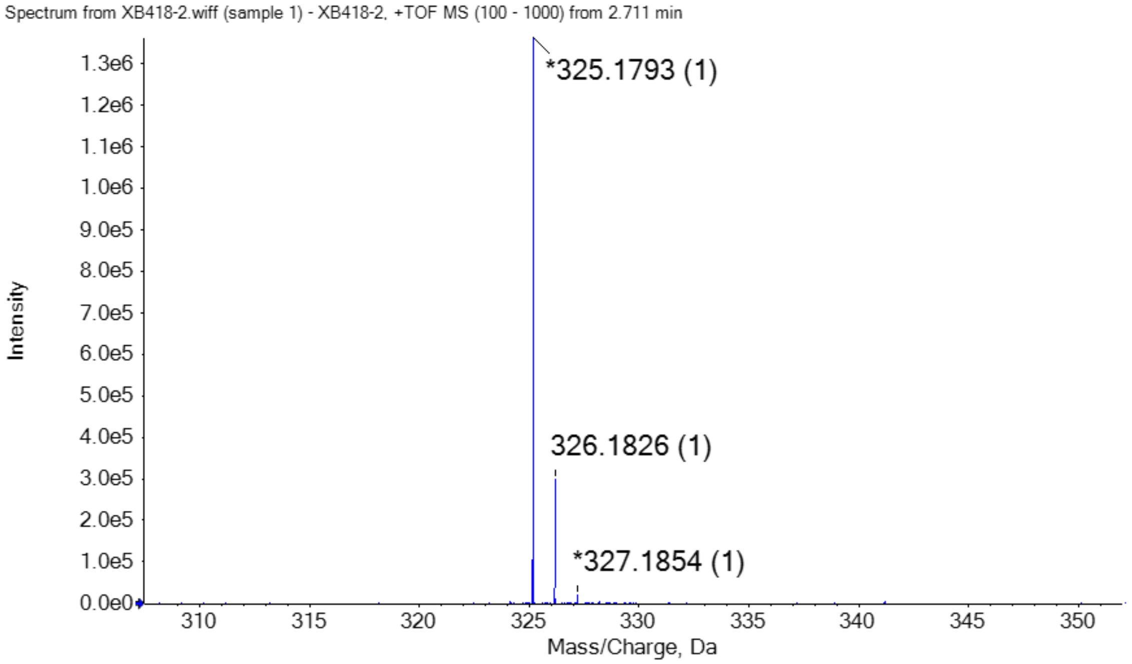


**Formula Calculator Results**

| Measured m/z | Cal m/z | Error(mmu) | Error(ppm) | Ion Formula | Ion |
|---|---|---|---|---|---|
| 325.1793 | 325.1798 | -0.5 | -1.6 | C21H25O3 | $[M+H]^+$ |

HRESIMS spectrum of compound **11l**

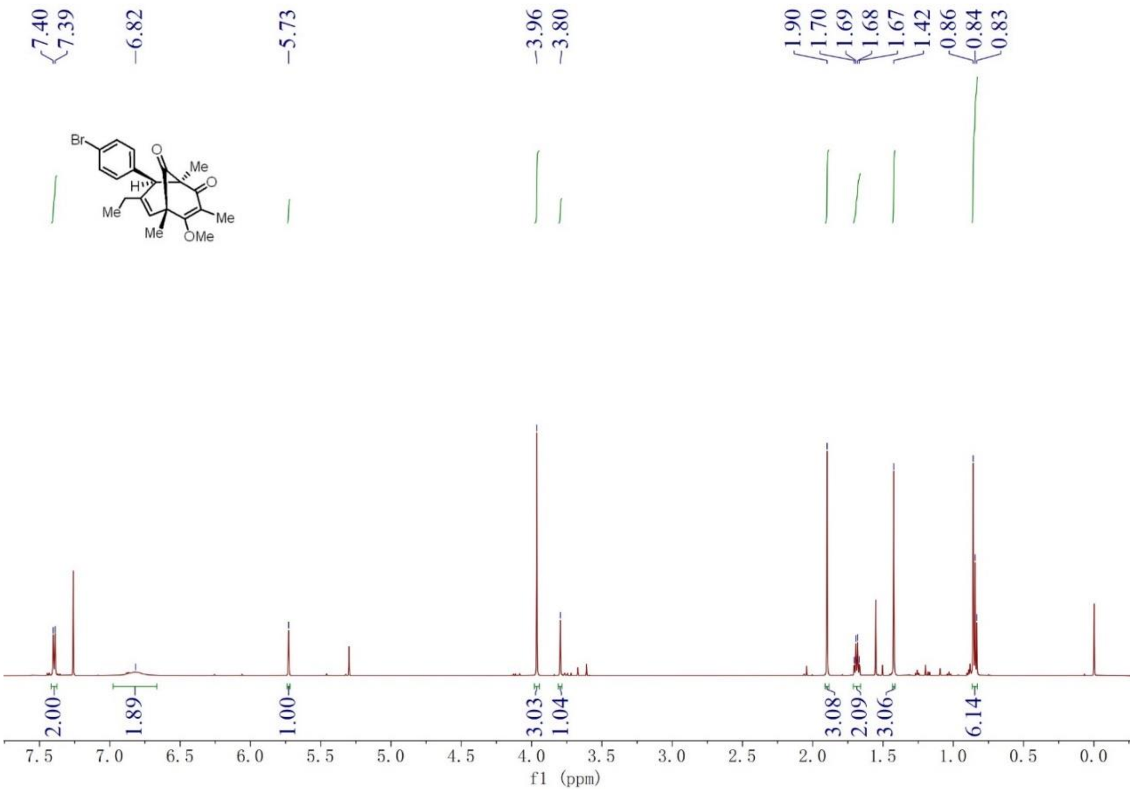


$^1$H NMR spectrum of compound **11m** (600MHz, $CDCl_3$)

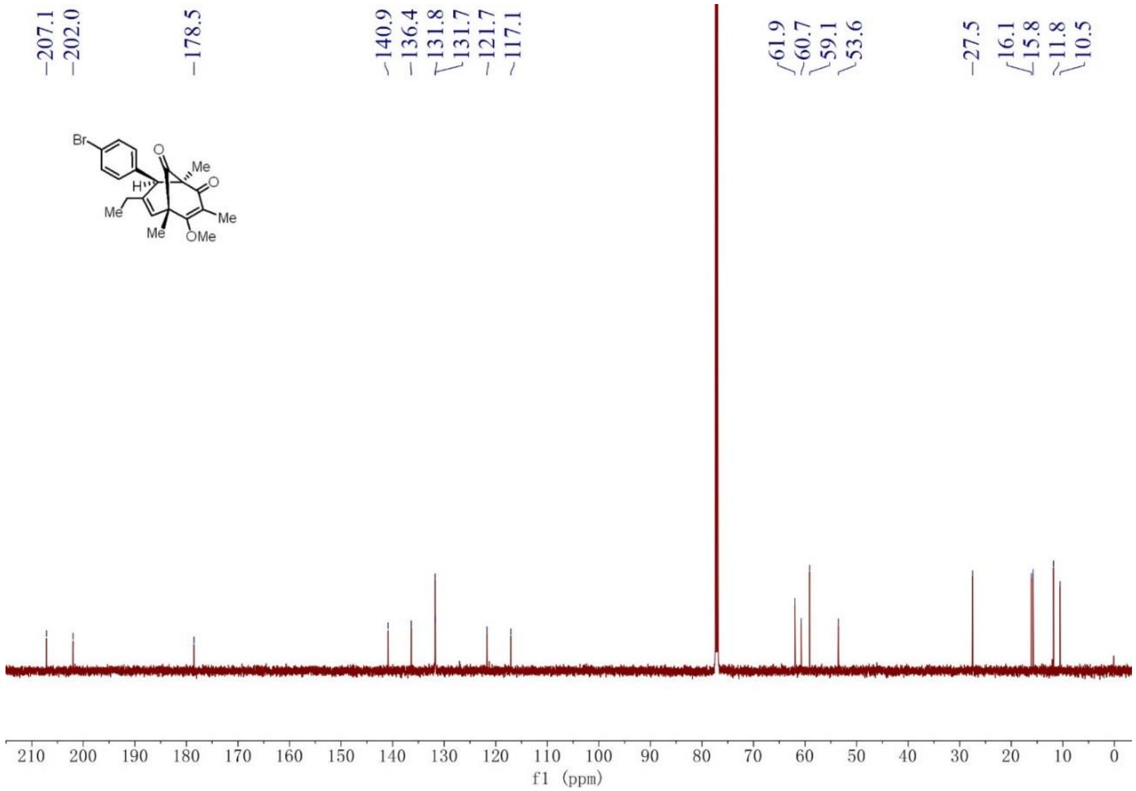


$^{13}$C NMR spectrum of compound **11m** (151MHz, $CDCl_3$)

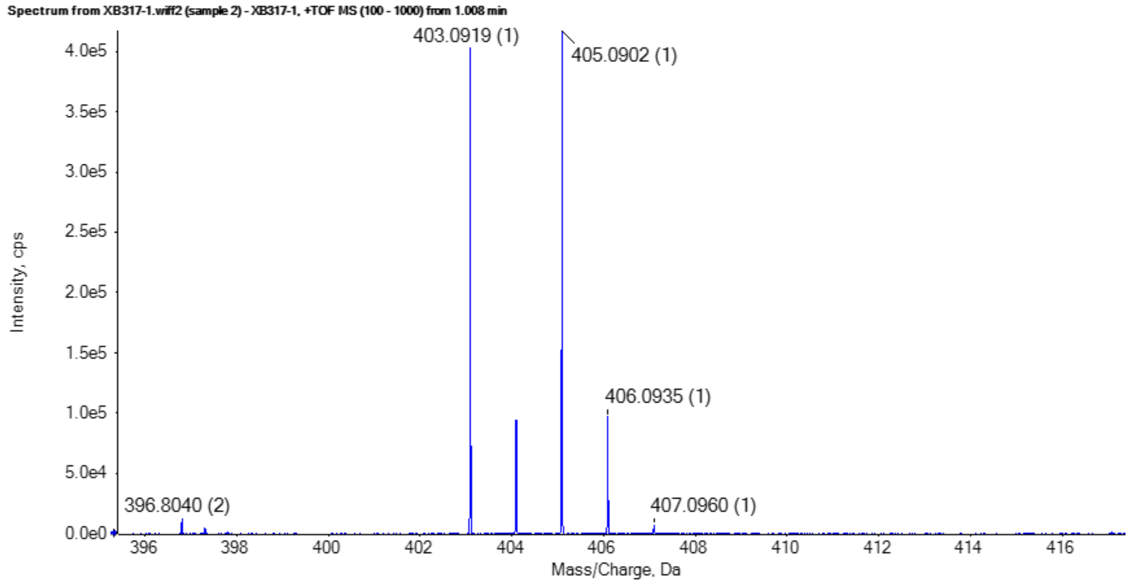


**Formula Calculator Results**

| Measured m/z | Cal m/z | Error(mmu) | Error(ppm) | Ion Formula | Ion |
|---|---|---|---|---|---|
| 403.0919 | 403.0903 | 1.6 | 3.9 | C21H24BrO3 | $[M+H]^+$ |

HRESIMS spectrum of compound **11m**

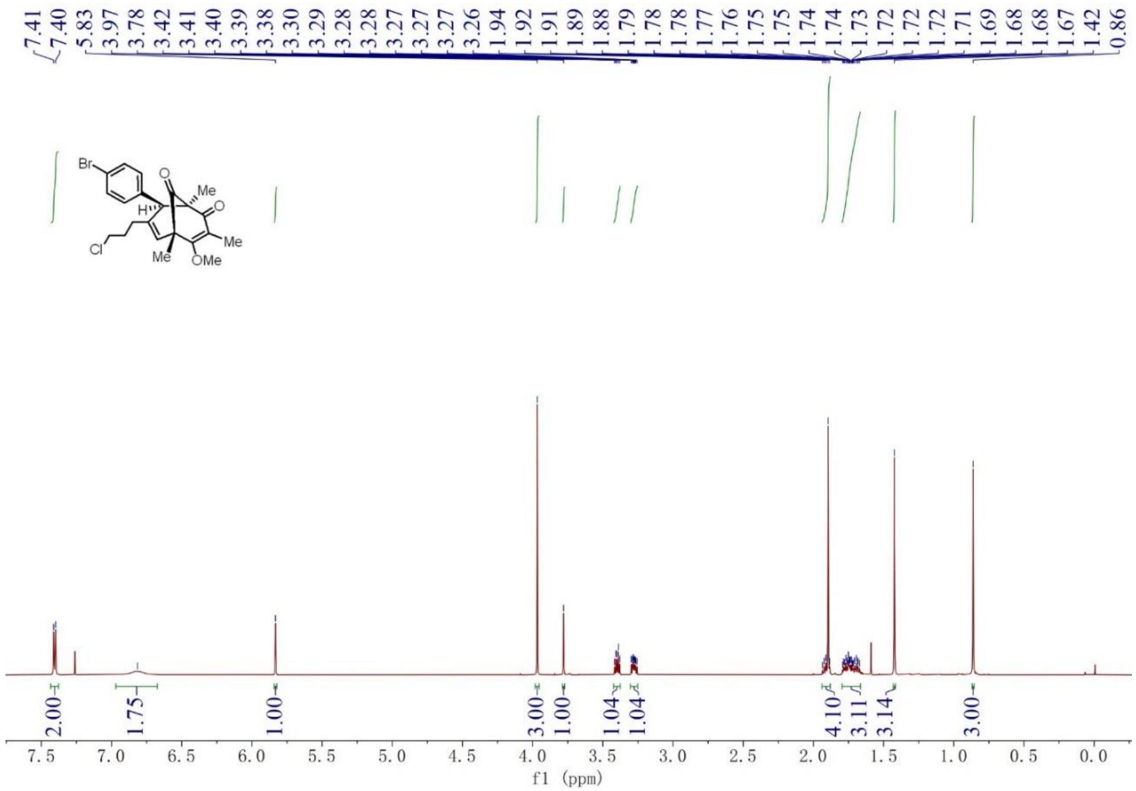


$^{1}$H NMR spectrum of compound **11n** (600MHz, $CDCl_3$)

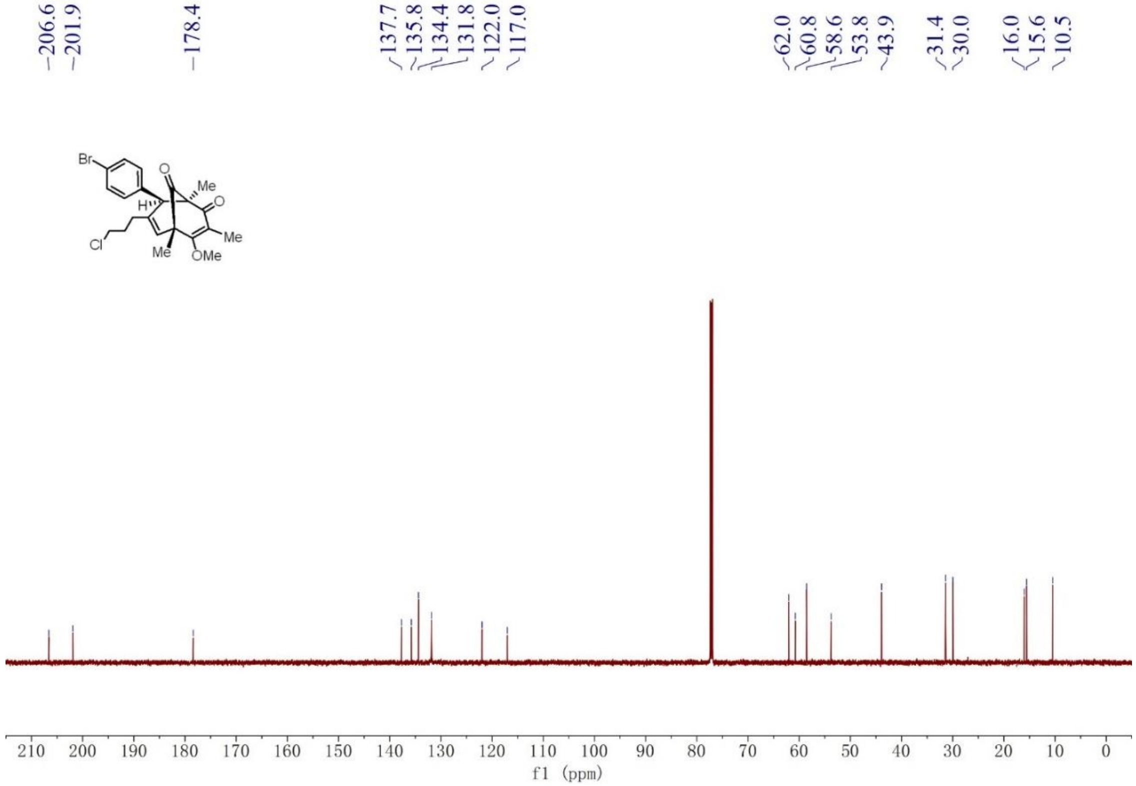


$^{13}$C NMR spectrum of compound **11n** (151MHz, $CDCl_3$)

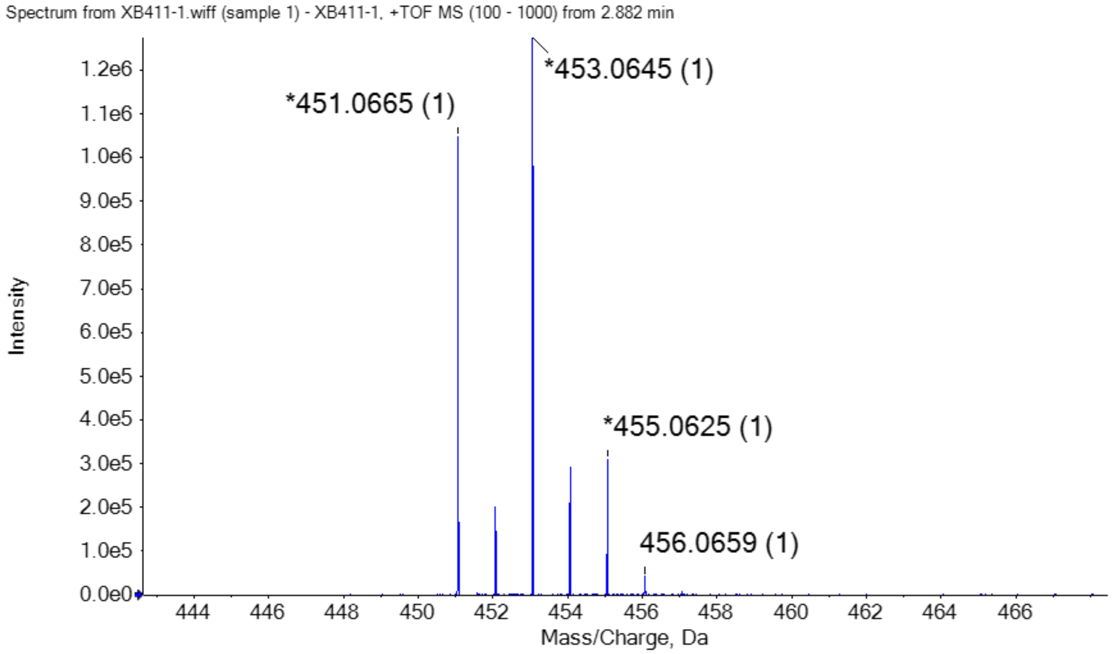


**Formula Calculator Results**

| Measured m/z | Cal m/z | Error(mmu) | Error(ppm) | Ion Formula | Ion |
|---|---|---|---|---|---|
| 451.0665 | 451.0670 | -0.5 | -1.1 | C22H25BrClO3 | $[M+H]^+$ |

HRESIMS spectrum of compound **11n**

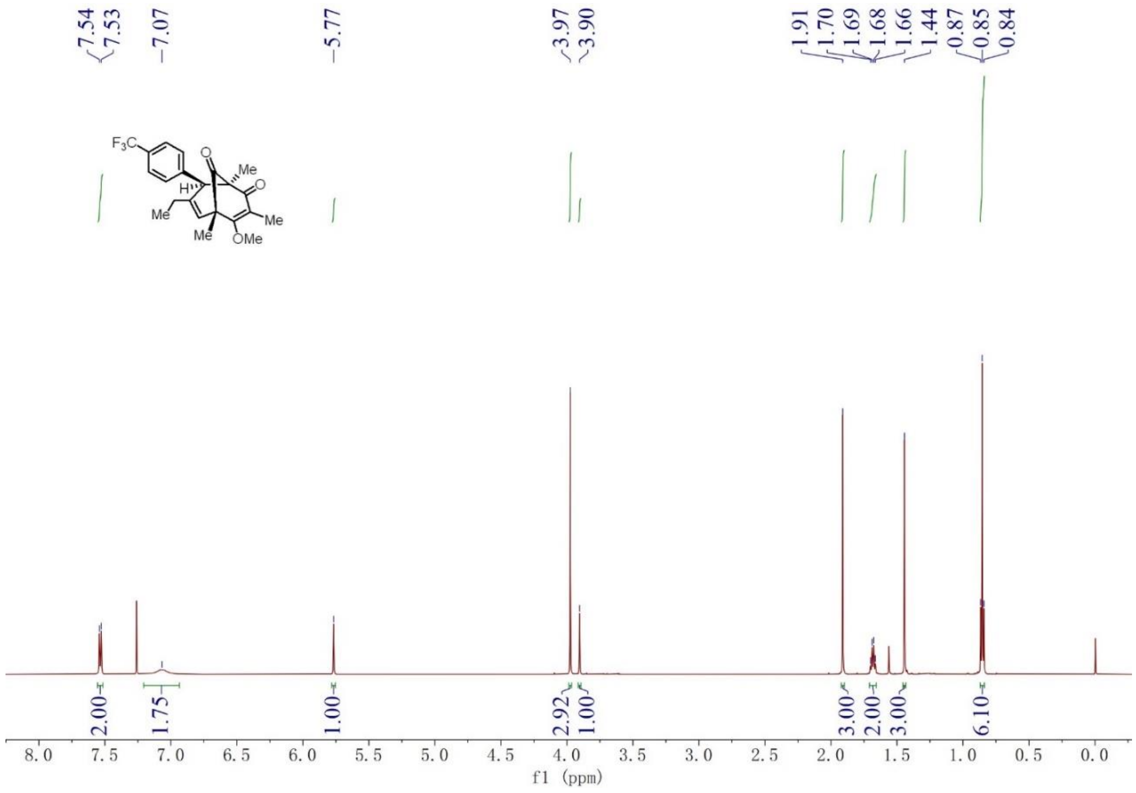


$^1$H NMR spectrum of compound **11o** (600MHz, $CDCl_3$)

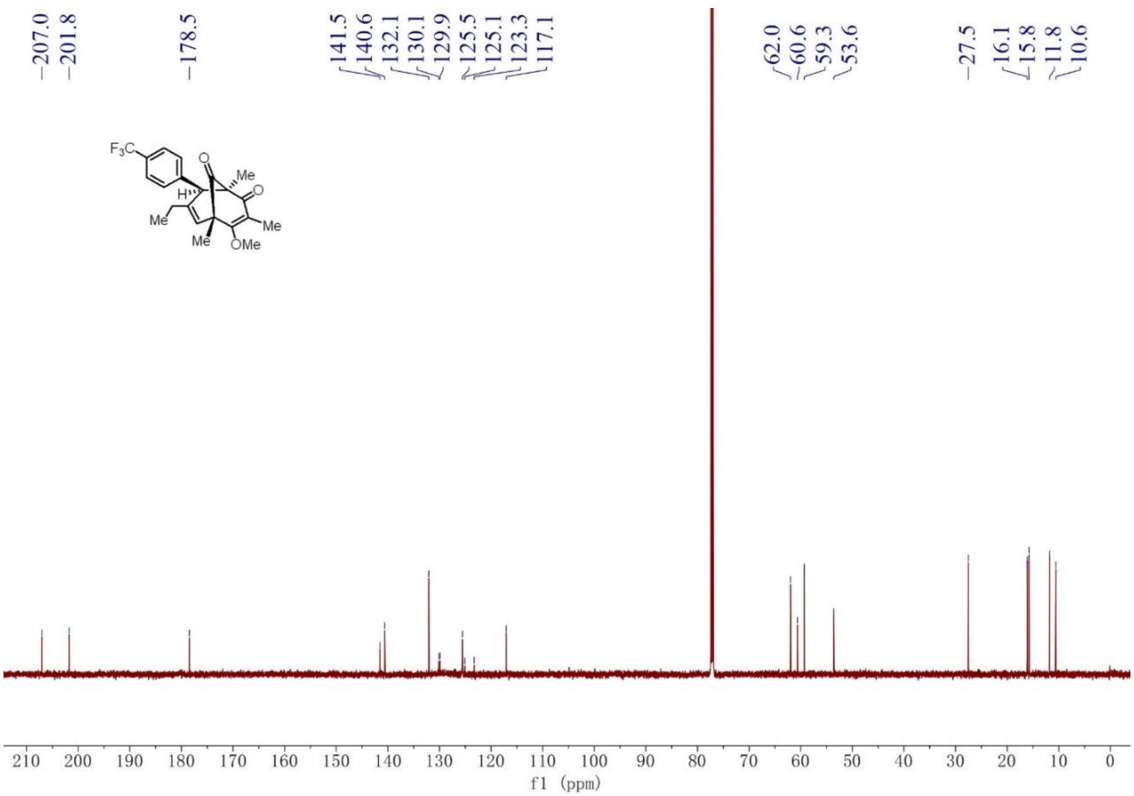

$^{13}$C NMR spectrum of compound **11o** (151MHz, $CDCl_3$)

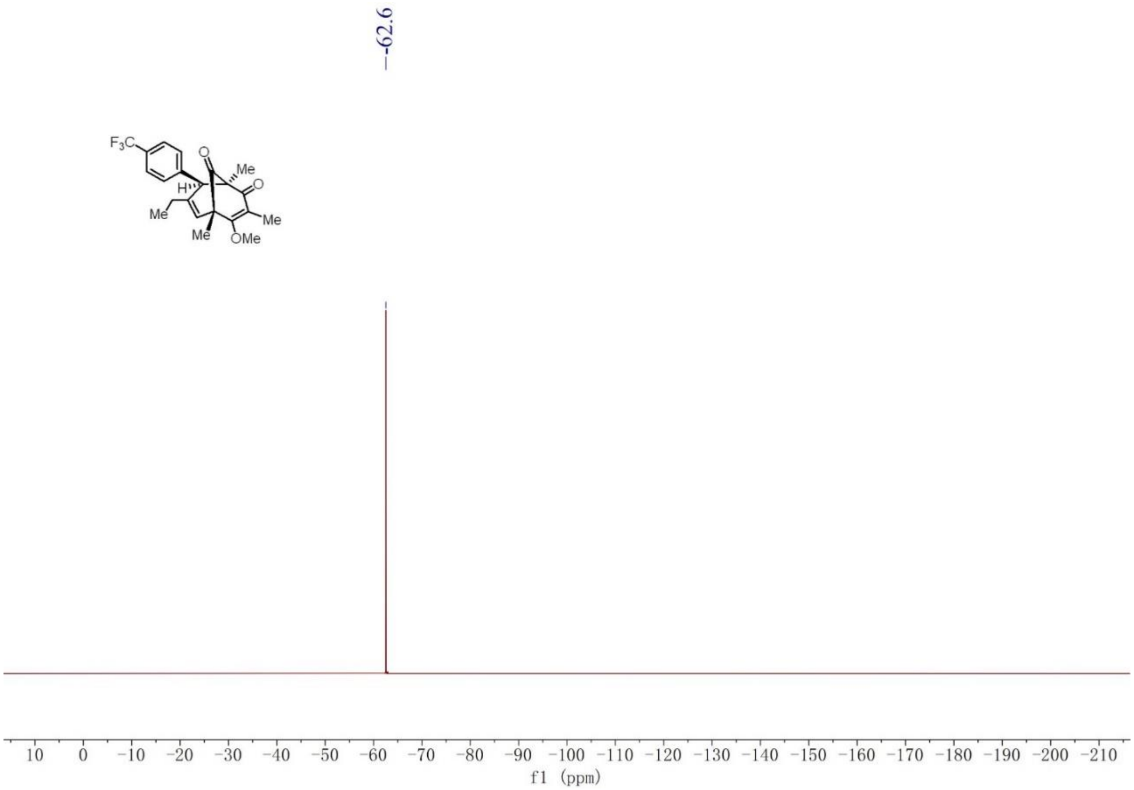

$^{19}$F NMR spectrum of compound **11o** (565MHz, $CDCl_3$)

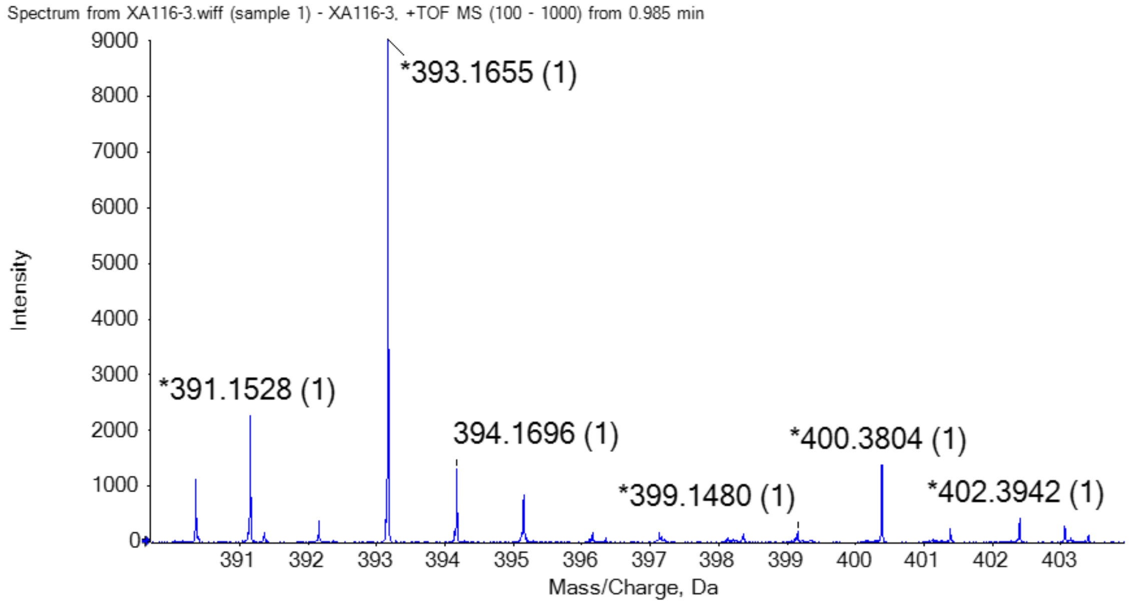


**Formula Calculator Results**

| Measured m/z | Cal m/z | Error(mmu) | Error(ppm) | Ion Formula | Ion |
|---|---|---|---|---|---|
| 393.1655 | 393.1672 | -1.7 | -4.3 | C22H24F3O3 | $[M+H]^+$ |

HRESIMS spectrum of compound **11o**

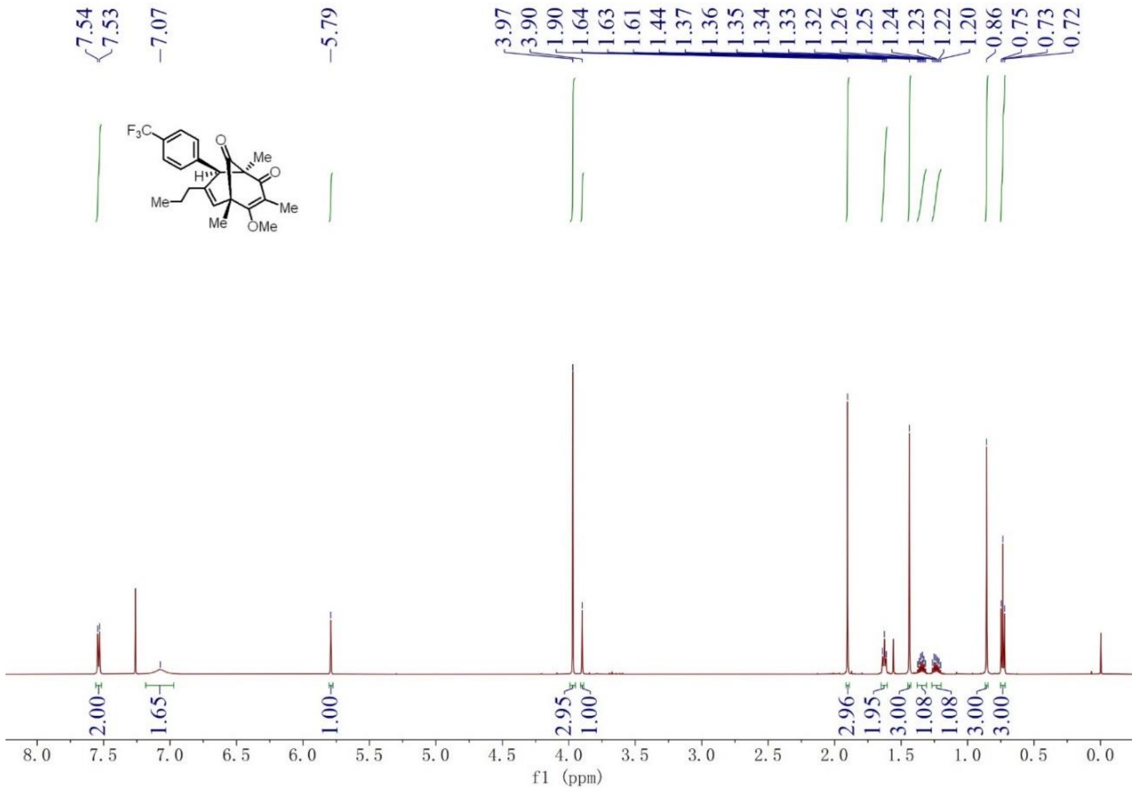


$^1$H NMR spectrum of compound **11p** (600MHz, $CDCl_3$)

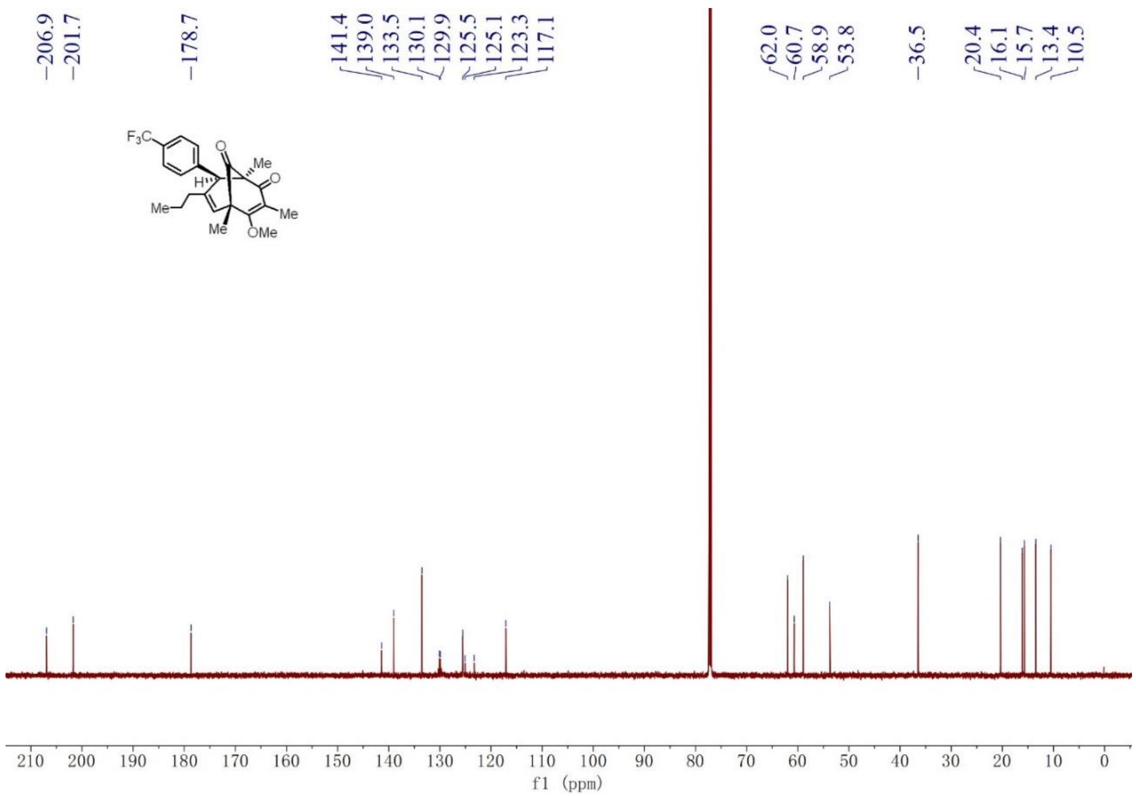


$^{13}$C NMR spectrum of compound **11p** (151MHz, $CDCl_3$)

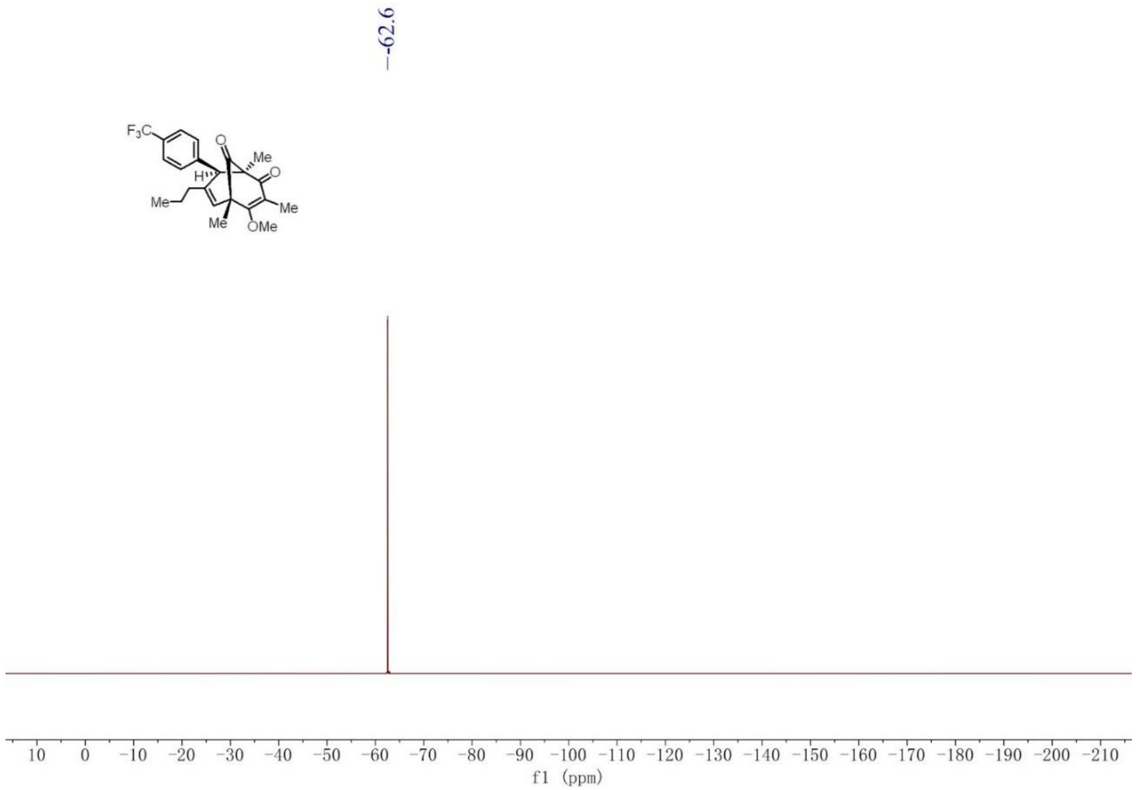


$^{19}$F NMR spectrum of compound **11p** (565MHz, $CDCl_3$)

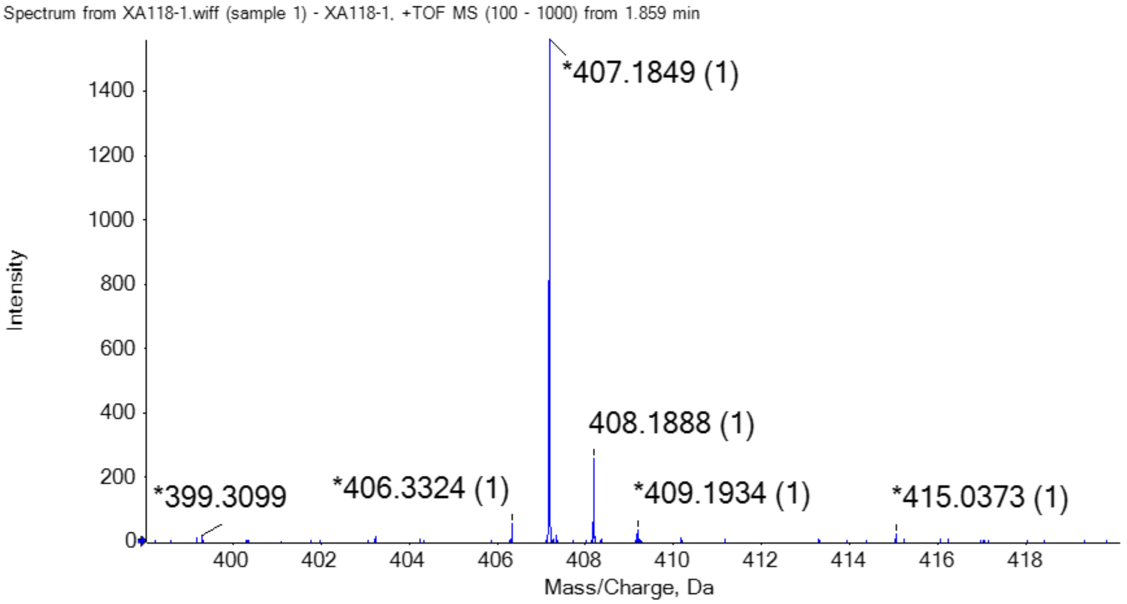


**Formula Calculator Results**

| Measured m/z | Cal m/z | Error(mmu) | Error(ppm) | Ion Formula | Ion |
|---|---|---|---|---|---|
| 407.1849 | 407.1828 | 2.0 | 5.0 | C23H26F3O3 | $[M+H]^+$ |

HRESIMS spectrum of compound **11p**

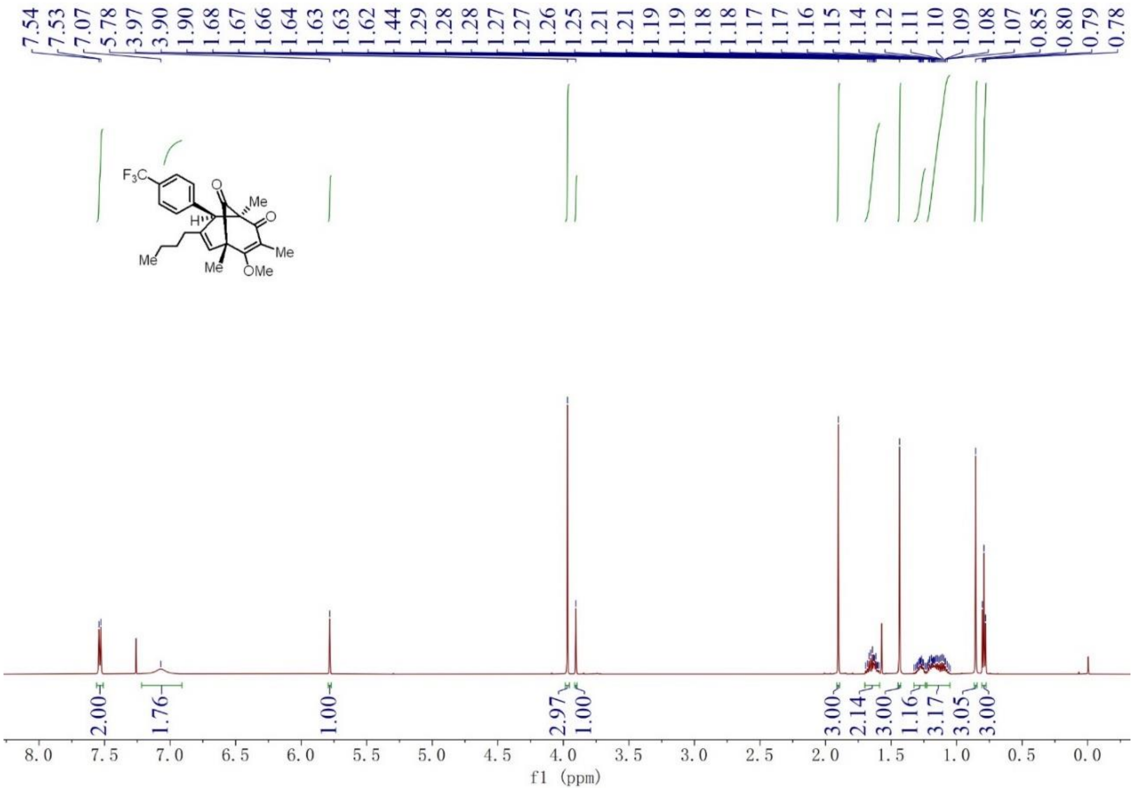


$^1$H NMR spectrum of compound **11q** (600MHz, $CDCl_3$)

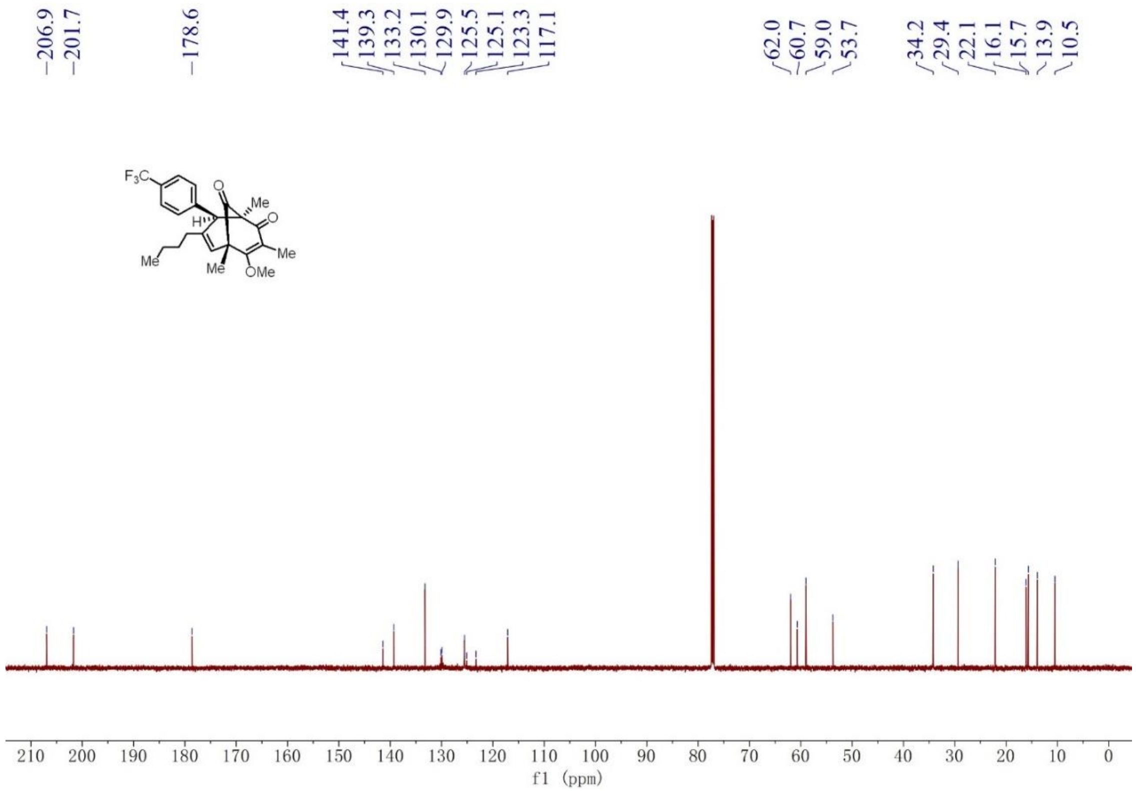


$^{13}$C NMR spectrum of compound **11q** (151MHz, $CDCl_3$)

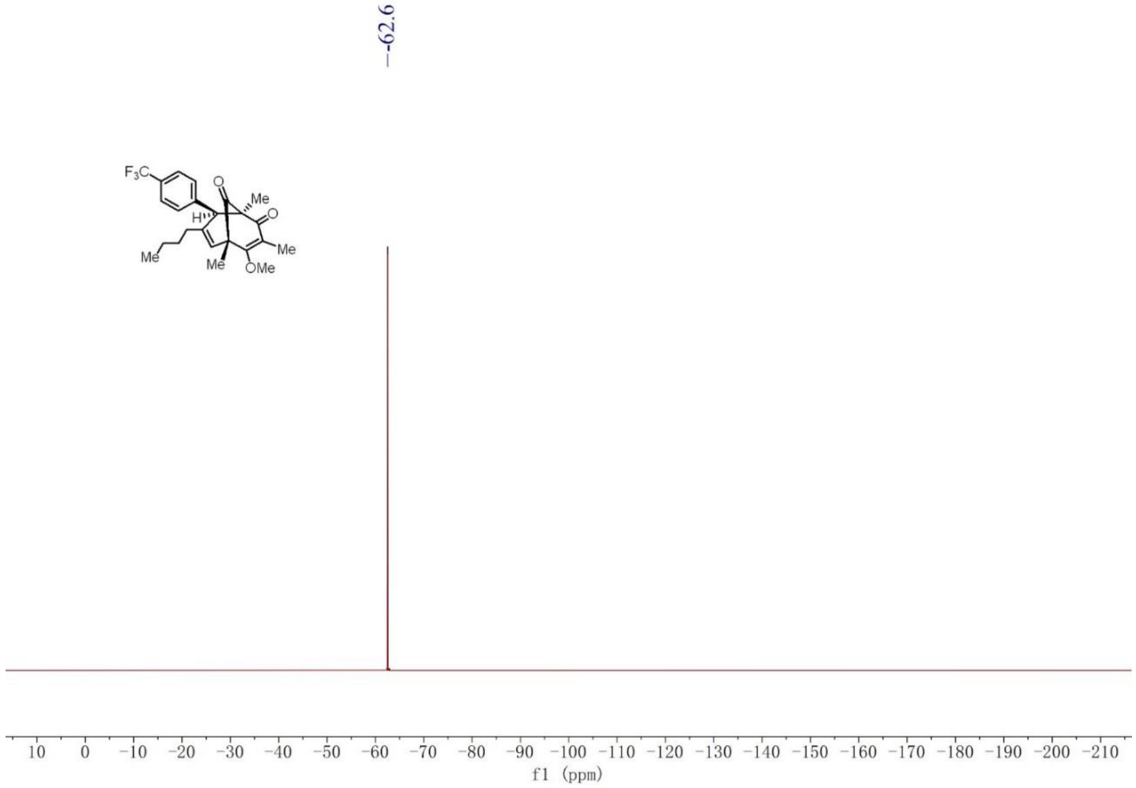


$^{19}$F NMR spectrum of compound **11q** (565MHz, $CDCl_3$)

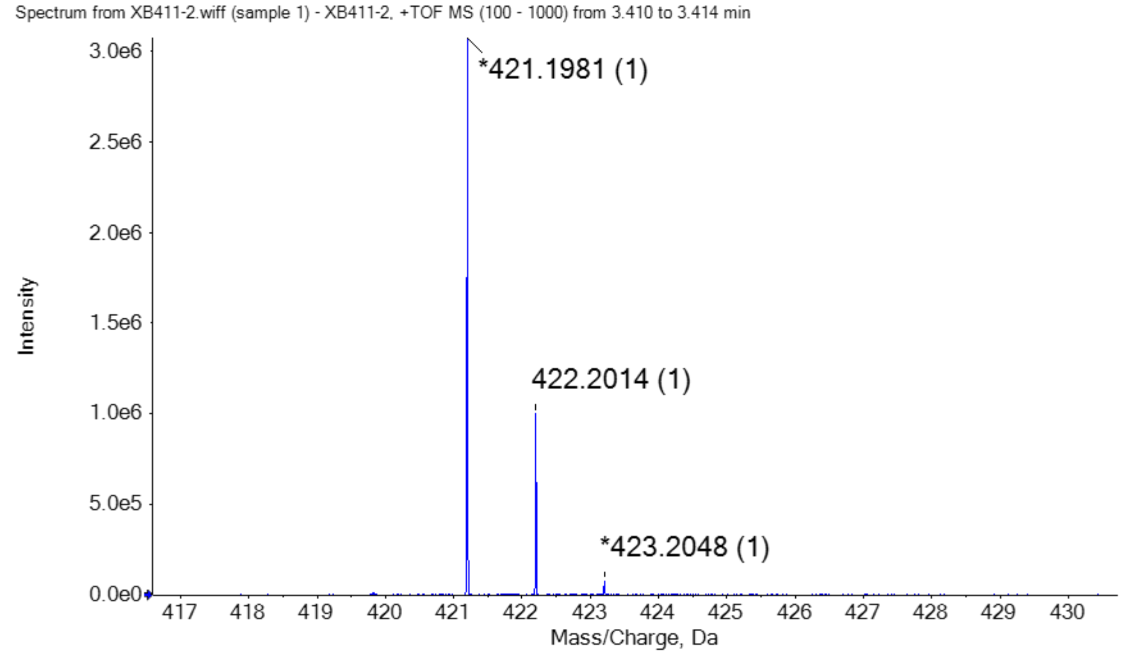


**Formula Calculator Results**

| Measured m/z | Cal m/z | Error(mmu) | Error(ppm) | Ion Formula | Ion |
|---|---|---|---|---|---|
| 421.1981 | 421.1985 | -0.4 | -1.0 | C24H28F3O3 | $[M+H]^+$ |

HRESIMS spectrum of compound **11q**

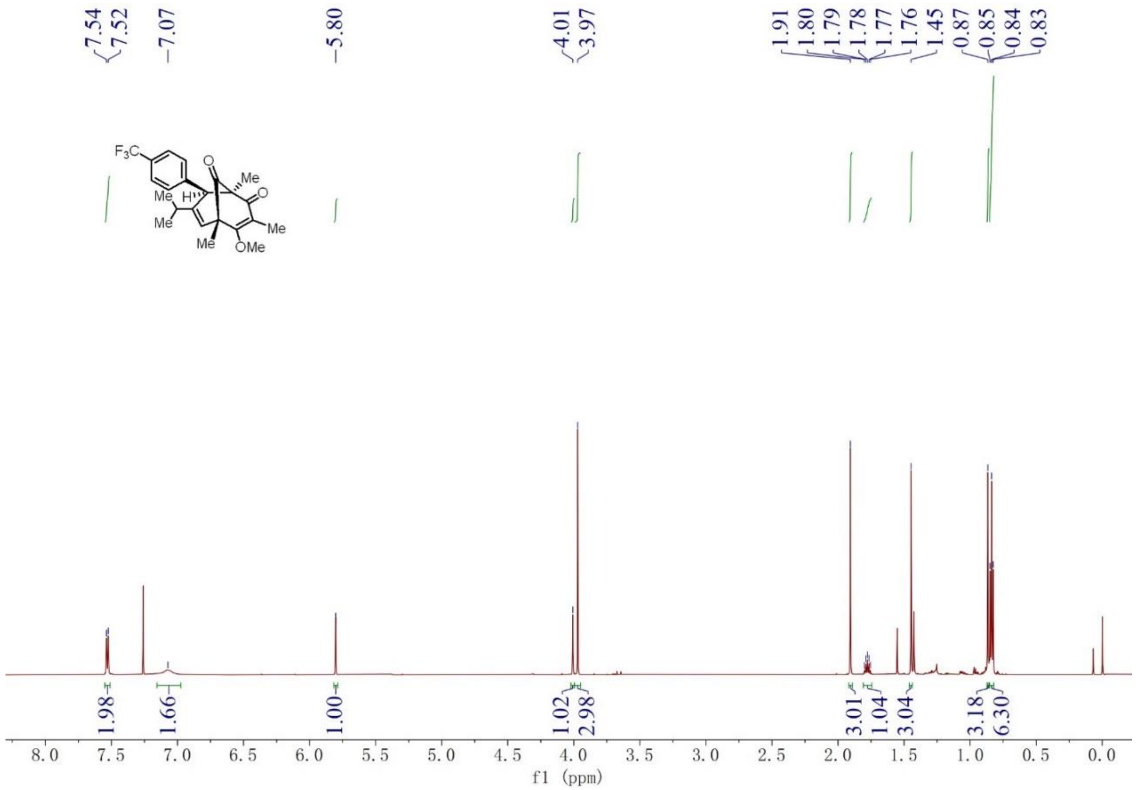


$^1$H NMR spectrum of compound **11r** (600MHz, $CDCl_3$)

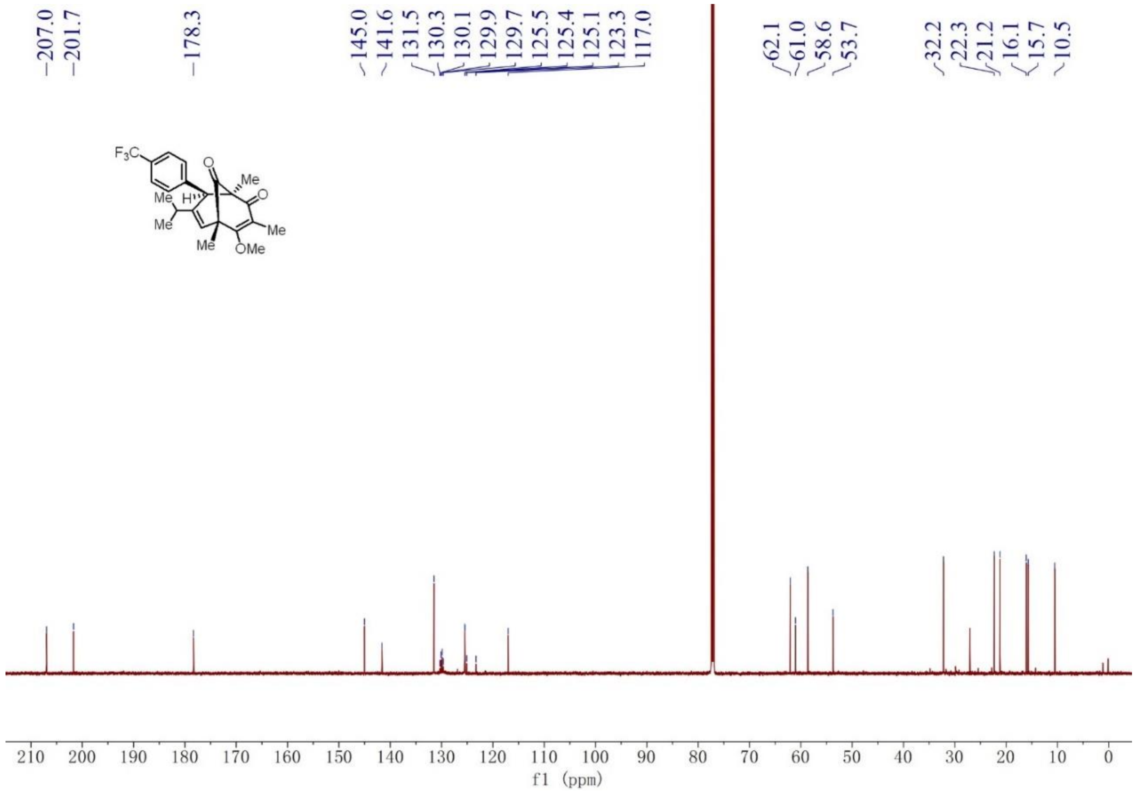


$^{13}$C NMR spectrum of compound **11r** (151MHz, $CDCl_3$)

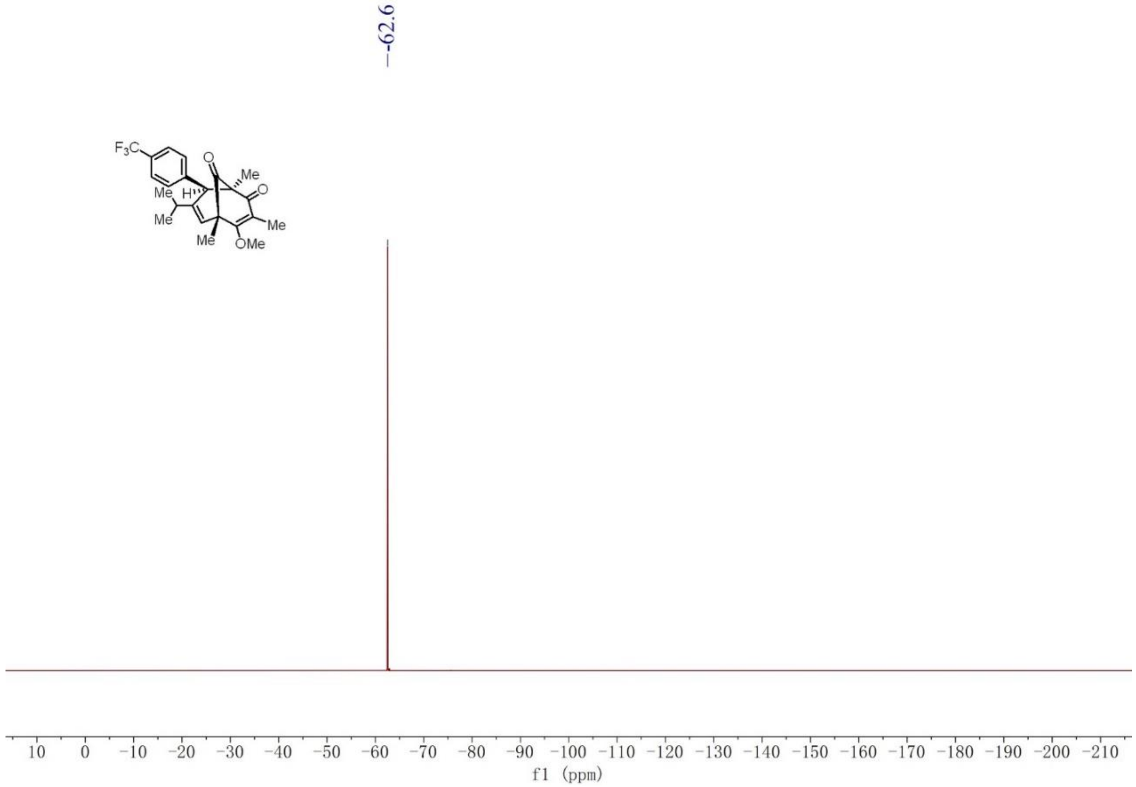


$^{19}$F NMR spectrum of compound **11r** (565MHz, $CDCl_3$)

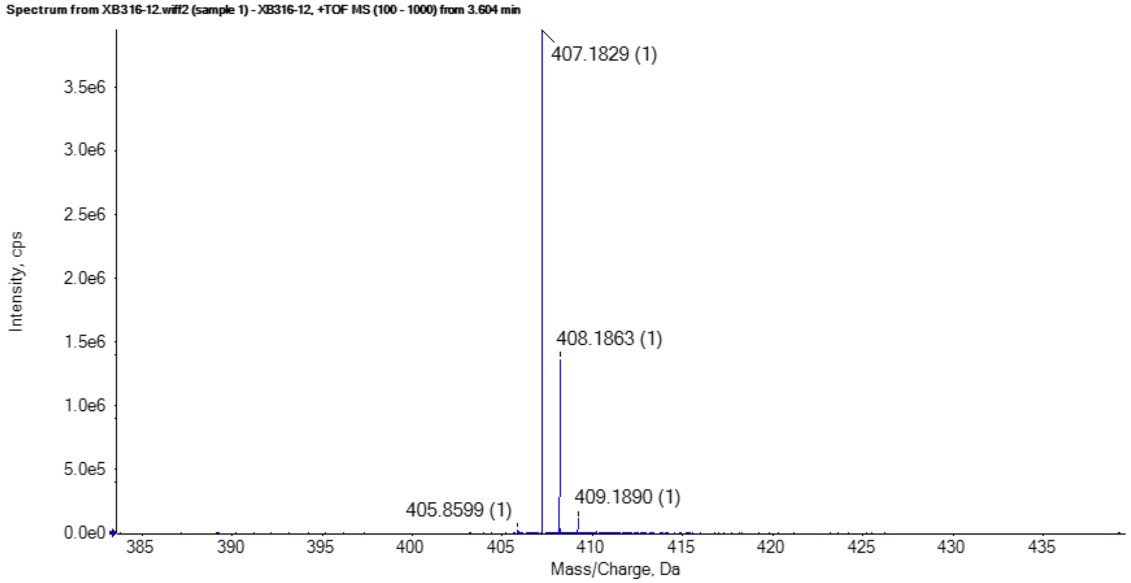


## Formula Calculator Results

| Measured m/z | Cal m/z | Error(mmu) | Error(ppm) | Ion Formula | Ion |
|---|---|---|---|---|---|
| 407.1829 | 407.1829 | 0 | 0 | C23H26F3O3 | $[M+H]^+$ |

HRESIMS spectrum of compound **11r**

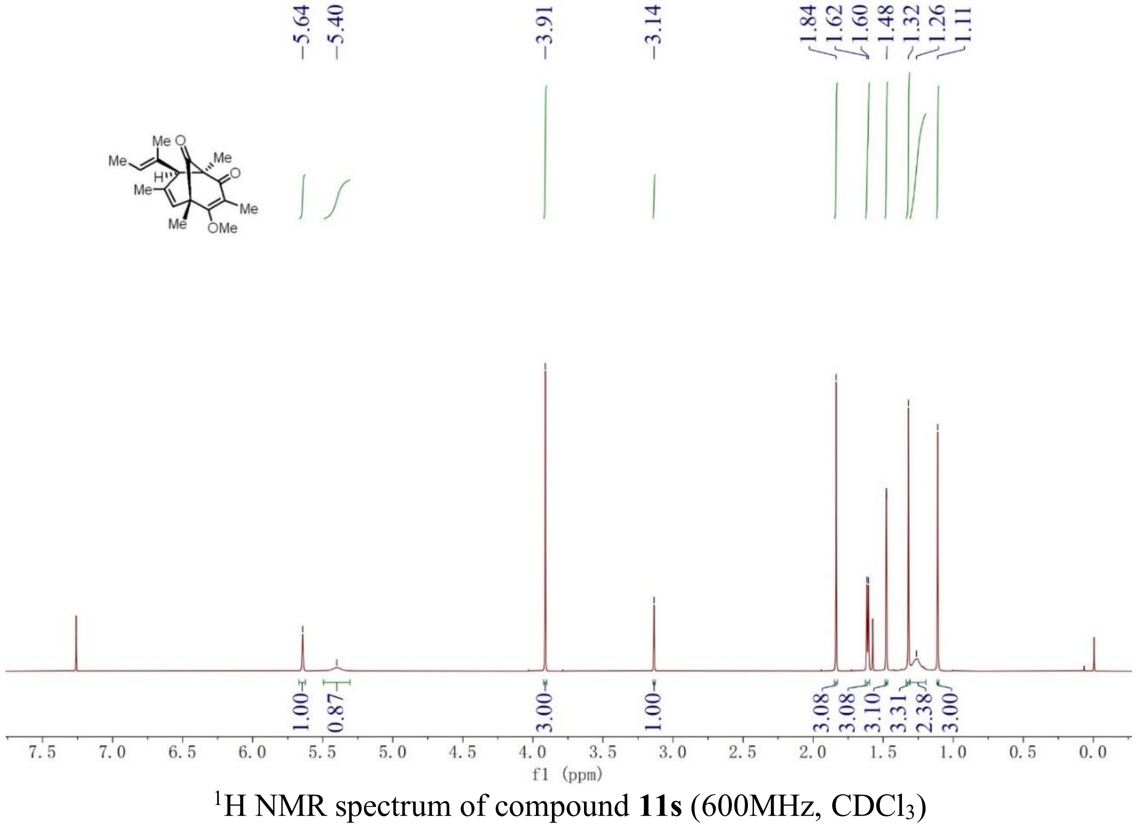


$^1$H NMR spectrum of compound **11s** (600MHz, $CDCl_3$)

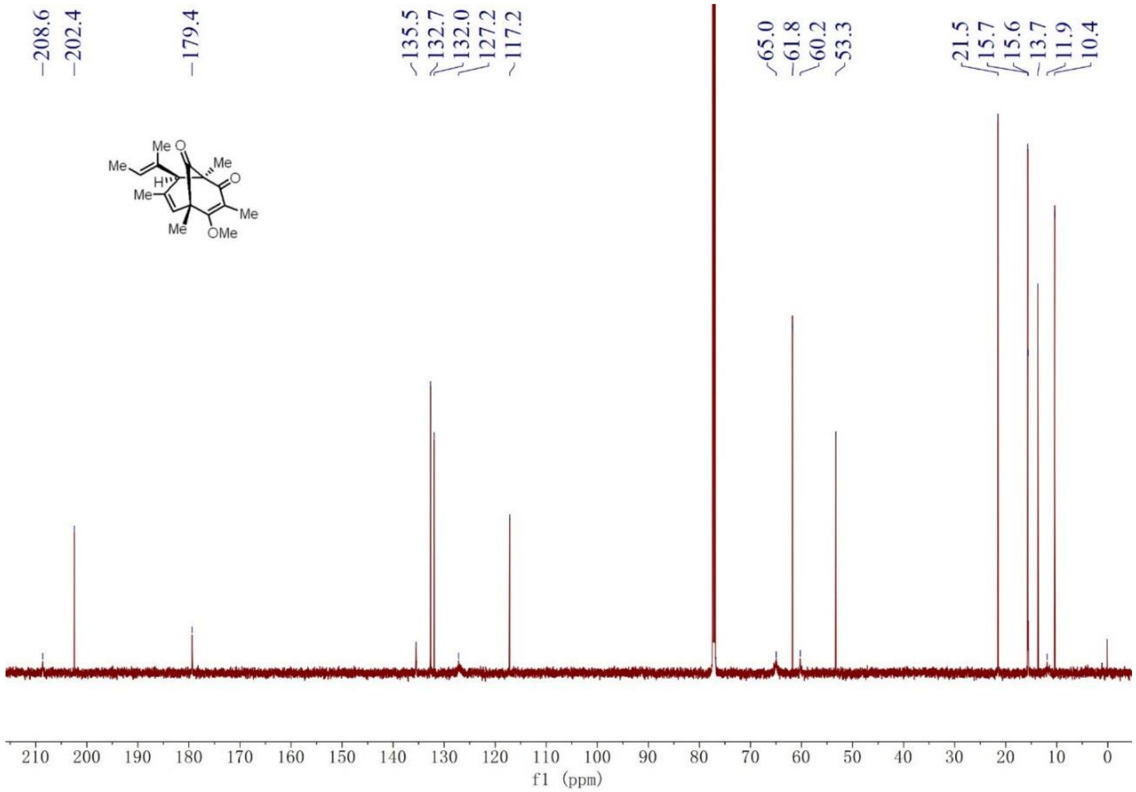


$^{13}$C NMR spectrum of compound **11s** (151MHz, $CDCl_3$)

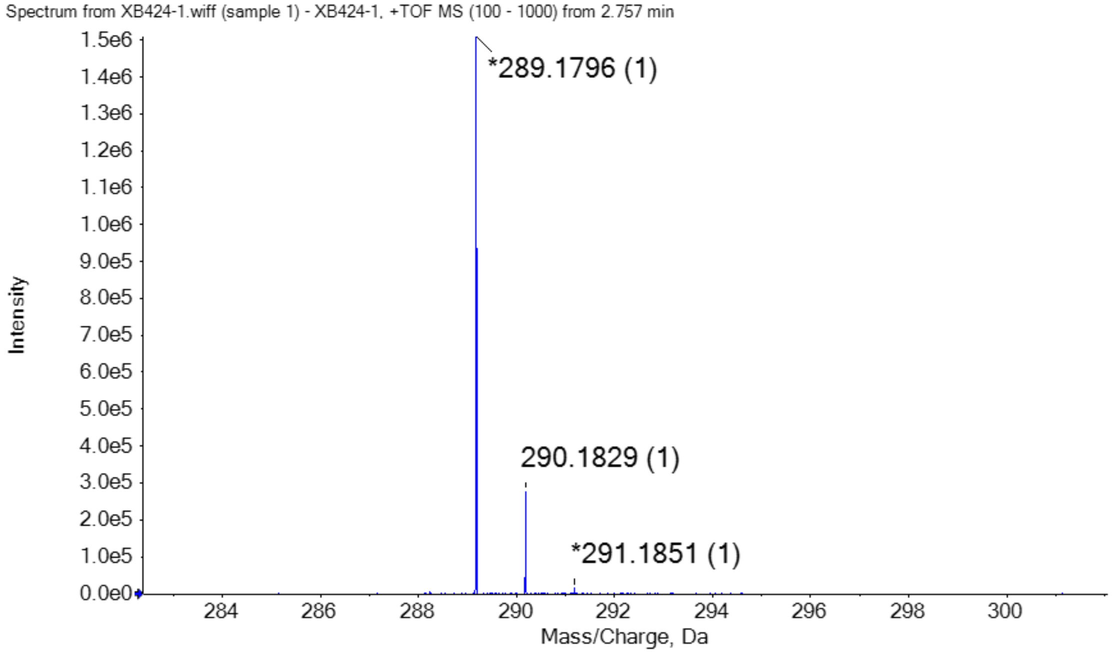


**Formula Calculator Results**

| Measured m/z | Cal m/z | Error(mmu) | Error(ppm) | Ion Formula | Ion |
|---|---|---|---|---|---|
| 289.1796 | 289.1798 | -0.2 | -0.8 | C18H25O3 | $[M+H]^+$ |

HRESIMS spectrum of compound **11s**

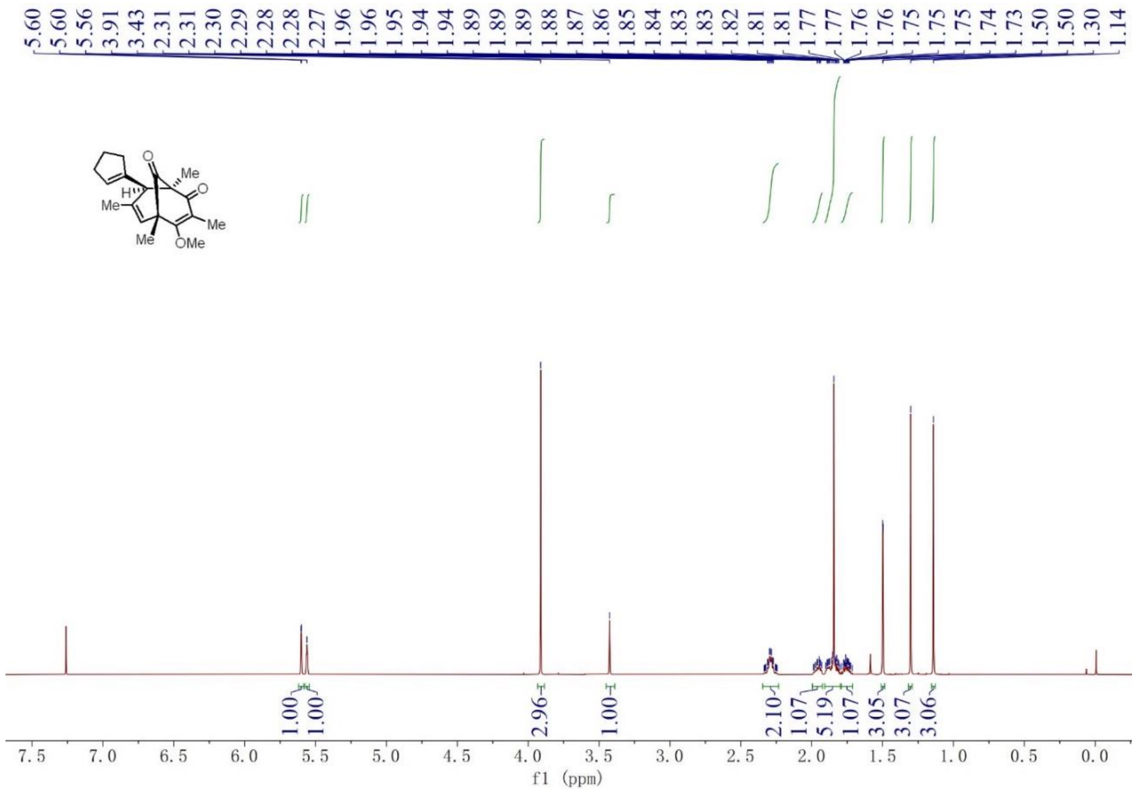

$^{1}H$ NMR spectrum of compound **11t** (600MHz, $CDCl_3$)

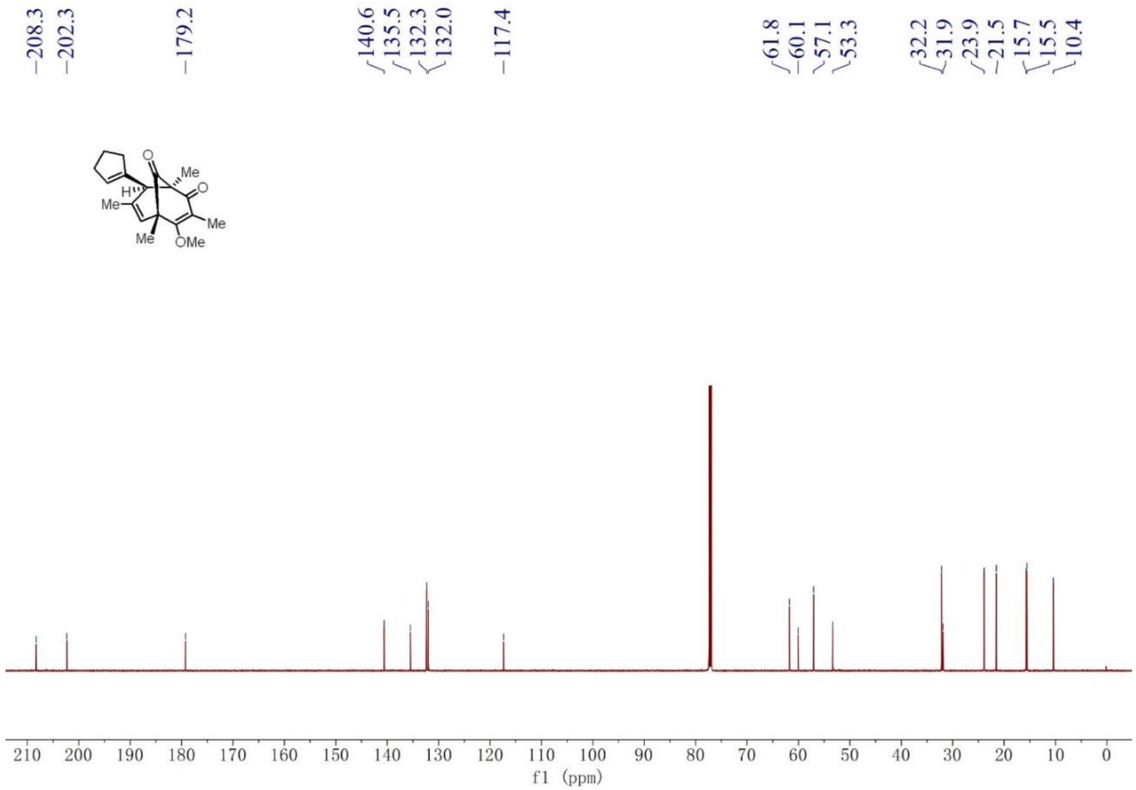

$^{13}C$ NMR spectrum of compound **11t** (151MHz, $CDCl_3$)

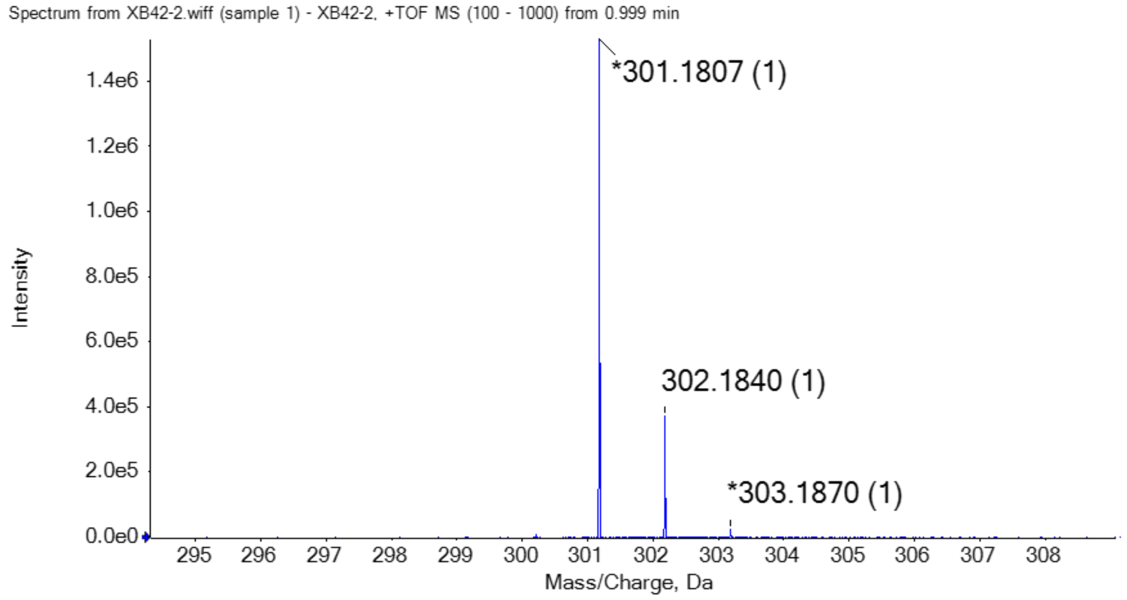


**Formula Calculator Results**

| Measure m/z | Cal m/z | Error(mmu) | Error(ppm) | Ion Formula | Ion |
|---|---|---|---|---|---|
| 301.1807 | 301.1798 | 0.9 | 2.9 | C19H25O3 | $[M+H]^+$ |

HRESIMS spectrum of compound **11t**

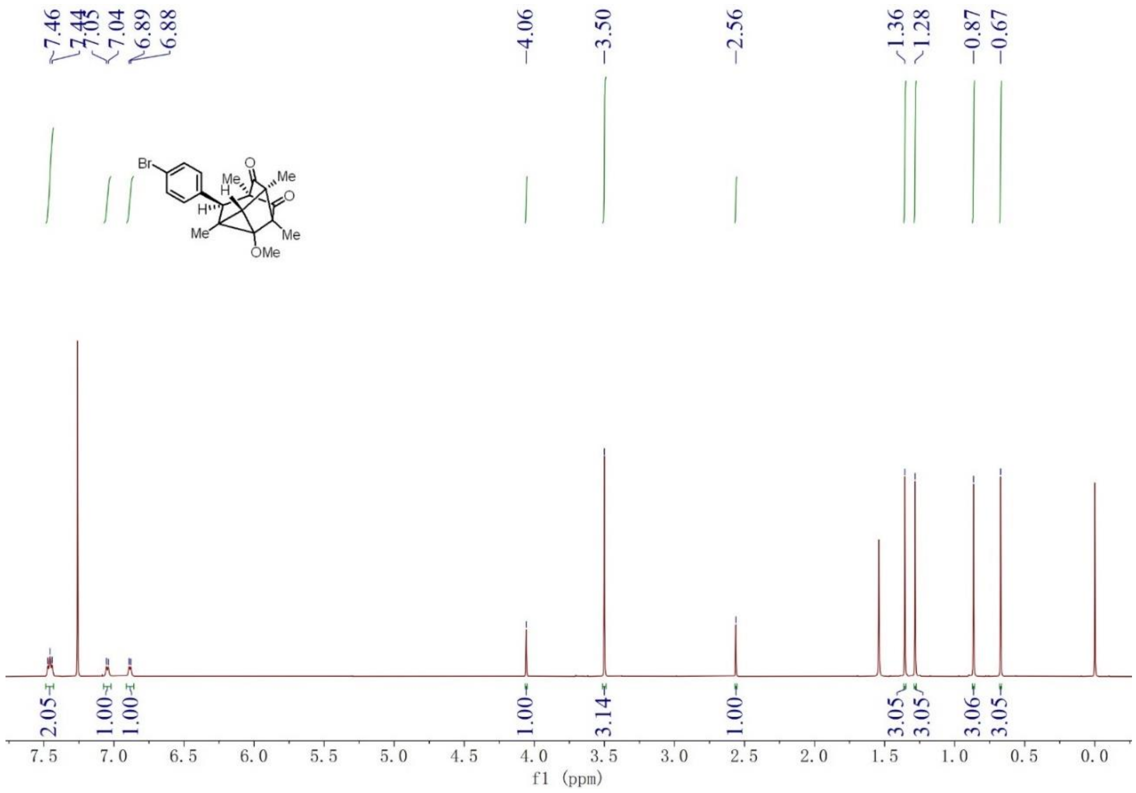


$^1$H NMR spectrum of compound **12** (600MHz, $CDCl_3$)

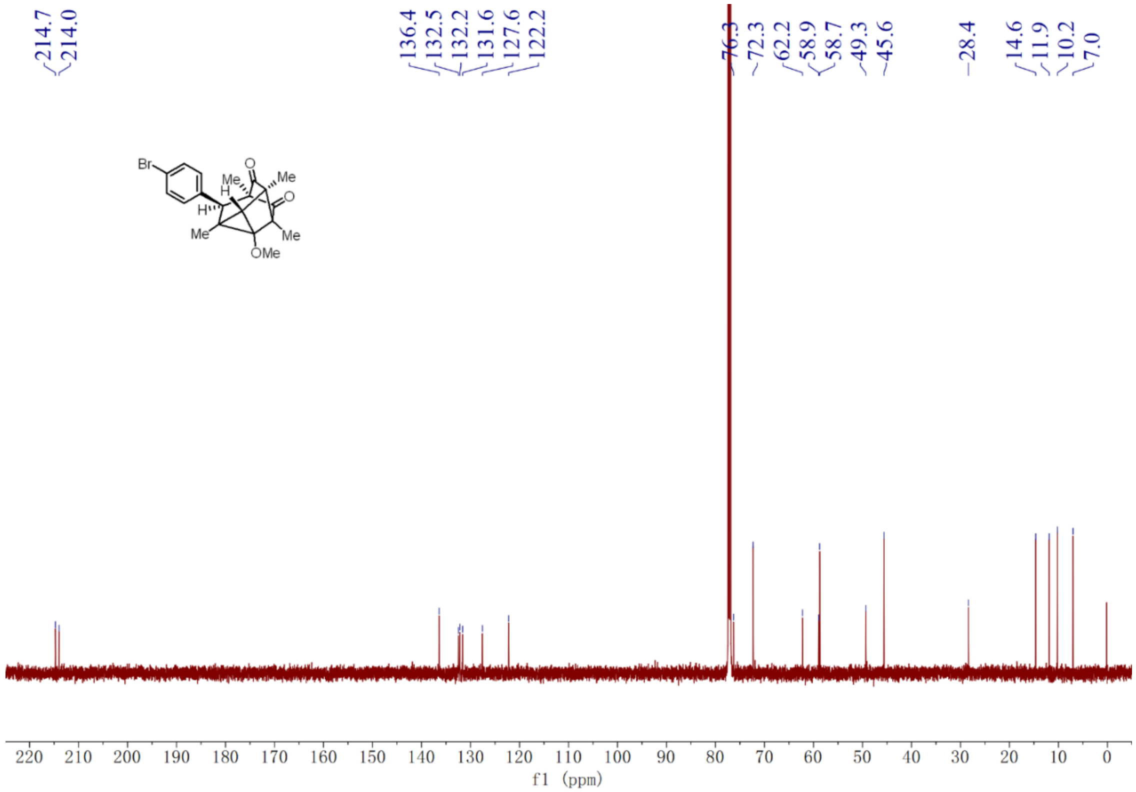


$^{13}$C NMR spectrum of compound **12** (151MHz, $CDCl_3$)

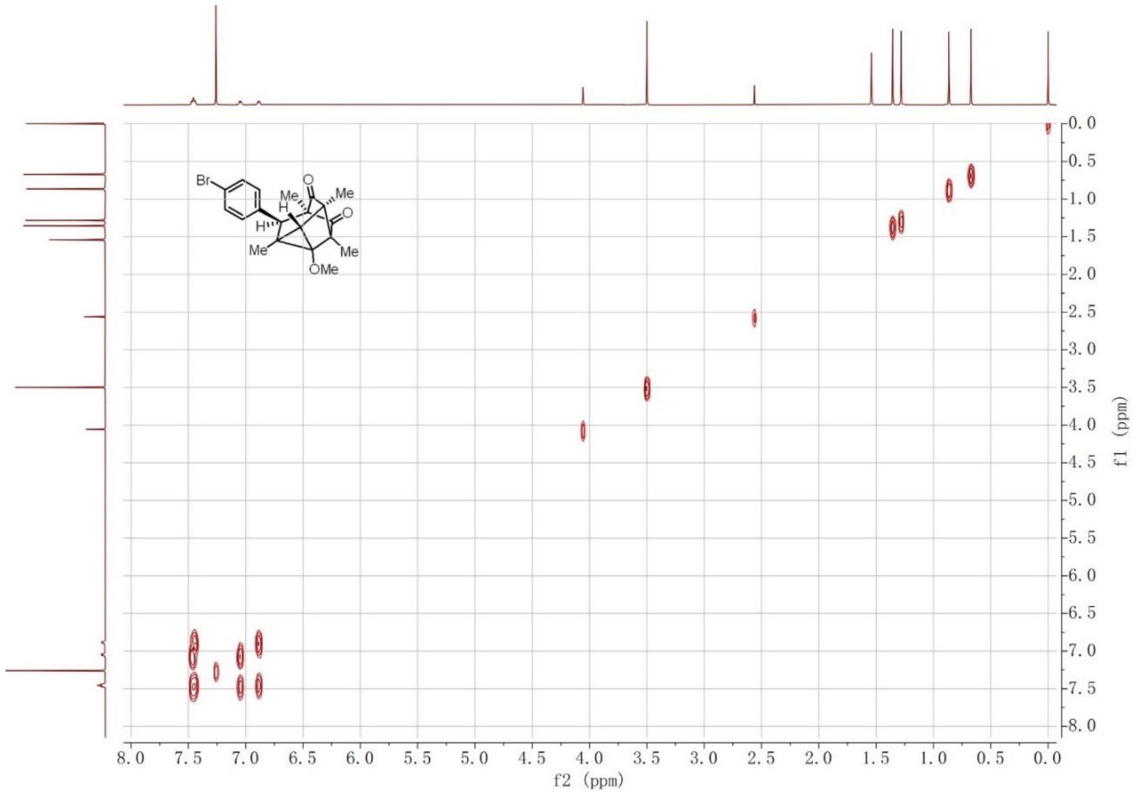


$^{1}$H-$^{1}$H COSY spectrum of compound **12** (600 MHz, $CDCl_3$)

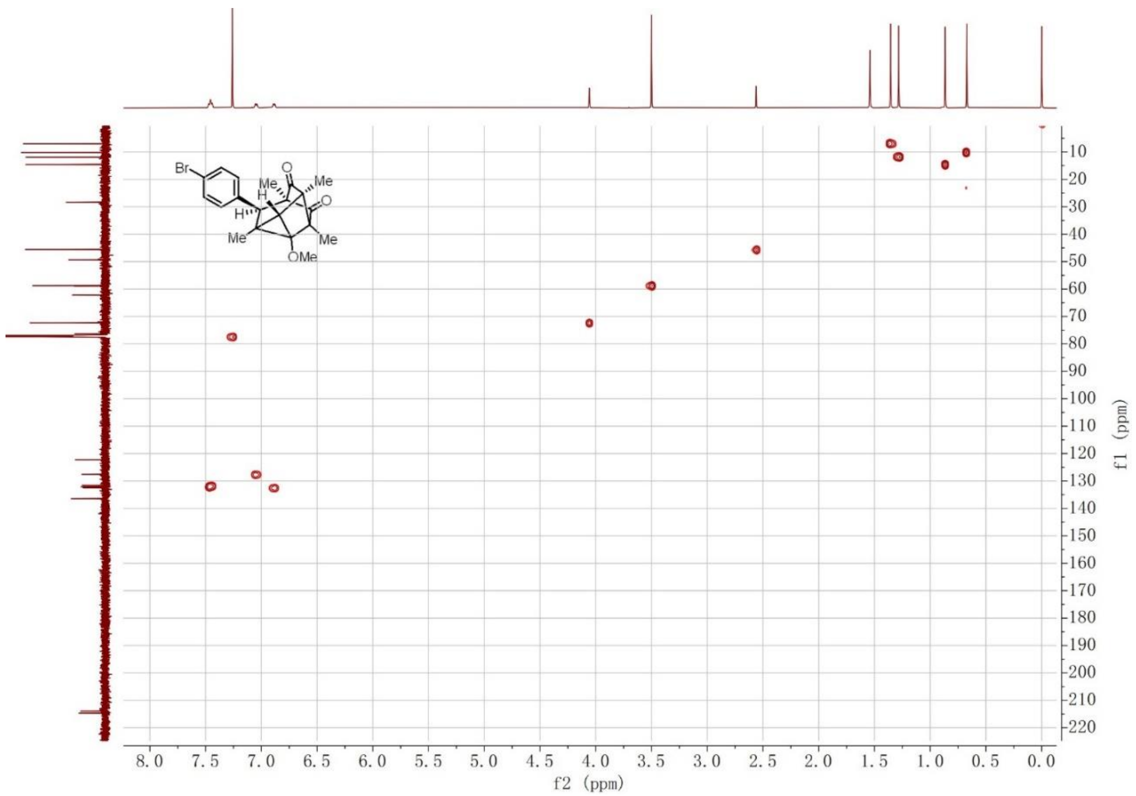

HSQC spectrum of compound **12** (600 MHz, $CDCl_3$)

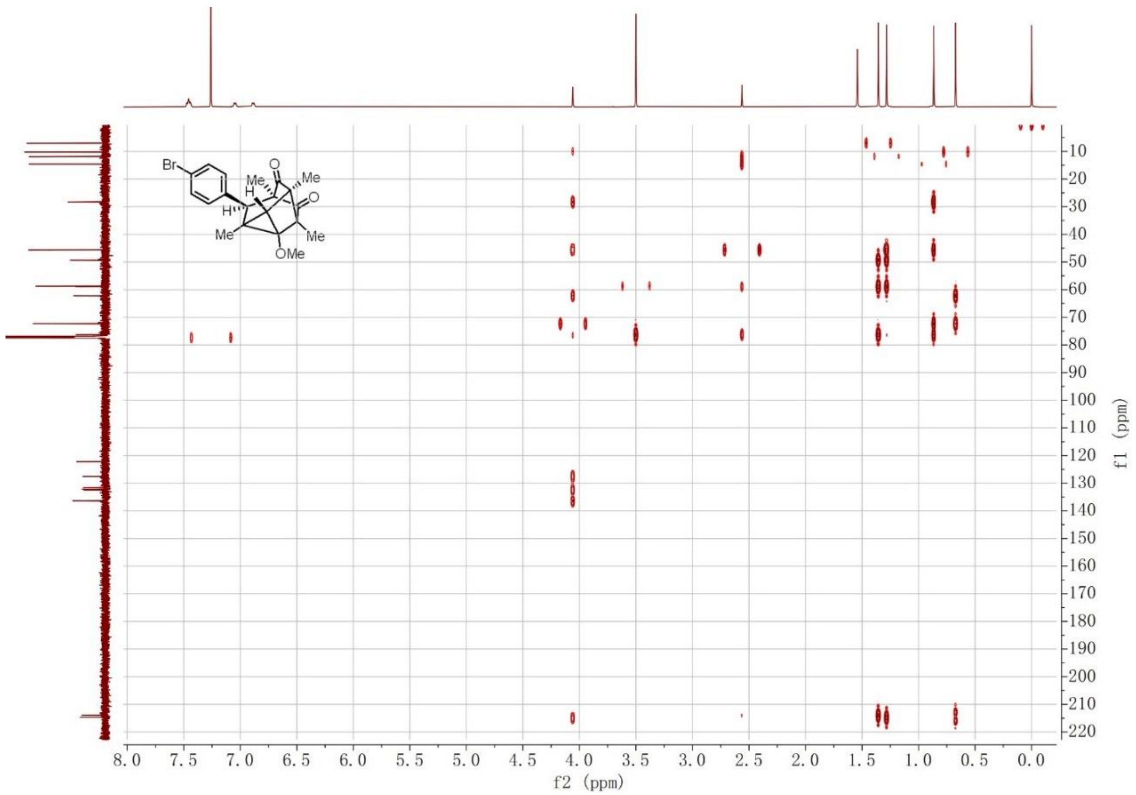

HMBC spectrum of compound **12** (600 MHz, $CDCl_3$)

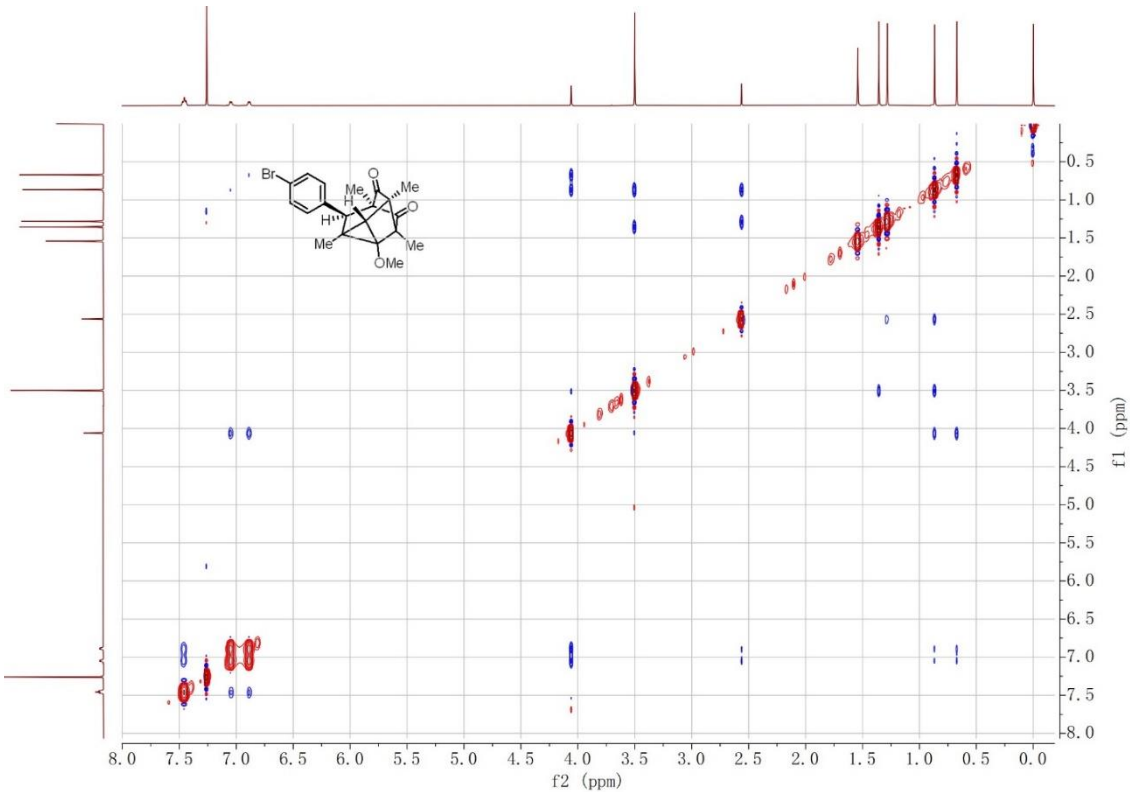


NOESY spectrum of compound **12** (600 MHz, $CDCl_3$)

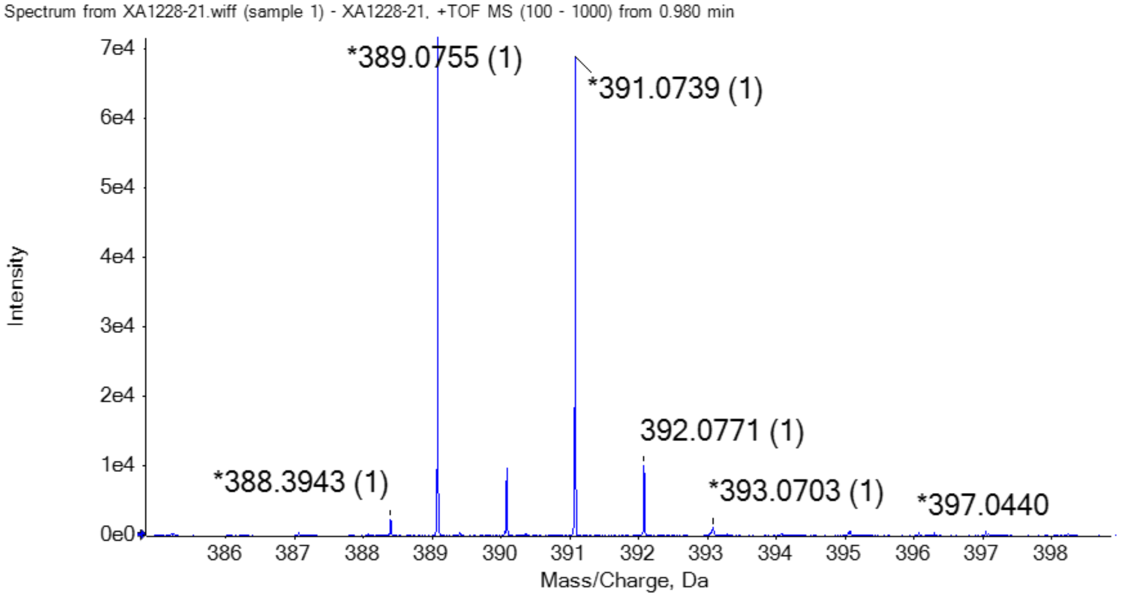


**Formula Calculator Results**

| Measure m/z | Cal m/z | Error(mmu) | Error(ppm) | Ion Formula | Ion |
|---|---|---|---|---|---|
| 389.0755 | 389.0746 | 0.8 | 2.1 | C20H22BrO3 | $[M+H]^+$ |

HRESIMS spectrum of compound **12**

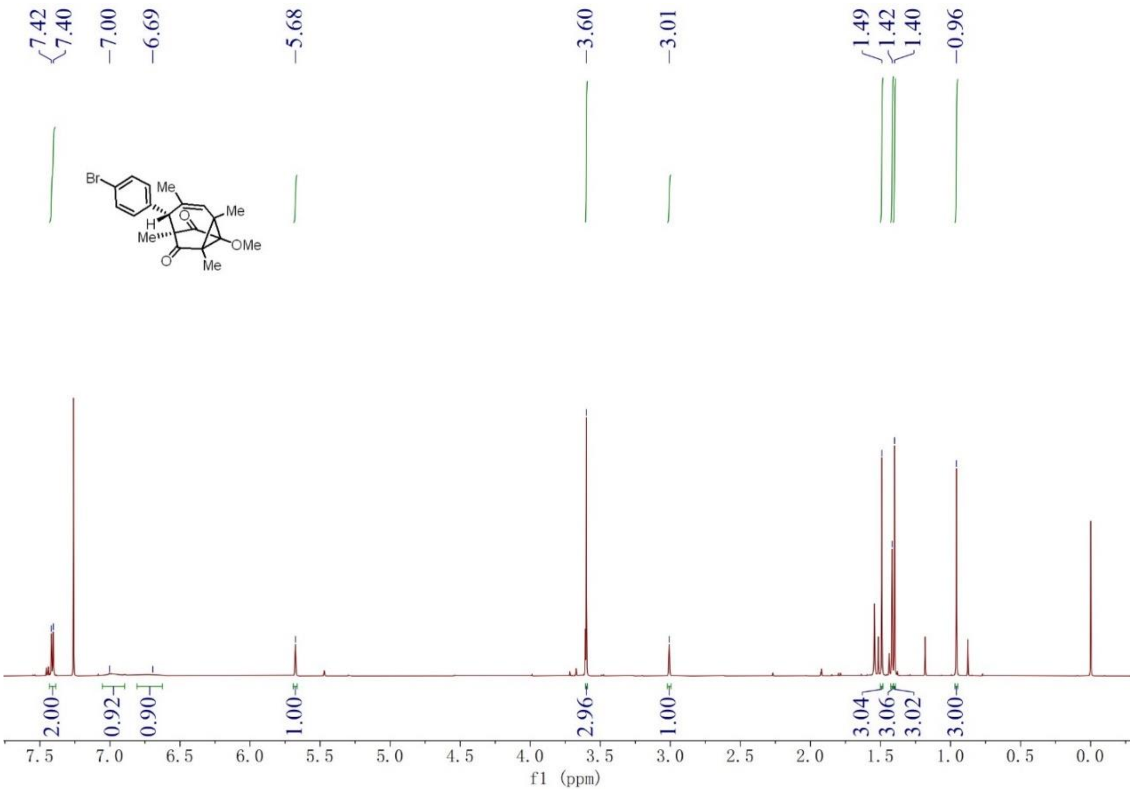


$^{1}$H NMR spectrum of compound **13** (600MHz, $CDCl_3$)

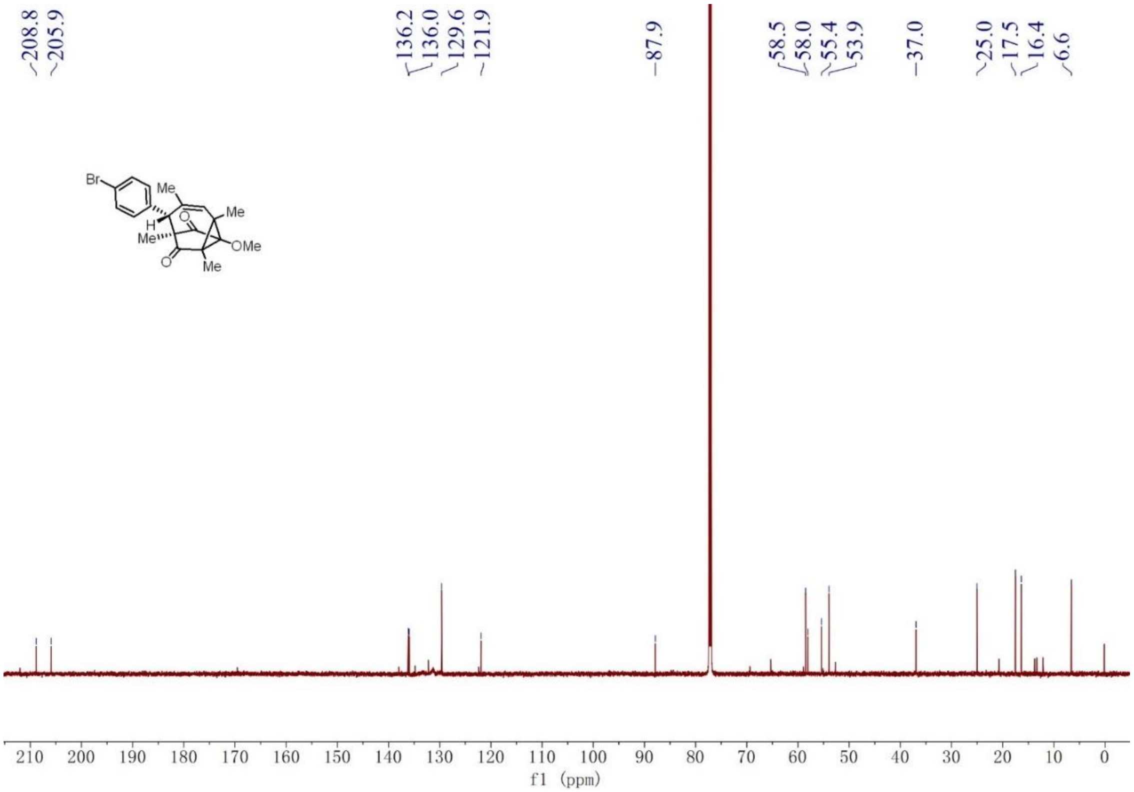


$^{13}$C NMR spectrum of compound **13** (151MHz, $CDCl_3$)

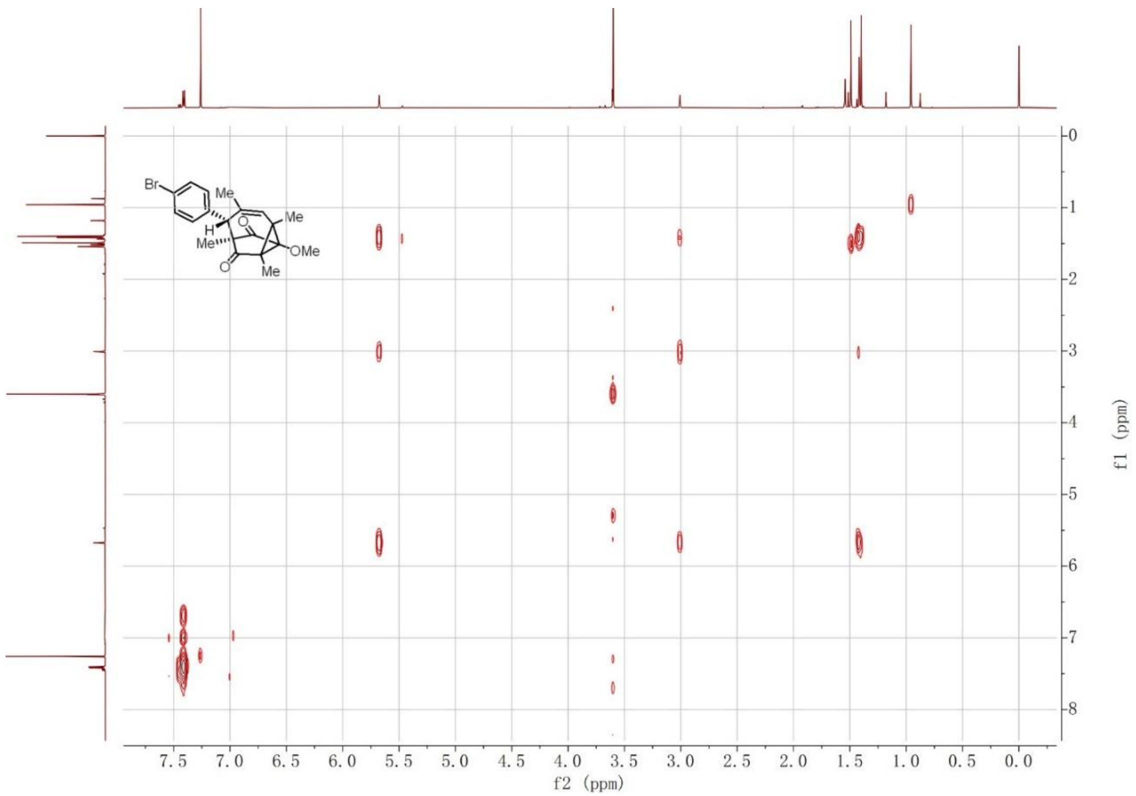

$^{1}H$-$^{1}H$ COSY spectrum of compound **13** (600 MHz, $CDCl_3$)

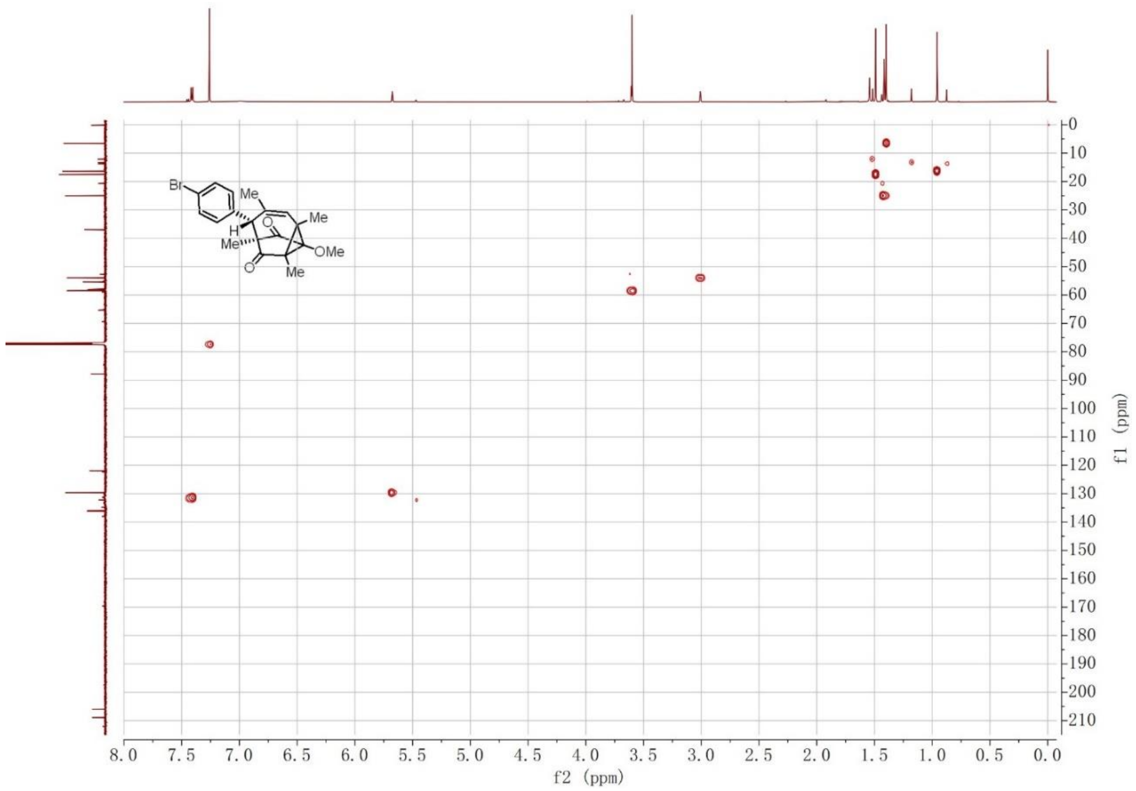

HSQC spectrum of compound **13** (600 MHz, $CDCl_3$)

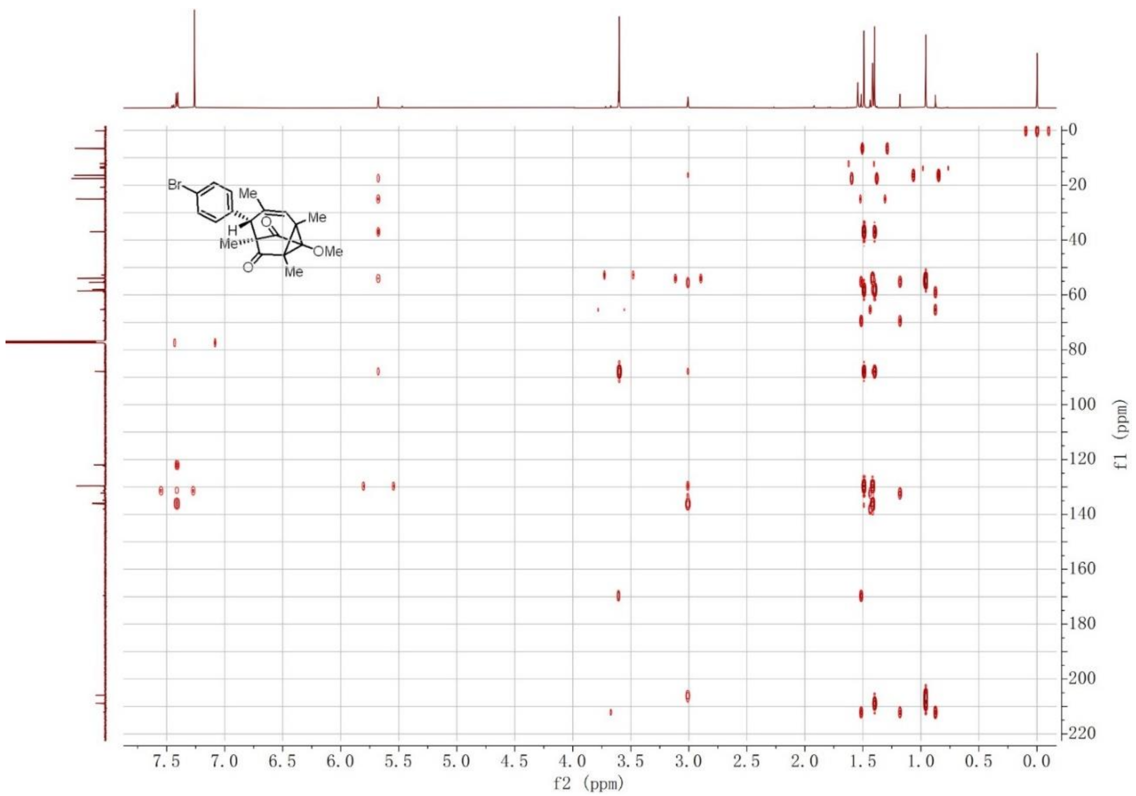

HMBC spectrum of compound **13** (600 MHz, $CDCl_3$)

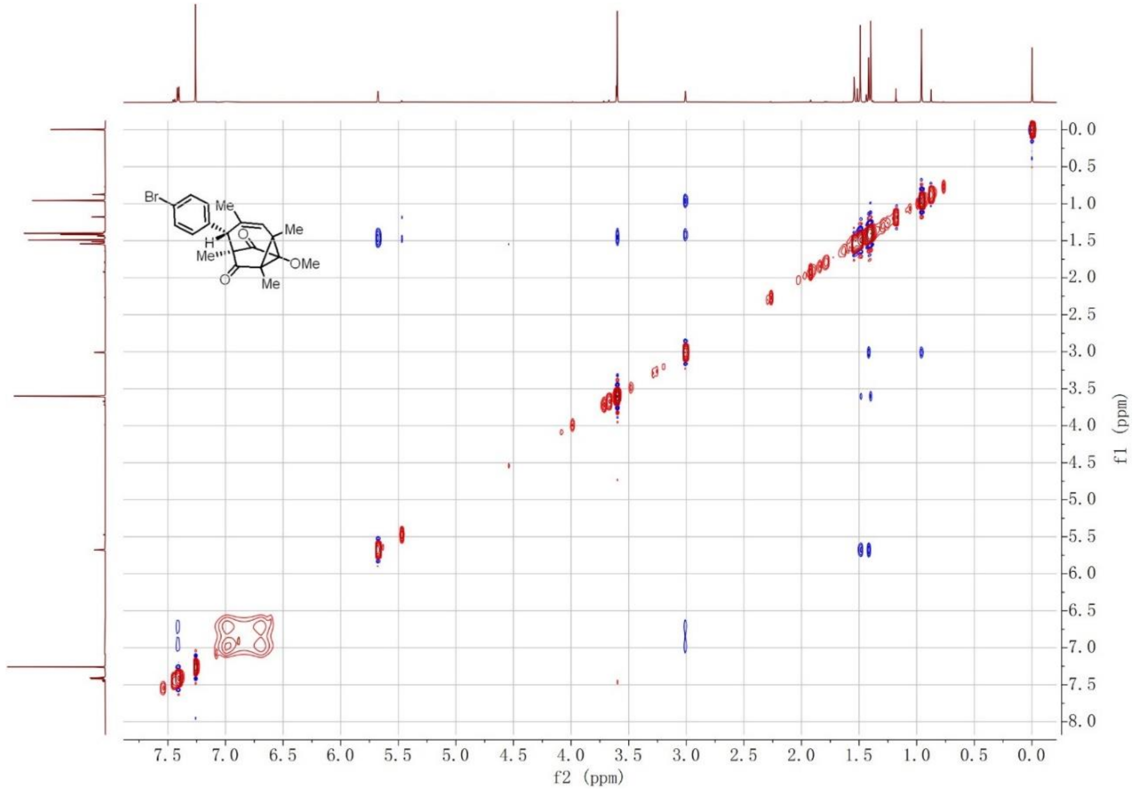

NOESY spectrum of compound **13** (600 MHz, $CDCl_3$)

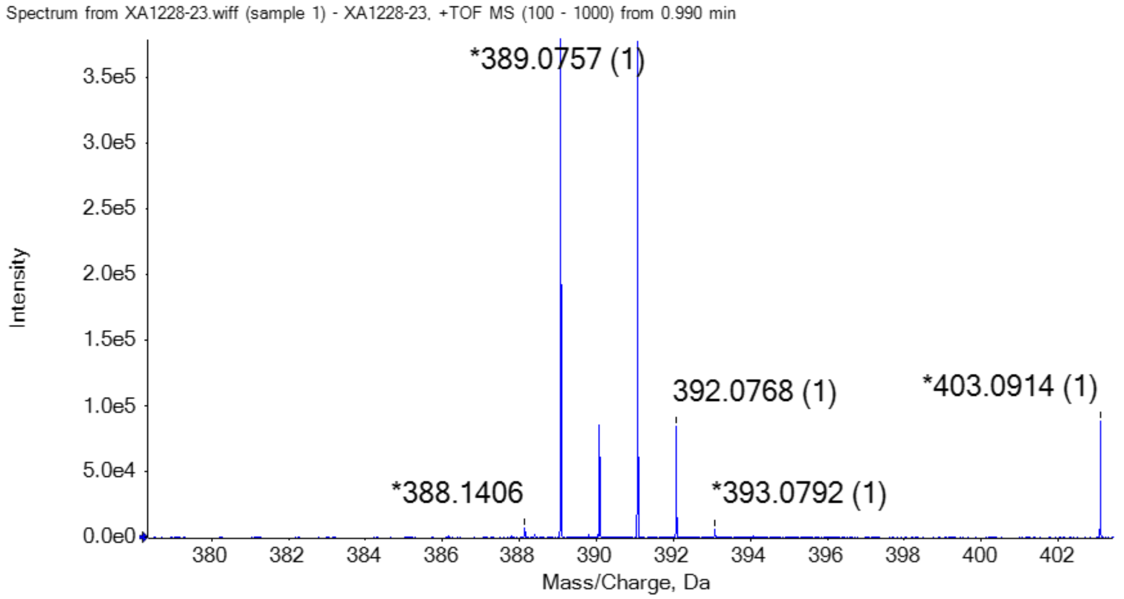


**Formula Calculator Results**

| Measure m/z | Cal m/z | Error(mmu) | Error(ppm) | Ion Formula | Ion |
|---|---|---|---|---|---|
| 389.0757 | 389.0746 | 1.0 | 2.6 | C20H22BrO3 | $[M+H]^+$ |

HRESIMS spectrum of compound **13**

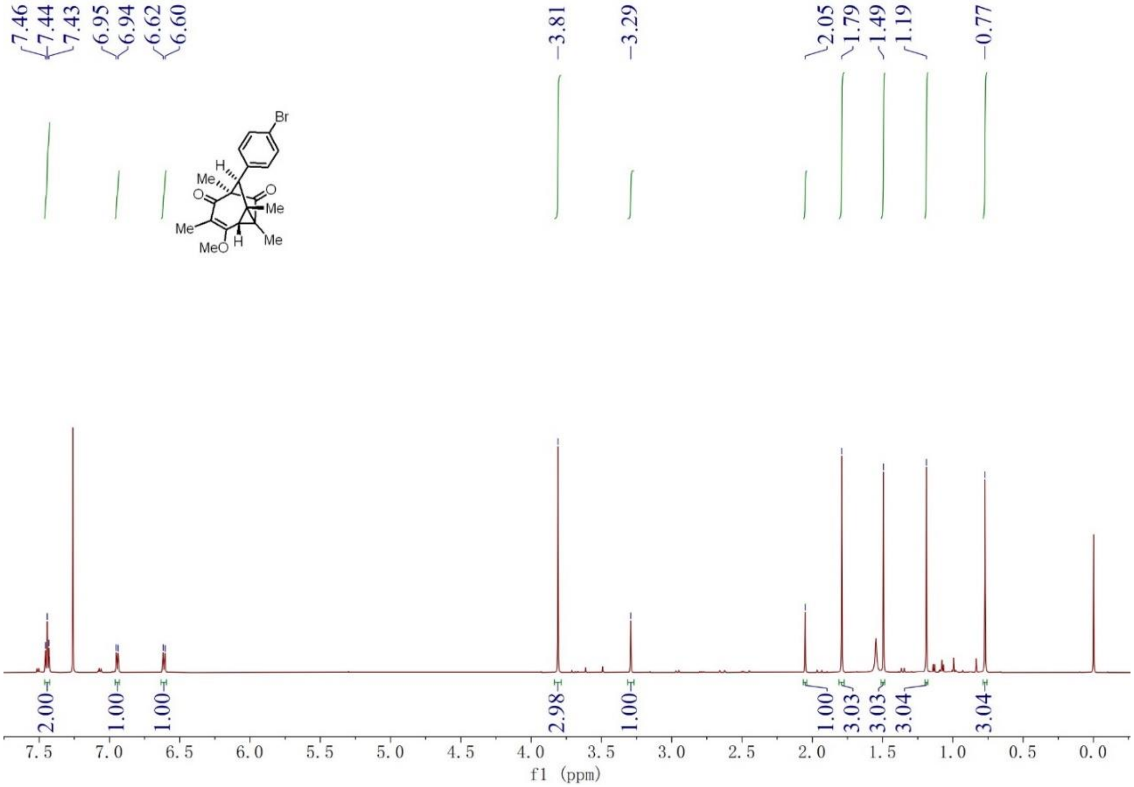


$^1$H NMR spectrum of compound **14** (600MHz, $CDCl_3$)

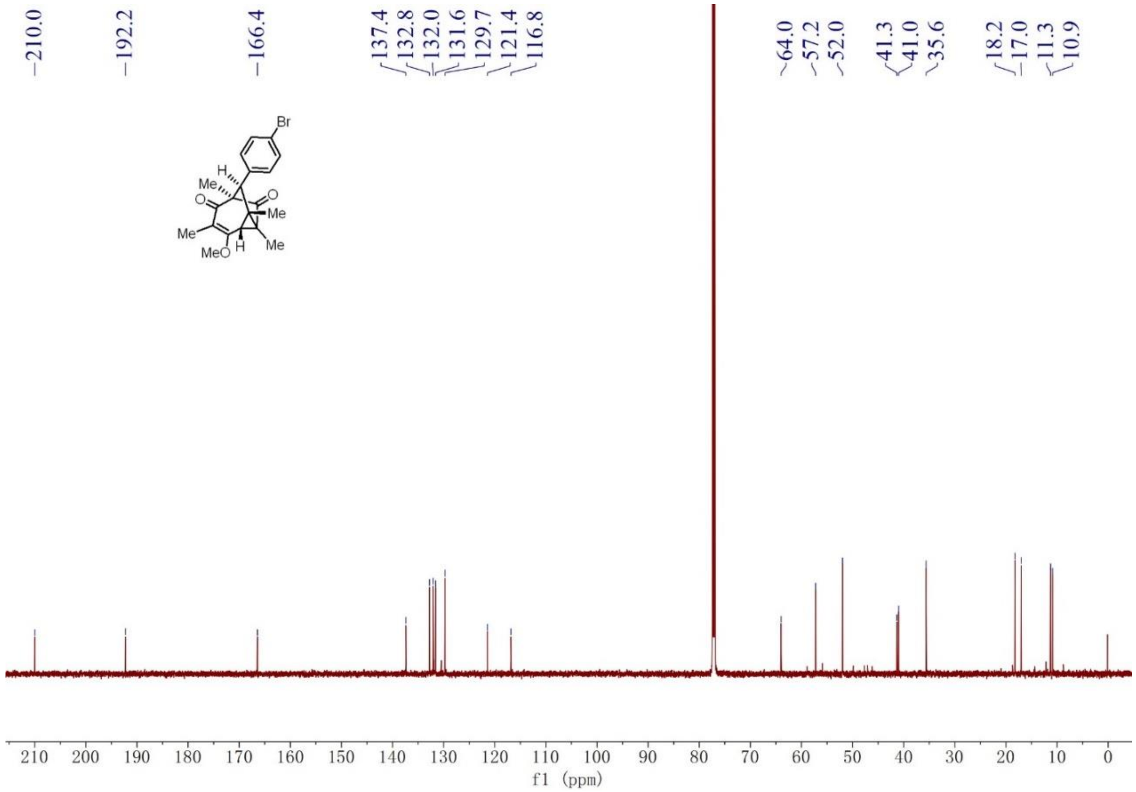


$^{13}$C NMR spectrum of compound **14** (151MHz, $CDCl_3$)

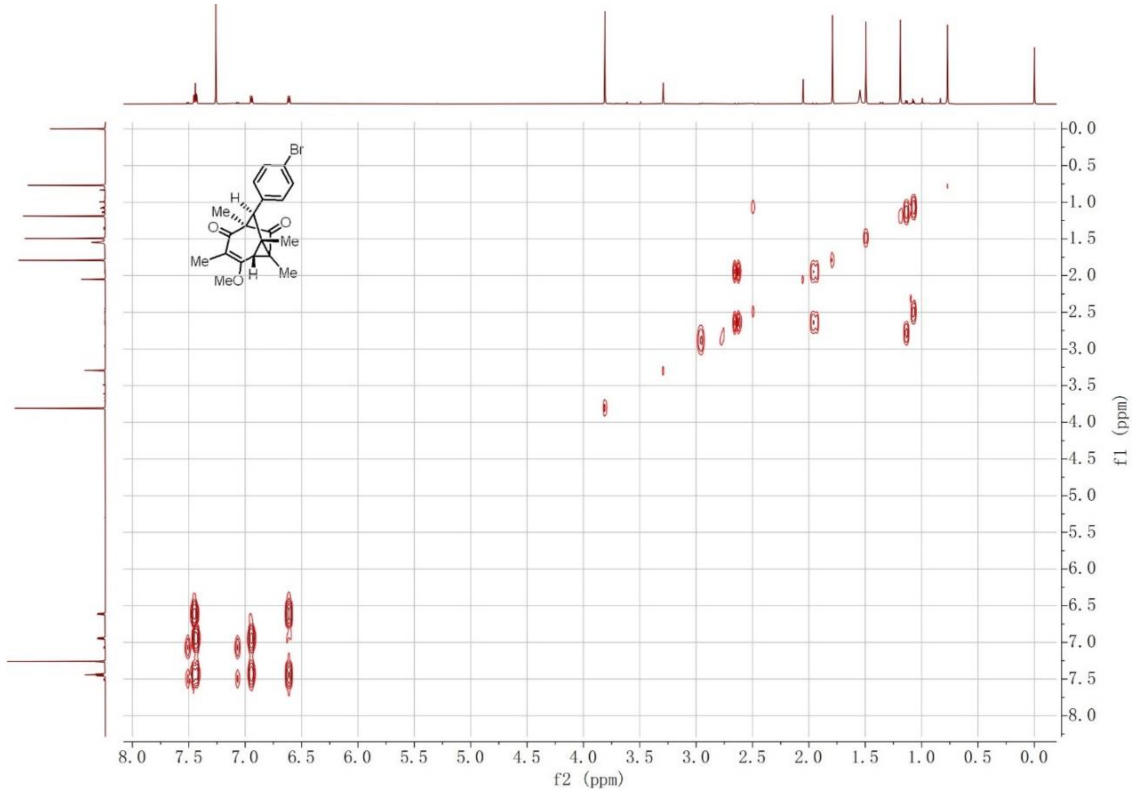


$^{1}$H-$^{1}$H COSY spectrum of compound **14** (600 MHz, $CDCl_3$)

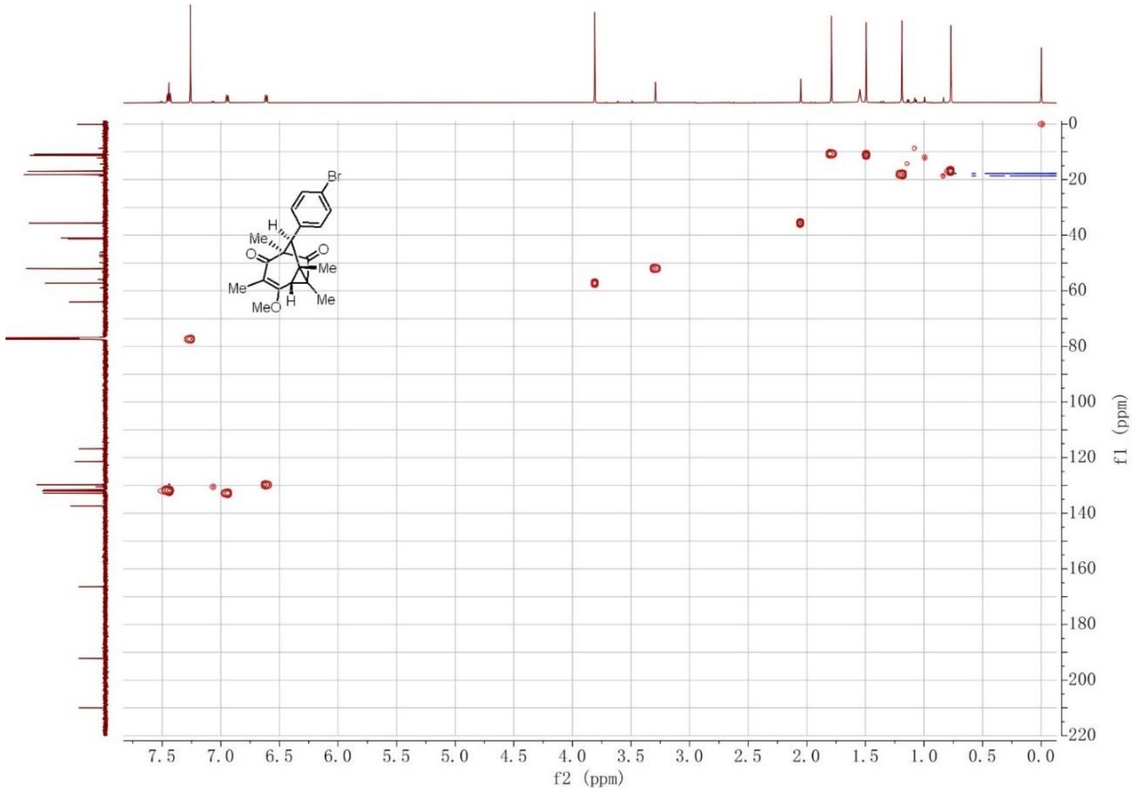

HSQC spectrum of compound **14** (600 MHz, $CDCl_3$)

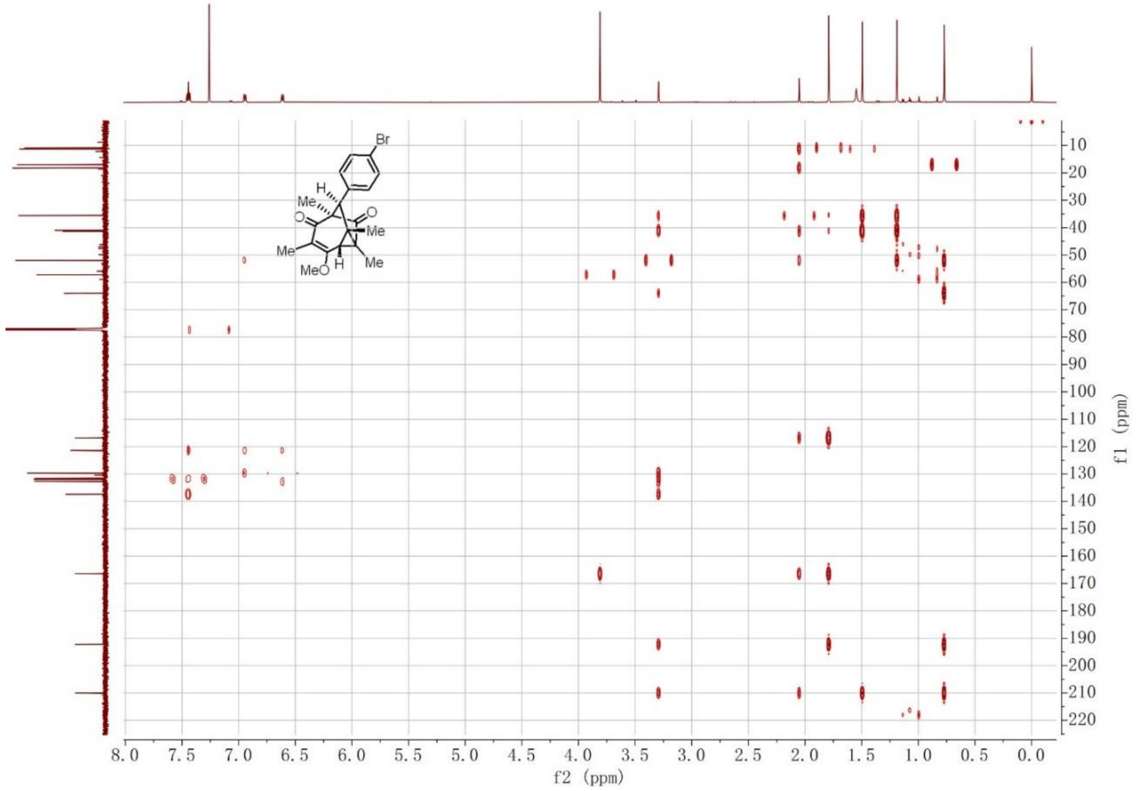

HMBC spectrum of compound **14** (600 MHz, $CDCl_3$)

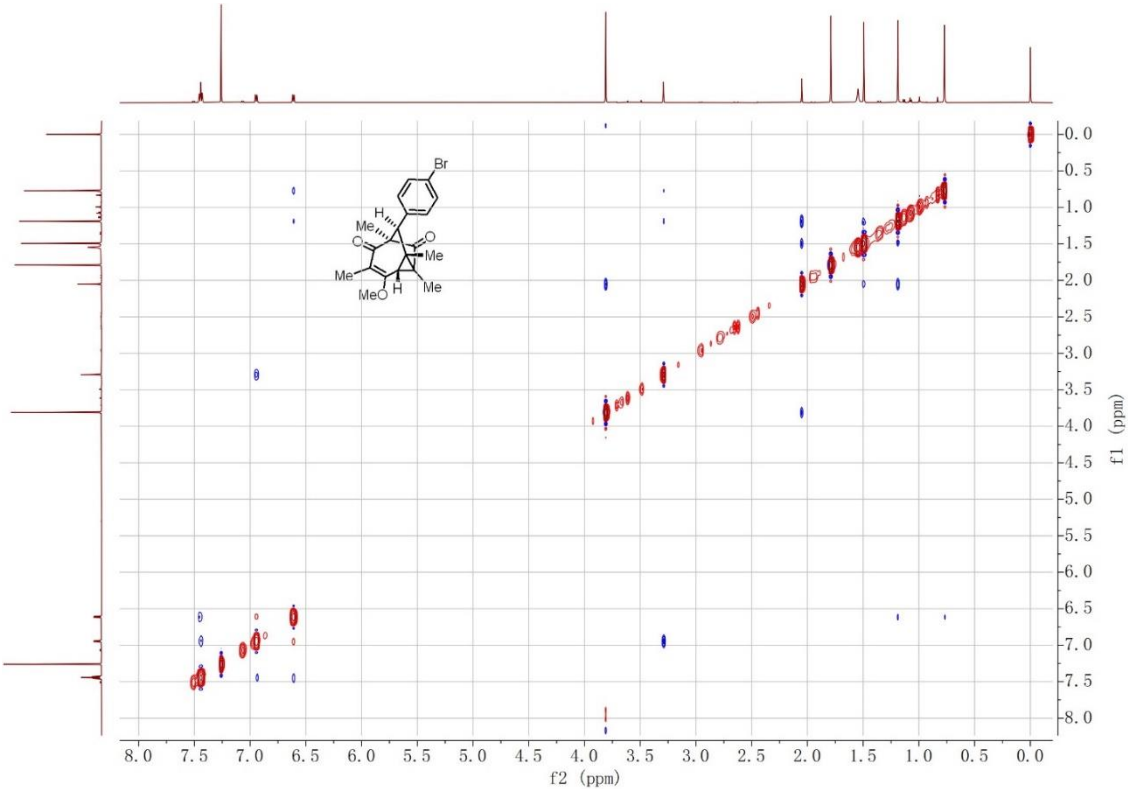


NOESY spectrum of compound **14** (600 MHz, $CDCl_3$)

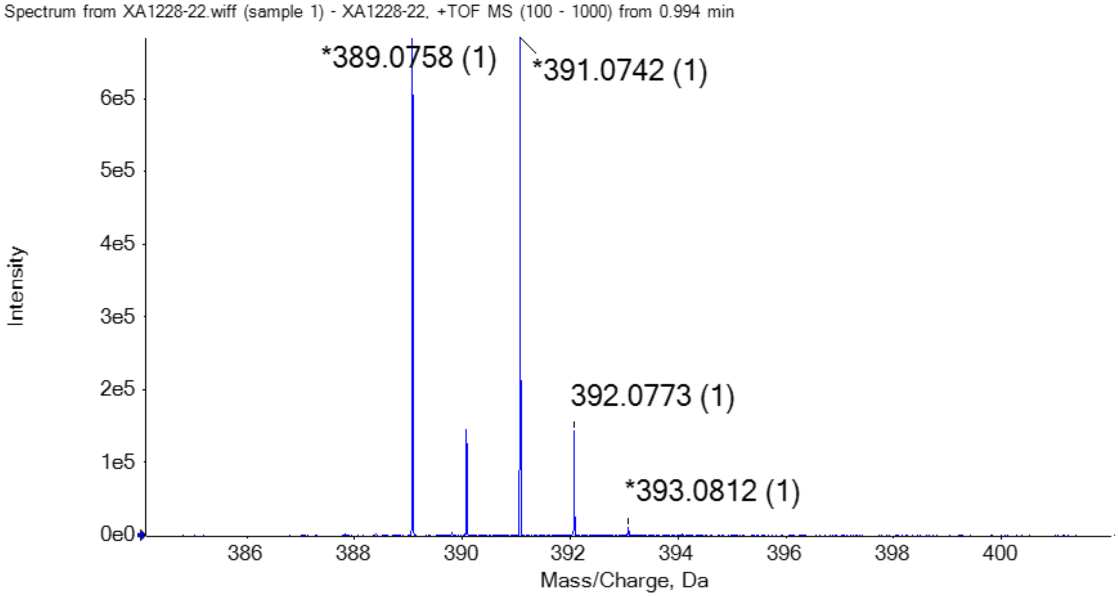


**Formula Calculator Results**

| Measure m/z | Cal m/z | Error(mmu) | Error(ppm) | Ion Formula | Ion |
|---|---|---|---|---|---|
| 389.0758 | 389.0746 | 1.1 | 2.9 | C20H22BrO3 | $[M+H]^+$ |

HRESIMS spectrum of compound **14**

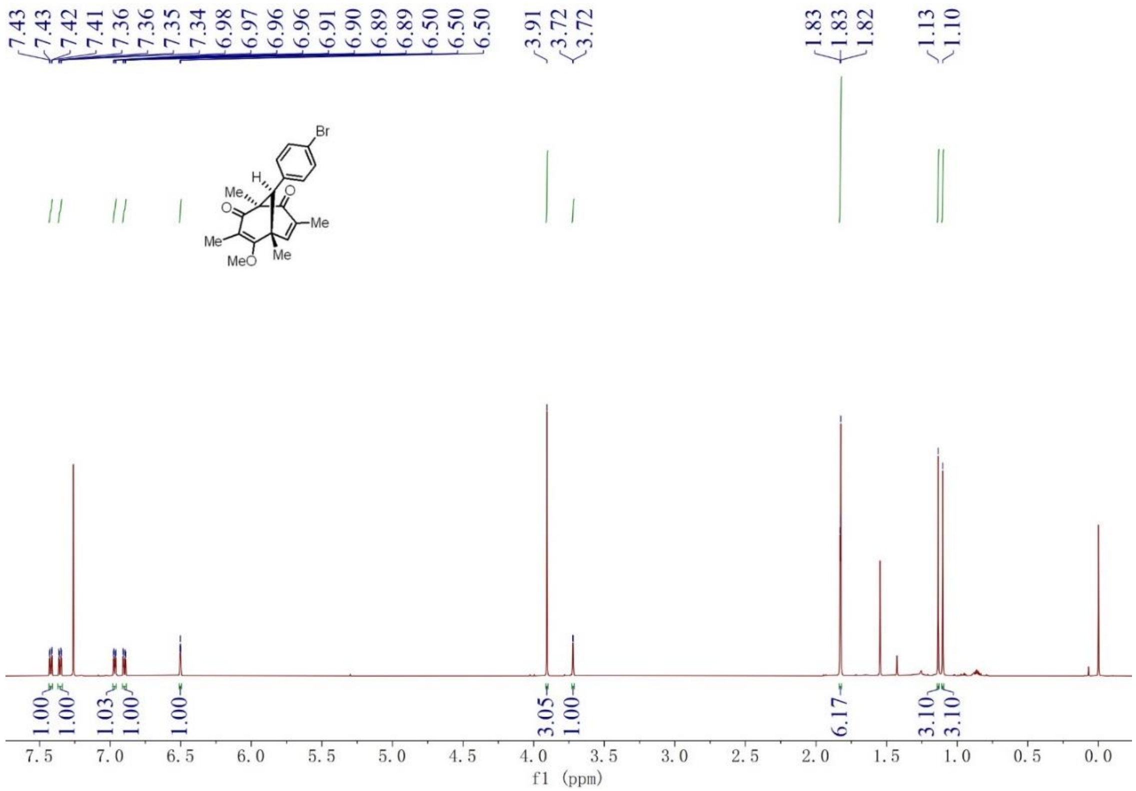

$^{1}H$ NMR spectrum of compound **15** (600MHz, $CDCl_3$)

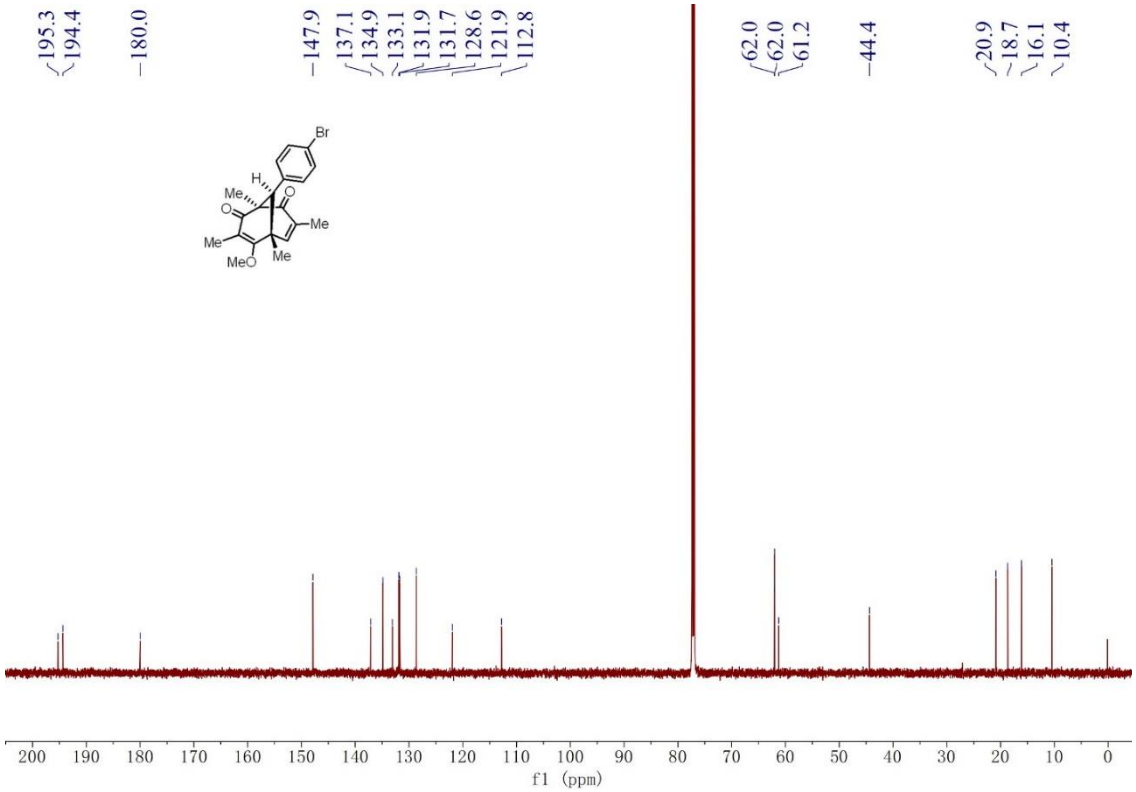

$^{13}C$ NMR spectrum of compound **15** (151MHz, $CDCl_3$)

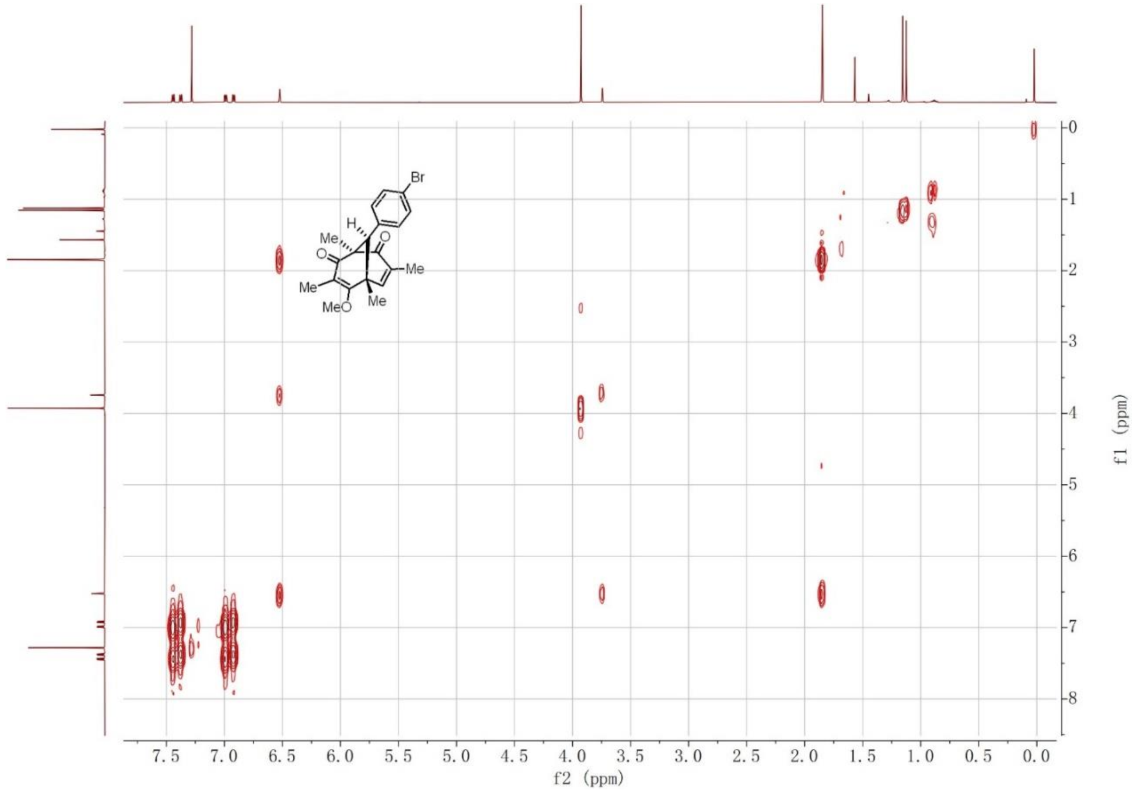


$^{1}H$-$^{1}H$ COSY spectrum of compound **15** (600 MHz, $CDCl_3$)

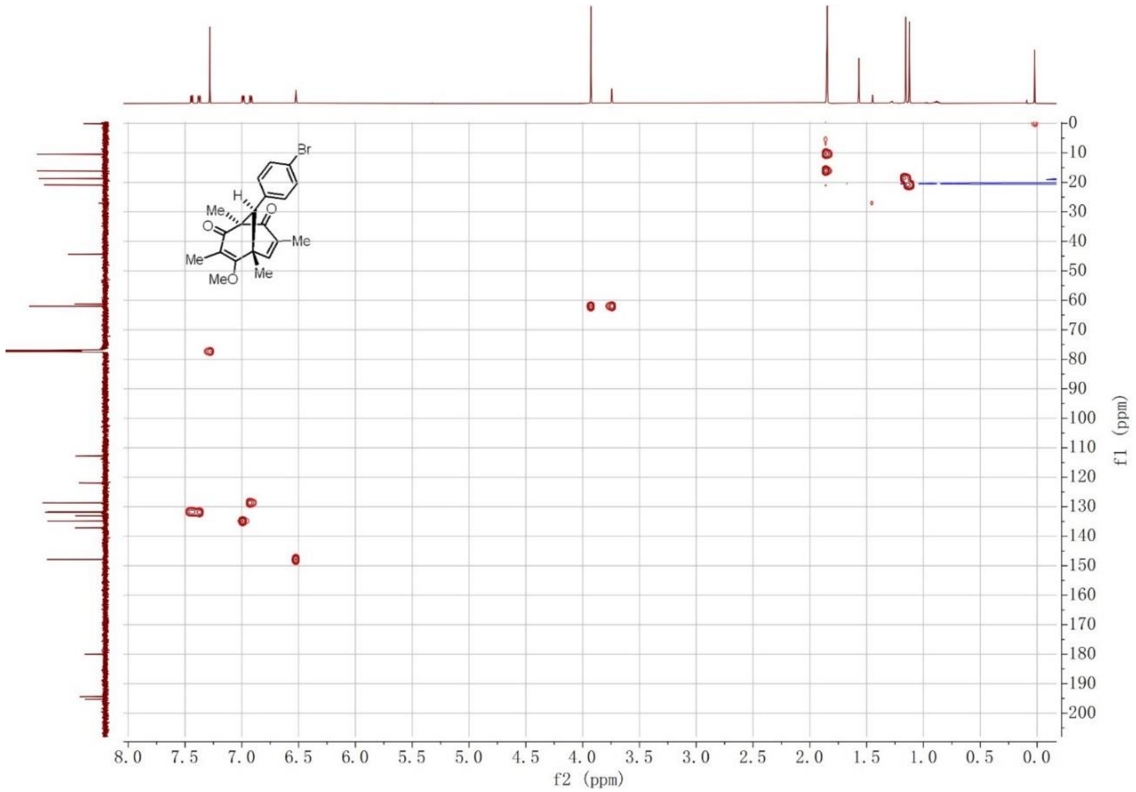


HSQC spectrum of compound **15** (600 MHz, $CDCl_3$)

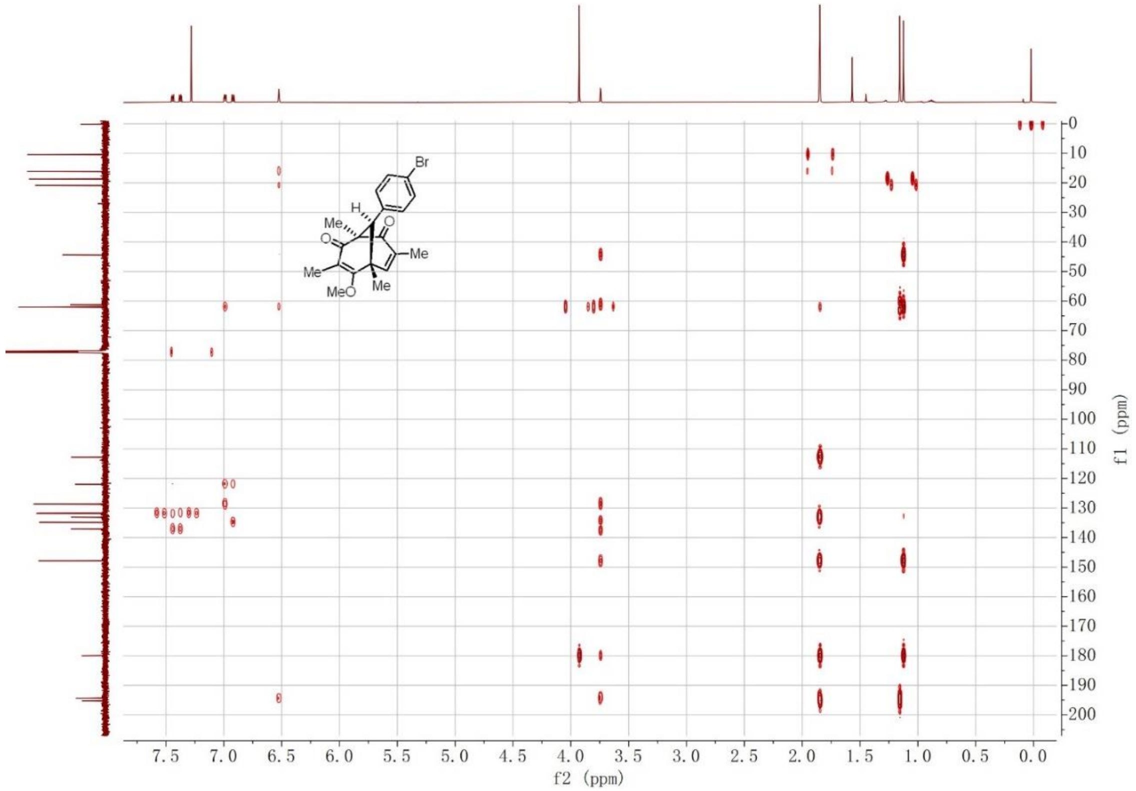


HMBC spectrum of compound **15** (600 MHz, $CDCl_3$)

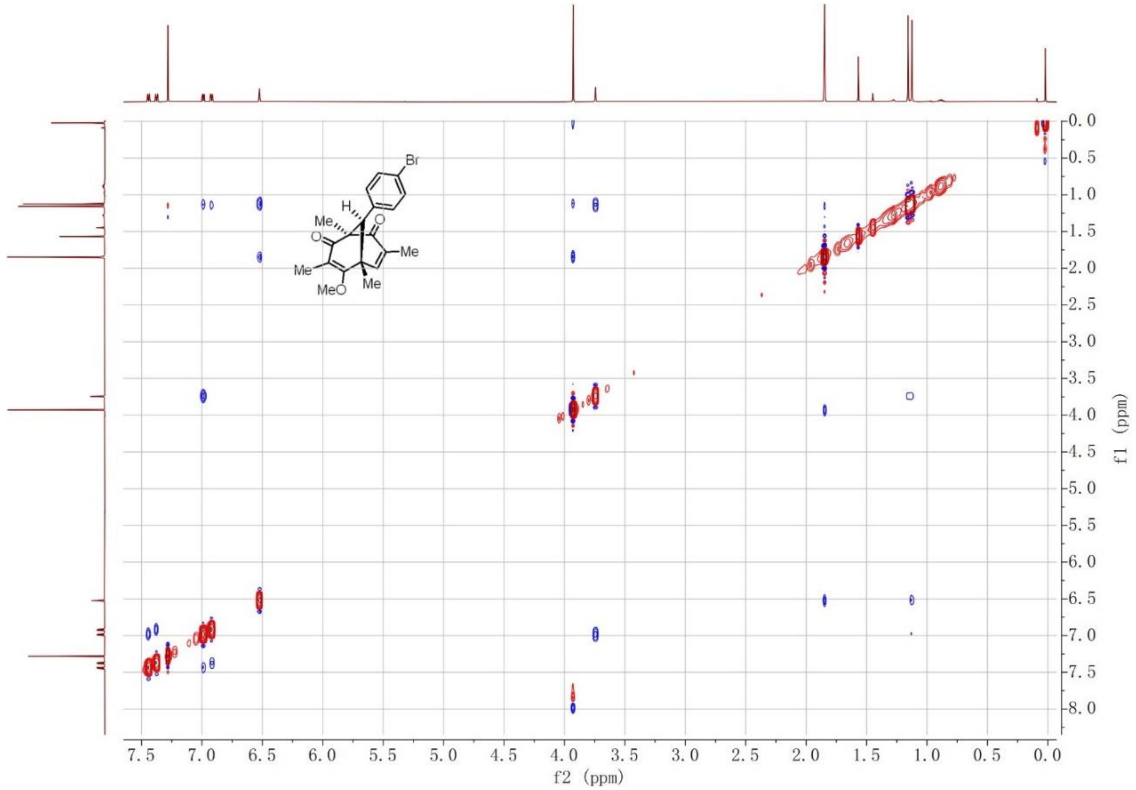


NOESY spectrum of compound **15** (600 MHz, $CDCl_3$)

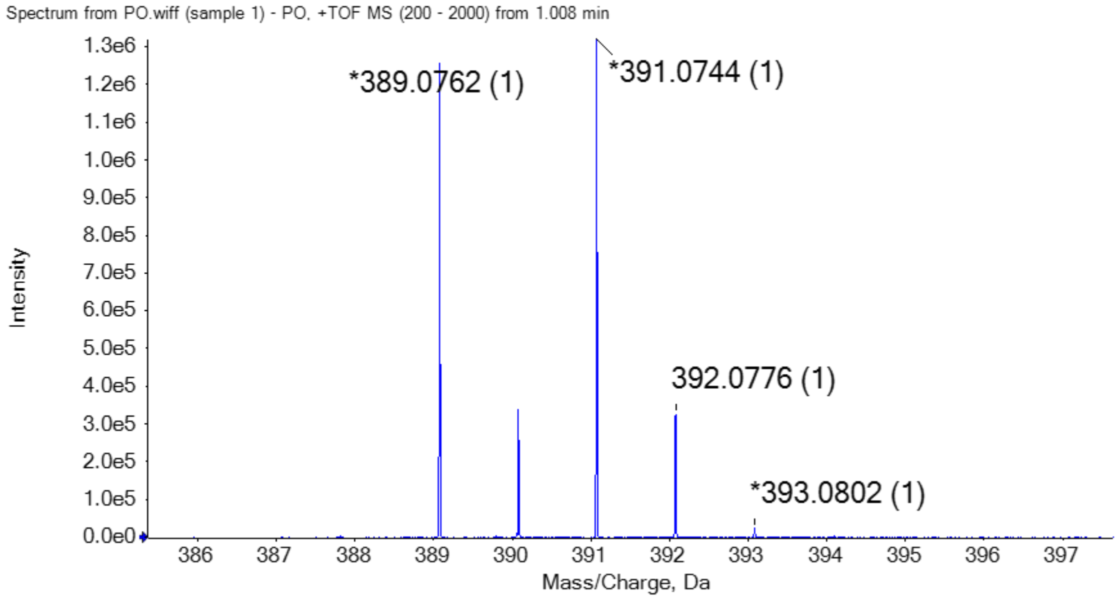


**Formula Calculator Results**

| Measure m/z | Cal m/z | Error(mmu) | Error(ppm) | Ion Formula | Ion |
|---|---|---|---|---|---|
| 389.0762 | 389.0746 | 1.5 | 3.9 | C20H22BrO3 | $[M+H]^+$ |

HRESIMS spectrum of compound **15**

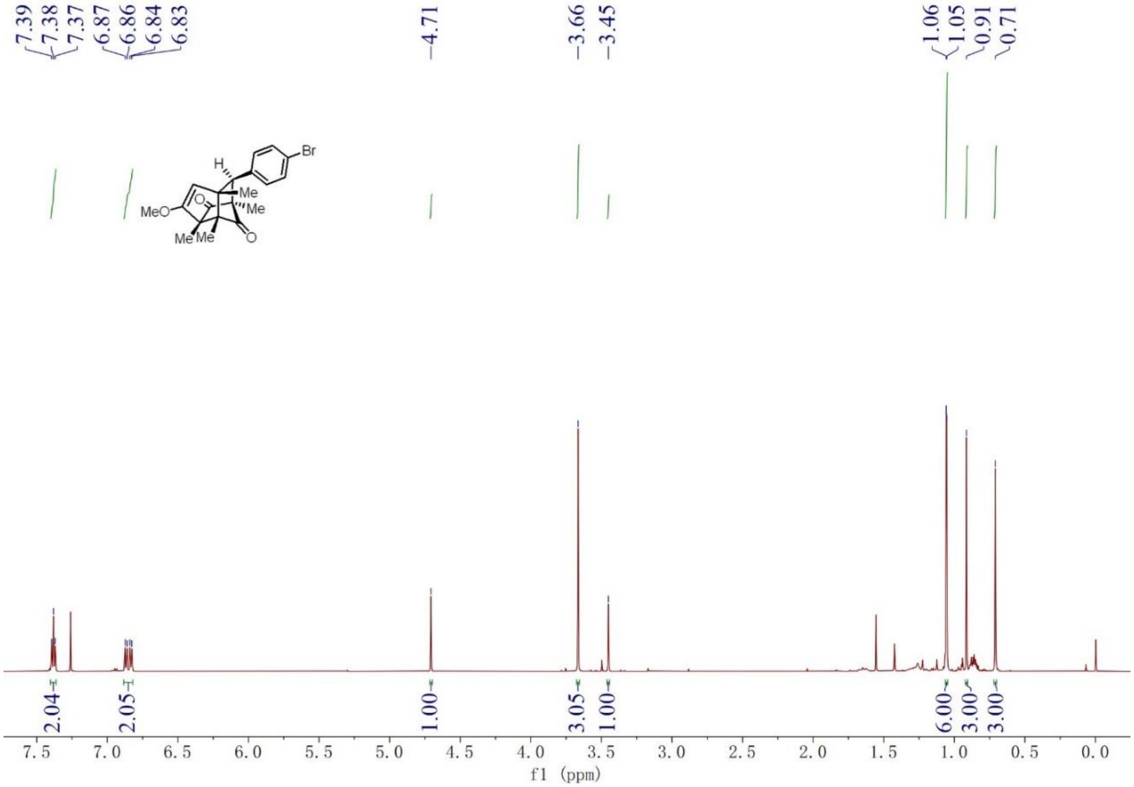


$^{1}$H NMR spectrum of compound **16** (600MHz, $CDCl_3$)

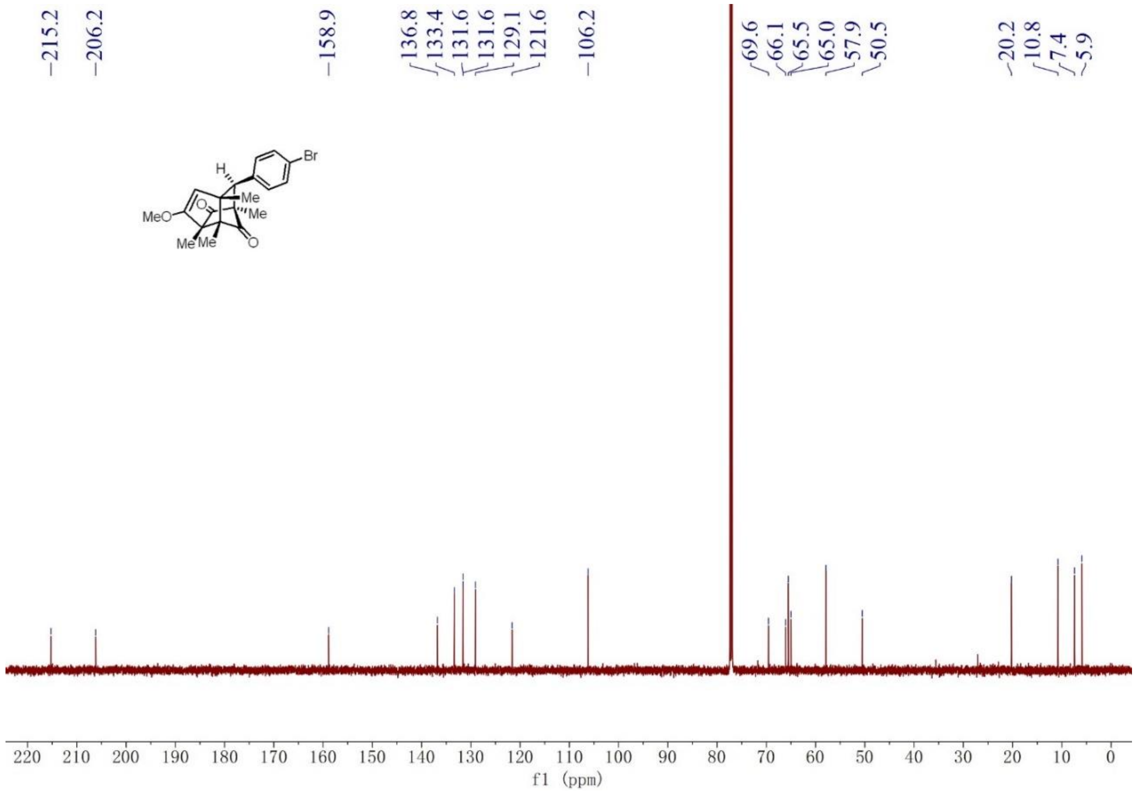


$^{13}$C NMR spectrum of compound **16** (151MHz, $CDCl_3$)

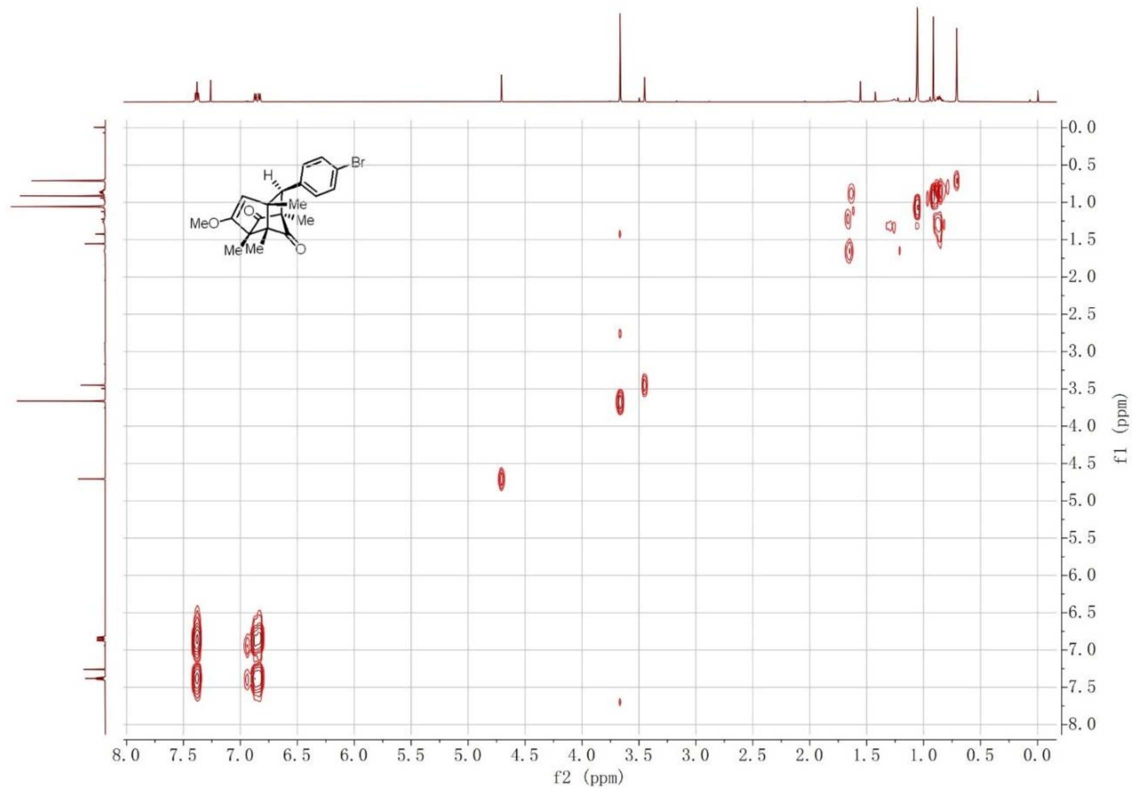


$^{1}$H-$^{1}$H COSY spectrum of compound **16** (600 MHz, $CDCl_3$)

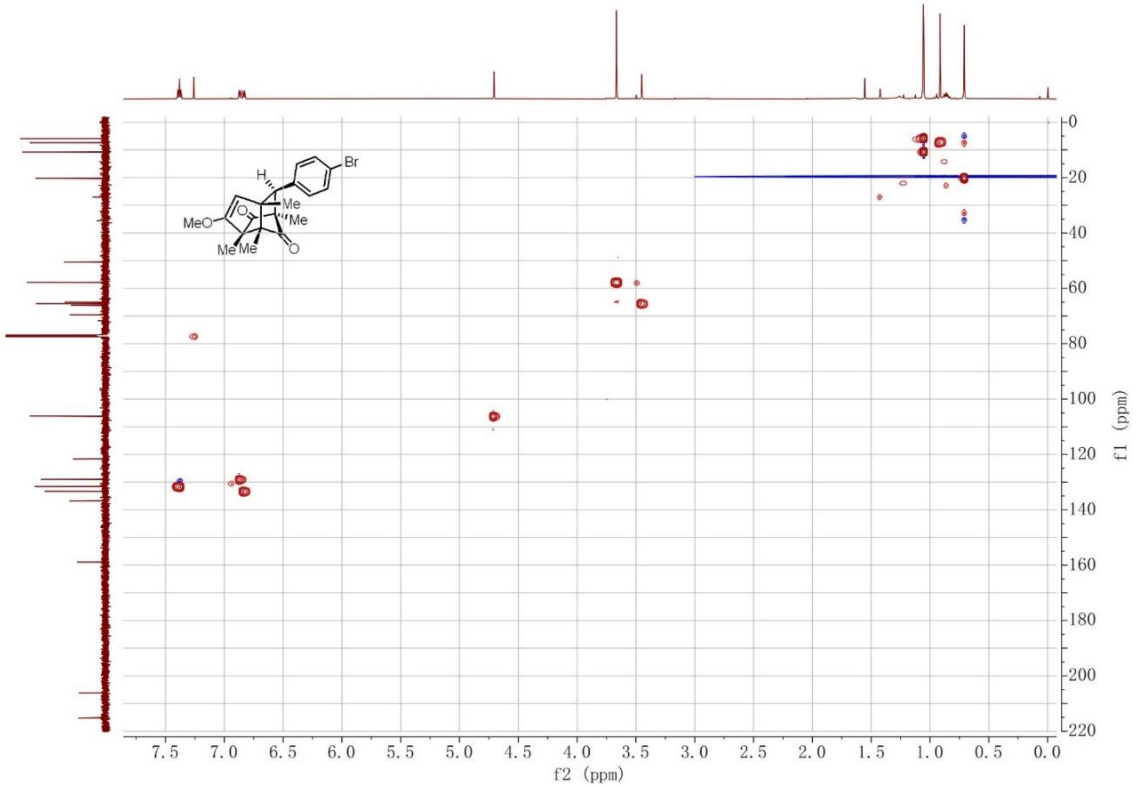

HSQC spectrum of compound **16** (600 MHz, $CDCl_3$)

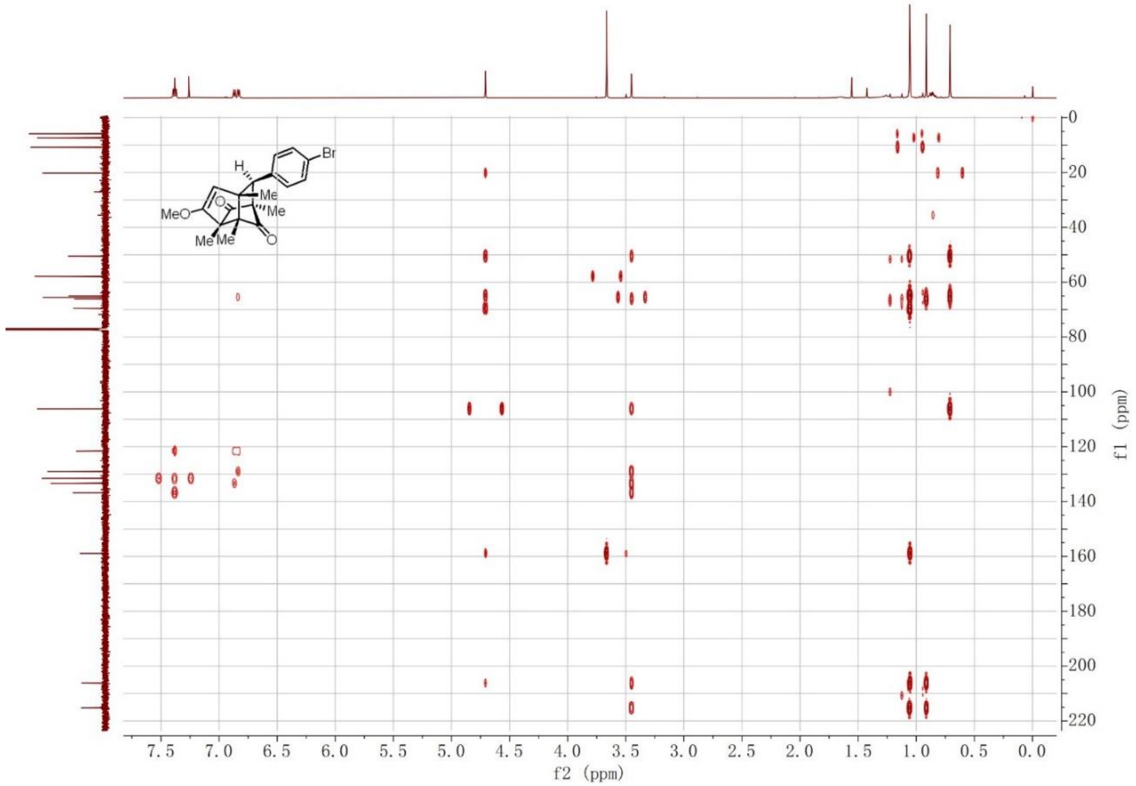

HMBC spectrum of compound **16** (600 MHz, $CDCl_3$)

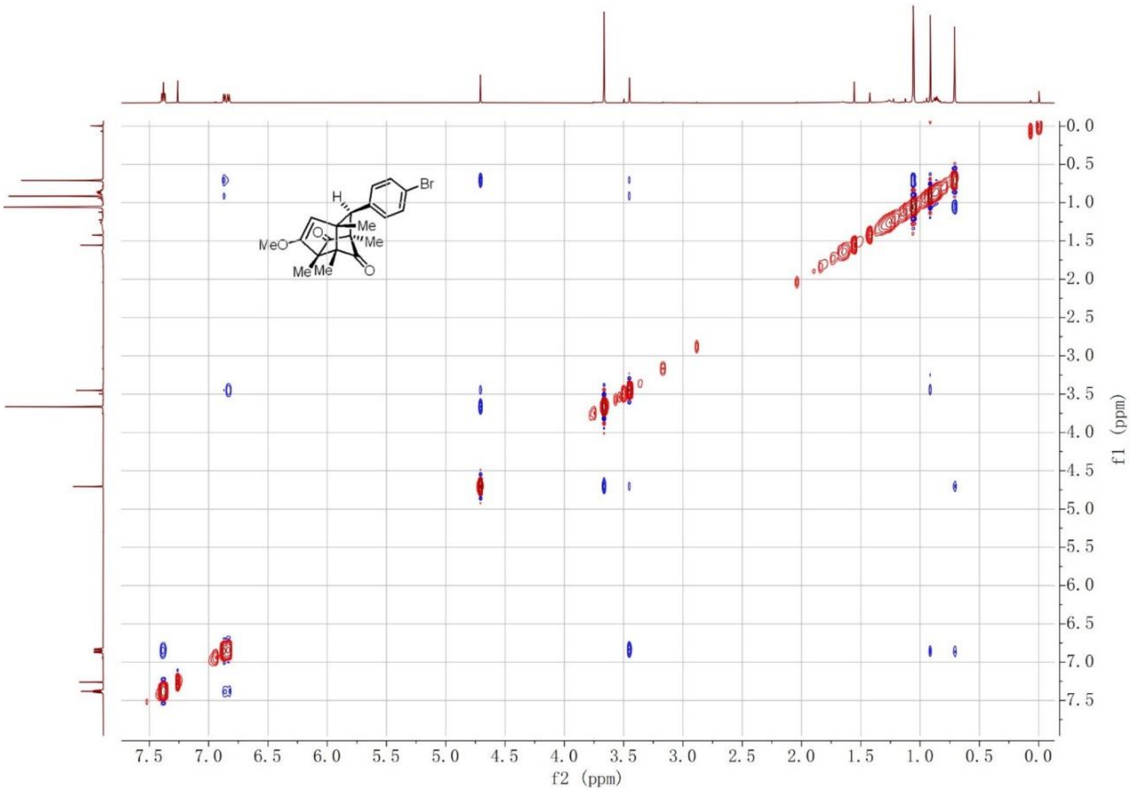

NOESY spectrum of compound **16** (600 MHz, $CDCl_3$)

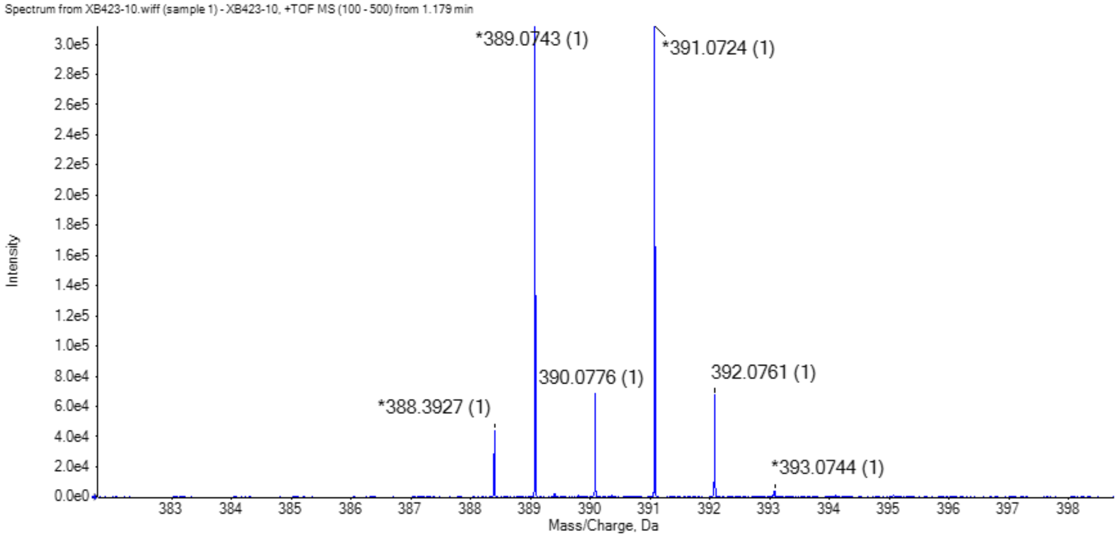


**Formula Calculator Results**

| Measure m/z | Cal m/z | Error(mmu) | Error(ppm) | Ion Formula | Ion |
|---|---|---|---|---|---|
| 389.0743 | 389.0746 | -0.4 | -1.0 | C20H22BrO3 | $[M+H]^+$ |

HRESIMS spectrum of compound **16**

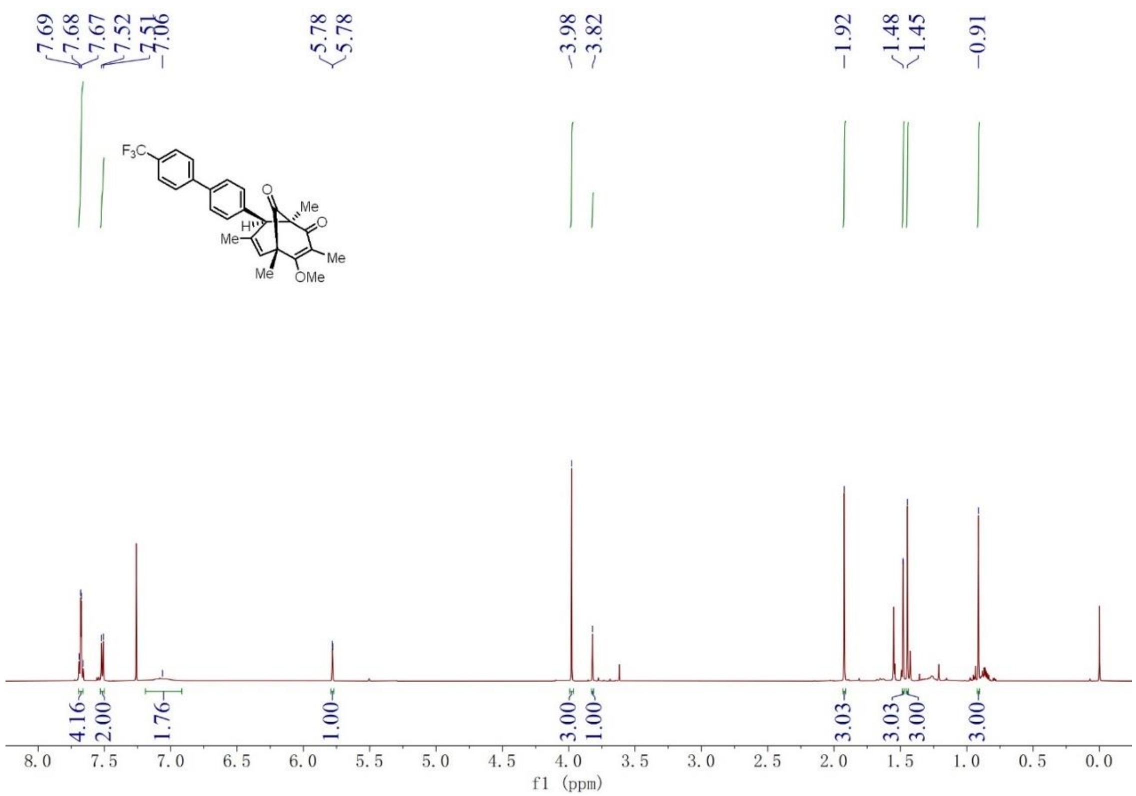


$^{1}$H NMR spectrum of compound **17a** (600MHz, $CDCl_3$)

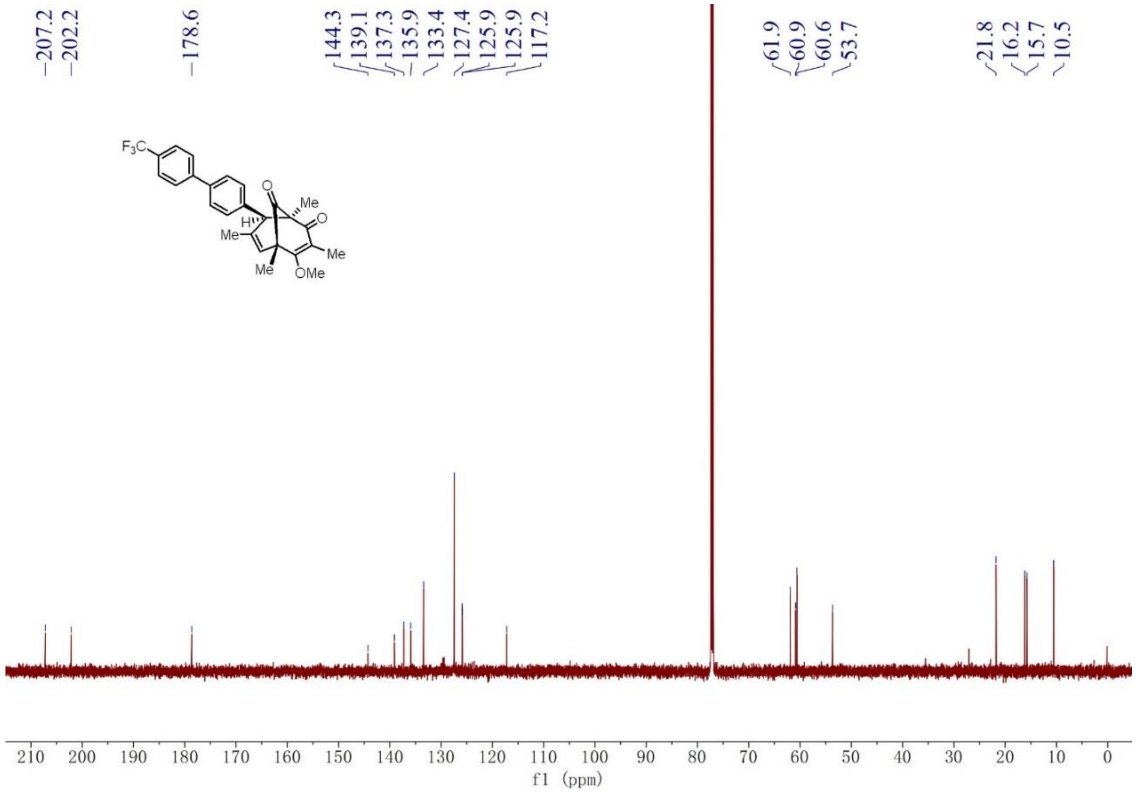


$^{13}$C NMR spectrum of compound **17a** (151MHz, $CDCl_3$)

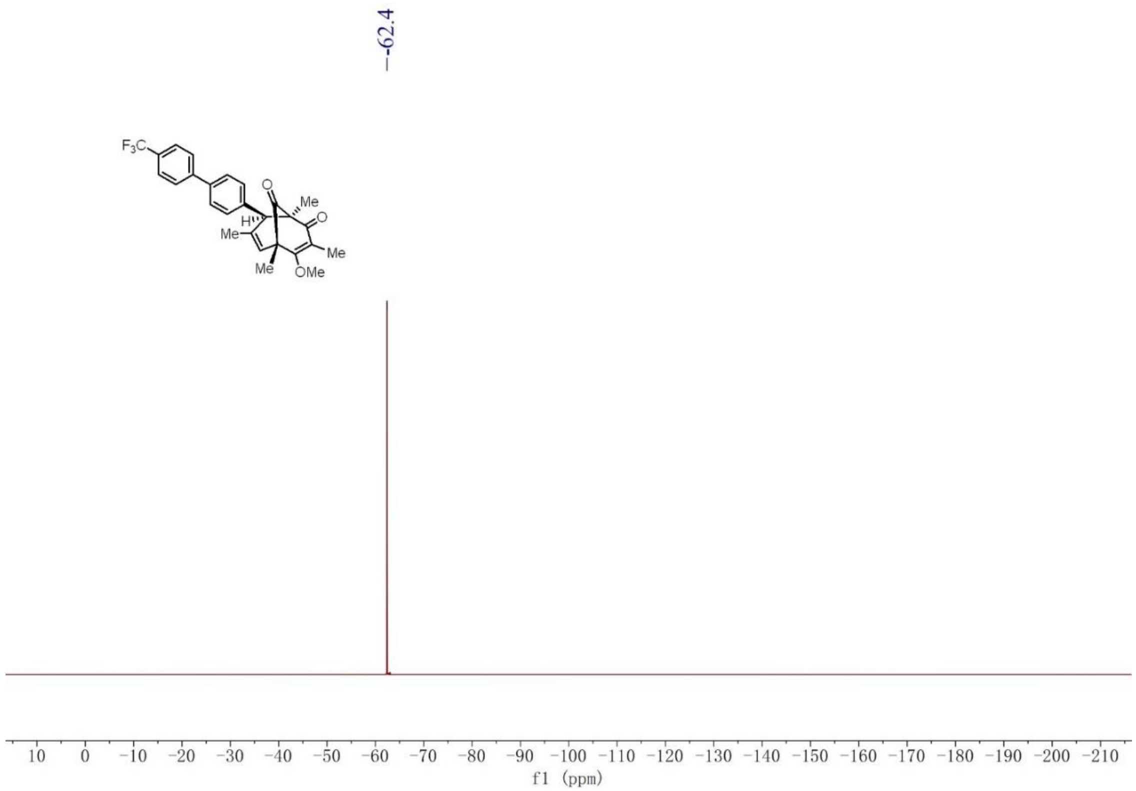


$^{19}$F NMR spectrum of compound **17a** (565MHz, $CDCl_3$)

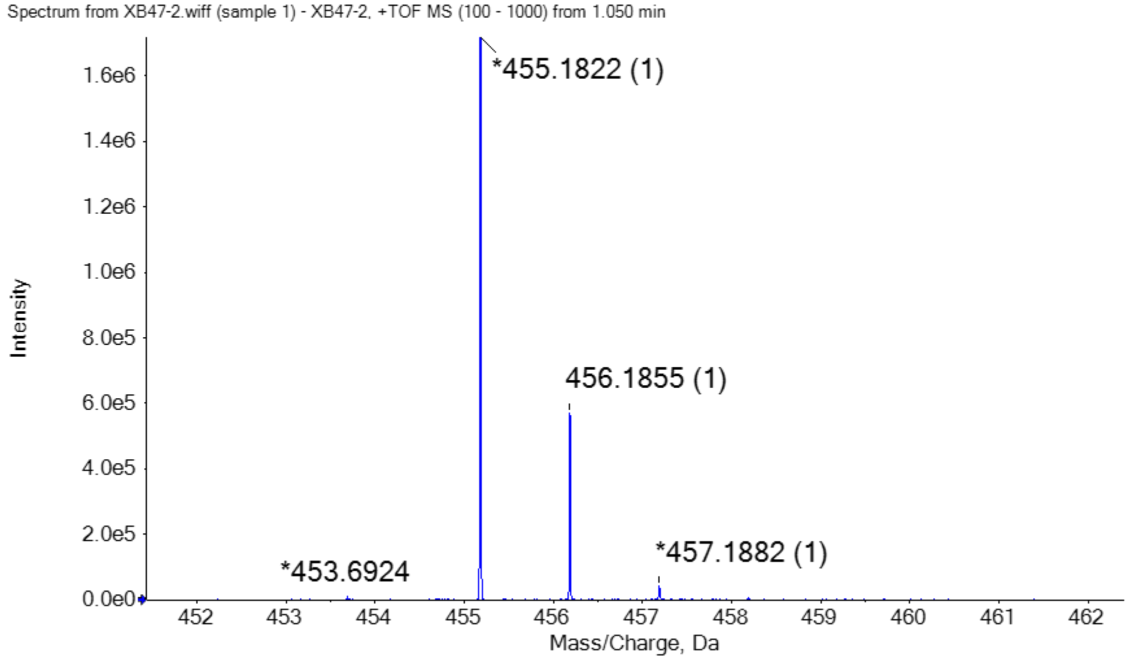


**Formula Calculator Results**

| Measured m/z | Cal m/z | Error(mmu) | Error(ppm) | Ion Formula | Ion |
|---|---|---|---|---|---|
| 455.1822 | 455.1828 | -0.7 | -1.4 | C27H26F3O3 | $[M+H]^+$ |

HRESIMS spectrum of compound **17a**

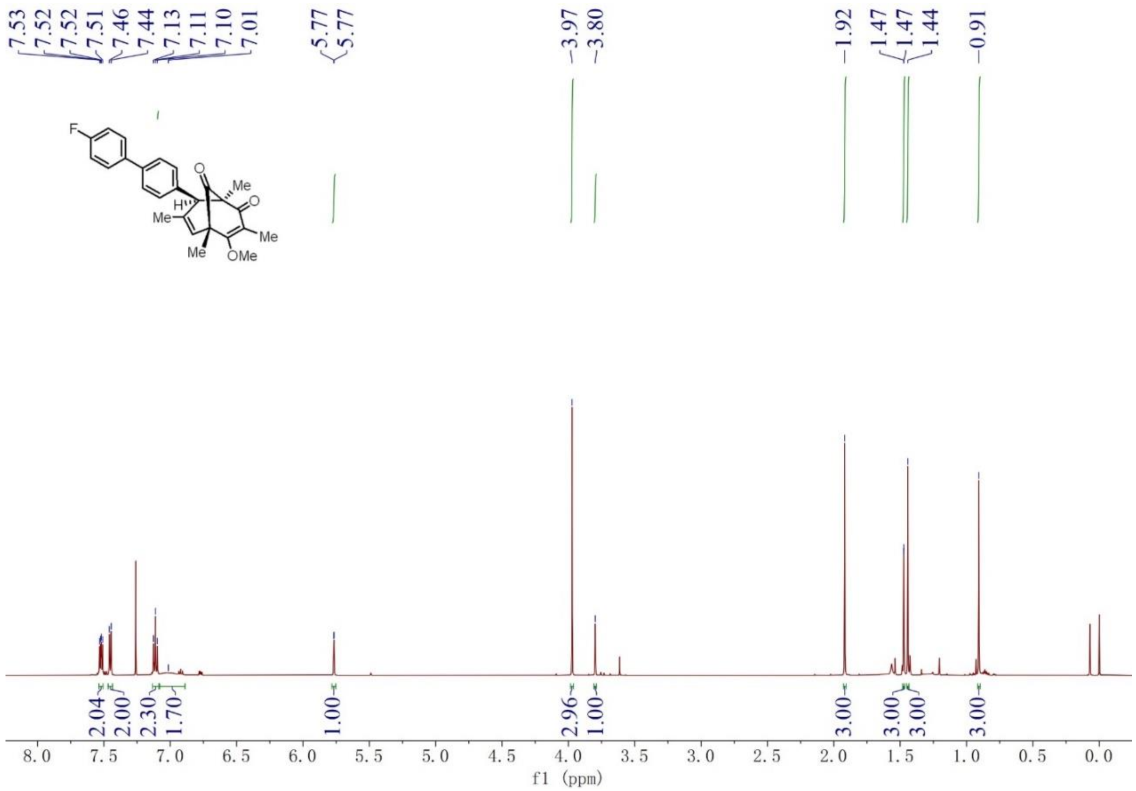

$^{1}$H NMR spectrum of compound **17b** (600MHz, $CDCl_3$)

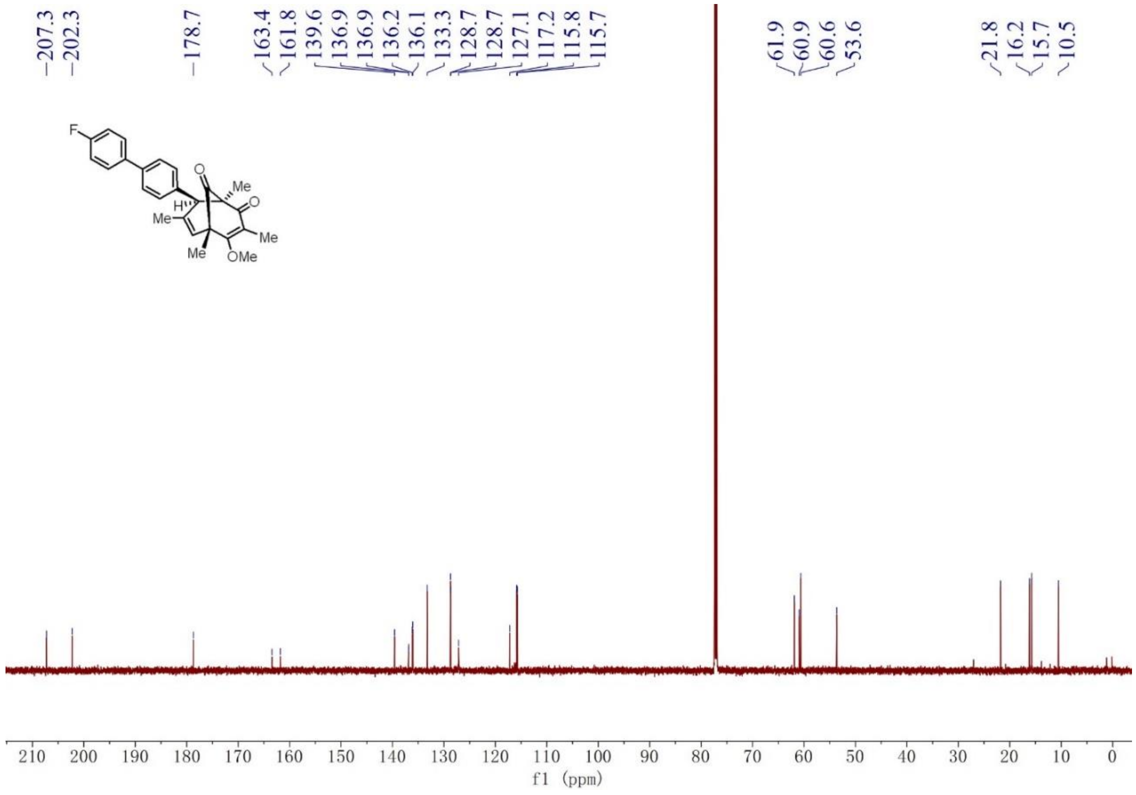

$^{13}$C NMR spectrum of compound **17b** (151MHz, $CDCl_3$)

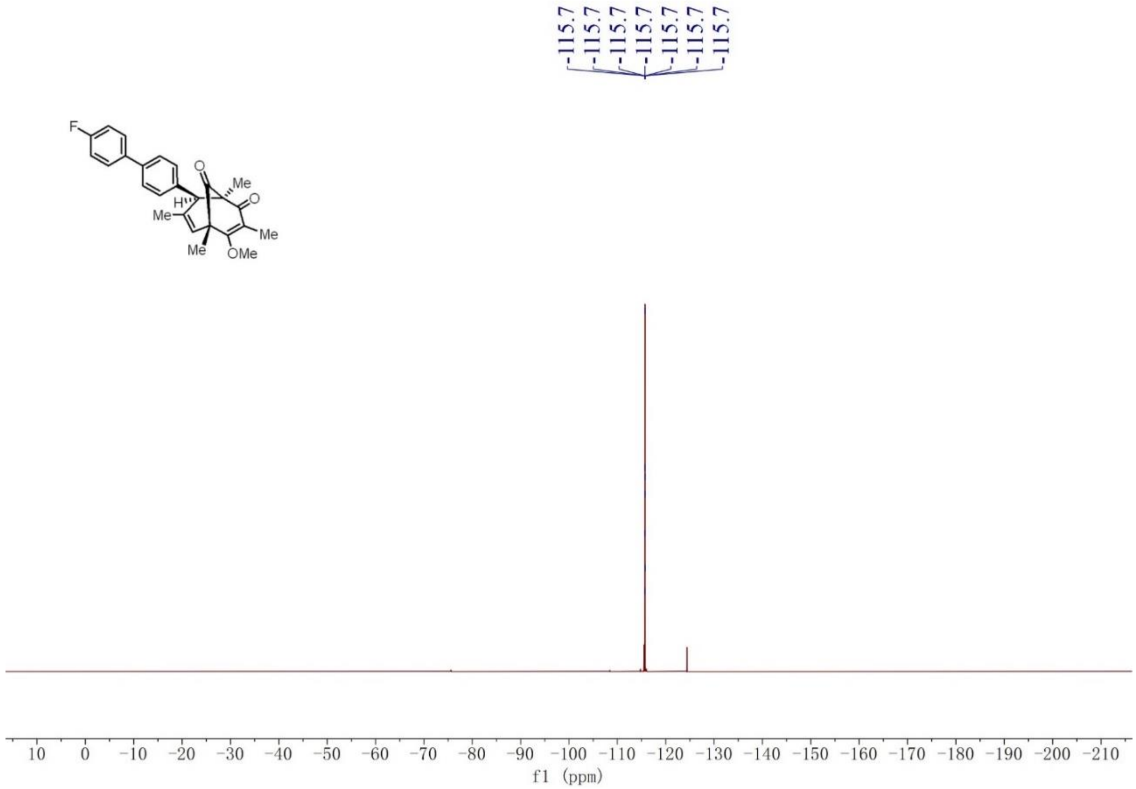


$^{19}$F NMR spectrum of compound **17b** (565MHz, $CDCl_3$)

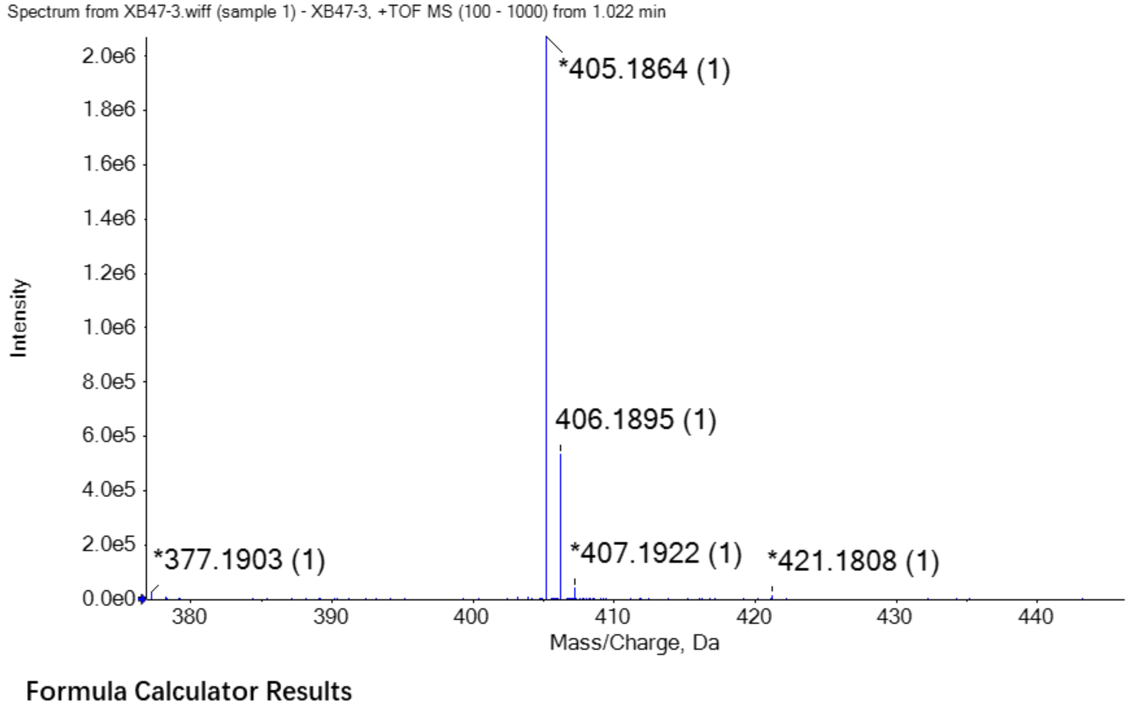


**Formula Calculator Results**

| Measured m/z | Cal m/z | Error(mmu) | Error(ppm) | Ion Formula | Ion |
|---|---|---|---|---|---|
| 405.1864 | 405.1860 | 0.3 | 0.9 | C26H26FO3 | $[M+H]^+$ |

HRESIMS spectrum of compound **17b**

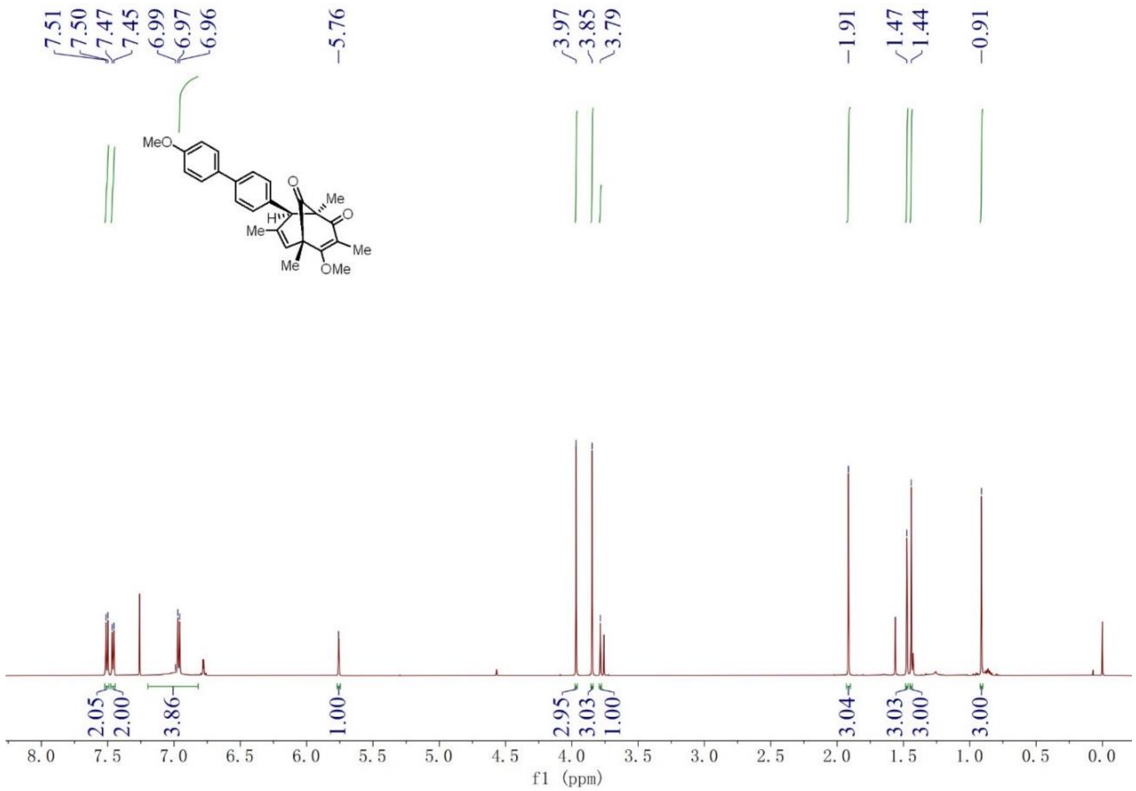

$^1H$ NMR spectrum of compound **17c** (600MHz, $CDCl_3$)

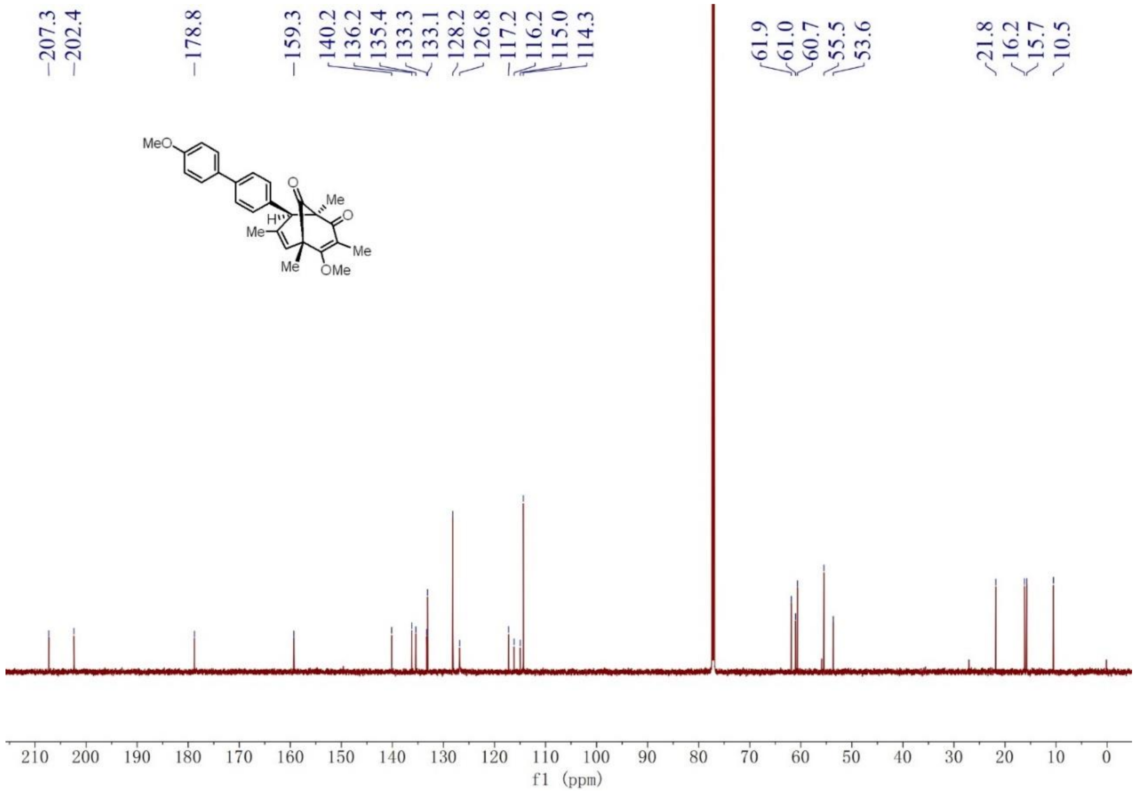

$^{13}C$ NMR spectrum of compound **17c** (151MHz, $CDCl_3$)

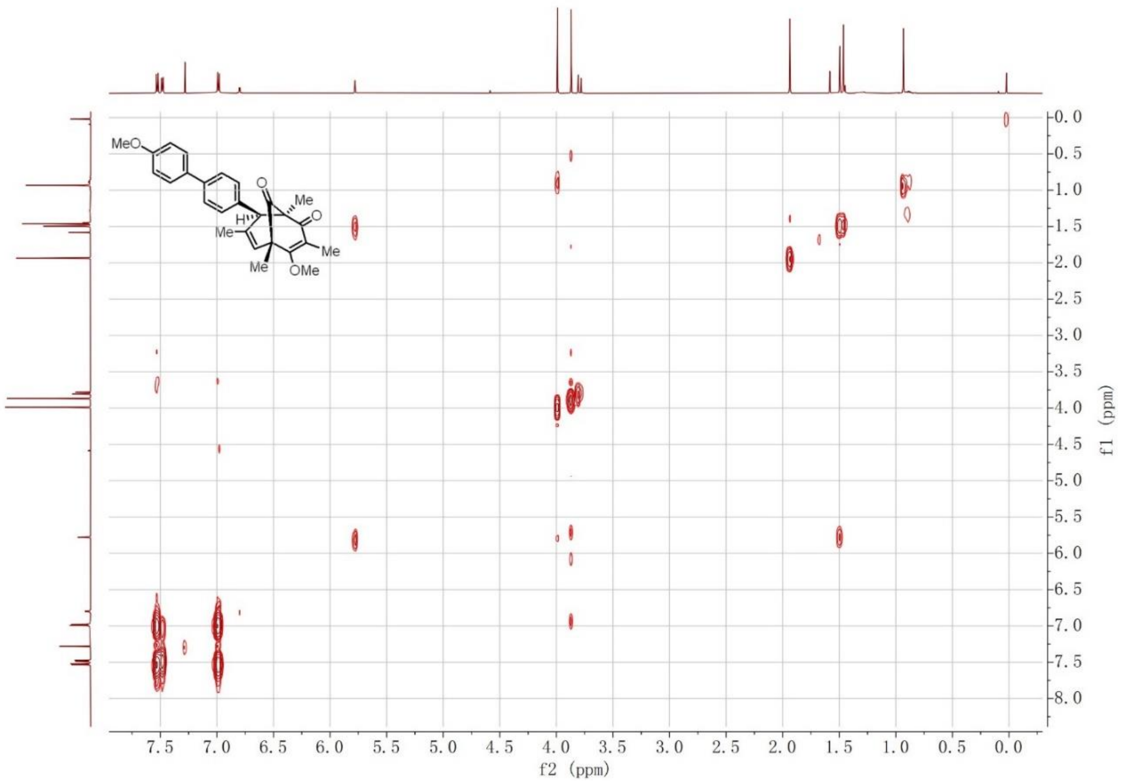

$^1$H-$^1$H COSY spectrum of compound **17c** (600 MHz, $CDCl_3$)

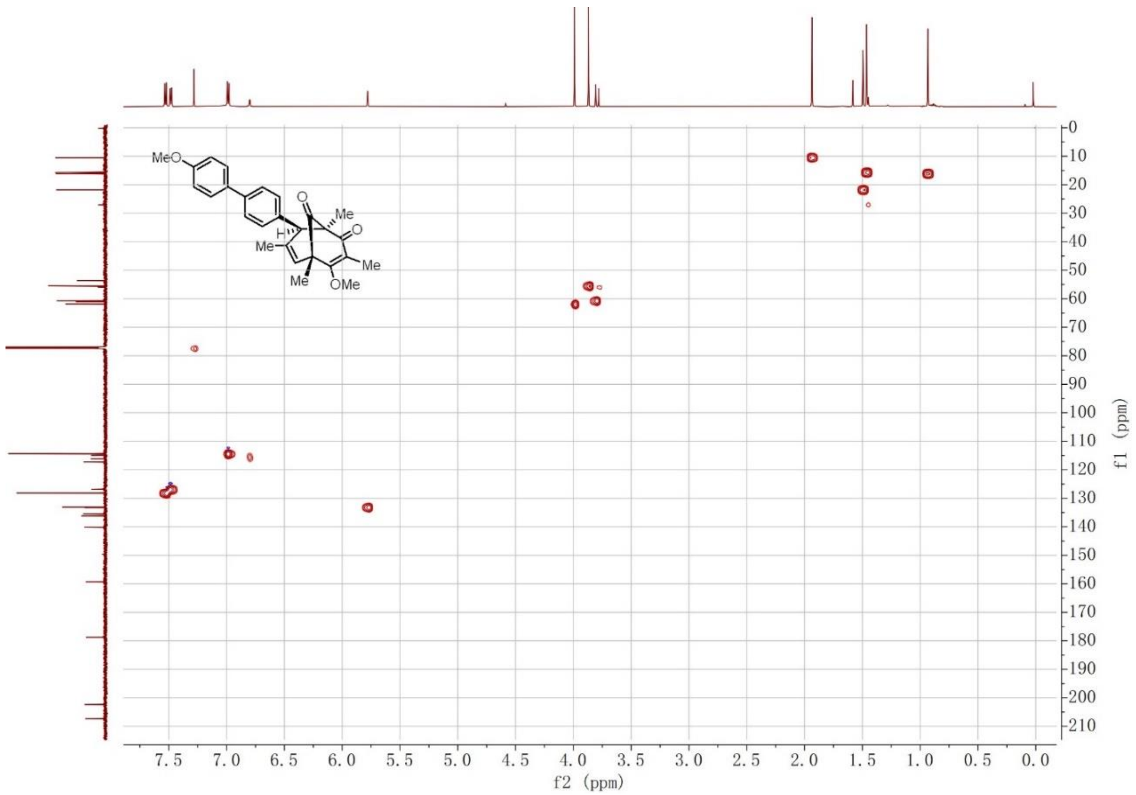

HSQC spectrum of compound **17c** (600 MHz, $CDCl_3$)

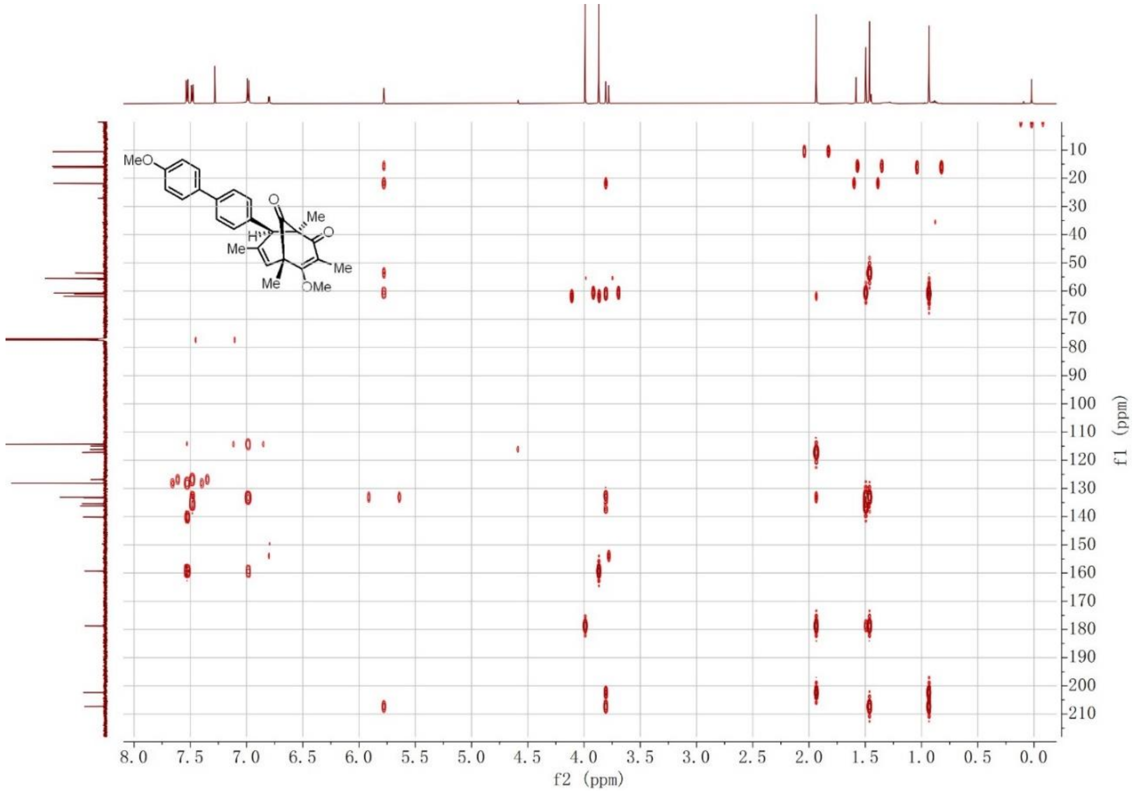


HMBC spectrum of compound **17c** (600 MHz, $CDCl_3$)

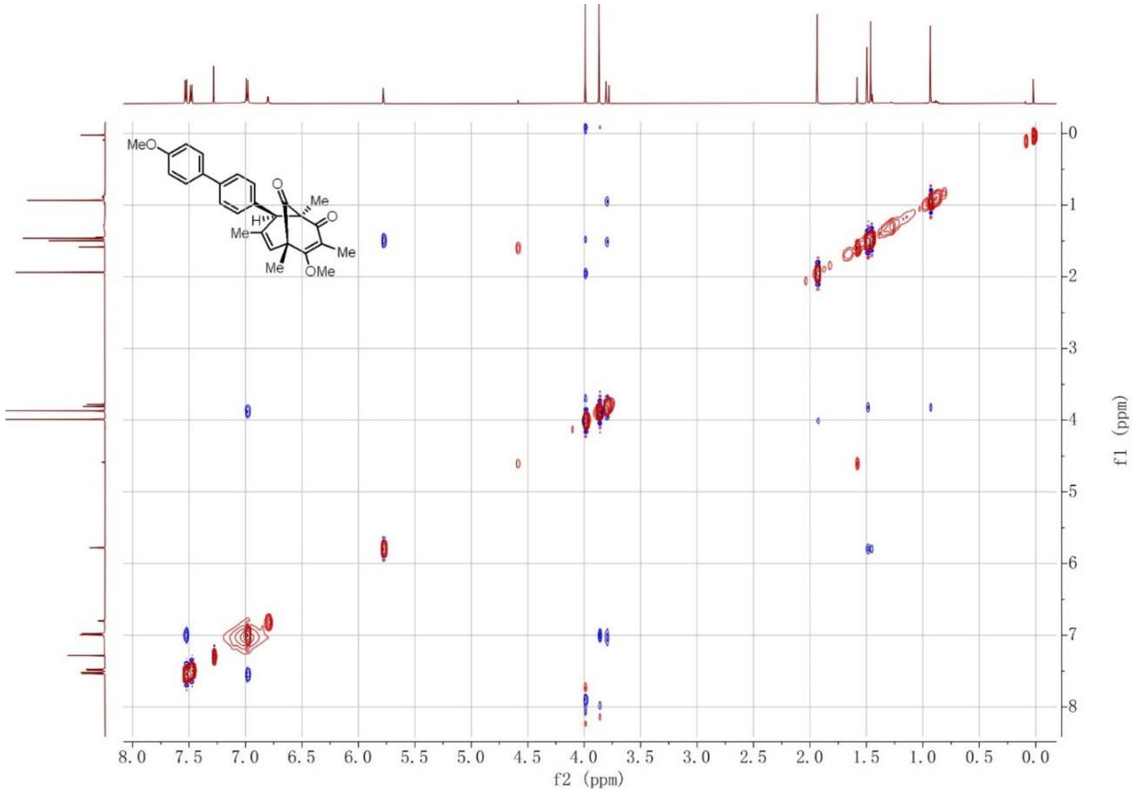


NOESY spectrum of compound **17c** (600 MHz, $CDCl_3$)

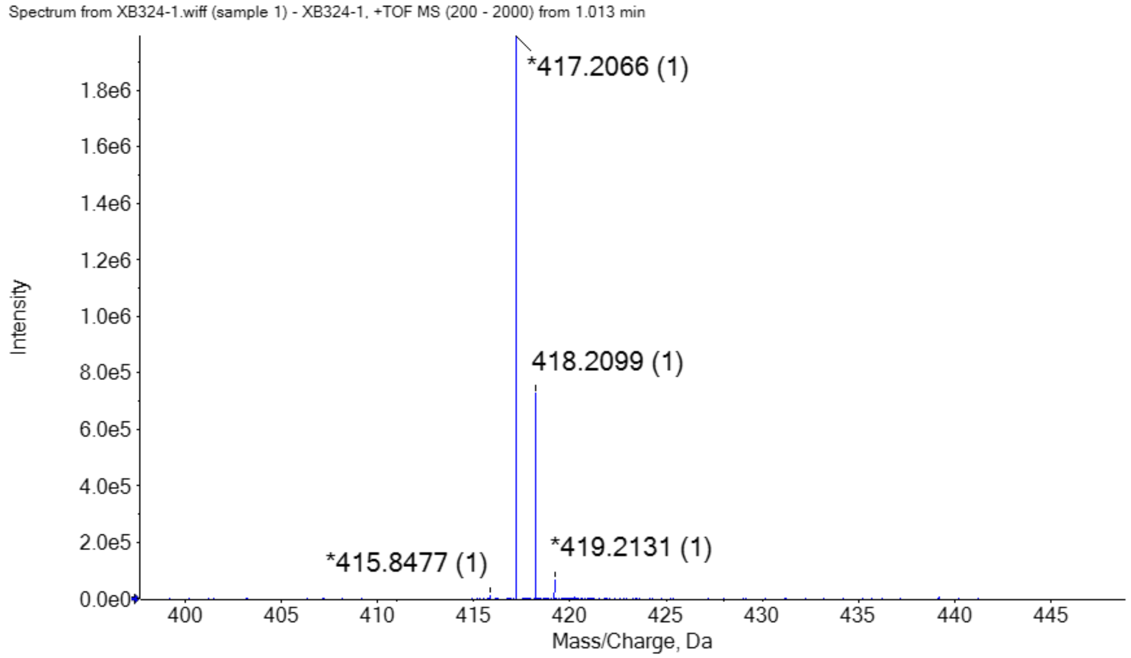


**Formula Calculator Results**

| Measured m/z | Cal m/z | Error(mmu) | Error(ppm) | Ion Formula | Ion |
|---|---|---|---|---|---|
| 417.2066 | 417.2060 | 0.6 | 1.3 | C27H29O4 | $[M+H]^+$ |

HRESIMS spectrum of compound **17c**

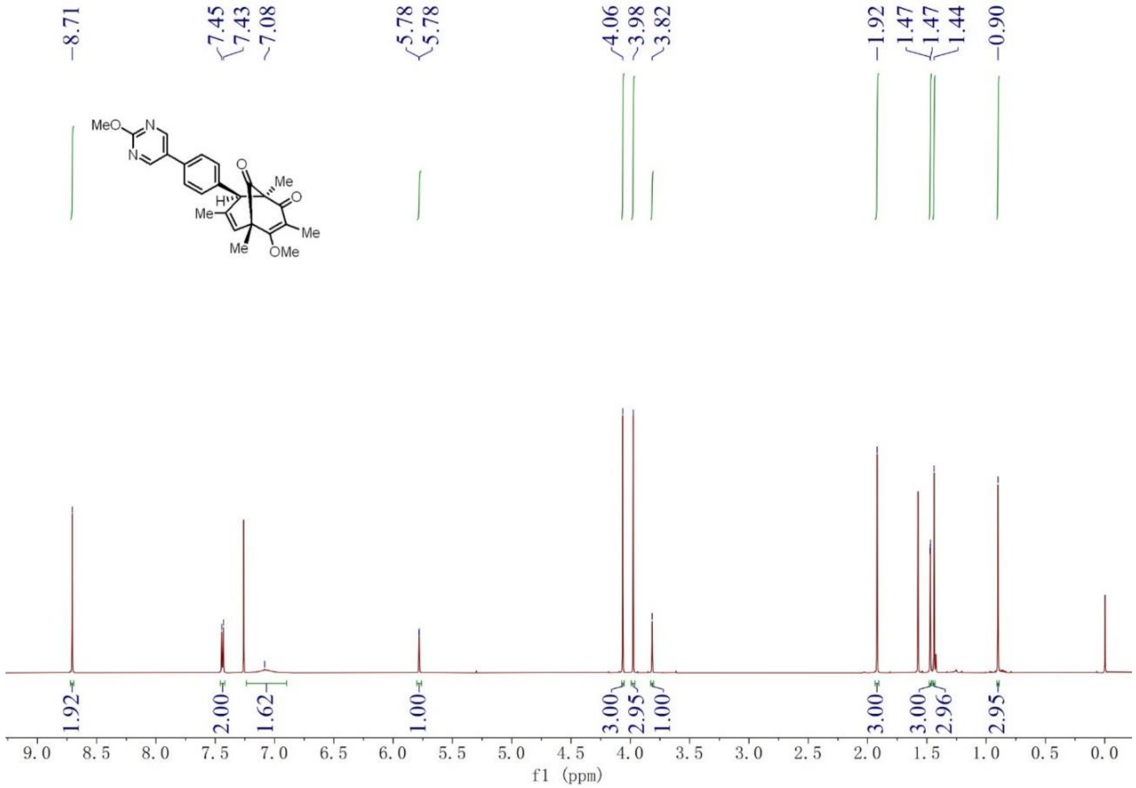


$^{1}$H NMR spectrum of compound **17d** (600MHz, $CDCl_3$)

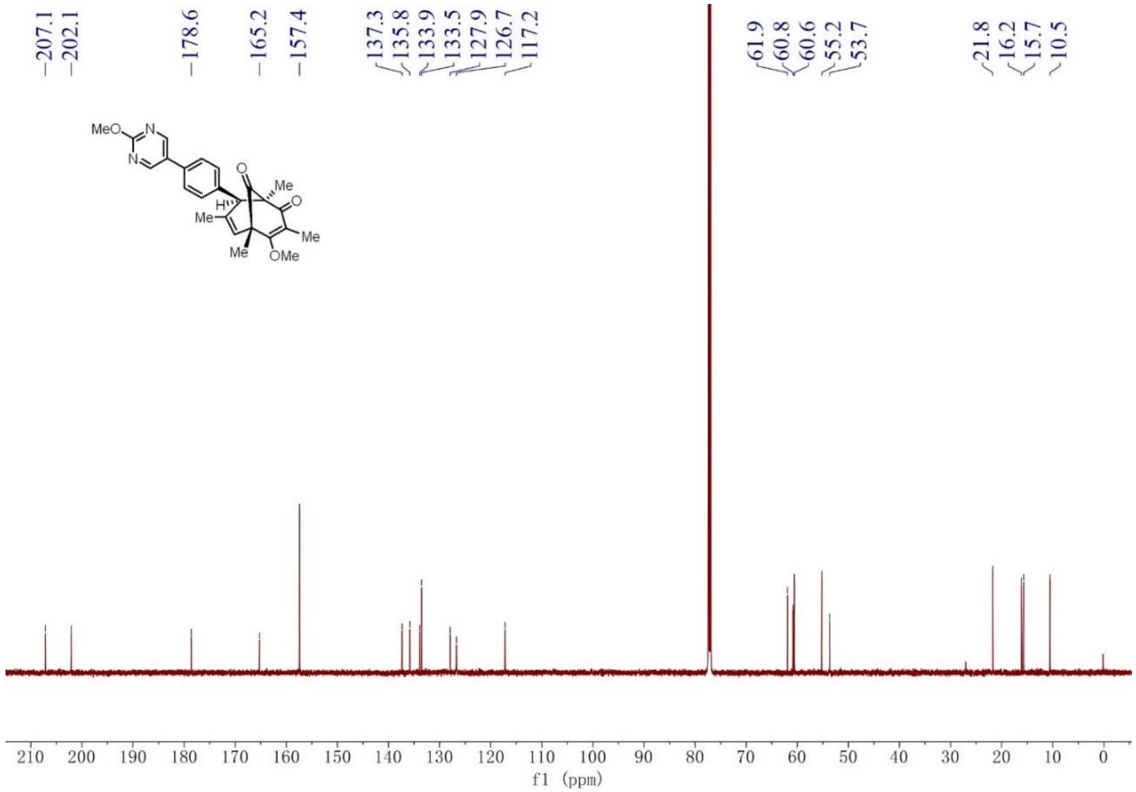


$^{13}C$ NMR spectrum of compound **17d** (151MHz, $CDCl_3$)

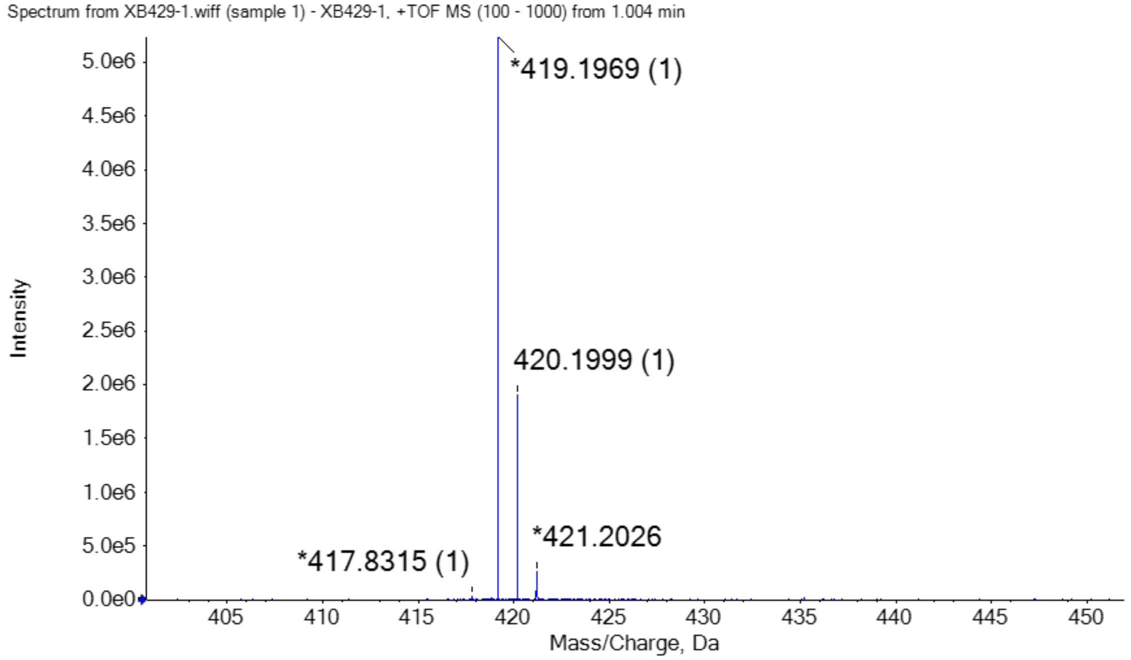


**Formula Calculator Results**

| Measured m/z | Cal m/z | Error(mmu) | Error(ppm) | Ion Formula | Ion |
|---|---|---|---|---|---|
| 419.1969 | 419.1965 | 0.4 | 0.9 | C25H27N2O4 | $[M+H]^+$ |

HRESIMS spectrum of compound **17d**

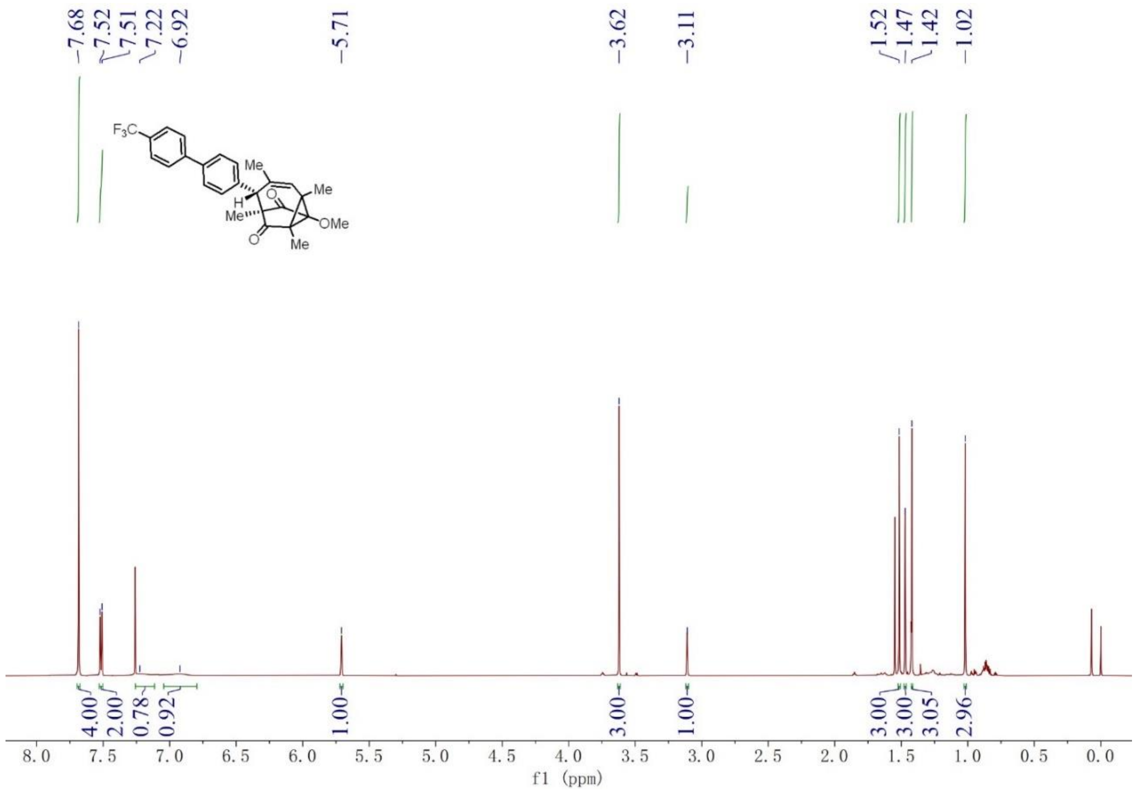


$^{1}H$ NMR spectrum of compound **19a** (600MHz, $CDCl_3$)

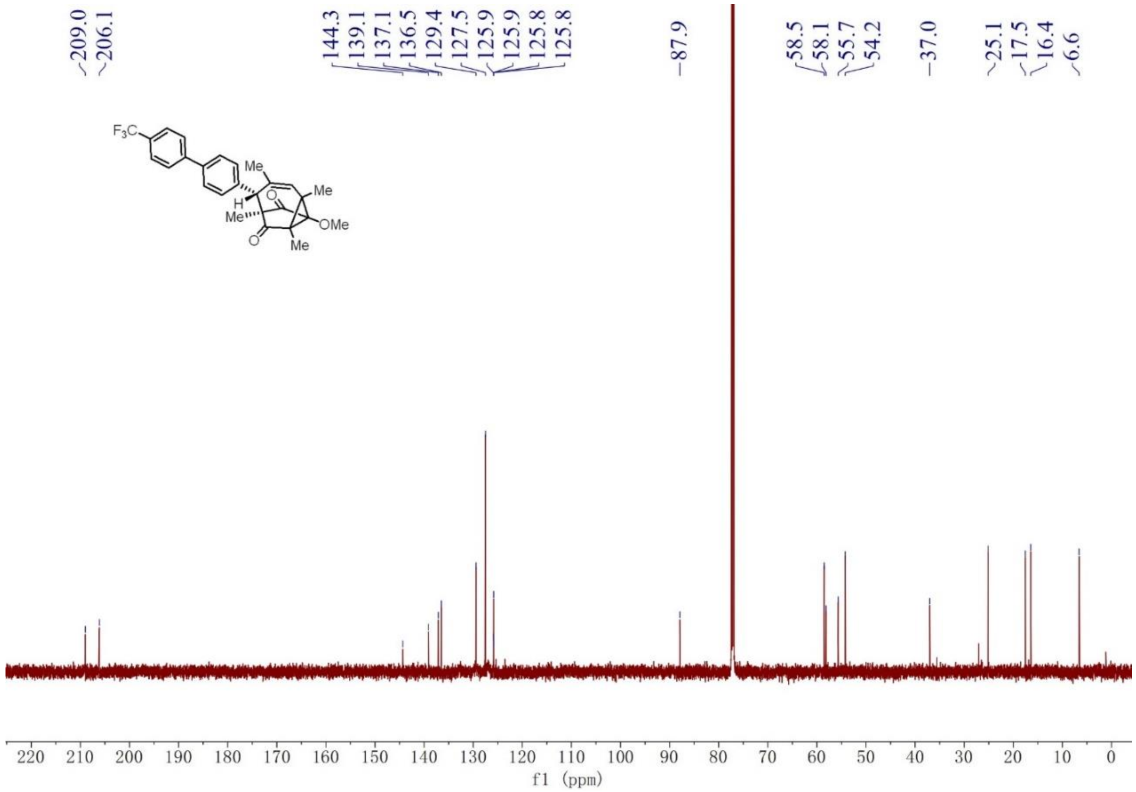


$^{13}C$ NMR spectrum of compound **19a** (151MHz, $CDCl_3$)

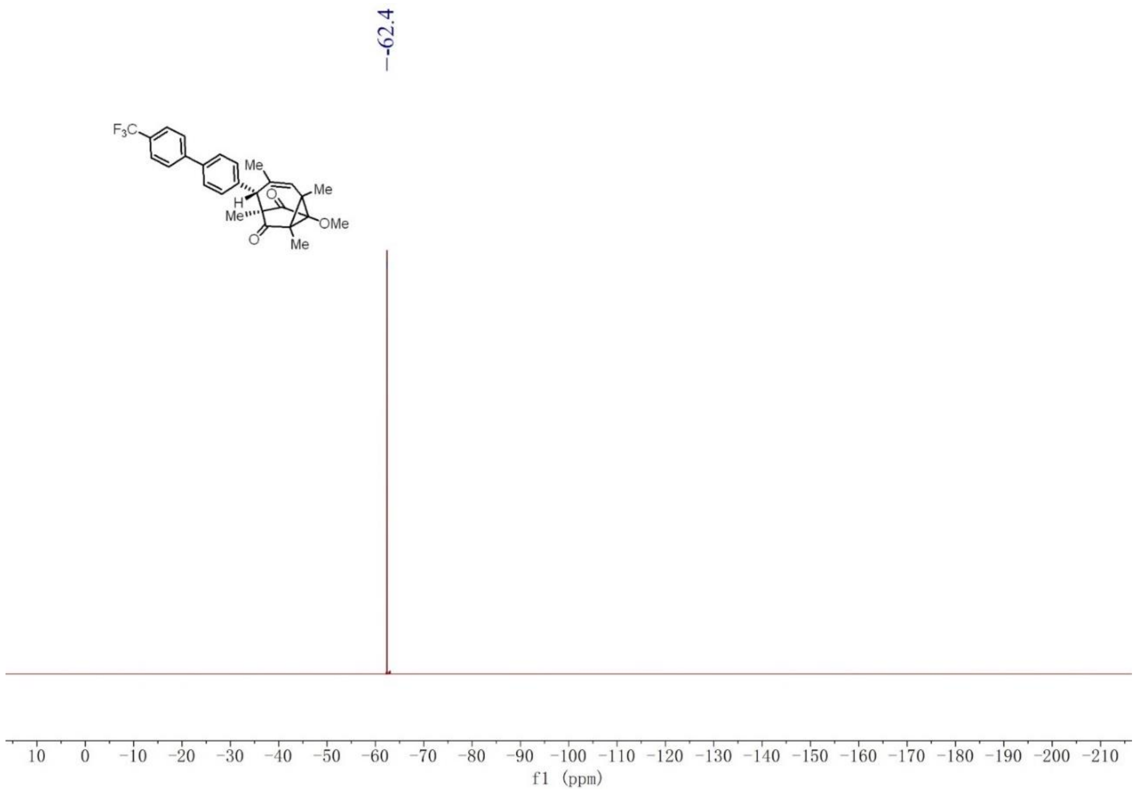


$^{19}$F NMR spectrum of compound **19a** (565MHz, $CDCl_3$)

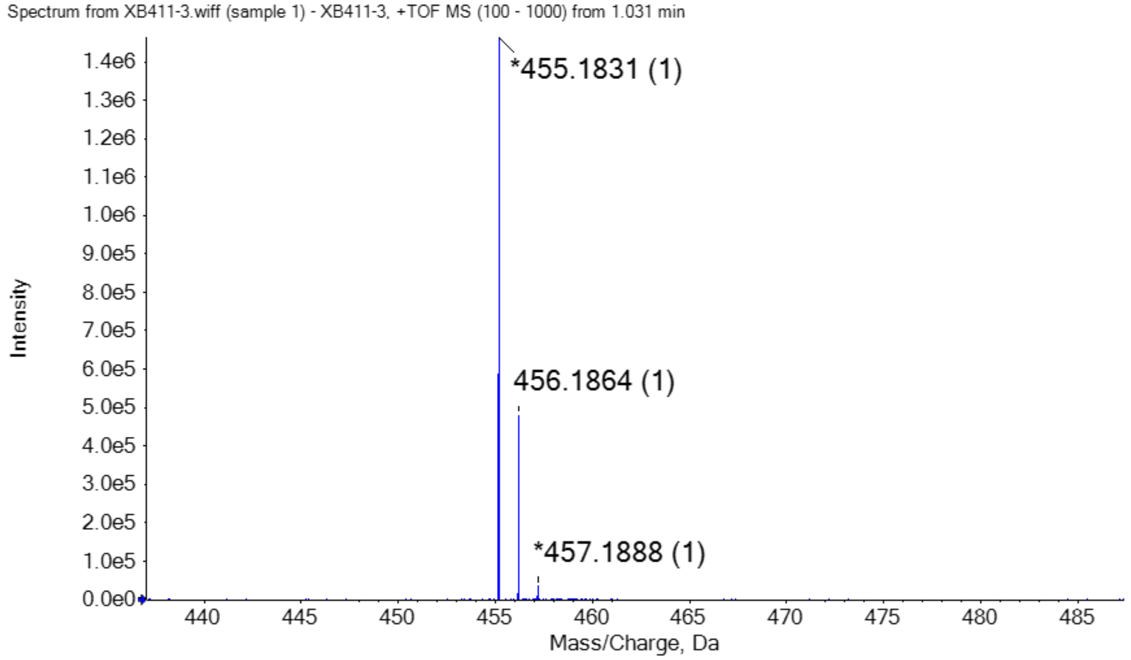


**Formula Calculator Results**

| Measured m/z | Cal m/z | Error(mmu) | Error(ppm) | Ion Formula | Ion |
|---|---|---|---|---|---|
| 455.1831 | 455.1828 | 0.2 | 0.5 | C27H26F3O3 | $[M+H]^+$ |

HRESIMS spectrum of compound **19a**

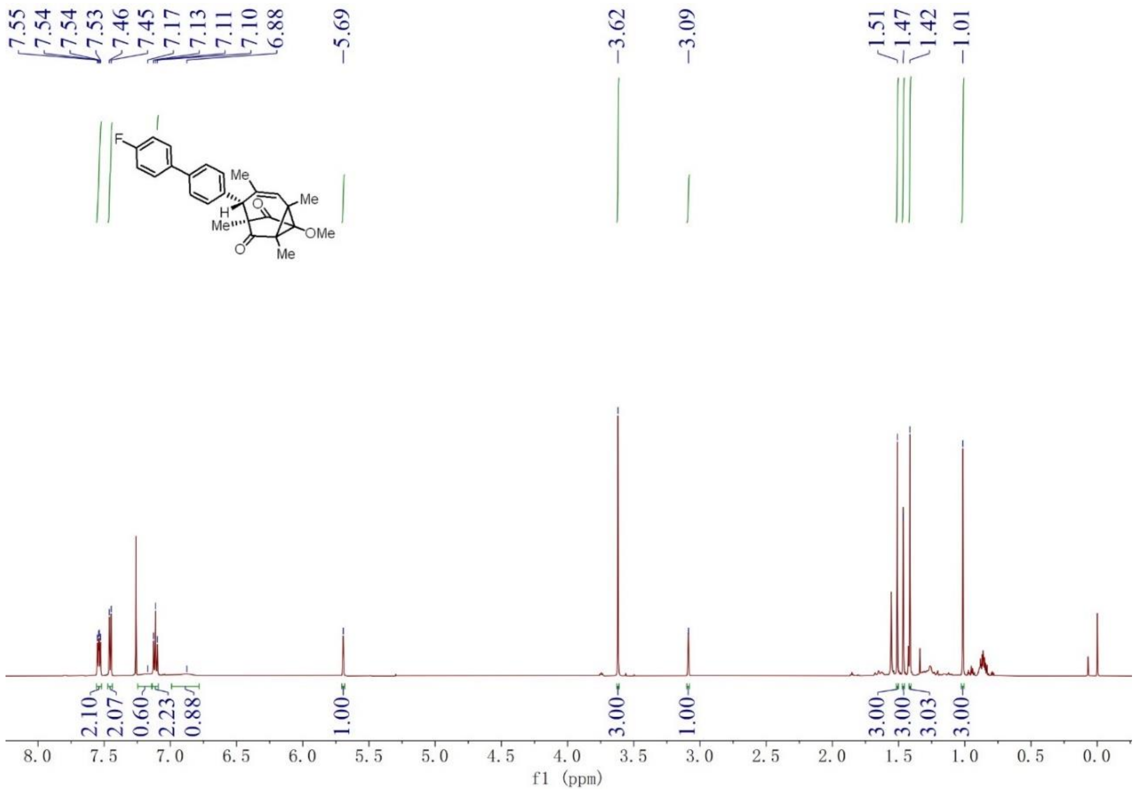


$^{1}H$ NMR spectrum of compound **19b** (600MHz, $CDCl_3$)

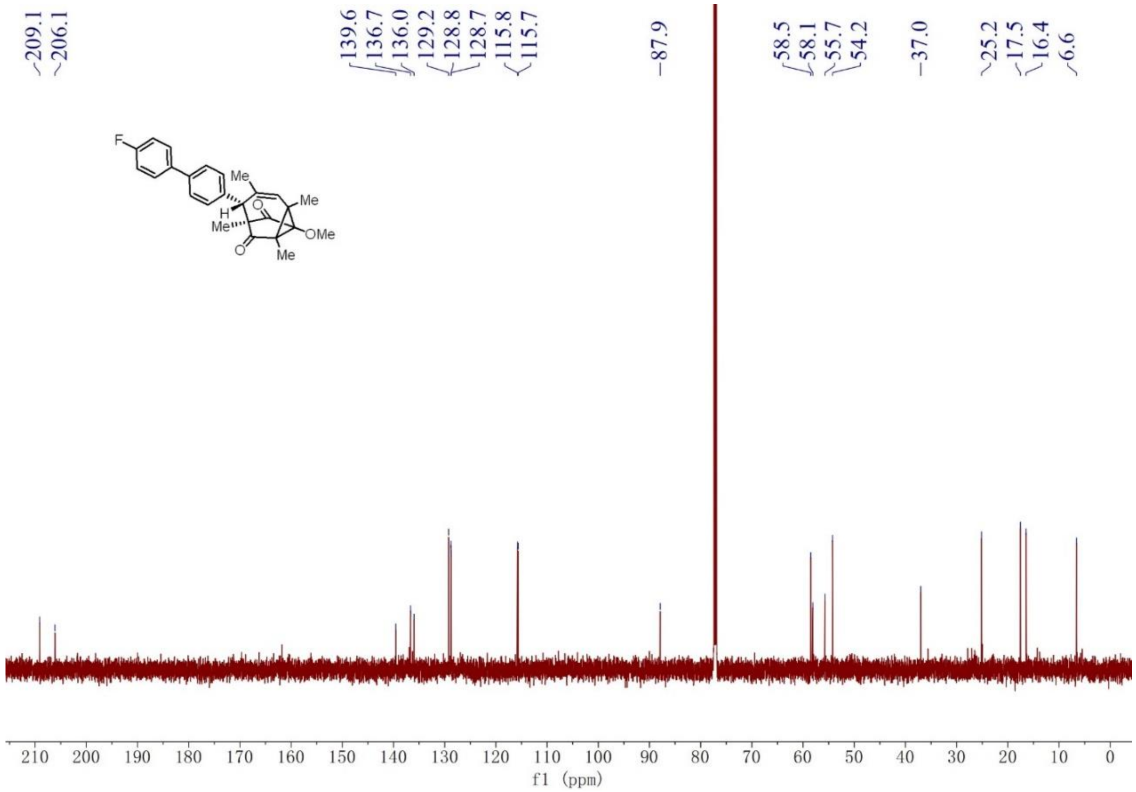


$^{13}C$ NMR spectrum of compound **19b** (151MHz, $CDCl_3$)

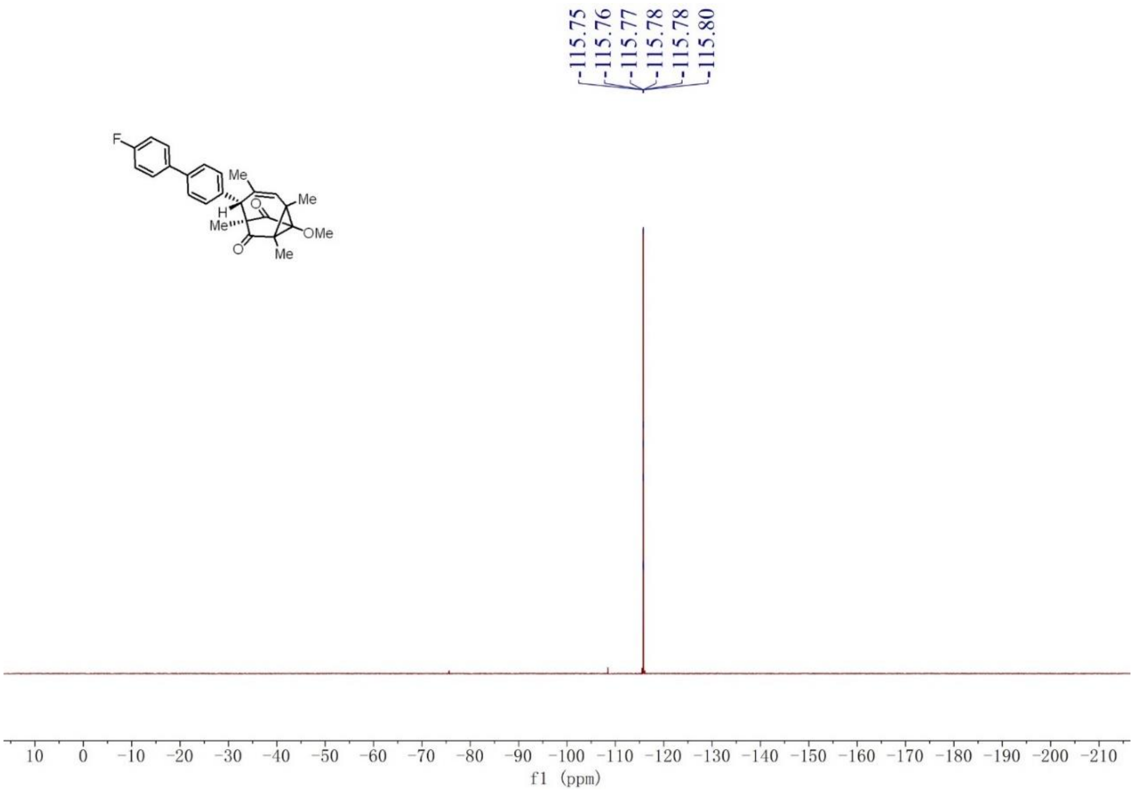


$^{19}$F NMR spectrum of compound **19b** (565MHz, $CDCl_3$)

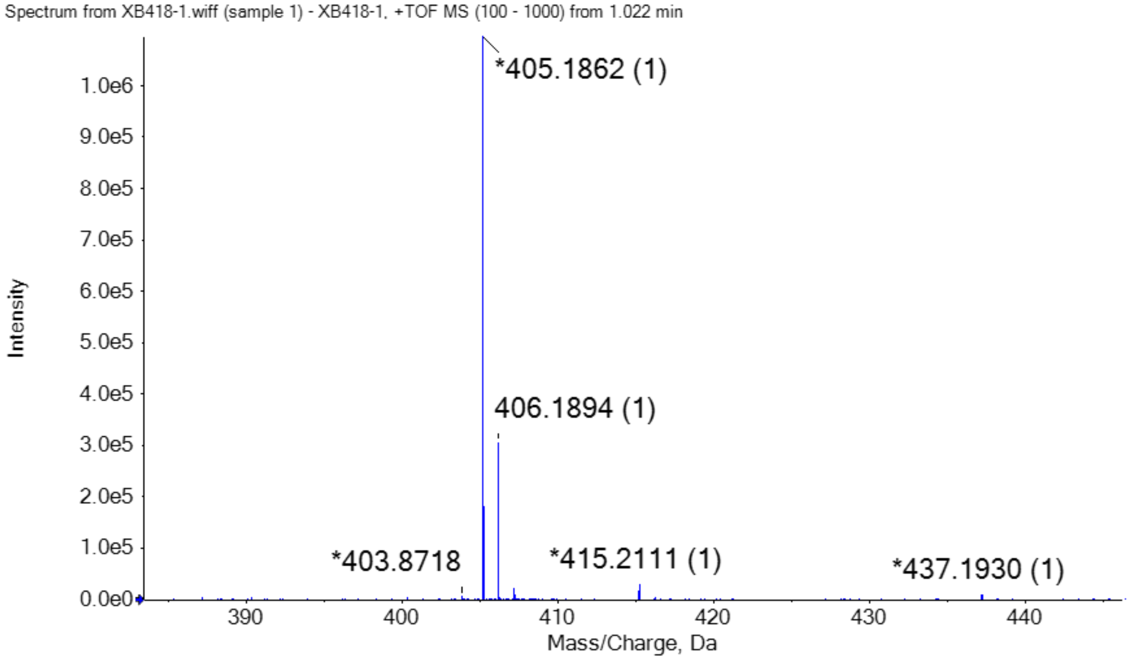


**Formula Calculator Results**

| Measured m/z | Cal m/z | Error(mmu) | Error(ppm) | Ion Formula | Ion |
|---|---|---|---|---|---|
| 405.1862 | 405.1860 | 0.1 | 0.4 | C26H26FO3 | $[M+H]^+$ |

HRESIMS spectrum of compound **19b**

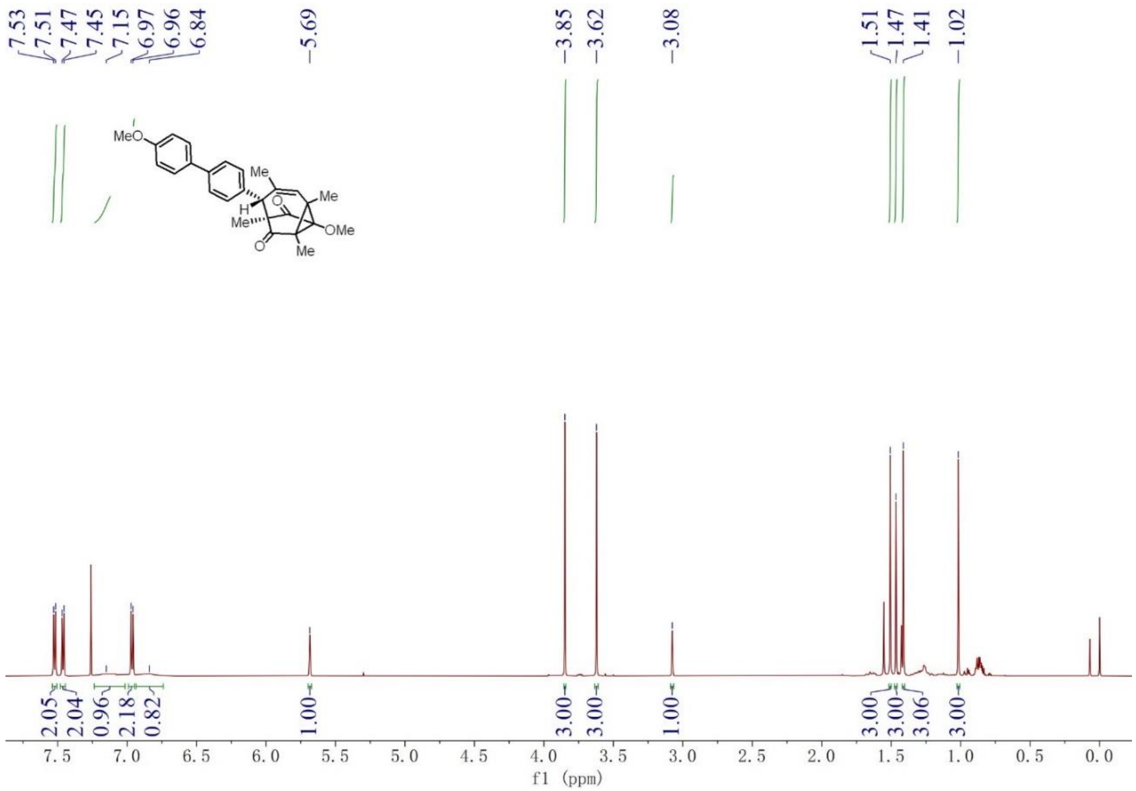

$^{1}H$ NMR spectrum of compound **19c** (600MHz, $CDCl_3$)

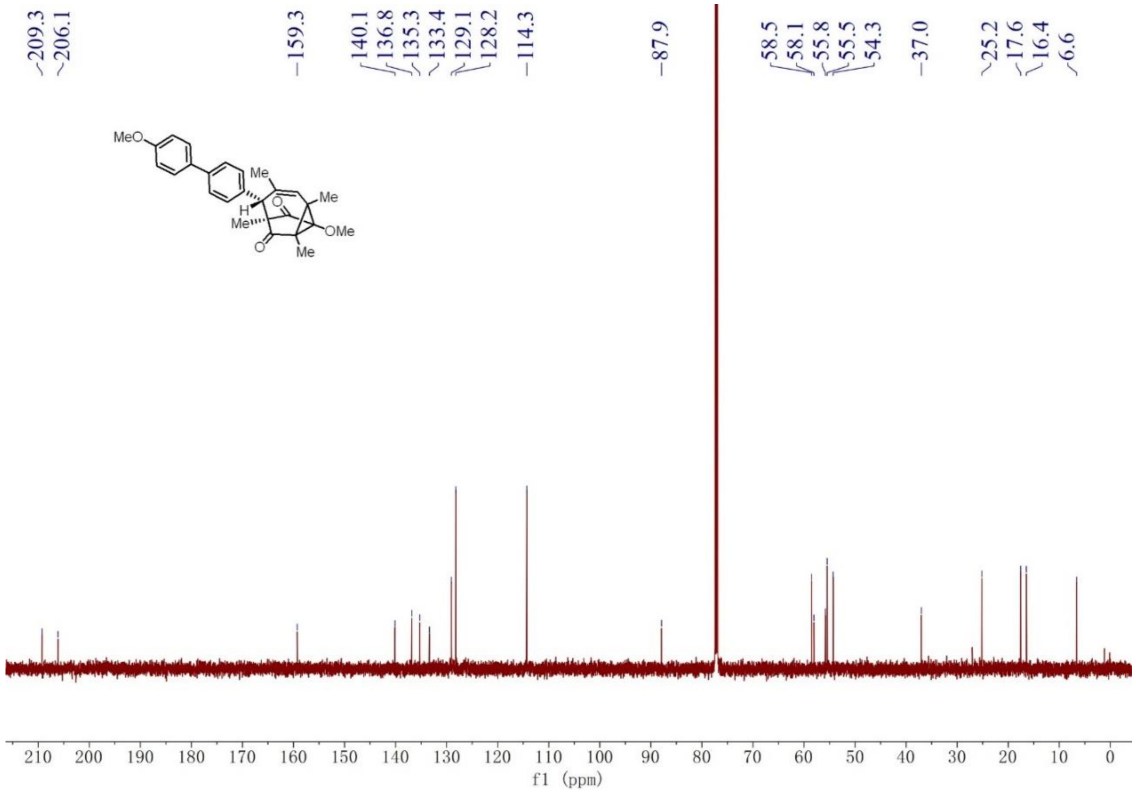

$^{13}C$ NMR spectrum of compound **19c** (151MHz, $CDCl_3$)

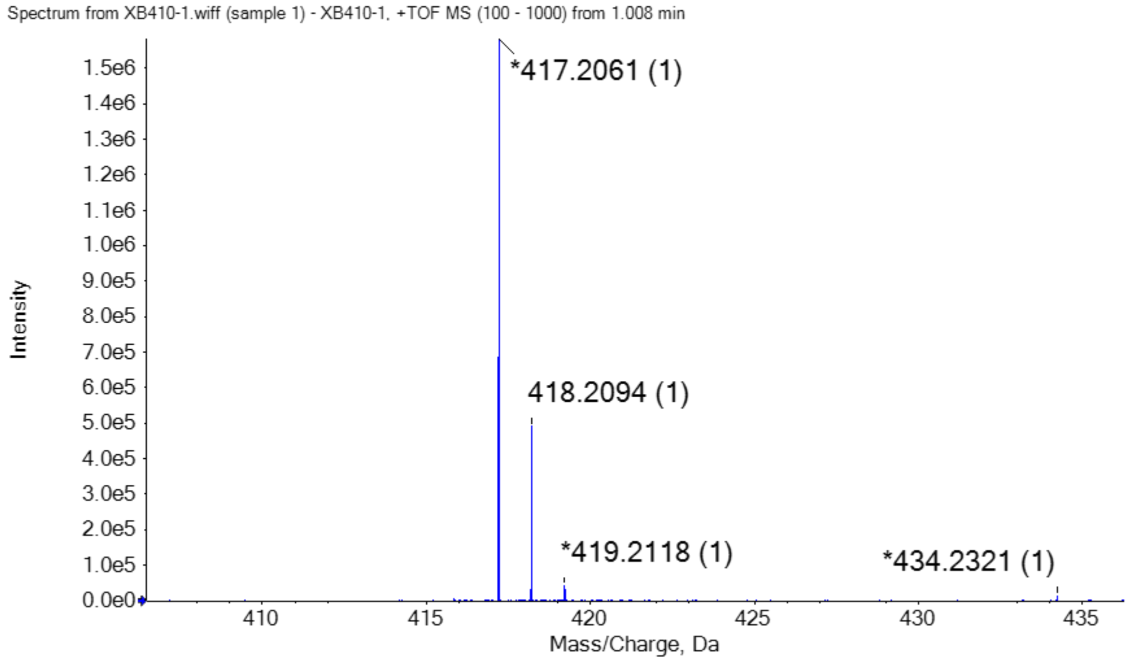


**Formula Calculator Results**

| Measured m/z | Cal m/z | Error(mmu) | Error(ppm) | Ion Formula | Ion |
|---|---|---|---|---|---|
| 417.2061 | 417.2060 | 0.1 | 0.1 | C27H29O4 | [M+H]+ |

HRESIMS spectrum of compound **19c**

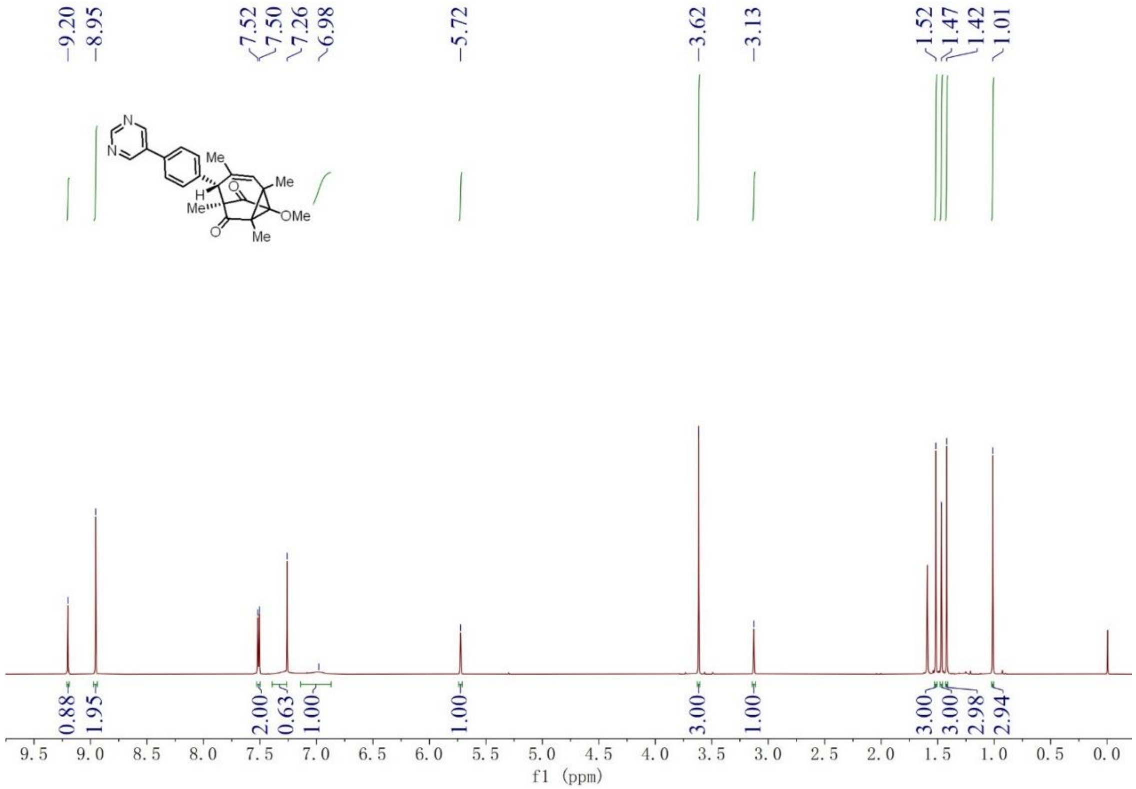


$^{1}$H NMR spectrum of compound **19d** (600MHz, $CDCl_3$)

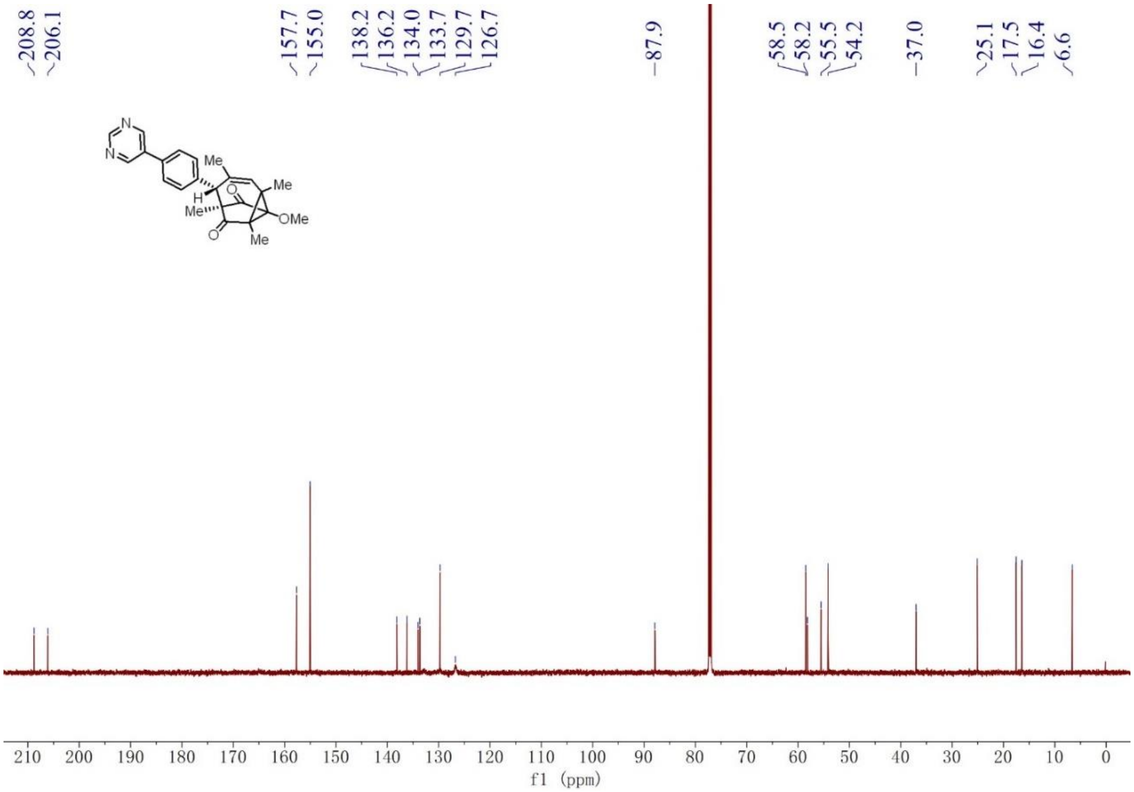


$^{13}C$ NMR spectrum of compound **19d** (151MHz, $CDCl_3$)

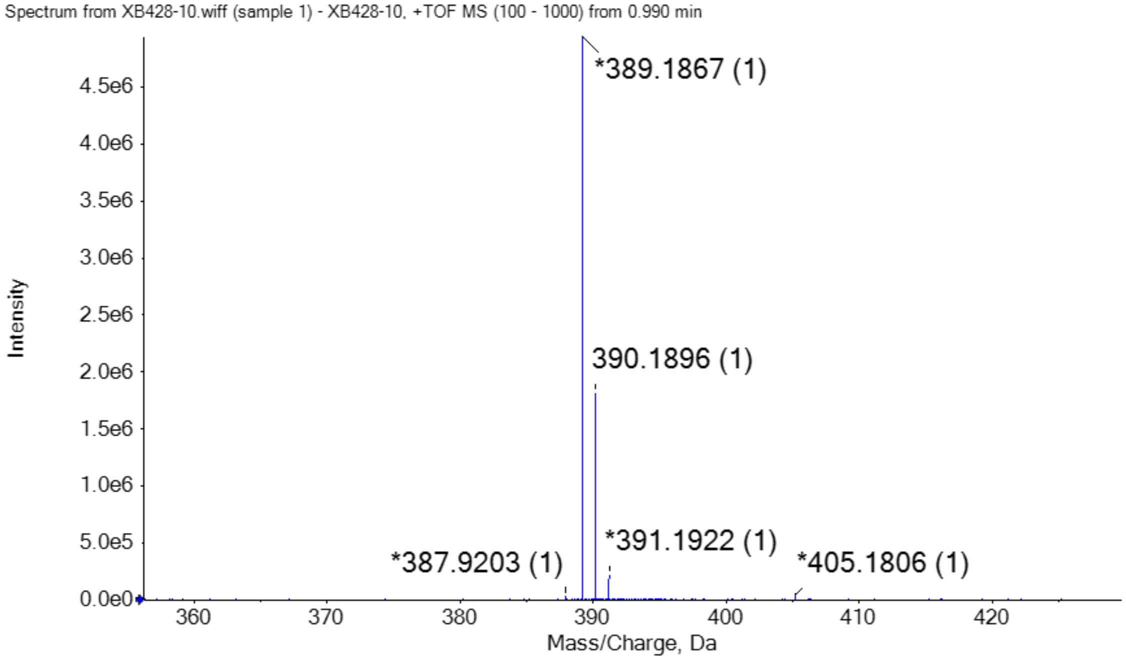


**Formula Calculator Results**

| Measured m/z | Cal m/z | Error(mmu) | Error(ppm) | Ion Formula | Ion |
|---|---|---|---|---|---|
| 389.1867 | 389.1859 | 0.7 | 1.9 | C24H25N2O3 | $[M+H]^+$ |

HRESIMS spectrum of compound **19d**

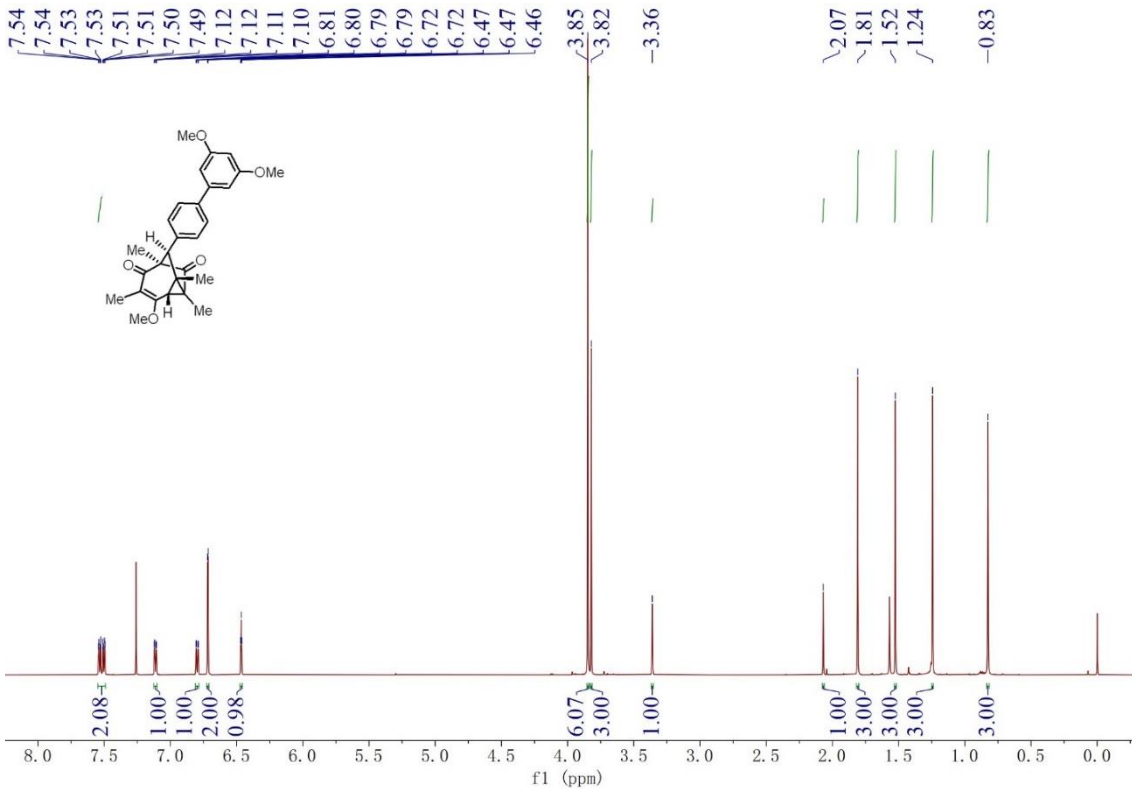

$^{1}$H NMR spectrum of compound **20** (600MHz, $CDCl_3$)

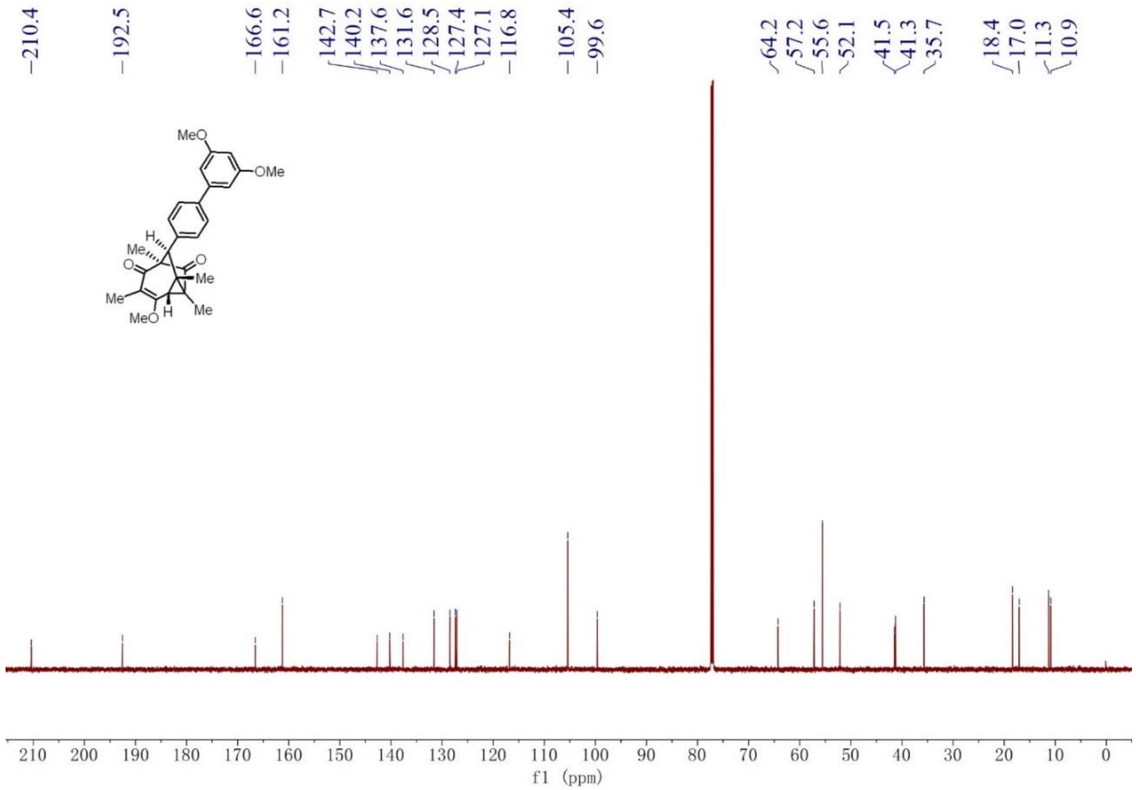

$^{13}$C NMR spectrum of compound **20** (151MHz, $CDCl_3$)

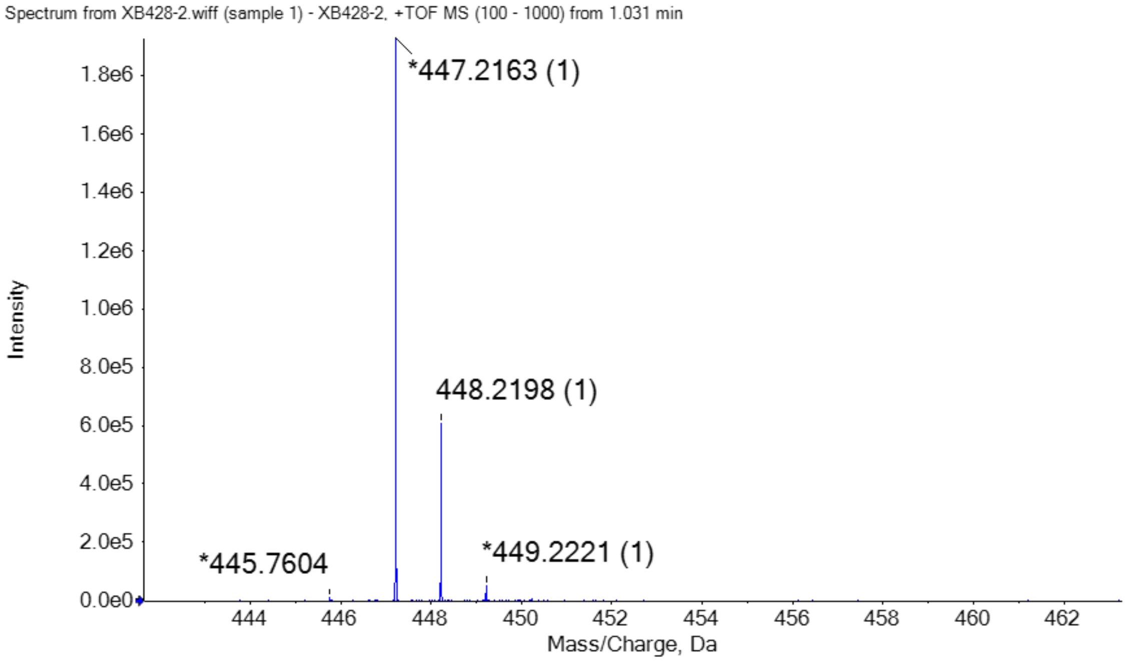


**Formula Calculator Results**

| Measured m/z | Cal m/z | Error(mmu) | Error(ppm) | Ion Formula | Ion |
|---|---|---|---|---|---|
| 447.2163 | 447.2166 | -0.3 | -0.7 | C28H31O5 | $[M+H]^+$ |

HRESIMS spectrum of compound **20**

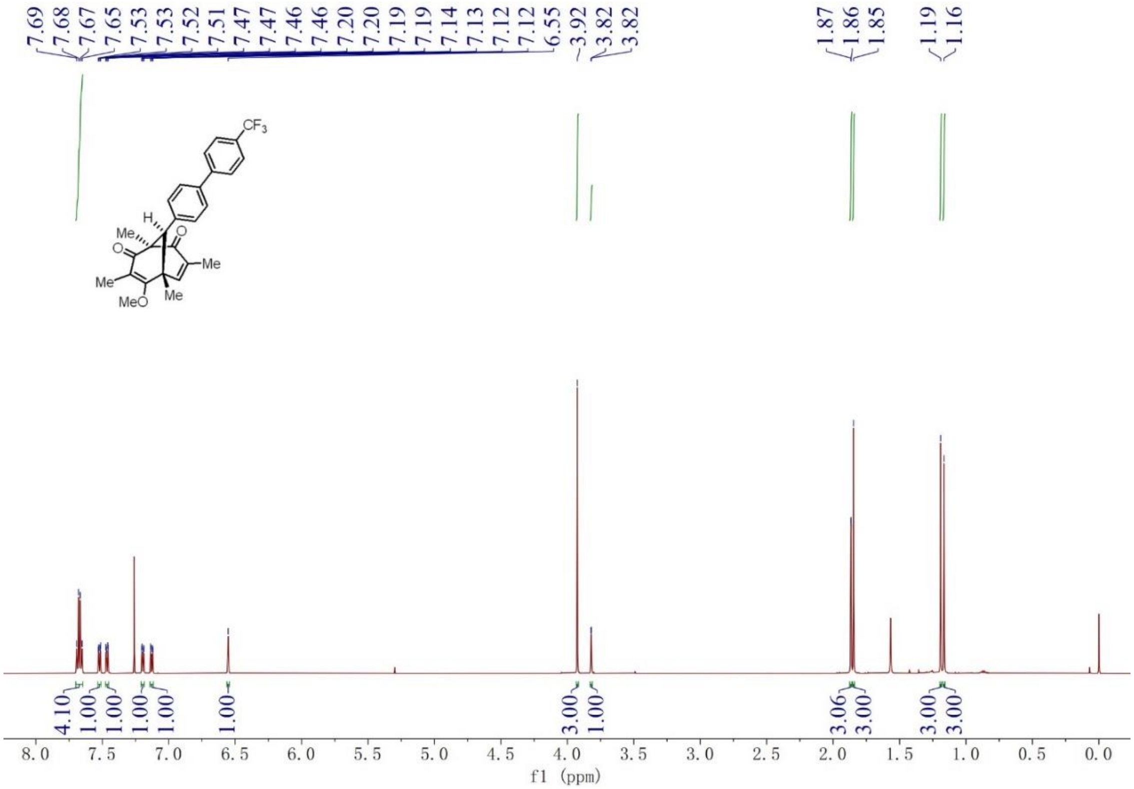


$^1$H NMR spectrum of compound **21a** (600MHz, $CDCl_3$)

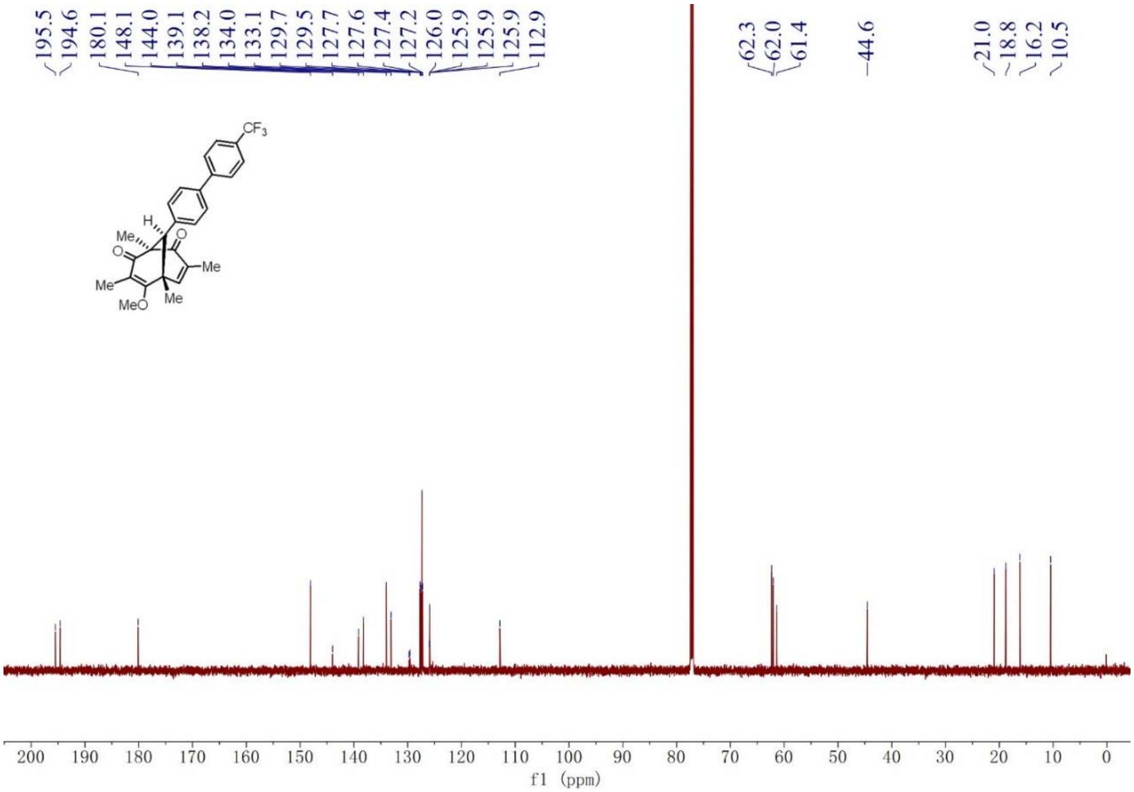


$^{13}$C NMR spectrum of compound **21a** (151MHz, $CDCl_3$)

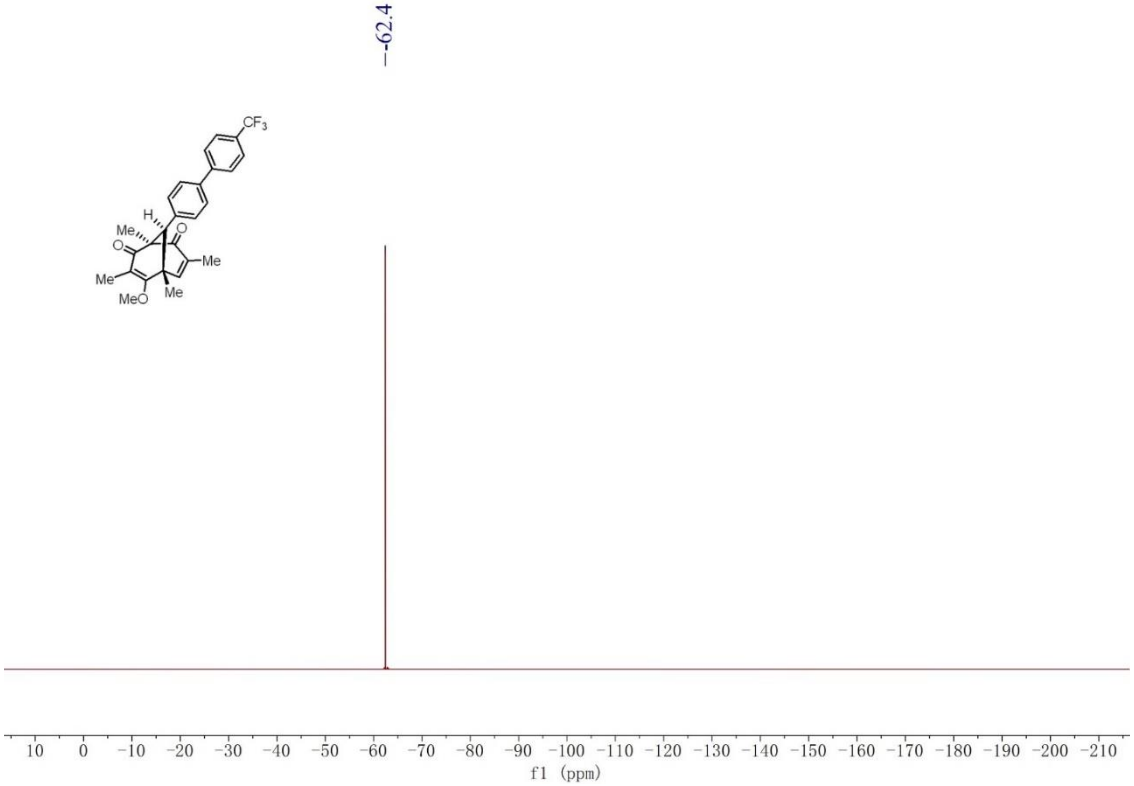


$^{19}$F NMR spectrum of compound **21a** (565MHz, $CDCl_3$)

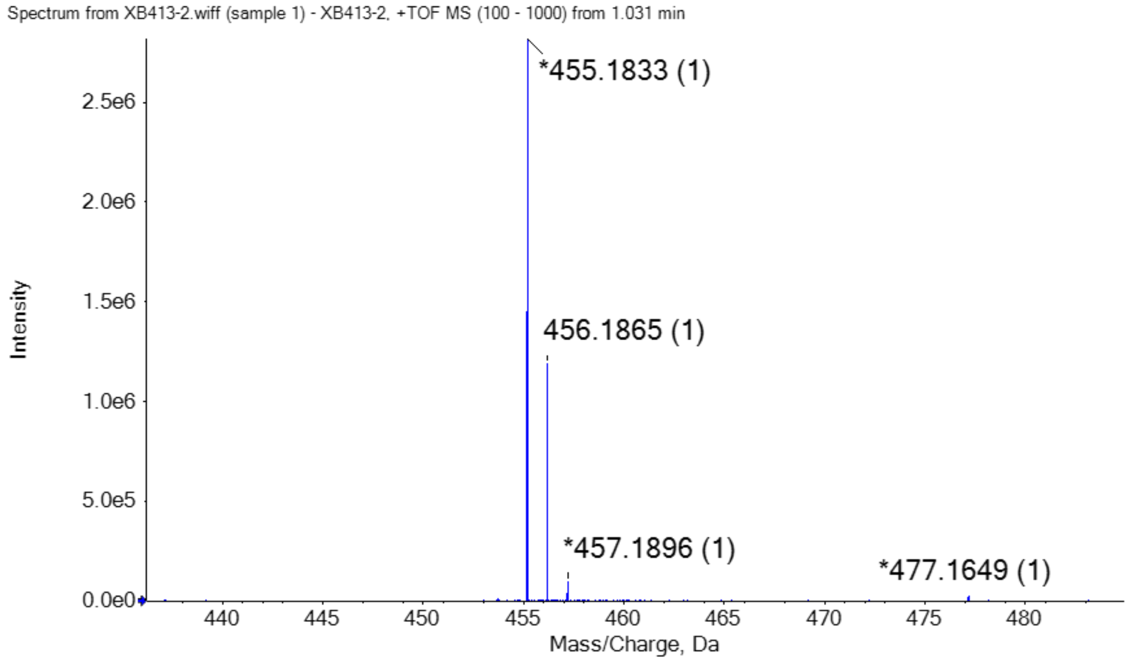


**Formula Calculator Results**

| Measured m/z | Cal m/z | Error(mmu) | Error(ppm) | Ion Formula | Ion |
|---|---|---|---|---|---|
| 455.1833 | 455.1828 | 0.4 | 1.0 | C27H26F3O3 | $[M+H]^+$ |

HRESIMS spectrum of compound **21a**

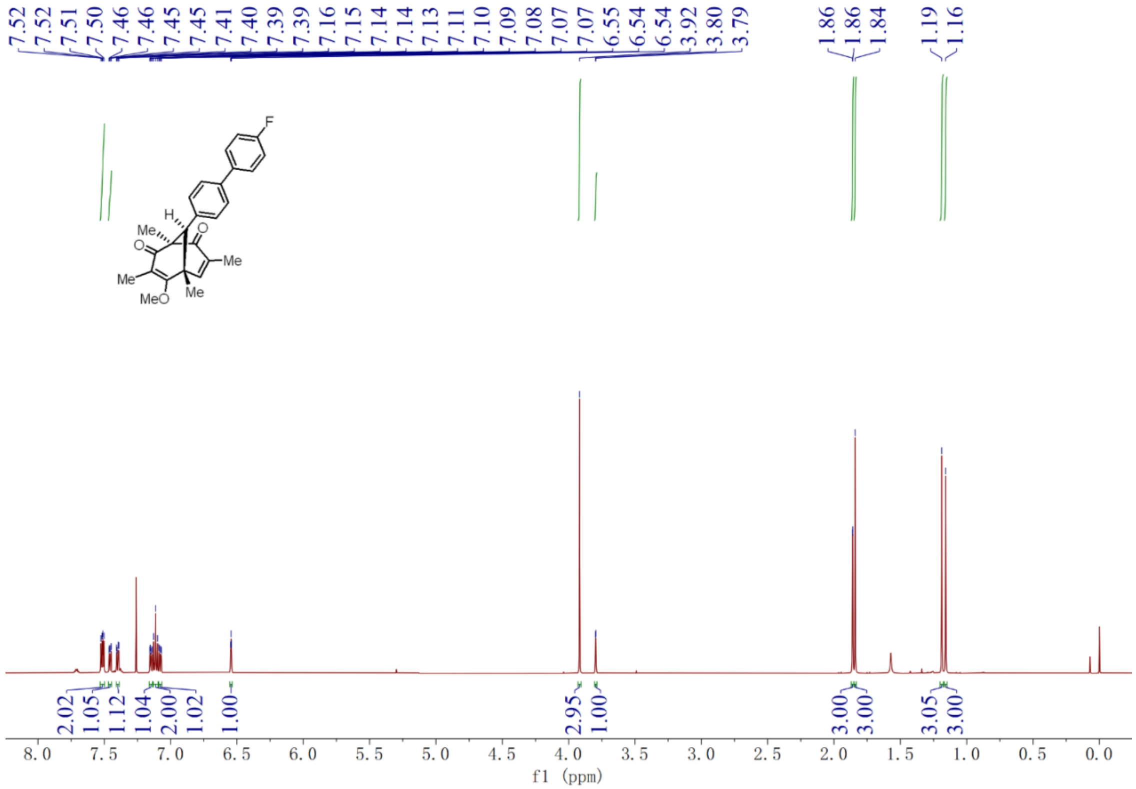


$^1$H NMR spectrum of compound **21b** (600MHz, $CDCl_3$)

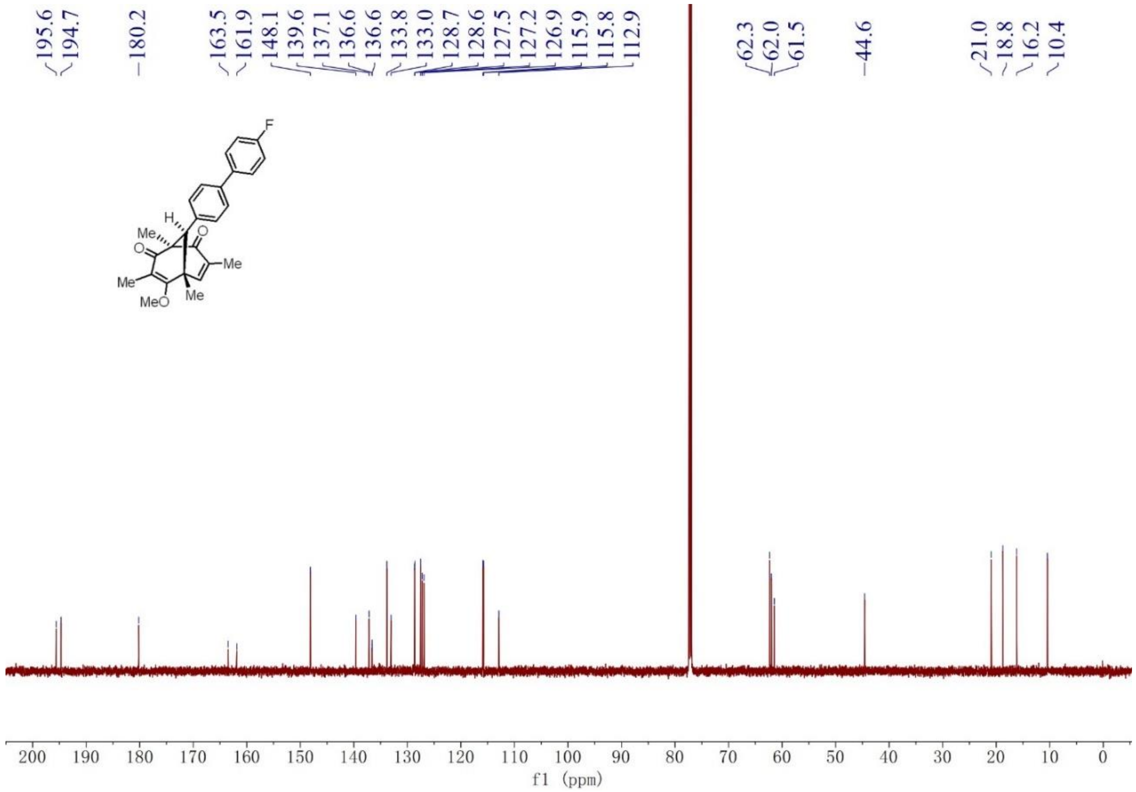


$^{13}$C NMR spectrum of compound **21b** (151MHz, $CDCl_3$)

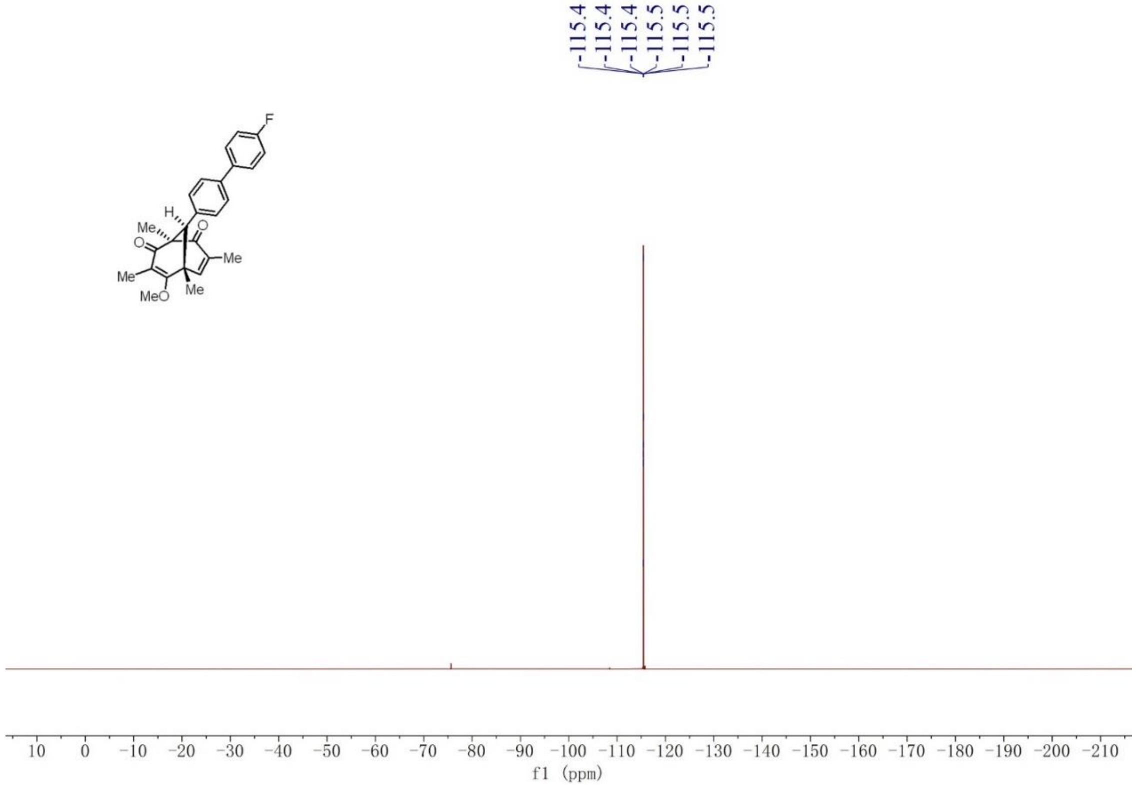


$^{19}$F NMR spectrum of compound **21b** (565MHz, $CDCl_3$)

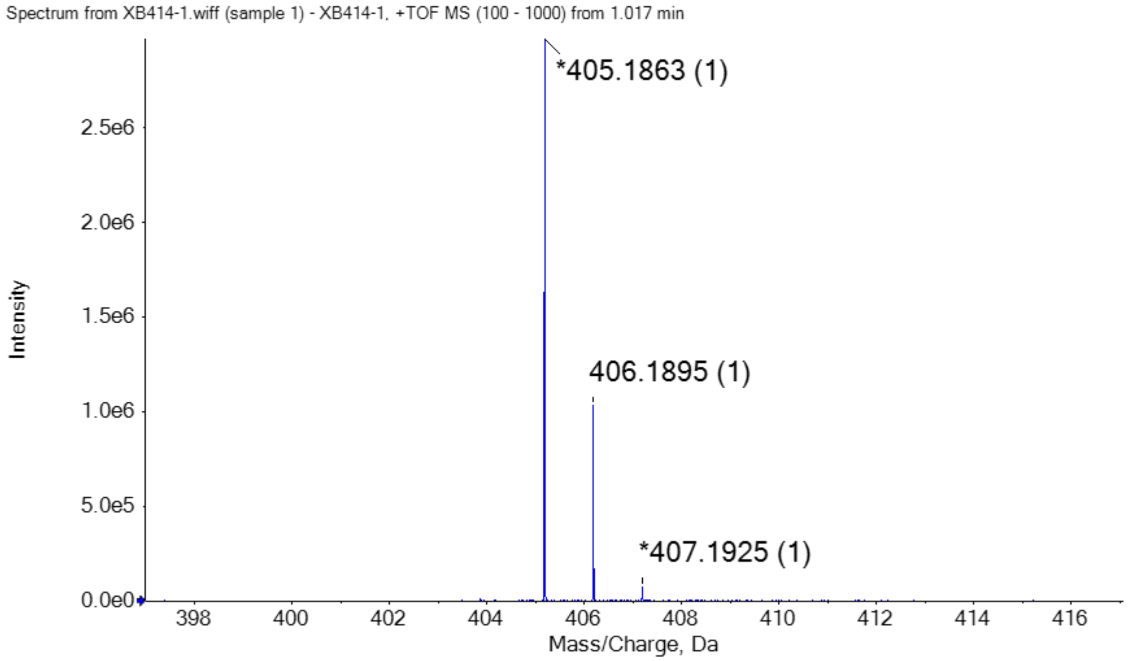


**Formula Calculator Results**

| Measured m/z | Cal m/z | Error(mmu) | Error(ppm) | Ion Formula | Ion |
|---|---|---|---|---|---|
| 405.1863 | 405.1860 | 0.2 | 0.6 | C26H26FO3 | $[M+H]^+$ |

HRESIMS spectrum of compound **21b**

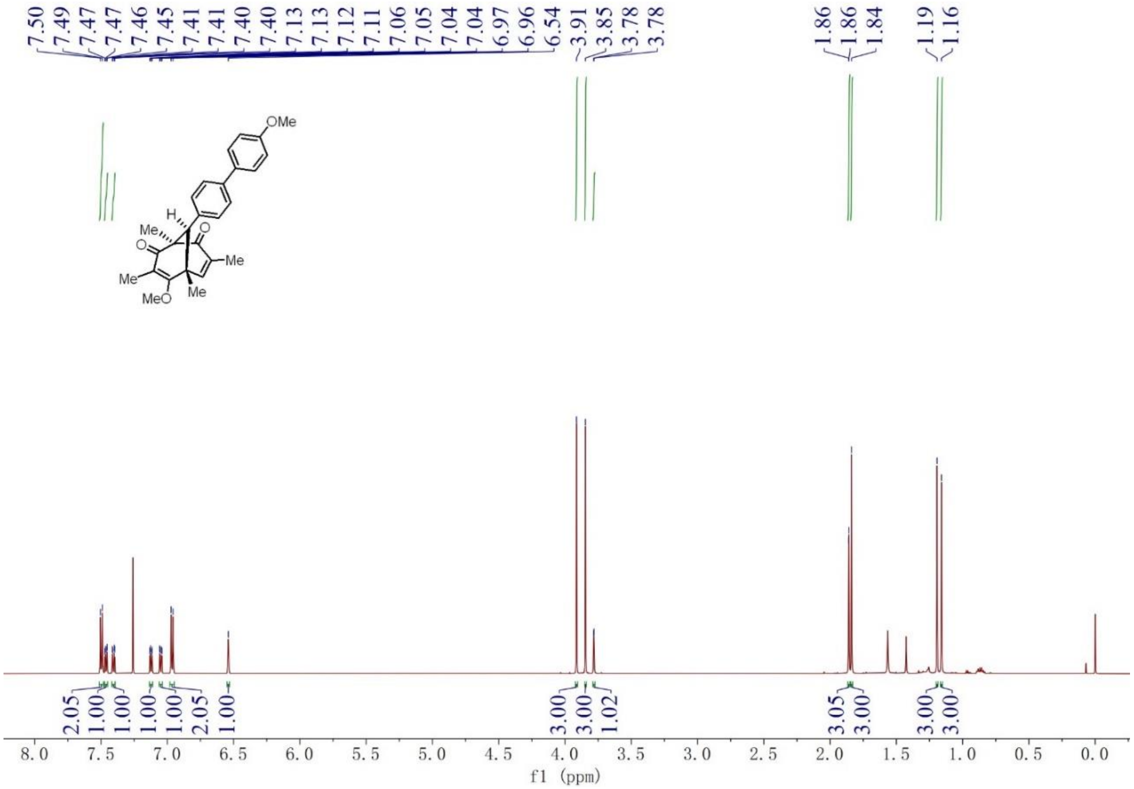


$^{1}$H NMR spectrum of compound **21c** (600MHz, $CDCl_3$)

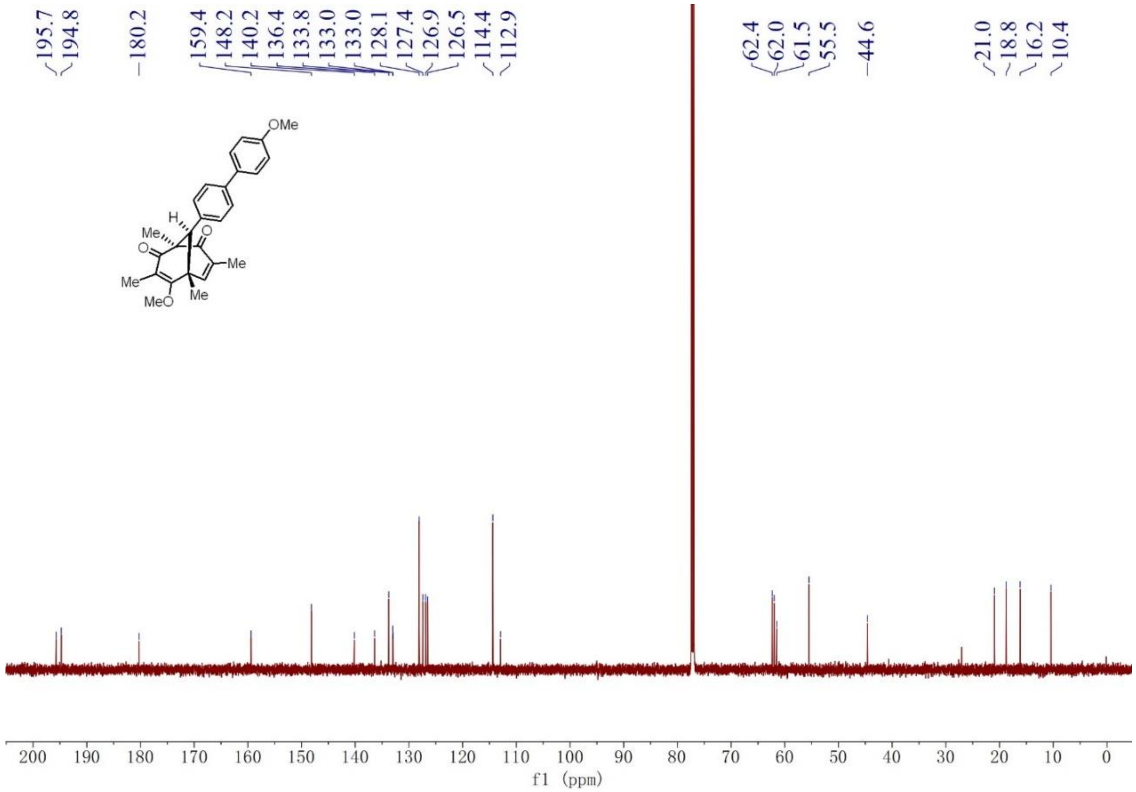


$^{13}$C NMR spectrum of compound **21c** (151MHz, $CDCl_3$)

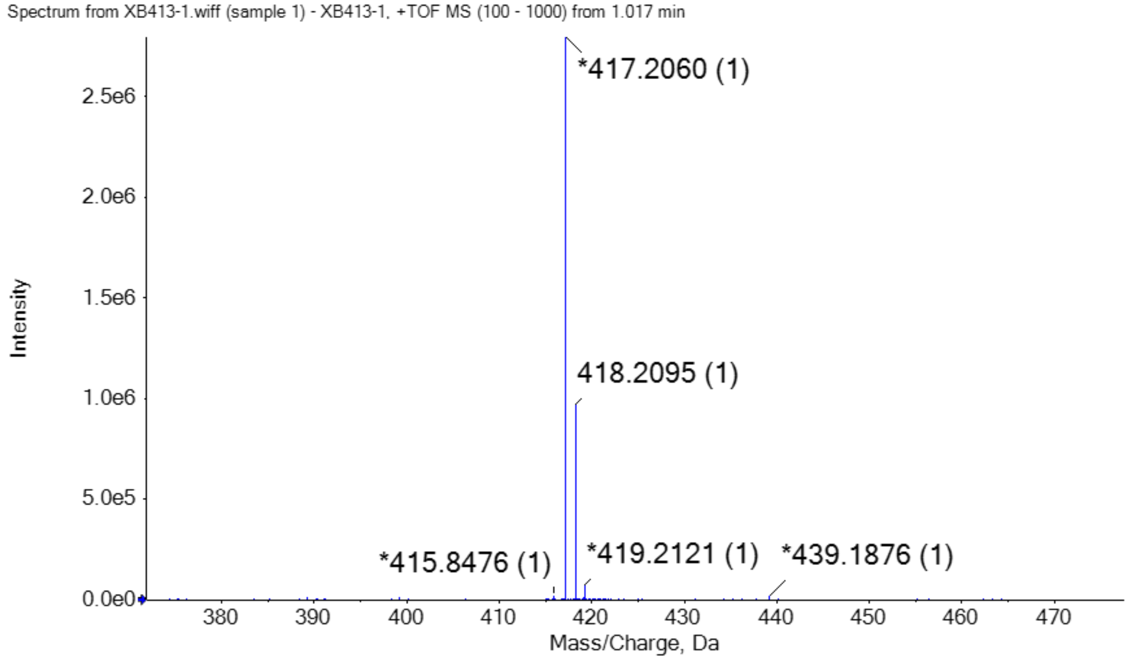


**Formula Calculator Results**

| Measured m/z | Cal m/z | Error(mmu) | Error(ppm) | Ion Formula | Ion |
|---|---|---|---|---|---|
| 417.2060 | 417.2060 | 0.0 | -0.1 | C27H29O4 | $[M+H]^+$ |

HRESIMS spectrum of compound **21c**

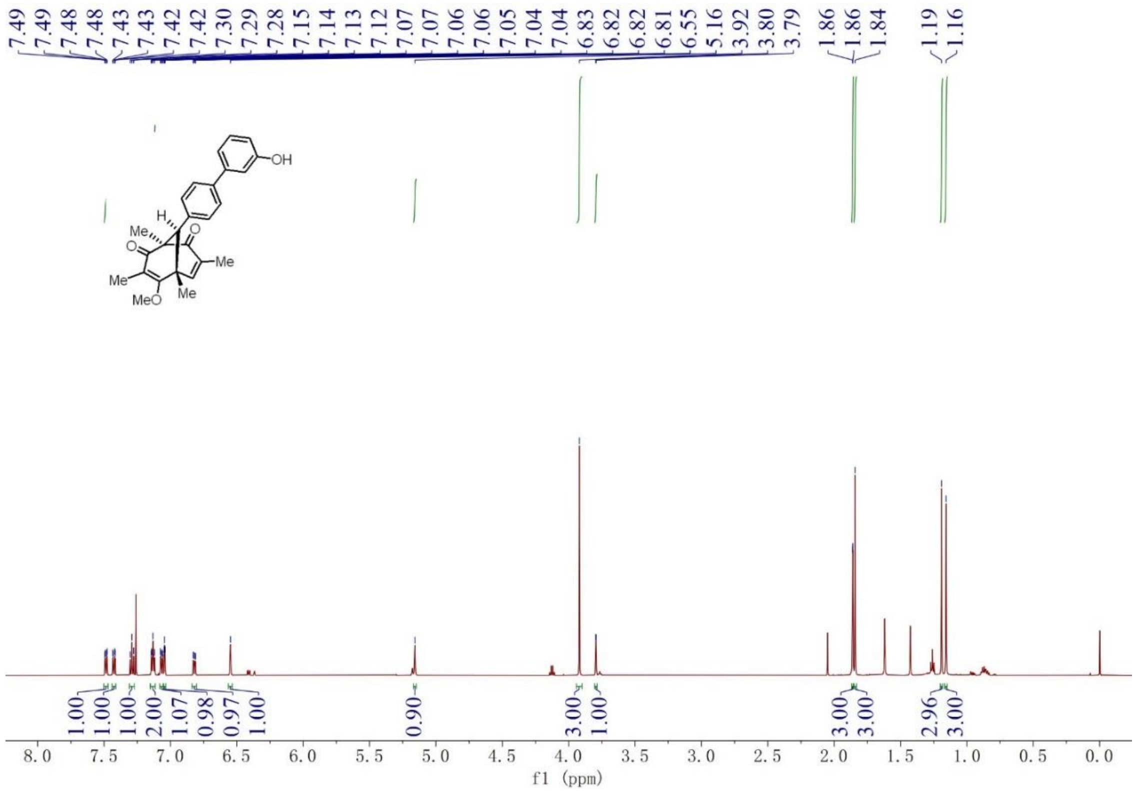


$^{1}H$ NMR spectrum of compound **21d** (600MHz, $CDCl_3$)

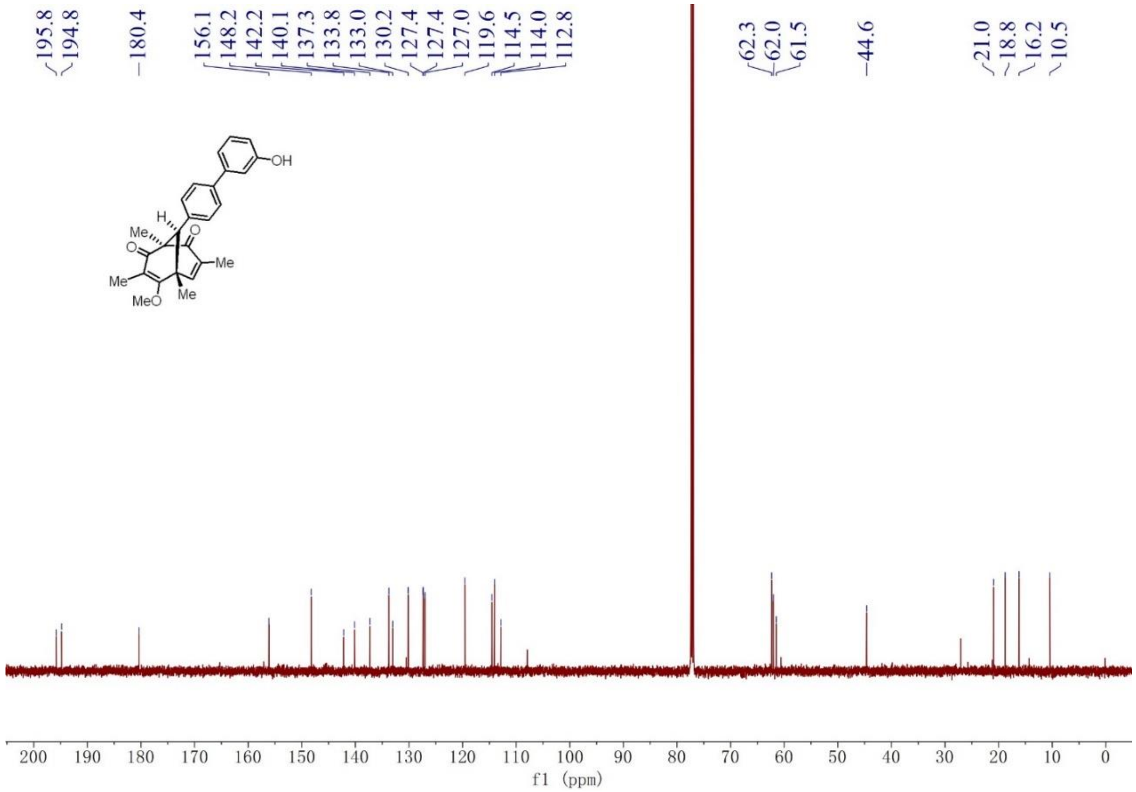


$^{13}C$ NMR spectrum of compound **21d** (151MHz, $CDCl_3$)

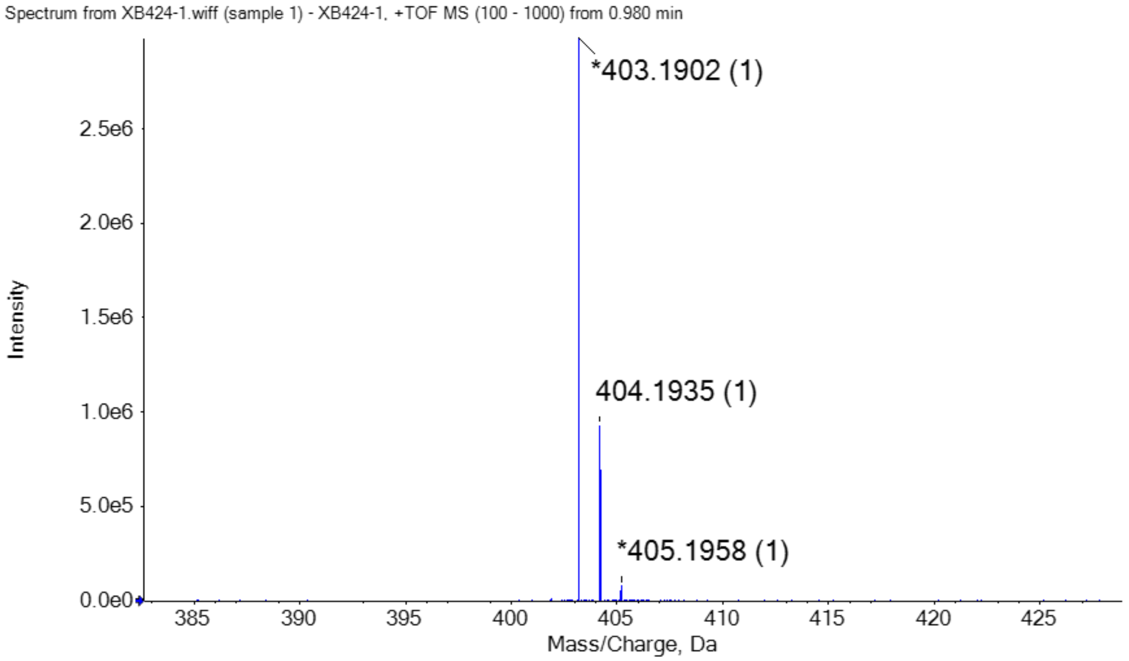


**Formula Calculator Results**

| Measured m/z | Cal m/z | Error(mmu) | Error(ppm) | Ion Formula | Ion |
|---|---|---|---|---|---|
| 403.1902 | 403.19039 | -0.2 | -0.5 | C26H27O4 | $[M+H]^+$ |

HRESIMS spectrum of compound **21d**

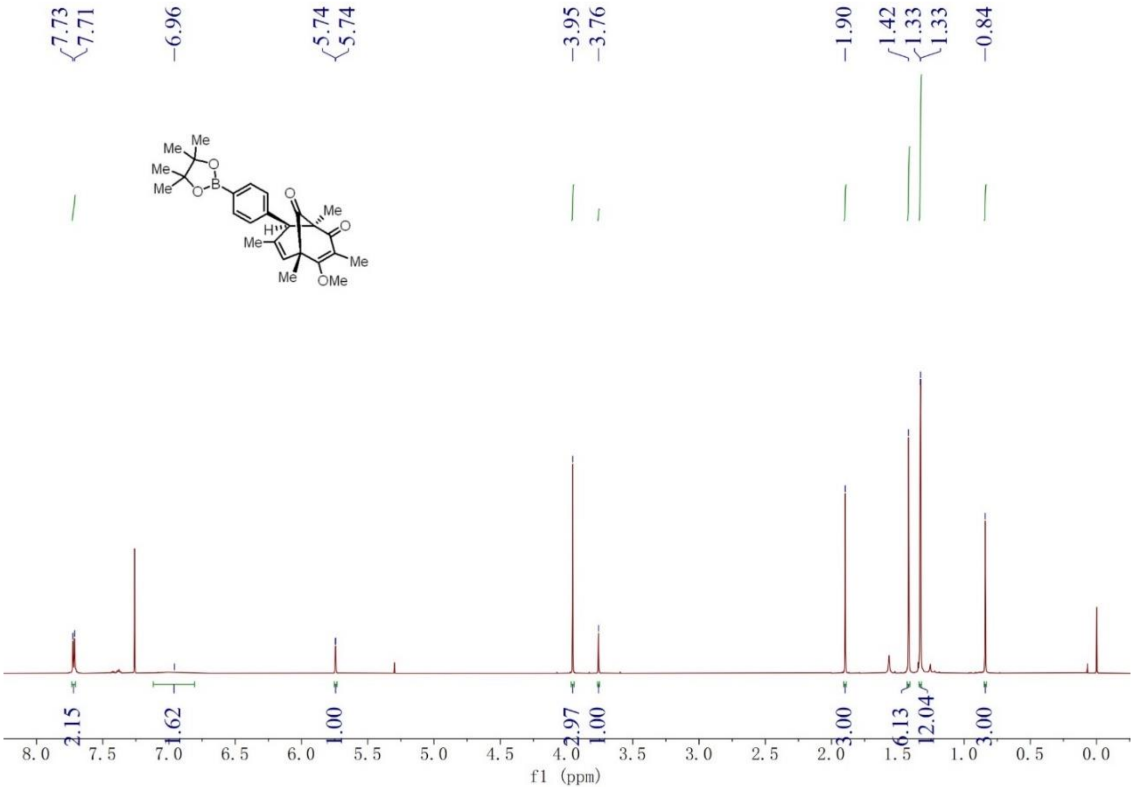


$^1$H NMR spectrum of compound **17e** (600MHz, $CDCl_3$)

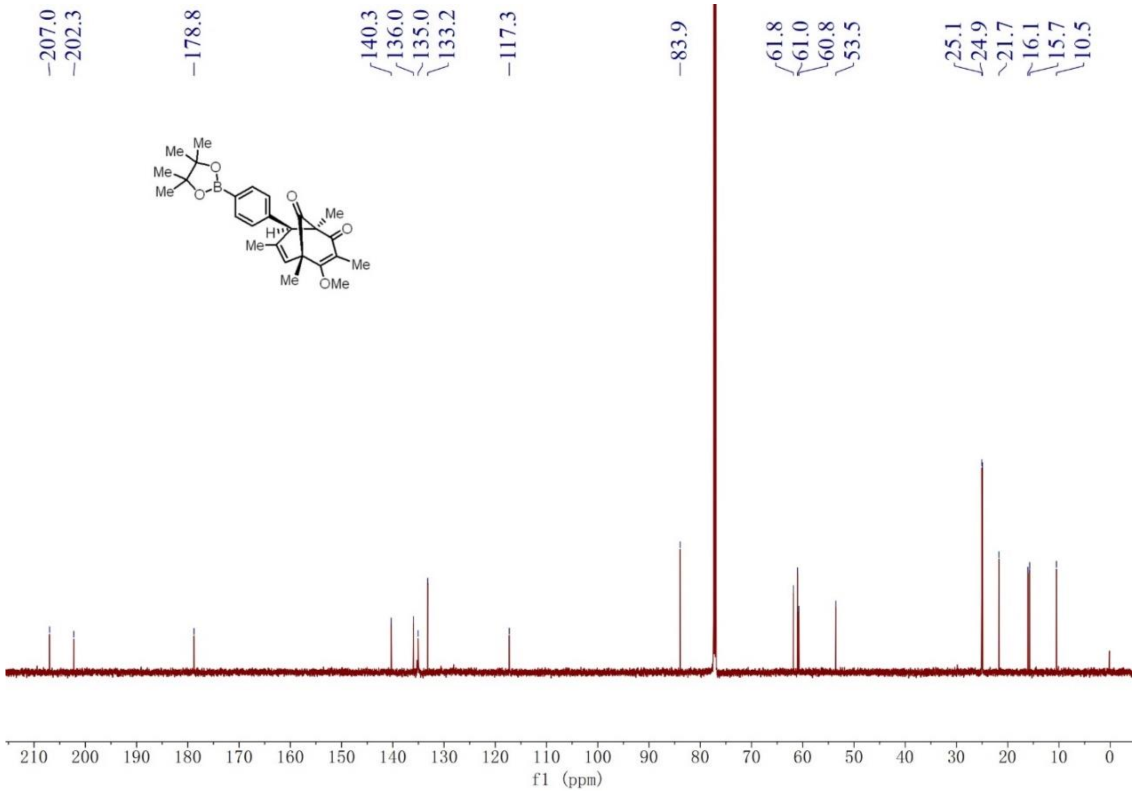


$^{13}$C NMR spectrum of compound **17e** (151MHz, $CDCl_3$)

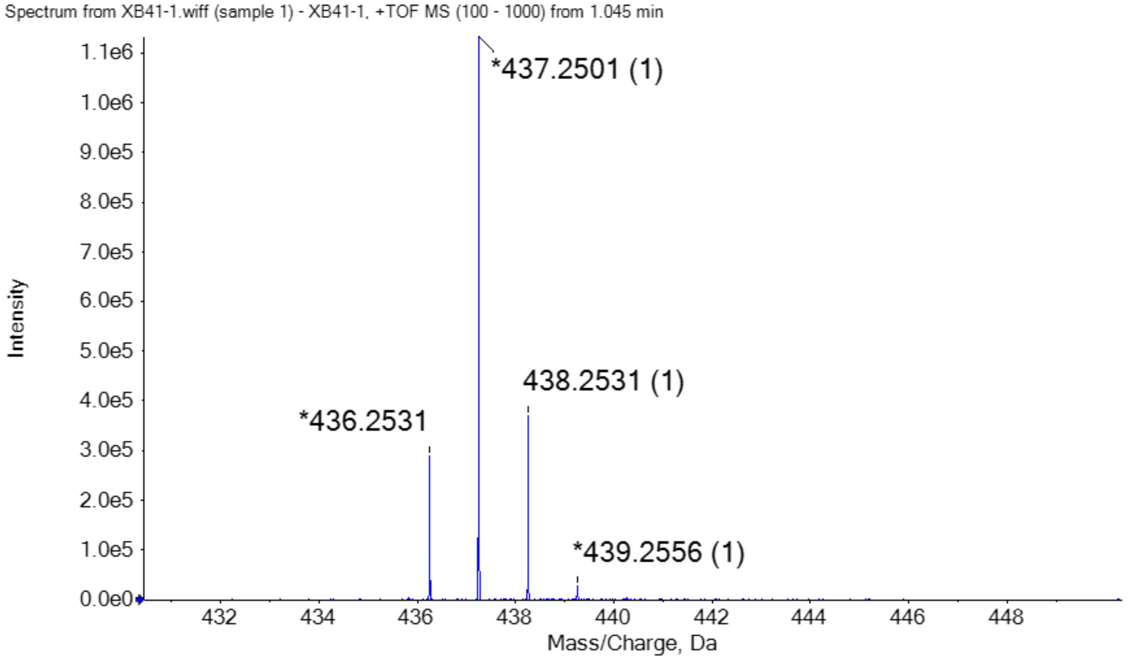


**Formula Calculator Results**

| Measured m/z | Cal m/z | Error(mmu) | Error(ppm) | Ion Formula | Ion |
|---|---|---|---|---|---|
| 437.2501 | 437.2493 | 0.7 | 1.6 | C26H34BO5 | $[M+H]^+$ |

HRESIMS spectrum of compound **17e**

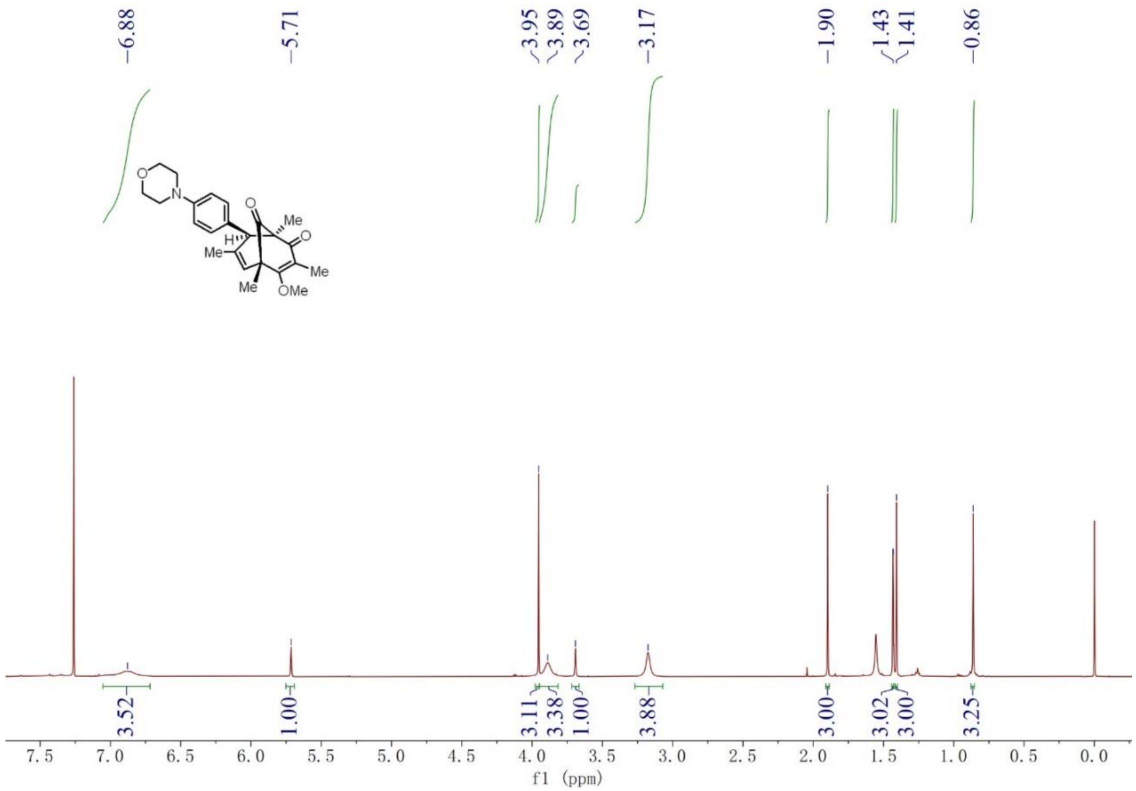


$^{1}H$ NMR spectrum of compound **17f** (600MHz, $CDCl_3$)

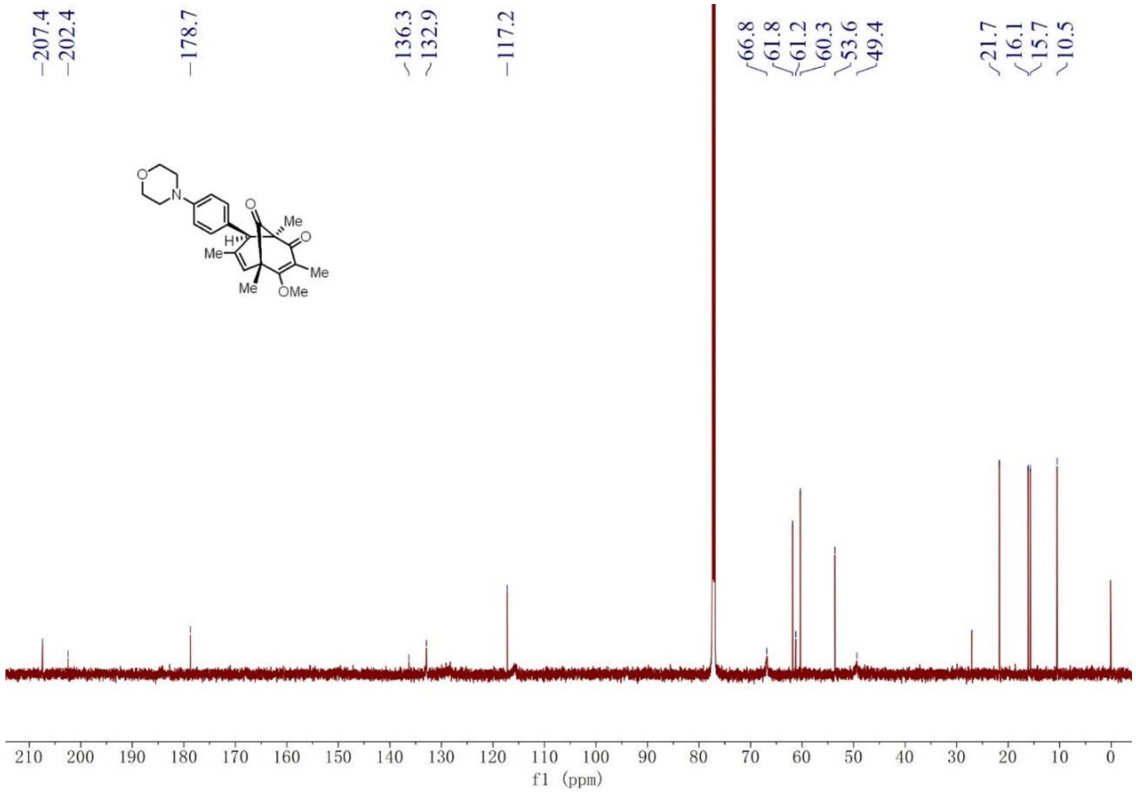


$^{13}C$ NMR spectrum of compound **17f** (151MHz, $CDCl_3$)

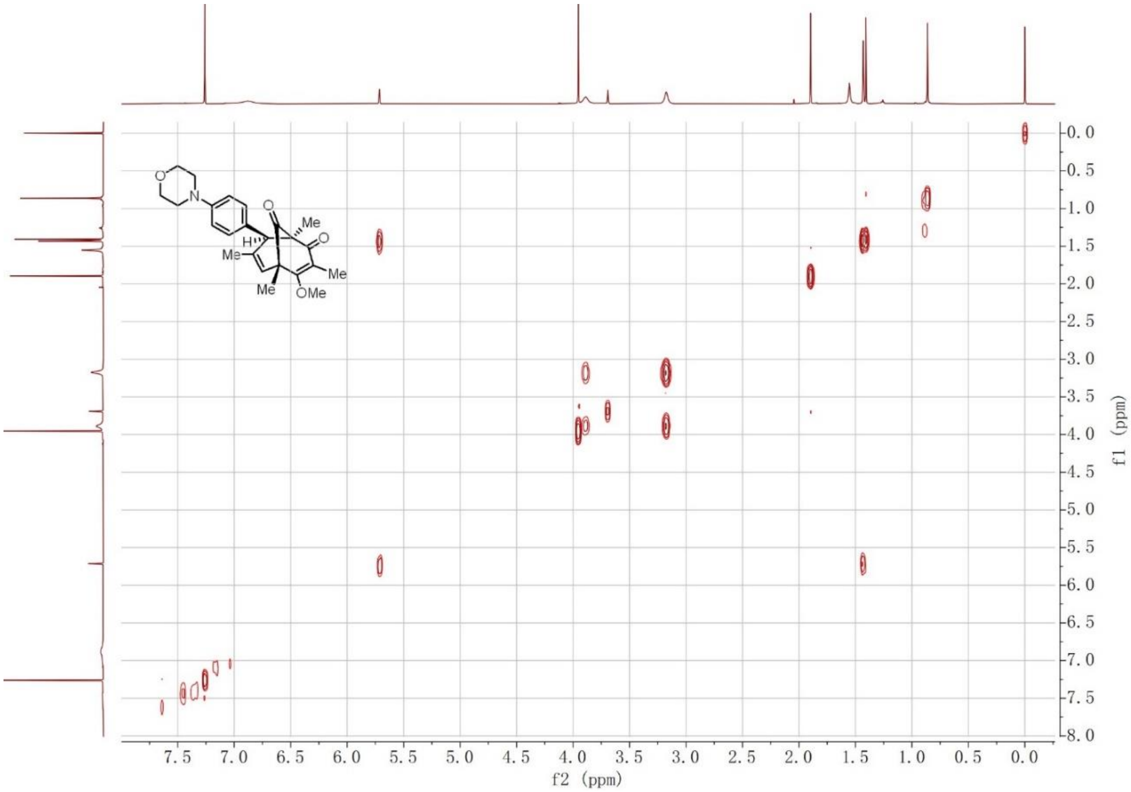


$^{1}H$-$^{1}H$ COSY spectrum of compound **17f** (600 MHz, $CDCl_3$)

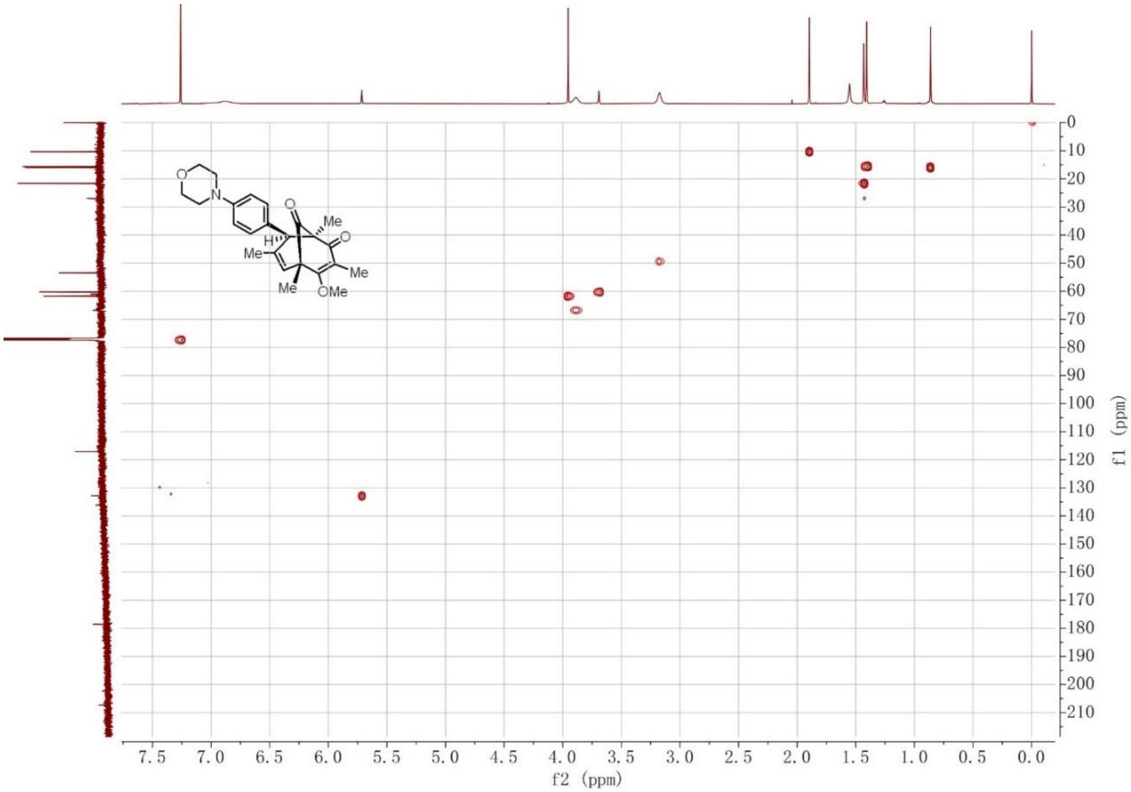


HSQC spectrum of compound **17f** (600 MHz, $CDCl_3$)

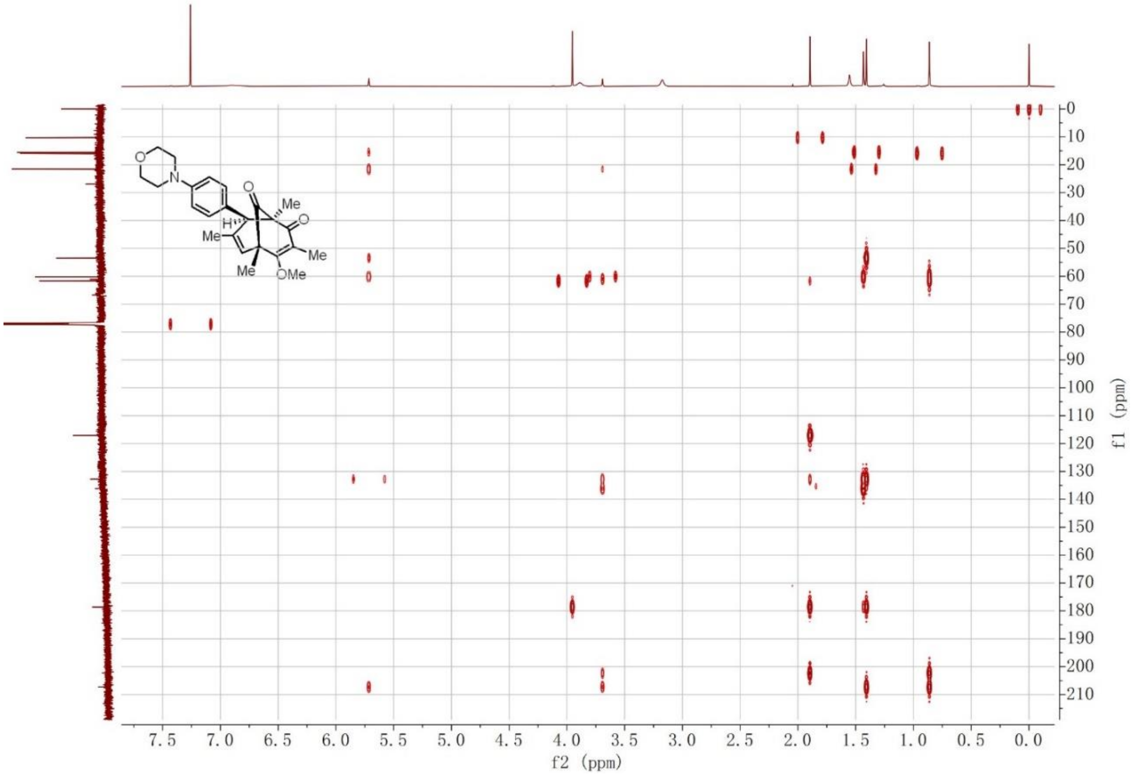

HMBC spectrum of compound **17f** (600 MHz, $CDCl_3$)

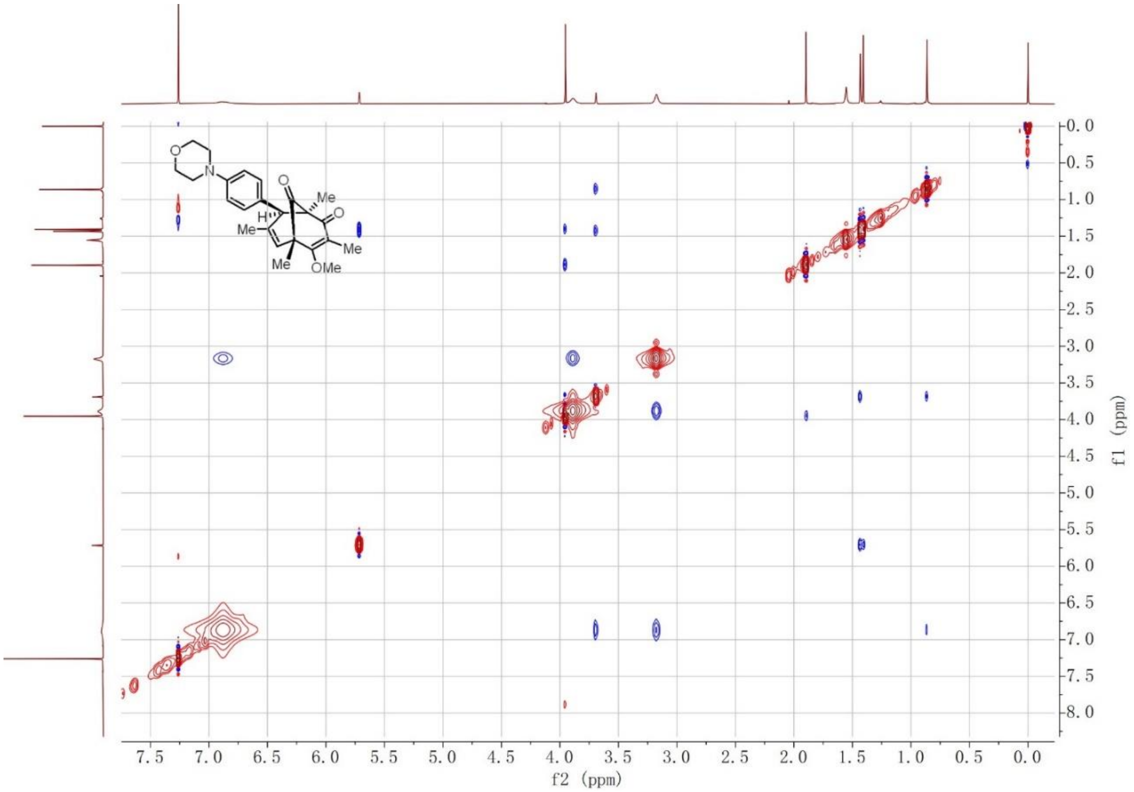

NOESY spectrum of compound **17f** (600 MHz, $CDCl_3$)

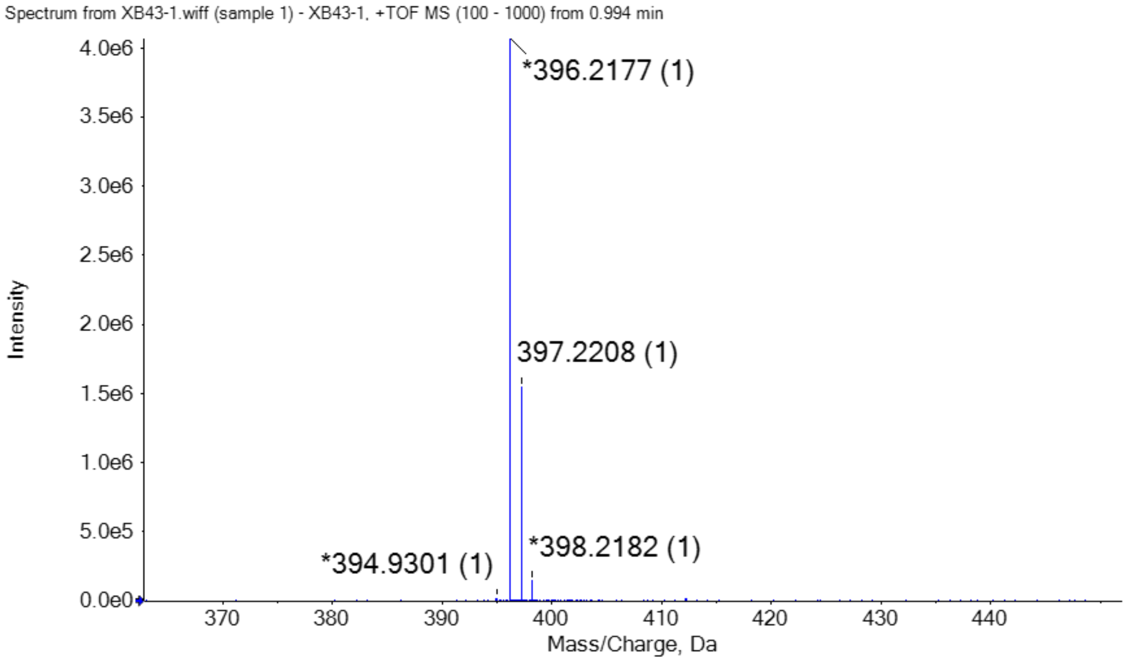


**Formula Calculator Results**

| Measured m/z | Cal m/z | Error(mmu) | Error(ppm) | Ion Formula | Ion |
|---|---|---|---|---|---|
| 396.2177 | 396.2169 | 0.8 | 1.9 | C24H30NO4 | $[M+H]^+$ |

HRESIMS spectrum of compound **17f**

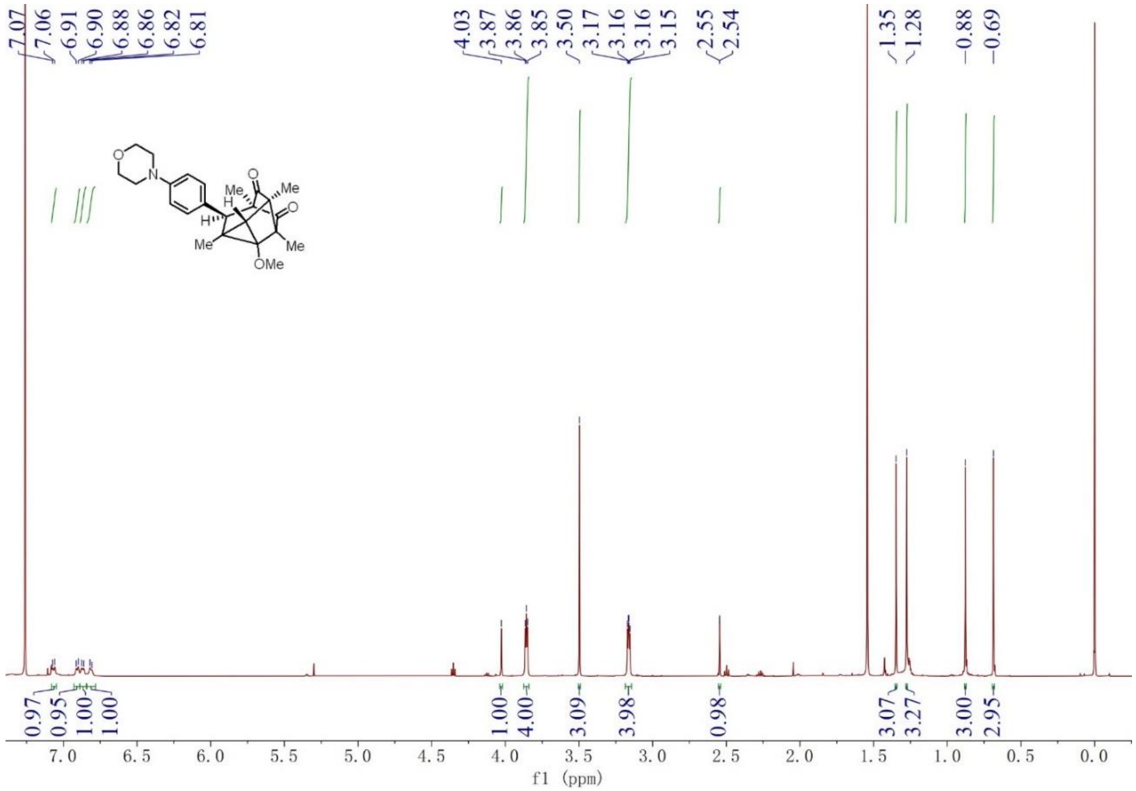


$^1$H NMR spectrum of compound **18b** (600MHz, $CDCl_3$)

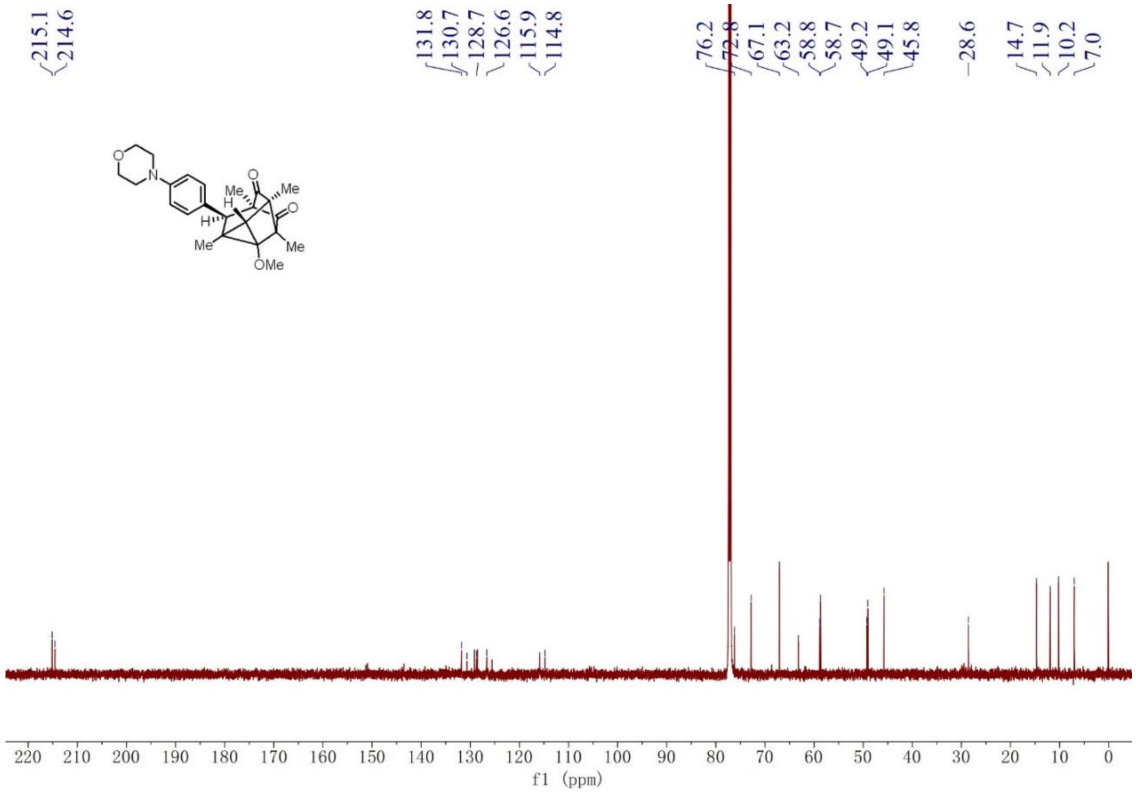


$^{13}$C NMR spectrum of compound **18b** (151MHz, $CDCl_3$)

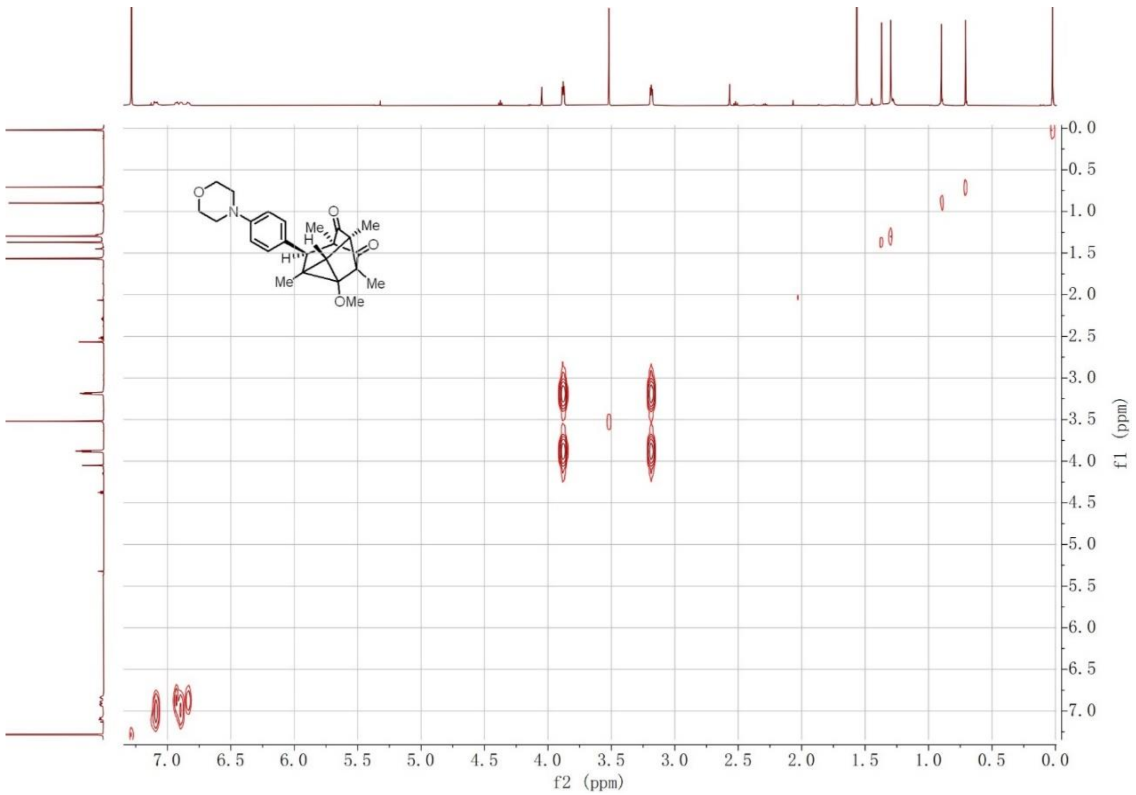


$^{1}$H-$^{1}$H COSY spectrum of compound **18b** (600 MHz, $CDCl_3$)

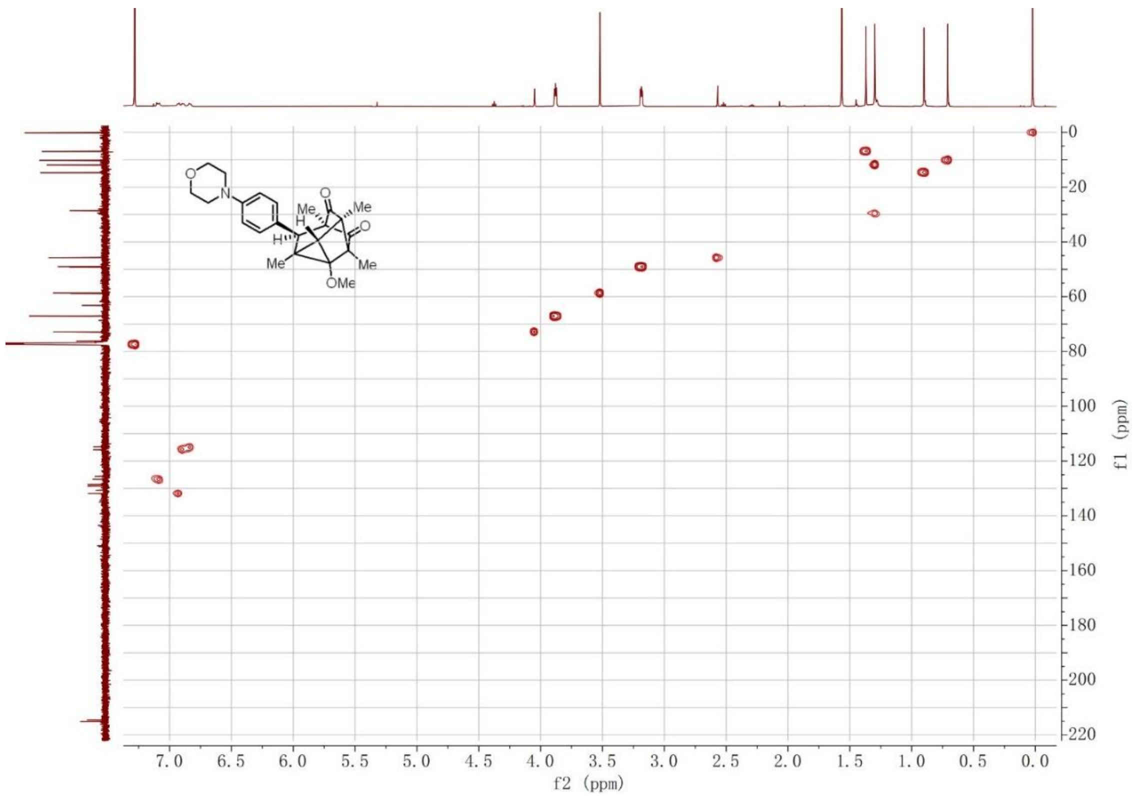


HSQC spectrum of compound **18b** (600 MHz, $CDCl_3$)

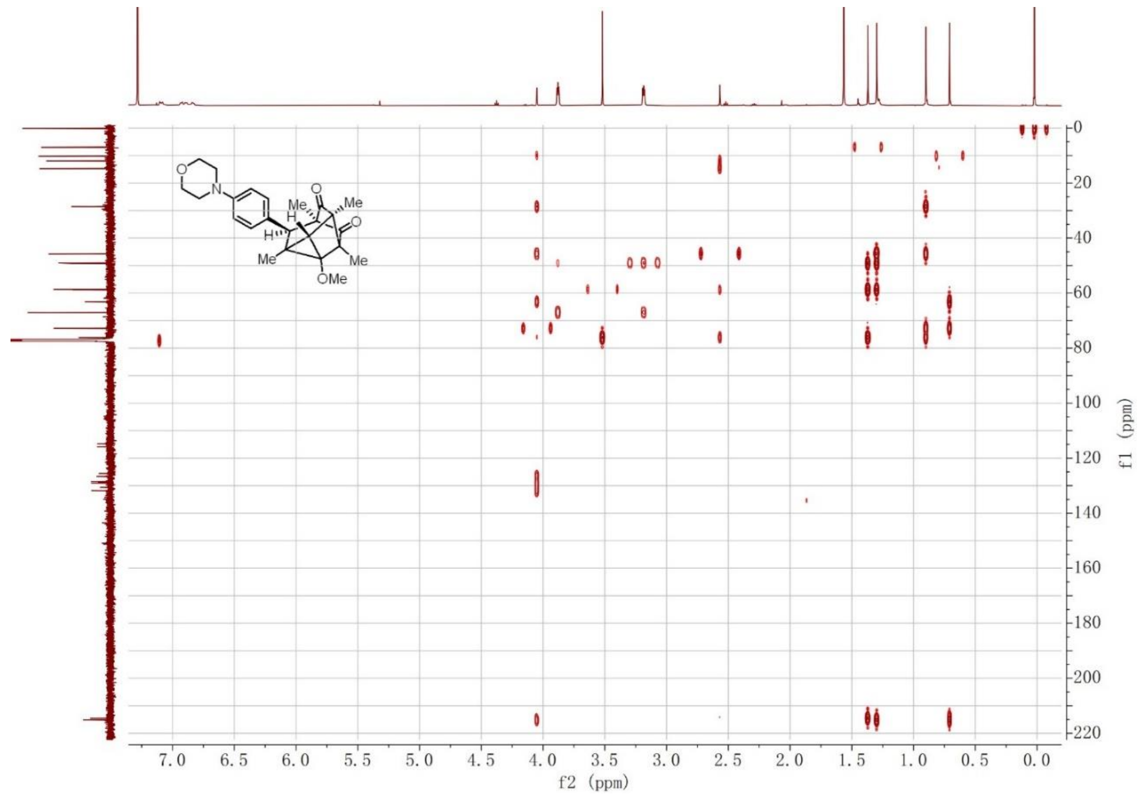


HMBC spectrum of compound **18b** (600 MHz, $CDCl_3$)

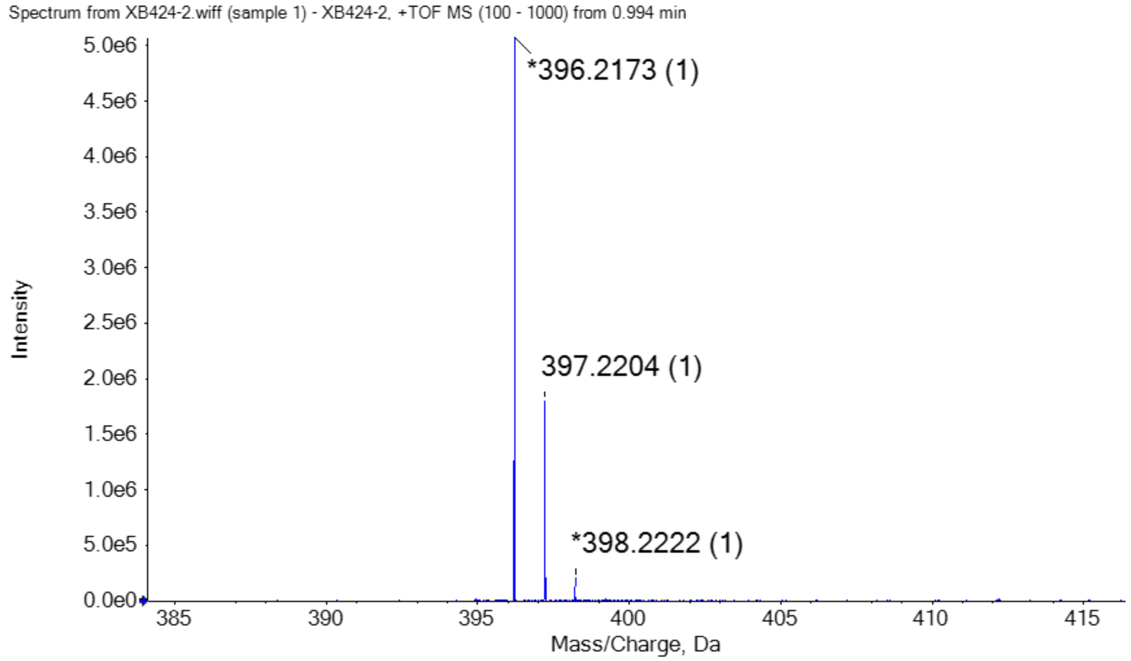


**Formula Calculator Results**

| Measured m/z | Cal m/z | Error(mmu) | Error(ppm) | Ion Formula | Ion |
|---|---|---|---|---|---|
| 396.2173 | 396.2169 | 0.4 | 0.9 | C24H30NO4 | $[M+H]^+$ |

HRESIMS spectrum of compound **18b**

**Other Supplementary Materials for this manuscript include the following:**

Crystallographic data for compounds **11a**, **11d**, **11t**, **12**, **13**, **14**, **15**, and **16** have been deposited at the Cambridge Crystallographic Data Centre (CCDC) under accession numbers CCDC 2504235, 2504237, 2504239, 2504240, 2504241, 2504242, 2504243, and 2504248, respectively. Copies of the data can be obtained free of charge via the CCDC website (www.ccdc.cam.ac.uk/data_request/cif).

## REFERENCES

1. A. R. Leach, V. J. Gillet, R. A. Lewis, R. Taylor, Three-dimensional pharmacophore methods in drug discovery. *J. Med. Chem.* **53**, 539–558 (2010).

2. Y. X. Li, X. M. Dong, M. N. Yu, H. S. Zhang, M. Eginligil, Y. J. Nie, L. H. Xie, Z. Q. Lin, J. Q. Liu, W. Huang, 3D steric bulky semiconductor molecules toward organic optoelectronic nanocrystals. *ACS Mater. Lett.* **3**, 1799–1818 (2021).

3. L. Feng, X. Wang, X. Guo, L. Shi, S. Su, X. Li, J. Wang, N. Tan, Y. Ma, Z. Wang, Identification of novel target DCTPP1 for colorectal cancer therapy with the natural small-molecule inhibitors regulating metabolic reprogramming. *Angew. Chem. Int. Ed. Engl.* **63**, e202402543 (2024).

4. H. Chen, H. Zhang, C. Niu, B. Wang, B. Gao, Z. Liu, G. Yao, H. A. Aisa, Anacyphrethines A and B as potent analgesics: Multiple ion channel inhibitors with an unprecedented chemical architecture. *Acta Pharm. Sin. B.* **15**, 3725–3737 (2025).

5. M. Takemoto, S. Delghandi, M. Abo, K. Yurimoto, M. Odagi, V. P. Singh, J. Wang, R. Nakagawa, S. I. Sato, Y. Takemoto, A. M. A. S. Farrag, Y. Kawaguchi, K. Nagasawa, T. Honjo, K. Chamoto, M. Uesugi, Covalent plant natural product that potentiates antitumor immunity. *J. Am. Chem. Soc.* **147**, 2902–2912 (2025).

6. B. Zhang, J. Zhao, S. Li, H. Liang, X. Hao, Y. Zhang, Ryanodane diterpenes: Occurrence, structural diversity, bioactivities, and synthesis. *Nat. Prod. Rep.* **43**, 132–166 (2026).

7. X. W. Yang, R. B. Grossman, G. Xu, Research progress of polycyclic polyprenylated acylphloroglucinols. *Chem. Rev.* **118**, 3508–3558 (2018).

8. W. Liu, B. Hong, J. Wang, X. Lei, New strategies in the efficient total syntheses of polycyclic natural products. *Acc. Chem. Res.* **53**, 2569–2586 (2020).

9. X. Zhang, E. King-Smith, L. B. Dong, L. C. Yang, J. D. Rudolf, B. Shen, H. Renata, Divergent synthesis of complex diterpenes through a hybrid oxidative approach. *Science* **369**, 799–806 (2020).

10. H. Deng, J. Yang, F. Li, J. Li, H. Renata, Synthesis of diverse terpenoid frameworks via enzyme-enabled abiotic scaffold hop. *Nat. Chem.* **17**, 1275–1283 (2025).

11. T. Okada, P. Thesmar, B. P. Zavesky, R. Sarpong, Total synthesis of dahurelmusin A and epi-dahurelmusin A: Effect of a single stereocenter change on biological activity. *Org. Lett.* **27**, 10231–10234 (2025).

12. F. H. Reis, G. L. Pardo-Andreu, Y. Nuñez-Figueredo, O. Cuesta-Rubio, J. Marín-Prida, S. A. Uyemura, C. Curti, L. C. Alberici, Clusianone, a naturally occurring nemorosone regioisomer, uncouples rat liver mitochondria and induces HepG2 cell death. *Chem. Biol. Interact.* **212**, 20–29 (2014).

13. Q. X. Liu, X. Yuan, J. Ye, R. C. Yue, Y. H. Shen, L. Shan, H. L. Li, W. D. Zhang, Isolation, identification, and bioactivity of microbial metabolites of cyclopamine and its congeners. *Phytochem. Lett.* **12**, 203–208 (2015).

14. R. Bao, H. Zhang, Y. Tang, Biomimetic synthesis of natural products: A journey to learn, to mimic, and to be better. *Acc. Chem. Res.* **54**, 3720–3733 (2021).

15. A. J. E. Novak, C. E. Grigglestone, D. Trauner, A biomimetic synthesis elucidates the origin of preuisolactone A. *J. Am. Chem. Soc.* **141**, 15515–15518 (2019).

16. J. Y. Artzy, D. J. Tantillo, D. H. Trauner, Biomimetic synthesis of azorellolide via cyclopropylcarbinyl cation chemistry. *J. Am. Chem. Soc.* **147**, 78–83 (2025).

17. G. Kang, S. Han, Synthesis of suffranidine B. *J. Am. Chem. Soc.* **145**, 24493–24498 (2023).

18. B. Yin, X. Li, Z.-X. Li, X.-X. Zhu, L. Zhang, X.-L. Zhou, J.-B. Xu, F.-Z. Chen, P. Tang, F. Gao, Adenophorone, an unprecedented sesquiterpene from *Eupatorium adenophorum*: Structural elucidation, bioinspired total synthesis and neuroprotective activity evaluation. *Angew. Chem. Int. Ed. Engl.* **62**, e202306326 (2023).

19. S. Liu, Y. Yang, Q. Song, Z. Liu, Y. Lu, Z. Wang, P. Sivaguru, X. Bi, Tunable molecular editing of indoles with fluoroalkyl carbenes. *Nat. Chem.* **16**, 988–997 (2024).

20. D. Tian, Y. P. He, L. S. Yang, Z. C. Li, H. Wu, Switchable skeletal editing of quinolines enabled by cyclizative sequential rearrangements. *Nat. Chem.* **17**, 952–960 (2025).

21. C. L. Gao, J. Q. Song, Z. N. Yang, H. Wang, X. Y. Wu, C. Shao, H. X. Dai, K. Chen, Y. W. Guo, T. Pang, X. W. Li, Chemoproteomics of marine natural product naamidine J unveils CSE1L as a therapeutic target in acute lung injury. *J. Am. Chem. Soc.* **146**, 28384–28397 (2024).

22. Q. Xu, Y. Q. Du, P. P. Chen, Y. Sun, Z. N. Yang, H. Zhang, B. Tang, H. Wang, J. Li, Y. W. Guo, X. W. Li, Computation assisted chemical study of photo-induced late-stage skeleton transformation of marine natural products towards new scaffolds with biological functions. *Chin. Chem. Lett.* **36**, 110141 (2025).

23. Q. Wu, S.-W. Li, H. Xu, H. Wang, P. Hu, H. Zhang, C. Luo, K.-X. Chen, B. Nay, Y.-W. Guo, X.-W. Li, Complex polypropionates from a South China Sea photosynthetic mollusk: Isolation and biomimetic synthesis highlighting novel rearrangements. *Angew. Chem. Int. Ed. Engl.* **59**, 12105–12112 (2020).

24. A. Sanchez, T. J. Maimone, Taming shapeshifting anions: Total synthesis of ocellatusone C. *J. Am. Chem. Soc.* **144**, 7594–7599 (2022).

25. A. Sanchez, A. Gurajapu, W. Guo, W. Y. Kong, C. J. Laconsay, N. S. Settineri, D. J. Tantillo, T. J. Maimone, A shapeshifting roadmap for polycyclic skeletal evolution. *J. Am. Chem. Soc.* **145**, 13452–13461 (2023).

26. M. Zhu, C. Zheng, Post-spin crossing dynamics determine the regioselectivity in open-shell singlet biradical recombination. *Org. Chem. Front.* **9**, 995–1003 (2022).

27. G. Sartori, F. Bigi, D. Baraldi, R. Maggi, G. Casnati, X. Tao, New direct synthesis of persubstituted 4-hydroxy-2-pyrones. *Synthesis* **1993**, 851–852 (1993).

28. R. Rodriguez, R. M. Adlington, S. J. Eade, M. W. Walter, J. E. Baldwin, J. E. Moses, Total synthesis of cyercene A and the biomimetic synthesis of (±)-9,10-deoxytridachione and (±)-ocellapyrone A. *Tetrahedron* **63**, 4500–4509 (2007).

29. P. Sharma, B. Lygo, W. Lewis, J. E. Moses, Biomimetic synthesis and structural reassignment of the tridachiahydropyrones. *J. Am. Chem. Soc.* **131**, 5966–5972 (2009).

30. H. F. Motiwala, A. M. Armaly, J. G. Cacioppo, T. C. Coombs, K. R. K. Koehn, V. M. Norwood, J. Aubé, HFIP in organic synthesis. *Chem. Rev.* **122**, 12544–12747 (2022).

31. L. Hu, M. Rombola, V. H. Rawal, Synthesis of 1,2-oxazinanes via hydrogen bond mediated [3 + 3] cycloaddition reactions of oxyallyl cations with nitrones. *Org. Lett.* **20**, 5384–5388 (2018).

32. A. N. V. Satyanarayana, T. Chatterjee, HFIP-mediated, highly chemo-, regio-, and stereoselective hydrofunctionalizations of ynamides: Access to stereodefined alkenes bearing drugs and natural products. *J. Org. Chem.* **89**, 12439–12451 (2024).

33. Q. Xu, B. Y. Yan, H. F. Wang, Q. Wu, X. W. Li, X. Zhang, Total synthesis of nocarterphenyl A, 2-dehydroxymethylnocarterphenyl A and nocarterphenyl D produced by marine *Nocardiopsis* spp. *J. Nat. Prod.* **88**, 2710–2718 (2025).

34. P. Sharma, N. Griffiths, J. E. Moses, Biomimetic synthesis and structural revision of (±)-tridachiahydropyrone. *Org. Lett.* **10**, 4025–4027 (2008).

35. E. Ji, H. Meng, Y. Zheng, V. Ramadoss, Y. Wang, Copper-catalyzed stereospecific hydroboration of internal allylic alcohols. *Eur. J. Org. Chem.* **2019**, 7367–7371 (2019).

36. H. Zhang, B. Gao, Y. Feng, G. Zheng, Z. Liu, L. Kong, L. Liu, J. Liu, H. A. Aisa, G. Yao, DFT calculations and dynamic NMR revealed the coalescent NMR phenomena of the 6/6/6/9 tetracyclic merosesquiterpenoids with an unprecedented 9,15-dioxatetracyclo[8.5.3.$0^{4,17}$.$0^{14,18}$] octadecane core skeleton. *Chin. Chem. Lett.* **36**, 111234 (2025).

37. J. M. Burns, E. D. Boittier, Pathway bifurcation in the (4 + 3)/(5 + 2)-cycloaddition of butadiene and oxidopyrylium ylides: The significance of molecular orbital isosymmetry. *J. Org. Chem.* **84**, 5997–6005 (2019).

38. M. Pu, C. D. Nielsen, E. Senol, T. Sperger, F. Schoenebeck, Post-transition-state dynamic effects in the transmetalation of Pd(II)-F to Pd(II)-$CF_3$. *JACS Au* **4**, 263–275 (2024).

39. T. Rigotti, R. Mas-Ballesté, J. Alemán, Enantioselective aminocatalytic [2 + 2] cycloaddition through visible light excitation. *ACS Catal.* **10**, 5335–5346 (2020).

40. Z. C. Girvin, L. F. Cotter, H. Yoon, S. J. Chapman, J. M. Mayer, T. P. Yoon, S. J. Miller, Asymmetric photochemical [2 + 2] cycloaddition of acyclic vinylpyridines through ternary complex formation and an uncontrolled sensitization mechanism. *J. Am. Chem. Soc.* **144**, 20109–20117 (2022).

41. N. Tanaka, Y. Yano, Y. Tatano, Y. Kashiwada, Hypatulins A and B, meroterpenes from *Hypericum patulum*. *Org. Lett.* **18**, 5360–5363 (2016).

42. A. Thakur, J. Louie, "Vinylcyclopropane–cyclopentene rearrangement," in *Molecular Rearrangements in Organic Synthesis* (John Wiley & Sons Ltd., 2015), pp. 323–362.

43. M. J. Frisch, G. W. Trucks, H. B. Schlegel, G. E. Scuseria, M. A. Robb, J. R. Cheeseman, G. Scalmani, V. Barone, G. A. Petersson, H. Nakatsuji, X. Li, M. Caricato, A. V. Marenich, J. Bloino, B. G. Janesko, R. Gomperts, B. Mennucci, H. P. Hratchian, J. V. Ortiz, A. F. Izmaylov, J. L. Sonnenberg, D. Williams-Young, F. Ding, F. Lipparini, F. Egidi, J. Goings, B. Peng, A. Petrone, T. Henderson, D. Ranasinghe, V. G. Zakrzewski, J. Gao, N. Rega, G. Zheng, W. Liang, M. Hada, M. Ehara, K. Toyota, R. Fukuda, J. Hasegawa, M. Ishida, T. Nakajima, Y. Honda, O. Kitao, H. Nakai, T. Vreven, K. Throssell, J. A. Montgomery Jr., J. E. Peralta, F. Ogliaro, M. J. Bearpark, J. J. Heyd, E. N. Brothers, K. N. Kudin, V. N. Staroverov, T. A. Keith, R. Kobayashi, J. Normand, K. Raghavachari, A. P. Rendell, J. C. Burant, S. S. Iyengar, J. Tomasi, M. Cossi, J. M. Millam, M. Klene, C. Adamo, R. Cammi, J. W. Ochterski, R. L. Martin, K. Morokuma, O. Farkas, J. B. Foresman, D. J. Fox, Gaussian 16, Gaussian Inc. (2019); https://scholar.google.com/scholar_lookup?hl=en&publication_
